# Transcriptional changes and preservation of bone mass in hibernating black bears

**DOI:** 10.1038/s41598-021-87785-9

**Published:** 2021-04-15

**Authors:** Anna V. Goropashnaya, Øivind Tøien, Thiruvarangan Ramaraj, Anitha Sundararajan, Faye D. Schilkey, Brian M. Barnes, Seth W. Donahue, Vadim B. Fedorov

**Affiliations:** 1 Institute of Arctic Biology, University of Alaska Fairbanks, 2140 Koyukuk Dr., Fairbanks, AK 99775 USA; 2 School of Computing, DePaul University, Chicago, IL USA; 3 National Center for Genome Resources, 2935 Rodeo Park Dr. East, Santa Fe, NM 87505 USA; 4 Department of Biomedical Engineering, University of Massachusetts, 240 Thatcher Road, Amherst, MA 01003 USA

**Keywords:** Transcriptomics, Bone, Bone quality and biomechanics

## Abstract

Physical inactivity leads to losses of bone mass and strength in most mammalian species. In contrast, hibernating bears show no bone loss over the prolonged periods (4–6 months) of immobility during winter, which suggests that they have adaptive mechanisms to preserve bone mass. To identify transcriptional changes that underlie molecular mechanisms preventing disuse osteoporosis, we conducted a large-scale gene expression screening in the trabecular bone and bone marrow, comparing hibernating and summer active bears through sequencing of the transcriptome. Gene set enrichment analysis showed a coordinated down-regulation of genes involved in bone resorption, osteoclast differentiation and signaling, and apoptosis during hibernation. These findings are consistent with previous histological findings and likely contribute to the preservation of bone during the immobility of hibernation. In contrast, no significant enrichment indicating directional changes in gene expression was detected in the gene sets of bone formation and osteoblast signaling in hibernating bears. Additionally, we revealed significant and coordinated transcriptional induction of gene sets involved in aerobic energy production including fatty acid beta oxidation, tricarboxylic acid cycle, oxidative phosphorylation, and mitochondrial metabolism. Mitochondrial oxidation was likely up-regulated by transcriptionally induced AMPK/PGC1α pathway, an upstream stimulator of mitochondrial function.

## Introduction

Suppressed metabolism for the conservation of metabolic energy is the hallmark of hibernation^1,2^. Bone metabolism is a metabolically expensive process that is substantially reduced during hibernation^3–5^. However, bone is also an organ that plays an essential role in integrative organismal physiology by maintaining calcium homeostasis. The maintenance of normal calcium concentrations in blood, extracellular fluid, and cytosol is critical to maintaining essential physiologic functions needed to survive hibernation, such as heart beat and skeletal muscle contraction for respiration and thermogenesis. Most (99%) of calcium in the body is stored in bone as hydroxyapatite mineral crystals. Calcium ions are readily exchanged between blood and bone by the continuous bone remodeling process in which osteoclasts resorb bone releasing calcium from the bone extracellular matrix, which can be taken up into blood vessels. This is normally countered by osteoblasts rebuilding bone (i.e., putting calcium ions back into bone matrix)^6^. Serum calcium concentration is preserved in hibernating bears when they do not drink, eat, urinate or defecate (i.e., excrete calcium)^5,7^. It is likely bone remodeling is suppressed in bears during hibernation to conserve energy, and that bone resorption and formation are balanced to preserve eucalcemia during anuria^5,8^. These changes would uniquely prevent disuse osteoporosis in mammalian hibernators by preserving the mechanical properties of bone material and whole bone, unlike the bone loss, reduced mechanical properties, and increased fracture risk that occurs with disuse in non hibernators including humans^9–16^. Elucidating the biological mechanisms that prevent bone loss in hibernating bears may lead to novel therapies for treating osteoporosis in humans^17–19^.

Hibernating bears show transcriptional changes in adipose, liver, heart and skeletal muscle that are consistent with reduced metabolism and energy expenditure, as well as the preservation of skeletal muscle^20–23^. While there is some common transcriptional regulation of metabolism in tissues and organs, these studies demonstrated, tissue specific gene networks also exist in hibernating bears. Bone metabolism presents a conundrum to hibernators: it is energetically expensive but essential for organismal calcium homeostasis and preservation of bone mass and strength. Systemic markers of bone resorption (− 25%) and formation (− 55%) are decreased during hibernation relative to summer^5^, but not to the level of organismal metabolic reduction (-75%) during hibernation relative to summer^2^. Mitochondrial oxidative metabolism is the main energy source for osteoclastogenesis, and glycolysis provides the main source for bone resorption by osteoclasts^24^. Fully differentiated osteoclasts have higher mitochondrial oxygen consumption and higher levels of enzymes involved in electron transport than do immature cells. Bone forming osteoblasts also utilize different energy sources depending on their differentiation stage^25^. There has been good progress on identifying the biological mechanisms that regulate bone metabolism in bears and other hibernators^26–30^. However, the transcriptional mechanisms underlying bone preservation and remodeling remain poorly understood. The first genomic study of transcriptional changes identified 241 differentially expressed genes in trabecular bone contrasting hibernating and non-hibernating bears^31^. The gene set enrichment analyses suggested increased anabolic bone activity, and three genes involved in bone resorption were significantly down regulated during hibernation. However, the low genome coverage and absence of cDNA probes representing the bone transcriptome specifically on the custom microarray used in that study provided limited insight into transcriptional changes at the pathway level.

In the present study to increase genome coverage we sequence transcriptomes and quantify gene expression changes in trabecular bone of hibernating black bears (*Ursus americanus*) in comparison to summer active bears. Genome wide expression data allows for conducting gene set enrichment analyses to identify functional sets of co-regulated genes and reveal how transcriptional changes may influence bone during hibernation. We also selected differentially expressed genes with known functional associations to bone remodeling and osteoporosis in non-hibernating mammals and considered these transcriptional changes in light of bone preservation during the disuse that occurs during hibernation.

## Results

On average 48 million paired-end 150 base pair sequencing reads were generated for each sample, 87–93% were mapped in pairs, and 72–80% of paired reads were mapped to exons of the brown bear reference genome. These results of mapping RNA-seq reads to a reference genome of a closely related species were appropriate for genome scale screening of transcriptional changes^32^.

### Differentially expressed genes

Transcriptome sequencing detected expression of 13,590 genes in all samples. Of these, 5404 were differentially expressed between hibernation and summer seasons (Table 1S). There were 2466 differentially expressed up-regulated genes and 2938 down-regulated genes during hibernation relative to summer.

### Gene set enrichment analysis

Gene set enrichment analysis (GSEA) identifies functional groups of co-regulated genes by estimating the significance of differences between observed direction of expression changes (up- or down-regulated) among genes involved in biological processes or pathways and expression changes expected by chance. Gene Ontology biological process categories of bone remodeling, bone cell development and mineralization were significantly enriched by down-regulated genes (Table 1). Similarly, the gene sets involved in bone resorption (Table 3S) and osteoclast differentiation and signaling were significantly underexpressed during hibernation relative to summer. It should be noted, that genes involved in bone resorption (48 genes, Table 1) are also members of bone remodeling category (71 genes, Table 1), thus, down-regulation of bone resorption genes contributes to overall negative enrichment of bone remodeling. Macrophage colony stimulating factor signaling pathway that promotes osteoclast differentiation was also down-regulated. Notably, the gene sets related to bone loss, osteoclast resorption, and osteoporosis that were reported in non hibernating mammalian models were underexpressed in hibernating bears relative to summer. In contrast to bone resorption and osteoclast activity, no significant enrichment (FDR > 0.05) indicating directional changes in gene expression was detected in the gene sets of ossification (Table 4S) or osteoblast signaling and differentiation between seasons (Table 1). Surprisingly, given the metabolic suppression that occurs during hibernation^2^, a number of gene sets involved in aerobic energy production and mitochondrial metabolism were up-regulated in hibernation compared to summer (Table 2). The highest normalized enrichment scores (NES) were observed for the oxidative phosphorylation and respiratory electron transport gene sets. Up-regulated genes were also over-represented in the fatty acid betta oxidation category that provides acetyl-CoA for transcriptionaly induced tricarboxylic acid cycle generating NADH as substrate for oxidative phosphorylation. A suite of gene sets involved in mitochondrial metabolism and biogenesis and turnover demonstrated significant transcriptional induction during hibernation (Table 2). In contrast to aerobic energy production, the glycolysis gene set was significantly underexpressed.

**Table 1:** Gene set enrichment for selected Gene Ontology (GO) biological processes, Biocarta (B), Hallmark (H), BioPlanet (BP), OMIM and Elsevier Pathway gene sets involved in bone homeostasis in bone of black bears during hibernation. Positive NES (normalized enrichment score) values indicate elevated proportion of overexpressed genes and negative scores indicate excess of under expressed genes during hibernation relative to summer. FDR is the false discovery rate.

Category	#Genes	NES	FDR
Bone remodeling GO	71	− 2.47	0.002
Bone cell development GO	30	− 2.60	< 0.001
Bone mineralization involved in bone maturation GO	8	− 1.97	0.030
Bone resorption GO	48	− 2.87	< 0.001
Osteoclast differentiation GO	69	− 1.82	0.058
Osteoclast signaling BP	13	− 1.64	0.033
Macrophage colony stimulating factor signaling pathway GO	5	− 1.87	0.047
Bone loss in osteoporosis EP	27	− 1.61	0.035
Bone resorption by osteoclast in osteoporosis EP	6	− 1.68	0.028
Osteoporosis OMIM	5	− 1.87	0.006
Osteoclasts function in osteopetrosis EP	18	− 2.18	0.004
Ossification GO	292	− 1.45	0.220 ns
Ossification involved in bone remodeling GO	5	− 0.60	0.980 ns
Osteoblast differentiation GO	165	− 1.52	0.167 ns
Osteoblast signaling BP	88	− 1.04	0.400 ns
Osteoblast decline in osteoporosis EP	29	0.66	0.900 ns
Apoptosis H	138	− 2.73	< 0.001
Caspase pathway B	17	− 2.02	0.025
Canonical Wnt signaling GO	270	2.15	0.020
Myogenesis H	168	6.03	< 0.001

**Table 2:** Gene set enrichment for selected Gene Ontology (GO) biological processes, Hallmark (H), Reactome (R) and KEGG (K) sets involved in metabolism, mitochondrial oxidation and biogenesis, and immunity in bone of black bears during hibernation. Positive NES (normalized enrichment score) values indicate elevated proportion of overexpressed genes and negative scores indicate excess of under expressed genes during hibernation relative to summer. FDR is the false discovery rate.

Category	#Genes	NES	FDR
Fatty acid beta oxidation GO	65	2.56	0.001
Tricarboxylic acid cycle GO	31	3.50	< 0.001
Glycolysis H	166	− 1.80	0.029
Oxidative phosphorylation H	165	7.12	< 0.001
Aerobic respiration GO	55	3.91	< 0.001
Respiratory electron transport R	61	6.90	< 0.001
Electron transport reaction in mitochondria B	9	2.42	0.012
Mitochondrial electron transport nadh to ubiquinone GO	46	6.43	< 0.001
Mitochondrial respiratory chain complex assembly GO	70	6.18	< 0.001
Mitochondrial fatty acid beta oxidation of saturated acids R	10	2.08	0.020
Mitochondrial Ca^+^ ion transport R	23	2.19	0.010
Mitochondrial protein import R	56	4.57	< 0.001
Mitochondrial biogenesis R	86	2.82	< 0.001
Mitophagy R	27	2.95	< 0.001
Mitochondrial translation GO	130	6.32	< 0.001
Mitochondrial gene expression GO	156	6.46	< 0.001
Inner mitochondrial membrane organization GO	42	4.18	< 0.001
Mitochondrial transmembrane transport GO	83	3.26	< 0.001
Go mitochondrial membrane organization GO	118	2.91	< 0.001
Activation of immune response GO	424	− 4.48	< 0.001
Innate immune response GO	558	− 5.61	< 0.001
Adaptive immune response GO	244	− 5.57	< 0.001
Leukocyte mediated immunity GO	558	− 7.39	< 0.001
Myeloid leukocyte mediated immunity GO	430	− 6.99	< 0.001

GSEA identified other gene sets significantly enriched by genes with coordinated changes in expression (Tables 1, 2; Table 2S). During hibernation, down regulated genes were over represented in the gene sets involved in apoptosis, caspase pathway, and innate, adaptive and leukocyte mediated immunity. Significant enrichment by up-regulated genes was detected for myogenesis and canonical Wnt signaling pathways.

### Differential expression of selected genes

**Figure 1:**
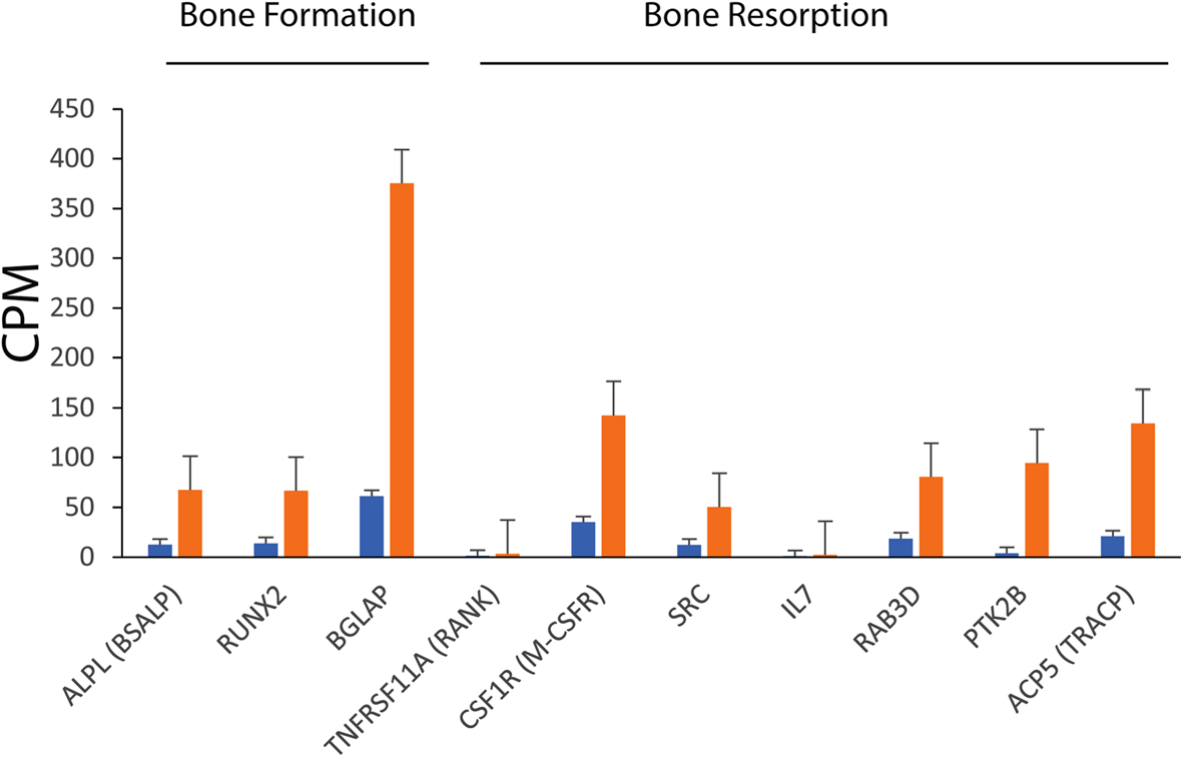
Normalized expression values of selected differentially expressed genes involved in bone homeostasis in hibernating (blue bars) and summer active (red bars) bears. CPM—the mean read count per million RNA-seq reads and its standard deviation.

**Figure 2:**
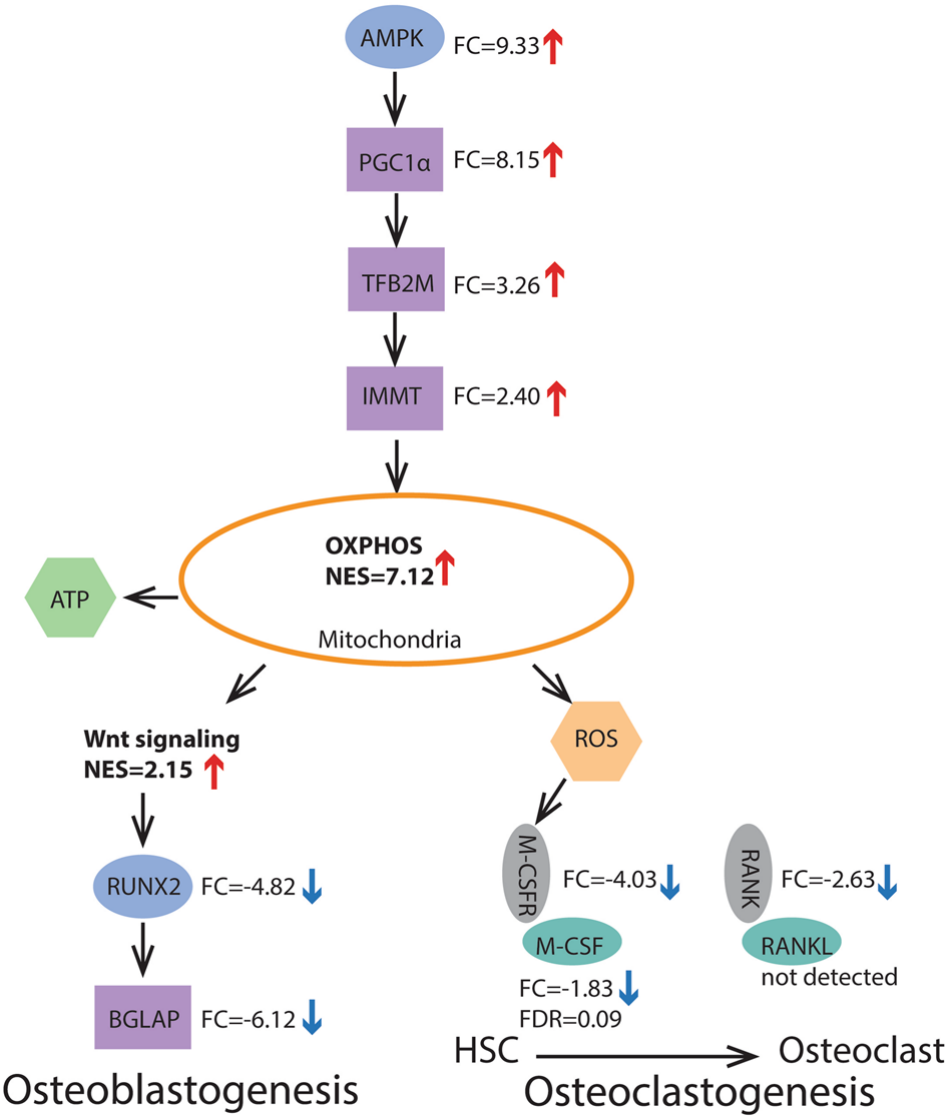
The AMPK/PGC1α stimulated mitochondrial oxidation (OXPHOS) in the regulation of mesenchymal stem cells osteoblastogenesis and osteoclastogenesis from hematopoietic stem cells (HSC)^33^ in the bone/bone marrow of hibernating bears. Blue ovals—upstream stimulators, purple rectangulars—nuclear/cytoplasmic communicators, green ovals—master mediators of osteoclastogenesis and grey ovals—receptors of the master mediators. FC—fold change in expression of gene during hibernation, red arrow—up-regulation, blue arrow—down-regulation. NES—GSEA normalized enrichment score (Tables 1, 2). M-CSF demonstrated tendency (FDR = 0.09) for down-regulation and expression of RANKL was not detected in the data set. ROS - reactive oxidative species.

In addition to the gene set enrichment analysis, we also considered significant expression differences (FDR < 0.05; Fig. 1, Table 1S) for individual genes known to be important for bone homeostasis and activation of mitochondrial metabolism. The bone formation marker, ALPL (also known as BSALP; FC = – 5.44) and bone resorption marker, ACP5 (also known as TRACP; FC = − 6.48) were both down-regulated at the transcriptional level during hibernation. Transcription factor RUNX2 (FC = − 4.82) and its down stream target, osteocalcin BGLAP (FC = − 6.12), activating osteoblast differentiation and bone formation were also underexpressed (Fig. 2). Several key genes that enchance bone resorption demonstrated transcriptional suppression in hibernating bears (Fig. 1). The downregulated genes involved in osteoclast differentiation and signaling include osteoclast differentiation factor receptor TNFRSF11A (also known as RANK; FC = − 2.63), colony stimulating factor 1 receptor CSF1R (also known as M-CSFR; FC = − 4.03) and osteoclast stimulating factor 1 OSTF1 (FC = − 3.95).

In contrast to reduced expression of bone remodeling genes, elevated expression was detected for members of the AMPK/PGC1α pathway (Fig. 2), which acts as upstream master stimulator of mitochondrial biogenesis and metabolism^33^. Overexpressed AMP protein kinase PRKAA2 (also known as AMPK; FC = 9.33) enhances mitochondrial biogenesis and respiration through induction of transcriptional co-activator, PPARGC1A (also known as PGC1α, FC = 8.15), mitochondrial transcription factor TFB2M (FC = 3.26), and the core component of the mitochondrial contact site, mitofilin IMMT (also known as Mic60; FC = 2.40). In addition, another gene, PPARGC1B (also known as PGC1β; FC = 6.55), promoting mitochondrial oxidative metabolism, was also up regulated during hibernation.

## Discussion

Physical inactivity leads to disuse osteoporosis in humans^34,35^ and in animal model species^36–40^. Bears demonstrate an exceptional ability to preserve bone mass over 4–6 months of immobility during winter hibernation^4,10–13,15^. Our study reveals coordinated transcriptional changes in genes related to bone remodeling and homeostasis that likely contribute to the prevention of disuse osteoporosis in hibernating bears by reducing bone turnover. These changes include the down-regulation of genes involved in bone resorption and remodeling as well as transcriptional reduction of apoptotic genes. Transcriptional reduction of both osteoblastic bone formation marker BSALP and osteoclastic resorption marker TRACP reported here is consistent with the significant decrease in the serum concentrations of BSALP and TRACP of hibernating black bears^5^. At the pathway level, elevated proportion of down-regulated genes involved in bone remodeling suggests suppression of bone turnover during hibernation, which is consistent with histological evidence showing decreased, but balanced, resorption and formation activity in cortical and trabecular bone in hibernating bears^4,16^.

In mammals, disuse induced bone loss results from unbalanced remodeling involving decreased bone formation and/or increased resorption^14^. Although several key genes (BSALP, RUNX2, BGLAP) involved in osteoblastic activity and bone formation were significantly down-regulated during hibernation (Fig. 1), we did not detect at the pathway level significant coordinated directional changes in transcription of genes involved in bone formation (ossification) and osteoblast signaling. We found significant transcriptional reduction of bone resorption and osteoclast signaling gene sets (Table 1). This reduction, as well as down-regulation of genes involved in bone resorption related to osteoporosis (Table 1), suggest suppression of bone resorption during immobility of hibernation. Decrease in expression of bone resorption genes likely counteracts the mechanisms responsible (e.g., osteocyte apoptosis^35–40^) for the induced osteoclastogenesis and bone resorption normally observed in mammals under disuse conditions.

Similar to bone resorption, sets of genes involved in apoptosis and caspase pathways were significantly enriched by down-regulated genes during hibernation. Osteocyte apoptosis and resulting bone loss are increased in cortical and trabecular bones under unloading in mice models^36,38^. Coordinated transcriptional suppression of apoptosis genes implies a decrease in apoptotic activity that promotes osteocyte survival and potentially reduces bone loss by preventing the activation of osteoclasts to target apoptotic osteocytes^37,40^. Transcriptional induction of key components in anti-apoptotic AMPK/PGC1α pathway^41^ reported here (Fig. 2) likely suppresses apoptosis in bone of hibernating bears. Additionally, a reduction in disuse-induced osteoblast apoptosis was suggested to support bone formation during hibernation^42^. Significant (FDR < 0.001) down-regulation of the two caspase genes (CASP3, FC = − 3.43 and CASP7, FC = − 3.77) is consistent with decline in caspase 3/7 activity detected in MC3T3-E1 osteoblast culture treated with hibernaton serum compared to serum from physically active black bears^42^. Furthermore, peripheral blood mononuclear cells treated with summer bear serum promoted the formation of multinuclear cells and high TRACP activity^43^. In contrast, hibernating bear serum failed to promote the formation of multinuclear cells and TRACP activity was more than tenfold lower compared to treatment with summer serum. Taken together, these findings raise the possibility that serum factors possibly reduce osteoblast apoptosis and maintain osteoblast activity when challeneged with reduced mechanical loading. They also support the idea that changes in circulating factors during hibernation may reduce osteocyte apoptosis and bone resoprtion during hibernation.

Reactive oxidgen species (ROS), byproducts of oxidative phosphorylation elevated in hibernating bears, amplify osteoclast differentiation from hematopoetic stem cells in the bone marrow and enchance bone resorption through activation of macrophage colony stimulating factor signaling and RANKL induced osteoclastogenesis^33^. However, transcriptional reduction of macrophage colony stimulating factor signaling pathway including its key component, CSF1R (also known as M-CSFR), and underexpression of RANK (also known as TNFRSF11A), master activator of RANKL pathway (Figs. 1, 2), provide no support for increase in ROS accelerated osteoclastogenesis and bone resorption during hibernation. ROS accumulation and associated oxidative stress are restrained by a major antioxidant, the resident manganese superoxide dismutase, SOD2^33^, which tends to elevate in expression (FC = 2.45; FDR = 0.06) in hibernating bears.

Hibernation is an energy conserving strategy associated with a 20–75% reduction in whole animal metabolic rate in bears^2^. Unanticipated under metabolic suppression, our study revealed significant and coordinated transcriptional induction of gene sets involved in aerobic energy production in bone marrow/bone of hibernating bears. Down-regulation of the glycolysis gene set supports a fuel shift from carbohydrates to lipids. Up-regulated gene sets include fatty acid beta oxidation providing acetyl-CoA for transcriptionaly induced tricarboxylic acid cycle generating NADH as substrate for elevated oxidative phosphorylation resulting in ATP production (Table 2). Transcriptional up-regulation of the fatty acid beta oxidation was previously reported in liver of hibernating bears^21^ and it is consistent with the consumption of stored fat as the primary energy source in winter^44^. Bone marrow adipocytes are suggested to mobilize fatty acids to yield acetyl-CoA via beta oxidation which can produce ATP by oxidative phosphorylation^45^. Bone marrow adiposity substantially increases during hibernation^3,26^. Thus, our findings of upregulated genes involved in beta-oxidation raise the possibility that marrow adipocytes provided an additional energy source for bone cells to promote organismal survival during the long winter season when food is unavalaiable. However, transcriptional induction of aerobic respiration gene sets detected here is tissue specific for the bone marrow/trabecular bone. Consistent to metabolic suppression, transcriptional reduction was observed for genes involved in cellular respiration and oxidation—reduction in liver and muscle of black bears^21,22^ as well as down-regulation of key genes in tricarboxylic acid cycle and oxidative phosphorylation in liver and muscle of hibernating grizzly bears^23^.

Mitochondria are key organelles for aerobic respiration and oxidative phosphorylation. We found significant transcriptional elevation for a number of gene sets involved in mitochondrial metabolism, biogenesis and turnover during hibernation. Mitochondrial oxidation and biogenesis are up-regulated by transcriptionaly induced AMPK/PGC1α pathway (Fig. 2), upstream master stimulator of mitochondrial function^33^. In addition to aerobic energy production, emerging evidence show an important role of mitochondrial metabolism in regulating bone marrow stem cells differentiation and bone homeostasis^33^. A metabolic shift from glycolysis to mitochondrial oxidative phosphorylation inferred here in the bone marrow/bone of hibernating bears was shown to promote osteoblastogenesis from mesenchymal stem cells of the bone marrow^33^ through up-regulation canonical Wnt signaling pathway, which transcriptionaly activates its down-stream target, runt-related transcription factor 2, RUNX2^46^. Although we detected transcriptional induction of canonical Wnt signaling pathway, the master regulator of osteogenesis RUNX2 was significantly down-regulated during hibernation (Figs. 1, 2). This finding together with the lack of significant enrichment in bone formation gene sets do not support increases in osteogenesis as a result of elevated mitochondrial oxidation in the bone marrow/bone of hibernating bears.

Additionally, we found a coordinated transcriptional reduction of genes involved in immune response including both innate and adaptive immunity that are likely attributed to the bone marrow of hibernating bears. This finding implies suppression of immune system function that is consistent with decrease in blood immune cell counts reported in hibernating brown bears^47,48^ and the transcriptional down-regulation of adaptive immunity described in the bone marrow of hibernating 13-lined squirrels^26^.

One of the limitations with this study is that the samples contained both bone and marrow cells. Therefore, it is unknown how much of the changes in expression can be attributed to cells from each tissue. Reasonable assumption is that transcriptional changes in bone remodeling genes associated with osteoblast and osteoclast signaling are originated mostly from the bone cells. Expression changes of gene sets involved in fatty acid betta oxidation, aerobic respiration, mitochondrial oxidative phosphorylation, the AMPK/PGC1α pathway and immunity are likely attributed to the bone marrow^26,33,45^**.** Future studies using single-cell RNA sequencing^49^ are needed to reveal cell and tissue specific transcriptional profiles.

In conclusion, this study reveals coordinated transcriptional suppression of bone resorption, osteoclast signaling and apoptosis that likely contributes to the preservation of bone mass and structure over the prolonged period of immobility and fasting during winter hibernation. The significance of detected transcriptional induction in mitochondrial oxidation, in addition to its anti-apoptotic effect^41^, for bone maintenance in hibernating bears remains to be elucidated in future studies. The follow up studies are also needed to quantify expression changes at protein level and conduct functional assessments of mitochondrial metabolism and biogenesis. In line with functional genomics paradigms, our inference is based on genome-wide transcriptional changes representing proxies for quantities of proteins involved in physiological processes. Although, post-transcriptional regulation reduces correlation between quantities of transcripts and proteins^50^, our conclusions on functional significance of transcriptional changes are supported by previously reported^5^ changes at protein level for bone remodeling markers (BSALP, TRACP) and histological findings^4,16^ in bone of hibernating bears. To date, no large scale comparison between expression changes at transcript and protein levels is available for bears. For another mammalian hibernator, significant correlation (Pearson’s r = 0.62; P < 0.001) was reported between expression changes at transcript and protein levels comparing hibernating and summer active arctic ground squirrels^51^.

## Material and methods

### Animals

Protocols for all animal work and experiments were approved by the University of Alaska Fairbanks, Institutional Animal Care and Use Committee (IACUC nos. 02-39, 02-44, 05-55, and 05-56). Animal work and experiments were carried out in accordance with IACUC approved animal protocols and in compliance with the ARRIVE guidelines. Animal care and monitoring of physiological conditions of the black bear (*Ursus americanus*) were described previously^2,20,31^. Bears (51–143 kg) were captured in the field by Alaska Department of Fish and Game in May–July and transferred to Fairbanks. Summer-active bears (n = 4) that were feeding and housed in an outdoor enclosure were euthanized and sampled for tissues in June and July. Feeding was stopped 24 h before these animals were euthanized. Bears in the hibernating condition (n = 4) were without food or water since October 27 and euthanized for tissue sampling between March 1 and 26, about 1 month before expected emergence from hibernation. Core body temperature was recorded with radio telemetry^20^. Oxygen consumption and respiratory quotient were monitored in hibernating bears with open flow respirometry by drawing air from the closed dens and through a tracheal tube just prior to euthanasia of summer active animals^20^. In hibernating bears anesthetized before euthanasia, body temperature 33.6 ± 1.0 °C and metabolic rate 0.105 ± 0.012 ml g^−1^ h^−1^. In two summer active bears anesthetized before euthanasia metabolic rates were 0.232 ml g^−1^ h^−1^ (range 0.252–0.213 ml g^−1^ h^−1^) and body temperatures were 37.2 °C^31^. Immediately before tissue sampling, the metabolic rate of anaesthetized hibernating bears was 45.4% of that of anaesthetized summer active animals, and body temperature was 3.6 °C lower. To decrease intragroup variation in gene expression, only males were sampled. Age distribution was similar in hibernating and summer active bears with two adults and two 3 years old bears sampled in each group. Animals were euthanized by an intravenous injection of pentobarbital. Tissue sampling followed immediately with samples placed in liquid nitrogen within 12 min of death. Trabecular bone (illium) cores 80 mm long and 20 mm diameter were cut from the ilium tuber coxae of each animal. Bone and bone marrow were not separated.

### RNA isolation and sequencing

RNA isolation and sequencing were described previously^31,32^. Briefly, trabecular bone together with bone marrow was pulverized in cooled metal cylinder with a piston (made in house) and then transferred into Trizol reagent with 0.1 volume of chloroform. The lizate was centrifuged at 13,000×*g* for 20 min at 4 °C, and a clear aqueous phase was added to 0.5 volume of 2-propanol and left for 10 min at room temperature followed by centrifugation at 13,000×*g* for 20 min at 4 °C and the pellet was washed with ethanol twice and resuspended in RNAse-free water. Additional RNA cleanup including DNase I treatment was performed with the RNeasy kit (Qiagen Inc., Valencia, CA, USA). The RNA quality was assessed with an Agilent 2100 Bioanalyzer and concentration was measured with a Nanodrop ND-1000. The total RNA samples were used for cDNA library construction and sequencing 50 million of 150 nucleotide paired-end reads for each sample on Illumina HiSeq. 4000 system at NCGR (Santa Fe, NM).

### Data analysis

RNA sequences mapping and differential gene expression analysis was done using CLC Genomics Workbench (v12, https://www.qiagenbioinformatics.com). Paired-end sequencing reads were aligned to the reference genome of *Ursus arctos horribilis* (brown bear, NCBI assembly ASM358476v1). After conducting preliminary tests we used the following parameters for the alignment: mismatch cost: 2, insertion cost: 3, deletion cost: 3, similarity fraction: 0.8, length fraction: 0.8, max number of hits for a read: 10. Total of 43,155 transcripts and 19,848 protein coding genes are annotated in the reference genome, so for mapping sequence reads we used the option “Genome annotated with genes and transcripts”. Counts of reads mapped in pairs to the exons represented expression values which were normalized for library size by using the TMM method^52^. The dispersion parameter of normalized read counts for each gene was estimated with the multi-factorial EdgeR method implementing negative binomial Generalized Linear Model^53^. Wald test was applied to compare expression values between hibernating and summer active bears. Only genes with at least 2 paired reads across all samples in a pairwise comparison were included in the analysis. The false discovery rate (FDR) for each gene was estimated using the procedure described by Benjamini and Hochberg^54^. Genes were considered differentially expressed if FDR was 0.05 or less.We estimated enrichment in gene sets corresponding to biological function or metabolic, signaling pathways using Gene Set Enrichment Analysis (http://software.broadinstitute.org/gsea). GSEA assesses overrepresentation of up- or down-regulated genes considering all of the genes with expression detected in an experiment^55^ and both over expressed and under expressed genes were analyzed in the same run to obtain integrative estimate of enrichment. Genes were pre-ranked according to their fold change between their expression values in pairwise comparison. An enrichment score (ES) was calculated to estimate the degree to which genes involved in a gene set were overrepresented at the extremes (up-regulated genes at the top and down-regulated genes at the bottom) of the entire ranked list of genes. The ES was normalized to adjust for the size of the gene sets presented in the experiment, providing a normalized enrichment score (NES). The positive values of the NES indicate enrichment by up-regulated genes and the negative values correspond to elevated proportion of under expressed genes. The statistical significance of the NES was estimated by the false discovery rate using permutation test based on gene set. Gene sets corresponding to biological processes and pathways were obtained from Molecular Signatures Database (http://www.broadinstitute.org/gsea/msigdb/index.jsp) and included the following collections: Gene Ontology Biological Processes, Biocarta, Hallmark, KEGG and Reactome (Table 2S). In addition, we analysed enrichment in several gene sets involved in osteoporosis in mammalian models available from Elsevier Pathway Collection, BioPlanet 2019, OMIM at Enrichr^56^ (https://maayanlab.cloud/Enrichr/#).

## Supplementary Information

Supplementary Tables.

## Data Availability

Transcriptome sequencing data were archived on the NCBI Short Read Archive (Bioproject PRJNA720155).
