# Supplementary material for: Transcriptional changes and preservation of bone mass in hibernating black bears
Source: Sci Rep. 2021 Apr 15;11:8281. doi: 10.1038/s41598-021-87785-9 (PMC8050052; doi:10.1038/s41598-021-87785-9)
Supplement: Supplementary file 1 — Supplementary Tables. [file 41598_2021_87785_MOESM1_ESM.pdf]

Transcriptional changes and preservation of bone mass in hibernating black bears

Anna V. Goropashnaya[1], Øivind Tøien[1], Thiruvarangan Ramaraj[2], Anitha Sundararajan[3], Faye D. Schilkey[3], Brian M. Barnes[1], Seth W. Donahue[4], Vadim B. Fedorov[1]*

[1]Institute of Arctic Biology, University of Alaska Fairbanks, 2140 Koyukuk Dr., Fairbanks, AK 99775, USA

[2]School of Computing, DePaul University, Chicago, IL USA

[3]National Center for Genome Resources, 2935 Rodeo Park Dr. East, Santa Fe, NM 87505, USA

[43]Department of Biomedical Engineering, University of Massachusetts, 240 Thatcher Road, Amherst, MA 01003, USA

*Contact Information: Vadim Fedorov, tel. +1 (907) 474 1533, e-mail: vfedorov@alaska.edu

Table 1S. Differentially expressed genes in bone of hibernating black bears comparing to summer active animals.

Gene Name	Gene ID	Fold Change	Log_2 fold change	P-value	FDR p-value
MYL6B	113256435	162.4740562	7.344066	0	0
KLHL41	113250800	114.9338219	6.84466	2.66E-13	6.91E-12
LMOD2	113263587	113.105562	6.821526	0	0
NRAP	113251946	106.0572481	6.728699	7.77E-16	3.01E-14
LOC113271211	113271211	97.75769862	6.611138	0	0
HSPB3	113263861	93.40003395	6.545351	0	0
EEF1A2	113258809	82.70525339	6.369907	3.37E-12	7.19E-11
SGCA	113265613	82.70160765	6.369843	0	0
PPP1R27	113244502	82.25970373	6.362114	0	0
ABRA	113252637	81.63162839	6.351056	0	0
ANKRD1	113243097	80.25648891	6.326546	0	0
PDLIM3	113262276	76.52717139	6.2579	9.99E-14	2.79E-12
MYOZ2	113255609	76.35136036	6.254582	0	0
LOC113241702	113241702	74.08287073	6.211068	0	0
PTP4A3	113247947	73.01551541	6.190131	0	0
TMOD4	113246165	70.96723559	6.149081	2.72E-11	4.86E-10

CORO6	113269076	68.94525041	6.107379	0	0
ANKRD2	113251786	68.65289234	6.101249	0	0
LRRC2	113256795	68.193157	6.091555	0	0
APOBEC2	113261323	67.8414811	6.084096	6.66E-16	2.62E-14
FBP2	113269786	66.54026372	6.056156	0	0
HSPB7	113270462	66.22117844	6.049221	0	0
CERS1	113256735	65.82250412	6.040509	0	0
ACTA1	113259054	65.34543282	6.030015	4.74E-10	6.77E-09
LRRC39	113254357	64.47340972	6.010632	0	0
ASB5	113262228	64.40407697	6.00908	0	0
MYL2	113267112	64.15830683	6.003564	9.49E-11	1.53E-09
TPM2	113260474	63.96095924	5.99912	2.73E-12	5.9E-11
FABP3	113268386	63.58680377	5.990655	0	0
CMYA5	113255166	63.43677193	5.987247	1.16E-09	1.54E-08
TRIM54	113271121	63.15459045	5.980816	0	0
MYOT	113261774	60.99748054	5.930678	1.77E-10	2.72E-09
ASB2	113267322	60.75613084	5.924958	0	0
SMTNL2	113271048	60.54542751	5.919946	0	0
KLHL40	113253826	59.75882181	5.90108	0	0
CAP2	113264496	59.75726479	5.901042	0	0
TNNI1	113242556	59.13503214	5.885941	1.13E-10	1.8E-09
CAVIN4	113260389	58.833484	5.878566	0	0
MB	113255877	58.74290628	5.876343	1.08E-10	1.72E-09
MYOM3	113268506	58.4232845	5.868472	0	0
DUSP26	113262149	58.31882464	5.86589	0	0
CASQ1	113246682	57.67343745	5.849835	3.66E-10	5.37E-09
ASB11	113240709	56.74105562	5.826321	0	0
TNNC1	113256540	56.45655863	5.819069	5.5E-10	7.77E-09

ATP1B2	113271155	55.50312957	5.794497	0	0
PYGM	113244160	55.03702984	5.782331	2.62E-09	3.21E-08
MYPN	113259106	54.78304708	5.775658	2.22E-16	9.61E-15
MYBPC1	113255963	54.14704334	5.758811	3.52E-09	4.2E-08
FAM189A2	113270200	53.79951851	5.749521	0	0
TRIM72	113267722	53.28074572	5.735542	0	0
LOC113258282	113258282	52.62286608	5.717618	0	0
LOC113271214	113271214	52.5809361	5.716468	1.06E-08	1.16E-07
ASB16	113265364	52.54323311	5.715433	0	0
OBSCN	113247488	51.94451583	5.6989	2.41E-09	3E-08
HJV	113247768	51.94080053	5.698796	0	0
RPL3L	113245098	51.43538693	5.684689	0	0
DES	113250426	51.36269872	5.682649	5.09E-10	7.25E-09
CKM	113243434	50.95619072	5.671186	8.64E-09	9.62E-08
ADPRHL1	113247549	50.86521864	5.668608	0	0
SCN4A	113265278	50.25589606	5.651221	0	0
PKIA	113253025	49.8661755	5.63999	0	0
KLHL33	113248953	49.6344082	5.633269	0	0
MYL3	113256802	49.53380788	5.630342	2.38E-11	4.31E-10
TNNC2	113258216	49.24170577	5.621809	9.74E-10	1.31E-08
CLTCL1	113245966	48.62898475	5.603745	0	0
MYO18B	113244987	48.61395626	5.603299	2.39E-13	6.28E-12
S100A1	113245339	48.50207757	5.599975	0	0
NDRG2	113248486	47.30762008	5.564001	6E-15	2.04E-13
ACTN3	113243937	47.26954546	5.562839	9.04E-09	1E-07
ATP1B4	113270910	47.05383876	5.556241	0	0
MYLPF	113267993	47.02949354	5.555494	9.33E-09	1.03E-07
TXLNB	113259499	46.81437708	5.54888	1.6E-13	4.33E-12

PLN	113259393	46.71479723	5.545808	1.78E-15	6.58E-14
LOC113246737	113246737	46.54309964	5.540495	1.2E-12	2.79E-11
NEB	113250884	46.51166283	5.539521	8.94E-10	1.21E-08
ROS1	113259385	46.49458672	5.538991	0	0
CACNA1S	113242563	46.37871487	5.535391	1.81E-09	2.3E-08
TNNT3	113243956	46.08139089	5.526112	2.97E-09	3.6E-08
SRL	113270854	46.01555237	5.52405	1.01E-14	3.29E-13
TRIM63	113268472	45.79260906	5.517043	0	0
ANKRD39	113246637	45.7594282	5.515997	4.44E-16	1.8E-14
LOC113245168	113245168	45.70763307	5.514363	1.18E-11	2.29E-10
SMCO1	113242744	44.86658752	5.48757	0	0
ZNF750	113244462	44.46898638	5.474728	0	0
DDIT4L	113263108	44.4514301	5.474158	2.22E-16	9.61E-15
FZD9	113267800	44.41040735	5.472826	8.88E-16	3.42E-14
RYR1	113243259	44.37972252	5.471829	8.64E-12	1.71E-10
ASB14	113257003	44.25144376	5.467653	0	0
FAM131B	113265526	44.13575741	5.463876	0	0
JPH1	113253041	43.73011459	5.450555	4.44E-16	1.8E-14
TNNI2	113243913	43.65286884	5.448005	3.79E-09	4.49E-08
LOC113259203	113259203	42.90104953	5.422941	0	0
CLCN1	113265521	42.72472791	5.416999	0	0
ATP1A4	113246653	42.65090503	5.414504	0	0
SMPX	113242236	42.63935178	5.414114	9.33E-15	3.06E-13
KLHL38	113252751	42.49717921	5.409295	0	0
CELF3	113246099	42.43181449	5.407074	0	0
DOK7	113245759	42.28715644	5.402148	0	0
TNNT1	113247366	42.24125594	5.400581	5.14E-09	5.94E-08
TTN	113250712	41.64228408	5.379977	1.55E-08	1.64E-07

CACNB1	113265527	41.62293541	5.379307	0	0
WNK2	113270229	41.44719811	5.373203	0	0
NEURL2	113258666	41.24958361	5.366308	0	0
LOC113258625	113258625	41.21690834	5.365164	0	0
SYNM	113263644	41.05142686	5.35936	6.88E-15	2.3E-13
MYOM2	113262202	41.04918401	5.359282	3.34E-11	5.85E-10
SYPL2	113244926	40.9467055	5.355675	1.32E-14	4.24E-13
ALPK3	113263572	40.67954301	5.346232	0	0
CAMK2A	113263946	40.46513054	5.338607	0	0
COL2A1	113257484	40.31740983	5.333331	8.28E-11	1.36E-09
SPEG	113250425	40.26843062	5.331577	2.66E-15	9.48E-14
CKMT2	113255151	39.89026539	5.317965	7.05E-12	1.41E-10
LOC113246045	113246045	39.86346626	5.316995	4.81E-08	4.7E-07
RNF207	113270576	39.80858689	5.315008	0	0
CAND2	113257195	39.57646199	5.306571	0	0
RRAD	113252338	38.8853795	5.281156	0	0
LOC113249987	113249987	38.88048808	5.280974	1.79E-12	4.04E-11
LOC113245998	113245998	38.81880449	5.278684	8.2E-08	7.7E-07
SRPK3	113247839	38.80569974	5.278197	0	0
ART3	113263253	38.47364383	5.265799	0	0
TCAP	113265517	37.9636024	5.246545	9.42E-09	1.04E-07
HSPB8	113267026	37.6778652	5.235645	0	0
ADSSL1	113267196	37.35381896	5.223184	0	0
STAC3	113256388	37.20360801	5.217371	1.11E-16	4.96E-15
TMEM266	113248086	37.06915228	5.212147	0	0
TPD52L1	113259410	37.0230225	5.210351	0	0
HRC	113243313	36.89827164	5.205481	0	0
MYOZ1	113259184	36.821773	5.202487	3.03E-08	3.06E-07

BVES	113264788	36.3697476	5.184667	0	0
PGAM2	113257974	36.26968124	5.180692	1.64E-08	1.74E-07
AKAP6	113266112	36.06004005	5.172329	0	0
TACC2	113252016	36.03971259	5.171516	0	0
CDNF	113241393	36.00418426	5.170093	0	0
AMIGO1	113244923	35.64969463	5.155818	0	0
ACTN2	113258941	35.60800534	5.15413	9.5E-08	8.77E-07
MAPT	113265313	35.22268668	5.138433	0	0
ATP2A1	113267625	35.03077308	5.130551	2.38E-09	2.96E-08
USP2	113259958	33.7646639	5.077442	0	0
SCN1B	113244832	33.68380644	5.073983	0	0
LOC113244095	113244095	33.46561561	5.064608	0	0
MLIP	113261174	33.40419591	5.061957	0	0
FLNC	113263925	32.90037977	5.040032	4.25E-12	8.87E-11
DUSP27	113262889	32.57068491	5.025502	1.67E-15	6.22E-14
AQP4	113253308	32.5643528	5.025222	0	0
USP13	113254700	32.45219379	5.020244	0	0
NR4A1	113257593	32.40313141	5.018061	0	0
HOMER1	113255168	32.38386424	5.017203	0	0
MYH7B	113258523	32.35298451	5.015827	0	0
ZNF280B	113248736	32.17944428	5.008068	0	0
NT5DC3	113256072	32.12080879	5.005436	0	0
TMEM52	113270703	31.69317083	4.9861	1.11E-16	4.96E-15
NEXN	113254499	31.31236649	4.968661	0	0
ARPP21	113253764	31.29037174	4.967647	0	0
LDB3	113259245	31.27808857	4.96708	5.19E-12	1.07E-10
FAM184B	113266728	30.97639081	4.953097	0	0
PEBP4	113269994	30.85490605	4.947428	3.1E-11	5.48E-10

FITM1	113246014	30.65653324	4.938123	0	0
CA14	113246183	30.52356431	4.931852	0	0
PLCD4	113250452	30.32375002	4.922376	0	0
KBTBD12	113257266	29.96916664	4.905407	0	0
MACROD1	113244190	29.70707643	4.892735	0	0
ENO3	113271065	29.00465007	4.858212	1.2E-09	1.57E-08
PARM1	113263269	28.60536947	4.838214	0	0
HS3ST5	113264706	28.37382588	4.826489	7.88E-15	2.63E-13
LOC113250506	113250506	28.32298524	4.823901	0	0
FRMD1	113247120	28.22204102	4.81875	1.22E-12	2.82E-11
LOC113249648	113249648	28.05044398	4.809952	1.41E-14	4.49E-13
TPM1	113269709	27.90294004	4.802345	1.34E-09	1.76E-08
PLPP7	113266530	27.68561416	4.791065	0	0
LRRC14B	113260767	27.52108054	4.782465	0	0
NEURL1	113251895	27.49336005	4.781011	0	0
RAPSN	113264883	27.48452835	4.780548	0	0
SLC38A4	113257697	27.39447589	4.775813	0	0
SYNPO2	113255608	27.34687177	4.773304	2.22E-16	9.61E-15
CPT1B	113241597	27.23123715	4.767191	0	0
LMCD1	113257123	27.11784215	4.76117	0	0
MAPK12	113241615	26.77943207	4.743053	0	0
UBE2QL1	113260707	26.75625991	4.741805	0	0
ZNF385B	113250707	26.75452918	4.741711	1.17E-13	3.22E-12
FGFR4	113264137	26.74584798	4.741243	7.77E-16	3.01E-14
DNAJB5	113260494	26.73840321	4.740841	0	0
ADCY2	113260807	26.70479146	4.739027	4.15E-14	1.23E-12
LOC113256824	113256824	26.30841318	4.717452	0	0
FBXO32	113252752	26.24864986	4.714171	6.66E-16	2.62E-14

TMEM182	113247490	26.20828617	4.711951	0	0
SLC38A3	113256899	26.17913364	4.710345	2.55E-15	9.16E-14
LOC113249041	113249041	26.14846854	4.708655	0	0
CA3	113252987	26.11258226	4.706673	1.61E-07	1.43E-06
LOC113240972	113240972	25.95515931	4.697949	9.29E-11	1.51E-09
MUSTN1	113256971	25.65016785	4.680896	0	0
FRZB	113250693	25.60900751	4.678579	0	0
NT5C1A	113268245	25.57782522	4.676822	0	0
TMEM145	113243519	25.47471501	4.670994	1.44E-15	5.4E-14
LOC113271210	113271210	25.42769363	4.668329	7.66E-13	1.85E-11
LINGO4	113246192	25.38848755	4.666103	1.47E-14	4.65E-13
MAMSTR	113243335	25.33575536	4.663103	0	0
RTN2	113243425	25.31319365	4.661818	0	0
GAMT	113242729	25.11104276	4.65025	0	0
PLIN5	113242843	24.76669567	4.630329	0	0
MYLK4	113264229	24.33180296	4.604771	2.23E-11	4.09E-10
KLHL31	113261176	24.22878428	4.59865	0	0
SPATA4	113262018	24.22749865	4.598574	9.77E-15	3.19E-13
TBX1	113245945	23.90696508	4.579359	1.13E-12	2.63E-11
ANO2	113258097	23.8441421	4.575563	2.84E-14	8.7E-13
SEMA6C	113246217	23.74040629	4.569273	0	0
PPP1R3C	113243160	23.46197434	4.552253	0	0
RXRG	113268688	23.23034621	4.537939	0	0
CASZ1	113270517	23.1791052	4.534753	0	0
MYBPC2	113247311	23.1002527	4.529837	1.23E-06	9.17E-06
SLC25A4	113262270	23.0901461	4.529205	3.48E-10	5.12E-09
LSMEM1	113263014	22.99534925	4.52327	0	0
LOC113256403	113256403	22.96196201	4.521174	4.66E-15	1.61E-13

LOC113249473	113249473	22.65100354	4.501503	5.02E-12	1.03E-10
HOMER2	113263748	22.62184702	4.499645	0	0
SIX4	113242404	22.55328048	4.495265	0	0
RORC	113246193	22.39434287	4.485062	0	0
MN1	113244996	22.24194643	4.475211	0	0
BIN1	113263478	22.0956411	4.46569	6.11E-15	2.07E-13
DMPK	113243415	21.88865462	4.452111	0	0
MYOM1	113253248	21.6244106	4.434589	7.35E-10	1.01E-08
PITX1	113261483	21.61580757	4.434015	5.24E-14	1.53E-12
FAM160A1	113255755	21.60266063	4.433137	0	0
MSS51	113259180	21.44166763	4.422345	0	0
LRRN1	113257105	21.38919151	4.41881	0	0
ASB4	113260356	21.15941066	4.403228	2.81E-13	7.25E-12
FSD2	113263745	21.0919981	4.398624	0	0
FRMD3	113269816	21.09151013	4.39859	0	0
CNKSR1	113268466	21.08204796	4.397943	0	0
LOC113248033	113248033	21.04991201	4.395742	0	0
SCN3B	113259926	20.73488041	4.373988	0	0
RCAN2	113261236	20.72692022	4.373434	0	0
COQ8A	113262935	20.51400838	4.358538	1.97E-09	2.49E-08
YBX3	113257886	19.9617992	4.31917	9.26E-09	1.02E-07
FHL1	113241866	19.64215401	4.295881	3.61E-08	3.61E-07
PPP1R14C	113259556	19.58049165	4.291345	0	0
CASTOR2	113267765	19.54017616	4.288372	0	0
MTLN	113268093	19.52426758	4.287197	0	0
DPYSL5	113270914	19.16217998	4.26019	1.71E-11	3.2E-10
SLCO5A1	113253069	18.97780146	4.246241	3.48E-12	7.37E-11
GJA9	113268256	18.7374123	4.22785	0	0

COQ10A	113256427	18.7313686	4.227384	0	0
LOC113247381	113247381	18.7265868	4.227016	0	0
USP28	113260038	18.54570303	4.213013	0	0
LOC113249039	113249039	18.48781892	4.208503	1.04E-09	1.39E-08
DOK5	113258743	18.35010911	4.197717	0	0
FXYD1	113244833	18.33042969	4.196169	1.11E-16	4.96E-15
RASL10B	113268955	18.1987292	4.185766	5.69E-13	1.4E-11
EXPH5	113260094	18.05460408	4.174295	4.44E-16	1.8E-14
STRIP2	113264076	18.03375495	4.172628	0	0
ANKRD34A	113247786	18.02393441	4.171842	0	0
LOC113244847	113244847	18.01792832	4.171361	0	0
PPM1J	113244419	17.92361364	4.16379	0	0
GAPDH	113257977	17.87975067	4.160255	4.74E-08	4.64E-07
SPATA20	113265626	17.81435299	4.154968	0	0
RRAGD	113264828	17.75163319	4.14988	0	0
ALDOA	113267652	17.74300317	4.149178	9.13E-08	8.49E-07
KCNJ3	113250873	17.697903	4.145507	3.97E-10	5.76E-09
DDO	113264740	17.64393071	4.1411	7.18E-13	1.75E-11
PDZRN3	113257089	17.55555216	4.133855	0	0
TTC9	113270381	17.48289143	4.127872	0	0
C1QTNF4	113264951	17.4141836	4.122191	1.33E-15	5.03E-14
TECTB	113251934	17.36317437	4.117959	1.38E-07	1.24E-06
GPT2	113252205	17.24461981	4.108074	9.99E-16	3.81E-14
PROX1	113262688	17.11889221	4.097517	0	0
FHL3	113268262	17.11505218	4.097194	5.77E-15	1.97E-13
PEG3	113248914	16.9987044	4.087353	0	0
LOC113246316	113246316	16.94831965	4.08307	0	0
TEAD4	113258014	16.82766745	4.072763	0	0

ANXA9	113246188	16.6508333	4.057522	1.73E-11	3.23E-10
DLG2	113269385	16.56349119	4.049935	1.53E-10	2.38E-09
LDHD	113252492	16.52084869	4.046216	0	0
LOC113271213	113271213	16.46472008	4.041306	0.00011	0.000546
PACSIN3	113264972	16.45353871	4.040326	0	0
ESRRB	113270307	16.4362126	4.038806	8.61E-12	1.71E-10
PROB1	113261803	16.41631398	4.037058	0	0
LOC113249819	113249819	16.28588112	4.02555	2.22E-16	9.61E-15
CHCHD10	113247085	16.21519824	4.019275	1.4E-11	2.65E-10
EGLN3	113266114	16.16236981	4.014567	0	0
ABLIM2	113245677	16.13580029	4.012193	0	0
ADAMTSL5	113242739	16.10689681	4.009607	0	0
DTNA	113253331	16.01166107	4.001051	0	0
CRYAB	113260065	15.94211325	3.994771	3.41E-13	8.7E-12
KIAA1257	113257240	15.90669646	3.991562	2.25E-09	2.82E-08
VWA3B	113246750	15.84253162	3.985731	1.23E-12	2.85E-11
PFKM	113257678	15.7475364	3.977054	3.41E-07	2.83E-06
SYNC	113268345	15.6729558	3.970205	1.65E-14	5.22E-13
OSBPL6	113250717	15.42269183	3.946983	0	0
SLC25A12	113250777	15.3417034	3.939387	1.89E-15	6.93E-14
ME3	113269404	15.25142108	3.930872	1.22E-15	4.64E-14
CCDC7	113244234	15.24029551	3.929819	1.06E-10	1.7E-09
DKK2	113255536	15.22584179	3.92845	0	0
TMEM38A	113256659	15.20050996	3.926048	1.16E-09	1.54E-08
METTL24	113264743	15.17689988	3.923805	1.69E-13	4.54E-12
CDHR3	113258697	15.14998193	3.921244	8.89E-11	1.45E-09
SCHIP1	113254128	15.13691433	3.919999	0	0
PCBP4	113256933	15.03355272	3.910114	0	0

GPR157	113270672	14.90577329	3.897799	0	0
MIEF2	113271288	14.74841767	3.882488	0	0
MUM1L1	113241216	14.73324515	3.881003	0	0
LIPT2	113269312	14.70946205	3.878673	0	0
NR4A3	113260396	14.60403308	3.868295	6.48E-08	6.21E-07
UCHL1	113262438	14.55584358	3.863527	0	0
PRKCQ	113241147	14.5446472	3.862416	3.15E-12	6.74E-11
ZIC1	113254037	14.44842315	3.85284	4.83E-08	4.72E-07
COBL	113257504	14.39894333	3.847891	3.59E-13	9.08E-12
SMKR1	113264091	14.34683901	3.842661	1.53E-09	1.97E-08
TCF15	113258440	14.28958261	3.836892	3.96E-10	5.75E-09
BHLHE41	113257783	14.27131756	3.835047	0	0
LMOD1	113242545	14.25217	3.83311	0	0
UBE2D1	113259064	14.25080625	3.832972	0	0
REEP1	113268227	14.20607286	3.828436	0	0
FEM1A	113242841	14.18295391	3.826086	2.06E-11	3.8E-10
LOC113270558	113270558	14.03606665	3.811067	6.44E-10	8.96E-09
B3GALT1	113250816	13.88638738	3.795599	4.35E-11	7.45E-10
PPP1R3G	113264416	13.82141448	3.788833	3.33E-16	1.39E-14
LOC113261185	113261185	13.76270211	3.782692	8.39E-11	1.37E-09
ACACB	113267083	13.73411113	3.779692	0	0
DBNDD2	113258650	13.69173045	3.775233	0	0
GOT2	113252314	13.60095164	3.765636	4.19E-10	6.05E-09
RNF157	113244674	13.56588427	3.761911	0	0
MRLN	113259072	13.55538021	3.760794	3.06E-11	5.42E-10
CDIP1	113270847	13.27849385	3.73102	0	0
LIN7B	113243317	13.21609431	3.724224	8.66E-15	2.86E-13
MYO5C	113269636	13.07779045	3.709047	5.55E-16	2.21E-14

ITGB1BP2	113245616	12.98617834	3.698905	8.49E-14	2.4E-12
AHCYL2	113264044	12.96776796	3.696858	0	0
LOC113245320	113245320	12.95844522	3.695821	2.13E-09	2.68E-08
GTF2IRD1	113267774	12.56704739	3.651574	1.11E-16	4.96E-15
CNN1	113243031	12.5517857	3.649821	2.75E-10	4.12E-09
TRIM7	113261531	12.53947688	3.648405	1.11E-16	4.96E-15
DUSP3	113265375	12.50579162	3.644524	0	0
HSPB6	113244835	12.40419367	3.632756	2.56E-06	1.81E-05
FAM161B	113270340	12.31762679	3.622652	7.77E-16	3.01E-14
LRRC20	113259140	12.2563838	3.615461	0	0
ATP2A2	113266954	12.25072286	3.614795	7.62E-07	5.94E-06
ZDBF2	113250538	12.117838	3.59906	4.66E-15	1.61E-13
CUNH14orf28	113266169	12.10996332	3.598123	1.04E-12	2.45E-11
RNF152	113253477	12.05352391	3.591383	0	0
PPP2R3A	113253964	12.02826709	3.588357	0	0
PTGES3L	113265392	12.02415982	3.587864	6.08E-10	8.48E-09
SERINC2	113268384	12.0160875	3.586895	5.75E-14	1.67E-12
PDLIM5	113263133	11.72741423	3.551813	4.02E-10	5.83E-09
CUNH19orf47	113243571	11.69516451	3.54784	0	0
DCUN1D2	113247566	11.64045588	3.541076	0	0
SYNPO	113263952	11.59543298	3.535485	1.66E-12	3.79E-11
ZDHHC2	113262160	11.47459919	3.520372	0	0
GRIP2	113257219	11.41523172	3.512888	3.33E-16	1.39E-14
PLCL1	113248092	11.40242399	3.511269	2.14E-13	5.68E-12
CTNNA3	113259098	11.39731699	3.510622	6.96E-09	7.83E-08
DYRK1B	113243579	11.25412422	3.492382	1.88E-14	5.9E-13
TMEM117	113257713	11.23870842	3.490404	1.33E-15	5.03E-14
HACD1	113241365	11.22898628	3.489156	2.44E-15	8.78E-14

LOC113250050	113250050	11.22170777	3.48822	2.64E-10	3.96E-09
TBX15	113244365	11.18353041	3.483304	5.66E-12	1.15E-10
CMBL	113260816	11.17996016	3.482843	1.62E-09	2.08E-08
TCEA3	113268525	11.07809052	3.469637	6.64E-12	1.34E-10
MPZ	113246642	11.06930892	3.468493	6.56E-09	7.43E-08
GDF1	113256734	11.060868	3.467393	8.9E-10	1.2E-08
LOC113248248	113248248	11.04713409	3.4656	3.64E-08	3.63E-07
ZNF703	113262107	11.02916963	3.463252	7.77E-16	3.01E-14
NMB	113263730	11.01290961	3.461124	3.38E-09	4.06E-08
FILIP1	113261108	10.98522993	3.457493	3.33E-16	1.39E-14
KIAA0408	113259429	10.98067464	3.456895	1.69E-11	3.17E-10
ACVR2B	113253788	10.97933258	3.456718	9.53E-12	1.87E-10
PDE4C	113256711	10.96620906	3.454993	1.57E-11	2.95E-10
SH3YL1	113269154	10.9577478	3.453879	3.98E-08	3.95E-07
BMPR1B	113263130	10.95324581	3.453287	2.55E-07	2.17E-06
TUBA8	113258067	10.94874436	3.452694	8.47E-13	2.01E-11
ETV4	113265377	10.92904538	3.450095	1.51E-07	1.35E-06
SMARCD3	113241487	10.80432786	3.433537	5.55E-16	2.21E-14
XPO4	113251171	10.7750516	3.429623	9.87E-12	1.94E-10
BHLHE40	113257116	10.73478682	3.424222	3.57E-13	9.06E-12
PGM5	113270190	10.7216839	3.42246	3.44E-15	1.21E-13
ATP2B2	113257169	10.71931779	3.422141	6.33E-15	2.13E-13
GREB1L	113253269	10.62380034	3.409228	3.9E-14	1.16E-12
NOL3	113252357	10.59975256	3.405959	2.22E-11	4.06E-10
CACNA2D1	113259255	10.57947992	3.403197	0	0
ZNF106	113241988	10.55174875	3.39941	4.37E-07	3.56E-06
LCN2	113266453	10.54388889	3.398335	2.07E-08	2.15E-07
LOC113263090	113263090	10.51515862	3.394399	7.77E-16	3.01E-14

PTGR2	113270354	10.51162854	3.393914	0	0
SLC26A10	113256369	10.50929149	3.393594	3.36E-10	4.94E-09
SRCIN1	113265547	10.50517033	3.393028	5.19E-09	5.99E-08
HCN2	113242650	10.41753446	3.380942	2.66E-07	2.25E-06
SH3RF2	113255347	10.28894803	3.363024	5.8E-10	8.15E-09
CAMK2B	113257849	10.25905152	3.358825	1.99E-13	5.3E-12
ACAN	113263578	10.21243271	3.352255	0.003789	0.012239
MCRIP2	113245384	10.20159817	3.350723	1.21E-13	3.32E-12
FOXO6	113254226	10.15664289	3.344352	9.15E-08	8.5E-07
SPATA9	113255079	10.13032418	3.340608	6.76E-09	7.63E-08
WNT9A	113247507	10.12202143	3.339426	2.99E-10	4.43E-09
HSPB2	113260064	10.1149035	3.338411	3.33E-16	1.39E-14
PRADC1	113244005	10.07854257	3.333215	1.11E-16	4.96E-15
FZD7	113250581	9.994844568	3.321184	3.5E-14	1.05E-12
AMOT	113241247	9.966833011	3.317135	0	0
NR1D1	113265501	9.884964804	3.305236	1.21E-10	1.91E-09
DDR1	113264347	9.876540156	3.304006	0	0
CACNA2D3	113256988	9.871773778	3.303309	2.48E-07	2.11E-06
LOC113261724	113261724	9.831799007	3.297455	5.6E-10	7.88E-09
CCDC9B	113242035	9.765677398	3.28772	0	0
RASSF10	113246248	9.732046886	3.282743	1.32E-08	1.42E-07
SV2B	113263668	9.70500917	3.27873	5.38E-09	6.2E-08
MSANTD3	113260390	9.681862045	3.275285	1.07E-13	2.97E-12
RBM20	113251925	9.626785212	3.267054	1.18E-07	1.07E-06
RNF43	113268873	9.581781082	3.260294	3.26E-07	2.71E-06
TSPAN13	113261026	9.499136062	3.247796	1.17E-10	1.86E-09
RILP	113269121	9.448842459	3.240138	2.66E-15	9.48E-14
TPT1	113251351	9.444293601	3.239443	1.88E-05	0.000111

RASA4B	113267819	9.414094691	3.234822	2.22E-16	9.61E-15
PGM1	113254583	9.382326542	3.229946	2.21E-06	1.59E-05
SIX1	113242402	9.377190578	3.229156	2.64E-12	5.73E-11
PRKAA2	113254619	9.330776929	3.221997	3.17E-09	3.83E-08
TEAD1	113246254	9.289847632	3.215655	2.99E-10	4.43E-09
ACO2	113241726	9.283716664	3.214702	2.69E-06	1.9E-05
CSDC2	113241725	9.272291578	3.212926	8.08E-12	1.61E-10
ZNF672	113261519	9.267678723	3.212208	2.22E-15	8.07E-14
SLC25A3	113256113	9.266978104	3.212099	1.22E-05	7.56E-05
TTYH2	113265661	9.258460874	3.210772	2.69E-13	6.95E-12
IRS1	113250390	9.251030043	3.209614	2.66E-15	9.48E-14
CRHR2	113266912	9.238443667	3.20765	2.92E-07	2.45E-06
SGCD	113264007	9.204363624	3.202318	4.44E-16	1.8E-14
LGI1	113243181	9.18303199	3.198971	1.08E-10	1.73E-09
KCNQ4	113254824	9.171682716	3.197186	8.65E-10	1.17E-08
FSD1L	113260351	9.170551517	3.197009	3.06E-14	9.3E-13
FHL2	113267650	9.081480323	3.182927	9.28E-11	1.51E-09
CUNH1orf21	113262743	9.056619177	3.178973	4.44E-16	1.8E-14
PPEF1	113242253	9.053836945	3.178529	2.08E-09	2.63E-08
FHOD3	113253352	9.05237135	3.178296	4.05E-12	8.48E-11
ADRA2B	113241981	9.036803612	3.175813	1.53E-08	1.63E-07
PPP1R12B	113242537	9.020056729	3.173137	2.75E-11	4.91E-10
RETREG1	113260842	9.012519237	3.17193	7.74E-14	2.2E-12
FKBP3	113266176	8.992312508	3.168692	3.66E-12	7.71E-11
LOC113255514	113255514	8.967968476	3.164781	3.66E-15	1.28E-13
YIPF7	113262453	8.950401807	3.161952	1.86E-12	4.18E-11
LMO7	113251426	8.932152604	3.159008	6.76E-07	5.31E-06
CUNH15orf61	113240898	8.928985446	3.158496	1.28E-10	2E-09

HCFC1R1	113245424	8.914780165	3.156199	5.36E-13	1.33E-11
SOX15	113271164	8.90907757	3.155276	1.35E-07	1.22E-06
TMEM143	113243352	8.853385758	3.146229	1.13E-10	1.81E-09
TM6SF1	113263753	8.822274857	3.141151	6.42E-14	1.84E-12
KCNC4	113244908	8.795588913	3.13678	7.57E-11	1.25E-09
DYSF	113244079	8.783128816	3.134735	6.12E-10	8.53E-09
ANK2	113255588	8.780634313	3.134325	8.33E-08	7.81E-07
SLC7A2	113262158	8.763167166	3.131452	3.86E-12	8.13E-11
FXR2	113271143	8.741395999	3.127864	1.48E-12	3.39E-11
ANKRD37	113262279	8.682593823	3.118126	2.48E-13	6.51E-12
SHISA4	113242547	8.681483827	3.117942	4.8E-12	9.92E-11
LOC113258058	113258058	8.664585418	3.115131	2.35E-11	4.27E-10
GOT1	113251814	8.648162536	3.112394	1.74E-10	2.67E-09
EGFLAM	113260905	8.62837484	3.109089	2.58E-08	2.63E-07
LOC113266262	113266262	8.588886287	3.102471	6.99E-08	6.66E-07
MUSK	113260327	8.583255315	3.101525	2.23E-06	1.59E-05
LOC113244886	113244886	8.546060679	3.09526	8.44E-15	2.8E-13
RGMA	113263660	8.542123035	3.094595	2.94E-11	5.24E-10
FAM13C	113259068	8.525994528	3.091868	2.47E-12	5.41E-11
BTBD1	113263752	8.49994394	3.087453	3.68E-10	5.39E-09
EPM2A	113259523	8.422398817	3.074231	4.19E-11	7.21E-10
LOC113247480	113247480	8.397088752	3.069889	5.62E-08	5.43E-07
HOXC10	113257525	8.390395348	3.068739	4.73E-12	9.83E-11
SEC14L5	113270829	8.383437751	3.067542	9.27E-11	1.51E-09
CUNH6orf163	113264846	8.36536706	3.064429	9.18E-08	8.51E-07
SLC9A2	113247464	8.356457413	3.062891	1.9E-06	1.38E-05
ADAM22	113259540	8.341512078	3.060309	6.47E-10	8.98E-09
LOC113240981	113240981	8.339079681	3.059888	2.85E-07	2.4E-06

ZNF641	113257676	8.330182525	3.058348	1.04E-14	3.39E-13
DOCK3	113256616	8.325687895	3.057569	1.6E-06	1.17E-05
AK1	113266444	8.313787311	3.055506	1.77E-09	2.25E-08
LOC113250796	113250796	8.287832843	3.050995	1.09E-13	3.02E-12
EDN2	113254819	8.221373809	3.039379	1.03E-07	9.5E-07
GATM	113269569	8.175029405	3.031224	5.54E-06	3.7E-05
RAVER2	113254204	8.158785787	3.028354	1.1E-11	2.14E-10
PPARGC1A	113266747	8.153201459	3.027367	7.22E-08	6.87E-07
ABCC9	113257803	8.148223005	3.026485	1.96E-12	4.37E-11
TSPAN7	113265864	8.130175216	3.023286	1.34E-14	4.31E-13
HOXC11	113257526	8.087732969	3.015735	2.39E-06	1.7E-05
PRKAB2	113247754	8.085592179	3.015353	1.32E-11	2.54E-10
LSMEM2	113256903	8.056556532	3.010163	3.44E-12	7.31E-11
ATP5MC1	113265594	8.033416253	3.006014	4.95E-09	5.77E-08
TLE1	113269821	8.007757073	3.001398	2.25E-14	7.04E-13
OBSL1	113250419	7.990962875	2.998369	1.03E-12	2.43E-11
LOC113250051	113250051	7.990444159	2.998276	2.18E-08	2.25E-07
DNAJC27	113270614	7.975546029	2.995583	1.5E-06	1.1E-05
ADAMTS8	113260177	7.965984675	2.993853	0.000184	0.000866
RDH13	113247404	7.960249498	2.992814	8.23E-11	1.35E-09
AFAP1L1	113263930	7.956171556	2.992074	2.45E-12	5.39E-11
KIF1C	113271057	7.947784003	2.990553	4.19E-07	3.42E-06
NRXN1	113245852	7.935712992	2.98836	1.09E-06	8.24E-06
IDH3A	113263797	7.930810514	2.987468	3.93E-10	5.72E-09
SSPN	113257784	7.868812915	2.976146	3.12E-12	6.69E-11
FBXO27	113243597	7.829969225	2.969007	1.64E-05	9.8E-05
PRUNE2	113269835	7.828159551	2.968673	1.85E-05	0.000109
PINK1	113268560	7.797582478	2.963027	4.68E-08	4.59E-07

SCNM1	113246162	7.752045284	2.954577	6.53E-14	1.87E-12
COQ9	113252286	7.745785269	2.953412	1.16E-08	1.27E-07
CFL2	113266119	7.72870623	2.950227	6.19E-08	5.95E-07
PPFIA4	113242527	7.712127244	2.947129	4.69E-07	3.79E-06
ACTG2	113243866	7.698187319	2.944519	2.41E-08	2.47E-07
LOC113243744	113243744	7.682995437	2.941669	4.68E-07	3.79E-06
LOC113261698	113261698	7.669830075	2.939195	2.7E-09	3.31E-08
CNKSR2	113242237	7.65211568	2.935859	4.14E-07	3.4E-06
CCNG1	113264054	7.640692952	2.933703	0.0001	0.000503
NDUFS3	113264954	7.624990397	2.930736	2.51E-09	3.1E-08
HDAC11	113257231	7.618428007	2.929493	1.53E-12	3.48E-11
DUSP8	113243950	7.588406797	2.923797	9.21E-08	8.54E-07
SAMD4A	113242354	7.581715834	2.922524	6.22E-15	2.1E-13
MME	113254091	7.554872214	2.917407	5.46E-08	5.3E-07
FAM174A	113255038	7.528820874	2.912424	8.77E-15	2.89E-13
LOC113246689	113246689	7.525646323	2.911815	3.4E-07	2.82E-06
RORA	113269696	7.517163641	2.910188	5.29E-09	6.1E-08
PDE4D	113254908	7.472489246	2.901589	2.29E-09	2.86E-08
LOC113245020	113245020	7.418497528	2.891127	1.39E-08	1.49E-07
BAG3	113251998	7.409756985	2.889426	7.71E-13	1.85E-11
IPO13	113254778	7.400090571	2.887543	1.21E-10	1.92E-09
LOC113252198	113252198	7.397482012	2.887034	5.91E-07	4.69E-06
LOC113247479	113247479	7.36636027	2.880952	5.13E-07	4.11E-06
LOC113261335	113261335	7.348616909	2.877473	5.48E-08	5.31E-07
LOC113253852	113253852	7.343596086	2.876487	2.23E-07	1.92E-06
LOC113270677	113270677	7.327563657	2.873334	2.64E-06	1.87E-05
SAMD11	113270658	7.316012421	2.871058	3.15E-12	6.74E-11
ALDH1A2	113269676	7.314981049	2.870854	1.25E-06	9.32E-06

ZNF853	113267949	7.310575234	2.869985	1.16E-10	1.84E-09
LOC113268666	113268666	7.252201343	2.858419	2.13E-11	3.9E-10
PTPN3	113260335	7.22532788	2.853063	6.14E-12	1.24E-10
PPP3CC	113270001	7.202154229	2.848428	2.3E-12	5.07E-11
MLYCD	113252546	7.172552624	2.842487	4.72E-13	1.17E-11
CARNS1	113244030	7.171515364	2.842278	1.8E-08	1.88E-07
STC2	113264106	7.170918189	2.842158	2.25E-07	1.93E-06
SOX17	113268095	7.169734879	2.84192	2.47E-10	3.71E-09
EXTL1	113268473	7.15619801	2.839193	2.74E-06	1.93E-05
PLAC8L1	113255346	7.147298766	2.837398	1.67E-06	1.22E-05
LTBP4	113243560	7.143776596	2.836687	9.1E-08	8.47E-07
SMPDL3B	113268215	7.104632599	2.82876	2.9E-06	2.04E-05
LOC113241646	113241646	7.065759189	2.820845	1.15E-06	8.65E-06
ZBED9	113248149	7.059299712	2.819525	3.06E-07	2.56E-06
TMEM178B	113265194	7.0542612	2.818495	4.12E-06	2.82E-05
FBN3	113242673	7.04808923	2.817232	4.9E-06	3.31E-05
PDP1	113252931	7.04434974	2.816467	5.66E-12	1.15E-10
ARMC2	113264761	7.043148465	2.81622	1.17E-07	1.06E-06
LOC113266367	113266367	7.017695517	2.810997	1.69E-13	4.54E-12
MYH11	113270760	7.003232255	2.808021	5.67E-10	7.96E-09
CHCHD3	113264293	6.98859925	2.805003	1.09E-07	9.95E-07
LOC113240812	113240812	6.982937789	2.803834	2.79E-10	4.17E-09
SYT9	113245781	6.953225525	2.797682	5.26E-06	3.53E-05
LRCH2	113241252	6.948900609	2.796785	7.51E-07	5.86E-06
RAB3A	113256710	6.921868242	2.791161	1.9E-12	4.27E-11
HDHD5	113258055	6.918837004	2.79053	1.97E-08	2.05E-07
ETFDH	113255802	6.898279169	2.786237	2.74E-07	2.31E-06
ATP1B1	113262865	6.881703517	2.782766	1.12E-10	1.79E-09

ACADVL	113271108	6.88066141	2.782547	1.16E-05	7.24E-05
SMYD1	113242840	6.880418017	2.782496	2.53E-13	6.61E-12
ADCY9	113270857	6.872362413	2.780806	2.12E-11	3.9E-10
FXR1	113254657	6.871710133	2.780669	2.35E-07	2.01E-06
EDA2R	113246887	6.870028991	2.780316	2.49E-06	1.77E-05
LOC113248397	113248397	6.866759396	2.779629	9.49E-08	8.77E-07
POPDC2	113251933	6.861604437	2.778546	2.28E-09	2.85E-08
CNTN3	113257091	6.85204373	2.776534	4.31E-06	2.95E-05
CDKL2	113263265	6.850639221	2.776239	6.22E-06	4.11E-05
RPL10	113247845	6.844918988	2.775033	0.000181	0.000857
CAMK2D	113255589	6.834240084	2.772781	4.36E-13	1.1E-11
NFIX	113247201	6.818836199	2.769526	2.1E-08	2.17E-07
TMEM65	113252747	6.805687333	2.766741	2.29E-12	5.06E-11
LOC113252883	113252883	6.804959637	2.766587	2.87E-07	2.41E-06
USF3	113251469	6.795833824	2.764651	1.35E-09	1.76E-08
LOC113267511	113267511	6.790577229	2.763534	1.53E-06	1.12E-05
MAP3K20	113250769	6.775245011	2.760273	3.59E-08	3.59E-07
CHRNB1	113271122	6.774343198	2.760081	3.9E-12	8.19E-11
AXIN2	113265246	6.768891798	2.75892	1.35E-09	1.76E-08
RNF122	113262147	6.757554864	2.756501	2.59E-08	2.64E-07
HBEGF	113261821	6.745916266	2.754014	7.12E-11	1.18E-09
PRKRA	113250716	6.741626358	2.753097	2.48E-12	5.42E-11
LOC113242772	113242772	6.741397747	2.753048	3.22E-11	5.67E-10
RAMP1	113250175	6.737072848	2.752122	1.39E-08	1.49E-07
DUSP28	113249733	6.715583732	2.747513	9.28E-12	1.83E-10
MRPS15	113268283	6.700553102	2.74428	3.2E-08	3.22E-07
ANK3	113259074	6.69115598	2.742255	1.41E-09	1.83E-08
ESRRA	113244171	6.679886455	2.739824	1.26E-10	1.98E-09

NDUFS8	113244009	6.654577016	2.734347	5.93E-09	6.77E-08
PRRG3	113246828	6.653092098	2.734025	7.97E-06	5.15E-05
PKNOX2	113259907	6.651612991	2.733704	1.49E-09	1.92E-08
LOC113242529	113242529	6.642758904	2.731783	2.44E-11	4.4E-10
GOLGA7B	113251802	6.640961131	2.731392	4.94E-07	3.98E-06
GRM2	113256929	6.637895442	2.730726	4.24E-06	2.9E-05
PI16	113243871	6.635627601	2.730233	2.46E-07	2.1E-06
MDH2	113267810	6.588914917	2.720041	1.06E-05	6.67E-05
HES1	113253430	6.568918095	2.715656	6.71E-11	1.12E-09
PPARGC1B	113263936	6.552232491	2.711987	5.53E-10	7.8E-09
SLC27A1	113256682	6.524759835	2.705925	5.7E-09	6.53E-08
PPP1R13L	113243431	6.518721048	2.704589	2.12E-09	2.66E-08
PDHA1	113242248	6.477209626	2.695372	4.12E-06	2.82E-05
LOC113242144	113242144	6.474494304	2.694768	8.91E-07	6.86E-06
TPMT	113264505	6.464765776	2.692598	4.62E-11	7.87E-10
P2RY1	113254080	6.460928721	2.691742	1.84E-07	1.61E-06
LOC113243236	113243236	6.454432555	2.69029	1.26E-11	2.42E-10
FYCO1	113256782	6.445972713	2.688398	5E-09	5.79E-08
LOC113271320	113271320	6.442484377	2.687617	4.77E-08	4.67E-07
NDUFAF6	113252915	6.423890089	2.683447	7.33E-09	8.22E-08
NDUFA12	113256147	6.421132556	2.682828	3.93E-09	4.64E-08
ETFRF1	113257793	6.409534792	2.68022	2.18E-07	1.87E-06
SHISA2	113251193	6.401701373	2.678455	7.36E-10	1.01E-08
SUCLA2	113251333	6.381590783	2.673916	4.66E-07	3.77E-06
RILPL1	113266895	6.364944067	2.670148	8.13E-11	1.33E-09
LOC113248617	113248617	6.354983589	2.667888	9.13E-06	5.82E-05
PPTC7	113266959	6.351822306	2.667171	8.43E-10	1.15E-08
MYH14	113247345	6.347551514	2.6662	5.19E-09	5.99E-08

SETD9	113255320	6.335526336	2.663464	2.1E-07	1.81E-06
TLR9	113256623	6.321367372	2.660237	4.07E-10	5.9E-09
ATP5MG	113260000	6.316439889	2.659112	2.65E-06	1.87E-05
ADAMTS20	113257720	6.311410602	2.657962	7.52E-06	4.89E-05
LGI2	113266752	6.309054272	2.657424	1.17E-05	7.28E-05
LOC113245770	113245770	6.30589226	2.656701	1.52E-05	9.16E-05
WISP3	113264685	6.298303873	2.654963	9.25E-07	7.1E-06
SERINC4	113241957	6.291165533	2.653327	2.39E-07	2.04E-06
SCX	113247694	6.290541149	2.653184	4.95E-05	0.000266
NDUFA4	113260639	6.283870149	2.651653	5.62E-06	3.75E-05
SLC35F2	113260101	6.282765938	2.6514	2.61E-09	3.2E-08
PPARA	113241644	6.273731879	2.649324	3.64E-11	6.34E-10
CAPN3	113241990	6.261786154	2.646574	0.00107	0.004083
CENPV	113271255	6.244828243	2.642662	1.73E-08	1.82E-07
NIPSNAP2	113267734	6.226946484	2.638525	6.12E-07	4.86E-06
TRH	113257205	6.205034911	2.633439	9.08E-07	6.98E-06
NDUFB9	113252742	6.178713451	2.627306	7.51E-07	5.86E-06
GPRC5C	113265205	6.171373065	2.625592	1.16E-07	1.05E-06
ZBTB18	113262963	6.13977987	2.618187	2.58E-11	4.64E-10
STBD1	113263249	6.119940837	2.613518	1.09E-06	8.25E-06
PHTF2	113259141	6.109753095	2.611114	2.13E-10	3.24E-09
DVL1	113270636	6.083712593	2.604952	3.15E-09	3.81E-08
KIAA1671	113244966	6.083302833	2.604855	4.95E-11	8.41E-10
LOC113248438	113248438	6.056798411	2.598555	1.54E-08	1.64E-07
NDUFB10	113245135	6.055005743	2.598128	1.87E-07	1.63E-06
BAG2	113261157	6.05338804	2.597743	3.42E-11	5.98E-10
MFAP4	113271275	6.048171175	2.596499	3.39E-05	0.000188
VDAC2	113259206	6.039324003	2.594387	0.000113	0.000562

ECI1	113245118	6.012121235	2.587874	7.46E-07	5.83E-06
HLF	113265652	6.003264619	2.585747	1.6E-09	2.06E-08
IDH2	113263704	5.99452789	2.583646	2.34E-05	0.000135
CLIP4	113240806	5.980095059	2.580168	1.82E-07	1.6E-06
CASQ2	113244385	5.97542833	2.579042	8.05E-07	6.25E-06
ZFP3	113271064	5.97326311	2.578519	1.63E-11	3.07E-10
SLC2A4	113271115	5.962536525	2.575926	1.09E-06	8.24E-06
ZNF784	113247372	5.955427153	2.574205	3.97E-11	6.86E-10
FAHD1	113245129	5.954289406	2.573929	3.88E-11	6.72E-10
PDK4	113260367	5.952428137	2.573478	0.000245	0.001117
LOC113255131	113255131	5.95038319	2.572983	6.54E-06	4.31E-05
TMC7	113270737	5.949157893	2.572685	2.27E-05	0.000131
KIZ	113246779	5.944854062	2.571641	2.34E-09	2.92E-08
TPH2	113256261	5.935556209	2.569383	1.1E-05	6.91E-05
MYRIP	113253804	5.932124679	2.568549	2.73E-06	1.92E-05
ANAPC13	113253961	5.928021719	2.567551	9.18E-08	8.51E-07
PTGES2	113266452	5.923355394	2.566415	7.32E-09	8.22E-08
SEL1L3	113266760	5.923243274	2.566387	2.24E-11	4.09E-10
KIF1B	113270521	5.904179759	2.561737	1.42E-07	1.27E-06
LOC113243583	113243583	5.897930184	2.560209	1.01E-06	7.68E-06
WNT11	113269338	5.886753609	2.557472	1.07E-05	6.7E-05
FAM210A	113245962	5.882731563	2.556486	5.1E-11	8.63E-10
MORN4	113251794	5.881932828	2.55629	1.49E-06	1.1E-05
PYGO1	113269652	5.876674515	2.555	3.73E-10	5.45E-09
CRISPLD1	113253036	5.874948368	2.554576	0.000327	0.001445
KLF11	113269781	5.87326924	2.554164	1.68E-08	1.78E-07
LOC113267549	113267549	5.85842565	2.550513	8.48E-06	5.45E-05
LOC113245579	113245579	5.851463409	2.548797	0.000598	0.002459

LOC113245723	113245723	5.84792332	2.547924	3.22E-05	0.00018
FREM2	113251271	5.842298963	2.546536	0.000314	0.00139
KCNA5	113257992	5.839539131	2.545855	0.000124	0.000604
PLPPR1	113260387	5.836672364	2.545146	0.000237	0.001083
NDUFA11	113242864	5.834026137	2.544492	5.9E-10	8.26E-09
NQO1	113252434	5.830650385	2.543657	5.19E-11	8.77E-10
TEAD3	113243691	5.82895446	2.543237	2.5E-07	2.13E-06
LOC113253727	113253727	5.825279948	2.542327	3.94E-05	0.000216
LOC113257480	113257480	5.820090642	2.541042	3.73E-06	2.57E-05
LOC113252181	113252181	5.797127588	2.535338	0.000166	0.000791
ZNF462	113260346	5.790765083	2.533754	2.38E-11	4.31E-10
CEACAM20	113243463	5.790555399	2.533702	3.83E-05	0.00021
LOC113254331	113254331	5.785271272	2.532385	9.79E-10	1.31E-08
TEX9	113269661	5.778979541	2.530815	2E-07	1.74E-06
ATP5MD	113251890	5.773479692	2.529441	3.46E-09	4.14E-08
LOC113257418	113257418	5.767537519	2.527955	3.52E-08	3.52E-07
DENND2D	113244894	5.767147856	2.527858	3.08E-09	3.73E-08
MRPL51	113257979	5.759873345	2.526037	1.43E-09	1.85E-08
UCKL1	113258827	5.757244893	2.525379	3.02E-11	5.35E-10
PHKA1	113245604	5.748705442	2.523237	4.42E-07	3.6E-06
LOC113271230	113271230	5.745748124	2.522495	1.39E-09	1.81E-08
BCL6B	113271094	5.743637129	2.521965	6.58E-09	7.44E-08
PRKN	113259330	5.734965474	2.519785	2.43E-06	1.73E-05
MLPH	113250295	5.680377425	2.505987	3.49E-05	0.000194
RNF150	113255701	5.661863557	2.501277	1.22E-08	1.32E-07
MLF1	113254118	5.659582318	2.500696	7.81E-08	7.38E-07
NDUFA3	113247437	5.644510808	2.496849	2.94E-09	3.58E-08
HMCN2	113266283	5.643602501	2.496616	3.77E-05	0.000207

SBK1	113267619	5.62824898	2.492686	6.77E-10	9.36E-09
FDFT1	113269919	5.616824613	2.489755	4.28E-08	4.23E-07
ERBB2	113265512	5.611378758	2.488355	1.84E-07	1.62E-06
LGALSL	113244940	5.608598646	2.48764	5.8E-08	5.59E-07
CUNH12orf73	113256066	5.593705378	2.483804	7.93E-10	1.08E-08
WDR17	113262226	5.591119622	2.483137	3.33E-05	0.000185
COPS9	113249750	5.589966007	2.48284	7.24E-09	8.14E-08
LIX1	113255058	5.585838905	2.481774	4.9E-07	3.95E-06
ANOS1	113240755	5.580970583	2.480516	1.81E-05	0.000107
YJEFN3	113256756	5.580872996	2.480491	3.31E-05	0.000184
FLT1	113251212	5.575517146	2.479106	4.86E-10	6.93E-09
NDUFA5	113263568	5.574741212	2.478905	3.45E-09	4.14E-08
UBE2B	113261739	5.566987162	2.476897	2.3E-07	1.97E-06
LOC113260502	113260502	5.564629564	2.476286	5.42E-06	3.62E-05
AHDC1	113268435	5.558151421	2.474605	1.94E-11	3.6E-10
CA11	113243341	5.548128136	2.472001	0.000118	0.00058
PPP1R1A	113257512	5.547066035	2.471725	0.000894	0.003492
PANK1	113243152	5.536769316	2.469044	2.44E-09	3.03E-08
BOLA3	113243813	5.5364189	2.468953	1.93E-08	2.02E-07
LRRC47	113270589	5.527979114	2.466752	1.88E-09	2.38E-08
NDUFS7	113242730	5.520934937	2.464913	4.39E-07	3.57E-06
KPNA3	113251312	5.517957346	2.464134	1.29E-06	9.62E-06
OTUD1	113241334	5.515815148	2.463574	2.08E-07	1.8E-06
CUNH6orf136	113264361	5.51266198	2.462749	1.7E-08	1.8E-07
OAZ2	113240855	5.510504	2.462184	1.54E-08	1.64E-07
MTTP	113263115	5.504583834	2.460633	1.32E-05	8.09E-05
SPAG7	113271070	5.499083617	2.459191	7.93E-10	1.08E-08
GRAMD2B	113261664	5.490260316	2.456875	6.83E-09	7.69E-08

LOC113248560	113248560	5.486721685	2.455944	5.1E-05	0.000273
FGF13	113241875	5.476154067	2.453163	1.48E-05	8.94E-05
SCN8A	113257599	5.475237559	2.452922	1.2E-09	1.57E-08
GPD1L	113253839	5.471042174	2.451816	7.46E-10	1.03E-08
CCDC155	113243304	5.455632571	2.447746	5.79E-05	0.000306
DYRK2	113256306	5.455516169	2.447716	4.96E-10	7.06E-09
MEF2C	113255121	5.444189822	2.444717	1.42E-06	1.05E-05
DOC2B	113268834	5.444159978	2.444709	1.57E-05	9.42E-05
LOC113247645	113247645	5.436475106	2.442672	0.000331	0.001456
ZNRF1	113252493	5.418873766	2.437993	2.96E-09	3.6E-08
TMEM38B	113260348	5.409990057	2.435626	4.27E-09	5E-08
HOXA10	113262042	5.397442729	2.432276	2.27E-09	2.83E-08
TMEM246	113260380	5.392308903	2.430903	7.85E-06	5.09E-05
ACADM	113254520	5.388383606	2.429853	1.45E-05	8.81E-05
ACOX3	113245696	5.386825448	2.429435	1.15E-08	1.26E-07
AAMDC	113269361	5.383916659	2.428656	3.98E-09	4.69E-08
NNT	113260959	5.371203587	2.425245	1.75E-06	1.28E-05
CUNH10orf62	113251793	5.354518136	2.420757	2.78E-05	0.000158
STOX2	113262252	5.344561294	2.418072	1.74E-07	1.53E-06
MRPS36	113255258	5.33261457	2.414843	1.08E-08	1.18E-07
LOC113250450	113250450	5.31103322	2.408993	2.98E-09	3.63E-08
NDUFA1	113241285	5.303990583	2.407078	1.5E-08	1.6E-07
SLITRK2	113241887	5.298592164	2.405609	0.000401	0.001727
PGAP3	113265515	5.294264167	2.40443	1.17E-11	2.28E-10
LOC113250233	113250233	5.285752321	2.402109	1.62E-10	2.5E-09
CTNNAL1	113260338	5.283956001	2.401618	1.88E-08	1.96E-07
PHF24	113260496	5.281801576	2.40103	1.34E-05	8.17E-05
CACNA1H	113245079	5.27816491	2.400036	1.2E-05	7.43E-05

NXN	113269097	5.276538079	2.399592	8.67E-09	9.65E-08
ZFAND5	113270228	5.276342198	2.399538	1.33E-06	9.89E-06
LOC113248562	113248562	5.270195848	2.397857	1.02E-08	1.12E-07
ITIH4	113256969	5.269590197	2.397691	0.000194	0.000906
CAGE1	113264234	5.257284543	2.394318	8.18E-05	0.000419
SPHKAP	113250375	5.254945421	2.393676	0.001112	0.004223
TCIM	113245539	5.254461323	2.393543	5.56E-08	5.38E-07
METTL22	113270820	5.251648646	2.39277	3.38E-09	4.05E-08
LOC113247989	113247989	5.245952889	2.391205	3.53E-06	2.44E-05
IDH3G	113247853	5.245782147	2.391158	6.66E-06	4.37E-05
TPI1	113257956	5.240625064	2.389739	0.000879	0.003438
WDR88	113244752	5.229630098	2.386709	2.98E-05	0.000168
NDUFAB1	113267578	5.215475644	2.382799	8.43E-08	7.89E-07
SIRT5	113264471	5.210388645	2.381391	1.21E-07	1.09E-06
CHRNE	113271084	5.204140984	2.37966	6.24E-05	0.000328
LOC113250232	113250232	5.191194441	2.376067	5.58E-10	7.87E-09
LOC113249271	113249271	5.188905333	2.37543	2.11E-09	2.65E-08
P2RX5	113269156	5.18605883	2.374639	2.21E-09	2.77E-08
SNURF	113263596	5.184131161	2.374102	6.04E-09	6.88E-08
PELI3	113244063	5.182695035	2.373703	5.64E-06	3.75E-05
NDUFB5	113254753	5.182254423	2.37358	6.48E-09	7.35E-08
NDUFS4	113263912	5.180952033	2.373217	6.79E-08	6.49E-07
PDE4B	113254561	5.176978039	2.37211	3.17E-07	2.64E-06
ACYP2	113245706	5.16460707	2.368659	1.02E-05	6.45E-05
TAGLN	113260017	5.162970495	2.368201	1.82E-07	1.6E-06
JAG2	113267148	5.154148089	2.365734	4.1E-09	4.82E-08
MRPL33	113240736	5.14920264	2.364349	5.08E-08	4.95E-07
KIAA1217	113241331	5.142682744	2.362521	9.76E-10	1.31E-08

BZW2	113261015	5.142298357	2.362413	2.76E-07	2.33E-06
MAP3K7CL	113267437	5.125506677	2.357695	2.11E-06	1.52E-05
FAM83C	113258536	5.124313321	2.357359	1.91E-05	0.000112
ACTA2	113258969	5.116930042	2.355279	1.19E-09	1.57E-08
RNF225	113248032	5.116265534	2.355091	7.65E-05	0.000394
LOC113244568	113244568	5.109196029	2.353096	8.37E-05	0.000427
LOC113266806	113266806	5.09912202	2.350249	0.002569	0.008747
VDAC3	113245581	5.097905706	2.349905	4.07E-05	0.000221
SULF2	113258690	5.091898189	2.348204	1.15E-06	8.66E-06
LOC113254515	113254515	5.08986265	2.347627	0.000189	0.000887
MAFF	113241783	5.088669802	2.347289	3.79E-08	3.77E-07
NDUFA8	113266369	5.088070083	2.347119	1.56E-06	1.15E-05
DNASE1L1	113247843	5.041284203	2.333791	7.82E-07	6.08E-06
NHLRC1	113264504	5.032565712	2.331294	1.38E-05	8.46E-05
DKK3	113246270	5.001037803	2.322228	0.00013	0.000631
IRAK1BP1	113261094	4.993969694	2.320187	2.14E-06	1.54E-05
HOXA7	113262001	4.990864408	2.31929	5.66E-07	4.51E-06
ARPP19	113269638	4.98805239	2.318477	2.94E-06	2.06E-05
LOC113248616	113248616	4.982546716	2.316883	2.11E-08	2.18E-07
LOC113252908	113252908	4.973140037	2.314157	1.29E-05	7.93E-05
IMMP2L	113262909	4.971730147	2.313748	3.44E-07	2.85E-06
SUSD4	113262898	4.968029602	2.312674	0.000622	0.002543
PDK2	113265612	4.967713081	2.312582	3.65E-06	2.52E-05
ATP5MPL	113267204	4.958596821	2.309932	2.1E-06	1.51E-05
ACADS	113267053	4.957977519	2.309752	2.71E-06	1.91E-05
LYSMD1	113246163	4.952779437	2.308238	6.55E-08	6.27E-07
SDHB	113270447	4.950619086	2.307609	1.91E-06	1.38E-05
HIF3A	113243399	4.946625659	2.306445	0.00019	0.000888

VDAC1	113261734	4.94236777	2.305202	0.000139	0.000671
HOMER3	113256738	4.941869015	2.305057	3.26E-09	3.93E-08
IGSF23	113243462	4.918616999	2.298253	4.02E-05	0.000219
MFN2	113270495	4.918232638	2.29814	6.26E-06	4.14E-05
CUEDC1	113268859	4.908684331	2.295336	7.67E-08	7.26E-07
CYR61	113254464	4.908344555	2.295237	1.77E-08	1.86E-07
TEX29	113248531	4.904636985	2.294146	8.51E-06	5.46E-05
LOC113257882	113257882	4.90434679	2.294061	0.000108	0.000539
NDUFA9	113257995	4.898221765	2.292258	1.11E-06	8.39E-06
ATP5MC2	113257544	4.896569563	2.291771	2.28E-05	0.000132
SLC25A11	113271067	4.883670965	2.287966	1.78E-06	1.3E-05
PPP1R3F	113265953	4.880940524	2.287159	1.24E-06	9.29E-06
MAGIX	113265825	4.876353542	2.285803	2.25E-07	1.93E-06
KHDRBS3	113252694	4.873685864	2.285013	0.000129	0.000629
SYT7	113246992	4.870581233	2.284094	8.28E-07	6.41E-06
SLC25A30	113251350	4.87050487	2.284071	1.72E-08	1.81E-07
SUCLG1	113243306	4.869676356	2.283826	8.45E-05	0.000431
NEU3	113269322	4.864477853	2.282285	8.99E-09	9.98E-08
LOC113261568	113261568	4.863444805	2.281979	1.16E-08	1.26E-07
TOB1	113265636	4.861364096	2.281361	6.93E-08	6.61E-07
SVIL	113244243	4.860904544	2.281225	3.36E-05	0.000187
CIPC	113270299	4.860780052	2.281188	3.53E-09	4.21E-08
CFAP54	113256123	4.850251988	2.27806	0.000331	0.001457
TNFRSF12A	113245425	4.846130893	2.276833	0.000362	0.001577
KIAA1755	113258589	4.842617008	2.275787	8.77E-05	0.000446
ATP5F1A	113253374	4.840685563	2.275211	0.001838	0.006532
TRIM45	113244373	4.840650775	2.275201	2.45E-08	2.5E-07
LOC113257633	113257633	4.83940976	2.274831	3.44E-09	4.12E-08

TENM1	113270934	4.835496757	2.273664	0.000142	0.000685
ZSCAN12	113248152	4.815036701	2.267547	1.75E-07	1.54E-06
LOC113244843	113244843	4.813134009	2.266977	6.72E-06	4.41E-05
LOC113258460	113258460	4.808523112	2.265594	9.73E-05	0.000489
HOXD9	113250743	4.807298689	2.265226	0.0002	0.000929
NDUFB11	113265993	4.801283092	2.26342	8.75E-07	6.74E-06
LOC113249623	113249623	4.791451023	2.260463	0.012365	0.03346
ITIH3	113256968	4.786123389	2.258858	0.000112	0.000555
PPP3CB	113259177	4.78374038	2.258139	4.94E-07	3.98E-06
INHBB	113263463	4.775124396	2.255538	6.33E-05	0.000332
LOC113251521	113251521	4.766539513	2.252942	0.002711	0.009173
FMC1	113264958	4.754493474	2.249292	2.01E-09	2.54E-08
CISD1	113259063	4.752934296	2.248818	1.33E-06	9.91E-06
COMP	113256730	4.744476845	2.246249	0.009866	0.027616
CLCN4	113240744	4.73774328	2.2442	3E-07	2.51E-06
CHST10	113247050	4.734594766	2.243241	5.93E-09	6.77E-08
CCDC28B	113268366	4.726893461	2.240892	7.64E-06	4.96E-05
EFCC1	113257241	4.726197745	2.24068	3.25E-05	0.000181
TGFB2	113268733	4.711644914	2.236231	1.79E-08	1.88E-07
LOC113246281	113246281	4.706216007	2.234568	0.000409	0.001754
SLC14A2	113253366	4.698756455	2.232279	0.000488	0.002054
ABLIM1	113251961	4.697014479	2.231744	2.1E-05	0.000122
NFIC	113242797	4.687890322	2.228939	9.2E-07	7.06E-06
LOC113247619	113247619	4.685299412	2.228141	2.21E-05	0.000128
ADSL	113241749	4.682097268	2.227155	8.61E-08	8.04E-07
KCNJ12	113271319	4.675115531	2.225002	1.92E-05	0.000113
CUTC	113251818	4.665234126	2.221949	9.21E-09	1.02E-07
COL11A2	113243790	4.660151595	2.220377	3.6E-05	0.000199

FAM124B	113250394	4.659211798	2.220086	0.000186	0.000874
SGIP1	113254558	4.646785809	2.216233	0.000167	0.000793
CES1	113252254	4.64672847	2.216215	4.11E-06	2.82E-05
LOC113256842	113256842	4.63743519	2.213327	6.71E-05	0.00035
LOC113248649	113248649	4.631006825	2.211326	7.68E-09	8.58E-08
ZNF358	113242937	4.63042649	2.211145	5.52E-06	3.69E-05
LOC113242741	113242741	4.627428619	2.210211	1.55E-06	1.14E-05
MPI	113240979	4.622105896	2.20855	3.32E-09	3.99E-08
PLEKHB1	113269294	4.617135661	2.206998	2.75E-05	0.000156
TRIP10	113242888	4.611715448	2.205303	4.22E-05	0.000229
NDUFV2	113253220	4.610394365	2.20489	1.13E-06	8.51E-06
MRPS5	113242194	4.607810707	2.204081	2.51E-07	2.13E-06
ATP5F1C	113241155	4.606158927	2.203564	0.000134	0.000653
CNGB1	113252297	4.605728598	2.203429	0.000224	0.00103
KLHL21	113270566	4.601924045	2.202237	2.21E-08	2.28E-07
CASKIN1	113245186	4.59980393	2.201572	4.62E-05	0.000249
DDAH1	113254466	4.593277413	2.199524	3.94E-06	2.71E-05
CCDC141	113250711	4.581733451	2.195894	1.16E-05	7.21E-05
CUNH11orf71	113260034	4.578405716	2.194845	0.000129	0.000628
MAP7D1	113268291	4.570537539	2.192364	8.82E-06	5.64E-05
PANK4	113270600	4.566698907	2.191152	5.44E-09	6.25E-08
NR1D2	113253869	4.562547466	2.18984	2.05E-05	0.00012
RHOBTB1	113259077	4.561696656	2.189571	3.58E-07	2.96E-06
RERGL	113257835	4.557411057	2.188215	0.000253	0.001147
LOC113245319	113245319	4.557249157	2.188163	0.002256	0.007816
MYPOP	113243407	4.551043222	2.186197	1.9E-06	1.38E-05
SYN1	113265974	4.545954548	2.184583	5.48E-05	0.000291
MAPRE3	113270924	4.535290107	2.181195	5.97E-05	0.000315

ATP5PB	113244431	4.535215669	2.181171	0.000435	0.00185
MRPS24	113257987	4.530834622	2.179777	5.94E-08	5.71E-07
ATP5PD	113244651	4.530074077	2.179535	0.000312	0.001383
HSF4	113252349	4.521528408	2.176811	0.000108	0.000536
PAM16	113270852	4.517055085	2.175383	4.24E-08	4.2E-07
CLIP1	113266923	4.516352661	2.175158	7.4E-06	4.82E-05
RASSF8	113257786	4.512926943	2.174063	1.95E-07	1.7E-06
LOC113264441	113264441	4.504072863	2.17123	5.39E-05	0.000286
VSIG10L	113247243	4.500321071	2.170028	8.84E-05	0.000449
ATP5F1B	113256410	4.498354309	2.169397	0.003664	0.011886
THBS2	113247125	4.494986643	2.168317	0.006076	0.018431
SLC2A4RG	113258822	4.49131406	2.167138	7.06E-06	4.61E-05
PYURF	113263153	4.489861547	2.166671	1.31E-07	1.18E-06
NDUFB8	113251835	4.489064163	2.166415	3.95E-06	2.72E-05
IMPDH2	113256856	4.48469474	2.16501	0.000203	0.000942
SMIM26	113258309	4.478474566	2.163007	9.85E-07	7.52E-06
ZNF579	113247353	4.477947008	2.162837	2.4E-08	2.46E-07
LEAP2	113261728	4.476806722	2.16247	0.000265	0.001195
TSLP	113254959	4.475269942	2.161975	0.000423	0.001808
KREMEN1	113245007	4.462405626	2.157822	1.69E-05	0.000101
SLC6A8	113247834	4.46073902	2.157283	1.89E-07	1.65E-06
MPPED2	113265079	4.455032552	2.155436	0.00019	0.000888
NFATC1	113253544	4.454581846	2.15529	4.64E-08	4.55E-07
SERHL2	113241696	4.454385124	2.155226	6.27E-05	0.00033
KCNMB2	113254860	4.449935514	2.153784	0.000377	0.001633
SMIM12	113268319	4.448409515	2.15329	1.17E-07	1.06E-06
DPH3	113253905	4.444907507	2.152153	2.72E-07	2.3E-06
ATP8B3	113242657	4.436335544	2.149368	0.000921	0.00358

DHDH	113243321	4.436195559	2.149323	0.001094	0.004163
DNAJB6	113241530	4.434751064	2.148853	7.28E-08	6.92E-07
NDUFC2	113269364	4.428130344	2.146698	8.04E-07	6.24E-06
FMO2	113262845	4.42664973	2.146215	2.12E-06	1.52E-05
RAB12	113253223	4.425734367	2.145917	1.39E-07	1.25E-06
OPTN	113241074	4.425208502	2.145745	1.15E-05	7.17E-05
ABCA5	113265229	4.420612284	2.144246	6.71E-08	6.41E-07
HMCN1	113262754	4.416665253	2.142957	3.74E-05	0.000206
GTF3A	113251205	4.412226894	2.141507	5.24E-08	5.1E-07
JUN	113254611	4.397946738	2.13683	1.88E-06	1.36E-05
SLC16A10	113264727	4.392778297	2.135134	4.82E-06	3.27E-05
NMS	113247084	4.39199225	2.134876	0.000262	0.001184
BTC	113263271	4.376733562	2.129855	0.000453	0.001918
SLC20A2	113245534	4.376228241	2.129688	1.02E-05	6.42E-05
PIGY	113263154	4.375063498	2.129304	5.61E-07	4.48E-06
KCNJ2	113265224	4.356441672	2.12315	0.000404	0.001739
RASGRF2	113255154	4.356300926	2.123104	6.31E-07	4.99E-06
CDC34	113242694	4.355551984	2.122856	1.63E-05	9.78E-05
ZNF32	113243142	4.34553578	2.119534	1.53E-06	1.13E-05
TRMT12	113252745	4.342817029	2.118631	5.25E-07	4.21E-06
ZNF30	113244783	4.342500574	2.118526	9.2E-06	5.86E-05
EGLN1	113259033	4.342314107	2.118464	7.61E-08	7.21E-07
PARD6A	113252374	4.33358364	2.115561	0.000371	0.001613
TBC1D4	113251422	4.329015358	2.114039	1.11E-07	1.01E-06
TMEM108	113253944	4.325687774	2.11293	5.28E-05	0.000282
LACTB2	113253065	4.321230847	2.111442	1.89E-07	1.65E-06
NDUFB6	113260540	4.314698538	2.10926	1.49E-06	1.1E-05
ALKBH3	113265015	4.314252031	2.10911	1.54E-08	1.64E-07

CHRM4	113264986	4.311736002	2.108269	0.00064	0.002609
MANEAL	113268270	4.307699492	2.106918	0.000112	0.000557
MEF2D	113245229	4.307003588	2.106685	5.42E-08	5.26E-07
SFXN4	113251991	4.306582053	2.106543	1.07E-07	9.78E-07
LIMCH1	113262439	4.304482081	2.10584	1.42E-05	8.63E-05
BBS5	113250801	4.302286775	2.105104	0.000195	0.000912
ZDHHC1	113252362	4.30146684	2.104829	1.42E-05	8.63E-05
PROCA1	113269040	4.296028313	2.103004	1.44E-06	1.07E-05
MRPL12	113244509	4.295070502	2.102682	2.59E-07	2.19E-06
EVA1C	113265647	4.290495384	2.101144	8.16E-05	0.000418
LOC113241107	113241107	4.285042243	2.099309	2.61E-05	0.000149
ECPAS	113260320	4.282533375	2.098464	0.000118	0.000583
DST	113261161	4.280889485	2.097911	3.36E-05	0.000187
EPHA4	113250413	4.278272915	2.097029	0.000117	0.000579
WNT10B	113257662	4.270749728	2.094489	0.000307	0.001367
APOO	113242225	4.270280369	2.094331	1.18E-06	8.89E-06
RASSF9	113256205	4.26931494	2.094005	0.000526	0.002195
TARSL2	113263620	4.267616578	2.093431	4.15E-07	3.4E-06
NDUFA6	113241708	4.266184849	2.092946	1.75E-07	1.54E-06
ZBTB16	113260036	4.24982606	2.087404	1.04E-06	7.94E-06
WNT4	113268542	4.248253429	2.08687	0.000584	0.00241
PLEKHM2	113270664	4.245444324	2.085916	0.000313	0.001389
NR4A2	113250870	4.242651123	2.084966	0.000452	0.001916
FHL5	113264813	4.239836849	2.084009	0.000607	0.002489
PGPEP1	113256715	4.234070833	2.082045	9.51E-08	8.78E-07
PHKB	113252209	4.23405246	2.082039	1.97E-06	1.42E-05
MLLT11	113246131	4.22653532	2.079476	4.41E-08	4.36E-07
R3HDM2	113256386	4.222824325	2.078208	4.6E-05	0.000248

KPNA4	113254139	4.22064132	2.077462	1.94E-05	0.000114
GPR17	113263488	4.220098925	2.077277	0.000415	0.001778
FGF1	113255360	4.218316536	2.076667	3.56E-05	0.000197
PDE8A	113263738	4.218252776	2.076646	6.17E-07	4.89E-06
WWP1	113252973	4.217108757	2.076254	1.89E-05	0.000112
KIF13A	113264500	4.216663332	2.076102	3.55E-06	2.46E-05
UBL4A	113247812	4.216429425	2.076022	3.99E-07	3.27E-06
PRRX1	113262848	4.209858507	2.073772	3.37E-05	0.000187
KLHL24	113254479	4.208477995	2.073299	2.56E-07	2.17E-06
ZFP37	113266304	4.203456897	2.071576	7.66E-05	0.000394
MMACHC	113254743	4.201090669	2.070764	1.09E-05	6.87E-05
ITGB4	113244620	4.193272817	2.068077	8.74E-06	5.6E-05
KLHL4	113242173	4.186142972	2.065622	0.000222	0.001026
ATP5F1D	113242724	4.181534895	2.064033	0.000108	0.000536
UBR3	113250793	4.176652239	2.062347	1.19E-05	7.41E-05
SEPT11	113263245	4.174966201	2.061765	1.72E-07	1.52E-06
LOC113271232	113271232	4.167411137	2.059151	0.000237	0.001084
MUL1	113268565	4.166976009	2.059001	2.21E-07	1.9E-06
HOXC8	113257531	4.165178301	2.058378	0.014055	0.037336
CARNMT1	113269844	4.164692229	2.05821	2.07E-07	1.79E-06
LOC113255247	113255247	4.16329449	2.057726	1.84E-05	0.000109
TEX2	113265274	4.162424807	2.057424	5.9E-06	3.92E-05
AMN1	113257748	4.162101096	2.057312	7.24E-07	5.67E-06
NFE2L1	113265569	4.161381194	2.057062	4.84E-05	0.00026
LOC113267950	113267950	4.159962606	2.056571	8.16E-05	0.000418
KRTCAP3	113271234	4.159795322	2.056513	8.86E-05	0.000449
PFN2	113254055	4.157565657	2.055739	5.87E-05	0.00031
TMEM136	113259949	4.155904664	2.055163	4.45E-06	3.03E-05

RELN	113258887	4.151154847	2.053513	5.59E-05	0.000296
FBXL22	113240838	4.150241397	2.053195	0.000116	0.000573
TOMM20	113259006	4.139755496	2.049546	1.45E-05	8.81E-05
TLN2	113269702	4.138270747	2.049028	9.14E-06	5.82E-05
PNPLA4	113240758	4.136742947	2.048495	6.59E-06	4.33E-05
CLIC5	113261239	4.135311168	2.047996	1.68E-05	0.0001
RRAGB	113247969	4.13393493	2.047516	3.66E-07	3.02E-06
FAM180B	113264952	4.13102556	2.0465	0.002374	0.008163
ANKRD9	113267237	4.130931855	2.046467	3.66E-07	3.01E-06
NDUFB7	113247157	4.129473203	2.045958	1.6E-07	1.42E-06
HSCB	113245003	4.129402962	2.045933	4.48E-07	3.64E-06
PABPC4	113268249	4.127203821	2.045165	0.000167	0.000795
ATP6V1G2	113243710	4.126183016	2.044808	0.000181	0.000857
FUNDC2	113248346	4.12503991	2.044408	5.79E-07	4.6E-06
TMEM161A	113256742	4.122038757	2.043358	1.39E-07	1.25E-06
RBFOX2	113255999	4.12072284	2.042897	1.52E-05	9.17E-05
SPATA24	113261807	4.118593142	2.042152	0.000184	0.000868
UBALD1	113270843	4.111761441	2.039757	1.07E-07	9.81E-07
COL4A4	113250384	4.107943586	2.038416	0.001005	0.00387
ATP5ME	113245737	4.098735652	2.035179	4.14E-05	0.000225
AGL	113254363	4.097772001	2.03484	9.53E-05	0.00048
NDUFS2	113246718	4.095137646	2.033912	0.000278	0.001247
SMIM1	113270590	4.08809008	2.031427	1.98E-07	1.72E-06
LOC113248801	113248801	4.087076884	2.031069	0.000958	0.003708
FAM131A	113254262	4.086332039	2.030806	7.7E-07	5.99E-06
RGS5	113268681	4.07691845	2.027479	3.76E-05	0.000207
CAMSAP1	113266592	4.075745948	2.027064	8.14E-08	7.65E-07
TMCC3	113256152	4.074911414	2.026769	3.74E-05	0.000206

NDUFS1	113250544	4.066890228	2.023926	0.000103	0.000516
PER2	113249994	4.058257955	2.020861	3.27E-06	2.27E-05
RDH14	113270195	4.053619162	2.019211	1.92E-06	1.39E-05
EGR1	113261792	4.051715409	2.018533	6.34E-06	4.18E-05
ASB8	113257679	4.049849317	2.017868	3.98E-07	3.27E-06
CUL5	113260213	4.049377129	2.0177	8.47E-08	7.92E-07
HIVEP2	113259505	4.046843153	2.016797	1.62E-08	1.72E-07
NDUFB4	113252045	4.043033156	2.015438	1.81E-06	1.32E-05
LOC113262535	113262535	4.037970685	2.01363	5.29E-06	3.55E-05
MFF	113250383	4.036782188	2.013206	1.26E-05	7.77E-05
AKAP1	113268847	4.033968049	2.0122	1.39E-06	1.03E-05
LOC113249853	113249853	4.020081521	2.007225	0.001319	0.004908
TSPAN15	113258877	4.019772276	2.007114	0.000661	0.002682
UBXN6	113242833	4.01906	2.006858	5.3E-05	0.000282
DNAJC19	113254646	4.012187675	2.004389	2.02E-06	1.45E-05
NREP	113254998	4.002028071	2.000731	9.66E-06	6.12E-05
CCDC43	113265348	3.99904936	1.999657	2.42E-07	2.07E-06
GPR21	113266387	3.998009682	1.999282	0.000501	0.002103
FBXO31	113252169	3.997940902	1.999257	2.87E-05	0.000162
TTC33	113260928	3.997247036	1.999007	9.87E-07	7.53E-06
CAMK2G	113259192	3.996334056	1.998677	3.96E-05	0.000216
DMD	113265965	3.993562762	1.997676	5.4E-05	0.000287
ZNF770	113242069	3.993032363	1.997485	3.71E-07	3.05E-06
HMGN3	113261087	3.992598334	1.997328	4.32E-07	3.53E-06
LOC113247902	113247902	3.989548809	1.996226	0.000607	0.002491
CHRNA1	113250753	3.988745087	1.995935	0.000228	0.001045
COQ7	113270738	3.988084658	1.995696	3.95E-06	2.72E-05
SLC25A20	113256849	3.987713349	1.995562	1.35E-05	8.27E-05

LZTS3	113258399	3.985565032	1.994784	8.66E-05	0.000441
IDH3B	113258411	3.982968404	1.993844	5.09E-05	0.000273
TWF2	113256944	3.975715508	1.991215	7E-05	0.000364
IGDCC4	113241013	3.97353948	1.990425	2.47E-07	2.11E-06
PDLIM7	113264157	3.970432078	1.989296	6.55E-07	5.17E-06
NDUFAF5	113258328	3.96883445	1.988715	2.75E-05	0.000156
HOXC9	113257530	3.956446757	1.984205	0.002072	0.007251
LOC113246178	113246178	3.952464885	1.982753	1.64E-07	1.46E-06
ISYNA1	113256718	3.950539913	1.98205	7.59E-05	0.000391
LOC113254727	113254727	3.950483699	1.982029	0.000105	0.000525
ATF3	113262677	3.950300408	1.981962	3.97E-05	0.000217
LOC113243815	113243815	3.946587306	1.980606	2.02E-06	1.46E-05
HADHB	113270781	3.942160329	1.978986	0.000617	0.002527
EIF4EBP1	113262099	3.941308331	1.978675	5.23E-06	3.51E-05
DEPTOR	113252772	3.939841863	1.978138	5.72E-06	3.81E-05
LOC113248580	113248580	3.930596699	1.974748	1.38E-05	8.43E-05
ZNF503	113259209	3.928341924	1.973921	5.47E-07	4.37E-06
KIF26A	113267199	3.927020843	1.973435	1.74E-05	0.000104
LOC113246863	113246863	3.926702902	1.973318	0.001765	0.006308
TRMT2B	113244297	3.924733079	1.972595	0.000265	0.001197
PFDN1	113261816	3.922935319	1.971934	3.73E-07	3.07E-06
COQ5	113267040	3.921745646	1.971496	7.52E-07	5.86E-06
TTC19	113271258	3.915333658	1.969135	3.47E-07	2.88E-06
ZSCAN9	113248163	3.911955053	1.96789	0.000313	0.001388
RASSF4	113243126	3.906260256	1.965788	4.1E-06	2.81E-05
NDUFB2	113265140	3.903614752	1.964811	7.65E-06	4.96E-05
LOC113259016	113259016	3.900719432	1.96374	6.57E-06	4.32E-05
INPP5E	113248636	3.897670056	1.962612	4.61E-05	0.000248

LOC113267907	113267907	3.896845459	1.962307	0.000295	0.001318
LOC113247923	113247923	3.894579431	1.961468	0.000349	0.001527
BLCAP	113258579	3.894471648	1.961428	0.000213	0.000988
DMAC1	113260618	3.885760421	1.958197	5E-07	4.02E-06
LOC113246288	113246288	3.879045538	1.955702	0.001244	0.004663
ADAMTS1	113269052	3.876470993	1.954744	5.07E-06	3.42E-05
NFKBIL1	113243709	3.872627078	1.953313	9.84E-07	7.52E-06
CRY2	113264999	3.867975785	1.951579	6.19E-06	4.1E-05
SOX7	113269935	3.85541899	1.946888	0.000346	0.001515
ADGRA3	113266735	3.853370354	1.946121	1.29E-06	9.64E-06
ADRA2C	113245678	3.850523892	1.945055	0.00389	0.012499
TCHH	113246136	3.849282321	1.944589	0.007877	0.022912
ACCSL	113265013	3.845553963	1.943191	0.000644	0.002623
CACNA1D	113256986	3.844295277	1.942719	0.00033	0.001453
FMO1	113262844	3.843777181	1.942525	0.002975	0.009955
LOC113261786	113261786	3.83742467	1.940138	0.000541	0.002248
UBE2G1	113271043	3.836993523	1.939976	0.000127	0.000618
SLC16A11	113271101	3.833046882	1.938492	0.000871	0.003415
BLOC1S1	113256461	3.830454079	1.937515	3.78E-05	0.000208
RHOBTB3	113255075	3.829117057	1.937012	2.74E-05	0.000156
FILIP1L	113250483	3.826354623	1.935971	5.56E-07	4.44E-06
NIM1K	113260953	3.825634634	1.935699	0.00321	0.010609
TMEM252	113270191	3.824024607	1.935092	0.003358	0.011027
MIB1	113253275	3.823556501	1.934915	1.43E-05	8.7E-05
ZNF185	113246825	3.821878687	1.934282	0.006224	0.018816
SDHD	113260049	3.818504196	1.933008	0.000135	0.000653
TRAK1	113253818	3.814270762	1.931407	2.07E-05	0.000121
CSDE1	113244393	3.812466253	1.930725	0.007437	0.021806

NUDT8	113244016	3.810721889	1.930064	0.000197	0.000917
RPP40	113264415	3.809678004	1.929669	8.55E-05	0.000436
RNF128	113241221	3.809557321	1.929623	0.010747	0.029637
TMEM185A	113241899	3.808124663	1.929081	1.26E-07	1.14E-06
NDUFV1	113244014	3.805938183	1.928252	9.2E-05	0.000464
CST6	113244082	3.805647596	1.928142	0.000113	0.000562
MRPL45	113265550	3.804602253	1.927746	1.05E-05	6.61E-05
NT5C3A	113262512	3.804600039	1.927745	9.17E-06	5.84E-05
MYOC	113262839	3.803393309	1.927287	0.016194	0.042128
LOC113248593	113248593	3.796438562	1.924647	6.74E-07	5.3E-06
DNAJB1	113247153	3.795523186	1.924299	5.77E-07	4.59E-06
LOC113260369	113260369	3.79468662	1.923981	1.12E-05	7.02E-05
ADAMTSL2	113266573	3.794410633	1.923876	0.000177	0.000838
PREPL	113246215	3.793631641	1.92358	3.1E-07	2.59E-06
ATP5MF	113267913	3.792405215	1.923113	2.22E-06	1.59E-05
TBRG4	113257719	3.784260079	1.920011	2.91E-05	0.000165
GRASP	113257465	3.782491444	1.919337	5.44E-05	0.000289
MSI2	113268852	3.77986431	1.918334	3.61E-07	2.98E-06
TMEM11	113271315	3.779381716	1.91815	1.14E-06	8.62E-06
NDUFAF2	113255305	3.775868104	1.916808	0.000761	0.003036
NDUFS6	113260793	3.774786582	1.916395	3.06E-06	2.14E-05
GOLGA4	113253773	3.770632602	1.914807	7.78E-05	0.0004
CALR3	113256650	3.770474155	1.914746	0.00055	0.00228
DEXI	113270801	3.767847945	1.913741	8.17E-07	6.34E-06
LOC113246871	113246871	3.763990755	1.912263	2.32E-06	1.65E-05
LOC113264253	113264253	3.762002117	1.911501	0.000197	0.000917
TMOD1	113260426	3.761371318	1.911259	0.00014	0.000676
VAMP2	113271170	3.761354847	1.911252	3.85E-06	2.65E-05

PPM1L	113254142	3.758561175	1.91018	2.06E-05	0.00012
NOS3	113241474	3.757553192	1.909794	1.25E-05	7.72E-05
DMAC2L	113266076	3.75632709	1.909323	7.66E-05	0.000394
REPIN1	113241464	3.753905154	1.908392	1.61E-07	1.43E-06
CELF5	113242796	3.752849238	1.907986	0.00181	0.006454
EPB41L4B	113260640	3.749679863	1.906767	0.000149	0.000715
AMACR	113260868	3.748598083	1.906351	1.66E-05	9.9E-05
CUNH3orf18	113256920	3.74835387	1.906257	0.00078	0.003101
TMEM184A	113247730	3.742373001	1.903953	0.00104	0.003981
IGFALS	113245094	3.742304539	1.903927	1.03E-05	6.52E-05
SMIM20	113266763	3.740271041	1.903143	9.98E-06	6.31E-05
SBDS	113267760	3.740005167	1.90304	5.13E-06	3.45E-05
KCNN3	113245302	3.732582874	1.900174	0.000389	0.001682
OXA1L	113246043	3.729845145	1.899116	0.000117	0.000576
LCMT1	113267594	3.72913362	1.89884	1.2E-06	8.98E-06
SLC4A3	113250414	3.728332035	1.89853	0.000489	0.002057
HEYL	113268247	3.728261085	1.898503	5.07E-07	4.07E-06
LOC113246846	113246846	3.716889029	1.894096	6.56E-06	4.32E-05
LRRC43	113266928	3.71688257	1.894093	0.001059	0.004045
TEF	113241732	3.714435706	1.893143	6.57E-06	4.32E-05
MALL	113242244	3.710609646	1.891656	0.00064	0.002609
MEIOC	113265676	3.708354466	1.890779	0.000312	0.001383
TSNARE1	113247953	3.700501604	1.887721	0.000125	0.000611
LOC113247922	113247922	3.695477365	1.885761	1.27E-05	7.8E-05
POMGNT1	113254729	3.692434443	1.884572	8.11E-06	5.22E-05
GYG1	113254042	3.691748289	1.884304	0.000867	0.003403
CUNH6orf106	113243827	3.690584246	1.883849	0.000256	0.00116
LOC113242783	113242783	3.684791335	1.881583	0.000235	0.001076

LOC113254636	113254636	3.681904493	1.880452	3.77E-06	2.6E-05
NDUFA4L2	113256390	3.677301083	1.878647	0.001745	0.006249
LOC113247877	113247877	3.676961928	1.878514	0.001742	0.006242
POLDIP2	113269025	3.676221558	1.878224	4.13E-06	2.82E-05
CDKL5	113242254	3.675688127	1.878014	0.00034	0.00149
LOC113241423	113241423	3.667294269	1.874716	0.000627	0.00256
ACTR3B	113241507	3.657492366	1.870855	1.11E-06	8.39E-06
EEF1D	113247655	3.65569526	1.870146	0.001422	0.005241
RASD2	113256004	3.653705827	1.86936	0.001031	0.003954
CATSPERD	113242861	3.65150505	1.868491	0.001273	0.004759
ATPAF1	113254713	3.650832753	1.868226	1.08E-06	8.19E-06
SPOCK2	113259155	3.650575148	1.868124	2.17E-05	0.000126
ROMO1	113258545	3.65005854	1.86792	4.67E-06	3.17E-05
ZMAT5	113245019	3.645539942	1.866133	2.27E-05	0.000131
INSYN1	113240951	3.644654032	1.865782	0.003466	0.011343
LRRTM2	113261797	3.644102694	1.865564	0.002992	0.009998
DCAF6	113262875	3.634561826	1.861781	0.000659	0.002677
CUNH9orf16	113266457	3.63428294	1.861671	3.14E-05	0.000175
ZNF391	113264258	3.632274446	1.860873	2.25E-05	0.00013
CCDC136	113263909	3.631012608	1.860372	4.25E-05	0.00023
ZYG11B	113254661	3.626846199	1.858716	4E-05	0.000218
MRPL21	113243993	3.615653444	1.854256	8.04E-06	5.18E-05
RAPGEF1	113266537	3.614830168	1.853928	1.72E-05	0.000103
FASTK	113241480	3.611712036	1.852683	7.63E-06	4.95E-05
NOX4	113269421	3.61000922	1.852003	0.002226	0.007732
LOC113247021	113247021	3.606306538	1.850522	0.001393	0.005149
YPEL2	113268888	3.605804345	1.850321	2.2E-05	0.000128
AVPI1	113251796	3.603725364	1.849489	0.000616	0.002525

PLEKHH3	113265409	3.603014788	1.849205	3.91E-06	2.69E-05
EML1	113267272	3.601332967	1.848531	0.000235	0.001077
PIH1D1	113243302	3.599848866	1.847936	2.04E-06	1.47E-05
CGNL1	113269670	3.59961167	1.847841	2.86E-05	0.000162
ANO5	113265108	3.598247884	1.847295	0.006651	0.019871
GMPS	113254098	3.593739088	1.845486	0.000134	0.000651
SLC7A6OS	113252408	3.592077977	1.844819	1.13E-05	7.03E-05
NDUFB5	113254753	5.182254423	2.37358	6.48E-09	7.35E-08
NDUFS4	113263912	5.180952033	2.373217	6.79E-08	6.49E-07
PDE4B	113254561	5.176978039	2.37211	3.17E-07	2.64E-06
ACYP2	113245706	5.16460707	2.368659	1.02E-05	6.45E-05
TAGLN	113260017	5.162970495	2.368201	1.82E-07	1.6E-06
JAG2	113267148	5.154148089	2.365734	4.1E-09	4.82E-08
MRPL33	113240736	5.14920264	2.364349	5.08E-08	4.95E-07
KIAA1217	113241331	5.142682744	2.362521	9.76E-10	1.31E-08
BZW2	113261015	5.142298357	2.362413	2.76E-07	2.33E-06
MAP3K7CL	113267437	5.125506677	2.357695	2.11E-06	1.52E-05
FAM83C	113258536	5.124313321	2.357359	1.91E-05	0.000112
ACTA2	113258969	5.116930042	2.355279	1.19E-09	1.57E-08
RNF225	113248032	5.116265534	2.355091	7.65E-05	0.000394
LOC113244568	113244568	5.109196029	2.353096	8.37E-05	0.000427
LOC113266806	113266806	5.09912202	2.350249	0.002569	0.008747
VDAC3	113245581	5.097905706	2.349905	4.07E-05	0.000221
SULF2	113258690	5.091898189	2.348204	1.15E-06	8.66E-06
LOC113254515	113254515	5.08986265	2.347627	0.000189	0.000887
MAFF	113241783	5.088669802	2.347289	3.79E-08	3.77E-07
NDUFA8	113266369	5.088070083	2.347119	1.56E-06	1.15E-05
DNASE1L1	113247843	5.041284203	2.333791	7.82E-07	6.08E-06

NHLRC1	113264504	5.032565712	2.331294	1.38E-05	8.46E-05
DKK3	113246270	5.001037803	2.322228	0.00013	0.000631
IRAK1BP1	113261094	4.993969694	2.320187	2.14E-06	1.54E-05
HOXA7	113262001	4.990864408	2.31929	5.66E-07	4.51E-06
ARPP19	113269638	4.98805239	2.318477	2.94E-06	2.06E-05
LOC113248616	113248616	4.982546716	2.316883	2.11E-08	2.18E-07
LOC113252908	113252908	4.973140037	2.314157	1.29E-05	7.93E-05
IMMP2L	113262909	4.971730147	2.313748	3.44E-07	2.85E-06
SUSD4	113262898	4.968029602	2.312674	0.000622	0.002543
PDK2	113265612	4.967713081	2.312582	3.65E-06	2.52E-05
ATP5MPL	113267204	4.958596821	2.309932	2.1E-06	1.51E-05
ACADS	113267053	4.957977519	2.309752	2.71E-06	1.91E-05
LYSMD1	113246163	4.952779437	2.308238	6.55E-08	6.27E-07
SDHB	113270447	4.950619086	2.307609	1.91E-06	1.38E-05
HIF3A	113243399	4.946625659	2.306445	0.00019	0.000888
VDAC1	113261734	4.94236777	2.305202	0.000139	0.000671
HOMER3	113256738	4.941869015	2.305057	3.26E-09	3.93E-08
IGSF23	113243462	4.918616999	2.298253	4.02E-05	0.000219
MFN2	113270495	4.918232638	2.29814	6.26E-06	4.14E-05
CUEDC1	113268859	4.908684331	2.295336	7.67E-08	7.26E-07
CYR61	113254464	4.908344555	2.295237	1.77E-08	1.86E-07
TEX29	113248531	4.904636985	2.294146	8.51E-06	5.46E-05
LOC113257882	113257882	4.90434679	2.294061	0.000108	0.000539
NDUFA9	113257995	4.898221765	2.292258	1.11E-06	8.39E-06
ATP5MC2	113257544	4.896569563	2.291771	2.28E-05	0.000132
SLC25A11	113271067	4.883670965	2.287966	1.78E-06	1.3E-05
PPP1R3F	113265953	4.880940524	2.287159	1.24E-06	9.29E-06
MAGIX	113265825	4.876353542	2.285803	2.25E-07	1.93E-06

KHDRBS3	113252694	4.873685864	2.285013	0.000129	0.000629
SYT7	113246992	4.870581233	2.284094	8.28E-07	6.41E-06
SLC25A30	113251350	4.87050487	2.284071	1.72E-08	1.81E-07
SUCLG1	113243306	4.869676356	2.283826	8.45E-05	0.000431
NEU3	113269322	4.864477853	2.282285	8.99E-09	9.98E-08
LOC113261568	113261568	4.863444805	2.281979	1.16E-08	1.26E-07
TOB1	113265636	4.861364096	2.281361	6.93E-08	6.61E-07
SVIL	113244243	4.860904544	2.281225	3.36E-05	0.000187
CIPC	113270299	4.860780052	2.281188	3.53E-09	4.21E-08
CFAP54	113256123	4.850251988	2.27806	0.000331	0.001457
TNFRSF12A	113245425	4.846130893	2.276833	0.000362	0.001577
KIAA1755	113258589	4.842617008	2.275787	8.77E-05	0.000446
ATP5F1A	113253374	4.840685563	2.275211	0.001838	0.006532
TRIM45	113244373	4.840650775	2.275201	2.45E-08	2.5E-07
LOC113257633	113257633	4.83940976	2.274831	3.44E-09	4.12E-08
TENM1	113270934	4.835496757	2.273664	0.000142	0.000685
ZSCAN12	113248152	4.815036701	2.267547	1.75E-07	1.54E-06
LOC113244843	113244843	4.813134009	2.266977	6.72E-06	4.41E-05
LOC113258460	113258460	4.808523112	2.265594	9.73E-05	0.000489
HOXD9	113250743	4.807298689	2.265226	0.0002	0.000929
NDUFB11	113265993	4.801283092	2.26342	8.75E-07	6.74E-06
LOC113249623	113249623	4.791451023	2.260463	0.012365	0.03346
ITIH3	113256968	4.786123389	2.258858	0.000112	0.000555
PPP3CB	113259177	4.78374038	2.258139	4.94E-07	3.98E-06
INHBB	113263463	4.775124396	2.255538	6.33E-05	0.000332
LOC113251521	113251521	4.766539513	2.252942	0.002711	0.009173
FMC1	113264958	4.754493474	2.249292	2.01E-09	2.54E-08
CISD1	113259063	4.752934296	2.248818	1.33E-06	9.91E-06

COMP	113256730	4.744476845	2.246249	0.009866	0.027616
CLCN4	113240744	4.73774328	2.2442	3E-07	2.51E-06
CHST10	113247050	4.734594766	2.243241	5.93E-09	6.77E-08
CCDC28B	113268366	4.726893461	2.240892	7.64E-06	4.96E-05
EFCC1	113257241	4.726197745	2.24068	3.25E-05	0.000181
TGFB2	113268733	4.711644914	2.236231	1.79E-08	1.88E-07
LOC113246281	113246281	4.706216007	2.234568	0.000409	0.001754
SLC14A2	113253366	4.698756455	2.232279	0.000488	0.002054
ABLIM1	113251961	4.697014479	2.231744	2.1E-05	0.000122
NFIC	113242797	4.687890322	2.228939	9.2E-07	7.06E-06
LOC113247619	113247619	4.685299412	2.228141	2.21E-05	0.000128
ADSL	113241749	4.682097268	2.227155	8.61E-08	8.04E-07
KCNJ12	113271319	4.675115531	2.225002	1.92E-05	0.000113
CUTC	113251818	4.665234126	2.221949	9.21E-09	1.02E-07
COL11A2	113243790	4.660151595	2.220377	3.6E-05	0.000199
FAM124B	113250394	4.659211798	2.220086	0.000186	0.000874
SGIP1	113254558	4.646785809	2.216233	0.000167	0.000793
CES1	113252254	4.64672847	2.216215	4.11E-06	2.82E-05
LOC113256842	113256842	4.63743519	2.213327	6.71E-05	0.00035
LOC113248649	113248649	4.631006825	2.211326	7.68E-09	8.58E-08
ZNF358	113242937	4.63042649	2.211145	5.52E-06	3.69E-05
LOC113242741	113242741	4.627428619	2.210211	1.55E-06	1.14E-05
MPI	113240979	4.622105896	2.20855	3.32E-09	3.99E-08
PLEKHB1	113269294	4.617135661	2.206998	2.75E-05	0.000156
TRIP10	113242888	4.611715448	2.205303	4.22E-05	0.000229
NDUFV2	113253220	4.610394365	2.20489	1.13E-06	8.51E-06
MRPS5	113242194	4.607810707	2.204081	2.51E-07	2.13E-06
ATP5F1C	113241155	4.606158927	2.203564	0.000134	0.000653

CNGB1	113252297	4.605728598	2.203429	0.000224	0.00103
KLHL21	113270566	4.601924045	2.202237	2.21E-08	2.28E-07
CASKIN1	113245186	4.59980393	2.201572	4.62E-05	0.000249
DDAH1	113254466	4.593277413	2.199524	3.94E-06	2.71E-05
CCDC141	113250711	4.581733451	2.195894	1.16E-05	7.21E-05
CUNH11orf71	113260034	4.578405716	2.194845	0.000129	0.000628
MAP7D1	113268291	4.570537539	2.192364	8.82E-06	5.64E-05
PANK4	113270600	4.566698907	2.191152	5.44E-09	6.25E-08
NR1D2	113253869	4.562547466	2.18984	2.05E-05	0.00012
RHOBTB1	113259077	4.561696656	2.189571	3.58E-07	2.96E-06
RERGL	113257835	4.557411057	2.188215	0.000253	0.001147
LOC113245319	113245319	4.557249157	2.188163	0.002256	0.007816
MYPOP	113243407	4.551043222	2.186197	1.9E-06	1.38E-05
SYN1	113265974	4.545954548	2.184583	5.48E-05	0.000291
MAPRE3	113270924	4.535290107	2.181195	5.97E-05	0.000315
ATP5PB	113244431	4.535215669	2.181171	0.000435	0.00185
MRPS24	113257987	4.530834622	2.179777	5.94E-08	5.71E-07
ATP5PD	113244651	4.530074077	2.179535	0.000312	0.001383
HSF4	113252349	4.521528408	2.176811	0.000108	0.000536
PAM16	113270852	4.517055085	2.175383	4.24E-08	4.2E-07
CLIP1	113266923	4.516352661	2.175158	7.4E-06	4.82E-05
RASSF8	113257786	4.512926943	2.174063	1.95E-07	1.7E-06
LOC113264441	113264441	4.504072863	2.17123	5.39E-05	0.000286
VSIG10L	113247243	4.500321071	2.170028	8.84E-05	0.000449
ATP5F1B	113256410	4.498354309	2.169397	0.003664	0.011886
THBS2	113247125	4.494986643	2.168317	0.006076	0.018431
SLC2A4RG	113258822	4.49131406	2.167138	7.06E-06	4.61E-05
PYURF	113263153	4.489861547	2.166671	1.31E-07	1.18E-06

NDUFB8	113251835	4.489064163	2.166415	3.95E-06	2.72E-05
IMPDH2	113256856	4.48469474	2.16501	0.000203	0.000942
SMIM26	113258309	4.478474566	2.163007	9.85E-07	7.52E-06
ZNF579	113247353	4.477947008	2.162837	2.4E-08	2.46E-07
LEAP2	113261728	4.476806722	2.16247	0.000265	0.001195
TSLP	113254959	4.475269942	2.161975	0.000423	0.001808
KREMEN1	113245007	4.462405626	2.157822	1.69E-05	0.000101
SLC6A8	113247834	4.46073902	2.157283	1.89E-07	1.65E-06
MPPED2	113265079	4.455032552	2.155436	0.00019	0.000888
NFATC1	113253544	4.454581846	2.15529	4.64E-08	4.55E-07
SERHL2	113241696	4.454385124	2.155226	6.27E-05	0.00033
KCNMB2	113254860	4.449935514	2.153784	0.000377	0.001633
SMIM12	113268319	4.448409515	2.15329	1.17E-07	1.06E-06
DPH3	113253905	4.444907507	2.152153	2.72E-07	2.3E-06
ATP8B3	113242657	4.436335544	2.149368	0.000921	0.00358
DHDH	113243321	4.436195559	2.149323	0.001094	0.004163
DNAJB6	113241530	4.434751064	2.148853	7.28E-08	6.92E-07
NDUFC2	113269364	4.428130344	2.146698	8.04E-07	6.24E-06
FMO2	113262845	4.42664973	2.146215	2.12E-06	1.52E-05
RAB12	113253223	4.425734367	2.145917	1.39E-07	1.25E-06
OPTN	113241074	4.425208502	2.145745	1.15E-05	7.17E-05
ABCA5	113265229	4.420612284	2.144246	6.71E-08	6.41E-07
HMCN1	113262754	4.416665253	2.142957	3.74E-05	0.000206
GTF3A	113251205	4.412226894	2.141507	5.24E-08	5.1E-07
JUN	113254611	4.397946738	2.13683	1.88E-06	1.36E-05
SLC16A10	113264727	4.392778297	2.135134	4.82E-06	3.27E-05
NMS	113247084	4.39199225	2.134876	0.000262	0.001184
BTC	113263271	4.376733562	2.129855	0.000453	0.001918

SLC20A2	113245534	4.376228241	2.129688	1.02E-05	6.42E-05
PIGY	113263154	4.375063498	2.129304	5.61E-07	4.48E-06
KCNJ2	113265224	4.356441672	2.12315	0.000404	0.001739
RASGRF2	113255154	4.356300926	2.123104	6.31E-07	4.99E-06
CDC34	113242694	4.355551984	2.122856	1.63E-05	9.78E-05
ZNF32	113243142	4.34553578	2.119534	1.53E-06	1.13E-05
TRMT12	113252745	4.342817029	2.118631	5.25E-07	4.21E-06
ZNF30	113244783	4.342500574	2.118526	9.2E-06	5.86E-05
EGLN1	113259033	4.342314107	2.118464	7.61E-08	7.21E-07
PARD6A	113252374	4.33358364	2.115561	0.000371	0.001613
TBC1D4	113251422	4.329015358	2.114039	1.11E-07	1.01E-06
TMEM108	113253944	4.325687774	2.11293	5.28E-05	0.000282
LACTB2	113253065	4.321230847	2.111442	1.89E-07	1.65E-06
NDUFB6	113260540	4.314698538	2.10926	1.49E-06	1.1E-05
ALKBH3	113265015	4.314252031	2.10911	1.54E-08	1.64E-07
CHRM4	113264986	4.311736002	2.108269	0.00064	0.002609
MANEAL	113268270	4.307699492	2.106918	0.000112	0.000557
MEF2D	113245229	4.307003588	2.106685	5.42E-08	5.26E-07
SFXN4	113251991	4.306582053	2.106543	1.07E-07	9.78E-07
LIMCH1	113262439	4.304482081	2.10584	1.42E-05	8.63E-05
BBS5	113250801	4.302286775	2.105104	0.000195	0.000912
ZDHHC1	113252362	4.30146684	2.104829	1.42E-05	8.63E-05
PROCA1	113269040	4.296028313	2.103004	1.44E-06	1.07E-05
MRPL12	113244509	4.295070502	2.102682	2.59E-07	2.19E-06
EVA1C	113265647	4.290495384	2.101144	8.16E-05	0.000418
LOC113241107	113241107	4.285042243	2.099309	2.61E-05	0.000149
ECPAS	113260320	4.282533375	2.098464	0.000118	0.000583
DST	113261161	4.280889485	2.097911	3.36E-05	0.000187

EPHA4	113250413	4.278272915	2.097029	0.000117	0.000579
WNT10B	113257662	4.270749728	2.094489	0.000307	0.001367
APOO	113242225	4.270280369	2.094331	1.18E-06	8.89E-06
RASSF9	113256205	4.26931494	2.094005	0.000526	0.002195
TARSL2	113263620	4.267616578	2.093431	4.15E-07	3.4E-06
NDUFA6	113241708	4.266184849	2.092946	1.75E-07	1.54E-06
ZBTB16	113260036	4.24982606	2.087404	1.04E-06	7.94E-06
WNT4	113268542	4.248253429	2.08687	0.000584	0.00241
PLEKHM2	113270664	4.245444324	2.085916	0.000313	0.001389
NR4A2	113250870	4.242651123	2.084966	0.000452	0.001916
FHL5	113264813	4.239836849	2.084009	0.000607	0.002489
PGPEP1	113256715	4.234070833	2.082045	9.51E-08	8.78E-07
PHKB	113252209	4.23405246	2.082039	1.97E-06	1.42E-05
MLLT11	113246131	4.22653532	2.079476	4.41E-08	4.36E-07
R3HDM2	113256386	4.222824325	2.078208	4.6E-05	0.000248
KPNA4	113254139	4.22064132	2.077462	1.94E-05	0.000114
GPR17	113263488	4.220098925	2.077277	0.000415	0.001778
FGF1	113255360	4.218316536	2.076667	3.56E-05	0.000197
PDE8A	113263738	4.218252776	2.076646	6.17E-07	4.89E-06
WWP1	113252973	4.217108757	2.076254	1.89E-05	0.000112
KIF13A	113264500	4.216663332	2.076102	3.55E-06	2.46E-05
UBL4A	113247812	4.216429425	2.076022	3.99E-07	3.27E-06
PRRX1	113262848	4.209858507	2.073772	3.37E-05	0.000187
KLHL24	113254479	4.208477995	2.073299	2.56E-07	2.17E-06
ZFP37	113266304	4.203456897	2.071576	7.66E-05	0.000394
MMACHC	113254743	4.201090669	2.070764	1.09E-05	6.87E-05
ITGB4	113244620	4.193272817	2.068077	8.74E-06	5.6E-05
KLHL4	113242173	4.186142972	2.065622	0.000222	0.001026

ATP5F1D	113242724	4.181534895	2.064033	0.000108	0.000536
UBR3	113250793	4.176652239	2.062347	1.19E-05	7.41E-05
SEPT11	113263245	4.174966201	2.061765	1.72E-07	1.52E-06
LOC113271232	113271232	4.167411137	2.059151	0.000237	0.001084
MUL1	113268565	4.166976009	2.059001	2.21E-07	1.9E-06
HOXC8	113257531	4.165178301	2.058378	0.014055	0.037336
CARNMT1	113269844	4.164692229	2.05821	2.07E-07	1.79E-06
LOC113255247	113255247	4.16329449	2.057726	1.84E-05	0.000109
TEX2	113265274	4.162424807	2.057424	5.9E-06	3.92E-05
AMN1	113257748	4.162101096	2.057312	7.24E-07	5.67E-06
NFE2L1	113265569	4.161381194	2.057062	4.84E-05	0.00026
LOC113267950	113267950	4.159962606	2.056571	8.16E-05	0.000418
KRTCAP3	113271234	4.159795322	2.056513	8.86E-05	0.000449
PFN2	113254055	4.157565657	2.055739	5.87E-05	0.00031
TMEM136	113259949	4.155904664	2.055163	4.45E-06	3.03E-05
RELN	113258887	4.151154847	2.053513	5.59E-05	0.000296
FBXL22	113240838	4.150241397	2.053195	0.000116	0.000573
TOMM20	113259006	4.139755496	2.049546	1.45E-05	8.81E-05
TLN2	113269702	4.138270747	2.049028	9.14E-06	5.82E-05
PNPLA4	113240758	4.136742947	2.048495	6.59E-06	4.33E-05
CLIC5	113261239	4.135311168	2.047996	1.68E-05	0.0001
RRAGB	113247969	4.13393493	2.047516	3.66E-07	3.02E-06
FAM180B	113264952	4.13102556	2.0465	0.002374	0.008163
ANKRD9	113267237	4.130931855	2.046467	3.66E-07	3.01E-06
NDUFB7	113247157	4.129473203	2.045958	1.6E-07	1.42E-06
HSCB	113245003	4.129402962	2.045933	4.48E-07	3.64E-06
PABPC4	113268249	4.127203821	2.045165	0.000167	0.000795
ATP6V1G2	113243710	4.126183016	2.044808	0.000181	0.000857

FUNDC2	113248346	4.12503991	2.044408	5.79E-07	4.6E-06
TMEM161A	113256742	4.122038757	2.043358	1.39E-07	1.25E-06
RBFOX2	113255999	4.12072284	2.042897	1.52E-05	9.17E-05
SPATA24	113261807	4.118593142	2.042152	0.000184	0.000868
UBALD1	113270843	4.111761441	2.039757	1.07E-07	9.81E-07
COL4A4	113250384	4.107943586	2.038416	0.001005	0.00387
ATP5ME	113245737	4.098735652	2.035179	4.14E-05	0.000225
AGL	113254363	4.097772001	2.03484	9.53E-05	0.00048
NDUFS2	113246718	4.095137646	2.033912	0.000278	0.001247
SMIM1	113270590	4.08809008	2.031427	1.98E-07	1.72E-06
LOC113248801	113248801	4.087076884	2.031069	0.000958	0.003708
FAM131A	113254262	4.086332039	2.030806	7.7E-07	5.99E-06
RGS5	113268681	4.07691845	2.027479	3.76E-05	0.000207
CAMSAP1	113266592	4.075745948	2.027064	8.14E-08	7.65E-07
TMCC3	113256152	4.074911414	2.026769	3.74E-05	0.000206
NDUFS1	113250544	4.066890228	2.023926	0.000103	0.000516
PER2	113249994	4.058257955	2.020861	3.27E-06	2.27E-05
RDH14	113270195	4.053619162	2.019211	1.92E-06	1.39E-05
EGR1	113261792	4.051715409	2.018533	6.34E-06	4.18E-05
ASB8	113257679	4.049849317	2.017868	3.98E-07	3.27E-06
CUL5	113260213	4.049377129	2.0177	8.47E-08	7.92E-07
HIVEP2	113259505	4.046843153	2.016797	1.62E-08	1.72E-07
NDUFB4	113252045	4.043033156	2.015438	1.81E-06	1.32E-05
LOC113262535	113262535	4.037970685	2.01363	5.29E-06	3.55E-05
MFF	113250383	4.036782188	2.013206	1.26E-05	7.77E-05
AKAP1	113268847	4.033968049	2.0122	1.39E-06	1.03E-05
LOC113249853	113249853	4.020081521	2.007225	0.001319	0.004908
TSPAN15	113258877	4.019772276	2.007114	0.000661	0.002682

UBXN6	113242833	4.01906	2.006858	5.3E-05	0.000282
DNAJC19	113254646	4.012187675	2.004389	2.02E-06	1.45E-05
NREP	113254998	4.002028071	2.000731	9.66E-06	6.12E-05
CCDC43	113265348	3.99904936	1.999657	2.42E-07	2.07E-06
GPR21	113266387	3.998009682	1.999282	0.000501	0.002103
FBXO31	113252169	3.997940902	1.999257	2.87E-05	0.000162
TTC33	113260928	3.997247036	1.999007	9.87E-07	7.53E-06
CAMK2G	113259192	3.996334056	1.998677	3.96E-05	0.000216
DMD	113265965	3.993562762	1.997676	5.4E-05	0.000287
ZNF770	113242069	3.993032363	1.997485	3.71E-07	3.05E-06
HMGN3	113261087	3.992598334	1.997328	4.32E-07	3.53E-06
LOC113247902	113247902	3.989548809	1.996226	0.000607	0.002491
CHRNA1	113250753	3.988745087	1.995935	0.000228	0.001045
COQ7	113270738	3.988084658	1.995696	3.95E-06	2.72E-05
SLC25A20	113256849	3.987713349	1.995562	1.35E-05	8.27E-05
LZTS3	113258399	3.985565032	1.994784	8.66E-05	0.000441
IDH3B	113258411	3.982968404	1.993844	5.09E-05	0.000273
TWF2	113256944	3.975715508	1.991215	7E-05	0.000364
IGDCC4	113241013	3.97353948	1.990425	2.47E-07	2.11E-06
PDLIM7	113264157	3.970432078	1.989296	6.55E-07	5.17E-06
NDUFAF5	113258328	3.96883445	1.988715	2.75E-05	0.000156
HOXC9	113257530	3.956446757	1.984205	0.002072	0.007251
LOC113246178	113246178	3.952464885	1.982753	1.64E-07	1.46E-06
ISYNA1	113256718	3.950539913	1.98205	7.59E-05	0.000391
LOC113254727	113254727	3.950483699	1.982029	0.000105	0.000525
ATF3	113262677	3.950300408	1.981962	3.97E-05	0.000217
LOC113243815	113243815	3.946587306	1.980606	2.02E-06	1.46E-05
HADHB	113270781	3.942160329	1.978986	0.000617	0.002527

EIF4EBP1	113262099	3.941308331	1.978675	5.23E-06	3.51E-05
DEPTOR	113252772	3.939841863	1.978138	5.72E-06	3.81E-05
LOC113248580	113248580	3.930596699	1.974748	1.38E-05	8.43E-05
ZNF503	113259209	3.928341924	1.973921	5.47E-07	4.37E-06
KIF26A	113267199	3.927020843	1.973435	1.74E-05	0.000104
LOC113246863	113246863	3.926702902	1.973318	0.001765	0.006308
TRMT2B	113244297	3.924733079	1.972595	0.000265	0.001197
PFDN1	113261816	3.922935319	1.971934	3.73E-07	3.07E-06
COQ5	113267040	3.921745646	1.971496	7.52E-07	5.86E-06
TTC19	113271258	3.915333658	1.969135	3.47E-07	2.88E-06
ZSCAN9	113248163	3.911955053	1.96789	0.000313	0.001388
RASSF4	113243126	3.906260256	1.965788	4.1E-06	2.81E-05
NDUFB2	113265140	3.903614752	1.964811	7.65E-06	4.96E-05
LOC113259016	113259016	3.900719432	1.96374	6.57E-06	4.32E-05
INPP5E	113248636	3.897670056	1.962612	4.61E-05	0.000248
LOC113267907	113267907	3.896845459	1.962307	0.000295	0.001318
LOC113247923	113247923	3.894579431	1.961468	0.000349	0.001527
BLCAP	113258579	3.894471648	1.961428	0.000213	0.000988
DMAC1	113260618	3.885760421	1.958197	5E-07	4.02E-06
LOC113246288	113246288	3.879045538	1.955702	0.001244	0.004663
ADAMTS1	113269052	3.876470993	1.954744	5.07E-06	3.42E-05
NFKBIL1	113243709	3.872627078	1.953313	9.84E-07	7.52E-06
CRY2	113264999	3.867975785	1.951579	6.19E-06	4.1E-05
SOX7	113269935	3.85541899	1.946888	0.000346	0.001515
ADGRA3	113266735	3.853370354	1.946121	1.29E-06	9.64E-06
ADRA2C	113245678	3.850523892	1.945055	0.00389	0.012499
TCHH	113246136	3.849282321	1.944589	0.007877	0.022912
ACCSL	113265013	3.845553963	1.943191	0.000644	0.002623

CACNA1D	113256986	3.844295277	1.942719	0.00033	0.001453
FMO1	113262844	3.843777181	1.942525	0.002975	0.009955
LOC113261786	113261786	3.83742467	1.940138	0.000541	0.002248
UBE2G1	113271043	3.836993523	1.939976	0.000127	0.000618
SLC16A11	113271101	3.833046882	1.938492	0.000871	0.003415
BLOC1S1	113256461	3.830454079	1.937515	3.78E-05	0.000208
RHOBTB3	113255075	3.829117057	1.937012	2.74E-05	0.000156
FILIP1L	113250483	3.826354623	1.935971	5.56E-07	4.44E-06
NIM1K	113260953	3.825634634	1.935699	0.00321	0.010609
TMEM252	113270191	3.824024607	1.935092	0.003358	0.011027
MIB1	113253275	3.823556501	1.934915	1.43E-05	8.7E-05
ZNF185	113246825	3.821878687	1.934282	0.006224	0.018816
SDHD	113260049	3.818504196	1.933008	0.000135	0.000653
TRAK1	113253818	3.814270762	1.931407	2.07E-05	0.000121
CSDE1	113244393	3.812466253	1.930725	0.007437	0.021806
NUDT8	113244016	3.810721889	1.930064	0.000197	0.000917
RPP40	113264415	3.809678004	1.929669	8.55E-05	0.000436
RNF128	113241221	3.809557321	1.929623	0.010747	0.029637
TMEM185A	113241899	3.808124663	1.929081	1.26E-07	1.14E-06
NDUFV1	113244014	3.805938183	1.928252	9.2E-05	0.000464
CST6	113244082	3.805647596	1.928142	0.000113	0.000562
MRPL45	113265550	3.804602253	1.927746	1.05E-05	6.61E-05
NT5C3A	113262512	3.804600039	1.927745	9.17E-06	5.84E-05
MYOC	113262839	3.803393309	1.927287	0.016194	0.042128
LOC113248593	113248593	3.796438562	1.924647	6.74E-07	5.3E-06
DNAJB1	113247153	3.795523186	1.924299	5.77E-07	4.59E-06
LOC113260369	113260369	3.79468662	1.923981	1.12E-05	7.02E-05
ADAMTSL2	113266573	3.794410633	1.923876	0.000177	0.000838

PREPL	113246215	3.793631641	1.92358	3.1E-07	2.59E-06
ATP5MF	113267913	3.792405215	1.923113	2.22E-06	1.59E-05
TBRG4	113257719	3.784260079	1.920011	2.91E-05	0.000165
GRASP	113257465	3.782491444	1.919337	5.44E-05	0.000289
MSI2	113268852	3.77986431	1.918334	3.61E-07	2.98E-06
TMEM11	113271315	3.779381716	1.91815	1.14E-06	8.62E-06
NDUFAF2	113255305	3.775868104	1.916808	0.000761	0.003036
NDUFS6	113260793	3.774786582	1.916395	3.06E-06	2.14E-05
GOLGA4	113253773	3.770632602	1.914807	7.78E-05	0.0004
CALR3	113256650	3.770474155	1.914746	0.00055	0.00228
DEXI	113270801	3.767847945	1.913741	8.17E-07	6.34E-06
LOC113246871	113246871	3.763990755	1.912263	2.32E-06	1.65E-05
LOC113264253	113264253	3.762002117	1.911501	0.000197	0.000917
TMOD1	113260426	3.761371318	1.911259	0.00014	0.000676
VAMP2	113271170	3.761354847	1.911252	3.85E-06	2.65E-05
PPM1L	113254142	3.758561175	1.91018	2.06E-05	0.00012
NOS3	113241474	3.757553192	1.909794	1.25E-05	7.72E-05
DMAC2L	113266076	3.75632709	1.909323	7.66E-05	0.000394
REPIN1	113241464	3.753905154	1.908392	1.61E-07	1.43E-06
CELF5	113242796	3.752849238	1.907986	0.00181	0.006454
EPB41L4B	113260640	3.749679863	1.906767	0.000149	0.000715
AMACR	113260868	3.748598083	1.906351	1.66E-05	9.9E-05
CUNH3orf18	113256920	3.74835387	1.906257	0.00078	0.003101
TMEM184A	113247730	3.742373001	1.903953	0.00104	0.003981
IGFALS	113245094	3.742304539	1.903927	1.03E-05	6.52E-05
SMIM20	113266763	3.740271041	1.903143	9.98E-06	6.31E-05
SBDS	113267760	3.740005167	1.90304	5.13E-06	3.45E-05
KCNN3	113245302	3.732582874	1.900174	0.000389	0.001682

OXA1L	113246043	3.729845145	1.899116	0.000117	0.000576
LCMT1	113267594	3.72913362	1.89884	1.2E-06	8.98E-06
SLC4A3	113250414	3.728332035	1.89853	0.000489	0.002057
HEYL	113268247	3.728261085	1.898503	5.07E-07	4.07E-06
LOC113246846	113246846	3.716889029	1.894096	6.56E-06	4.32E-05
LRRC43	113266928	3.71688257	1.894093	0.001059	0.004045
TEF	113241732	3.714435706	1.893143	6.57E-06	4.32E-05
MALL	113242244	3.710609646	1.891656	0.00064	0.002609
MEIOC	113265676	3.708354466	1.890779	0.000312	0.001383
TSNARE1	113247953	3.700501604	1.887721	0.000125	0.000611
LOC113247922	113247922	3.695477365	1.885761	1.27E-05	7.8E-05
POMGNT1	113254729	3.692434443	1.884572	8.11E-06	5.22E-05
GYG1	113254042	3.691748289	1.884304	0.000867	0.003403
CUNH6orf106	113243827	3.690584246	1.883849	0.000256	0.00116
LOC113242783	113242783	3.684791335	1.881583	0.000235	0.001076
LOC113254636	113254636	3.681904493	1.880452	3.77E-06	2.6E-05
NDUFA4L2	113256390	3.677301083	1.878647	0.001745	0.006249
LOC113247877	113247877	3.676961928	1.878514	0.001742	0.006242
POLDIP2	113269025	3.676221558	1.878224	4.13E-06	2.82E-05
CDKL5	113242254	3.675688127	1.878014	0.00034	0.00149
LOC113241423	113241423	3.667294269	1.874716	0.000627	0.00256
ACTR3B	113241507	3.657492366	1.870855	1.11E-06	8.39E-06
EEF1D	113247655	3.65569526	1.870146	0.001422	0.005241
RASD2	113256004	3.653705827	1.86936	0.001031	0.003954
CATSPERD	113242861	3.65150505	1.868491	0.001273	0.004759
ATPAF1	113254713	3.650832753	1.868226	1.08E-06	8.19E-06
SPOCK2	113259155	3.650575148	1.868124	2.17E-05	0.000126
ROMO1	113258545	3.65005854	1.86792	4.67E-06	3.17E-05

ZMAT5	113245019	3.645539942	1.866133	2.27E-05	0.000131
INSYN1	113240951	3.644654032	1.865782	0.003466	0.011343
LRRTM2	113261797	3.644102694	1.865564	0.002992	0.009998
DCAF6	113262875	3.634561826	1.861781	0.000659	0.002677
CUNH9orf16	113266457	3.63428294	1.861671	3.14E-05	0.000175
ZNF391	113264258	3.632274446	1.860873	2.25E-05	0.00013
CCDC136	113263909	3.631012608	1.860372	4.25E-05	0.00023
ZYG11B	113254661	3.626846199	1.858716	4E-05	0.000218
MRPL21	113243993	3.615653444	1.854256	8.04E-06	5.18E-05
RAPGEF1	113266537	3.614830168	1.853928	1.72E-05	0.000103
FASTK	113241480	3.611712036	1.852683	7.63E-06	4.95E-05
NOX4	113269421	3.61000922	1.852003	0.002226	0.007732
LOC113247021	113247021	3.606306538	1.850522	0.001393	0.005149
YPEL2	113268888	3.605804345	1.850321	2.2E-05	0.000128
AVPI1	113251796	3.603725364	1.849489	0.000616	0.002525
PLEKHH3	113265409	3.603014788	1.849205	3.91E-06	2.69E-05
EML1	113267272	3.601332967	1.848531	0.000235	0.001077
PIH1D1	113243302	3.599848866	1.847936	2.04E-06	1.47E-05
CGNL1	113269670	3.59961167	1.847841	2.86E-05	0.000162
ANO5	113265108	3.598247884	1.847295	0.006651	0.019871
GMPS	113254098	3.593739088	1.845486	0.000134	0.000651
SLC7A6OS	113252408	3.592077977	1.844819	1.13E-05	7.03E-05
NDUFB5	113254753	5.182254423	2.37358	6.48E-09	7.35E-08
NDUFS4	113263912	5.180952033	2.373217	6.79E-08	6.49E-07
PDE4B	113254561	5.176978039	2.37211	3.17E-07	2.64E-06
ACYP2	113245706	5.16460707	2.368659	1.02E-05	6.45E-05
TAGLN	113260017	5.162970495	2.368201	1.82E-07	1.6E-06
JAG2	113267148	5.154148089	2.365734	4.1E-09	4.82E-08

MRPL33	113240736	5.14920264	2.364349	5.08E-08	4.95E-07
KIAA1217	113241331	5.142682744	2.362521	9.76E-10	1.31E-08
BZW2	113261015	5.142298357	2.362413	2.76E-07	2.33E-06
MAP3K7CL	113267437	5.125506677	2.357695	2.11E-06	1.52E-05
FAM83C	113258536	5.124313321	2.357359	1.91E-05	0.000112
ACTA2	113258969	5.116930042	2.355279	1.19E-09	1.57E-08
RNF225	113248032	5.116265534	2.355091	7.65E-05	0.000394
LOC113244568	113244568	5.109196029	2.353096	8.37E-05	0.000427
LOC113266806	113266806	5.09912202	2.350249	0.002569	0.008747
VDAC3	113245581	5.097905706	2.349905	4.07E-05	0.000221
SULF2	113258690	5.091898189	2.348204	1.15E-06	8.66E-06
LOC113254515	113254515	5.08986265	2.347627	0.000189	0.000887
MAFF	113241783	5.088669802	2.347289	3.79E-08	3.77E-07
NDUFA8	113266369	5.088070083	2.347119	1.56E-06	1.15E-05
DNASE1L1	113247843	5.041284203	2.333791	7.82E-07	6.08E-06
NHLRC1	113264504	5.032565712	2.331294	1.38E-05	8.46E-05
DKK3	113246270	5.001037803	2.322228	0.00013	0.000631
IRAK1BP1	113261094	4.993969694	2.320187	2.14E-06	1.54E-05
HOXA7	113262001	4.990864408	2.31929	5.66E-07	4.51E-06
ARPP19	113269638	4.98805239	2.318477	2.94E-06	2.06E-05
LOC113248616	113248616	4.982546716	2.316883	2.11E-08	2.18E-07
LOC113252908	113252908	4.973140037	2.314157	1.29E-05	7.93E-05
IMMP2L	113262909	4.971730147	2.313748	3.44E-07	2.85E-06
SUSD4	113262898	4.968029602	2.312674	0.000622	0.002543
PDK2	113265612	4.967713081	2.312582	3.65E-06	2.52E-05
ATP5MPL	113267204	4.958596821	2.309932	2.1E-06	1.51E-05
ACADS	113267053	4.957977519	2.309752	2.71E-06	1.91E-05
LYSMD1	113246163	4.952779437	2.308238	6.55E-08	6.27E-07

SDHB	113270447	4.950619086	2.307609	1.91E-06	1.38E-05
HIF3A	113243399	4.946625659	2.306445	0.00019	0.000888
VDAC1	113261734	4.94236777	2.305202	0.000139	0.000671
HOMER3	113256738	4.941869015	2.305057	3.26E-09	3.93E-08
IGSF23	113243462	4.918616999	2.298253	4.02E-05	0.000219
MFN2	113270495	4.918232638	2.29814	6.26E-06	4.14E-05
CUEDC1	113268859	4.908684331	2.295336	7.67E-08	7.26E-07
CYR61	113254464	4.908344555	2.295237	1.77E-08	1.86E-07
TEX29	113248531	4.904636985	2.294146	8.51E-06	5.46E-05
LOC113257882	113257882	4.90434679	2.294061	0.000108	0.000539
NDUFA9	113257995	4.898221765	2.292258	1.11E-06	8.39E-06
ATP5MC2	113257544	4.896569563	2.291771	2.28E-05	0.000132
SLC25A11	113271067	4.883670965	2.287966	1.78E-06	1.3E-05
PPP1R3F	113265953	4.880940524	2.287159	1.24E-06	9.29E-06
MAGIX	113265825	4.876353542	2.285803	2.25E-07	1.93E-06
KHDRBS3	113252694	4.873685864	2.285013	0.000129	0.000629
SYT7	113246992	4.870581233	2.284094	8.28E-07	6.41E-06
SLC25A30	113251350	4.87050487	2.284071	1.72E-08	1.81E-07
SUCLG1	113243306	4.869676356	2.283826	8.45E-05	0.000431
NEU3	113269322	4.864477853	2.282285	8.99E-09	9.98E-08
LOC113261568	113261568	4.863444805	2.281979	1.16E-08	1.26E-07
TOB1	113265636	4.861364096	2.281361	6.93E-08	6.61E-07
SVIL	113244243	4.860904544	2.281225	3.36E-05	0.000187
CIPC	113270299	4.860780052	2.281188	3.53E-09	4.21E-08
CFAP54	113256123	4.850251988	2.27806	0.000331	0.001457
TNFRSF12A	113245425	4.846130893	2.276833	0.000362	0.001577
KIAA1755	113258589	4.842617008	2.275787	8.77E-05	0.000446
ATP5F1A	113253374	4.840685563	2.275211	0.001838	0.006532

TRIM45	113244373	4.840650775	2.275201	2.45E-08	2.5E-07
LOC113257633	113257633	4.83940976	2.274831	3.44E-09	4.12E-08
TENM1	113270934	4.835496757	2.273664	0.000142	0.000685
ZSCAN12	113248152	4.815036701	2.267547	1.75E-07	1.54E-06
LOC113244843	113244843	4.813134009	2.266977	6.72E-06	4.41E-05
LOC113258460	113258460	4.808523112	2.265594	9.73E-05	0.000489
HOXD9	113250743	4.807298689	2.265226	0.0002	0.000929
NDUFB11	113265993	4.801283092	2.26342	8.75E-07	6.74E-06
LOC113249623	113249623	4.791451023	2.260463	0.012365	0.03346
ITIH3	113256968	4.786123389	2.258858	0.000112	0.000555
PPP3CB	113259177	4.78374038	2.258139	4.94E-07	3.98E-06
INHBB	113263463	4.775124396	2.255538	6.33E-05	0.000332
LOC113251521	113251521	4.766539513	2.252942	0.002711	0.009173
FMC1	113264958	4.754493474	2.249292	2.01E-09	2.54E-08
CISD1	113259063	4.752934296	2.248818	1.33E-06	9.91E-06
COMP	113256730	4.744476845	2.246249	0.009866	0.027616
CLCN4	113240744	4.73774328	2.2442	3E-07	2.51E-06
CHST10	113247050	4.734594766	2.243241	5.93E-09	6.77E-08
CCDC28B	113268366	4.726893461	2.240892	7.64E-06	4.96E-05
EFCC1	113257241	4.726197745	2.24068	3.25E-05	0.000181
TGFB2	113268733	4.711644914	2.236231	1.79E-08	1.88E-07
LOC113246281	113246281	4.706216007	2.234568	0.000409	0.001754
SLC14A2	113253366	4.698756455	2.232279	0.000488	0.002054
ABLIM1	113251961	4.697014479	2.231744	2.1E-05	0.000122
NFIC	113242797	4.687890322	2.228939	9.2E-07	7.06E-06
LOC113247619	113247619	4.685299412	2.228141	2.21E-05	0.000128
ADSL	113241749	4.682097268	2.227155	8.61E-08	8.04E-07
KCNJ12	113271319	4.675115531	2.225002	1.92E-05	0.000113

CUTC	113251818	4.665234126	2.221949	9.21E-09	1.02E-07
COL11A2	113243790	4.660151595	2.220377	3.6E-05	0.000199
FAM124B	113250394	4.659211798	2.220086	0.000186	0.000874
SGIP1	113254558	4.646785809	2.216233	0.000167	0.000793
CES1	113252254	4.64672847	2.216215	4.11E-06	2.82E-05
LOC113256842	113256842	4.63743519	2.213327	6.71E-05	0.00035
LOC113248649	113248649	4.631006825	2.211326	7.68E-09	8.58E-08
ZNF358	113242937	4.63042649	2.211145	5.52E-06	3.69E-05
LOC113242741	113242741	4.627428619	2.210211	1.55E-06	1.14E-05
MPI	113240979	4.622105896	2.20855	3.32E-09	3.99E-08
PLEKHB1	113269294	4.617135661	2.206998	2.75E-05	0.000156
TRIP10	113242888	4.611715448	2.205303	4.22E-05	0.000229
NDUFV2	113253220	4.610394365	2.20489	1.13E-06	8.51E-06
MRPS5	113242194	4.607810707	2.204081	2.51E-07	2.13E-06
ATP5F1C	113241155	4.606158927	2.203564	0.000134	0.000653
CNGB1	113252297	4.605728598	2.203429	0.000224	0.00103
KLHL21	113270566	4.601924045	2.202237	2.21E-08	2.28E-07
CASKIN1	113245186	4.59980393	2.201572	4.62E-05	0.000249
DDAH1	113254466	4.593277413	2.199524	3.94E-06	2.71E-05
CCDC141	113250711	4.581733451	2.195894	1.16E-05	7.21E-05
CUNH11orf71	113260034	4.578405716	2.194845	0.000129	0.000628
MAP7D1	113268291	4.570537539	2.192364	8.82E-06	5.64E-05
PANK4	113270600	4.566698907	2.191152	5.44E-09	6.25E-08
NR1D2	113253869	4.562547466	2.18984	2.05E-05	0.00012
RHOBTB1	113259077	4.561696656	2.189571	3.58E-07	2.96E-06
RERGL	113257835	4.557411057	2.188215	0.000253	0.001147
LOC113245319	113245319	4.557249157	2.188163	0.002256	0.007816
MYPOP	113243407	4.551043222	2.186197	1.9E-06	1.38E-05

SYN1	113265974	4.545954548	2.184583	5.48E-05	0.000291
MAPRE3	113270924	4.535290107	2.181195	5.97E-05	0.000315
ATP5PB	113244431	4.535215669	2.181171	0.000435	0.00185
MRPS24	113257987	4.530834622	2.179777	5.94E-08	5.71E-07
ATP5PD	113244651	4.530074077	2.179535	0.000312	0.001383
HSF4	113252349	4.521528408	2.176811	0.000108	0.000536
PAM16	113270852	4.517055085	2.175383	4.24E-08	4.2E-07
CLIP1	113266923	4.516352661	2.175158	7.4E-06	4.82E-05
RASSF8	113257786	4.512926943	2.174063	1.95E-07	1.7E-06
LOC113264441	113264441	4.504072863	2.17123	5.39E-05	0.000286
VSIG10L	113247243	4.500321071	2.170028	8.84E-05	0.000449
ATP5F1B	113256410	4.498354309	2.169397	0.003664	0.011886
THBS2	113247125	4.494986643	2.168317	0.006076	0.018431
SLC2A4RG	113258822	4.49131406	2.167138	7.06E-06	4.61E-05
PYURF	113263153	4.489861547	2.166671	1.31E-07	1.18E-06
NDUFB8	113251835	4.489064163	2.166415	3.95E-06	2.72E-05
IMPDH2	113256856	4.48469474	2.16501	0.000203	0.000942
SMIM26	113258309	4.478474566	2.163007	9.85E-07	7.52E-06
ZNF579	113247353	4.477947008	2.162837	2.4E-08	2.46E-07
LEAP2	113261728	4.476806722	2.16247	0.000265	0.001195
TSLP	113254959	4.475269942	2.161975	0.000423	0.001808
KREMEN1	113245007	4.462405626	2.157822	1.69E-05	0.000101
SLC6A8	113247834	4.46073902	2.157283	1.89E-07	1.65E-06
MPPED2	113265079	4.455032552	2.155436	0.00019	0.000888
NFATC1	113253544	4.454581846	2.15529	4.64E-08	4.55E-07
SERHL2	113241696	4.454385124	2.155226	6.27E-05	0.00033
KCNMB2	113254860	4.449935514	2.153784	0.000377	0.001633
SMIM12	113268319	4.448409515	2.15329	1.17E-07	1.06E-06

DPH3	113253905	4.444907507	2.152153	2.72E-07	2.3E-06
ATP8B3	113242657	4.436335544	2.149368	0.000921	0.00358
DHDH	113243321	4.436195559	2.149323	0.001094	0.004163
DNAJB6	113241530	4.434751064	2.148853	7.28E-08	6.92E-07
NDUFC2	113269364	4.428130344	2.146698	8.04E-07	6.24E-06
FMO2	113262845	4.42664973	2.146215	2.12E-06	1.52E-05
RAB12	113253223	4.425734367	2.145917	1.39E-07	1.25E-06
OPTN	113241074	4.425208502	2.145745	1.15E-05	7.17E-05
ABCA5	113265229	4.420612284	2.144246	6.71E-08	6.41E-07
HMCN1	113262754	4.416665253	2.142957	3.74E-05	0.000206
GTF3A	113251205	4.412226894	2.141507	5.24E-08	5.1E-07
JUN	113254611	4.397946738	2.13683	1.88E-06	1.36E-05
SLC16A10	113264727	4.392778297	2.135134	4.82E-06	3.27E-05
NMS	113247084	4.39199225	2.134876	0.000262	0.001184
BTC	113263271	4.376733562	2.129855	0.000453	0.001918
SLC20A2	113245534	4.376228241	2.129688	1.02E-05	6.42E-05
PIGY	113263154	4.375063498	2.129304	5.61E-07	4.48E-06
KCNJ2	113265224	4.356441672	2.12315	0.000404	0.001739
RASGRF2	113255154	4.356300926	2.123104	6.31E-07	4.99E-06
CDC34	113242694	4.355551984	2.122856	1.63E-05	9.78E-05
ZNF32	113243142	4.34553578	2.119534	1.53E-06	1.13E-05
TRMT12	113252745	4.342817029	2.118631	5.25E-07	4.21E-06
ZNF30	113244783	4.342500574	2.118526	9.2E-06	5.86E-05
EGLN1	113259033	4.342314107	2.118464	7.61E-08	7.21E-07
PARD6A	113252374	4.33358364	2.115561	0.000371	0.001613
TBC1D4	113251422	4.329015358	2.114039	1.11E-07	1.01E-06
TMEM108	113253944	4.325687774	2.11293	5.28E-05	0.000282
LACTB2	113253065	4.321230847	2.111442	1.89E-07	1.65E-06

NDUFB6	113260540	4.314698538	2.10926	1.49E-06	1.1E-05
ALKBH3	113265015	4.314252031	2.10911	1.54E-08	1.64E-07
CHRM4	113264986	4.311736002	2.108269	0.00064	0.002609
MANEAL	113268270	4.307699492	2.106918	0.000112	0.000557
MEF2D	113245229	4.307003588	2.106685	5.42E-08	5.26E-07
SFXN4	113251991	4.306582053	2.106543	1.07E-07	9.78E-07
LIMCH1	113262439	4.304482081	2.10584	1.42E-05	8.63E-05
BBS5	113250801	4.302286775	2.105104	0.000195	0.000912
ZDHHC1	113252362	4.30146684	2.104829	1.42E-05	8.63E-05
PROCA1	113269040	4.296028313	2.103004	1.44E-06	1.07E-05
MRPL12	113244509	4.295070502	2.102682	2.59E-07	2.19E-06
EVA1C	113265647	4.290495384	2.101144	8.16E-05	0.000418
LOC113241107	113241107	4.285042243	2.099309	2.61E-05	0.000149
ECPAS	113260320	4.282533375	2.098464	0.000118	0.000583
DST	113261161	4.280889485	2.097911	3.36E-05	0.000187
EPHA4	113250413	4.278272915	2.097029	0.000117	0.000579
WNT10B	113257662	4.270749728	2.094489	0.000307	0.001367
APOO	113242225	4.270280369	2.094331	1.18E-06	8.89E-06
RASSF9	113256205	4.26931494	2.094005	0.000526	0.002195
TARSL2	113263620	4.267616578	2.093431	4.15E-07	3.4E-06
NDUFA6	113241708	4.266184849	2.092946	1.75E-07	1.54E-06
ZBTB16	113260036	4.24982606	2.087404	1.04E-06	7.94E-06
WNT4	113268542	4.248253429	2.08687	0.000584	0.00241
PLEKHM2	113270664	4.245444324	2.085916	0.000313	0.001389
NR4A2	113250870	4.242651123	2.084966	0.000452	0.001916
FHL5	113264813	4.239836849	2.084009	0.000607	0.002489
PGPEP1	113256715	4.234070833	2.082045	9.51E-08	8.78E-07
PHKB	113252209	4.23405246	2.082039	1.97E-06	1.42E-05

MLLT11	113246131	4.22653532	2.079476	4.41E-08	4.36E-07
R3HDM2	113256386	4.222824325	2.078208	4.6E-05	0.000248
KPNA4	113254139	4.22064132	2.077462	1.94E-05	0.000114
GPR17	113263488	4.220098925	2.077277	0.000415	0.001778
FGF1	113255360	4.218316536	2.076667	3.56E-05	0.000197
PDE8A	113263738	4.218252776	2.076646	6.17E-07	4.89E-06
WWP1	113252973	4.217108757	2.076254	1.89E-05	0.000112
KIF13A	113264500	4.216663332	2.076102	3.55E-06	2.46E-05
UBL4A	113247812	4.216429425	2.076022	3.99E-07	3.27E-06
PRRX1	113262848	4.209858507	2.073772	3.37E-05	0.000187
KLHL24	113254479	4.208477995	2.073299	2.56E-07	2.17E-06
ZFP37	113266304	4.203456897	2.071576	7.66E-05	0.000394
MMACHC	113254743	4.201090669	2.070764	1.09E-05	6.87E-05
ITGB4	113244620	4.193272817	2.068077	8.74E-06	5.6E-05
KLHL4	113242173	4.186142972	2.065622	0.000222	0.001026
ATP5F1D	113242724	4.181534895	2.064033	0.000108	0.000536
UBR3	113250793	4.176652239	2.062347	1.19E-05	7.41E-05
SEPT11	113263245	4.174966201	2.061765	1.72E-07	1.52E-06
LOC113271232	113271232	4.167411137	2.059151	0.000237	0.001084
MUL1	113268565	4.166976009	2.059001	2.21E-07	1.9E-06
HOXC8	113257531	4.165178301	2.058378	0.014055	0.037336
CARNMT1	113269844	4.164692229	2.05821	2.07E-07	1.79E-06
LOC113255247	113255247	4.16329449	2.057726	1.84E-05	0.000109
TEX2	113265274	4.162424807	2.057424	5.9E-06	3.92E-05
AMN1	113257748	4.162101096	2.057312	7.24E-07	5.67E-06
NFE2L1	113265569	4.161381194	2.057062	4.84E-05	0.00026
LOC113267950	113267950	4.159962606	2.056571	8.16E-05	0.000418
KRTCAP3	113271234	4.159795322	2.056513	8.86E-05	0.000449

PFN2	113254055	4.157565657	2.055739	5.87E-05	0.00031
TMEM136	113259949	4.155904664	2.055163	4.45E-06	3.03E-05
RELN	113258887	4.151154847	2.053513	5.59E-05	0.000296
FBXL22	113240838	4.150241397	2.053195	0.000116	0.000573
TOMM20	113259006	4.139755496	2.049546	1.45E-05	8.81E-05
TLN2	113269702	4.138270747	2.049028	9.14E-06	5.82E-05
PNPLA4	113240758	4.136742947	2.048495	6.59E-06	4.33E-05
CLIC5	113261239	4.135311168	2.047996	1.68E-05	0.0001
RRAGB	113247969	4.13393493	2.047516	3.66E-07	3.02E-06
FAM180B	113264952	4.13102556	2.0465	0.002374	0.008163
ANKRD9	113267237	4.130931855	2.046467	3.66E-07	3.01E-06
NDUFB7	113247157	4.129473203	2.045958	1.6E-07	1.42E-06
HSCB	113245003	4.129402962	2.045933	4.48E-07	3.64E-06
PABPC4	113268249	4.127203821	2.045165	0.000167	0.000795
ATP6V1G2	113243710	4.126183016	2.044808	0.000181	0.000857
FUNDC2	113248346	4.12503991	2.044408	5.79E-07	4.6E-06
TMEM161A	113256742	4.122038757	2.043358	1.39E-07	1.25E-06
RBFOX2	113255999	4.12072284	2.042897	1.52E-05	9.17E-05
SPATA24	113261807	4.118593142	2.042152	0.000184	0.000868
UBALD1	113270843	4.111761441	2.039757	1.07E-07	9.81E-07
COL4A4	113250384	4.107943586	2.038416	0.001005	0.00387
ATP5ME	113245737	4.098735652	2.035179	4.14E-05	0.000225
AGL	113254363	4.097772001	2.03484	9.53E-05	0.00048
NDUFS2	113246718	4.095137646	2.033912	0.000278	0.001247
SMIM1	113270590	4.08809008	2.031427	1.98E-07	1.72E-06
LOC113248801	113248801	4.087076884	2.031069	0.000958	0.003708
FAM131A	113254262	4.086332039	2.030806	7.7E-07	5.99E-06
RGS5	113268681	4.07691845	2.027479	3.76E-05	0.000207

CAMSAP1	113266592	4.075745948	2.027064	8.14E-08	7.65E-07
TMCC3	113256152	4.074911414	2.026769	3.74E-05	0.000206
NDUFS1	113250544	4.066890228	2.023926	0.000103	0.000516
PER2	113249994	4.058257955	2.020861	3.27E-06	2.27E-05
RDH14	113270195	4.053619162	2.019211	1.92E-06	1.39E-05
EGR1	113261792	4.051715409	2.018533	6.34E-06	4.18E-05
ASB8	113257679	4.049849317	2.017868	3.98E-07	3.27E-06
CUL5	113260213	4.049377129	2.0177	8.47E-08	7.92E-07
HIVEP2	113259505	4.046843153	2.016797	1.62E-08	1.72E-07
NDUFB4	113252045	4.043033156	2.015438	1.81E-06	1.32E-05
LOC113262535	113262535	4.037970685	2.01363	5.29E-06	3.55E-05
MFF	113250383	4.036782188	2.013206	1.26E-05	7.77E-05
AKAP1	113268847	4.033968049	2.0122	1.39E-06	1.03E-05
LOC113249853	113249853	4.020081521	2.007225	0.001319	0.004908
TSPAN15	113258877	4.019772276	2.007114	0.000661	0.002682
UBXN6	113242833	4.01906	2.006858	5.3E-05	0.000282
DNAJC19	113254646	4.012187675	2.004389	2.02E-06	1.45E-05
NREP	113254998	4.002028071	2.000731	9.66E-06	6.12E-05
CCDC43	113265348	3.99904936	1.999657	2.42E-07	2.07E-06
GPR21	113266387	3.998009682	1.999282	0.000501	0.002103
FBXO31	113252169	3.997940902	1.999257	2.87E-05	0.000162
TTC33	113260928	3.997247036	1.999007	9.87E-07	7.53E-06
CAMK2G	113259192	3.996334056	1.998677	3.96E-05	0.000216
DMD	113265965	3.993562762	1.997676	5.4E-05	0.000287
ZNF770	113242069	3.993032363	1.997485	3.71E-07	3.05E-06
HMGN3	113261087	3.992598334	1.997328	4.32E-07	3.53E-06
LOC113247902	113247902	3.989548809	1.996226	0.000607	0.002491
CHRNA1	113250753	3.988745087	1.995935	0.000228	0.001045

COQ7	113270738	3.988084658	1.995696	3.95E-06	2.72E-05
SLC25A20	113256849	3.987713349	1.995562	1.35E-05	8.27E-05
LZTS3	113258399	3.985565032	1.994784	8.66E-05	0.000441
IDH3B	113258411	3.982968404	1.993844	5.09E-05	0.000273
TWF2	113256944	3.975715508	1.991215	7E-05	0.000364
IGDCC4	113241013	3.97353948	1.990425	2.47E-07	2.11E-06
PDLIM7	113264157	3.970432078	1.989296	6.55E-07	5.17E-06
NDUFAF5	113258328	3.96883445	1.988715	2.75E-05	0.000156
HOXC9	113257530	3.956446757	1.984205	0.002072	0.007251
LOC113246178	113246178	3.952464885	1.982753	1.64E-07	1.46E-06
ISYNA1	113256718	3.950539913	1.98205	7.59E-05	0.000391
LOC113254727	113254727	3.950483699	1.982029	0.000105	0.000525
ATF3	113262677	3.950300408	1.981962	3.97E-05	0.000217
LOC113243815	113243815	3.946587306	1.980606	2.02E-06	1.46E-05
HADHB	113270781	3.942160329	1.978986	0.000617	0.002527
EIF4EBP1	113262099	3.941308331	1.978675	5.23E-06	3.51E-05
DEPTOR	113252772	3.939841863	1.978138	5.72E-06	3.81E-05
LOC113248580	113248580	3.930596699	1.974748	1.38E-05	8.43E-05
ZNF503	113259209	3.928341924	1.973921	5.47E-07	4.37E-06
KIF26A	113267199	3.927020843	1.973435	1.74E-05	0.000104
LOC113246863	113246863	3.926702902	1.973318	0.001765	0.006308
TRMT2B	113244297	3.924733079	1.972595	0.000265	0.001197
PFDN1	113261816	3.922935319	1.971934	3.73E-07	3.07E-06
COQ5	113267040	3.921745646	1.971496	7.52E-07	5.86E-06
TTC19	113271258	3.915333658	1.969135	3.47E-07	2.88E-06
ZSCAN9	113248163	3.911955053	1.96789	0.000313	0.001388
RASSF4	113243126	3.906260256	1.965788	4.1E-06	2.81E-05
NDUFB2	113265140	3.903614752	1.964811	7.65E-06	4.96E-05

LOC113259016	113259016	3.900719432	1.96374	6.57E-06	4.32E-05
INPP5E	113248636	3.897670056	1.962612	4.61E-05	0.000248
LOC113267907	113267907	3.896845459	1.962307	0.000295	0.001318
LOC113247923	113247923	3.894579431	1.961468	0.000349	0.001527
BLCAP	113258579	3.894471648	1.961428	0.000213	0.000988
DMAC1	113260618	3.885760421	1.958197	5E-07	4.02E-06
LOC113246288	113246288	3.879045538	1.955702	0.001244	0.004663
ADAMTS1	113269052	3.876470993	1.954744	5.07E-06	3.42E-05
NFKBIL1	113243709	3.872627078	1.953313	9.84E-07	7.52E-06
CRY2	113264999	3.867975785	1.951579	6.19E-06	4.1E-05
SOX7	113269935	3.85541899	1.946888	0.000346	0.001515
ADGRA3	113266735	3.853370354	1.946121	1.29E-06	9.64E-06
ADRA2C	113245678	3.850523892	1.945055	0.00389	0.012499
TCHH	113246136	3.849282321	1.944589	0.007877	0.022912
ACCSL	113265013	3.845553963	1.943191	0.000644	0.002623
CACNA1D	113256986	3.844295277	1.942719	0.00033	0.001453
FMO1	113262844	3.843777181	1.942525	0.002975	0.009955
LOC113261786	113261786	3.83742467	1.940138	0.000541	0.002248
UBE2G1	113271043	3.836993523	1.939976	0.000127	0.000618
SLC16A11	113271101	3.833046882	1.938492	0.000871	0.003415
BLOC1S1	113256461	3.830454079	1.937515	3.78E-05	0.000208
RHOBTB3	113255075	3.829117057	1.937012	2.74E-05	0.000156
FILIP1L	113250483	3.826354623	1.935971	5.56E-07	4.44E-06
NIM1K	113260953	3.825634634	1.935699	0.00321	0.010609
TMEM252	113270191	3.824024607	1.935092	0.003358	0.011027
MIB1	113253275	3.823556501	1.934915	1.43E-05	8.7E-05
ZNF185	113246825	3.821878687	1.934282	0.006224	0.018816
SDHD	113260049	3.818504196	1.933008	0.000135	0.000653

TRAK1	113253818	3.814270762	1.931407	2.07E-05	0.000121
CSDE1	113244393	3.812466253	1.930725	0.007437	0.021806
NUDT8	113244016	3.810721889	1.930064	0.000197	0.000917
RPP40	113264415	3.809678004	1.929669	8.55E-05	0.000436
RNF128	113241221	3.809557321	1.929623	0.010747	0.029637
TMEM185A	113241899	3.808124663	1.929081	1.26E-07	1.14E-06
NDUFV1	113244014	3.805938183	1.928252	9.2E-05	0.000464
CST6	113244082	3.805647596	1.928142	0.000113	0.000562
MRPL45	113265550	3.804602253	1.927746	1.05E-05	6.61E-05
NT5C3A	113262512	3.804600039	1.927745	9.17E-06	5.84E-05
MYOC	113262839	3.803393309	1.927287	0.016194	0.042128
LOC113248593	113248593	3.796438562	1.924647	6.74E-07	5.3E-06
DNAJB1	113247153	3.795523186	1.924299	5.77E-07	4.59E-06
LOC113260369	113260369	3.79468662	1.923981	1.12E-05	7.02E-05
ADAMTSL2	113266573	3.794410633	1.923876	0.000177	0.000838
PREPL	113246215	3.793631641	1.92358	3.1E-07	2.59E-06
ATP5MF	113267913	3.792405215	1.923113	2.22E-06	1.59E-05
TBRG4	113257719	3.784260079	1.920011	2.91E-05	0.000165
GRASP	113257465	3.782491444	1.919337	5.44E-05	0.000289
MSI2	113268852	3.77986431	1.918334	3.61E-07	2.98E-06
TMEM11	113271315	3.779381716	1.91815	1.14E-06	8.62E-06
NDUFAF2	113255305	3.775868104	1.916808	0.000761	0.003036
NDUFS6	113260793	3.774786582	1.916395	3.06E-06	2.14E-05
GOLGA4	113253773	3.770632602	1.914807	7.78E-05	0.0004
CALR3	113256650	3.770474155	1.914746	0.00055	0.00228
DEXI	113270801	3.767847945	1.913741	8.17E-07	6.34E-06
LOC113246871	113246871	3.763990755	1.912263	2.32E-06	1.65E-05
LOC113264253	113264253	3.762002117	1.911501	0.000197	0.000917

TMOD1	113260426	3.761371318	1.911259	0.00014	0.000676
VAMP2	113271170	3.761354847	1.911252	3.85E-06	2.65E-05
PPM1L	113254142	3.758561175	1.91018	2.06E-05	0.00012
NOS3	113241474	3.757553192	1.909794	1.25E-05	7.72E-05
DMAC2L	113266076	3.75632709	1.909323	7.66E-05	0.000394
REPIN1	113241464	3.753905154	1.908392	1.61E-07	1.43E-06
CELF5	113242796	3.752849238	1.907986	0.00181	0.006454
EPB41L4B	113260640	3.749679863	1.906767	0.000149	0.000715
AMACR	113260868	3.748598083	1.906351	1.66E-05	9.9E-05
CUNH3orf18	113256920	3.74835387	1.906257	0.00078	0.003101
TMEM184A	113247730	3.742373001	1.903953	0.00104	0.003981
IGFALS	113245094	3.742304539	1.903927	1.03E-05	6.52E-05
SMIM20	113266763	3.740271041	1.903143	9.98E-06	6.31E-05
SBDS	113267760	3.740005167	1.90304	5.13E-06	3.45E-05
KCNN3	113245302	3.732582874	1.900174	0.000389	0.001682
OXA1L	113246043	3.729845145	1.899116	0.000117	0.000576
LCMT1	113267594	3.72913362	1.89884	1.2E-06	8.98E-06
SLC4A3	113250414	3.728332035	1.89853	0.000489	0.002057
HEYL	113268247	3.728261085	1.898503	5.07E-07	4.07E-06
LOC113246846	113246846	3.716889029	1.894096	6.56E-06	4.32E-05
LRRC43	113266928	3.71688257	1.894093	0.001059	0.004045
TEF	113241732	3.714435706	1.893143	6.57E-06	4.32E-05
MALL	113242244	3.710609646	1.891656	0.00064	0.002609
MEIOC	113265676	3.708354466	1.890779	0.000312	0.001383
TSNARE1	113247953	3.700501604	1.887721	0.000125	0.000611
LOC113247922	113247922	3.695477365	1.885761	1.27E-05	7.8E-05
POMGNT1	113254729	3.692434443	1.884572	8.11E-06	5.22E-05
GYG1	113254042	3.691748289	1.884304	0.000867	0.003403

CUNH6orf106	113243827	3.690584246	1.883849	0.000256	0.00116
LOC113242783	113242783	3.684791335	1.881583	0.000235	0.001076
LOC113254636	113254636	3.681904493	1.880452	3.77E-06	2.6E-05
NDUFA4L2	113256390	3.677301083	1.878647	0.001745	0.006249
LOC113247877	113247877	3.676961928	1.878514	0.001742	0.006242
POLDIP2	113269025	3.676221558	1.878224	4.13E-06	2.82E-05
CDKL5	113242254	3.675688127	1.878014	0.00034	0.00149
LOC113241423	113241423	3.667294269	1.874716	0.000627	0.00256
ACTR3B	113241507	3.657492366	1.870855	1.11E-06	8.39E-06
EEF1D	113247655	3.65569526	1.870146	0.001422	0.005241
RASD2	113256004	3.653705827	1.86936	0.001031	0.003954
CATSPERD	113242861	3.65150505	1.868491	0.001273	0.004759
ATPAF1	113254713	3.650832753	1.868226	1.08E-06	8.19E-06
SPOCK2	113259155	3.650575148	1.868124	2.17E-05	0.000126
ROMO1	113258545	3.65005854	1.86792	4.67E-06	3.17E-05
ZMAT5	113245019	3.645539942	1.866133	2.27E-05	0.000131
INSYN1	113240951	3.644654032	1.865782	0.003466	0.011343
LRRTM2	113261797	3.644102694	1.865564	0.002992	0.009998
DCAF6	113262875	3.634561826	1.861781	0.000659	0.002677
CUNH9orf16	113266457	3.63428294	1.861671	3.14E-05	0.000175
ZNF391	113264258	3.632274446	1.860873	2.25E-05	0.00013
CCDC136	113263909	3.631012608	1.860372	4.25E-05	0.00023
ZYG11B	113254661	3.626846199	1.858716	4E-05	0.000218
MRPL21	113243993	3.615653444	1.854256	8.04E-06	5.18E-05
RAPGEF1	113266537	3.614830168	1.853928	1.72E-05	0.000103
FASTK	113241480	3.611712036	1.852683	7.63E-06	4.95E-05
NOX4	113269421	3.61000922	1.852003	0.002226	0.007732
LOC113247021	113247021	3.606306538	1.850522	0.001393	0.005149

YPEL2	113268888	3.605804345	1.850321	2.2E-05	0.000128
AVPI1	113251796	3.603725364	1.849489	0.000616	0.002525
PLEKHH3	113265409	3.603014788	1.849205	3.91E-06	2.69E-05
EML1	113267272	3.601332967	1.848531	0.000235	0.001077
PIH1D1	113243302	3.599848866	1.847936	2.04E-06	1.47E-05
CGNL1	113269670	3.59961167	1.847841	2.86E-05	0.000162
ANO5	113265108	3.598247884	1.847295	0.006651	0.019871
GMPS	113254098	3.593739088	1.845486	0.000134	0.000651
SLC7A6OS	113252408	3.592077977	1.844819	1.13E-05	7.03E-05
CCDC43	113265348	3.99904936	1.999657	2.42E-07	2.07E-06
GPR21	113266387	3.998009682	1.999282	0.000501	0.002103
FBXO31	113252169	3.997940902	1.999257	2.87E-05	0.000162
TTC33	113260928	3.997247036	1.999007	9.87E-07	7.53E-06
CAMK2G	113259192	3.996334056	1.998677	3.96E-05	0.000216
DMD	113265965	3.993562762	1.997676	5.4E-05	0.000287
ZNF770	113242069	3.993032363	1.997485	3.71E-07	3.05E-06
HMGN3	113261087	3.992598334	1.997328	4.32E-07	3.53E-06
LOC113247902	113247902	3.989548809	1.996226	0.000607	0.002491
CHRNA1	113250753	3.988745087	1.995935	0.000228	0.001045
COQ7	113270738	3.988084658	1.995696	3.95E-06	2.72E-05
SLC25A20	113256849	3.987713349	1.995562	1.35E-05	8.27E-05
LZTS3	113258399	3.985565032	1.994784	8.66E-05	0.000441
IDH3B	113258411	3.982968404	1.993844	5.09E-05	0.000273
TWF2	113256944	3.975715508	1.991215	7E-05	0.000364
IGDCC4	113241013	3.97353948	1.990425	2.47E-07	2.11E-06
PDLIM7	113264157	3.970432078	1.989296	6.55E-07	5.17E-06
NDUFAF5	113258328	3.96883445	1.988715	2.75E-05	0.000156
HOXC9	113257530	3.956446757	1.984205	0.002072	0.007251

LOC113246178	113246178	3.952464885	1.982753	1.64E-07	1.46E-06
ISYNA1	113256718	3.950539913	1.98205	7.59E-05	0.000391
LOC113254727	113254727	3.950483699	1.982029	0.000105	0.000525
ATF3	113262677	3.950300408	1.981962	3.97E-05	0.000217
LOC113243815	113243815	3.946587306	1.980606	2.02E-06	1.46E-05
HADHB	113270781	3.942160329	1.978986	0.000617	0.002527
EIF4EBP1	113262099	3.941308331	1.978675	5.23E-06	3.51E-05
DEPTOR	113252772	3.939841863	1.978138	5.72E-06	3.81E-05
LOC113248580	113248580	3.930596699	1.974748	1.38E-05	8.43E-05
ZNF503	113259209	3.928341924	1.973921	5.47E-07	4.37E-06
KIF26A	113267199	3.927020843	1.973435	1.74E-05	0.000104
LOC113246863	113246863	3.926702902	1.973318	0.001765	0.006308
TRMT2B	113244297	3.924733079	1.972595	0.000265	0.001197
PFDN1	113261816	3.922935319	1.971934	3.73E-07	3.07E-06
COQ5	113267040	3.921745646	1.971496	7.52E-07	5.86E-06
TTC19	113271258	3.915333658	1.969135	3.47E-07	2.88E-06
ZSCAN9	113248163	3.911955053	1.96789	0.000313	0.001388
RASSF4	113243126	3.906260256	1.965788	4.1E-06	2.81E-05
NDUFB2	113265140	3.903614752	1.964811	7.65E-06	4.96E-05
LOC113259016	113259016	3.900719432	1.96374	6.57E-06	4.32E-05
INPP5E	113248636	3.897670056	1.962612	4.61E-05	0.000248
LOC113267907	113267907	3.896845459	1.962307	0.000295	0.001318
LOC113247923	113247923	3.894579431	1.961468	0.000349	0.001527
BLCAP	113258579	3.894471648	1.961428	0.000213	0.000988
DMAC1	113260618	3.885760421	1.958197	5E-07	4.02E-06
LOC113246288	113246288	3.879045538	1.955702	0.001244	0.004663
ADAMTS1	113269052	3.876470993	1.954744	5.07E-06	3.42E-05
NFKBIL1	113243709	3.872627078	1.953313	9.84E-07	7.52E-06

CRY2	113264999	3.867975785	1.951579	6.19E-06	4.1E-05
SOX7	113269935	3.85541899	1.946888	0.000346	0.001515
ADGRA3	113266735	3.853370354	1.946121	1.29E-06	9.64E-06
ADRA2C	113245678	3.850523892	1.945055	0.00389	0.012499
TCHH	113246136	3.849282321	1.944589	0.007877	0.022912
ACCSL	113265013	3.845553963	1.943191	0.000644	0.002623
CACNA1D	113256986	3.844295277	1.942719	0.00033	0.001453
FMO1	113262844	3.843777181	1.942525	0.002975	0.009955
LOC113261786	113261786	3.83742467	1.940138	0.000541	0.002248
UBE2G1	113271043	3.836993523	1.939976	0.000127	0.000618
SLC16A11	113271101	3.833046882	1.938492	0.000871	0.003415
BLOC1S1	113256461	3.830454079	1.937515	3.78E-05	0.000208
RHOBTB3	113255075	3.829117057	1.937012	2.74E-05	0.000156
FILIP1L	113250483	3.826354623	1.935971	5.56E-07	4.44E-06
NIM1K	113260953	3.825634634	1.935699	0.00321	0.010609
TMEM252	113270191	3.824024607	1.935092	0.003358	0.011027
MIB1	113253275	3.823556501	1.934915	1.43E-05	8.7E-05
ZNF185	113246825	3.821878687	1.934282	0.006224	0.018816
SDHD	113260049	3.818504196	1.933008	0.000135	0.000653
TRAK1	113253818	3.814270762	1.931407	2.07E-05	0.000121
CSDE1	113244393	3.812466253	1.930725	0.007437	0.021806
NUDT8	113244016	3.810721889	1.930064	0.000197	0.000917
RPP40	113264415	3.809678004	1.929669	8.55E-05	0.000436
RNF128	113241221	3.809557321	1.929623	0.010747	0.029637
TMEM185A	113241899	3.808124663	1.929081	1.26E-07	1.14E-06
NDUFV1	113244014	3.805938183	1.928252	9.2E-05	0.000464
CST6	113244082	3.805647596	1.928142	0.000113	0.000562
MRPL45	113265550	3.804602253	1.927746	1.05E-05	6.61E-05

NT5C3A	113262512	3.804600039	1.927745	9.17E-06	5.84E-05
MYOC	113262839	3.803393309	1.927287	0.016194	0.042128
LOC113248593	113248593	3.796438562	1.924647	6.74E-07	5.3E-06
DNAJB1	113247153	3.795523186	1.924299	5.77E-07	4.59E-06
LOC113260369	113260369	3.79468662	1.923981	1.12E-05	7.02E-05
ADAMTSL2	113266573	3.794410633	1.923876	0.000177	0.000838
PREPL	113246215	3.793631641	1.92358	3.1E-07	2.59E-06
ATP5MF	113267913	3.792405215	1.923113	2.22E-06	1.59E-05
TBRG4	113257719	3.784260079	1.920011	2.91E-05	0.000165
GRASP	113257465	3.782491444	1.919337	5.44E-05	0.000289
MSI2	113268852	3.77986431	1.918334	3.61E-07	2.98E-06
TMEM11	113271315	3.779381716	1.91815	1.14E-06	8.62E-06
NDUFAF2	113255305	3.775868104	1.916808	0.000761	0.003036
NDUFS6	113260793	3.774786582	1.916395	3.06E-06	2.14E-05
GOLGA4	113253773	3.770632602	1.914807	7.78E-05	0.0004
CALR3	113256650	3.770474155	1.914746	0.00055	0.00228
DEXI	113270801	3.767847945	1.913741	8.17E-07	6.34E-06
LOC113246871	113246871	3.763990755	1.912263	2.32E-06	1.65E-05
LOC113264253	113264253	3.762002117	1.911501	0.000197	0.000917
TMOD1	113260426	3.761371318	1.911259	0.00014	0.000676
VAMP2	113271170	3.761354847	1.911252	3.85E-06	2.65E-05
PPM1L	113254142	3.758561175	1.91018	2.06E-05	0.00012
NOS3	113241474	3.757553192	1.909794	1.25E-05	7.72E-05
DMAC2L	113266076	3.75632709	1.909323	7.66E-05	0.000394
REPIN1	113241464	3.753905154	1.908392	1.61E-07	1.43E-06
CELF5	113242796	3.752849238	1.907986	0.00181	0.006454
EPB41L4B	113260640	3.749679863	1.906767	0.000149	0.000715
AMACR	113260868	3.748598083	1.906351	1.66E-05	9.9E-05

CUNH3orf18	113256920	3.74835387	1.906257	0.00078	0.003101
TMEM184A	113247730	3.742373001	1.903953	0.00104	0.003981
IGFALS	113245094	3.742304539	1.903927	1.03E-05	6.52E-05
SMIM20	113266763	3.740271041	1.903143	9.98E-06	6.31E-05
SBDS	113267760	3.740005167	1.90304	5.13E-06	3.45E-05
KCNN3	113245302	3.732582874	1.900174	0.000389	0.001682
OXA1L	113246043	3.729845145	1.899116	0.000117	0.000576
LCMT1	113267594	3.72913362	1.89884	1.2E-06	8.98E-06
SLC4A3	113250414	3.728332035	1.89853	0.000489	0.002057
HEYL	113268247	3.728261085	1.898503	5.07E-07	4.07E-06
LOC113246846	113246846	3.716889029	1.894096	6.56E-06	4.32E-05
LRRC43	113266928	3.71688257	1.894093	0.001059	0.004045
TEF	113241732	3.714435706	1.893143	6.57E-06	4.32E-05
MALL	113242244	3.710609646	1.891656	0.00064	0.002609
MEIOC	113265676	3.708354466	1.890779	0.000312	0.001383
TSNARE1	113247953	3.700501604	1.887721	0.000125	0.000611
LOC113247922	113247922	3.695477365	1.885761	1.27E-05	7.8E-05
POMGNT1	113254729	3.692434443	1.884572	8.11E-06	5.22E-05
GYG1	113254042	3.691748289	1.884304	0.000867	0.003403
CUNH6orf106	113243827	3.690584246	1.883849	0.000256	0.00116
LOC113242783	113242783	3.684791335	1.881583	0.000235	0.001076
LOC113254636	113254636	3.681904493	1.880452	3.77E-06	2.6E-05
NDUFA4L2	113256390	3.677301083	1.878647	0.001745	0.006249
LOC113247877	113247877	3.676961928	1.878514	0.001742	0.006242
POLDIP2	113269025	3.676221558	1.878224	4.13E-06	2.82E-05
CDKL5	113242254	3.675688127	1.878014	0.00034	0.00149
LOC113241423	113241423	3.667294269	1.874716	0.000627	0.00256
ACTR3B	113241507	3.657492366	1.870855	1.11E-06	8.39E-06

EEF1D	113247655	3.65569526	1.870146	0.001422	0.005241
RASD2	113256004	3.653705827	1.86936	0.001031	0.003954
CATSPERD	113242861	3.65150505	1.868491	0.001273	0.004759
ATPAF1	113254713	3.650832753	1.868226	1.08E-06	8.19E-06
SPOCK2	113259155	3.650575148	1.868124	2.17E-05	0.000126
ROMO1	113258545	3.65005854	1.86792	4.67E-06	3.17E-05
ZMAT5	113245019	3.645539942	1.866133	2.27E-05	0.000131
INSYN1	113240951	3.644654032	1.865782	0.003466	0.011343
LRRTM2	113261797	3.644102694	1.865564	0.002992	0.009998
DCAF6	113262875	3.634561826	1.861781	0.000659	0.002677
CUNH9orf16	113266457	3.63428294	1.861671	3.14E-05	0.000175
ZNF391	113264258	3.632274446	1.860873	2.25E-05	0.00013
CCDC136	113263909	3.631012608	1.860372	4.25E-05	0.00023
ZYG11B	113254661	3.626846199	1.858716	4E-05	0.000218
MRPL21	113243993	3.615653444	1.854256	8.04E-06	5.18E-05
RAPGEF1	113266537	3.614830168	1.853928	1.72E-05	0.000103
FASTK	113241480	3.611712036	1.852683	7.63E-06	4.95E-05
NOX4	113269421	3.61000922	1.852003	0.002226	0.007732
LOC113247021	113247021	3.606306538	1.850522	0.001393	0.005149
YPEL2	113268888	3.605804345	1.850321	2.2E-05	0.000128
AVPI1	113251796	3.603725364	1.849489	0.000616	0.002525
PLEKHH3	113265409	3.603014788	1.849205	3.91E-06	2.69E-05
EML1	113267272	3.601332967	1.848531	0.000235	0.001077
PIH1D1	113243302	3.599848866	1.847936	2.04E-06	1.47E-05
CGNL1	113269670	3.59961167	1.847841	2.86E-05	0.000162
ANO5	113265108	3.598247884	1.847295	0.006651	0.019871
GMPS	113254098	3.593739088	1.845486	0.000134	0.000651
SLC7A6OS	113252408	3.592077977	1.844819	1.13E-05	7.03E-05

CCDC43	113265348	3.99904936	1.999657	2.42E-07	2.07E-06
GPR21	113266387	3.998009682	1.999282	0.000501	0.002103
FBXO31	113252169	3.997940902	1.999257	2.87E-05	0.000162
TTC33	113260928	3.997247036	1.999007	9.87E-07	7.53E-06
CAMK2G	113259192	3.996334056	1.998677	3.96E-05	0.000216
DMD	113265965	3.993562762	1.997676	5.4E-05	0.000287
ZNF770	113242069	3.993032363	1.997485	3.71E-07	3.05E-06
HMGN3	113261087	3.992598334	1.997328	4.32E-07	3.53E-06
LOC113247902	113247902	3.989548809	1.996226	0.000607	0.002491
CHRNA1	113250753	3.988745087	1.995935	0.000228	0.001045
COQ7	113270738	3.988084658	1.995696	3.95E-06	2.72E-05
SLC25A20	113256849	3.987713349	1.995562	1.35E-05	8.27E-05
LZTS3	113258399	3.985565032	1.994784	8.66E-05	0.000441
IDH3B	113258411	3.982968404	1.993844	5.09E-05	0.000273
TWF2	113256944	3.975715508	1.991215	7E-05	0.000364
IGDCC4	113241013	3.97353948	1.990425	2.47E-07	2.11E-06
PDLIM7	113264157	3.970432078	1.989296	6.55E-07	5.17E-06
NDUFAF5	113258328	3.96883445	1.988715	2.75E-05	0.000156
HOXC9	113257530	3.956446757	1.984205	0.002072	0.007251
LOC113246178	113246178	3.952464885	1.982753	1.64E-07	1.46E-06
ISYNA1	113256718	3.950539913	1.98205	7.59E-05	0.000391
LOC113254727	113254727	3.950483699	1.982029	0.000105	0.000525
ATF3	113262677	3.950300408	1.981962	3.97E-05	0.000217
LOC113243815	113243815	3.946587306	1.980606	2.02E-06	1.46E-05
HADHB	113270781	3.942160329	1.978986	0.000617	0.002527
EIF4EBP1	113262099	3.941308331	1.978675	5.23E-06	3.51E-05
DEPTOR	113252772	3.939841863	1.978138	5.72E-06	3.81E-05
LOC113248580	113248580	3.930596699	1.974748	1.38E-05	8.43E-05

ZNF503	113259209	3.928341924	1.973921	5.47E-07	4.37E-06
KIF26A	113267199	3.927020843	1.973435	1.74E-05	0.000104
LOC113246863	113246863	3.926702902	1.973318	0.001765	0.006308
TRMT2B	113244297	3.924733079	1.972595	0.000265	0.001197
PFDN1	113261816	3.922935319	1.971934	3.73E-07	3.07E-06
COQ5	113267040	3.921745646	1.971496	7.52E-07	5.86E-06
TTC19	113271258	3.915333658	1.969135	3.47E-07	2.88E-06
ZSCAN9	113248163	3.911955053	1.96789	0.000313	0.001388
RASSF4	113243126	3.906260256	1.965788	4.1E-06	2.81E-05
NDUFB2	113265140	3.903614752	1.964811	7.65E-06	4.96E-05
LOC113259016	113259016	3.900719432	1.96374	6.57E-06	4.32E-05
INPP5E	113248636	3.897670056	1.962612	4.61E-05	0.000248
LOC113267907	113267907	3.896845459	1.962307	0.000295	0.001318
LOC113247923	113247923	3.894579431	1.961468	0.000349	0.001527
BLCAP	113258579	3.894471648	1.961428	0.000213	0.000988
DMAC1	113260618	3.885760421	1.958197	5E-07	4.02E-06
LOC113246288	113246288	3.879045538	1.955702	0.001244	0.004663
ADAMTS1	113269052	3.876470993	1.954744	5.07E-06	3.42E-05
NFKBIL1	113243709	3.872627078	1.953313	9.84E-07	7.52E-06
CRY2	113264999	3.867975785	1.951579	6.19E-06	4.1E-05
SOX7	113269935	3.85541899	1.946888	0.000346	0.001515
ADGRA3	113266735	3.853370354	1.946121	1.29E-06	9.64E-06
ADRA2C	113245678	3.850523892	1.945055	0.00389	0.012499
TCHH	113246136	3.849282321	1.944589	0.007877	0.022912
ACCSL	113265013	3.845553963	1.943191	0.000644	0.002623
CACNA1D	113256986	3.844295277	1.942719	0.00033	0.001453
FMO1	113262844	3.843777181	1.942525	0.002975	0.009955
LOC113261786	113261786	3.83742467	1.940138	0.000541	0.002248

UBE2G1	113271043	3.836993523	1.939976	0.000127	0.000618
SLC16A11	113271101	3.833046882	1.938492	0.000871	0.003415
BLOC1S1	113256461	3.830454079	1.937515	3.78E-05	0.000208
RHOBTB3	113255075	3.829117057	1.937012	2.74E-05	0.000156
FILIP1L	113250483	3.826354623	1.935971	5.56E-07	4.44E-06
NIM1K	113260953	3.825634634	1.935699	0.00321	0.010609
TMEM252	113270191	3.824024607	1.935092	0.003358	0.011027
MIB1	113253275	3.823556501	1.934915	1.43E-05	8.7E-05
ZNF185	113246825	3.821878687	1.934282	0.006224	0.018816
SDHD	113260049	3.818504196	1.933008	0.000135	0.000653
TRAK1	113253818	3.814270762	1.931407	2.07E-05	0.000121
CSDE1	113244393	3.812466253	1.930725	0.007437	0.021806
NUDT8	113244016	3.810721889	1.930064	0.000197	0.000917
RPP40	113264415	3.809678004	1.929669	8.55E-05	0.000436
RNF128	113241221	3.809557321	1.929623	0.010747	0.029637
TMEM185A	113241899	3.808124663	1.929081	1.26E-07	1.14E-06
NDUFV1	113244014	3.805938183	1.928252	9.2E-05	0.000464
CST6	113244082	3.805647596	1.928142	0.000113	0.000562
MRPL45	113265550	3.804602253	1.927746	1.05E-05	6.61E-05
NT5C3A	113262512	3.804600039	1.927745	9.17E-06	5.84E-05
MYOC	113262839	3.803393309	1.927287	0.016194	0.042128
LOC113248593	113248593	3.796438562	1.924647	6.74E-07	5.3E-06
DNAJB1	113247153	3.795523186	1.924299	5.77E-07	4.59E-06
LOC113260369	113260369	3.79468662	1.923981	1.12E-05	7.02E-05
ADAMTSL2	113266573	3.794410633	1.923876	0.000177	0.000838
PREPL	113246215	3.793631641	1.92358	3.1E-07	2.59E-06
ATP5MF	113267913	3.792405215	1.923113	2.22E-06	1.59E-05
TBRG4	113257719	3.784260079	1.920011	2.91E-05	0.000165

GRASP	113257465	3.782491444	1.919337	5.44E-05	0.000289
MSI2	113268852	3.77986431	1.918334	3.61E-07	2.98E-06
TMEM11	113271315	3.779381716	1.91815	1.14E-06	8.62E-06
NDUFAF2	113255305	3.775868104	1.916808	0.000761	0.003036
NDUFS6	113260793	3.774786582	1.916395	3.06E-06	2.14E-05
GOLGA4	113253773	3.770632602	1.914807	7.78E-05	0.0004
CALR3	113256650	3.770474155	1.914746	0.00055	0.00228
DEXI	113270801	3.767847945	1.913741	8.17E-07	6.34E-06
LOC113246871	113246871	3.763990755	1.912263	2.32E-06	1.65E-05
LOC113264253	113264253	3.762002117	1.911501	0.000197	0.000917
TMOD1	113260426	3.761371318	1.911259	0.00014	0.000676
VAMP2	113271170	3.761354847	1.911252	3.85E-06	2.65E-05
PPM1L	113254142	3.758561175	1.91018	2.06E-05	0.00012
NOS3	113241474	3.757553192	1.909794	1.25E-05	7.72E-05
DMAC2L	113266076	3.75632709	1.909323	7.66E-05	0.000394
REPIN1	113241464	3.753905154	1.908392	1.61E-07	1.43E-06
CELF5	113242796	3.752849238	1.907986	0.00181	0.006454
EPB41L4B	113260640	3.749679863	1.906767	0.000149	0.000715
AMACR	113260868	3.748598083	1.906351	1.66E-05	9.9E-05
CUNH3orf18	113256920	3.74835387	1.906257	0.00078	0.003101
TMEM184A	113247730	3.742373001	1.903953	0.00104	0.003981
IGFALS	113245094	3.742304539	1.903927	1.03E-05	6.52E-05
SMIM20	113266763	3.740271041	1.903143	9.98E-06	6.31E-05
SBDS	113267760	3.740005167	1.90304	5.13E-06	3.45E-05
KCNN3	113245302	3.732582874	1.900174	0.000389	0.001682
OXA1L	113246043	3.729845145	1.899116	0.000117	0.000576
LCMT1	113267594	3.72913362	1.89884	1.2E-06	8.98E-06
SLC4A3	113250414	3.728332035	1.89853	0.000489	0.002057

HEYL	113268247	3.728261085	1.898503	5.07E-07	4.07E-06
LOC113246846	113246846	3.716889029	1.894096	6.56E-06	4.32E-05
LRRC43	113266928	3.71688257	1.894093	0.001059	0.004045
TEF	113241732	3.714435706	1.893143	6.57E-06	4.32E-05
MALL	113242244	3.710609646	1.891656	0.00064	0.002609
MEIOC	113265676	3.708354466	1.890779	0.000312	0.001383
TSNARE1	113247953	3.700501604	1.887721	0.000125	0.000611
LOC113247922	113247922	3.695477365	1.885761	1.27E-05	7.8E-05
POMGNT1	113254729	3.692434443	1.884572	8.11E-06	5.22E-05
GYG1	113254042	3.691748289	1.884304	0.000867	0.003403
CUNH6orf106	113243827	3.690584246	1.883849	0.000256	0.00116
LOC113242783	113242783	3.684791335	1.881583	0.000235	0.001076
LOC113254636	113254636	3.681904493	1.880452	3.77E-06	2.6E-05
NDUFA4L2	113256390	3.677301083	1.878647	0.001745	0.006249
LOC113247877	113247877	3.676961928	1.878514	0.001742	0.006242
POLDIP2	113269025	3.676221558	1.878224	4.13E-06	2.82E-05
CDKL5	113242254	3.675688127	1.878014	0.00034	0.00149
LOC113241423	113241423	3.667294269	1.874716	0.000627	0.00256
ACTR3B	113241507	3.657492366	1.870855	1.11E-06	8.39E-06
EEF1D	113247655	3.65569526	1.870146	0.001422	0.005241
RASD2	113256004	3.653705827	1.86936	0.001031	0.003954
CATSPERD	113242861	3.65150505	1.868491	0.001273	0.004759
ATPAF1	113254713	3.650832753	1.868226	1.08E-06	8.19E-06
SPOCK2	113259155	3.650575148	1.868124	2.17E-05	0.000126
ROMO1	113258545	3.65005854	1.86792	4.67E-06	3.17E-05
ZMAT5	113245019	3.645539942	1.866133	2.27E-05	0.000131
INSYN1	113240951	3.644654032	1.865782	0.003466	0.011343
LRRTM2	113261797	3.644102694	1.865564	0.002992	0.009998

DCAF6	113262875	3.634561826	1.861781	0.000659	0.002677
CUNH9orf16	113266457	3.63428294	1.861671	3.14E-05	0.000175
ZNF391	113264258	3.632274446	1.860873	2.25E-05	0.00013
CCDC136	113263909	3.631012608	1.860372	4.25E-05	0.00023
ZYG11B	113254661	3.626846199	1.858716	4E-05	0.000218
MRPL21	113243993	3.615653444	1.854256	8.04E-06	5.18E-05
RAPGEF1	113266537	3.614830168	1.853928	1.72E-05	0.000103
FASTK	113241480	3.611712036	1.852683	7.63E-06	4.95E-05
NOX4	113269421	3.61000922	1.852003	0.002226	0.007732
LOC113247021	113247021	3.606306538	1.850522	0.001393	0.005149
YPEL2	113268888	3.605804345	1.850321	2.2E-05	0.000128
AVPI1	113251796	3.603725364	1.849489	0.000616	0.002525
PLEKHH3	113265409	3.603014788	1.849205	3.91E-06	2.69E-05
EML1	113267272	3.601332967	1.848531	0.000235	0.001077
PIH1D1	113243302	3.599848866	1.847936	2.04E-06	1.47E-05
CGNL1	113269670	3.59961167	1.847841	2.86E-05	0.000162
ANO5	113265108	3.598247884	1.847295	0.006651	0.019871
GMPS	113254098	3.593739088	1.845486	0.000134	0.000651
SLC7A6OS	113252408	3.592077977	1.844819	1.13E-05	7.03E-05
NEDD4L	113253456	3.590042364	1.844001	4.92E-06	3.32E-05
GAL3ST2	113249264	3.588002696	1.843181	0.000785	0.003116
F3	113254382	3.58712502	1.842828	0.001606	0.005826
TMEM134	113244024	3.586823694	1.842707	2.17E-06	1.56E-05
APPL1	113257000	3.586415354	1.842543	3.51E-06	2.43E-05
CRYBG3	113250328	3.58226459	1.840872	0.000178	0.000843
MEF2A	113263641	3.579281776	1.83967	6.59E-07	5.2E-06
ADCY10	113262877	3.579032156	1.83957	0.003191	0.010557
PAIP2B	113244155	3.576908199	1.838713	2.02E-05	0.000118

ATP5MC3	113250747	3.575720243	1.838234	0.001031	0.003954
CEP85L	113259392	3.574804437	1.837864	5.18E-06	3.49E-05
TMEM159	113267545	3.573363401	1.837283	1.3E-06	9.7E-06
ZNF524	113247354	3.56960476	1.835764	9.97E-06	6.3E-05
TUFM	113267631	3.569499479	1.835722	0.000335	0.001469
DLD	113258483	3.566928697	1.834682	0.001165	0.004399
CRIM1	113241217	3.564725219	1.833791	9.95E-05	0.000499
SDHA	113260769	3.551950163	1.828611	0.000354	0.001546
CLEC14A	113266145	3.551781527	1.828543	4.67E-06	3.17E-05
SDR39U1	113246062	3.549307038	1.827537	9.03E-05	0.000457
TMEM30B	113242411	3.54742764	1.826773	0.002505	0.008557
LOC113258537	113258537	3.546022548	1.826202	1.72E-05	0.000102
LOC113253205	113253205	3.545114425	1.825832	2.89E-05	0.000164
PCBD2	113261752	3.544442697	1.825559	4.42E-06	3.01E-05
ADAMTSL3	113263758	3.544401778	1.825542	0.000146	0.000702
PLCB4	113258346	3.539653277	1.823608	0.000218	0.001005
CATSPER3	113261753	3.536271087	1.822229	0.001098	0.004174
ZXDC	113257283	3.532674258	1.820761	2.51E-06	1.78E-05
VEGFB	113244181	3.528584524	1.81909	0.001352	0.005014
TIGD3	113243923	3.526810685	1.818364	0.001581	0.005754
APOPT1	113267216	3.524507604	1.817422	1.52E-05	9.17E-05
LOC113265291	113265291	3.520374274	1.815729	8.54E-06	5.48E-05
PIK3IP1	113245057	3.518942195	1.815142	0.000187	0.000879
PPFIBP1	113257771	3.510416902	1.811642	3.28E-06	2.28E-05
PDHX	113265044	3.507148103	1.810298	0.000126	0.000617
LOC113248583	113248583	3.503187132	1.808668	0.000305	0.001356
RPS29	113266188	3.499883319	1.807307	0.00435	0.013767
COQ6	113270341	3.49963143	1.807203	3E-06	2.1E-05

YBX2	113271116	3.499617162	1.807197	0.000601	0.002467
MAVS	113258384	3.4958603	1.805648	1.64E-06	1.2E-05
ACKR3	113250308	3.488821828	1.80274	0.003605	0.011736
EIF3G	113242974	3.488490533	1.802603	0.000421	0.0018
ENHO	113260508	3.488172505	1.802471	0.000769	0.003064
SPINK2	113262527	3.483467	1.800524	0.000823	0.003254
MRPS25	113257208	3.480569347	1.799323	4.47E-06	3.04E-05
SLC4A4	113263295	3.480401025	1.799254	0.007118	0.021017
LOC113247912	113247912	3.47871395	1.798554	0.002339	0.008064
ATXN7L2	113244924	3.475356065	1.797161	0.000112	0.000557
POU6F1	113257612	3.472595388	1.796014	6.11E-05	0.000322
AMOTL1	113259818	3.468205742	1.794189	4.56E-05	0.000246
PRKAR2A	113256848	3.467441067	1.793871	0.000783	0.003113
LOC113254662	113254662	3.465723371	1.793157	0.000167	0.000793
ZNF594	113271063	3.463772534	1.792344	0.001363	0.00505
ID1	113258459	3.461662916	1.791465	1.94E-05	0.000114
DIP2C	113241102	3.459304406	1.790482	1.98E-06	1.43E-05
CREM	113244224	3.458214465	1.790027	1.19E-05	7.41E-05
MAP4	113256822	3.457188853	1.789599	0.002493	0.008523
CUNH14orf132	113267299	3.455865418	1.789047	0.012174	0.033026
RIC8B	113256034	3.454698727	1.78856	4.38E-06	2.99E-05
CDH6	113260848	3.452467856	1.787628	0.000698	0.002819
LRRC66	113262481	3.449724097	1.786481	0.000739	0.002959
VMA21	113246824	3.444533538	1.784309	2.16E-05	0.000126
NDUFC1	113255686	3.442579605	1.78349	1.68E-05	0.0001
SGCB	113262483	3.442086369	1.783283	0.000701	0.002825
LOC113260556	113260556	3.440184957	1.782486	0.002187	0.007616
ACAA2	113253408	3.439193221	1.78207	0.000414	0.001775

OLFML2A	113266401	3.434517205	1.780107	0.000115	0.000569
HINT3	113259418	3.434023859	1.7799	0.000189	0.000886
AMIGO2	113257695	3.43372626	1.779775	0.01055	0.029196
PDCD2L	113244745	3.432722361	1.779353	0.000268	0.001207
TMEM255A	113270909	3.426475939	1.776726	0.003208	0.010606
CPEB3	113243165	3.425567628	1.776343	3.1E-05	0.000173
LOC113265073	113265073	3.418345673	1.773298	0.007151	0.021095
NDUFB1	113267167	3.415993453	1.772305	5.17E-06	3.48E-05
UBE3D	113261067	3.415799139	1.772223	2.93E-05	0.000165
NDUFS5	113268254	3.411185816	1.770273	6.72E-05	0.00035
LRRC28	113263643	3.410676985	1.770058	9.2E-05	0.000464
ASB9	113240712	3.409259744	1.769459	0.00058	0.002394
LAMA3	113253294	3.407777798	1.768831	2.38E-05	0.000137
NR3C2	113255738	3.406989183	1.768497	4.56E-05	0.000246
RAMP3	113257714	3.406345448	1.768225	0.000741	0.002967
LYSMD2	113269627	3.404966137	1.76764	9.9E-05	0.000497
RAB9B	113248103	3.395980463	1.763828	0.002074	0.007258
MICU3	113262162	3.391900113	1.762094	7.07E-05	0.000367
IGFBP3	113257692	3.391405912	1.761883	0.000638	0.002602
PAQR6	113245241	3.388994821	1.760857	0.001753	0.006275
HOXD8	113250741	3.38647232	1.759783	0.000437	0.00186
ATP5F1E	113258773	3.383238802	1.758405	0.001436	0.005284
PPT2	113243752	3.382448144	1.758068	3.13E-05	0.000175
CRYGN	113241492	3.381813683	1.757797	0.002068	0.00724
LOC113249678	113249678	3.381784643	1.757785	0.00072	0.002894
GKAP1	113269812	3.381160965	1.757519	2.87E-05	0.000163
F8	113248344	3.378847678	1.756531	8.01E-06	5.17E-05
AKTIP	113252238	3.3778185	1.756092	5.18E-06	3.48E-05

RPL36	113242860	3.377394781	1.755911	0.001506	0.005519
PPIF	113259219	3.377145178	1.755804	5.11E-05	0.000274
PAX1	113246770	3.375600611	1.755144	0.000765	0.003053
NICN1	113256875	3.374928071	1.754857	5.15E-05	0.000276
DOCK9	113251489	3.372852403	1.753969	0.000307	0.001367
KLHL3	113261768	3.372512597	1.753824	0.000216	0.001
EIF1	113265448	3.369293016	1.752446	0.002273	0.00787
ETFA	113248087	3.366827865	1.75139	0.0006	0.002463
PPP1R2	113253297	3.365070153	1.750637	9.29E-05	0.000469
MYLK3	113252202	3.364743685	1.750497	0.000791	0.003136
CA8	113268071	3.362669725	1.749607	0.005569	0.017079
TIMM10	113246386	3.360979145	1.748882	1.46E-05	8.86E-05
DNAJB4	113254500	3.360159745	1.74853	3.75E-05	0.000206
HSF2BP	113253534	3.359879498	1.748409	0.003219	0.010634
KANK3	113242903	3.35878905	1.747941	1.97E-05	0.000115
EGFL7	113248635	3.358451504	1.747796	0.000227	0.001044
LOC113249852	113249852	3.345980624	1.742429	0.004494	0.014173
LOC113251944	113251944	3.344200335	1.741661	2.83E-05	0.000161
RPS11	113243297	3.342725509	1.741025	0.015891	0.041499
NFU1	113244593	3.342541885	1.740946	0.000218	0.001006
MTHFD2L	113263273	3.342135295	1.74077	0.001544	0.005636
PLCH2	113270598	3.340930487	1.74025	0.004913	0.015351
LOC113266881	113266881	3.340637861	1.740124	0.003867	0.012438
DLL4	113242017	3.337376829	1.738715	9.73E-05	0.000489
IL20RB	113253976	3.33627415	1.738238	0.001155	0.00437
SEZ6L2	113267659	3.333547304	1.737058	0.00139	0.00514
SLC35E4	113245043	3.333370453	1.736982	0.001078	0.004107
STAC2	113265524	3.331141012	1.736016	0.003093	0.010266

KIF5A	113256376	3.330410028	1.7357	0.002441	0.008371
KLC2	113244076	3.329548735	1.735327	2.99E-05	0.000168
GPR176	113242047	3.329280627	1.73521	0.003092	0.010262
EEPD1	113262749	3.3271882	1.734303	1.7E-05	0.000101
FAN1	113263610	3.326754004	1.734115	3.25E-06	2.26E-05
METTL25	113256211	3.324669791	1.733211	1.31E-05	8.04E-05
HOXA4	113261989	3.321005488	1.73162	0.002582	0.008785
TRNP1	113268445	3.319231645	1.730849	0.00246	0.008424
KIRREL2	113244768	3.317684807	1.730177	0.004848	0.015177
OTUD6B	113252945	3.314109655	1.728621	7E-06	4.58E-05
LOC113245484	113245484	3.313583272	1.728392	0.002849	0.009569
HSD11B1	113262645	3.312689487	1.728003	0.004546	0.014315
AIMP2	113267935	3.31022476	1.726929	5.39E-06	3.61E-05
ZSCAN23	113248160	3.309281715	1.726518	0.000293	0.001312
CBX7	113241765	3.308187355	1.726041	0.000142	0.000685
SORBS3	113270000	3.304664506	1.724504	1.94E-05	0.000114
NDUFA2	113261833	3.304150656	1.724279	4.75E-05	0.000255
PPIP5K1	113241961	3.303392757	1.723949	7.88E-06	5.1E-05
LOC113251647	113251647	3.300370629	1.722628	6.58E-05	0.000343
LOC113249996	113249996	3.299999582	1.722466	0.000824	0.003256
ZNF768	113267684	3.296789498	1.721062	2.54E-05	0.000145
LRRFIP2	113253771	3.291459626	1.718728	8.89E-06	5.68E-05
SMC1B	113241654	3.289621151	1.717921	0.003595	0.011709
CD101	113244376	3.288270827	1.717329	0.008184	0.023616
CNTNAP2	113241449	3.287882427	1.717159	0.007861	0.022875
DOCK6	113243014	3.287431334	1.716961	1.3E-05	7.99E-05
STAU2	113253047	3.287052728	1.716795	8.96E-06	5.72E-05
NCBP2-AS2	113253142	3.285664605	1.716185	3.53E-05	0.000195

FEZ1	113259906	3.28492096	1.715859	0.00271	0.009173
RTKN	113243549	3.282075499	1.714608	2.72E-05	0.000155
HSPB1	113267813	3.278535964	1.713052	0.000425	0.001817
IL33	113260625	3.276372815	1.7121	0.002602	0.008838
TMEM150A	113243094	3.27145468	1.709932	2.66E-05	0.000152
LOC113247253	113247253	3.271232642	1.709834	1.3E-05	7.98E-05
PERP	113259487	3.270614368	1.709562	0.002334	0.008052
TMEM63C	113270297	3.266683308	1.707827	0.004453	0.014053
ANHX	113266821	3.264827791	1.707007	0.004274	0.013565
ARHGEF10L	113270439	3.264590935	1.706902	7.82E-06	5.07E-05
BNIP3	113252072	3.264225442	1.706741	0.002829	0.009515
LOC113241880	113241880	3.262983836	1.706192	0.008391	0.024114
TFB2M	113262945	3.261730541	1.705638	6.36E-05	0.000334
ECSIT	113243030	3.261076682	1.705348	1.06E-05	6.69E-05
CTF1	113267705	3.260390783	1.705045	0.003193	0.01056
CPN1	113251825	3.259239549	1.704535	0.001032	0.003957
TBX3	113267002	3.258324578	1.70413	0.00092	0.003577
TOMM40L	113246746	3.257914495	1.703949	5.92E-05	0.000312
ALKBH5	113271291	3.255584654	1.702917	6.56E-05	0.000343
BHLHB9	113244314	3.255550596	1.702902	2.94E-05	0.000166
LOC113242947	113242947	3.254080071	1.70225	0.000158	0.000755
LOC113247867	113247867	3.252606655	1.701596	0.000699	0.002822
CUNH6orf203	113264776	3.252515718	1.701556	3.9E-05	0.000213
CACNG7	113247410	3.25223201	1.70143	0.006226	0.018821
B3GALNT2	113259001	3.250909546	1.700843	7.3E-05	0.000378
HIGD2A	113264125	3.247908071	1.699511	6.3E-05	0.000331
MRPL24	113245221	3.247124686	1.699163	2.84E-05	0.000161
MAP3K10	113243573	3.247044922	1.699127	0.00012	0.000589

GRTP1	113247574	3.244403028	1.697953	0.005745	0.017561
GBX1	113241483	3.244314094	1.697913	0.003987	0.012774
FBXL8	113252354	3.243087029	1.697368	0.002653	0.008994
WSCD1	113271054	3.241164239	1.696512	1.95E-05	0.000114
TMEM51	113270481	3.240518983	1.696225	0.00107	0.004082
MYO18A	113269060	3.238414511	1.695288	0.000779	0.003097
PADI2	113270446	3.236029531	1.694225	0.009881	0.02763
GPR75	113245790	3.235099395	1.69381	0.003227	0.010653
CCDC85A	113245393	3.232152111	1.692495	0.003833	0.01236
ZBTB47	113253822	3.2260961	1.689789	0.000535	0.002222
MMP24	113258534	3.225922897	1.689712	0.001628	0.005891
TRHDE	113256259	3.225791202	1.689653	0.005813	0.017745
KCNQ5	113261119	3.22546534	1.689507	0.008045	0.023317
RPL35A	113252711	3.223755385	1.688742	0.012074	0.032784
NRN1	113264419	3.222334922	1.688106	0.005384	0.016621
NDUFB3	113250598	3.21772801	1.686042	6.63E-05	0.000346
MYORG	113260509	3.215011339	1.684824	0.008114	0.023467
PSTPIP2	113253373	3.212991557	1.683917	0.000226	0.001039
LOC113254434	113254434	3.209359011	1.682285	9.07E-05	0.000459
CIART	113246110	3.208303655	1.681811	0.006768	0.020136
MRPS2	113266589	3.20653382	1.681015	0.000104	0.00052
THSD1	113251287	3.205870368	1.680716	0.003461	0.011332
STAC	113253765	3.205029479	1.680338	0.013498	0.036046
SLC25A42	113256741	3.203746704	1.67976	0.002398	0.008235
SQSTM1	113261555	3.20347816	1.679639	0.002571	0.008752
TCTA	113256877	3.203353044	1.679583	1.4E-05	8.55E-05
RITA1	113266990	3.202574242	1.679232	1.28E-05	7.86E-05
CDC37L1	113270165	3.198859087	1.677557	2.56E-06	1.82E-05

ISLR	113240961	3.197064578	1.676748	0.000753	0.00301
TSPAN3	113241005	3.193557791	1.675165	0.000507	0.002125
HES4	113270655	3.192903465	1.674869	4.17E-05	0.000226
L2HGDH	113266207	3.192701414	1.674778	4.56E-05	0.000246
SH3GL3	113263757	3.191801043	1.674371	0.001704	0.006125
CUNH16orf95	113252171	3.187520806	1.672435	0.010321	0.028684
NOV	113252778	3.18730676	1.672338	0.005888	0.017944
LOC113271076	113271076	3.183638048	1.670676	0.006671	0.019916
RTN4	113245629	3.183131295	1.670447	0.01305	0.035064
GPIHBP1	113247595	3.18062491	1.66931	0.0023	0.007948
ITGA3	113265609	3.18030223	1.669164	0.000486	0.002047
LOC113270384	113270384	3.180292033	1.669159	0.000119	0.000587
WSB2	113267016	3.180198765	1.669117	8.34E-05	0.000426
RUNDC3B	113259507	3.179494246	1.668797	4.68E-05	0.000252
DLGAP1	113253243	3.179394387	1.668752	0.002276	0.007879
LOC113247252	113247252	3.175718691	1.667083	0.003653	0.011857
AGMO	113260890	3.174381325	1.666475	0.006392	0.019223
TCEAL1	113249836	3.173393193	1.666026	4.06E-05	0.000221
LOC113246566	113246566	3.17200725	1.665396	5.81E-05	0.000307
LOC113249742	113249742	3.170074408	1.664517	3.01E-05	0.000169
CMC4	113248350	3.169996927	1.664481	0.000369	0.001602
SPARCL1	113263168	3.168479972	1.663791	0.00688	0.020393
CUNH8orf59	113252990	3.167856619	1.663507	2.91E-05	0.000164
HOXB8	113265579	3.166925327	1.663083	0.013044	0.035057
NRIP2	113258024	3.166214322	1.662759	0.004041	0.012929
RSL24D1	113269645	3.16592679	1.662628	7.27E-06	4.74E-05
N4BP2L2	113251246	3.165777312	1.66256	1.9E-05	0.000112
EPB41L4A	113254993	3.165122854	1.662261	2.78E-05	0.000158

PARP6	113240936	3.165115072	1.662258	7.75E-06	5.02E-05
CCDC127	113260770	3.164643826	1.662043	2.45E-05	0.00014
LOC113249961	113249961	3.16102192	1.660391	0.001924	0.006793
CRADD	113256160	3.15696846	1.65854	0.000787	0.003125
PPP1R3B	113262116	3.155693908	1.657957	0.001434	0.005279
SNX21	113258661	3.152923641	1.65669	0.000715	0.002875
CXXC5	113261811	3.149974377	1.65534	4.74E-06	3.21E-05
UBAP1L	113240866	3.148494603	1.654662	0.003127	0.010372
RTN4RL1	113268835	3.148352228	1.654597	0.006576	0.019686
PTPRM	113253224	3.147507696	1.65421	0.000406	0.001747
LOC113268544	113268544	3.145088594	1.653101	3.82E-05	0.00021
CCL21	113260498	3.143514349	1.652378	0.007823	0.022777
ZHX2	113252762	3.138849228	1.650236	0.000123	0.000601
FOXN3	113267374	3.136606716	1.649205	1.47E-05	8.91E-05
NUDT13	113259171	3.128458385	1.645452	0.000299	0.001334
LARGE1	113256015	3.127513933	1.645016	0.008572	0.024544
EMCN	113263107	3.124377145	1.643569	2.59E-05	0.000148
LOC113253436	113253436	3.123275119	1.64306	0.008909	0.025359
TMEM236	113241363	3.123263769	1.643054	0.001395	0.005153
SLC45A3	113242479	3.123023743	1.642944	0.001883	0.006671
ZNF385C	113265680	3.122282027	1.642601	0.005295	0.01637
ALKBH8	113260110	3.121870715	1.642411	0.001384	0.005121
PCDH19	113244289	3.115815013	1.63961	0.00244	0.008369
SLC18B1	113259462	3.114702259	1.639094	0.000133	0.000644
BOD1	113264110	3.113880542	1.638714	0.000369	0.001602
SBSPON	113253052	3.113493738	1.638534	0.004117	0.013133
NDUFA13	113256757	3.111234702	1.637487	0.000117	0.000578
NRBF2	113259094	3.10769837	1.635846	9.48E-05	0.000478

PRXL2B	113270607	3.105641184	1.634891	0.000725	0.002911
RNF7	113254010	3.104978476	1.634583	5.11E-05	0.000274
DNAJC28	113264408	3.10462868	1.634421	0.000128	0.000625
CAPN12	113243610	3.100449573	1.632477	0.004223	0.013424
CARD10	113241807	3.099355978	1.631968	0.000917	0.003567
LOC113249681	113249681	3.098280033	1.631468	0.001884	0.006674
VOPP1	113257409	3.097741038	1.631217	0.004617	0.014528
LIMD1	113256777	3.097736055	1.631214	3.14E-05	0.000175
RPL38	113265208	3.096636073	1.630702	0.001757	0.006281
YPEL1	113247045	3.093371988	1.62918	8.11E-05	0.000416
AR	113245650	3.092719756	1.628876	3.91E-05	0.000214
LOC113248605	113248605	3.090354863	1.627773	0.001261	0.004721
MAN2A2	113263716	3.089952192	1.627585	8.81E-05	0.000448
NCALD	113252864	3.089374358	1.627315	0.00385	0.012393
ZNF76	113243840	3.089250653	1.627257	2.22E-05	0.000129
COPS3	113271306	3.088961464	1.627122	2.2E-05	0.000128
VEGFA	113261040	3.088011465	1.626678	0.0001	0.000503
PDE7B	113259477	3.087595446	1.626484	1.6E-05	9.6E-05
TM4SF1	113254048	3.084033852	1.624819	0.000682	0.002763
TOMM7	113261301	3.08198868	1.623862	7.41E-05	0.000383
VEZT	113256140	3.081964155	1.62385	3.8E-05	0.000209
PBX1	113268685	3.08132823	1.623552	1.42E-05	8.63E-05
RHOBTB2	113269988	3.078871668	1.622402	6.48E-05	0.000339
ARHGAP29	113254388	3.076986041	1.621518	0.000246	0.00112
DMWD	113243413	3.075466176	1.620805	0.000118	0.00058
LOC113261640	113261640	3.074625005	1.62041	0.003889	0.012499
AMT	113256872	3.07061378	1.618527	0.000723	0.002904
LOC113257493	113257493	3.070480985	1.618465	0.005897	0.017965

SLIRP	113270282	3.070346893	1.618402	8.82E-05	0.000448
TMLHE	113248335	3.066465787	1.616577	0.000237	0.001082
GUCY1A2	113260215	3.064568545	1.615684	0.004317	0.013677
PLB1	113267984	3.061448905	1.614215	0.011304	0.030966
LOC113243636	113243636	3.059412963	1.613255	5.29E-05	0.000282
NDUFA7	113242904	3.058628647	1.612885	3.51E-05	0.000194
TMED1	113243002	3.057609485	1.612404	7.91E-05	0.000406
LYRM1	113267537	3.056264922	1.61177	0.003377	0.011077
ST3GAL3	113254779	3.055638811	1.611474	1.61E-05	9.67E-05
SCMH1	113254820	3.053364966	1.6104	0.000228	0.001045
USP25	113244784	3.051220059	1.609386	2E-05	0.000117
SORCS2	113245719	3.049896892	1.60876	0.001735	0.006222
GNG7	113242789	3.049512409	1.608579	0.00028	0.001257
MAP4K3	113241667	3.04813507	1.607927	7.15E-06	4.67E-05
PDGFB	113241764	3.047850226	1.607792	0.001582	0.005755
TRIAP1	113267045	3.047335792	1.607548	2.77E-05	0.000157
MTFR1L	113268482	3.047187335	1.607478	5.44E-05	0.000289
FSCN2	113244518	3.046949897	1.607366	0.004356	0.013784
CLDN5	113245974	3.042604861	1.605307	0.001608	0.005828
PSMG3	113247741	3.040650957	1.60438	5.3E-05	0.000282
OTUD4	113255723	3.04010752	1.604122	5.45E-05	0.000289
HADHA	113270772	3.037818145	1.603036	0.005896	0.017965
ARHGAP20	113260078	3.036761746	1.602534	0.00105	0.004018
CMC1	113253855	3.03418237	1.601308	0.000117	0.000579
FOXP3	113265955	3.034167155	1.601301	0.002717	0.009185
GABBR2	113260410	3.033035157	1.600762	0.004718	0.014813
SPATA25	113258663	3.031651749	1.600104	0.004054	0.012959
ISCU	113267071	3.031013367	1.5998	0.001177	0.004438

PARP3	113256932	3.026914092	1.597848	0.01965	0.049571
IL17RD	113256999	3.026326726	1.597568	0.005218	0.016178
KCNJ8	113257805	3.026287878	1.597549	0.003619	0.011776
EFCAB2	113262948	3.021602339	1.595314	7.31E-05	0.000379
MEOX2	113260901	3.02123643	1.595139	0.002503	0.008555
HS3ST2	113267566	3.021017279	1.595034	0.001918	0.006776
LOC113247899	113247899	3.01898878	1.594065	0.000404	0.001738
MVB12B	113266416	3.017825097	1.593509	3.04E-05	0.00017
CPT2	113254653	3.010955135	1.590221	0.000871	0.003415
TNFRSF9	113270556	3.0090342	1.589301	0.000596	0.002449
PATL1	113248883	3.007148392	1.588396	3.04E-05	0.00017
TMEM8B	113260461	3.007100132	1.588373	0.000592	0.002438
SGCG	113251183	3.007094587	1.58837	0.015773	0.041262
SLC7A6	113252404	3.007052225	1.58835	7.14E-05	0.00037
SHISA8	113241716	3.006590546	1.588128	0.011706	0.031939
N6AMT1	113268381	3.004651186	1.587198	0.000104	0.000518
TMEM147	113244830	3.003625296	1.586705	0.00049	0.002062
KCNJ16	113265225	3.002202817	1.586021	0.005876	0.01792
PEX5	113257939	3.001294996	1.585585	7.73E-05	0.000397
SDHAF3	113260440	3.000598165	1.58525	0.000521	0.002176
MRPL19	113243374	2.997723905	1.583868	0.000378	0.001639
IMPA2	113245922	2.997096942	1.583566	3.14E-05	0.000175
ANGPT4	113258433	2.996431864	1.583246	0.000148	0.000711
RGS11	113245366	2.993369447	1.58177	0.003204	0.010593
RPL30	113252895	2.99263702	1.581417	0.019658	0.049573
YAP1	113259721	2.992505241	1.581354	0.000161	0.000769
SELENOW	113243361	2.990183274	1.580234	5.33E-05	0.000284
PTOV1	113243281	2.989402743	1.579857	0.000122	0.000599

MNAT1	113242407	2.98846362	1.579404	4.73E-05	0.000255
DMAC2	113243534	2.985978266	1.578204	4.45E-05	0.000241
SNTA1	113258493	2.978898336	1.574779	0.007595	0.02222
LHFPL1	113241246	2.977397011	1.574052	0.004399	0.013897
ESAM	113259915	2.973636336	1.572228	0.000103	0.000513
CREB5	113262153	2.971958151	1.571414	0.002347	0.008081
NDUFV3	113254594	2.97096868	1.570933	6.08E-05	0.00032
ZNF532	113253462	2.965553097	1.568301	2E-05	0.000117
NDST1	113263951	2.965256413	1.568157	3.91E-05	0.000214
LOC113248316	113248316	2.960585158	1.565882	0.000394	0.001698
TRMT10C	113250698	2.959536003	1.565371	0.000251	0.001138
DNAJC21	113260878	2.958566218	1.564898	2.44E-05	0.00014
MRS2	113264337	2.958544357	1.564888	0.001212	0.004558
LOC113242682	113242682	2.957615628	1.564435	0.004298	0.013627
SMIM11A	113262792	2.956998962	1.564134	8.48E-05	0.000432
HEY1	113253020	2.954742827	1.563033	0.000919	0.003576
USP24	113254622	2.954093942	1.562716	0.000761	0.003036
SESN1	113264757	2.950433559	1.560927	0.000187	0.000879
MBNL2	113251479	2.950423602	1.560922	0.004415	0.013945
AFDN	113247099	2.94858014	1.56002	0.000191	0.000892
NOSTRIN	113250810	2.947519223	1.559501	0.009267	0.026214
KEAP1	113242989	2.947263427	1.559376	0.000882	0.003448
HYKK	113263805	2.945437606	1.558482	0.003228	0.010653
SLC3A1	113246247	2.944148651	1.557851	0.000513	0.002148
FTO	113252242	2.9435233	1.557544	3.28E-05	0.000183
SPRYD7	113251311	2.94269936	1.55714	0.0003	0.001339
DNPEP	113250427	2.941216489	1.556413	9.72E-05	0.000489
GARNL3	113266425	2.940251845	1.55594	0.008053	0.023333

MITF	113257074	2.939075273	1.555362	0.000393	0.001694
HSPA12B	113258381	2.936236129	1.553968	0.000656	0.002668
GFRA1	113251969	2.935761494	1.553735	0.00528	0.016329
DNAJA4	113263798	2.93492034	1.553321	9.03E-05	0.000457
L3MBTL3	113259438	2.934605628	1.553167	8E-05	0.00041
ATP5PO	113263260	2.93403816	1.552888	0.004122	0.013145
LARP1B	113255656	2.931840068	1.551806	3.52E-05	0.000195
CHCHD1	113259188	2.927282865	1.549562	0.000169	0.000801
KYAT1	113266490	2.923363805	1.547629	0.005628	0.017245
LOC113242946	113242946	2.919496922	1.54572	0.011755	0.032052
KLHDC8B	113256866	2.919411931	1.545678	0.00731	0.02149
CCDC68	113253439	2.918585813	1.545269	0.003667	0.011892
LRRC3B	113253861	2.918402612	1.545179	0.003737	0.012089
THRA	113265499	2.918082629	1.545021	0.00027	0.001217
BTBD6	113267181	2.917702393	1.544833	0.001323	0.004922
LRRC4	113263735	2.917198147	1.544583	0.001806	0.006441
PHLDB2	113251155	2.913497426	1.542752	0.000246	0.001119
LOC113241998	113241998	2.913488195	1.542747	0.000583	0.002408
ADAMTSL1	113260599	2.911099064	1.541564	0.001197	0.004504
MTIF3	113251204	2.910563068	1.541298	7.93E-05	0.000406
USHBP1	113256668	2.910311631	1.541174	0.001122	0.004256
SMIM10L1	113257883	2.907503736	1.539781	9.69E-05	0.000488
PPP2R2D	113252071	2.903817087	1.537951	0.000311	0.001381
ZNF583	113248544	2.900189723	1.536147	0.002551	0.008693
TLE2	113242769	2.898648816	1.535381	0.000139	0.000671
NAXE	113245227	2.8983189	1.535216	0.000183	0.000864
TMEM126A	113269391	2.896920576	1.53452	0.000331	0.001457
LOC113245455	113245455	2.895314031	1.53372	2.87E-05	0.000162

DUSP10	113268713	2.894576679	1.533352	0.000147	0.000708
ASPSCR1	113244489	2.894098945	1.533114	0.001354	0.005019
MTMR11	113246122	2.89183422	1.531985	0.007635	0.022299
AES	113242788	2.890980216	1.531559	0.001257	0.004707
LOC113265650	113265650	2.889994747	1.531067	0.000199	0.000926
APBB1	113245850	2.888588584	1.530365	0.000398	0.001717
SDHC	113246688	2.888194773	1.530168	0.00285	0.00957
KIF21A	113257734	2.887569514	1.529856	0.001807	0.006444
VPS9D1	113252123	2.887557279	1.52985	0.000117	0.000579
LOC113249791	113249791	2.88730854	1.529725	0.004843	0.015169
FST	113263911	2.884834234	1.528488	0.008711	0.02488
USP11	113265969	2.883876225	1.528009	0.000546	0.002268
OSBPL1A	113253295	2.883243946	1.527693	0.001308	0.004874
KDM4D	113260162	2.88169687	1.526919	0.006911	0.020475
PVR	113243460	2.879585564	1.525861	0.001149	0.00435
SLC47A2	113271269	2.8791563	1.525646	0.008818	0.025143
PSMG4	113264402	2.877491927	1.524812	0.003848	0.012393
PDCD5	113244718	2.877340909	1.524736	0.000266	0.001198
CLYBL	113251501	2.877097215	1.524614	0.000728	0.002919
THNSL2	113242812	2.875942421	1.524035	0.00016	0.000763
FRS3	113261310	2.875317562	1.523721	0.000592	0.002436
CPED1	113263459	2.875250734	1.523688	0.001445	0.005315
LOC113245523	113245523	2.874889968	1.523507	0.000565	0.002337
LOC113248542	113248542	2.872694052	1.522404	0.001833	0.006522
USH1C	113246620	2.870756202	1.521431	0.008655	0.024745
DUSP23	113246655	2.869531271	1.520815	0.000873	0.003419
ACP2	113264970	2.869230846	1.520664	0.000158	0.000755
KAT5	113244098	2.868804899	1.52045	0.000119	0.000584

ZFP92	113247841	2.867292399	1.519689	0.008112	0.023467
PYROXD2	113251809	2.866435352	1.519258	0.005347	0.016519
RTL10	113245950	2.866365346	1.519223	0.001351	0.005013
GATA6	113253276	2.864077732	1.518071	0.012175	0.033026
USP53	113255610	2.862461563	1.517256	0.000126	0.000618
MAB21L1	113251254	2.8597858	1.515907	0.009872	0.027617
DENND1B	113242587	2.859505159	1.515766	0.001617	0.005857
LRIG1	113257056	2.858371338	1.515193	0.00987	0.027617
DEAF1	113248806	2.857667686	1.514838	8.56E-05	0.000436
PRMT1	113243287	2.856683873	1.514341	9.24E-05	0.000466
RFK	113269838	2.855092535	1.513538	0.000113	0.000559
ATP5IF1	113268421	2.852917366	1.512438	0.002292	0.007929
ZNF662	113253831	2.849905462	1.510914	0.001017	0.00391
IARS2	113268724	2.849463381	1.51069	0.000836	0.0033
SERTAD4	113262655	2.848330683	1.510117	0.019824	0.049863
SMYD2	113262691	2.848065215	1.509982	8.94E-05	0.000453
LOC113248302	113248302	2.845539536	1.508702	0.000848	0.00334
STUB1	113245397	2.844908975	1.508382	0.000667	0.002704
PACS2	113267179	2.839461873	1.505618	0.000345	0.001511
UBXN1	113246911	2.838427217	1.505092	0.000201	0.000936
THAP12	113269339	2.838329417	1.505042	0.000511	0.002142
RAB40B	113244465	2.83753057	1.504636	0.005138	0.015963
ABLIM3	113263929	2.833253489	1.50246	0.008185	0.023616
EIF4E3	113257082	2.831513015	1.501573	0.000257	0.001164
PALM	113242702	2.831354661	1.501492	0.004201	0.013364
HMGCLL1	113261167	2.830764188	1.501192	0.009027	0.025627
CXCL14	113261492	2.826119282	1.498822	0.0006	0.002464
FOXO4	113245656	2.825672457	1.498594	9.44E-05	0.000476

NUDT6	113255639	2.823321547	1.497393	0.000174	0.000823
SLC43A1	113246534	2.819761773	1.495573	7.82E-05	0.000401
SAR1B	113261745	2.817270929	1.494298	0.001116	0.004234
TIMM13	113242767	2.816740185	1.494026	0.000191	0.000894
COQ3	113264805	2.816310879	1.493807	0.000366	0.001594
CFAP58	113251623	2.813905165	1.492574	0.003322	0.010932
NOD1	113262332	2.81337358	1.492301	5.25E-05	0.000281
POLR1E	113260444	2.81193223	1.491562	6.06E-05	0.00032
LOC113246836	113246836	2.811013111	1.49109	0.007408	0.021724
ITGB8	113261164	2.810524774	1.49084	0.007735	0.022547
FBXL17	113255014	2.806189301	1.488612	4.15E-05	0.000225
NKIRAS1	113253873	2.804711617	1.487852	0.001512	0.005535
OPN1SW	113263870	2.804583554	1.487787	0.008516	0.024405
FKRP	113243391	2.802230364	1.486576	6.49E-05	0.000339
LURAP1	113254728	2.801988129	1.486451	0.005938	0.018069
RPS28	113242905	2.801091137	1.485989	0.008745	0.024959
DCXR	113244486	2.800603058	1.485738	0.000935	0.003633
GCSH	113252527	2.800183062	1.485521	0.003667	0.011892
LRPPRC	113246283	2.800156925	1.485508	0.003626	0.011793
FIBIN	113265095	2.79973605	1.485291	0.0028	0.009438
CCSER2	113259236	2.799554721	1.485197	0.000737	0.002953
PSEN2	113262931	2.795198781	1.482951	5.56E-05	0.000295
NFIB	113260613	2.795157034	1.482929	0.003377	0.011077
EIF2A	113254058	2.794226593	1.482449	0.000892	0.003486
HTR1B	113261102	2.793797258	1.482227	0.018017	0.046033
SHANK3	113241594	2.79354764	1.482098	0.000203	0.000941
SLC39A2	113248487	2.790749426	1.480653	0.006009	0.018262
MARS2	113250623	2.789786053	1.480154	0.000561	0.002323

STARD7	113241969	2.789785696	1.480154	0.002021	0.007094
LSM14B	113258785	2.789705375	1.480113	2.93E-05	0.000165
TIMM8B	113260055	2.788768646	1.479628	0.000281	0.001259
PGP	113245113	2.787390235	1.478915	0.000244	0.001112
SYNGR2	113244572	2.786576912	1.478494	0.000808	0.003198
SOX8	113245394	2.786111226	1.478253	0.005526	0.016982
RDH5	113256460	2.786091712	1.478243	0.006679	0.019937
ZCCHC24	113259220	2.785344893	1.477856	0.000496	0.002083
MTUS1	113262156	2.785128402	1.477744	0.007893	0.022948
MRPL34	113256673	2.776683984	1.473363	0.000734	0.002944
PSMC3	113264959	2.776673875	1.473358	0.000691	0.002791
CUNH11orf94	113264998	2.775642318	1.472822	0.012065	0.032766
PPP1R14B	113244179	2.77503988	1.472509	0.000957	0.003706
MRPL11	113244065	2.774259528	1.472103	0.000456	0.001929
ZNHIT2	113244132	2.773840008	1.471885	0.000182	0.000858
ABCC2	113251819	2.773251518	1.471578	0.005678	0.017384
ARHGEF15	113271183	2.772692735	1.471288	0.000658	0.002674
AKT1S1	113243278	2.770359677	1.470073	0.00129	0.004812
LOC113257828	113257828	2.769482881	1.469617	0.000444	0.001884
SNRPN	113263595	2.767657817	1.468666	0.002025	0.007106
KLF2	113256645	2.766123411	1.467866	0.000154	0.000738
ACVR2A	113250906	2.765061207	1.467311	0.001976	0.006956
LOC113249995	113249995	2.764735173	1.467141	0.004745	0.014883
ANKRD53	113244188	2.763558236	1.466527	0.00486	0.015196
NTAN1	113270768	2.76154135	1.465474	0.000363	0.001578
TMEM94	113244634	2.760900033	1.465139	5.41E-05	0.000287
GATC	113267042	2.760692971	1.46503	0.000374	0.001624
HSD17B8	113243793	2.759606558	1.464463	0.000187	0.000876

RRM2B	113252861	2.753958932	1.461507	0.000329	0.001451
ELOB	113245416	2.753279535	1.461151	0.000686	0.002772
APPL2	113256053	2.750749363	1.459825	0.001015	0.003902
SNRNP25	113245172	2.749923821	1.459392	0.000121	0.000596
ZNF365	113259088	2.748556723	1.458674	0.0031	0.010285
LOC113242771	113242771	2.747428928	1.458082	0.004071	0.013003
ETV7	113243859	2.746515693	1.457603	0.018037	0.046076
RASD1	113271303	2.746160388	1.457416	0.001874	0.006645
ISPD	113260950	2.746087227	1.457377	0.004381	0.013849
LOC113247766	113247766	2.744715613	1.456657	0.004263	0.013538
NKAPL	113248162	2.744366229	1.456473	0.013088	0.035144
MRPS16	113259173	2.743139757	1.455828	0.000123	0.000601
GPRASP1	113244313	2.742820733	1.45566	0.014852	0.039145
ZNF25	113258958	2.741605915	1.455021	0.00039	0.001686
LOC113252203	113252203	2.740667593	1.454527	0.000285	0.001276
CD99L2	113246822	2.73968058	1.454008	0.002996	0.010008
CX3CL1	113252283	2.739317228	1.453816	0.007124	0.021028
TRIM3	113245858	2.737538772	1.452879	0.002452	0.008404
LOC113245502	113245502	2.737483985	1.452851	0.000184	0.000865
SEMA6A	113261619	2.734838206	1.451455	0.000199	0.000926
METAP1	113263122	2.733324154	1.450657	0.000323	0.001427
HABP4	113269773	2.728440448	1.448077	0.000495	0.002083
SMTN	113245049	2.7277959	1.447736	0.000224	0.001031
S100A13	113245338	2.724239579	1.445854	0.000407	0.001748
VMO1	113271087	2.723303807	1.445358	0.017753	0.045462
NAT14	113247427	2.722950948	1.445171	0.008305	0.023896
MARCH9	113256359	2.720545463	1.443896	0.000845	0.003327
DRG2	113271293	2.719660812	1.443427	0.000101	0.000504

LOC113250465	113250465	2.716306518	1.441646	0.000369	0.001602
PDLIM1	113251752	2.714350661	1.440607	0.001248	0.004676
ENPP5	113261237	2.713108734	1.439947	0.000356	0.001553
HUS1	113257638	2.710556982	1.438589	0.000861	0.003385
MLH3	113270322	2.710137689	1.438366	0.000737	0.002952
SH3BP4	113250314	2.707829001	1.437137	0.002206	0.007671
BANF1	113244083	2.704376322	1.435296	0.000347	0.001516
POLR2M	113269673	2.704156344	1.435179	0.000427	0.001819
LOC113244925	113244925	2.70369669	1.434933	0.000168	0.000797
PCYT1A	113252916	2.701390734	1.433702	0.000203	0.000942
LOC113246337	113246337	2.700568588	1.433263	0.001753	0.006275
NINJ2	113258034	2.700170122	1.43305	0.010105	0.028151
CIAO2B	113252339	2.700167581	1.433049	0.000227	0.001042
MOB2	113243948	2.696608749	1.431146	0.000262	0.001182
SELENOK	113256985	2.696058847	1.430852	0.000332	0.001459
CD59	113265058	2.6940546	1.429779	0.007293	0.021452
FER1L6	113252749	2.691273961	1.428289	0.008612	0.024644
ETFBKMT	113257750	2.690743813	1.428005	0.002052	0.007193
SYP	113265951	2.690571135	1.427912	0.006351	0.019124
ADGRF5	113261229	2.690265488	1.427749	0.009159	0.025949
POMGNT2	113253834	2.688880252	1.427006	0.013858	0.036857
ZADH2	113253530	2.688598982	1.426855	0.000411	0.00176
MRPS18C	113263198	2.688381423	1.426738	0.000259	0.001171
OXSM	113253864	2.687339432	1.426179	0.000595	0.002449
SLC25A25	113266451	2.684895286	1.424866	0.001313	0.004892
DLAT	113260048	2.684746162	1.424786	0.00604	0.018341
IKZF4	113256443	2.684239857	1.424514	0.000736	0.002952
LOC113261535	113261535	2.683102833	1.423902	0.00159	0.005779

GJA5	113247790	2.681869164	1.423239	0.001486	0.00545
ARHGEF25	113256370	2.681421871	1.422998	0.0071	0.020977
WDR77	113244430	2.680997402	1.42277	0.000306	0.001363
GADD45G	113270128	2.679192922	1.421798	0.00133	0.004939
EEF2K	113267554	2.678286548	1.42131	0.000217	0.001002
ETS2	113258013	2.6782852	1.42131	0.000166	0.000791
MTHFR	113270501	2.675957982	1.420055	0.000304	0.001353
LOC113251149	113251149	2.675435402	1.419774	0.01477	0.038954
DCAF8	113246657	2.674621904	1.419335	0.002685	0.009097
HSD17B1	113265419	2.672667376	1.41828	0.006869	0.020374
ECHS1	113252096	2.672265184	1.418063	0.005707	0.017459
TIMMDC1	113251876	2.670442157	1.417079	0.000789	0.003131
ARAF	113265980	2.669560783	1.416602	0.000345	0.00151
ZAN	113267522	2.668748636	1.416163	0.007909	0.022982
ZNF846	113242959	2.668407801	1.415979	0.012227	0.033127
CUNH2orf92	113246726	2.668304856	1.415924	0.008497	0.024358
ANKRD33	113257597	2.667899432	1.415704	0.010456	0.028983
ZNF485	113243138	2.667578834	1.415531	0.00499	0.015564
DBP	113243343	2.666944995	1.415188	0.003776	0.012205
CUNH19orf54	113243556	2.666436593	1.414913	0.002747	0.009279
TOM1L2	113271297	2.666237522	1.414805	0.00025	0.001137
LOC113245461	113245461	2.664981594	1.414126	0.002314	0.007989
TPPP	113260777	2.663682692	1.413422	0.004333	0.013718
CAPZA2	113263223	2.663376944	1.413257	0.001727	0.006193
AVPR2	113247864	2.662167697	1.412601	0.017666	0.045297
DYNLL2	113268866	2.659436383	1.411121	0.000945	0.003666
STRN3	113266095	2.658935892	1.410849	0.000176	0.000833
EXOSC9	113255628	2.658911862	1.410836	0.000205	0.000953

LGR4	113265090	2.658684374	1.410713	0.000457	0.00193
UBE2E3	113250703	2.658364414	1.410539	0.000183	0.000864
CASKIN2	113244635	2.657993432	1.410338	0.000341	0.001495
HOXA9	113262053	2.657003399	1.4098	0.000771	0.003071
CILP	113240867	2.655169709	1.408804	0.014119	0.037475
MET	113263211	2.649262943	1.405591	0.005247	0.016251
DNAJA3	113270848	2.648236619	1.405032	0.000409	0.001754
CCDC121	113271301	2.646730635	1.404211	0.001098	0.004174
LOC113248539	113248539	2.646360422	1.40401	0.000385	0.001666
ITGA7	113256462	2.645579995	1.403584	0.016156	0.042037
RUFY3	113263301	2.645358128	1.403463	0.000282	0.001266
KIAA1107	113254412	2.643513275	1.402457	0.000533	0.002217
ZNF134	113247987	2.640180258	1.400636	0.000225	0.001034
ACYP1	113270321	2.640123852	1.400606	0.001947	0.006864
ARHGEF7	113248529	2.635871142	1.39828	0.000365	0.001587
PGAM5	113266837	2.634784311	1.397685	0.000139	0.000673
FAM135A	113261136	2.632566089	1.39647	0.000527	0.002196
CDH15	113252138	2.629044551	1.394539	0.01354	0.036128
LOC113253631	113253631	2.628706105	1.394353	0.009331	0.026357
POLM	113257880	2.627902792	1.393912	0.009514	0.026792
EPHX4	113254413	2.626481904	1.393132	0.017802	0.04556
LOC113262545	113262545	2.626332169	1.393049	0.000514	0.002149
ATP6V1FNB	113263980	2.625485498	1.392584	0.011994	0.032626
POLR3C	113247755	2.62503039	1.392334	0.000749	0.002994
SDHAF1	113244838	2.624640219	1.39212	0.002306	0.007965
SLX4	113270863	2.622966178	1.391199	0.000533	0.002217
RABEP1	113271062	2.621750844	1.390531	0.000227	0.001042
TESK1	113260478	2.620448478	1.389814	0.000426	0.001818

ISLR2	113240958	2.618798424	1.388905	0.007874	0.022909
ECSCR	113261806	2.618048231	1.388492	0.002695	0.009128
CUNH16orf89	113270825	2.617842651	1.388378	0.019062	0.048323
ARHGEF28	113255216	2.615795411	1.38725	0.007231	0.021293
ST8SIA5	113253380	2.615175501	1.386908	0.002025	0.007106
EMC9	113246088	2.614557513	1.386567	0.00092	0.003577
SETD3	113267284	2.611390372	1.384818	0.000328	0.001449
DIPK2B	113265908	2.611369114	1.384806	0.003603	0.011731
UACA	113240926	2.61014728	1.384131	0.000617	0.002527
NELL2	113257712	2.609464431	1.383754	0.01239	0.033516
LNX1	113262494	2.608234369	1.383074	0.000426	0.001818
NDUFAF1	113242009	2.607483368	1.382658	0.001123	0.00426
ZC2HC1A	113253024	2.607145311	1.382471	0.007711	0.022488
BCKDK	113267717	2.60631585	1.382012	0.0009	0.003513
ALS2	113250585	2.602743323	1.380033	0.000497	0.002087
RHOD	113244042	2.599590842	1.378285	0.009806	0.027476
MRPL36	113260792	2.599500271	1.378234	0.001909	0.006751
LOC113270508	113270508	2.599294154	1.37812	0.013765	0.036622
GPT	113247624	2.598829924	1.377862	0.006454	0.019389
FRY	113251237	2.598054688	1.377432	0.000553	0.002292
VTN	113269030	2.597344376	1.377037	0.002075	0.00726
DIXDC1	113260059	2.597285198	1.377004	0.0019	0.006725
ZNF142	113250451	2.596902833	1.376792	0.001026	0.00394
PDE4A	113242988	2.596365837	1.376494	0.00323	0.010657
MESP1	113263697	2.594481606	1.375446	0.010024	0.027955
LOC113254720	113254720	2.594398478	1.3754	0.005026	0.015657
AGPAT5	113262188	2.592372217	1.374273	0.000608	0.002494
MRPS35	113257772	2.591817118	1.373964	0.001619	0.00586

MB21D2	113253491	2.588293952	1.372001	0.015779	0.04127
MSRB2	113241336	2.587988228	1.371831	0.005347	0.016519
ALDH1A3	113263632	2.585569228	1.370482	0.001044	0.003995
AP4S1	113266097	2.584333853	1.369792	0.002109	0.00737
ABI2	113250562	2.580843651	1.367843	0.000855	0.003365
PLCE1	113243183	2.580535756	1.367671	0.001177	0.004438
CCDC85B	113244087	2.580446554	1.367621	0.000491	0.002066
HYPK	113241956	2.577598414	1.366028	0.000408	0.001753
BRMS1L	113266134	2.577378006	1.365904	0.000739	0.002959
CUL3	113250393	2.575699561	1.364964	0.001435	0.005281
DLST	113270325	2.571926828	1.36285	0.005233	0.016214
INKA2	113244426	2.568761128	1.361073	0.011983	0.03261
B3GALT4	113243802	2.568661206	1.361017	0.008077	0.023384
TJP1	113263619	2.568406447	1.360874	0.001427	0.005259
LY6G5B	113243725	2.567988267	1.360639	0.001065	0.004066
ZNF484	113270082	2.566855256	1.360002	0.008425	0.02419
DCAF6	113262875	3.634561826	1.861781	0.000659	0.002677
CUNH9orf16	113266457	3.63428294	1.861671	3.14E-05	0.000175
ZNF391	113264258	3.632274446	1.860873	2.25E-05	0.00013
CCDC136	113263909	3.631012608	1.860372	4.25E-05	0.00023
ZYG11B	113254661	3.626846199	1.858716	4E-05	0.000218
MRPL21	113243993	3.615653444	1.854256	8.04E-06	5.18E-05
RAPGEF1	113266537	3.614830168	1.853928	1.72E-05	0.000103
FASTK	113241480	3.611712036	1.852683	7.63E-06	4.95E-05
NOX4	113269421	3.61000922	1.852003	0.002226	0.007732
LOC113247021	113247021	3.606306538	1.850522	0.001393	0.005149
YPEL2	113268888	3.605804345	1.850321	2.2E-05	0.000128
AVPI1	113251796	3.603725364	1.849489	0.000616	0.002525

PLEKHH3	113265409	3.603014788	1.849205	3.91E-06	2.69E-05
EML1	113267272	3.601332967	1.848531	0.000235	0.001077
PIH1D1	113243302	3.599848866	1.847936	2.04E-06	1.47E-05
CGNL1	113269670	3.59961167	1.847841	2.86E-05	0.000162
ANO5	113265108	3.598247884	1.847295	0.006651	0.019871
GMPS	113254098	3.593739088	1.845486	0.000134	0.000651
SLC7A6OS	113252408	3.592077977	1.844819	1.13E-05	7.03E-05
NEDD4L	113253456	3.590042364	1.844001	4.92E-06	3.32E-05
GAL3ST2	113249264	3.588002696	1.843181	0.000785	0.003116
F3	113254382	3.58712502	1.842828	0.001606	0.005826
TMEM134	113244024	3.586823694	1.842707	2.17E-06	1.56E-05
APPL1	113257000	3.586415354	1.842543	3.51E-06	2.43E-05
CRYBG3	113250328	3.58226459	1.840872	0.000178	0.000843
MEF2A	113263641	3.579281776	1.83967	6.59E-07	5.2E-06
ADCY10	113262877	3.579032156	1.83957	0.003191	0.010557
PAIP2B	113244155	3.576908199	1.838713	2.02E-05	0.000118
ATP5MC3	113250747	3.575720243	1.838234	0.001031	0.003954
CEP85L	113259392	3.574804437	1.837864	5.18E-06	3.49E-05
TMEM159	113267545	3.573363401	1.837283	1.3E-06	9.7E-06
ZNF524	113247354	3.56960476	1.835764	9.97E-06	6.3E-05
TUFM	113267631	3.569499479	1.835722	0.000335	0.001469
DLD	113258483	3.566928697	1.834682	0.001165	0.004399
CRIM1	113241217	3.564725219	1.833791	9.95E-05	0.000499
SDHA	113260769	3.551950163	1.828611	0.000354	0.001546
CLEC14A	113266145	3.551781527	1.828543	4.67E-06	3.17E-05
SDR39U1	113246062	3.549307038	1.827537	9.03E-05	0.000457
TMEM30B	113242411	3.54742764	1.826773	0.002505	0.008557
LOC113258537	113258537	3.546022548	1.826202	1.72E-05	0.000102

LOC113253205	113253205	3.545114425	1.825832	2.89E-05	0.000164
PCBD2	113261752	3.544442697	1.825559	4.42E-06	3.01E-05
ADAMTSL3	113263758	3.544401778	1.825542	0.000146	0.000702
PLCB4	113258346	3.539653277	1.823608	0.000218	0.001005
CATSPER3	113261753	3.536271087	1.822229	0.001098	0.004174
ZXDC	113257283	3.532674258	1.820761	2.51E-06	1.78E-05
VEGFB	113244181	3.528584524	1.81909	0.001352	0.005014
TIGD3	113243923	3.526810685	1.818364	0.001581	0.005754
APOPT1	113267216	3.524507604	1.817422	1.52E-05	9.17E-05
LOC113265291	113265291	3.520374274	1.815729	8.54E-06	5.48E-05
PIK3IP1	113245057	3.518942195	1.815142	0.000187	0.000879
PPFIBP1	113257771	3.510416902	1.811642	3.28E-06	2.28E-05
PDHX	113265044	3.507148103	1.810298	0.000126	0.000617
LOC113248583	113248583	3.503187132	1.808668	0.000305	0.001356
RPS29	113266188	3.499883319	1.807307	0.00435	0.013767
COQ6	113270341	3.49963143	1.807203	3E-06	2.1E-05
YBX2	113271116	3.499617162	1.807197	0.000601	0.002467
MAVS	113258384	3.4958603	1.805648	1.64E-06	1.2E-05
ACKR3	113250308	3.488821828	1.80274	0.003605	0.011736
EIF3G	113242974	3.488490533	1.802603	0.000421	0.0018
ENHO	113260508	3.488172505	1.802471	0.000769	0.003064
SPINK2	113262527	3.483467	1.800524	0.000823	0.003254
MRPS25	113257208	3.480569347	1.799323	4.47E-06	3.04E-05
SLC4A4	113263295	3.480401025	1.799254	0.007118	0.021017
LOC113247912	113247912	3.47871395	1.798554	0.002339	0.008064
ATXN7L2	113244924	3.475356065	1.797161	0.000112	0.000557
POU6F1	113257612	3.472595388	1.796014	6.11E-05	0.000322
AMOTL1	113259818	3.468205742	1.794189	4.56E-05	0.000246

PRKAR2A	113256848	3.467441067	1.793871	0.000783	0.003113
LOC113254662	113254662	3.465723371	1.793157	0.000167	0.000793
ZNF594	113271063	3.463772534	1.792344	0.001363	0.00505
ID1	113258459	3.461662916	1.791465	1.94E-05	0.000114
DIP2C	113241102	3.459304406	1.790482	1.98E-06	1.43E-05
CREM	113244224	3.458214465	1.790027	1.19E-05	7.41E-05
MAP4	113256822	3.457188853	1.789599	0.002493	0.008523
CUNH14orf132	113267299	3.455865418	1.789047	0.012174	0.033026
RIC8B	113256034	3.454698727	1.78856	4.38E-06	2.99E-05
CDH6	113260848	3.452467856	1.787628	0.000698	0.002819
LRRC66	113262481	3.449724097	1.786481	0.000739	0.002959
VMA21	113246824	3.444533538	1.784309	2.16E-05	0.000126
NDUFC1	113255686	3.442579605	1.78349	1.68E-05	0.0001
SGCB	113262483	3.442086369	1.783283	0.000701	0.002825
LOC113260556	113260556	3.440184957	1.782486	0.002187	0.007616
ACAA2	113253408	3.439193221	1.78207	0.000414	0.001775
OLFML2A	113266401	3.434517205	1.780107	0.000115	0.000569
HINT3	113259418	3.434023859	1.7799	0.000189	0.000886
AMIGO2	113257695	3.43372626	1.779775	0.01055	0.029196
PDCD2L	113244745	3.432722361	1.779353	0.000268	0.001207
TMEM255A	113270909	3.426475939	1.776726	0.003208	0.010606
CPEB3	113243165	3.425567628	1.776343	3.1E-05	0.000173
LOC113265073	113265073	3.418345673	1.773298	0.007151	0.021095
NDUFB1	113267167	3.415993453	1.772305	5.17E-06	3.48E-05
UBE3D	113261067	3.415799139	1.772223	2.93E-05	0.000165
NDUFS5	113268254	3.411185816	1.770273	6.72E-05	0.00035
LRRC28	113263643	3.410676985	1.770058	9.2E-05	0.000464
ASB9	113240712	3.409259744	1.769459	0.00058	0.002394

LAMA3	113253294	3.407777798	1.768831	2.38E-05	0.000137
NR3C2	113255738	3.406989183	1.768497	4.56E-05	0.000246
RAMP3	113257714	3.406345448	1.768225	0.000741	0.002967
LYSMD2	113269627	3.404966137	1.76764	9.9E-05	0.000497
RAB9B	113248103	3.395980463	1.763828	0.002074	0.007258
MICU3	113262162	3.391900113	1.762094	7.07E-05	0.000367
IGFBP3	113257692	3.391405912	1.761883	0.000638	0.002602
PAQR6	113245241	3.388994821	1.760857	0.001753	0.006275
HOXD8	113250741	3.38647232	1.759783	0.000437	0.00186
ATP5F1E	113258773	3.383238802	1.758405	0.001436	0.005284
PPT2	113243752	3.382448144	1.758068	3.13E-05	0.000175
CRYGN	113241492	3.381813683	1.757797	0.002068	0.00724
LOC113249678	113249678	3.381784643	1.757785	0.00072	0.002894
GKAP1	113269812	3.381160965	1.757519	2.87E-05	0.000163
F8	113248344	3.378847678	1.756531	8.01E-06	5.17E-05
AKTIP	113252238	3.3778185	1.756092	5.18E-06	3.48E-05
RPL36	113242860	3.377394781	1.755911	0.001506	0.005519
PPIF	113259219	3.377145178	1.755804	5.11E-05	0.000274
PAX1	113246770	3.375600611	1.755144	0.000765	0.003053
NICN1	113256875	3.374928071	1.754857	5.15E-05	0.000276
DOCK9	113251489	3.372852403	1.753969	0.000307	0.001367
KLHL3	113261768	3.372512597	1.753824	0.000216	0.001
EIF1	113265448	3.369293016	1.752446	0.002273	0.00787
ETFA	113248087	3.366827865	1.75139	0.0006	0.002463
PPP1R2	113253297	3.365070153	1.750637	9.29E-05	0.000469
MYLK3	113252202	3.364743685	1.750497	0.000791	0.003136
CA8	113268071	3.362669725	1.749607	0.005569	0.017079
TIMM10	113246386	3.360979145	1.748882	1.46E-05	8.86E-05

DNAJB4	113254500	3.360159745	1.74853	3.75E-05	0.000206
HSF2BP	113253534	3.359879498	1.748409	0.003219	0.010634
KANK3	113242903	3.35878905	1.747941	1.97E-05	0.000115
EGFL7	113248635	3.358451504	1.747796	0.000227	0.001044
LOC113249852	113249852	3.345980624	1.742429	0.004494	0.014173
LOC113251944	113251944	3.344200335	1.741661	2.83E-05	0.000161
RPS11	113243297	3.342725509	1.741025	0.015891	0.041499
NFU1	113244593	3.342541885	1.740946	0.000218	0.001006
MTHFD2L	113263273	3.342135295	1.74077	0.001544	0.005636
PLCH2	113270598	3.340930487	1.74025	0.004913	0.015351
LOC113266881	113266881	3.340637861	1.740124	0.003867	0.012438
DLL4	113242017	3.337376829	1.738715	9.73E-05	0.000489
IL20RB	113253976	3.33627415	1.738238	0.001155	0.00437
SEZ6L2	113267659	3.333547304	1.737058	0.00139	0.00514
SLC35E4	113245043	3.333370453	1.736982	0.001078	0.004107
STAC2	113265524	3.331141012	1.736016	0.003093	0.010266
KIF5A	113256376	3.330410028	1.7357	0.002441	0.008371
KLC2	113244076	3.329548735	1.735327	2.99E-05	0.000168
GPR176	113242047	3.329280627	1.73521	0.003092	0.010262
EEPD1	113262749	3.3271882	1.734303	1.7E-05	0.000101
FAN1	113263610	3.326754004	1.734115	3.25E-06	2.26E-05
METTL25	113256211	3.324669791	1.733211	1.31E-05	8.04E-05
HOXA4	113261989	3.321005488	1.73162	0.002582	0.008785
TRNP1	113268445	3.319231645	1.730849	0.00246	0.008424
KIRREL2	113244768	3.317684807	1.730177	0.004848	0.015177
OTUD6B	113252945	3.314109655	1.728621	7E-06	4.58E-05
LOC113245484	113245484	3.313583272	1.728392	0.002849	0.009569
HSD11B1	113262645	3.312689487	1.728003	0.004546	0.014315

AIMP2	113267935	3.31022476	1.726929	5.39E-06	3.61E-05
ZSCAN23	113248160	3.309281715	1.726518	0.000293	0.001312
CBX7	113241765	3.308187355	1.726041	0.000142	0.000685
SORBS3	113270000	3.304664506	1.724504	1.94E-05	0.000114
NDUFA2	113261833	3.304150656	1.724279	4.75E-05	0.000255
PPIP5K1	113241961	3.303392757	1.723949	7.88E-06	5.1E-05
LOC113251647	113251647	3.300370629	1.722628	6.58E-05	0.000343
LOC113249996	113249996	3.299999582	1.722466	0.000824	0.003256
ZNF768	113267684	3.296789498	1.721062	2.54E-05	0.000145
LRRFIP2	113253771	3.291459626	1.718728	8.89E-06	5.68E-05
SMC1B	113241654	3.289621151	1.717921	0.003595	0.011709
CD101	113244376	3.288270827	1.717329	0.008184	0.023616
CNTNAP2	113241449	3.287882427	1.717159	0.007861	0.022875
DOCK6	113243014	3.287431334	1.716961	1.3E-05	7.99E-05
STAU2	113253047	3.287052728	1.716795	8.96E-06	5.72E-05
NCBP2-AS2	113253142	3.285664605	1.716185	3.53E-05	0.000195
FEZ1	113259906	3.28492096	1.715859	0.00271	0.009173
RTKN	113243549	3.282075499	1.714608	2.72E-05	0.000155
HSPB1	113267813	3.278535964	1.713052	0.000425	0.001817
IL33	113260625	3.276372815	1.7121	0.002602	0.008838
TMEM150A	113243094	3.27145468	1.709932	2.66E-05	0.000152
LOC113247253	113247253	3.271232642	1.709834	1.3E-05	7.98E-05
PERP	113259487	3.270614368	1.709562	0.002334	0.008052
TMEM63C	113270297	3.266683308	1.707827	0.004453	0.014053
ANHX	113266821	3.264827791	1.707007	0.004274	0.013565
ARHGEF10L	113270439	3.264590935	1.706902	7.82E-06	5.07E-05
BNIP3	113252072	3.264225442	1.706741	0.002829	0.009515
LOC113241880	113241880	3.262983836	1.706192	0.008391	0.024114

TFB2M	113262945	3.261730541	1.705638	6.36E-05	0.000334
ECSIT	113243030	3.261076682	1.705348	1.06E-05	6.69E-05
CTF1	113267705	3.260390783	1.705045	0.003193	0.01056
CPN1	113251825	3.259239549	1.704535	0.001032	0.003957
TBX3	113267002	3.258324578	1.70413	0.00092	0.003577
TOMM40L	113246746	3.257914495	1.703949	5.92E-05	0.000312
ALKBH5	113271291	3.255584654	1.702917	6.56E-05	0.000343
BHLHB9	113244314	3.255550596	1.702902	2.94E-05	0.000166
LOC113242947	113242947	3.254080071	1.70225	0.000158	0.000755
LOC113247867	113247867	3.252606655	1.701596	0.000699	0.002822
CUNH6orf203	113264776	3.252515718	1.701556	3.9E-05	0.000213
CACNG7	113247410	3.25223201	1.70143	0.006226	0.018821
B3GALNT2	113259001	3.250909546	1.700843	7.3E-05	0.000378
HIGD2A	113264125	3.247908071	1.699511	6.3E-05	0.000331
MRPL24	113245221	3.247124686	1.699163	2.84E-05	0.000161
MAP3K10	113243573	3.247044922	1.699127	0.00012	0.000589
GRTP1	113247574	3.244403028	1.697953	0.005745	0.017561
GBX1	113241483	3.244314094	1.697913	0.003987	0.012774
FBXL8	113252354	3.243087029	1.697368	0.002653	0.008994
WSCD1	113271054	3.241164239	1.696512	1.95E-05	0.000114
TMEM51	113270481	3.240518983	1.696225	0.00107	0.004082
MYO18A	113269060	3.238414511	1.695288	0.000779	0.003097
PADI2	113270446	3.236029531	1.694225	0.009881	0.02763
GPR75	113245790	3.235099395	1.69381	0.003227	0.010653
CCDC85A	113245393	3.232152111	1.692495	0.003833	0.01236
ZBTB47	113253822	3.2260961	1.689789	0.000535	0.002222
MMP24	113258534	3.225922897	1.689712	0.001628	0.005891
TRHDE	113256259	3.225791202	1.689653	0.005813	0.017745

KCNQ5	113261119	3.22546534	1.689507	0.008045	0.023317
RPL35A	113252711	3.223755385	1.688742	0.012074	0.032784
NRN1	113264419	3.222334922	1.688106	0.005384	0.016621
NDUFB3	113250598	3.21772801	1.686042	6.63E-05	0.000346
MYORG	113260509	3.215011339	1.684824	0.008114	0.023467
PSTPIP2	113253373	3.212991557	1.683917	0.000226	0.001039
LOC113254434	113254434	3.209359011	1.682285	9.07E-05	0.000459
CIART	113246110	3.208303655	1.681811	0.006768	0.020136
MRPS2	113266589	3.20653382	1.681015	0.000104	0.00052
THSD1	113251287	3.205870368	1.680716	0.003461	0.011332
STAC	113253765	3.205029479	1.680338	0.013498	0.036046
SLC25A42	113256741	3.203746704	1.67976	0.002398	0.008235
SQSTM1	113261555	3.20347816	1.679639	0.002571	0.008752
TCTA	113256877	3.203353044	1.679583	1.4E-05	8.55E-05
RITA1	113266990	3.202574242	1.679232	1.28E-05	7.86E-05
CDC37L1	113270165	3.198859087	1.677557	2.56E-06	1.82E-05
ISLR	113240961	3.197064578	1.676748	0.000753	0.00301
TSPAN3	113241005	3.193557791	1.675165	0.000507	0.002125
HES4	113270655	3.192903465	1.674869	4.17E-05	0.000226
L2HGDH	113266207	3.192701414	1.674778	4.56E-05	0.000246
SH3GL3	113263757	3.191801043	1.674371	0.001704	0.006125
CUNH16orf95	113252171	3.187520806	1.672435	0.010321	0.028684
NOV	113252778	3.18730676	1.672338	0.005888	0.017944
LOC113271076	113271076	3.183638048	1.670676	0.006671	0.019916
RTN4	113245629	3.183131295	1.670447	0.01305	0.035064
GPIHBP1	113247595	3.18062491	1.66931	0.0023	0.007948
ITGA3	113265609	3.18030223	1.669164	0.000486	0.002047
LOC113270384	113270384	3.180292033	1.669159	0.000119	0.000587

WSB2	113267016	3.180198765	1.669117	8.34E-05	0.000426
RUNDC3B	113259507	3.179494246	1.668797	4.68E-05	0.000252
DLGAP1	113253243	3.179394387	1.668752	0.002276	0.007879
LOC113247252	113247252	3.175718691	1.667083	0.003653	0.011857
AGMO	113260890	3.174381325	1.666475	0.006392	0.019223
TCEAL1	113249836	3.173393193	1.666026	4.06E-05	0.000221
LOC113246566	113246566	3.17200725	1.665396	5.81E-05	0.000307
LOC113249742	113249742	3.170074408	1.664517	3.01E-05	0.000169
CMC4	113248350	3.169996927	1.664481	0.000369	0.001602
SPARCL1	113263168	3.168479972	1.663791	0.00688	0.020393
CUNH8orf59	113252990	3.167856619	1.663507	2.91E-05	0.000164
HOXB8	113265579	3.166925327	1.663083	0.013044	0.035057
NRIP2	113258024	3.166214322	1.662759	0.004041	0.012929
RSL24D1	113269645	3.16592679	1.662628	7.27E-06	4.74E-05
N4BP2L2	113251246	3.165777312	1.66256	1.9E-05	0.000112
EPB41L4A	113254993	3.165122854	1.662261	2.78E-05	0.000158
PARP6	113240936	3.165115072	1.662258	7.75E-06	5.02E-05
CCDC127	113260770	3.164643826	1.662043	2.45E-05	0.00014
LOC113249961	113249961	3.16102192	1.660391	0.001924	0.006793
CRADD	113256160	3.15696846	1.65854	0.000787	0.003125
PPP1R3B	113262116	3.155693908	1.657957	0.001434	0.005279
SNX21	113258661	3.152923641	1.65669	0.000715	0.002875
CXXC5	113261811	3.149974377	1.65534	4.74E-06	3.21E-05
UBAP1L	113240866	3.148494603	1.654662	0.003127	0.010372
RTN4RL1	113268835	3.148352228	1.654597	0.006576	0.019686
PTPRM	113253224	3.147507696	1.65421	0.000406	0.001747
LOC113268544	113268544	3.145088594	1.653101	3.82E-05	0.00021
CCL21	113260498	3.143514349	1.652378	0.007823	0.022777

ZHX2	113252762	3.138849228	1.650236	0.000123	0.000601
FOXN3	113267374	3.136606716	1.649205	1.47E-05	8.91E-05
NUDT13	113259171	3.128458385	1.645452	0.000299	0.001334
LARGE1	113256015	3.127513933	1.645016	0.008572	0.024544
EMCN	113263107	3.124377145	1.643569	2.59E-05	0.000148
LOC113253436	113253436	3.123275119	1.64306	0.008909	0.025359
TMEM236	113241363	3.123263769	1.643054	0.001395	0.005153
SLC45A3	113242479	3.123023743	1.642944	0.001883	0.006671
ZNF385C	113265680	3.122282027	1.642601	0.005295	0.01637
ALKBH8	113260110	3.121870715	1.642411	0.001384	0.005121
PCDH19	113244289	3.115815013	1.63961	0.00244	0.008369
SLC18B1	113259462	3.114702259	1.639094	0.000133	0.000644
BOD1	113264110	3.113880542	1.638714	0.000369	0.001602
SBSPON	113253052	3.113493738	1.638534	0.004117	0.013133
NDUFA13	113256757	3.111234702	1.637487	0.000117	0.000578
NRBF2	113259094	3.10769837	1.635846	9.48E-05	0.000478
PRXL2B	113270607	3.105641184	1.634891	0.000725	0.002911
RNF7	113254010	3.104978476	1.634583	5.11E-05	0.000274
DNAJC28	113264408	3.10462868	1.634421	0.000128	0.000625
CAPN12	113243610	3.100449573	1.632477	0.004223	0.013424
CARD10	113241807	3.099355978	1.631968	0.000917	0.003567
LOC113249681	113249681	3.098280033	1.631468	0.001884	0.006674
VOPP1	113257409	3.097741038	1.631217	0.004617	0.014528
LIMD1	113256777	3.097736055	1.631214	3.14E-05	0.000175
RPL38	113265208	3.096636073	1.630702	0.001757	0.006281
YPEL1	113247045	3.093371988	1.62918	8.11E-05	0.000416
AR	113245650	3.092719756	1.628876	3.91E-05	0.000214
LOC113248605	113248605	3.090354863	1.627773	0.001261	0.004721

MAN2A2	113263716	3.089952192	1.627585	8.81E-05	0.000448
NCALD	113252864	3.089374358	1.627315	0.00385	0.012393
ZNF76	113243840	3.089250653	1.627257	2.22E-05	0.000129
COPS3	113271306	3.088961464	1.627122	2.2E-05	0.000128
VEGFA	113261040	3.088011465	1.626678	0.0001	0.000503
PDE7B	113259477	3.087595446	1.626484	1.6E-05	9.6E-05
TM4SF1	113254048	3.084033852	1.624819	0.000682	0.002763
TOMM7	113261301	3.08198868	1.623862	7.41E-05	0.000383
VEZT	113256140	3.081964155	1.62385	3.8E-05	0.000209
PBX1	113268685	3.08132823	1.623552	1.42E-05	8.63E-05
RHOBTB2	113269988	3.078871668	1.622402	6.48E-05	0.000339
ARHGAP29	113254388	3.076986041	1.621518	0.000246	0.00112
DMWD	113243413	3.075466176	1.620805	0.000118	0.00058
LOC113261640	113261640	3.074625005	1.62041	0.003889	0.012499
AMT	113256872	3.07061378	1.618527	0.000723	0.002904
LOC113257493	113257493	3.070480985	1.618465	0.005897	0.017965
SLIRP	113270282	3.070346893	1.618402	8.82E-05	0.000448
TMLHE	113248335	3.066465787	1.616577	0.000237	0.001082
GUCY1A2	113260215	3.064568545	1.615684	0.004317	0.013677
PLB1	113267984	3.061448905	1.614215	0.011304	0.030966
LOC113243636	113243636	3.059412963	1.613255	5.29E-05	0.000282
NDUFA7	113242904	3.058628647	1.612885	3.51E-05	0.000194
TMED1	113243002	3.057609485	1.612404	7.91E-05	0.000406
LYRM1	113267537	3.056264922	1.61177	0.003377	0.011077
ST3GAL3	113254779	3.055638811	1.611474	1.61E-05	9.67E-05
SCMH1	113254820	3.053364966	1.6104	0.000228	0.001045
USP25	113244784	3.051220059	1.609386	2E-05	0.000117
SORCS2	113245719	3.049896892	1.60876	0.001735	0.006222

GNG7	113242789	3.049512409	1.608579	0.00028	0.001257
MAP4K3	113241667	3.04813507	1.607927	7.15E-06	4.67E-05
PDGFB	113241764	3.047850226	1.607792	0.001582	0.005755
TRIAP1	113267045	3.047335792	1.607548	2.77E-05	0.000157
MTFR1L	113268482	3.047187335	1.607478	5.44E-05	0.000289
FSCN2	113244518	3.046949897	1.607366	0.004356	0.013784
CLDN5	113245974	3.042604861	1.605307	0.001608	0.005828
PSMG3	113247741	3.040650957	1.60438	5.3E-05	0.000282
OTUD4	113255723	3.04010752	1.604122	5.45E-05	0.000289
HADHA	113270772	3.037818145	1.603036	0.005896	0.017965
ARHGAP20	113260078	3.036761746	1.602534	0.00105	0.004018
CMC1	113253855	3.03418237	1.601308	0.000117	0.000579
FOXP3	113265955	3.034167155	1.601301	0.002717	0.009185
GABBR2	113260410	3.033035157	1.600762	0.004718	0.014813
SPATA25	113258663	3.031651749	1.600104	0.004054	0.012959
ISCU	113267071	3.031013367	1.5998	0.001177	0.004438
PARP3	113256932	3.026914092	1.597848	0.01965	0.049571
IL17RD	113256999	3.026326726	1.597568	0.005218	0.016178
KCNJ8	113257805	3.026287878	1.597549	0.003619	0.011776
EFCAB2	113262948	3.021602339	1.595314	7.31E-05	0.000379
MEOX2	113260901	3.02123643	1.595139	0.002503	0.008555
HS3ST2	113267566	3.021017279	1.595034	0.001918	0.006776
LOC113247899	113247899	3.01898878	1.594065	0.000404	0.001738
MVB12B	113266416	3.017825097	1.593509	3.04E-05	0.00017
CPT2	113254653	3.010955135	1.590221	0.000871	0.003415
TNFRSF9	113270556	3.0090342	1.589301	0.000596	0.002449
PATL1	113248883	3.007148392	1.588396	3.04E-05	0.00017
TMEM8B	113260461	3.007100132	1.588373	0.000592	0.002438

SGCG	113251183	3.007094587	1.58837	0.015773	0.041262
SLC7A6	113252404	3.007052225	1.58835	7.14E-05	0.00037
SHISA8	113241716	3.006590546	1.588128	0.011706	0.031939
N6AMT1	113268381	3.004651186	1.587198	0.000104	0.000518
TMEM147	113244830	3.003625296	1.586705	0.00049	0.002062
KCNJ16	113265225	3.002202817	1.586021	0.005876	0.01792
PEX5	113257939	3.001294996	1.585585	7.73E-05	0.000397
SDHAF3	113260440	3.000598165	1.58525	0.000521	0.002176
MRPL19	113243374	2.997723905	1.583868	0.000378	0.001639
IMPA2	113245922	2.997096942	1.583566	3.14E-05	0.000175
ANGPT4	113258433	2.996431864	1.583246	0.000148	0.000711
RGS11	113245366	2.993369447	1.58177	0.003204	0.010593
RPL30	113252895	2.99263702	1.581417	0.019658	0.049573
YAP1	113259721	2.992505241	1.581354	0.000161	0.000769
SELENOW	113243361	2.990183274	1.580234	5.33E-05	0.000284
PTOV1	113243281	2.989402743	1.579857	0.000122	0.000599
MNAT1	113242407	2.98846362	1.579404	4.73E-05	0.000255
DMAC2	113243534	2.985978266	1.578204	4.45E-05	0.000241
SNTA1	113258493	2.978898336	1.574779	0.007595	0.02222
LHFPL1	113241246	2.977397011	1.574052	0.004399	0.013897
ESAM	113259915	2.973636336	1.572228	0.000103	0.000513
CREB5	113262153	2.971958151	1.571414	0.002347	0.008081
NDUFV3	113254594	2.97096868	1.570933	6.08E-05	0.00032
ZNF532	113253462	2.965553097	1.568301	2E-05	0.000117
NDST1	113263951	2.965256413	1.568157	3.91E-05	0.000214
LOC113248316	113248316	2.960585158	1.565882	0.000394	0.001698
TRMT10C	113250698	2.959536003	1.565371	0.000251	0.001138
DNAJC21	113260878	2.958566218	1.564898	2.44E-05	0.00014

MRS2	113264337	2.958544357	1.564888	0.001212	0.004558
LOC113242682	113242682	2.957615628	1.564435	0.004298	0.013627
SMIM11A	113262792	2.956998962	1.564134	8.48E-05	0.000432
HEY1	113253020	2.954742827	1.563033	0.000919	0.003576
USP24	113254622	2.954093942	1.562716	0.000761	0.003036
SESN1	113264757	2.950433559	1.560927	0.000187	0.000879
MBNL2	113251479	2.950423602	1.560922	0.004415	0.013945
AFDN	113247099	2.94858014	1.56002	0.000191	0.000892
NOSTRIN	113250810	2.947519223	1.559501	0.009267	0.026214
KEAP1	113242989	2.947263427	1.559376	0.000882	0.003448
HYKK	113263805	2.945437606	1.558482	0.003228	0.010653
SLC3A1	113246247	2.944148651	1.557851	0.000513	0.002148
FTO	113252242	2.9435233	1.557544	3.28E-05	0.000183
SPRYD7	113251311	2.94269936	1.55714	0.0003	0.001339
DNPEP	113250427	2.941216489	1.556413	9.72E-05	0.000489
GARNL3	113266425	2.940251845	1.55594	0.008053	0.023333
MITF	113257074	2.939075273	1.555362	0.000393	0.001694
HSPA12B	113258381	2.936236129	1.553968	0.000656	0.002668
GFRA1	113251969	2.935761494	1.553735	0.00528	0.016329
DNAJA4	113263798	2.93492034	1.553321	9.03E-05	0.000457
L3MBTL3	113259438	2.934605628	1.553167	8E-05	0.00041
ATP5PO	113263260	2.93403816	1.552888	0.004122	0.013145
LARP1B	113255656	2.931840068	1.551806	3.52E-05	0.000195
CHCHD1	113259188	2.927282865	1.549562	0.000169	0.000801
KYAT1	113266490	2.923363805	1.547629	0.005628	0.017245
LOC113242946	113242946	2.919496922	1.54572	0.011755	0.032052
KLHDC8B	113256866	2.919411931	1.545678	0.00731	0.02149
CCDC68	113253439	2.918585813	1.545269	0.003667	0.011892

LRRC3B	113253861	2.918402612	1.545179	0.003737	0.012089
THRA	113265499	2.918082629	1.545021	0.00027	0.001217
BTBD6	113267181	2.917702393	1.544833	0.001323	0.004922
LRRC4	113263735	2.917198147	1.544583	0.001806	0.006441
PHLDB2	113251155	2.913497426	1.542752	0.000246	0.001119
LOC113241998	113241998	2.913488195	1.542747	0.000583	0.002408
ADAMTSL1	113260599	2.911099064	1.541564	0.001197	0.004504
MTIF3	113251204	2.910563068	1.541298	7.93E-05	0.000406
USHBP1	113256668	2.910311631	1.541174	0.001122	0.004256
SMIM10L1	113257883	2.907503736	1.539781	9.69E-05	0.000488
PPP2R2D	113252071	2.903817087	1.537951	0.000311	0.001381
ZNF583	113248544	2.900189723	1.536147	0.002551	0.008693
TLE2	113242769	2.898648816	1.535381	0.000139	0.000671
NAXE	113245227	2.8983189	1.535216	0.000183	0.000864
TMEM126A	113269391	2.896920576	1.53452	0.000331	0.001457
LOC113245455	113245455	2.895314031	1.53372	2.87E-05	0.000162
DUSP10	113268713	2.894576679	1.533352	0.000147	0.000708
ASPSCR1	113244489	2.894098945	1.533114	0.001354	0.005019
MTMR11	113246122	2.89183422	1.531985	0.007635	0.022299
AES	113242788	2.890980216	1.531559	0.001257	0.004707
LOC113265650	113265650	2.889994747	1.531067	0.000199	0.000926
APBB1	113245850	2.888588584	1.530365	0.000398	0.001717
SDHC	113246688	2.888194773	1.530168	0.00285	0.00957
KIF21A	113257734	2.887569514	1.529856	0.001807	0.006444
VPS9D1	113252123	2.887557279	1.52985	0.000117	0.000579
LOC113249791	113249791	2.88730854	1.529725	0.004843	0.015169
FST	113263911	2.884834234	1.528488	0.008711	0.02488
USP11	113265969	2.883876225	1.528009	0.000546	0.002268

OSBPL1A	113253295	2.883243946	1.527693	0.001308	0.004874
KDM4D	113260162	2.88169687	1.526919	0.006911	0.020475
PVR	113243460	2.879585564	1.525861	0.001149	0.00435
SLC47A2	113271269	2.8791563	1.525646	0.008818	0.025143
PSMG4	113264402	2.877491927	1.524812	0.003848	0.012393
PDCD5	113244718	2.877340909	1.524736	0.000266	0.001198
CLYBL	113251501	2.877097215	1.524614	0.000728	0.002919
THNSL2	113242812	2.875942421	1.524035	0.00016	0.000763
FRS3	113261310	2.875317562	1.523721	0.000592	0.002436
CPED1	113263459	2.875250734	1.523688	0.001445	0.005315
LOC113245523	113245523	2.874889968	1.523507	0.000565	0.002337
LOC113248542	113248542	2.872694052	1.522404	0.001833	0.006522
USH1C	113246620	2.870756202	1.521431	0.008655	0.024745
DUSP23	113246655	2.869531271	1.520815	0.000873	0.003419
ACP2	113264970	2.869230846	1.520664	0.000158	0.000755
KAT5	113244098	2.868804899	1.52045	0.000119	0.000584
ZFP92	113247841	2.867292399	1.519689	0.008112	0.023467
PYROXD2	113251809	2.866435352	1.519258	0.005347	0.016519
RTL10	113245950	2.866365346	1.519223	0.001351	0.005013
GATA6	113253276	2.864077732	1.518071	0.012175	0.033026
USP53	113255610	2.862461563	1.517256	0.000126	0.000618
MAB21L1	113251254	2.8597858	1.515907	0.009872	0.027617
DENND1B	113242587	2.859505159	1.515766	0.001617	0.005857
LRIG1	113257056	2.858371338	1.515193	0.00987	0.027617
DEAF1	113248806	2.857667686	1.514838	8.56E-05	0.000436
PRMT1	113243287	2.856683873	1.514341	9.24E-05	0.000466
RFK	113269838	2.855092535	1.513538	0.000113	0.000559
ATP5IF1	113268421	2.852917366	1.512438	0.002292	0.007929

ZNF662	113253831	2.849905462	1.510914	0.001017	0.00391
IARS2	113268724	2.849463381	1.51069	0.000836	0.0033
SERTAD4	113262655	2.848330683	1.510117	0.019824	0.049863
SMYD2	113262691	2.848065215	1.509982	8.94E-05	0.000453
LOC113248302	113248302	2.845539536	1.508702	0.000848	0.00334
STUB1	113245397	2.844908975	1.508382	0.000667	0.002704
PACS2	113267179	2.839461873	1.505618	0.000345	0.001511
UBXN1	113246911	2.838427217	1.505092	0.000201	0.000936
THAP12	113269339	2.838329417	1.505042	0.000511	0.002142
RAB40B	113244465	2.83753057	1.504636	0.005138	0.015963
ABLIM3	113263929	2.833253489	1.50246	0.008185	0.023616
EIF4E3	113257082	2.831513015	1.501573	0.000257	0.001164
PALM	113242702	2.831354661	1.501492	0.004201	0.013364
HMGCLL1	113261167	2.830764188	1.501192	0.009027	0.025627
CXCL14	113261492	2.826119282	1.498822	0.0006	0.002464
FOXO4	113245656	2.825672457	1.498594	9.44E-05	0.000476
NUDT6	113255639	2.823321547	1.497393	0.000174	0.000823
SLC43A1	113246534	2.819761773	1.495573	7.82E-05	0.000401
SAR1B	113261745	2.817270929	1.494298	0.001116	0.004234
TIMM13	113242767	2.816740185	1.494026	0.000191	0.000894
COQ3	113264805	2.816310879	1.493807	0.000366	0.001594
CFAP58	113251623	2.813905165	1.492574	0.003322	0.010932
NOD1	113262332	2.81337358	1.492301	5.25E-05	0.000281
POLR1E	113260444	2.81193223	1.491562	6.06E-05	0.00032
LOC113246836	113246836	2.811013111	1.49109	0.007408	0.021724
ITGB8	113261164	2.810524774	1.49084	0.007735	0.022547
FBXL17	113255014	2.806189301	1.488612	4.15E-05	0.000225
NKIRAS1	113253873	2.804711617	1.487852	0.001512	0.005535

OPN1SW	113263870	2.804583554	1.487787	0.008516	0.024405
FKRP	113243391	2.802230364	1.486576	6.49E-05	0.000339
LURAP1	113254728	2.801988129	1.486451	0.005938	0.018069
RPS28	113242905	2.801091137	1.485989	0.008745	0.024959
DCXR	113244486	2.800603058	1.485738	0.000935	0.003633
GCSH	113252527	2.800183062	1.485521	0.003667	0.011892
LRPPRC	113246283	2.800156925	1.485508	0.003626	0.011793
FIBIN	113265095	2.79973605	1.485291	0.0028	0.009438
CCSER2	113259236	2.799554721	1.485197	0.000737	0.002953
PSEN2	113262931	2.795198781	1.482951	5.56E-05	0.000295
NFIB	113260613	2.795157034	1.482929	0.003377	0.011077
EIF2A	113254058	2.794226593	1.482449	0.000892	0.003486
HTR1B	113261102	2.793797258	1.482227	0.018017	0.046033
SHANK3	113241594	2.79354764	1.482098	0.000203	0.000941
SLC39A2	113248487	2.790749426	1.480653	0.006009	0.018262
MARS2	113250623	2.789786053	1.480154	0.000561	0.002323
STARD7	113241969	2.789785696	1.480154	0.002021	0.007094
LSM14B	113258785	2.789705375	1.480113	2.93E-05	0.000165
TIMM8B	113260055	2.788768646	1.479628	0.000281	0.001259
PGP	113245113	2.787390235	1.478915	0.000244	0.001112
SYNGR2	113244572	2.786576912	1.478494	0.000808	0.003198
SOX8	113245394	2.786111226	1.478253	0.005526	0.016982
RDH5	113256460	2.786091712	1.478243	0.006679	0.019937
ZCCHC24	113259220	2.785344893	1.477856	0.000496	0.002083
MTUS1	113262156	2.785128402	1.477744	0.007893	0.022948
MRPL34	113256673	2.776683984	1.473363	0.000734	0.002944
PSMC3	113264959	2.776673875	1.473358	0.000691	0.002791
CUNH11orf94	113264998	2.775642318	1.472822	0.012065	0.032766

PPP1R14B	113244179	2.77503988	1.472509	0.000957	0.003706
MRPL11	113244065	2.774259528	1.472103	0.000456	0.001929
ZNHIT2	113244132	2.773840008	1.471885	0.000182	0.000858
ABCC2	113251819	2.773251518	1.471578	0.005678	0.017384
ARHGEF15	113271183	2.772692735	1.471288	0.000658	0.002674
AKT1S1	113243278	2.770359677	1.470073	0.00129	0.004812
LOC113257828	113257828	2.769482881	1.469617	0.000444	0.001884
SNRPN	113263595	2.767657817	1.468666	0.002025	0.007106
KLF2	113256645	2.766123411	1.467866	0.000154	0.000738
ACVR2A	113250906	2.765061207	1.467311	0.001976	0.006956
LOC113249995	113249995	2.764735173	1.467141	0.004745	0.014883
ANKRD53	113244188	2.763558236	1.466527	0.00486	0.015196
NTAN1	113270768	2.76154135	1.465474	0.000363	0.001578
TMEM94	113244634	2.760900033	1.465139	5.41E-05	0.000287
GATC	113267042	2.760692971	1.46503	0.000374	0.001624
HSD17B8	113243793	2.759606558	1.464463	0.000187	0.000876
RRM2B	113252861	2.753958932	1.461507	0.000329	0.001451
ELOB	113245416	2.753279535	1.461151	0.000686	0.002772
APPL2	113256053	2.750749363	1.459825	0.001015	0.003902
SNRNP25	113245172	2.749923821	1.459392	0.000121	0.000596
ZNF365	113259088	2.748556723	1.458674	0.0031	0.010285
LOC113242771	113242771	2.747428928	1.458082	0.004071	0.013003
ETV7	113243859	2.746515693	1.457603	0.018037	0.046076
RASD1	113271303	2.746160388	1.457416	0.001874	0.006645
ISPD	113260950	2.746087227	1.457377	0.004381	0.013849
LOC113247766	113247766	2.744715613	1.456657	0.004263	0.013538
NKAPL	113248162	2.744366229	1.456473	0.013088	0.035144
MRPS16	113259173	2.743139757	1.455828	0.000123	0.000601

GPRASP1	113244313	2.742820733	1.45566	0.014852	0.039145
ZNF25	113258958	2.741605915	1.455021	0.00039	0.001686
LOC113252203	113252203	2.740667593	1.454527	0.000285	0.001276
CD99L2	113246822	2.73968058	1.454008	0.002996	0.010008
CX3CL1	113252283	2.739317228	1.453816	0.007124	0.021028
TRIM3	113245858	2.737538772	1.452879	0.002452	0.008404
LOC113245502	113245502	2.737483985	1.452851	0.000184	0.000865
SEMA6A	113261619	2.734838206	1.451455	0.000199	0.000926
METAP1	113263122	2.733324154	1.450657	0.000323	0.001427
HABP4	113269773	2.728440448	1.448077	0.000495	0.002083
SMTN	113245049	2.7277959	1.447736	0.000224	0.001031
S100A13	113245338	2.724239579	1.445854	0.000407	0.001748
VMO1	113271087	2.723303807	1.445358	0.017753	0.045462
NAT14	113247427	2.722950948	1.445171	0.008305	0.023896
MARCH9	113256359	2.720545463	1.443896	0.000845	0.003327
DRG2	113271293	2.719660812	1.443427	0.000101	0.000504
LOC113250465	113250465	2.716306518	1.441646	0.000369	0.001602
PDLIM1	113251752	2.714350661	1.440607	0.001248	0.004676
ENPP5	113261237	2.713108734	1.439947	0.000356	0.001553
HUS1	113257638	2.710556982	1.438589	0.000861	0.003385
MLH3	113270322	2.710137689	1.438366	0.000737	0.002952
SH3BP4	113250314	2.707829001	1.437137	0.002206	0.007671
BANF1	113244083	2.704376322	1.435296	0.000347	0.001516
POLR2M	113269673	2.704156344	1.435179	0.000427	0.001819
LOC113244925	113244925	2.70369669	1.434933	0.000168	0.000797
PCYT1A	113252916	2.701390734	1.433702	0.000203	0.000942
LOC113246337	113246337	2.700568588	1.433263	0.001753	0.006275
NINJ2	113258034	2.700170122	1.43305	0.010105	0.028151

CIAO2B	113252339	2.700167581	1.433049	0.000227	0.001042
MOB2	113243948	2.696608749	1.431146	0.000262	0.001182
SELENOK	113256985	2.696058847	1.430852	0.000332	0.001459
CD59	113265058	2.6940546	1.429779	0.007293	0.021452
FER1L6	113252749	2.691273961	1.428289	0.008612	0.024644
ETFBKMT	113257750	2.690743813	1.428005	0.002052	0.007193
SYP	113265951	2.690571135	1.427912	0.006351	0.019124
STAC	113253765	3.205029479	1.680338	0.013498	0.036046
SLC25A42	113256741	3.203746704	1.67976	0.002398	0.008235
SQSTM1	113261555	3.20347816	1.679639	0.002571	0.008752
TCTA	113256877	3.203353044	1.679583	1.4E-05	8.55E-05
RITA1	113266990	3.202574242	1.679232	1.28E-05	7.86E-05
CDC37L1	113270165	3.198859087	1.677557	2.56E-06	1.82E-05
ISLR	113240961	3.197064578	1.676748	0.000753	0.00301
TSPAN3	113241005	3.193557791	1.675165	0.000507	0.002125
HES4	113270655	3.192903465	1.674869	4.17E-05	0.000226
L2HGDH	113266207	3.192701414	1.674778	4.56E-05	0.000246
SH3GL3	113263757	3.191801043	1.674371	0.001704	0.006125
CUNH16orf95	113252171	3.187520806	1.672435	0.010321	0.028684
NOV	113252778	3.18730676	1.672338	0.005888	0.017944
LOC113271076	113271076	3.183638048	1.670676	0.006671	0.019916
RTN4	113245629	3.183131295	1.670447	0.01305	0.035064
GPIHBP1	113247595	3.18062491	1.66931	0.0023	0.007948
ITGA3	113265609	3.18030223	1.669164	0.000486	0.002047
LOC113270384	113270384	3.180292033	1.669159	0.000119	0.000587
WSB2	113267016	3.180198765	1.669117	8.34E-05	0.000426
RUNDC3B	113259507	3.179494246	1.668797	4.68E-05	0.000252
DLGAP1	113253243	3.179394387	1.668752	0.002276	0.007879

LOC113247252	113247252	3.175718691	1.667083	0.003653	0.011857
AGMO	113260890	3.174381325	1.666475	0.006392	0.019223
TCEAL1	113249836	3.173393193	1.666026	4.06E-05	0.000221
LOC113246566	113246566	3.17200725	1.665396	5.81E-05	0.000307
LOC113249742	113249742	3.170074408	1.664517	3.01E-05	0.000169
CMC4	113248350	3.169996927	1.664481	0.000369	0.001602
SPARCL1	113263168	3.168479972	1.663791	0.00688	0.020393
CUNH8orf59	113252990	3.167856619	1.663507	2.91E-05	0.000164
HOXB8	113265579	3.166925327	1.663083	0.013044	0.035057
NRIP2	113258024	3.166214322	1.662759	0.004041	0.012929
RSL24D1	113269645	3.16592679	1.662628	7.27E-06	4.74E-05
N4BP2L2	113251246	3.165777312	1.66256	1.9E-05	0.000112
EPB41L4A	113254993	3.165122854	1.662261	2.78E-05	0.000158
PARP6	113240936	3.165115072	1.662258	7.75E-06	5.02E-05
CCDC127	113260770	3.164643826	1.662043	2.45E-05	0.00014
LOC113249961	113249961	3.16102192	1.660391	0.001924	0.006793
CRADD	113256160	3.15696846	1.65854	0.000787	0.003125
PPP1R3B	113262116	3.155693908	1.657957	0.001434	0.005279
SNX21	113258661	3.152923641	1.65669	0.000715	0.002875
CXXC5	113261811	3.149974377	1.65534	4.74E-06	3.21E-05
UBAP1L	113240866	3.148494603	1.654662	0.003127	0.010372
RTN4RL1	113268835	3.148352228	1.654597	0.006576	0.019686
PTPRM	113253224	3.147507696	1.65421	0.000406	0.001747
LOC113268544	113268544	3.145088594	1.653101	3.82E-05	0.00021
CCL21	113260498	3.143514349	1.652378	0.007823	0.022777
ZHX2	113252762	3.138849228	1.650236	0.000123	0.000601
FOXN3	113267374	3.136606716	1.649205	1.47E-05	8.91E-05
NUDT13	113259171	3.128458385	1.645452	0.000299	0.001334

LARGE1	113256015	3.127513933	1.645016	0.008572	0.024544
EMCN	113263107	3.124377145	1.643569	2.59E-05	0.000148
LOC113253436	113253436	3.123275119	1.64306	0.008909	0.025359
TMEM236	113241363	3.123263769	1.643054	0.001395	0.005153
SLC45A3	113242479	3.123023743	1.642944	0.001883	0.006671
ZNF385C	113265680	3.122282027	1.642601	0.005295	0.01637
ALKBH8	113260110	3.121870715	1.642411	0.001384	0.005121
PCDH19	113244289	3.115815013	1.63961	0.00244	0.008369
SLC18B1	113259462	3.114702259	1.639094	0.000133	0.000644
BOD1	113264110	3.113880542	1.638714	0.000369	0.001602
SBSPON	113253052	3.113493738	1.638534	0.004117	0.013133
NDUFA13	113256757	3.111234702	1.637487	0.000117	0.000578
NRBF2	113259094	3.10769837	1.635846	9.48E-05	0.000478
PRXL2B	113270607	3.105641184	1.634891	0.000725	0.002911
RNF7	113254010	3.104978476	1.634583	5.11E-05	0.000274
DNAJC28	113264408	3.10462868	1.634421	0.000128	0.000625
CAPN12	113243610	3.100449573	1.632477	0.004223	0.013424
CARD10	113241807	3.099355978	1.631968	0.000917	0.003567
LOC113249681	113249681	3.098280033	1.631468	0.001884	0.006674
VOPP1	113257409	3.097741038	1.631217	0.004617	0.014528
LIMD1	113256777	3.097736055	1.631214	3.14E-05	0.000175
RPL38	113265208	3.096636073	1.630702	0.001757	0.006281
YPEL1	113247045	3.093371988	1.62918	8.11E-05	0.000416
AR	113245650	3.092719756	1.628876	3.91E-05	0.000214
LOC113248605	113248605	3.090354863	1.627773	0.001261	0.004721
MAN2A2	113263716	3.089952192	1.627585	8.81E-05	0.000448
NCALD	113252864	3.089374358	1.627315	0.00385	0.012393
ZNF76	113243840	3.089250653	1.627257	2.22E-05	0.000129

COPS3	113271306	3.088961464	1.627122	2.2E-05	0.000128
VEGFA	113261040	3.088011465	1.626678	0.0001	0.000503
PDE7B	113259477	3.087595446	1.626484	1.6E-05	9.6E-05
TM4SF1	113254048	3.084033852	1.624819	0.000682	0.002763
TOMM7	113261301	3.08198868	1.623862	7.41E-05	0.000383
VEZT	113256140	3.081964155	1.62385	3.8E-05	0.000209
PBX1	113268685	3.08132823	1.623552	1.42E-05	8.63E-05
RHOBTB2	113269988	3.078871668	1.622402	6.48E-05	0.000339
ARHGAP29	113254388	3.076986041	1.621518	0.000246	0.00112
DMWD	113243413	3.075466176	1.620805	0.000118	0.00058
LOC113261640	113261640	3.074625005	1.62041	0.003889	0.012499
AMT	113256872	3.07061378	1.618527	0.000723	0.002904
LOC113257493	113257493	3.070480985	1.618465	0.005897	0.017965
SLIRP	113270282	3.070346893	1.618402	8.82E-05	0.000448
TMLHE	113248335	3.066465787	1.616577	0.000237	0.001082
GUCY1A2	113260215	3.064568545	1.615684	0.004317	0.013677
PLB1	113267984	3.061448905	1.614215	0.011304	0.030966
LOC113243636	113243636	3.059412963	1.613255	5.29E-05	0.000282
NDUFA7	113242904	3.058628647	1.612885	3.51E-05	0.000194
TMED1	113243002	3.057609485	1.612404	7.91E-05	0.000406
LYRM1	113267537	3.056264922	1.61177	0.003377	0.011077
ST3GAL3	113254779	3.055638811	1.611474	1.61E-05	9.67E-05
SCMH1	113254820	3.053364966	1.6104	0.000228	0.001045
USP25	113244784	3.051220059	1.609386	2E-05	0.000117
SORCS2	113245719	3.049896892	1.60876	0.001735	0.006222
GNG7	113242789	3.049512409	1.608579	0.00028	0.001257
MAP4K3	113241667	3.04813507	1.607927	7.15E-06	4.67E-05
PDGFB	113241764	3.047850226	1.607792	0.001582	0.005755

TRIAP1	113267045	3.047335792	1.607548	2.77E-05	0.000157
MTFR1L	113268482	3.047187335	1.607478	5.44E-05	0.000289
FSCN2	113244518	3.046949897	1.607366	0.004356	0.013784
CLDN5	113245974	3.042604861	1.605307	0.001608	0.005828
PSMG3	113247741	3.040650957	1.60438	5.3E-05	0.000282
OTUD4	113255723	3.04010752	1.604122	5.45E-05	0.000289
HADHA	113270772	3.037818145	1.603036	0.005896	0.017965
ARHGAP20	113260078	3.036761746	1.602534	0.00105	0.004018
CMC1	113253855	3.03418237	1.601308	0.000117	0.000579
FOXP3	113265955	3.034167155	1.601301	0.002717	0.009185
GABBR2	113260410	3.033035157	1.600762	0.004718	0.014813
SPATA25	113258663	3.031651749	1.600104	0.004054	0.012959
ISCU	113267071	3.031013367	1.5998	0.001177	0.004438
PARP3	113256932	3.026914092	1.597848	0.01965	0.049571
IL17RD	113256999	3.026326726	1.597568	0.005218	0.016178
KCNJ8	113257805	3.026287878	1.597549	0.003619	0.011776
EFCAB2	113262948	3.021602339	1.595314	7.31E-05	0.000379
MEOX2	113260901	3.02123643	1.595139	0.002503	0.008555
HS3ST2	113267566	3.021017279	1.595034	0.001918	0.006776
LOC113247899	113247899	3.01898878	1.594065	0.000404	0.001738
MVB12B	113266416	3.017825097	1.593509	3.04E-05	0.00017
CPT2	113254653	3.010955135	1.590221	0.000871	0.003415
TNFRSF9	113270556	3.0090342	1.589301	0.000596	0.002449
PATL1	113248883	3.007148392	1.588396	3.04E-05	0.00017
TMEM8B	113260461	3.007100132	1.588373	0.000592	0.002438
SGCG	113251183	3.007094587	1.58837	0.015773	0.041262
SLC7A6	113252404	3.007052225	1.58835	7.14E-05	0.00037
SHISA8	113241716	3.006590546	1.588128	0.011706	0.031939

N6AMT1	113268381	3.004651186	1.587198	0.000104	0.000518
TMEM147	113244830	3.003625296	1.586705	0.00049	0.002062
KCNJ16	113265225	3.002202817	1.586021	0.005876	0.01792
PEX5	113257939	3.001294996	1.585585	7.73E-05	0.000397
SDHAF3	113260440	3.000598165	1.58525	0.000521	0.002176
MRPL19	113243374	2.997723905	1.583868	0.000378	0.001639
IMPA2	113245922	2.997096942	1.583566	3.14E-05	0.000175
ANGPT4	113258433	2.996431864	1.583246	0.000148	0.000711
RGS11	113245366	2.993369447	1.58177	0.003204	0.010593
RPL30	113252895	2.99263702	1.581417	0.019658	0.049573
YAP1	113259721	2.992505241	1.581354	0.000161	0.000769
SELENOW	113243361	2.990183274	1.580234	5.33E-05	0.000284
PTOV1	113243281	2.989402743	1.579857	0.000122	0.000599
MNAT1	113242407	2.98846362	1.579404	4.73E-05	0.000255
DMAC2	113243534	2.985978266	1.578204	4.45E-05	0.000241
SNTA1	113258493	2.978898336	1.574779	0.007595	0.02222
LHFPL1	113241246	2.977397011	1.574052	0.004399	0.013897
ESAM	113259915	2.973636336	1.572228	0.000103	0.000513
CREB5	113262153	2.971958151	1.571414	0.002347	0.008081
NDUFV3	113254594	2.97096868	1.570933	6.08E-05	0.00032
ZNF532	113253462	2.965553097	1.568301	2E-05	0.000117
NDST1	113263951	2.965256413	1.568157	3.91E-05	0.000214
LOC113248316	113248316	2.960585158	1.565882	0.000394	0.001698
TRMT10C	113250698	2.959536003	1.565371	0.000251	0.001138
DNAJC21	113260878	2.958566218	1.564898	2.44E-05	0.00014
MRS2	113264337	2.958544357	1.564888	0.001212	0.004558
LOC113242682	113242682	2.957615628	1.564435	0.004298	0.013627
SMIM11A	113262792	2.956998962	1.564134	8.48E-05	0.000432

HEY1	113253020	2.954742827	1.563033	0.000919	0.003576
USP24	113254622	2.954093942	1.562716	0.000761	0.003036
SESN1	113264757	2.950433559	1.560927	0.000187	0.000879
MBNL2	113251479	2.950423602	1.560922	0.004415	0.013945
AFDN	113247099	2.94858014	1.56002	0.000191	0.000892
NOSTRIN	113250810	2.947519223	1.559501	0.009267	0.026214
KEAP1	113242989	2.947263427	1.559376	0.000882	0.003448
HYKK	113263805	2.945437606	1.558482	0.003228	0.010653
SLC3A1	113246247	2.944148651	1.557851	0.000513	0.002148
FTO	113252242	2.9435233	1.557544	3.28E-05	0.000183
SPRYD7	113251311	2.94269936	1.55714	0.0003	0.001339
DNPEP	113250427	2.941216489	1.556413	9.72E-05	0.000489
GARNL3	113266425	2.940251845	1.55594	0.008053	0.023333
MITF	113257074	2.939075273	1.555362	0.000393	0.001694
HSPA12B	113258381	2.936236129	1.553968	0.000656	0.002668
GFRA1	113251969	2.935761494	1.553735	0.00528	0.016329
DNAJA4	113263798	2.93492034	1.553321	9.03E-05	0.000457
L3MBTL3	113259438	2.934605628	1.553167	8E-05	0.00041
ATP5PO	113263260	2.93403816	1.552888	0.004122	0.013145
LARP1B	113255656	2.931840068	1.551806	3.52E-05	0.000195
CHCHD1	113259188	2.927282865	1.549562	0.000169	0.000801
KYAT1	113266490	2.923363805	1.547629	0.005628	0.017245
LOC113242946	113242946	2.919496922	1.54572	0.011755	0.032052
KLHDC8B	113256866	2.919411931	1.545678	0.00731	0.02149
CCDC68	113253439	2.918585813	1.545269	0.003667	0.011892
LRRC3B	113253861	2.918402612	1.545179	0.003737	0.012089
THRA	113265499	2.918082629	1.545021	0.00027	0.001217
BTBD6	113267181	2.917702393	1.544833	0.001323	0.004922

LRRC4	113263735	2.917198147	1.544583	0.001806	0.006441
PHLDB2	113251155	2.913497426	1.542752	0.000246	0.001119
LOC113241998	113241998	2.913488195	1.542747	0.000583	0.002408
ADAMTSL1	113260599	2.911099064	1.541564	0.001197	0.004504
MTIF3	113251204	2.910563068	1.541298	7.93E-05	0.000406
USHBP1	113256668	2.910311631	1.541174	0.001122	0.004256
SMIM10L1	113257883	2.907503736	1.539781	9.69E-05	0.000488
PPP2R2D	113252071	2.903817087	1.537951	0.000311	0.001381
ZNF583	113248544	2.900189723	1.536147	0.002551	0.008693
TLE2	113242769	2.898648816	1.535381	0.000139	0.000671
NAXE	113245227	2.8983189	1.535216	0.000183	0.000864
TMEM126A	113269391	2.896920576	1.53452	0.000331	0.001457
LOC113245455	113245455	2.895314031	1.53372	2.87E-05	0.000162
DUSP10	113268713	2.894576679	1.533352	0.000147	0.000708
ASPSCR1	113244489	2.894098945	1.533114	0.001354	0.005019
MTMR11	113246122	2.89183422	1.531985	0.007635	0.022299
AES	113242788	2.890980216	1.531559	0.001257	0.004707
LOC113265650	113265650	2.889994747	1.531067	0.000199	0.000926
APBB1	113245850	2.888588584	1.530365	0.000398	0.001717
SDHC	113246688	2.888194773	1.530168	0.00285	0.00957
KIF21A	113257734	2.887569514	1.529856	0.001807	0.006444
VPS9D1	113252123	2.887557279	1.52985	0.000117	0.000579
LOC113249791	113249791	2.88730854	1.529725	0.004843	0.015169
FST	113263911	2.884834234	1.528488	0.008711	0.02488
USP11	113265969	2.883876225	1.528009	0.000546	0.002268
OSBPL1A	113253295	2.883243946	1.527693	0.001308	0.004874
KDM4D	113260162	2.88169687	1.526919	0.006911	0.020475
PVR	113243460	2.879585564	1.525861	0.001149	0.00435

SLC47A2	113271269	2.8791563	1.525646	0.008818	0.025143
PSMG4	113264402	2.877491927	1.524812	0.003848	0.012393
PDCD5	113244718	2.877340909	1.524736	0.000266	0.001198
CLYBL	113251501	2.877097215	1.524614	0.000728	0.002919
THNSL2	113242812	2.875942421	1.524035	0.00016	0.000763
FRS3	113261310	2.875317562	1.523721	0.000592	0.002436
CPED1	113263459	2.875250734	1.523688	0.001445	0.005315
LOC113245523	113245523	2.874889968	1.523507	0.000565	0.002337
LOC113248542	113248542	2.872694052	1.522404	0.001833	0.006522
USH1C	113246620	2.870756202	1.521431	0.008655	0.024745
DUSP23	113246655	2.869531271	1.520815	0.000873	0.003419
ACP2	113264970	2.869230846	1.520664	0.000158	0.000755
KAT5	113244098	2.868804899	1.52045	0.000119	0.000584
ZFP92	113247841	2.867292399	1.519689	0.008112	0.023467
PYROXD2	113251809	2.866435352	1.519258	0.005347	0.016519
RTL10	113245950	2.866365346	1.519223	0.001351	0.005013
GATA6	113253276	2.864077732	1.518071	0.012175	0.033026
USP53	113255610	2.862461563	1.517256	0.000126	0.000618
MAB21L1	113251254	2.8597858	1.515907	0.009872	0.027617
DENND1B	113242587	2.859505159	1.515766	0.001617	0.005857
LRIG1	113257056	2.858371338	1.515193	0.00987	0.027617
DEAF1	113248806	2.857667686	1.514838	8.56E-05	0.000436
PRMT1	113243287	2.856683873	1.514341	9.24E-05	0.000466
RFK	113269838	2.855092535	1.513538	0.000113	0.000559
ATP5IF1	113268421	2.852917366	1.512438	0.002292	0.007929
ZNF662	113253831	2.849905462	1.510914	0.001017	0.00391
IARS2	113268724	2.849463381	1.51069	0.000836	0.0033
SERTAD4	113262655	2.848330683	1.510117	0.019824	0.049863

SMYD2	113262691	2.848065215	1.509982	8.94E-05	0.000453
LOC113248302	113248302	2.845539536	1.508702	0.000848	0.00334
STUB1	113245397	2.844908975	1.508382	0.000667	0.002704
PACS2	113267179	2.839461873	1.505618	0.000345	0.001511
UBXN1	113246911	2.838427217	1.505092	0.000201	0.000936
THAP12	113269339	2.838329417	1.505042	0.000511	0.002142
RAB40B	113244465	2.83753057	1.504636	0.005138	0.015963
ABLIM3	113263929	2.833253489	1.50246	0.008185	0.023616
EIF4E3	113257082	2.831513015	1.501573	0.000257	0.001164
PALM	113242702	2.831354661	1.501492	0.004201	0.013364
HMGCLL1	113261167	2.830764188	1.501192	0.009027	0.025627
CXCL14	113261492	2.826119282	1.498822	0.0006	0.002464
FOXO4	113245656	2.825672457	1.498594	9.44E-05	0.000476
NUDT6	113255639	2.823321547	1.497393	0.000174	0.000823
SLC43A1	113246534	2.819761773	1.495573	7.82E-05	0.000401
SAR1B	113261745	2.817270929	1.494298	0.001116	0.004234
TIMM13	113242767	2.816740185	1.494026	0.000191	0.000894
COQ3	113264805	2.816310879	1.493807	0.000366	0.001594
CFAP58	113251623	2.813905165	1.492574	0.003322	0.010932
NOD1	113262332	2.81337358	1.492301	5.25E-05	0.000281
POLR1E	113260444	2.81193223	1.491562	6.06E-05	0.00032
LOC113246836	113246836	2.811013111	1.49109	0.007408	0.021724
ITGB8	113261164	2.810524774	1.49084	0.007735	0.022547
FBXL17	113255014	2.806189301	1.488612	4.15E-05	0.000225
NKIRAS1	113253873	2.804711617	1.487852	0.001512	0.005535
OPN1SW	113263870	2.804583554	1.487787	0.008516	0.024405
FKRP	113243391	2.802230364	1.486576	6.49E-05	0.000339
LURAP1	113254728	2.801988129	1.486451	0.005938	0.018069

RPS28	113242905	2.801091137	1.485989	0.008745	0.024959
DCXR	113244486	2.800603058	1.485738	0.000935	0.003633
GCSH	113252527	2.800183062	1.485521	0.003667	0.011892
LRPPRC	113246283	2.800156925	1.485508	0.003626	0.011793
FIBIN	113265095	2.79973605	1.485291	0.0028	0.009438
CCSER2	113259236	2.799554721	1.485197	0.000737	0.002953
PSEN2	113262931	2.795198781	1.482951	5.56E-05	0.000295
NFIB	113260613	2.795157034	1.482929	0.003377	0.011077
EIF2A	113254058	2.794226593	1.482449	0.000892	0.003486
HTR1B	113261102	2.793797258	1.482227	0.018017	0.046033
SHANK3	113241594	2.79354764	1.482098	0.000203	0.000941
SLC39A2	113248487	2.790749426	1.480653	0.006009	0.018262
MARS2	113250623	2.789786053	1.480154	0.000561	0.002323
STARD7	113241969	2.789785696	1.480154	0.002021	0.007094
LSM14B	113258785	2.789705375	1.480113	2.93E-05	0.000165
TIMM8B	113260055	2.788768646	1.479628	0.000281	0.001259
PGP	113245113	2.787390235	1.478915	0.000244	0.001112
SYNGR2	113244572	2.786576912	1.478494	0.000808	0.003198
SOX8	113245394	2.786111226	1.478253	0.005526	0.016982
RDH5	113256460	2.786091712	1.478243	0.006679	0.019937
ZCCHC24	113259220	2.785344893	1.477856	0.000496	0.002083
MTUS1	113262156	2.785128402	1.477744	0.007893	0.022948
MRPL34	113256673	2.776683984	1.473363	0.000734	0.002944
PSMC3	113264959	2.776673875	1.473358	0.000691	0.002791
CUNH11orf94	113264998	2.775642318	1.472822	0.012065	0.032766
PPP1R14B	113244179	2.77503988	1.472509	0.000957	0.003706
MRPL11	113244065	2.774259528	1.472103	0.000456	0.001929
ZNHIT2	113244132	2.773840008	1.471885	0.000182	0.000858

ABCC2	113251819	2.773251518	1.471578	0.005678	0.017384
ARHGEF15	113271183	2.772692735	1.471288	0.000658	0.002674
AKT1S1	113243278	2.770359677	1.470073	0.00129	0.004812
LOC113257828	113257828	2.769482881	1.469617	0.000444	0.001884
SNRPN	113263595	2.767657817	1.468666	0.002025	0.007106
KLF2	113256645	2.766123411	1.467866	0.000154	0.000738
ACVR2A	113250906	2.765061207	1.467311	0.001976	0.006956
LOC113249995	113249995	2.764735173	1.467141	0.004745	0.014883
ANKRD53	113244188	2.763558236	1.466527	0.00486	0.015196
NTAN1	113270768	2.76154135	1.465474	0.000363	0.001578
TMEM94	113244634	2.760900033	1.465139	5.41E-05	0.000287
GATC	113267042	2.760692971	1.46503	0.000374	0.001624
HSD17B8	113243793	2.759606558	1.464463	0.000187	0.000876
RRM2B	113252861	2.753958932	1.461507	0.000329	0.001451
ELOB	113245416	2.753279535	1.461151	0.000686	0.002772
APPL2	113256053	2.750749363	1.459825	0.001015	0.003902
SNRNP25	113245172	2.749923821	1.459392	0.000121	0.000596
ZNF365	113259088	2.748556723	1.458674	0.0031	0.010285
LOC113242771	113242771	2.747428928	1.458082	0.004071	0.013003
ETV7	113243859	2.746515693	1.457603	0.018037	0.046076
RASD1	113271303	2.746160388	1.457416	0.001874	0.006645
ISPD	113260950	2.746087227	1.457377	0.004381	0.013849
LOC113247766	113247766	2.744715613	1.456657	0.004263	0.013538
NKAPL	113248162	2.744366229	1.456473	0.013088	0.035144
MRPS16	113259173	2.743139757	1.455828	0.000123	0.000601
GPRASP1	113244313	2.742820733	1.45566	0.014852	0.039145
ZNF25	113258958	2.741605915	1.455021	0.00039	0.001686
LOC113252203	113252203	2.740667593	1.454527	0.000285	0.001276

CD99L2	113246822	2.73968058	1.454008	0.002996	0.010008
CX3CL1	113252283	2.739317228	1.453816	0.007124	0.021028
TRIM3	113245858	2.737538772	1.452879	0.002452	0.008404
LOC113245502	113245502	2.737483985	1.452851	0.000184	0.000865
SEMA6A	113261619	2.734838206	1.451455	0.000199	0.000926
METAP1	113263122	2.733324154	1.450657	0.000323	0.001427
HABP4	113269773	2.728440448	1.448077	0.000495	0.002083
SMTN	113245049	2.7277959	1.447736	0.000224	0.001031
S100A13	113245338	2.724239579	1.445854	0.000407	0.001748
VMO1	113271087	2.723303807	1.445358	0.017753	0.045462
NAT14	113247427	2.722950948	1.445171	0.008305	0.023896
MARCH9	113256359	2.720545463	1.443896	0.000845	0.003327
DRG2	113271293	2.719660812	1.443427	0.000101	0.000504
LOC113250465	113250465	2.716306518	1.441646	0.000369	0.001602
PDLIM1	113251752	2.714350661	1.440607	0.001248	0.004676
ENPP5	113261237	2.713108734	1.439947	0.000356	0.001553
HUS1	113257638	2.710556982	1.438589	0.000861	0.003385
MLH3	113270322	2.710137689	1.438366	0.000737	0.002952
SH3BP4	113250314	2.707829001	1.437137	0.002206	0.007671
BANF1	113244083	2.704376322	1.435296	0.000347	0.001516
POLR2M	113269673	2.704156344	1.435179	0.000427	0.001819
LOC113244925	113244925	2.70369669	1.434933	0.000168	0.000797
PCYT1A	113252916	2.701390734	1.433702	0.000203	0.000942
LOC113246337	113246337	2.700568588	1.433263	0.001753	0.006275
NINJ2	113258034	2.700170122	1.43305	0.010105	0.028151
CIAO2B	113252339	2.700167581	1.433049	0.000227	0.001042
MOB2	113243948	2.696608749	1.431146	0.000262	0.001182
SELENOK	113256985	2.696058847	1.430852	0.000332	0.001459

CD59	113265058	2.6940546	1.429779	0.007293	0.021452
FER1L6	113252749	2.691273961	1.428289	0.008612	0.024644
ETFBKMT	113257750	2.690743813	1.428005	0.002052	0.007193
SYP	113265951	2.690571135	1.427912	0.006351	0.019124
ADGRF5	113261229	2.690265488	1.427749	0.009159	0.025949
POMGNT2	113253834	2.688880252	1.427006	0.013858	0.036857
ZADH2	113253530	2.688598982	1.426855	0.000411	0.00176
MRPS18C	113263198	2.688381423	1.426738	0.000259	0.001171
OXSM	113253864	2.687339432	1.426179	0.000595	0.002449
SLC25A25	113266451	2.684895286	1.424866	0.001313	0.004892
DLAT	113260048	2.684746162	1.424786	0.00604	0.018341
IKZF4	113256443	2.684239857	1.424514	0.000736	0.002952
LOC113261535	113261535	2.683102833	1.423902	0.00159	0.005779
GJA5	113247790	2.681869164	1.423239	0.001486	0.00545
ARHGEF25	113256370	2.681421871	1.422998	0.0071	0.020977
WDR77	113244430	2.680997402	1.42277	0.000306	0.001363
GADD45G	113270128	2.679192922	1.421798	0.00133	0.004939
EEF2K	113267554	2.678286548	1.42131	0.000217	0.001002
ETS2	113258013	2.6782852	1.42131	0.000166	0.000791
MTHFR	113270501	2.675957982	1.420055	0.000304	0.001353
LOC113251149	113251149	2.675435402	1.419774	0.01477	0.038954
DCAF8	113246657	2.674621904	1.419335	0.002685	0.009097
HSD17B1	113265419	2.672667376	1.41828	0.006869	0.020374
ECHS1	113252096	2.672265184	1.418063	0.005707	0.017459
TIMMDC1	113251876	2.670442157	1.417079	0.000789	0.003131
ARAF	113265980	2.669560783	1.416602	0.000345	0.00151
ZAN	113267522	2.668748636	1.416163	0.007909	0.022982
ZNF846	113242959	2.668407801	1.415979	0.012227	0.033127

CUNH2orf92	113246726	2.668304856	1.415924	0.008497	0.024358
ANKRD33	113257597	2.667899432	1.415704	0.010456	0.028983
ZNF485	113243138	2.667578834	1.415531	0.00499	0.015564
DBP	113243343	2.666944995	1.415188	0.003776	0.012205
CUNH19orf54	113243556	2.666436593	1.414913	0.002747	0.009279
TOM1L2	113271297	2.666237522	1.414805	0.00025	0.001137
LOC113245461	113245461	2.664981594	1.414126	0.002314	0.007989
TPPP	113260777	2.663682692	1.413422	0.004333	0.013718
CAPZA2	113263223	2.663376944	1.413257	0.001727	0.006193
AVPR2	113247864	2.662167697	1.412601	0.017666	0.045297
DYNLL2	113268866	2.659436383	1.411121	0.000945	0.003666
STRN3	113266095	2.658935892	1.410849	0.000176	0.000833
EXOSC9	113255628	2.658911862	1.410836	0.000205	0.000953
LGR4	113265090	2.658684374	1.410713	0.000457	0.00193
UBE2E3	113250703	2.658364414	1.410539	0.000183	0.000864
CASKIN2	113244635	2.657993432	1.410338	0.000341	0.001495
HOXA9	113262053	2.657003399	1.4098	0.000771	0.003071
CILP	113240867	2.655169709	1.408804	0.014119	0.037475
MET	113263211	2.649262943	1.405591	0.005247	0.016251
DNAJA3	113270848	2.648236619	1.405032	0.000409	0.001754
CCDC121	113271301	2.646730635	1.404211	0.001098	0.004174
LOC113248539	113248539	2.646360422	1.40401	0.000385	0.001666
ITGA7	113256462	2.645579995	1.403584	0.016156	0.042037
RUFY3	113263301	2.645358128	1.403463	0.000282	0.001266
KIAA1107	113254412	2.643513275	1.402457	0.000533	0.002217
ZNF134	113247987	2.640180258	1.400636	0.000225	0.001034
ACYP1	113270321	2.640123852	1.400606	0.001947	0.006864
ARHGEF7	113248529	2.635871142	1.39828	0.000365	0.001587

PGAM5	113266837	2.634784311	1.397685	0.000139	0.000673
FAM135A	113261136	2.632566089	1.39647	0.000527	0.002196
CDH15	113252138	2.629044551	1.394539	0.01354	0.036128
LOC113253631	113253631	2.628706105	1.394353	0.009331	0.026357
POLM	113257880	2.627902792	1.393912	0.009514	0.026792
EPHX4	113254413	2.626481904	1.393132	0.017802	0.04556
LOC113262545	113262545	2.626332169	1.393049	0.000514	0.002149
ATP6V1FNB	113263980	2.625485498	1.392584	0.011994	0.032626
POLR3C	113247755	2.62503039	1.392334	0.000749	0.002994
SDHAF1	113244838	2.624640219	1.39212	0.002306	0.007965
SLX4	113270863	2.622966178	1.391199	0.000533	0.002217
RABEP1	113271062	2.621750844	1.390531	0.000227	0.001042
TESK1	113260478	2.620448478	1.389814	0.000426	0.001818
ISLR2	113240958	2.618798424	1.388905	0.007874	0.022909
ECSCR	113261806	2.618048231	1.388492	0.002695	0.009128
CUNH16orf89	113270825	2.617842651	1.388378	0.019062	0.048323
ARHGEF28	113255216	2.615795411	1.38725	0.007231	0.021293
ST8SIA5	113253380	2.615175501	1.386908	0.002025	0.007106
EMC9	113246088	2.614557513	1.386567	0.00092	0.003577
SETD3	113267284	2.611390372	1.384818	0.000328	0.001449
DIPK2B	113265908	2.611369114	1.384806	0.003603	0.011731
UACA	113240926	2.61014728	1.384131	0.000617	0.002527
NELL2	113257712	2.609464431	1.383754	0.01239	0.033516
LNX1	113262494	2.608234369	1.383074	0.000426	0.001818
NDUFAF1	113242009	2.607483368	1.382658	0.001123	0.00426
ZC2HC1A	113253024	2.607145311	1.382471	0.007711	0.022488
BCKDK	113267717	2.60631585	1.382012	0.0009	0.003513
ALS2	113250585	2.602743323	1.380033	0.000497	0.002087

RHOD	113244042	2.599590842	1.378285	0.009806	0.027476
MRPL36	113260792	2.599500271	1.378234	0.001909	0.006751
LOC113270508	113270508	2.599294154	1.37812	0.013765	0.036622
GPT	113247624	2.598829924	1.377862	0.006454	0.019389
FRY	113251237	2.598054688	1.377432	0.000553	0.002292
VTN	113269030	2.597344376	1.377037	0.002075	0.00726
DIXDC1	113260059	2.597285198	1.377004	0.0019	0.006725
ZNF142	113250451	2.596902833	1.376792	0.001026	0.00394
PDE4A	113242988	2.596365837	1.376494	0.00323	0.010657
MESP1	113263697	2.594481606	1.375446	0.010024	0.027955
LOC113254720	113254720	2.594398478	1.3754	0.005026	0.015657
AGPAT5	113262188	2.592372217	1.374273	0.000608	0.002494
MRPS35	113257772	2.591817118	1.373964	0.001619	0.00586
MB21D2	113253491	2.588293952	1.372001	0.015779	0.04127
MSRB2	113241336	2.587988228	1.371831	0.005347	0.016519
ALDH1A3	113263632	2.585569228	1.370482	0.001044	0.003995
AP4S1	113266097	2.584333853	1.369792	0.002109	0.00737
ABI2	113250562	2.580843651	1.367843	0.000855	0.003365
PLCE1	113243183	2.580535756	1.367671	0.001177	0.004438
CCDC85B	113244087	2.580446554	1.367621	0.000491	0.002066
HYPK	113241956	2.577598414	1.366028	0.000408	0.001753
BRMS1L	113266134	2.577378006	1.365904	0.000739	0.002959
CUL3	113250393	2.575699561	1.364964	0.001435	0.005281
DLST	113270325	2.571926828	1.36285	0.005233	0.016214
INKA2	113244426	2.568761128	1.361073	0.011983	0.03261
B3GALT4	113243802	2.568661206	1.361017	0.008077	0.023384
TJP1	113263619	2.568406447	1.360874	0.001427	0.005259
LY6G5B	113243725	2.567988267	1.360639	0.001065	0.004066

ZNF484	113270082	2.566855256	1.360002	0.008425	0.02419
LOC113249794	113249794	2.566120931	1.359589	0.008919	0.02537
SLCO3A1	113263665	2.564288938	1.358559	0.00038	0.001646
SMARCA1	113270938	2.560598376	1.356481	0.001567	0.005704
KCMF1	113243249	2.556285618	1.354049	0.0004	0.001725
JCAD	113244246	2.556223685	1.354014	0.00101	0.003885
CFAP46	113252084	2.555771158	1.353759	0.01239	0.033516
SRF	113261274	2.555692587	1.353714	0.000444	0.001883
SMDT1	113241709	2.554495876	1.353039	0.000389	0.00168
TRAPPC6B	113266154	2.553601676	1.352534	0.000318	0.001408
PCSK4	113242736	2.553594108	1.352529	0.001901	0.006726
ZNF10	113266827	2.552878771	1.352125	0.000379	0.001642
ZSWIM5	113254749	2.552834404	1.3521	0.016366	0.042503
FAM110D	113268468	2.551348442	1.35126	0.007649	0.022332
JTB	113245325	2.550925395	1.351021	0.002988	0.00999
LOC113241121	113241121	2.547009178	1.348804	0.000894	0.003492
MCC	113254984	2.546974728	1.348785	0.001314	0.004894
LOC113248518	113248518	2.545460399	1.347927	0.000529	0.002202
S100A16	113245341	2.545048521	1.347693	0.001127	0.004275
GMPR	113264491	2.544958181	1.347642	0.012094	0.032825
IFT81	113266949	2.543255085	1.346676	0.002717	0.009185
ETNK2	113242508	2.542797722	1.346417	0.013329	0.035665
ARL6IP5	113257071	2.542110543	1.346027	0.005813	0.017745
EPHX2	113269956	2.541894927	1.345904	0.006296	0.01899
GFM1	113254120	2.541724351	1.345808	0.0011	0.004182
UHRF1BP1	113243830	2.540707032	1.34523	0.000383	0.001658
NDUFA10	113249793	2.540112824	1.344893	0.001669	0.006019
NTMT1	113266511	2.538504436	1.343979	0.000901	0.003514

ANKRD40	113265631	2.536947305	1.343094	0.000913	0.003556
LOC113248398	113248398	2.536369415	1.342765	0.000682	0.002763
ZFP62	113261523	2.535671283	1.342368	0.000523	0.002186
RNF31	113245982	2.533927965	1.341376	0.000913	0.003556
FDX1	113260080	2.531426975	1.339951	0.002928	0.00981
DGKE	113268844	2.531243739	1.339846	0.000172	0.000814
MRPL47	113254740	2.529614082	1.338917	0.001161	0.004386
FNIP1	113261697	2.529316779	1.338748	0.000784	0.003116
DPP8	113240872	2.529013186	1.338575	0.000726	0.002915
SOCS1	113270798	2.52891443	1.338518	0.008912	0.025359
SAT2	113271158	2.52666257	1.337233	0.01278	0.034448
NMD3	113254147	2.525918022	1.336808	0.000952	0.003692
CHDH	113256982	2.525428158	1.336528	0.000577	0.002383
OLA1	113250762	2.524761185	1.336147	0.001059	0.004045
FGD5	113257212	2.52301624	1.335149	0.000458	0.001936
SLC10A6	113263176	2.521967215	1.33455	0.017654	0.045275
ELOC	113253044	2.521738037	1.334418	0.000389	0.001681
CBY3	113261946	2.52129906	1.334167	0.009363	0.026422
SLC25A26	113257057	2.520937543	1.33396	0.002014	0.007079
FXN	113270198	2.519386232	1.333072	0.000772	0.003074
IDS	113241894	2.518136924	1.332357	0.002478	0.008478
NOP10	113242084	2.517681806	1.332096	0.001067	0.004071
LRRC42	113254642	2.516731398	1.331551	0.001115	0.004231
KLF4	113260343	2.516066125	1.33117	0.000632	0.002581
APOA1	113260022	2.515538139	1.330867	0.005535	0.017003
UCHL5	113268752	2.51520086	1.330674	0.000368	0.0016
GLO1	113243888	2.514142037	1.330066	0.008134	0.023516
TMTC2	113256210	2.51253682	1.329145	0.006567	0.019665

EIF3K	113243612	2.51180052	1.328722	0.00604	0.018341
RPP21	113264371	2.51027319	1.327844	0.007681	0.02241
TRMT13	113254356	2.510180153	1.327791	0.00035	0.001528
CEP68	113244834	2.510158687	1.327779	0.000418	0.001787
PAIP2	113261802	2.509717567	1.327525	0.003978	0.012746
BAIAP2	113244528	2.508585827	1.326874	0.001779	0.006352
LOC113249956	113249956	2.507610451	1.326313	0.01438	0.038056
KLF9	113270210	2.505981803	1.325376	0.002053	0.007195
PIGX	113242811	2.505036668	1.324832	0.004317	0.013677
FAM43A	113253342	2.50088316	1.322438	0.000832	0.003284
SERTAD1	113243566	2.499547669	1.321667	0.000872	0.003418
MKKS	113258338	2.499324954	1.321538	0.00081	0.003204
OGFOD1	113252259	2.497405862	1.32043	0.002884	0.009671
LOC113248766	113248766	2.493641841	1.318254	0.001196	0.004504
KATNAL2	113253383	2.493209392	1.318004	0.016677	0.043137
PMPCB	113258960	2.493049726	1.317912	0.001944	0.006857
TANGO2	113245938	2.492634274	1.317671	0.00096	0.003716
CIAO3	113245391	2.492292998	1.317474	0.00043	0.001832
SUMO3	113251043	2.490690722	1.316546	0.000273	0.001226
TOB2	113241727	2.490378116	1.316365	0.000408	0.001753
MOAP1	113267336	2.489164897	1.315662	0.007621	0.022263
PNPO	113265563	2.488623575	1.315348	0.000961	0.003718
DNAJB9	113262843	2.488211176	1.315109	0.000646	0.002629
PRPF40B	113257645	2.487240886	1.314546	0.001298	0.00484
OGDH	113257822	2.484130236	1.312741	0.010344	0.028742
LOC113248597	113248597	2.481723117	1.311342	0.001677	0.006042
AUTS2	113267983	2.481319769	1.311108	0.001534	0.005603
QKI	113259634	2.481300497	1.311096	0.006313	0.019028

SMIM27	113260541	2.480514239	1.310639	0.001882	0.006669
DNTTIP1	113258656	2.478909768	1.309706	0.000476	0.002008
FHOD1	113252346	2.478776634	1.309628	0.001221	0.004586
NELL1	113265109	2.477810724	1.309066	0.015255	0.040052
YY2	113242235	2.476952383	1.308566	0.012592	0.033966
ZNF862	113241422	2.4763217	1.308199	0.00151	0.005528
LRRC32	113269342	2.476311241	1.308193	0.01299	0.034942
ZNF835	113248545	2.475315631	1.307612	0.011262	0.030869
ENO2	113257953	2.474773178	1.307296	0.008973	0.025501
PDF	113252427	2.471317643	1.30528	0.003142	0.010412
LOC113250723	113250723	2.47124946	1.305241	0.017233	0.044364
MAF	113251743	2.471107447	1.305158	0.000684	0.002766
AVEN	113242077	2.470527306	1.304819	0.003683	0.011935
EPN1	113247373	2.470119841	1.304581	0.003852	0.012395
ATF4	113241758	2.468423568	1.30359	0.015985	0.041689
COPS5	113268036	2.466822059	1.302654	0.000839	0.003308
ZBTB44	113259859	2.46551767	1.301891	0.001473	0.005408
BMPR1A	113259247	2.464702442	1.301413	0.001196	0.004504
ZNF496	113247501	2.460577141	1.298997	0.000527	0.002196
PDCL3	113247123	2.459849146	1.29857	0.000821	0.003247
EFNB2	113251527	2.459752368	1.298513	0.000916	0.003566
LOC113250195	113250195	2.457652289	1.297281	0.00452	0.014237
LOC113247187	113247187	2.45743026	1.29715	0.018086	0.046176
SNCAIP	113261642	2.456513978	1.296612	0.002609	0.008856
SNX16	113252997	2.45482393	1.29562	0.005172	0.016053
SHROOM4	113265963	2.454373303	1.295355	0.001563	0.005694
GPC1	113249723	2.454200086	1.295253	0.00165	0.005964
ATP5PF	113270115	2.453070366	1.294589	0.001059	0.004045

CEP85	113268462	2.45181516	1.29385	0.005944	0.018078
CRYZ	113254523	2.450440643	1.293041	0.001674	0.006033
LOC113248075	113248075	2.4490086	1.292198	0.012466	0.033661
EIF3F	113245854	2.447861365	1.291522	0.015826	0.041376
IRF2BPL	113270301	2.447013914	1.291022	0.000537	0.002229
FAM149B1	113259170	2.445966732	1.290405	0.00088	0.003443
MSRB3	113256321	2.445354445	1.290044	0.002333	0.008049
TBC1D17	113243277	2.445185008	1.289944	0.002846	0.009562
PALMD	113254365	2.445069248	1.289875	0.016598	0.042947
MAP2K6	113265227	2.443599776	1.289008	0.010784	0.029715
ST6GALNAC3	113254511	2.443282881	1.288821	0.016717	0.043231
LRP3	113244753	2.440521737	1.28719	0.006468	0.019427
BMT2	113263037	2.439590129	1.286639	0.000517	0.002163
NFATC2	113258729	2.439177004	1.286394	0.001277	0.004773
TIMM44	113242912	2.437622709	1.285475	0.000866	0.0034
NLRX1	113259964	2.436060661	1.28455	0.001329	0.004938
NEK7	113242585	2.434999588	1.283922	0.000808	0.003198
APLNR	113246359	2.434618001	1.283695	0.004243	0.01348
CD320	113242906	2.433699176	1.283151	0.001564	0.005695
TIMM8A	113244302	2.429945525	1.280924	0.001242	0.004656
QRSL1	113264780	2.424935734	1.277947	0.00137	0.005074
CCDC90B	113269384	2.423559429	1.277127	0.001038	0.003977
JMJD4	113247512	2.423022169	1.276808	0.00121	0.004551
FZD6	113252851	2.421221246	1.275735	0.00593	0.018054
MRPS22	113253995	2.417433506	1.273476	0.001045	0.004
RAPGEFL1	113265495	2.413720836	1.271259	0.018534	0.047168
METAP1D	113250774	2.413229384	1.270965	0.011453	0.031324
PODXL	113265973	2.412975864	1.270813	0.00397	0.012725

THRB	113253868	2.412073824	1.270274	0.002828	0.009513
HMOX2	113270845	2.411322687	1.269825	0.002452	0.008404
LOC113261831	113261831	2.410521093	1.269345	0.011041	0.030349
STARD8	113245626	2.409269358	1.268596	0.0023	0.007948
NOTCH4	113243767	2.40674323	1.267082	0.005204	0.016143
SHE	113245167	2.406638151	1.267019	0.00084	0.003311
GUF1	113262451	2.406438384	1.266899	0.000724	0.00291
DCBLD1	113259386	2.405569044	1.266378	0.010594	0.0293
RPP30	113243155	2.404721226	1.26587	0.001601	0.005812
MRPS7	113244641	2.403612751	1.265204	0.001756	0.006281
CUL1	113241451	2.403456443	1.265111	0.001241	0.004655
NRP1	113244231	2.401055075	1.263668	0.005113	0.015896
ETFB	113247289	2.399819069	1.262926	0.001656	0.005978
NAA50	113251480	2.399540494	1.262758	0.009302	0.026288
ANO1	113243981	2.399057123	1.262468	0.013108	0.035163
OSGEPL1	113250668	2.398133642	1.261912	0.004678	0.014707
EMC6	113269159	2.396847459	1.261138	0.001521	0.005564
IMMT	113242973	2.395743371	1.260473	0.004939	0.015423
PDAP1	113267911	2.395168052	1.260127	0.00114	0.004319
ATAD1	113258977	2.393678412	1.259229	0.001008	0.003878
EGFL8	113243749	2.393016899	1.258831	0.017542	0.045022
ABHD18	113255655	2.391541111	1.257941	0.001727	0.006193
MRPL48	113269296	2.391316781	1.257805	0.001642	0.005941
IL11RA	113260499	2.391196667	1.257733	0.004121	0.013143
LOC113252411	113252411	2.391118215	1.257685	0.017159	0.044197
JAM2	113269860	2.390645817	1.2574	0.017163	0.044199
GRK4	113245697	2.386913556	1.255146	0.000964	0.003727
CDH5	113252324	2.385289443	1.254164	0.011704	0.031939

RIPK2	113252964	2.384724761	1.253823	0.002493	0.008523
HSPA14	113241392	2.383269835	1.252942	0.000996	0.003836
P2RY2	113269285	2.380601867	1.251326	0.01245	0.033638
NSFL1C	113258424	2.380394341	1.251201	0.000693	0.0028
PET117	113258317	2.377385591	1.249376	0.016062	0.041813
KLHDC2	113266198	2.376020379	1.248547	0.001682	0.006056
PTCH1	113269779	2.37434622	1.24753	0.002943	0.009856
TRAP1	113270859	2.371511455	1.245807	0.001742	0.006242
MRPL4	113242977	2.370860766	1.245411	0.002304	0.007958
MRPL30	113246927	2.370041822	1.244913	0.003246	0.010702
PEX2	113253032	2.369846779	1.244794	0.002866	0.009619
FAIM	113253988	2.369662354	1.244682	0.016543	0.042873
HSF2	113259403	2.368368729	1.243894	0.00104	0.003981
LOC113248653	113248653	2.367782311	1.243536	0.005131	0.015945
RNF217	113259409	2.367567955	1.243406	0.00255	0.008693
SLC35A4	113261830	2.367073255	1.243104	0.001102	0.004186
FRAS1	113263237	2.365722233	1.242281	0.008895	0.025327
ANKRD28	113253911	2.365104348	1.241904	0.001506	0.005519
MECOM	113255350	2.364013954	1.241239	0.006584	0.019697
TUSC2	113256909	2.363518018	1.240936	0.001526	0.005579
DCTN6	113262126	2.357285868	1.237127	0.002107	0.007366
CLASP1	113263468	2.356227095	1.236479	0.001724	0.006187
ZNF205	113245431	2.356189668	1.236456	0.006757	0.020116
POP5	113267048	2.354914719	1.235675	0.00145	0.005331
NAMPT	113258637	2.353798466	1.234991	0.016242	0.042219
PSMB6	113271086	2.352656647	1.234291	0.002819	0.009489
ZC3HC1	113264149	2.351136217	1.233358	0.00138	0.00511
LOC113250022	113250022	2.34921651	1.23218	0.015193	0.039912

PTPRB	113256273	2.349074371	1.232092	0.001722	0.006181
AFG3L2	113245913	2.348622901	1.231815	0.008155	0.023561
MLST8	113245112	2.348169302	1.231536	0.002291	0.007927
UFSP1	113267845	2.348039732	1.231457	0.017156	0.044197
SMYD4	113269128	2.345655902	1.229991	0.000947	0.003675
RND3	113250895	2.342853253	1.228267	0.001812	0.00646
RAMP2	113265404	2.341893952	1.227676	0.003328	0.010944
TNKS2	113243161	2.340985499	1.227116	0.003916	0.012565
ZBTB46	113258821	2.33798233	1.225264	0.001405	0.005186
UXT	113265998	2.336714665	1.224482	0.000959	0.003711
FYTTD1	113252775	2.335879753	1.223966	0.001937	0.006837
APOOL	113242162	2.334985923	1.223414	0.002707	0.009165
KTN1	113242338	2.334924958	1.223376	0.007812	0.022752
CSPG4	113240998	2.332904395	1.222127	0.014029	0.037288
RCC1L	113267767	2.330764046	1.220803	0.000863	0.003391
DTWD1	113269605	2.330423168	1.220592	0.006728	0.020061
FLII	113271289	2.330387953	1.22057	0.012138	0.032939
LOC113245563	113245563	2.329619497	1.220094	0.002384	0.008196
MICALL1	113241788	2.328784883	1.219577	0.000593	0.002441
GINM1	113259546	2.328377135	1.219325	0.000948	0.003678
PTPN4	113263458	2.326198937	1.217974	0.001689	0.006077
ZNF704	113253012	2.325940328	1.217814	0.002186	0.007616
PHC1	113257919	2.323630676	1.216381	0.000981	0.003782
BCL7A	113266930	2.323191013	1.216108	0.003608	0.011741
SOBP	113264771	2.32264781	1.21577	0.002763	0.00933
MTSS1L	113251737	2.321956658	1.215341	0.000969	0.00374
EVPL	113244608	2.319853878	1.214034	0.019532	0.049328
TMEM250	113266597	2.317077481	1.212306	0.002087	0.007299

RTRAF	113242380	2.316127933	1.211715	0.008329	0.023961
LOC113249372	113249372	2.315117905	1.211086	0.004372	0.013828
LOC113246034	113246034	2.313958548	1.210363	0.004448	0.014043
MRPL23	113243957	2.312888569	1.209696	0.002643	0.008965
TMEM109	113247003	2.311465397	1.208808	0.006187	0.018722
DET1	113263675	2.307750424	1.206487	0.001142	0.004325
AKAP7	113259446	2.307526408	1.206347	0.008646	0.024725
EIF3J	113241944	2.307274448	1.20619	0.00153	0.005593
MRPL2	113261278	2.306956562	1.205991	0.004359	0.01379
ZFP91	113246399	2.306768242	1.205873	0.002194	0.007638
CHMP7	113269987	2.30508822	1.204822	0.001607	0.005826
TTBK2	113241979	2.305083683	1.204819	0.003832	0.012359
CDKN1C	113243966	2.303842272	1.204042	0.003036	0.010111
ZKSCAN5	113267889	2.297009871	1.199757	0.002349	0.008083
LOC113246791	113246791	2.295944221	1.199088	0.002916	0.009773
MMP24OS	113258533	2.295800921	1.198998	0.006501	0.019511
PNRC1	113264836	2.294229698	1.19801	0.005149	0.01599
POLR2F	113241794	2.293460889	1.197526	0.002323	0.008017
CACNA1C	113258052	2.292589207	1.196978	0.016857	0.043545
LOC113248147	113248147	2.29234737	1.196826	0.002453	0.008404
SELENOH	113246412	2.291741208	1.196444	0.001413	0.005212
FAM220A	113267940	2.291630422	1.196374	0.002054	0.007195
FAM107A	113257027	2.291165325	1.196082	0.004469	0.0141
ACOT6	113270358	2.289989159	1.195341	0.018479	0.047046
C1QBP	113271068	2.287869834	1.194005	0.003907	0.012546
PLA2G12A	113255558	2.285625614	1.192589	0.003445	0.011285
FIS1	113267833	2.283436833	1.191207	0.014986	0.039455
ZNF667	113248541	2.276697215	1.186942	0.004715	0.014808

PLGRKT	113270155	2.276113101	1.186572	0.008474	0.024307
RAB33B	113255687	2.275859951	1.186412	0.001279	0.004778
BBIP1	113251929	2.275766489	1.186353	0.003868	0.012438
RERE	113270548	2.274007245	1.185237	0.008469	0.024298
MRPS34	113245131	2.273070671	1.184643	0.003074	0.010222
HIPK3	113265060	2.272814497	1.18448	0.013286	0.035556
GATB	113255756	2.272181881	1.184078	0.003995	0.012792
SMIM4	113256962	2.270601542	1.183075	0.012445	0.033631
CNOT7	113262161	2.266575897	1.180514	0.000906	0.003532
PLS3	113241253	2.264571597	1.179238	0.007404	0.021718
PER1	113271140	2.264211923	1.179009	0.003375	0.011077
CLDN12	113259732	2.26414949	1.178969	0.007202	0.021227
MTERF2	113256032	2.263751319	1.178715	0.005025	0.015657
ARL2	113244145	2.262811449	1.178116	0.008201	0.023651
OPA3	113243423	2.262112507	1.177671	0.002604	0.008841
SGTB	113255279	2.260831531	1.176853	0.006584	0.019697
LOC113240740	113240740	2.260789431	1.176827	0.001581	0.005752
RAC3	113244670	2.260555509	1.176677	0.015906	0.041529
CUNH1orf123	113254652	2.260092853	1.176382	0.002195	0.007641
LOC113249770	113249770	2.259307034	1.17588	0.002582	0.008785
PDE7A	113268055	2.258967281	1.175663	0.006051	0.018367
PHB2	113257947	2.258782923	1.175546	0.008691	0.024835
TIMM9	113242320	2.258510142	1.175371	0.003965	0.012712
EBPL	113251313	2.25819667	1.175171	0.006163	0.018659
PDE2A	113269273	2.257801259	1.174918	0.012028	0.032683
TSPAN5	113263124	2.256965323	1.174384	0.010706	0.029555
SDHAF4	113261135	2.255368841	1.173363	0.002521	0.008605
PRKCA	113265250	2.255145979	1.173221	0.001765	0.006308

MTFMT	113240861	2.254575134	1.172856	0.014551	0.038442
MRPL40	113245891	2.253921045	1.172437	0.002911	0.009759
RABGAP1	113266382	2.251856768	1.171115	0.003474	0.011365
MLLT3	113260585	2.25152346	1.170902	0.009879	0.02763
LOC113248431	113248431	2.250110373	1.169996	0.005111	0.015895
ZBED1	113240783	2.248807695	1.16916	0.003589	0.011694
ZNF865	113247371	2.247950959	1.168611	0.002524	0.008614
ZNF639	113254787	2.247168258	1.168108	0.002516	0.008592
FGD6	113256139	2.246797692	1.16787	0.013057	0.035068
PIGBOS1	113269651	2.246648709	1.167775	0.00999	0.027888
LYRM7	113261690	2.241928525	1.16474	0.013682	0.036459
KIAA1328	113253353	2.241476257	1.164449	0.005475	0.01685
CUNH1orf50	113254804	2.241279184	1.164322	0.008073	0.023378
LOC113249854	113249854	2.240700025	1.16395	0.009668	0.02716
OPLAH	113247625	2.239194943	1.16298	0.002728	0.009219
MCF2L	113247581	2.238096005	1.162272	0.009454	0.026643
BBS7	113255630	2.237866259	1.162124	0.012024	0.032683
EIF2S3	113242224	2.237836375	1.162105	0.017309	0.044518
FAM219B	113240980	2.235563865	1.160639	0.002588	0.008797
CA9	113260473	2.234046993	1.15966	0.014917	0.039292
PPP1R15A	113243327	2.233534366	1.159328	0.001987	0.00699
NCKAP1	113250692	2.233423846	1.159257	0.007226	0.021284
COPS7A	113257966	2.232859454	1.158892	0.003485	0.011397
MAGI1	113257053	2.231518269	1.158026	0.010873	0.029948
MGRN1	113270841	2.228580508	1.156125	0.001921	0.006785
CNTF	113246400	2.228488986	1.156066	0.003022	0.010071
POLR3D	113270004	2.228311783	1.155951	0.002282	0.007899
FAM76A	113268432	2.227774214	1.155603	0.002354	0.0081

ORAI1	113266942	2.227579143	1.155477	0.002591	0.008806
NOP53	113243362	2.227418441	1.155373	0.017391	0.044686
LOC113248319	113248319	2.22686513	1.155014	0.0047	0.014769
TTC1	113264040	2.224354884	1.153387	0.002177	0.007586
TEK	113260555	2.223473379	1.152815	0.001245	0.004666
CYYR1	113269125	2.223401948	1.152769	0.003628	0.011795
PAIP1	113260958	2.22266773	1.152292	0.003536	0.011542
TFB1M	113259582	2.222245764	1.152018	0.004724	0.014828
SLC2A12	113259466	2.220194204	1.150686	0.007457	0.021854
HS6ST1	113263500	2.218589024	1.149642	0.003016	0.01006
LIPT1	113246901	2.218024864	1.149276	0.010494	0.029068
PHLDB1	113259986	2.216150331	1.148056	0.01656	0.042891
FAT4	113255648	2.215350561	1.147535	0.005755	0.017585
UTP18	113265640	2.215206534	1.147441	0.003352	0.011013
ARHGAP5	113266109	2.21291837	1.14595	0.008174	0.023601
SEC22C	113253823	2.212726037	1.145825	0.001913	0.006763
MIPEP	113251186	2.212469722	1.145658	0.006783	0.020166
ITPA	113258397	2.211780973	1.145209	0.00313	0.010378
EVI5L	113242919	2.210986386	1.14469	0.006156	0.01864
ALDH5A1	113264335	2.210943808	1.144662	0.016594	0.042946
TOMM6	113261308	2.210682202	1.144492	0.005552	0.017043
MAP2K4	113271224	2.20985546	1.143952	0.003091	0.010262
ABHD8	113256672	2.209305576	1.143593	0.004851	0.015178
TMEM140	113264458	2.208074773	1.142789	0.004172	0.013285
ANXA3	113263239	2.204690208	1.140576	0.00684	0.020301
ST6GALNAC6	113266442	2.203833264	1.140015	0.018869	0.047894
PRKCE	113246159	2.203584231	1.139852	0.009615	0.027026
UBFD1	113267577	2.202498278	1.139141	0.003043	0.010129

CAMLG	113261747	2.201559978	1.138526	0.003467	0.011344
CCDC58	113252235	2.199049937	1.13688	0.01351	0.03607
PSMD7	113252438	2.197318876	1.135744	0.003568	0.011634
MARCH6	113260817	2.196284803	1.135065	0.013242	0.035468
PAK1	113269353	2.196180183	1.134996	0.01204	0.032705
KLF10	113252856	2.194227081	1.133713	0.002588	0.008797
STN1	113251897	2.194042433	1.133591	0.002394	0.008222
RIOX2	113250339	2.192386072	1.132502	0.006514	0.019548
DUSP1	113264095	2.191996959	1.132246	0.009484	0.026717
SEC61A2	113241066	2.191796701	1.132114	0.014527	0.038402
CSGALNACT1	113245577	2.190190039	1.131056	0.005932	0.018054
ZNF775	113241469	2.187108254	1.129025	0.006388	0.019219
NIT2	113250540	2.186257582	1.128463	0.004034	0.012911
ZNHIT3	113268916	2.185055262	1.12767	0.018313	0.046685
RCHY1	113263266	2.184495845	1.1273	0.003636	0.011811
RNF113A	113241284	2.183172314	1.126426	0.010321	0.028684
CUNH1orf52	113254470	2.183111634	1.126386	0.008106	0.023462
SOCS6	113253516	2.182506418	1.125986	0.001902	0.006729
MLX	113265415	2.182279062	1.125836	0.005282	0.016331
MOSMO	113267551	2.181229331	1.125141	0.014182	0.037592
RAD23B	113260344	2.18022187	1.124475	0.014135	0.037506
AKAP2	113260334	2.180005752	1.124332	0.003327	0.010942
TSPAN17	113264132	2.179116972	1.123744	0.007518	0.022009
SNAPIN	113245335	2.179050634	1.1237	0.004697	0.014762
LAMA5	113258790	2.178366672	1.123247	0.016227	0.042192
NTPCR	113259022	2.177822944	1.122887	0.005386	0.016621
TCP1	113259618	2.177180952	1.122461	0.017289	0.044488
CUNH9orf3	113269782	2.176627882	1.122095	0.007356	0.021597

PCMTD1	113268108	2.175582729	1.121402	0.005991	0.018213
MRPL39	113269991	2.174231156	1.120505	0.004075	0.013012
TULP4	113259603	2.174219163	1.120497	0.008286	0.023861
NENF	113262675	2.172515007	1.119366	0.004827	0.015123
GON7	113267335	2.172127801	1.119109	0.007306	0.021481
REX1BD	113256726	2.172055124	1.119061	0.007061	0.02087
UCHL3	113251425	2.169894608	1.117625	0.013209	0.035384
CUNH18orf25	113253377	2.168930667	1.116984	0.003091	0.010262
AFF4	113261726	2.168254019	1.116534	0.004211	0.013389
FARS2	113264417	2.168183583	1.116487	0.011835	0.03225
SLMAP	113257009	2.166749613	1.115532	0.006627	0.019811
RNF126	113242698	2.165357777	1.114605	0.006583	0.019697
FASTKD3	113260808	2.165300065	1.114567	0.019822	0.049863
PKD1	113245185	2.164645153	1.114131	0.011157	0.030619
TPRKB	113243931	2.164181843	1.113822	0.008716	0.024889
ZNF629	113267696	2.162994623	1.11303	0.005185	0.016092
BRF1	113267180	2.161559477	1.112073	0.002027	0.007111
CIR1	113250759	2.157819374	1.109574	0.003571	0.011639
SUPV3L1	113259126	2.157306024	1.109231	0.005153	0.016
TEN1	113244615	2.156326612	1.108576	0.010553	0.029196
ARHGAP10	113255740	2.154797472	1.107552	0.011911	0.032432
RRNAD1	113245219	2.154283985	1.107208	0.005381	0.016615
PIM3	113241626	2.15081447	1.104883	0.003745	0.012109
VAPA	113253213	2.149306722	1.103871	0.015933	0.041583
MRTFB	113270776	2.146943492	1.102284	0.012628	0.034057
IRGQ	113243492	2.146065445	1.101694	0.003028	0.010088
DCUN1D5	113260132	2.145211629	1.10112	0.007605	0.022246
NPR1	113245333	2.141151951	1.098387	0.011588	0.031661

PCBD1	113259147	2.14058762	1.098007	0.005725	0.017511
BRI3	113267926	2.13920483	1.097075	0.004544	0.014311
JOSD2	113247298	2.13855448	1.096636	0.003444	0.011285
TOR1AIP1	113262694	2.137411416	1.095865	0.00625	0.018871
PFDN5	113257559	2.134731076	1.094054	0.006435	0.019335
MRPS33	113265203	2.133599301	1.093289	0.004183	0.013317
MDM2	113256295	2.133236279	1.093044	0.00514	0.015965
HAS3	113252417	2.13252626	1.092564	0.016021	0.041734
TBKBP1	113265554	2.12852895	1.089857	0.016425	0.042616
VAPB	113258765	2.124596463	1.087189	0.007948	0.02308
KLHL7	113261355	2.124019368	1.086797	0.003254	0.010726
PDZD2	113260852	2.124008269	1.086789	0.013279	0.035545
PGBD1	113248151	2.123513511	1.086453	0.019282	0.048834
SDC4	113258649	2.121876456	1.085341	0.00675	0.020108
BLZF1	113262863	2.121468701	1.085063	0.003811	0.0123
CRIPT	113246107	2.121446929	1.085049	0.004045	0.012941
NES	113245223	2.121209369	1.084887	0.007617	0.022262
LOC113256935	113256935	2.120999125	1.084744	0.016962	0.043782
PRKACA	113247197	2.120383005	1.084325	0.00748	0.021909
LDLRAD4	113245900	2.120175605	1.084184	0.012842	0.034592
PPP1R13B	113267205	2.118783348	1.083236	0.004332	0.013717
CUNH16orf70	113252344	2.117046673	1.082053	0.004163	0.013265
PCNX1	113270377	2.115226954	1.080812	0.006237	0.01884
ZNF550	113247988	2.112918318	1.079237	0.013882	0.036911
PIK3R3	113254730	2.11211251	1.078687	0.014531	0.038404
MRPL14	113261251	2.11182339	1.078489	0.006332	0.019079
ZFYVE9	113254667	2.110000958	1.077244	0.005448	0.016777
CAMTA2	113271056	2.109955378	1.077212	0.004238	0.013466

GABARAPL2	113252504	2.109824285	1.077123	0.006058	0.018382
ANGEL1	113270304	2.107184236	1.075316	0.011741	0.032026
LMAN2L	113246590	2.107181117	1.075314	0.009102	0.025807
MRPL55	113247506	2.106968975	1.075169	0.007609	0.022251
MAP3K14	113265323	2.104995358	1.073817	0.003552	0.011588
CDC42EP2	113244118	2.104675206	1.073598	0.008362	0.024036
NCKAP5L	113257641	2.103399311	1.072723	0.003391	0.01112
DCTN3	113260505	2.103180161	1.072572	0.006894	0.020429
MIA2	113266157	2.102945431	1.072411	0.008034	0.023291
ANKRA2	113255219	2.099864898	1.070297	0.010058	0.028038
VSIG10	113267020	2.099206089	1.069844	0.010626	0.029369
ST7L	113244418	2.09892262	1.069649	0.005096	0.015859
MAST4	113255270	2.098318771	1.069234	0.00656	0.019649
UBE3A	113263593	2.092809696	1.065441	0.010441	0.028952
REPS1	113259497	2.092479029	1.065213	0.006568	0.019665
NDUFAF7	113241364	2.092119459	1.064965	0.004934	0.015413
ATP9A	113258730	2.091767442	1.064722	0.018187	0.046388
TEFM	113268996	2.090677021	1.06397	0.01773	0.045419
RPS6KA3	113242238	2.090370727	1.063759	0.007828	0.022786
DPY30	113240973	2.086797236	1.06129	0.003079	0.010233
VPS37A	113262159	2.085010432	1.060055	0.004848	0.015177
MAGED1	113248824	2.081315572	1.057496	0.010743	0.029633
IPPK	113270091	2.081162974	1.05739	0.005648	0.017302
LOC113248525	113248525	2.075767449	1.053645	0.010555	0.029196
PPP3R1	113244743	2.07571988	1.053612	0.019726	0.049717
HDDC2	113259412	2.075608437	1.053534	0.007194	0.021212
KLHDC10	113264155	2.075309577	1.053327	0.006971	0.02064
PTPN21	113267384	2.072394906	1.051299	0.009706	0.027232

S1PR1	113254338	2.069265041	1.049118	0.009202	0.026064
RPS27L	113269713	2.068841709	1.048823	0.007691	0.022434
FARP1	113251485	2.067948001	1.0482	0.005845	0.017833
CISD3	113265543	2.066838351	1.047426	0.015515	0.040634
LOC113247090	113247090	2.064064055	1.045488	0.018665	0.047448
BOLA1	113246109	2.063833714	1.045327	0.006589	0.019705
TXN2	113255991	2.060723806	1.043151	0.010638	0.029389
ARL1	113256094	2.060593123	1.04306	0.016353	0.042477
TSG101	113265122	2.059197451	1.042082	0.007617	0.022262
KAT8	113267718	2.057254003	1.04072	0.004253	0.013508
ARNT2	113263574	2.05699608	1.040539	0.0145	0.038338
RABGGTA	113246008	2.051709	1.036826	0.005271	0.016305
MRPS18B	113264365	2.051383261	1.036597	0.013149	0.035259
WISP2	113258639	2.050300699	1.035836	0.015284	0.040118
NFS1	113258544	2.049901287	1.035554	0.014646	0.038679
RRP7A	113241695	2.0493664	1.035178	0.008111	0.023467
MRPL10	113265560	2.048943121	1.03488	0.01019	0.028372
MRPS14	113262800	2.048244571	1.034388	0.013248	0.03547
TMEM201	113270539	2.048088172	1.034278	0.01809	0.046178
RER1	113270608	2.047399103	1.033792	0.010001	0.027915
TUT4	113254666	2.046820142	1.033384	0.007314	0.021495
RASGRP3	113241175	2.044690319	1.031882	0.007281	0.021422
TTL	113242468	2.043335086	1.030926	0.007621	0.022263
TMEM242	113259595	2.042183682	1.030113	0.008438	0.024218
LOC113249905	113249905	2.042031871	1.030005	0.016285	0.042325
FOXJ3	113254815	2.039762491	1.028401	0.006302	0.019002
MTOR	113270510	2.039016695	1.027874	0.009872	0.027617
MUS81	113244088	2.038036516	1.02718	0.003404	0.01116

LOC113248505	113248505	2.036414337	1.026031	0.013	0.034957
MRPL57	113251178	2.036129813	1.02583	0.017126	0.044137
OTUD7B	113246121	2.03602031	1.025752	0.008332	0.023964
LOC113249903	113249903	2.036015978	1.025749	0.017834	0.045625
ZDHHC4	113267945	2.036013495	1.025747	0.016752	0.043306
BAG1	113260655	2.034930997	1.02498	0.015957	0.041632
APBB2	113262436	2.034899098	1.024957	0.003213	0.010618
NUFIP1	113251357	2.031336481	1.022429	0.017233	0.044364
COPS8	113250304	2.031315131	1.022414	0.010019	0.027952
MRPS17	113267731	2.031220092	1.022347	0.010396	0.028859
LOC113248526	113248526	2.030376829	1.021748	0.016727	0.043251
RAB28	113266684	2.022698862	1.016282	0.015655	0.040968
MKRN2	113257192	2.021812104	1.015649	0.008656	0.024745
PRELID3B	113258772	2.021065576	1.015116	0.012019	0.032683
DNAJC4	113244185	2.020003582	1.014358	0.009132	0.025878
ARIH2	113256850	2.018711912	1.013435	0.006155	0.01864
SLC41A1	113242474	2.01767028	1.01269	0.016198	0.04213
PELP1	113271092	2.016720888	1.012011	0.00757	0.022158
SLF2	113251839	2.013701915	1.00985	0.006832	0.020284
NCOR2	113266878	2.012637234	1.009087	0.017872	0.045715
MRPL53	113243720	2.012417852	1.00893	0.013207	0.035384
ZEB1	113244240	2.012349953	1.008881	0.006734	0.020074
DNAAF2	113266189	2.012176365	1.008757	0.015956	0.041632
LOC113253334	113253334	2.012145022	1.008734	0.008523	0.024421
GTPBP6	113240797	2.008214433	1.005913	0.009012	0.025594
CUNH11orf98	113247002	2.007615471	1.005483	0.009675	0.02716
KATNAL1	113251223	2.007515537	1.005411	0.011983	0.03261
CRBN	113257101	2.006116856	1.004406	0.007484	0.021916

ARPC5L	113266404	2.005516689	1.003974	0.007032	0.020799
CUL2	113244226	2.003726659	1.002686	0.006385	0.019215
VPS51	113244128	2.001258551	1.000908	0.010315	0.028684
LOC113244008	113244008	1.997469756	0.998174	0.00936	0.026419
GALNT2	113259280	1.997239813	0.998008	0.008234	0.023723
THAP4	113249348	1.996898795	0.997761	0.012967	0.034904
CUTA	113243809	1.994559842	0.99607	0.010902	0.030016
RAPGEF5	113261264	1.994259985	0.995854	0.012212	0.033101
DOCK4	113262961	1.992166038	0.994338	0.011604	0.031692
ANKMY2	113261003	1.991510573	0.993863	0.006553	0.019638
FOXK1	113267532	1.991462212	0.993828	0.00767	0.022382
CBFA2T2	113258496	1.990898901	0.99342	0.007047	0.020837
ZNF576	113243494	1.98947717	0.992389	0.011288	0.030934
EXO5	113268225	1.987498163	0.990954	0.01644	0.042645
MRPS30	113260961	1.985884742	0.989782	0.015999	0.041703
ZNF658	113269767	1.983934525	0.988364	0.016054	0.041803
SIK1	113253815	1.983442468	0.988007	0.01229	0.033265
TSR2	113247540	1.978195981	0.984185	0.010107	0.028152
DELE1	113255368	1.978141791	0.984146	0.006041	0.018341
TIGD5	113247635	1.976157753	0.982698	0.017793	0.045547
CUNH5orf22	113260851	1.975949866	0.982546	0.0104	0.028859
KCNMA1	113259211	1.975873591	0.982491	0.01965	0.049571
LOC113248019	113248019	1.975627028	0.982311	0.011909	0.032432
RNF25	113250449	1.975303712	0.982074	0.016469	0.042704
TCF4	113253440	1.974822663	0.981723	0.005545	0.017027
SGK1	113259468	1.973572814	0.98081	0.019041	0.048286
TXNDC15	113261751	1.973278507	0.980595	0.01076	0.029666
LANCL1	113250507	1.97096601	0.978903	0.013611	0.03629

UBXN2A	113270385	1.969723211	0.977993	0.006949	0.020579
ADAMTSL4	113246199	1.969546845	0.977864	0.017385	0.04468
ZNF280D	113269663	1.96900858	0.977469	0.010841	0.029867
MAP2K7	113242915	1.965735591	0.975069	0.010765	0.029674
SYNJ2BP	113270383	1.963776591	0.973631	0.010923	0.030062
SRP14	113242044	1.963417434	0.973367	0.018768	0.047674
ZNF330	113255703	1.96264644	0.9728	0.010972	0.030185
TSR1	113269138	1.962371198	0.972598	0.006751	0.020108
TIMM17A	113242544	1.962180731	0.972458	0.016577	0.042924
ANAPC15	113269268	1.961803676	0.972181	0.013363	0.03574
PIP4K2B	113265540	1.961090162	0.971656	0.011875	0.032346
E2F6	113269983	1.959741658	0.970663	0.011595	0.031674
EIF2B1	113266890	1.955998188	0.967905	0.016975	0.043799
TNFAIP1	113269026	1.95549555	0.967534	0.018047	0.046093
MAGI3	113244407	1.955011599	0.967177	0.019604	0.049485
LOC113243631	113243631	1.949713651	0.963262	0.015739	0.041181
NAA38	113271166	1.949328801	0.962977	0.016	0.041703
EPM2AIP1	113253770	1.947107919	0.961333	0.013097	0.035148
DAAM2	113243899	1.947106088	0.961331	0.013454	0.035944
TARS2	113246206	1.945133039	0.959869	0.010605	0.029316
WDR33	113263491	1.9397777	0.955891	0.0145	0.038338
SASH1	113259535	1.939297836	0.955534	0.00995	0.027802
SNUPN	113240995	1.938671912	0.955069	0.017402	0.044706
GPS2	113271126	1.934274834	0.951793	0.015611	0.040869
MTX2	113250734	1.934162306	0.951709	0.013054	0.035066
BTBD3	113258336	1.933084609	0.950905	0.015801	0.041318
DHRS3	113270489	1.931193238	0.949493	0.015307	0.040152
TMEM218	113259909	1.93004691	0.948636	0.016384	0.042533

VPS50	113260046	1.92876844	0.94768	0.013585	0.036228
PRUNE1	113246187	1.928594763	0.94755	0.012187	0.033052
LOC113248155	113248155	1.923603524	0.943811	0.015467	0.040524
ATP2B1	113256184	1.921902142	0.942535	0.013733	0.036565
CLASP2	113253749	1.92068636	0.941622	0.016997	0.043848
PDPR	113252482	1.91243113	0.935408	0.01678	0.043371
OPA1	113253461	1.911455687	0.934672	0.015226	0.039986
IFT172	113271207	1.910931725	0.934276	0.018636	0.047392
HSPBP1	113247405	1.908394541	0.932359	0.019432	0.049123
MRRF	113266372	1.908022665	0.932078	0.017593	0.045128
MRPL46	113263673	1.906824658	0.931172	0.018319	0.046692
ODR4	113262757	1.905222207	0.929959	0.009274	0.026225
TPP2	113251515	1.902730613	0.928071	0.011057	0.030382
ZNF277	113262977	1.901909837	0.927449	0.012976	0.034919
TSNAX	113259031	1.900731017	0.926554	0.015095	0.039702
MPV17	113271127	1.897531056	0.924123	0.018306	0.046676
ACTR8	113256981	1.895485453	0.922567	0.01767	0.0453
SPSB3	113245105	1.8929978	0.920673	0.014709	0.038823
UBE2M	113248037	1.890155083	0.918505	0.015286	0.040118
MTMR6	113251190	1.889258111	0.91782	0.017968	0.045934
VWA8	113251375	1.884830663	0.914435	0.012959	0.034894
DRAP1	113244085	1.87825624	0.909394	0.0105	0.029081
LOC113243118	113243118	1.876515061	0.908056	0.016397	0.042549
ARFGAP2	113264971	1.873745334	0.905925	0.017988	0.045969
UBIAD1	113270509	1.871109738	0.903894	0.014254	0.037767
THAP5	113262830	1.869813098	0.902894	0.0122	0.033081
KLF12	113251420	1.868654359	0.902	0.015318	0.040171
CUNH5orf24	113261749	1.866359762	0.900227	0.01543	0.040443

GPAT4	113245549	1.865616625	0.899653	0.017291	0.044488
KAT14	113258316	1.858519607	0.894154	0.013113	0.03517
MECP2	113247815	1.857492399	0.893356	0.016917	0.043683
NR1H2	113247267	1.856113472	0.892285	0.018443	0.046962
DCTN4	113263959	1.854340684	0.890906	0.016228	0.042192
APC	113254991	1.852250506	0.889279	0.0163	0.042346
ARL10	113264122	1.84792668	0.885908	0.019794	0.049815
EMC10	113247286	1.844282046	0.883059	0.018355	0.046756
AEBP2	113257831	1.842836887	0.881928	0.01871	0.047553
LOC113266825	113266825	1.839525826	0.879334	0.014679	0.038751
DHPS	113247138	1.822705393	0.866081	0.016935	0.04372
ATP13A1	113256602	-1.77899588	-0.83106	0.015075	0.039656
PRR3	113264368	-1.80018185	-0.84814	0.017022	0.043895
UBLCP1	113264035	-1.80957652	-0.85565	0.019478	0.04922
MFAP1	113241951	-1.8096146	-0.85568	0.019756	0.049765
MAT2B	113264057	-1.81905836	-0.86319	0.019302	0.048874
ZBTB33	113270907	-1.82743154	-0.86982	0.017115	0.044119
TATDN2	113257164	-1.82780274	-0.87011	0.01937	0.049001
SSR3	113254103	-1.83014397	-0.87196	0.019392	0.049039
CCNY	113244222	-1.8313167	-0.87288	0.01939	0.049039
LOC113246334	113246334	-1.83699636	-0.87735	0.017675	0.045305
ZMPSTE24	113268234	-1.8378264	-0.878	0.01363	0.036326
CERS4	113242908	-1.83948807	-0.8793	0.018186	0.046388
MPST	113255974	-1.84077454	-0.88031	0.01853	0.047166
HACE1	113264792	-1.84131973	-0.88074	0.019774	0.049776
DDX41	113264156	-1.84297826	-0.88204	0.017951	0.045899
CHORDC1	113259840	-1.84299347	-0.88205	0.016794	0.04339
DIS3	113251414	-1.85312097	-0.88996	0.018185	0.046388

ATP11C	113241878	-1.86043083	-0.89564	0.019311	0.04889
CDC14B	113269770	-1.86049049	-0.89568	0.019486	0.049232
LOC113269632	113269632	-1.8613788	-0.89637	0.014216	0.037675
VIRMA	113252924	-1.86151002	-0.89647	0.016045	0.041788
KHDRBS1	113268373	-1.86257033	-0.89729	0.019605	0.049485
KRI1	113242991	-1.86374674	-0.89821	0.018324	0.046695
BAZ1B	113267799	-1.86760675	-0.90119	0.018059	0.046115
GTPBP1	113241769	-1.86981987	-0.9029	0.018192	0.046392
RNMT	113245948	-1.87566055	-0.9074	0.019342	0.048949
RETREG2	113250438	-1.88158278	-0.91195	0.014468	0.038268
KDM5B	113242535	-1.88280261	-0.91288	0.016295	0.042342
TBC1D15	113256262	-1.88403321	-0.91382	0.008698	0.02485
TMEM33	113262442	-1.88420781	-0.91396	0.01019	0.028372
ZFC3H1	113256267	-1.88735128	-0.91636	0.018841	0.047834
GPAA1	113247675	-1.88828899	-0.91708	0.012793	0.034476
CNOT3	113247382	-1.88980028	-0.91823	0.011062	0.030388
EHMT2	113243735	-1.89038913	-0.91868	0.018768	0.047674
RTEL1	113258816	-1.89480048	-0.92205	0.017782	0.045527
TMOD3	113269629	-1.8953402	-0.92246	0.013098	0.035148
SRGAP2	113242467	-1.89546452	-0.92255	0.011456	0.031326
RNF4	113245685	-1.89602302	-0.92298	0.014356	0.038001
SUOX	113256446	-1.89839656	-0.92478	0.013625	0.03632
KLHL42	113257768	-1.90162148	-0.92723	0.016861	0.043547
TAF10	113245819	-1.90177108	-0.92734	0.01768	0.045308
TINF2	113246031	-1.90471966	-0.92958	0.017988	0.045969
ERMP1	113270147	-1.90501963	-0.92981	0.012459	0.033652
N4BP2	113262429	-1.90819801	-0.93221	0.019655	0.049573
WDFY1	113250397	-1.90855464	-0.93248	0.012209	0.033098

JRKL	113259806	-1.91029109	-0.93379	0.018917	0.047998
LOC113246844	113246844	-1.91067207	-0.93408	0.012476	0.033681
TTC14	113254672	-1.91229447	-0.9353	0.017459	0.044828
SNRPD1	113253272	-1.91335296	-0.9361	0.01648	0.042716
ANAPC7	113266956	-1.91748477	-0.93922	0.007334	0.021546
MFSD1	113254126	-1.91765613	-0.93934	0.011548	0.031564
TRAPPC10	113252526	-1.9181716	-0.93973	0.019099	0.048406
EAF1	113253915	-1.91953859	-0.94076	0.012983	0.034933
TMEM68	113268092	-1.91971271	-0.94089	0.013809	0.036732
EXOC2	113264377	-1.92059231	-0.94155	0.007091	0.020953
CPSF3	113269703	-1.92579851	-0.94546	0.014045	0.037316
CORO1B	113244027	-1.9260377	-0.94564	0.019759	0.049765
STT3A	113259904	-1.92753743	-0.94676	0.015973	0.041664
ARFGEF2	113258693	-1.9291095	-0.94794	0.011761	0.032063
RBM19	113267000	-1.92921967	-0.94802	0.019256	0.048787
NUP37	113256081	-1.93014012	-0.94871	0.018886	0.04793
DZIP3	113250993	-1.93117711	-0.94948	0.011045	0.030354
DNAJB11	113253948	-1.9318114	-0.94995	0.011224	0.030777
SMIM15	113255304	-1.93360404	-0.95129	0.014731	0.038865
TGS1	113268091	-1.93389067	-0.95151	0.018658	0.04744
DAGLA	113246950	-1.93435522	-0.95185	0.01637	0.042505
ASXL2	113270731	-1.93455166	-0.952	0.013358	0.035735
NOP56	113258410	-1.93523931	-0.95251	0.01027	0.028566
ADGRG6	113259504	-1.93619373	-0.95322	0.018563	0.047234
LOC113249006	113249006	-1.93697017	-0.9538	0.012275	0.03323
STX2	113266862	-1.93786241	-0.95447	0.0086	0.024619
APPBP2	113268908	-1.93792072	-0.95451	0.007885	0.022931
NELFE	113243750	-1.93837586	-0.95485	0.010102	0.028149

B3GLCT	113251234	-1.94063222	-0.95653	0.010064	0.028049
SHPK	113269155	-1.94180898	-0.9574	0.019266	0.048803
VPS25	113265402	-1.94197582	-0.95753	0.014096	0.037423
PIAS4	113242820	-1.94463651	-0.9595	0.0166	0.042947
LOC113252430	113252430	-1.94603539	-0.96054	0.017751	0.045462
CUNH9orf78	113266515	-1.94703488	-0.96128	0.012493	0.033719
PSEN1	113270366	-1.94710204	-0.96133	0.009527	0.026811
GXYLT1	113257488	-1.94713275	-0.96135	0.017443	0.044795
NRF1	113264100	-1.94777069	-0.96182	0.019676	0.04961
ANKLE2	113266836	-1.94796259	-0.96197	0.015009	0.039507
VPS4B	113253497	-1.94880833	-0.96259	0.009972	0.027849
TMEM208	113252358	-1.94916113	-0.96285	0.016558	0.042891
LOC113249879	113249879	-1.94947658	-0.96309	0.016968	0.043789
DNAJC3	113251474	-1.94976088	-0.9633	0.016479	0.042716
NKAPD1	113260051	-1.9501115	-0.96356	0.008911	0.025359
PDCD6	113260771	-1.95220505	-0.9651	0.012967	0.034904
DNAJA1	113260532	-1.95443012	-0.96675	0.013402	0.035826
IFT52	113258621	-1.9551115	-0.96725	0.013725	0.036551
COG4	113252472	-1.95528046	-0.96738	0.008731	0.024927
HAUS3	113245692	-1.95558589	-0.9676	0.016458	0.042684
DHX15	113266749	-1.95731316	-0.96887	0.018359	0.046757
CLINT1	113264026	-1.95735356	-0.9689	0.014919	0.039292
TRA2A	113261451	-1.9574904	-0.96901	0.015924	0.04157
GPR146	113247718	-1.95862167	-0.96984	0.016114	0.041936
RAB4B	113243553	-1.95918781	-0.97026	0.015161	0.039853
ATM	113260096	-1.95983944	-0.97074	0.012899	0.03474
NSF	113265315	-1.96046738	-0.9712	0.006152	0.018637
ENOPH1	113263217	-1.9607183	-0.97138	0.010009	0.02793

TRAPPC2	113240720	-1.96175078	-0.97214	0.009826	0.027521
VHL	113257162	-1.96197117	-0.9723	0.015073	0.039656
XRCC5	113250485	-1.96247584	-0.97267	0.014154	0.037539
PITPNM1	113244022	-1.96301403	-0.97307	0.015599	0.040846
YWHAH	113245070	-1.96347103	-0.97341	0.009065	0.025712
RHOF	113266940	-1.96478499	-0.97437	0.013188	0.035351
MAP3K1	113255319	-1.96513306	-0.97463	0.010466	0.028998
TXNRD1	113256059	-1.96551393	-0.97491	0.009344	0.026379
UGCG	113260314	-1.97013652	-0.9783	0.014456	0.038244
WDR81	113269127	-1.97025763	-0.97838	0.007185	0.02119
KIAA0040	113262795	-1.97297519	-0.98037	0.014562	0.038463
OSMR	113260912	-1.97307856	-0.98045	0.017433	0.044776
TMEM214	113270931	-1.97350822	-0.98076	0.008626	0.024676
BCAP29	113258566	-1.97468243	-0.98162	0.007753	0.022594
AAAS	113257557	-1.97625154	-0.98277	0.010544	0.029191
GABPB1	113269609	-1.97688005	-0.98323	0.015376	0.040317
LOC113246295	113246295	-1.97698818	-0.9833	0.009956	0.027812
VMP1	113268893	-1.97792544	-0.98399	0.009835	0.027536
CCDC88A	113245543	-1.97820932	-0.9842	0.012664	0.034149
CUNH20orf27	113258390	-1.97924023	-0.98495	0.015127	0.039779
LRRC14	113247678	-1.97955056	-0.98517	0.009764	0.027382
AGPS	113250728	-1.98021403	-0.98566	0.013391	0.03581
RBM15B	113256925	-1.98128998	-0.98644	0.00795	0.02308
HLTF	113254041	-1.98157761	-0.98665	0.007809	0.02275
CHTF8	113252418	-1.98174311	-0.98677	0.008029	0.02328
SH3GL1	113242831	-1.98247834	-0.98731	0.01473	0.038865
SNX27	113246219	-1.98249739	-0.98732	0.012088	0.032816
HMG20B	113242805	-1.98329729	-0.9879	0.009477	0.026705

TMEM165	113262510	-1.9836607	-0.98817	0.008109	0.023466
ORC2	113250599	-1.98373033	-0.98822	0.011519	0.031491
TRANK1	113253767	-1.9846244	-0.98887	0.014657	0.0387
FBXO38	113263924	-1.98545253	-0.98947	0.010408	0.028873
KMT5A	113266900	-1.98653649	-0.99026	0.006286	0.018963
SCAMP2	113240978	-1.98777564	-0.99115	0.007754	0.022594
MYDGF	113242846	-1.99162482	-0.99395	0.01195	0.032532
GLYR1	113270832	-1.99358461	-0.99536	0.012223	0.033122
ENTPD1	113251758	-1.9938131	-0.99553	0.015997	0.041703
U2AF1	113253956	-1.99515903	-0.9965	0.009104	0.025807
GTF2H3	113266889	-1.99653179	-0.9975	0.006189	0.018724
ARF6	113266201	-1.99824188	-0.99873	0.009671	0.02716
LOC113270621	113270621	-1.99887058	-0.99919	0.015865	0.041463
PIKFYVE	113250516	-1.99918803	-0.99941	0.015137	0.039798
RAB43	113257245	-2.00069318	-1.0005	0.012411	0.033549
PMF1	113245243	-2.00081411	-1.00059	0.01093	0.030074
MOSPD2	113240710	-2.001967	-1.00142	0.01073	0.029603
NAGPA	113270828	-2.00280644	-1.00202	0.0131	0.035148
FOPNL	113270759	-2.00310252	-1.00224	0.010601	0.029313
GALK2	113269599	-2.00415579	-1.00299	0.009714	0.027247
DCTN5	113267581	-2.00673592	-1.00485	0.005873	0.017917
TMEM106A	113265382	-2.00810517	-1.00583	0.014138	0.037506
PRKAB1	113267029	-2.00859483	-1.00619	0.015482	0.040555
HIP1R	113266911	-2.00885003	-1.00637	0.016581	0.042924
RABGAP1L	113262797	-2.01000124	-1.0072	0.008072	0.023378
SMARCA4	113243008	-2.01039976	-1.00748	0.008779	0.025038
IKBKG	113247832	-2.01144632	-1.00823	0.014095	0.037423
NUP188	113266493	-2.01251129	-1.009	0.005809	0.017742

LRRK1	113263631	-2.01486122	-1.01068	0.008019	0.023262
LOC113246280	113246280	-2.01694515	-1.01217	0.015879	0.041481
SLC8B1	113266994	-2.01715103	-1.01232	0.006813	0.020246
ALDH9A1	113268692	-2.01717514	-1.01234	0.011399	0.031195
ENPP4	113261238	-2.01792671	-1.01287	0.010249	0.028524
LRPAP1	113245731	-2.02172954	-1.01559	0.011088	0.030448
RHBDD1	113250388	-2.02372151	-1.01701	0.010917	0.03005
PI4KA	113247022	-2.02373783	-1.01702	0.015069	0.039656
HSD17B11	113263170	-2.0256874	-1.01841	0.006236	0.01884
POMK	113245578	-2.02787134	-1.01997	0.01407	0.037369
PGS1	113244672	-2.02912976	-1.02086	0.008568	0.024539
ATG7	113257175	-2.03198647	-1.02289	0.006408	0.019262
CDYL	113264414	-2.03247278	-1.02324	0.006772	0.020144
SLC30A5	113255261	-2.03307389	-1.02366	0.007019	0.020764
KLHL8	113263172	-2.03375694	-1.02415	0.004889	0.015282
TMEM183A	113242528	-2.03519881	-1.02517	0.005248	0.016251
NMRAL1	113270846	-2.03647683	-1.02608	0.008418	0.024176
AGRN	113270653	-2.03898208	-1.02785	0.005885	0.017944
SUMF2	113267740	-2.03919002	-1.028	0.017384	0.04468
IFT80	113254134	-2.03968216	-1.02834	0.012259	0.0332
PLOD3	113267834	-2.04039163	-1.02885	0.012746	0.034361
PAXIP1	113241515	-2.04112707	-1.02937	0.014885	0.039219
HEATR5A	113266100	-2.0416193	-1.02971	0.006865	0.020366
ZNF646	113267713	-2.04171083	-1.02978	0.008993	0.025545
RASA2	113254007	-2.04822819	-1.03438	0.005925	0.018045
PKN1	113247145	-2.04940002	-1.0352	0.007661	0.022362
PHF20L1	113252706	-2.05015545	-1.03573	0.008303	0.023896
LUC7L	113245359	-2.05023843	-1.03579	0.008204	0.023651

HERC4	113259104	-2.05199222	-1.03703	0.005591	0.017139
TRIM56	113267840	-2.05481102	-1.03901	0.00886	0.025249
THOC3	113264118	-2.05735147	-1.04079	0.013899	0.036949
SERTAD2	113244895	-2.05749367	-1.04089	0.011181	0.030679
ALYREF	113244498	-2.05806758	-1.04129	0.010231	0.02848
PLXNB2	113241610	-2.05946342	-1.04227	0.010267	0.028562
BRD3	113266580	-2.05961906	-1.04238	0.009429	0.026584
SLC38A6	113242406	-2.06038385	-1.04291	0.018945	0.048062
DPAGT1	113259971	-2.06084959	-1.04324	0.007199	0.021223
FAM204A	113251985	-2.06144614	-1.04366	0.004206	0.013378
HNRNPC	113248475	-2.0619695	-1.04402	0.014334	0.03795
PRKD3	113241389	-2.06232575	-1.04427	0.006541	0.019609
DYNLT1	113259604	-2.06274224	-1.04456	0.005931	0.018054
TMEM123	113260141	-2.06274602	-1.04457	0.007459	0.021856
SMARCD2	113265284	-2.06382986	-1.04532	0.005126	0.015935
RAB8B	113240830	-2.06556621	-1.04654	0.011684	0.03189
EIF2AK2	113241346	-2.06603659	-1.04687	0.005999	0.018233
TGOLN2	113243219	-2.06607143	-1.04689	0.019838	0.049888
SH3GLB2	113266495	-2.07030098	-1.04984	0.007004	0.020727
ATP6V1H	113268102	-2.07101933	-1.05034	0.004514	0.014224
SNAP23	113241986	-2.07159617	-1.05074	0.006744	0.020098
SNRPA	113243557	-2.07197095	-1.051	0.009448	0.026634
TBCD	113244459	-2.07248511	-1.05136	0.005748	0.017565
CD2AP	113261223	-2.0731208	-1.0518	0.006767	0.020136
LOC113264906	113264906	-2.07362464	-1.05215	0.005523	0.016979
PPP1R10	113264363	-2.0739922	-1.05241	0.004169	0.013281
FEM1C	113261602	-2.07499016	-1.0531	0.017136	0.044156
PLAGL1	113259514	-2.07596598	-1.05378	0.013745	0.036582

NUP98	113269254	-2.07808034	-1.05525	0.017823	0.045606
SELENOI	113270840	-2.0790996	-1.05596	0.007339	0.021556
LOC113248325	113248325	-2.08041594	-1.05687	0.017018	0.043894
SEPT9	113244579	-2.08122264	-1.05743	0.019622	0.049519
TNRC18	113267529	-2.0824096	-1.05825	0.014168	0.037562
SS18	113253303	-2.0841149	-1.05943	0.00622	0.01881
PLD3	113243570	-2.08426036	-1.05954	0.010784	0.029715
CUNH14orf119	113246018	-2.08434256	-1.05959	0.010393	0.028859
LOC113248842	113248842	-2.08483109	-1.05993	0.007256	0.021354
UST	113259539	-2.08591786	-1.06068	0.009589	0.026969
DCP2	113254985	-2.09043276	-1.0638	0.01255	0.033867
NUP93	113252267	-2.0906358	-1.06394	0.00384	0.012379
SLC7A8	113246015	-2.09163379	-1.06463	0.004708	0.014791
NPL	113262728	-2.09287515	-1.06549	0.019771	0.049776
CWF19L1	113251824	-2.09292561	-1.06552	0.013045	0.035057
SLC27A4	113266465	-2.0949574	-1.06692	0.005607	0.017186
RPS6KA4	113244169	-2.09522761	-1.06711	0.002796	0.009427
ABI1	113248690	-2.09578098	-1.06749	0.004192	0.013341
TCF3	113242743	-2.09606397	-1.06768	0.006751	0.020108
CDK4	113256363	-2.09821934	-1.06917	0.005034	0.01568
VRK3	113243270	-2.09842469	-1.06931	0.005446	0.016774
TXNDC12	113254678	-2.09853137	-1.06938	0.002987	0.009989
TRIM8	113251875	-2.09943601	-1.07	0.006683	0.019944
RGS7BP	113255291	-2.10112559	-1.07116	0.017703	0.045359
CHST12	113247737	-2.10124283	-1.07124	0.00968	0.027169
INTS9	113269938	-2.10210064	-1.07183	0.007225	0.021284
LFNG	113247705	-2.10305208	-1.07248	0.007926	0.023027
WASHC5	113252736	-2.10504223	-1.07385	0.003536	0.011542

SIGMAR1	113260503	-2.10522631	-1.07398	0.007244	0.021327
TTC9C	113246971	-2.10549835	-1.07416	0.008834	0.025184
HIRIP3	113267655	-2.10633229	-1.07473	0.010027	0.027958
CRAMP1	113245085	-2.10645274	-1.07482	0.009087	0.025771
PTPN9	113240994	-2.10645472	-1.07482	0.009336	0.026366
PRKX	113240773	-2.10646383	-1.07482	0.003457	0.011319
LSM7	113242763	-2.10692565	-1.07514	0.015876	0.041481
AP1M1	113256644	-2.10726424	-1.07537	0.00357	0.011638
MAP4K2	113244157	-2.10921244	-1.0767	0.008919	0.02537
SHC1	113245295	-2.10955545	-1.07694	0.016014	0.041733
NUDCD1	113252816	-2.10960635	-1.07697	0.005666	0.017349
IP6K1	113256884	-2.11309757	-1.07936	0.005189	0.016101
CEP162	113261056	-2.11328025	-1.07948	0.019753	0.049765
CDK19	113264736	-2.11401788	-1.07999	0.005887	0.017944
HELZ2	113258813	-2.1180902	-1.08276	0.007392	0.021698
GANAB	113246965	-2.11822871	-1.08286	0.010533	0.029166
ZNF518A	113251764	-2.12066966	-1.08452	0.007818	0.022766
WSB1	113269014	-2.12175986	-1.08526	0.011119	0.030521
LOC113268043	113268043	-2.12299105	-1.0861	0.003267	0.010759
NCLN	113242776	-2.1230355	-1.08613	0.003239	0.010681
TAPBP	113243801	-2.12333213	-1.08633	0.005016	0.015636
RELL1	113262399	-2.12514389	-1.08756	0.004231	0.013447
C2CD3	113269304	-2.12521407	-1.08761	0.00505	0.015726
LOC113246305	113246305	-2.12736939	-1.08907	0.006691	0.019962
WASF2	113268437	-2.12765145	-1.08926	0.014166	0.037562
WASHC1	113258027	-2.12920278	-1.09031	0.004619	0.01453
OGFRL1	113261127	-2.1300979	-1.09092	0.00276	0.009321
PTPN1	113258718	-2.13161624	-1.09195	0.006833	0.020284

AIDA	113268703	-2.1330516	-1.09292	0.008885	0.025309
KDM1B	113264506	-2.13375142	-1.09339	0.008775	0.025034
CBFB	113252342	-2.13583392	-1.0948	0.007907	0.02298
PBRM1	113256958	-2.13585037	-1.09481	0.007119	0.021017
STAT5A	113265423	-2.13697181	-1.09557	0.00367	0.011899
RANBP1	113245918	-2.1378855	-1.09618	0.004492	0.014172
WRAP53	113271147	-2.13850793	-1.0966	0.012269	0.033222
THADA	113246384	-2.13975624	-1.09745	0.00385	0.012393
CHSY1	113263628	-2.14004028	-1.09764	0.005228	0.016207
RBM14	113244054	-2.14066195	-1.09806	0.007614	0.022262
PSD3	113245558	-2.14335739	-1.09987	0.00539	0.01663
TMX4	113258348	-2.14335894	-1.09987	0.00656	0.019649
CPQ	113252904	-2.14421039	-1.10045	0.006761	0.020122
CAND1	113256307	-2.14483717	-1.10087	0.003649	0.01185
CELF2	113241055	-2.14638275	-1.10191	0.00302	0.010069
RBBP5	113242494	-2.14940104	-1.10393	0.008487	0.024334
TET2	113255522	-2.14950353	-1.104	0.010431	0.02893
PACS1	113244077	-2.1498984	-1.10427	0.00274	0.009257
OSBPL10	113253845	-2.15075544	-1.10484	0.015414	0.040409
CNIH4	113262906	-2.15347125	-1.10666	0.006929	0.020526
CCDC77	113258032	-2.15379681	-1.10688	0.010401	0.028859
LOC113259854	113259854	-2.15410777	-1.10709	0.00688	0.020393
PXMP4	113258499	-2.15493263	-1.10764	0.011339	0.031049
TTLL5	113270310	-2.15601919	-1.10837	0.005475	0.01685
PHF8	113247534	-2.15609949	-1.10842	0.008179	0.02361
BTD	113253913	-2.15672936	-1.10885	0.003266	0.010759
CHD1	113255048	-2.15689588	-1.10896	0.011112	0.030508
SMAGP	113257611	-2.15735978	-1.10927	0.009701	0.027222

RBM10	113265967	-2.15775511	-1.10953	0.00462	0.014532
LIMS1	113267768	-2.15817921	-1.10981	0.019051	0.048304
TRPM6	113269845	-2.1582415	-1.10986	0.012025	0.032683
STRBP	113266383	-2.1585214	-1.11004	0.00485	0.015177
BRPF3	113243856	-2.15954968	-1.11073	0.008983	0.025525
LOC113248305	113248305	-2.15958756	-1.11076	0.013547	0.036135
FAM20B	113262775	-2.16038723	-1.11129	0.003533	0.011536
BTAF1	113243164	-2.16057085	-1.11141	0.006211	0.018785
CHST3	113259154	-2.16312007	-1.11311	0.011309	0.030974
VPS35L	113270732	-2.16366531	-1.11348	0.001592	0.005785
LOC113255248	113255248	-2.16388739	-1.11363	0.015206	0.039941
PTGER4	113260927	-2.16505137	-1.1144	0.003482	0.011387
TAF5	113251886	-2.16520161	-1.1145	0.01495	0.039366
TLK1	113250784	-2.16737419	-1.11595	0.005089	0.015841
SLC25A51	113260437	-2.16810138	-1.11643	0.004441	0.014021
PTDSS1	113252906	-2.16862285	-1.11678	0.006538	0.019604
TERF1	113253053	-2.1699867	-1.11769	0.002955	0.009892
VPS16	113258406	-2.17314307	-1.11978	0.00185	0.006572
NSG1	113266669	-2.17474364	-1.12085	0.01101	0.030276
GLIPR1	113256250	-2.17511699	-1.12109	0.01107	0.030404
FMNL3	113257643	-2.1761376	-1.12177	0.017551	0.045036
GATAD2A	113256754	-2.17618253	-1.1218	0.001922	0.006789
TWF1	113257715	-2.17656966	-1.12206	0.010465	0.028998
SLC35A5	113251304	-2.17847684	-1.12332	0.002867	0.009619
BOD1L1	113266685	-2.17881324	-1.12354	0.010552	0.029196
NUP107	113256298	-2.17885122	-1.12357	0.004288	0.0136
FGL2	113259042	-2.17913613	-1.12376	0.009661	0.02715
WDR1	113266678	-2.17978312	-1.12418	0.018348	0.046746

CHD1L	113247760	-2.17988162	-1.12425	0.003259	0.010738
ARHGEF18	113242936	-2.18133477	-1.12521	0.006354	0.019128
OSTC	113255549	-2.18170587	-1.12546	0.003005	0.010028
PDZD8	113251975	-2.18240502	-1.12592	0.003136	0.010394
REXO2	113260032	-2.18339043	-1.12657	0.003849	0.012393
FBXO33	113266159	-2.18478714	-1.12749	0.008927	0.025386
DAP	113260820	-2.18568224	-1.12808	0.006347	0.019116
LOC113261754	113261754	-2.18604109	-1.12832	0.010685	0.029508
SRRT	113267842	-2.18613513	-1.12838	0.005491	0.016896
TMSB15B	113247226	-2.18622159	-1.12844	0.008892	0.025322
RNF114	113258707	-2.18755881	-1.12932	0.002339	0.008064
NFYA	113261320	-2.18789106	-1.12954	0.002707	0.009165
WRAP73	113270592	-2.19019129	-1.13106	0.006136	0.018593
PIGM	113246643	-2.19072928	-1.13141	0.015831	0.041381
LOC113241577	113241577	-2.19142143	-1.13187	0.016064	0.041813
MSL2	113253965	-2.19212275	-1.13233	0.002953	0.009888
RNF145	113264034	-2.1930055	-1.13291	0.002549	0.008693
STAT6	113256394	-2.19515218	-1.13432	0.003596	0.011712
ALG11	113251292	-2.19537786	-1.13447	0.004089	0.013053
CCAR1	113259115	-2.19646351	-1.13518	0.006285	0.018963
SREBF2	113241715	-2.19650195	-1.13521	0.006802	0.020219
SH2B2	113267828	-2.19717616	-1.13565	0.019598	0.049485
CNPY2	113256424	-2.19756683	-1.13591	0.004825	0.015118
NIPAL3	113268500	-2.1976577	-1.13597	0.006876	0.02039
ANTXR2	113263231	-2.19863171	-1.13661	0.004993	0.01557
SLC43A2	113269118	-2.19899077	-1.13684	0.005239	0.016228
EVL	113267273	-2.19910085	-1.13691	0.010023	0.027955
RPIA	113242784	-2.20094292	-1.13812	0.005563	0.017069

HEATR5B	113241303	-2.201003	-1.13816	0.005515	0.016962
SPTSSA	113266116	-2.20242861	-1.1391	0.002017	0.007084
ATP6V0D1	113252366	-2.20332995	-1.13969	0.002575	0.008764
PIP4K2C	113256375	-2.20370184	-1.13993	0.002673	0.009058
SNRNP40	113268389	-2.20592073	-1.14138	0.005512	0.016955
CCND2	113258003	-2.20662388	-1.14184	0.00814	0.023528
GABPB2	113246129	-2.20677837	-1.14194	0.01725	0.044398
FITM2	113258631	-2.20695729	-1.14206	0.003686	0.011941
SUPT20H	113251262	-2.20935903	-1.14363	0.004172	0.013285
CLHC1	113245595	-2.21044829	-1.14434	0.013699	0.036498
DHX9	113262730	-2.21087982	-1.14462	0.003708	0.012006
SF3B3	113252470	-2.21155096	-1.14506	0.008002	0.023228
AMMECR1L	113263495	-2.21253902	-1.1457	0.003004	0.010028
LIN9	113262924	-2.21254072	-1.1457	0.01119	0.030691
MSL1	113265497	-2.21262145	-1.14576	0.00494	0.015424
EIF2AK3	113242804	-2.21263127	-1.14576	0.001972	0.006948
IPMK	113259062	-2.21300716	-1.14601	0.015443	0.040469
S100PBP	113268341	-2.21497651	-1.14729	0.004501	0.014193
SURF4	113266566	-2.21598869	-1.14795	0.006718	0.020033
SMPD4	113245964	-2.21653156	-1.1483	0.002799	0.009436
SMC5	113270208	-2.21787047	-1.14918	0.005007	0.01561
TIAM2	113259585	-2.22026752	-1.15073	0.011743	0.032028
SLC9A9	113254028	-2.22067743	-1.151	0.005527	0.016982
NUMB	113270364	-2.22331344	-1.15271	0.001345	0.004994
CREG1	113262883	-2.2252514	-1.15397	0.014326	0.037937
RASA3	113247558	-2.22529906	-1.154	0.012408	0.033549
CNNM4	113246616	-2.22537401	-1.15405	0.003016	0.01006
ADCK5	113247605	-2.22560385	-1.1542	0.00282	0.009489

SLC11A2	113257616	-2.22604881	-1.15449	0.006081	0.018439
CFD	113242706	-2.22639523	-1.15471	0.00282	0.009489
ISY1	113257244	-2.22748166	-1.15541	0.002494	0.008523
FLI1	113259871	-2.22783915	-1.15565	0.004943	0.015427
DDOST	113268561	-2.22792762	-1.1557	0.015183	0.039894
ABCD3	113254387	-2.23006734	-1.15709	0.002404	0.008252
SLC16A3	113244477	-2.23132564	-1.1579	0.013248	0.03547
FCHSD1	113255372	-2.23363579	-1.15939	0.004796	0.015031
LRMDA	113258872	-2.23466201	-1.16006	0.015632	0.040916
MCCC1	113254564	-2.23809333	-1.16227	0.006421	0.019298
COMMD6	113251423	-2.23851456	-1.16254	0.008303	0.023896
SLCO2B1	113269323	-2.23903257	-1.16288	0.004319	0.013678
HCFC1	113247827	-2.23979955	-1.16337	0.009385	0.026473
IRF9	113245987	-2.24054332	-1.16385	0.003062	0.010183
COPG2	113264252	-2.2406071	-1.16389	0.002	0.007034
UBA7	113256887	-2.24315476	-1.16553	0.002299	0.007948
TRAFD1	113266979	-2.24511533	-1.16679	0.001689	0.006077
LOC113264918	113264918	-2.24522771	-1.16686	0.00608	0.018438
SLC46A3	113251217	-2.24582686	-1.16725	0.007135	0.021057
RSAD2	113269410	-2.24598502	-1.16735	0.006755	0.020113
EXOSC2	113266524	-2.24716535	-1.16811	0.00835	0.024011
DONSON	113263956	-2.24848183	-1.16895	0.003005	0.010028
ZFAND2A	113247742	-2.24849536	-1.16896	0.006086	0.018449
MUTYH	113254745	-2.24890584	-1.16922	0.008125	0.023494
FAM13B	113261777	-2.25296808	-1.17183	0.003153	0.010444
C2CD2L	113259970	-2.25531955	-1.17333	0.005395	0.016638
TRIOBP	113241800	-2.25578004	-1.17363	0.002615	0.008872
LOC113257253	113257253	-2.25582359	-1.17365	0.002386	0.008199

ERN1	113265667	-2.25633525	-1.17398	0.003519	0.0115
DLG3	113245635	-2.25703163	-1.17443	0.010318	0.028684
GNAI3	113244920	-2.25914498	-1.17578	0.002221	0.007721
CTSO	113255784	-2.25983633	-1.17622	0.002782	0.009389
ATXN7	113257039	-2.26004984	-1.17635	0.008432	0.024207
EIF2AK4	113242045	-2.26067022	-1.17675	0.003648	0.01185
RSU1	113241374	-2.26077241	-1.17682	0.002167	0.007553
TOP3A	113271284	-2.26118086	-1.17708	0.002387	0.008201
SAMD1	113247173	-2.26124632	-1.17712	0.002845	0.009562
NDC1	113254647	-2.26236851	-1.17783	0.007904	0.022978
BRWD3	113242156	-2.26248408	-1.17791	0.005952	0.0181
DARS2	113262813	-2.26407876	-1.17892	0.008746	0.024959
ATG3	113251330	-2.26425375	-1.17904	0.001745	0.006249
CBX3	113261778	-2.26561911	-1.17991	0.007401	0.021718
SLK	113251899	-2.2671161	-1.18086	0.011993	0.032626
SH3BP2	113245745	-2.26828514	-1.1816	0.001587	0.005769
RYBP	113257084	-2.26855687	-1.18177	0.002999	0.010017
LOC113249365	113249365	-2.26893527	-1.18202	0.001664	0.006006
HSPA4L	113255652	-2.27200188	-1.18396	0.002939	0.009845
RBM43	113250891	-2.2724275	-1.18423	0.002471	0.008456
PDCD10	113255525	-2.27265123	-1.18438	0.002224	0.007726
PIAS1	113240901	-2.27281241	-1.18448	0.004056	0.012963
NAA40	113244193	-2.27292752	-1.18455	0.002106	0.007361
CCNC	113264799	-2.27331088	-1.18479	0.000679	0.002751
AGFG2	113267853	-2.27551856	-1.1862	0.002066	0.007236
HNMT	113263407	-2.27884305	-1.1883	0.003262	0.010748
ARHGEF6	113241872	-2.2795886	-1.18877	0.011291	0.030938
ADAR	113245303	-2.2800601	-1.18907	0.002481	0.008485

THAP1	113245584	-2.28023465	-1.18918	0.002848	0.009569
ZNF746	113241462	-2.28050028	-1.18935	0.003919	0.012572
SINHCAF	113257753	-2.28080557	-1.18954	0.01119	0.030691
POC5	113255194	-2.28366938	-1.19135	0.003831	0.012359
TPRA1	113257274	-2.28393083	-1.19152	0.003373	0.011072
PRDM10	113259863	-2.28458327	-1.19193	0.008626	0.024676
WWOX	113251649	-2.28465829	-1.19198	0.019808	0.049841
SLC30A7	113254342	-2.28484127	-1.19209	0.00998	0.027866
SSH3	113244038	-2.28662811	-1.19322	0.003661	0.01188
DKC1	113247818	-2.28760101	-1.19384	0.002669	0.009049
OTULIN	113260835	-2.2881677	-1.19419	0.001465	0.005383
LYPLA2	113268516	-2.28837227	-1.19432	0.001967	0.006932
UGGT2	113251476	-2.29013882	-1.19544	0.017572	0.045084
FAM126B	113250597	-2.29286621	-1.19715	0.006828	0.020282
SH3KBP1	113242243	-2.29299719	-1.19723	0.002528	0.008626
ACCS	113265012	-2.29344708	-1.19752	0.019317	0.048895
ABCC4	113251471	-2.2950493	-1.19853	0.019432	0.049123
ZNF689	113267685	-2.29551853	-1.19882	0.005108	0.015889
NAPRT	113247656	-2.29612939	-1.1992	0.002206	0.007671
TMEM170B	113264454	-2.29854873	-1.20072	0.010689	0.029513
PRKDC	113268119	-2.30231002	-1.20308	0.002346	0.008081
GAPVD1	113266412	-2.30290401	-1.20345	0.009777	0.027408
AKAP10	113271266	-2.30324951	-1.20367	0.001588	0.00577
UNC119	113269034	-2.30337836	-1.20375	0.002907	0.009748
SND1	113263729	-2.30431806	-1.20434	0.003794	0.012254
RAP1GDS1	113263127	-2.30539178	-1.20501	0.001647	0.005956
RBM23	113246050	-2.30593509	-1.20535	0.003696	0.011972
PDS5A	113262428	-2.30785451	-1.20655	0.001816	0.00647

SORD	113269562	-2.30831858	-1.20684	0.002268	0.007858
PBX3	113266415	-2.30976815	-1.20775	0.005557	0.017054
PAPOLA	113267289	-2.30977477	-1.20775	0.007734	0.022547
CUNH5orf15	113261733	-2.31074838	-1.20836	0.001776	0.006345
TAF15	113268952	-2.31150525	-1.20883	0.001715	0.006159
UHMK1	113268670	-2.31237234	-1.20937	0.014447	0.038228
STRN	113241282	-2.31406865	-1.21043	0.003895	0.012515
MERTK	113242400	-2.31485132	-1.21092	0.008054	0.023333
BAX	113243322	-2.31537928	-1.21125	0.008359	0.024033
ATP6V0A2	113266887	-2.31538259	-1.21125	0.00202	0.007093
JAK2	113270160	-2.31567987	-1.21144	0.018734	0.047605
PPM1D	113268907	-2.31676753	-1.21211	0.003796	0.012255
SRPK1	113243851	-2.31697367	-1.21224	0.004285	0.013592
BBS9	113262551	-2.31850259	-1.21319	0.008776	0.025034
STARD3NL	113258380	-2.31897481	-1.21349	0.001831	0.006519
SLC5A3	113263134	-2.31898417	-1.21349	0.006341	0.019103
RMI1	113269806	-2.32111265	-1.21482	0.011003	0.030264
CLCN7	113245088	-2.3211295	-1.21483	0.002992	0.009998
MAP3K2	113263482	-2.32319634	-1.21611	0.005099	0.015863
MAML1	113261557	-2.32503192	-1.21725	0.00354	0.01155
SLC38A10	113244523	-2.32513438	-1.21731	0.003678	0.011922
ATF7IP	113257860	-2.32602224	-1.21786	0.002239	0.007766
ITPRIP	113251903	-2.32690561	-1.21841	0.003524	0.011511
CEP70	113253986	-2.32752156	-1.21879	0.001242	0.004656
FCHSD2	113269281	-2.32814028	-1.21918	0.001915	0.006767
PDIA6	113269914	-2.32942726	-1.21998	0.006525	0.019576
MCRIP1	113244504	-2.3314335	-1.22122	0.003076	0.010227
CCDC167	113243881	-2.33443525	-1.22307	0.004734	0.014855

ZMYND8	113258683	-2.33462993	-1.22319	0.0022	0.007654
NOP58	113250578	-2.33473017	-1.22326	0.001557	0.005674
TRIM38	113264255	-2.33573658	-1.22388	0.001022	0.003925
SEC11A	113263731	-2.33716377	-1.22476	0.000646	0.002629
PIGG	113245729	-2.33752383	-1.22498	0.000938	0.003642
GMFB	113242356	-2.33873989	-1.22573	0.001086	0.004134
LOC113246347	113246347	-2.34055183	-1.22685	0.003004	0.010028
RAP2B	113254081	-2.34062711	-1.2269	0.001852	0.006578
AP2A2	113243945	-2.34106323	-1.22716	0.006842	0.020301
ATXN3	113267348	-2.34547643	-1.22988	0.002566	0.008741
M6PR	113257920	-2.34640737	-1.23045	0.001472	0.005406
HSPH1	113251233	-2.34679217	-1.23069	0.000957	0.003707
PIK3CB	113253989	-2.35038022	-1.23289	0.002128	0.007429
FUK	113252471	-2.35119523	-1.23339	0.003361	0.011034
ERRFI1	113270554	-2.35311367	-1.23457	0.004718	0.014813
TPST1	113267749	-2.35650253	-1.23665	0.009833	0.027534
FRYL	113262473	-2.35743054	-1.23722	0.002066	0.007236
ZDHHC3	113256770	-2.35891662	-1.23812	0.001395	0.005153
MPDU1	113271157	-2.36116124	-1.2395	0.002237	0.00776
AGO2	113248463	-2.36168936	-1.23982	0.004094	0.013066
FRMD8	113244113	-2.36304443	-1.24065	0.001827	0.006504
TXNDC11	113270790	-2.36323402	-1.24076	0.00103	0.003954
LGALS3	113242344	-2.36349122	-1.24092	0.016396	0.042549
STT3B	113253844	-2.36522174	-1.24198	0.00927	0.026219
MPP1	113247819	-2.36535933	-1.24206	0.001454	0.005343
INSIG1	113241516	-2.36564376	-1.24223	0.002234	0.007756
LOC113250365	113250365	-2.36638627	-1.24269	0.001427	0.005259
POLA1	113242207	-2.36813846	-1.24375	0.003842	0.012383

INAFM2	113242037	-2.36858365	-1.24402	0.001796	0.00641
LRR1	113266193	-2.37058346	-1.24524	0.010446	0.02896
SLC12A7	113260783	-2.37213559	-1.24619	0.001159	0.004379
LOC113244953	113244953	-2.37564775	-1.24832	0.001225	0.0046
DHX58	113265431	-2.37645784	-1.24881	0.007464	0.021867
FAM49A	113270107	-2.37910348	-1.25042	0.002519	0.0086
CASP9	113270476	-2.3796313	-1.25074	0.001847	0.006562
INTS2	113268902	-2.3797396	-1.2508	0.004063	0.012983
LOC113265899	113265899	-2.38059876	-1.25132	0.004951	0.015448
TEX30	113251516	-2.38134796	-1.25178	0.019458	0.049179
GMEB1	113268407	-2.38156891	-1.25191	0.001279	0.004779
TGFB1	113243540	-2.38282206	-1.25267	0.003021	0.01007
SAP30	113269904	-2.38511157	-1.25406	0.002816	0.009484
ATP6V0E1	113264101	-2.38523439	-1.25413	0.000968	0.003739
ASAH1	113262148	-2.38555865	-1.25433	0.006657	0.019878
TBCK	113255530	-2.38742277	-1.25545	0.000694	0.002801
COLGALT1	113256686	-2.38752404	-1.25552	0.002458	0.008419
ASB1	113249875	-2.38766829	-1.2556	0.013193	0.035355
MAN1A1	113259398	-2.39195053	-1.25819	0.002458	0.008419
ZEB2	113263391	-2.39499576	-1.26002	0.00565	0.017304
NCBP1	113260423	-2.396178	-1.26074	0.001269	0.004748
WLS	113254544	-2.39715961	-1.26133	0.003155	0.010448
HERPUD2	113262709	-2.39716868	-1.26133	0.003633	0.011808
SIPA1L1	113270378	-2.39795791	-1.26181	0.001857	0.006592
DCAF7	113265292	-2.39869696	-1.26225	0.003716	0.012026
PHF6	113270972	-2.39971997	-1.26287	0.000563	0.00233
PRR14L	113245064	-2.40044334	-1.2633	0.004592	0.014453
RAD50	113261715	-2.4009676	-1.26362	0.000525	0.002191

ATL3	113244200	-2.40356268	-1.26517	0.006022	0.018297
LOC113249588	113249588	-2.40490421	-1.26598	0.006656	0.019878
TBC1D2	113260414	-2.40544456	-1.2663	0.007939	0.02306
LOC113271041	113271041	-2.40669879	-1.26706	0.001411	0.005206
RIMS3	113254828	-2.41546477	-1.2723	0.016556	0.042891
LOC113245500	113245500	-2.41703716	-1.27324	0.0054	0.01664
RAD51C	113268879	-2.41965131	-1.2748	0.0058	0.017716
ERO1A	113242373	-2.42090801	-1.27555	0.000895	0.003496
CKS1B	113245297	-2.42096286	-1.27558	0.000712	0.002868
CUNH4orf33	113255669	-2.42176556	-1.27606	0.007349	0.02158
SRGAP1	113256335	-2.42266905	-1.2766	0.013092	0.035148
CTDP1	113253546	-2.42374623	-1.27724	0.001405	0.005186
IL4R	113267608	-2.42542281	-1.27824	0.001087	0.004138
PLEKHO1	113246200	-2.42762186	-1.27954	0.000866	0.0034
TMEM41A	113254077	-2.42813552	-1.27985	0.001216	0.004569
LOC113248631	113248631	-2.43070733	-1.28138	0.002987	0.009989
AGA	113262234	-2.43100602	-1.28155	0.002255	0.007816
ZNF281	113242574	-2.43322973	-1.28287	0.003862	0.012426
LOC113257653	113257653	-2.43483615	-1.28382	0.010654	0.029428
SLC5A6	113271061	-2.43540185	-1.28416	0.014322	0.037934
PARP16	113240869	-2.43598966	-1.28451	0.004148	0.013223
TESK2	113254746	-2.43704357	-1.28513	0.00356	0.011611
GCC2	113267759	-2.4371348	-1.28519	0.000963	0.003721
BTBD19	113254758	-2.43773996	-1.28554	0.014741	0.038883
LOC113249565	113249565	-2.43920986	-1.28641	0.009216	0.026083
TSPYL5	113252901	-2.4393394	-1.28649	0.004	0.012807
TMEM181	113259336	-2.43934649	-1.28649	0.000979	0.00378
GLTP	113267095	-2.44100227	-1.28747	0.000729	0.002923

NUP155	113260902	-2.44130759	-1.28765	0.001602	0.005812
P4HA2	113261705	-2.44239897	-1.2883	0.004071	0.013003
CCDC34	113265091	-2.44243863	-1.28832	0.005728	0.017517
SLC9A8	113258703	-2.44398274	-1.28923	0.001882	0.006669
FZR1	113242800	-2.44979586	-1.29266	0.001276	0.00477
ASXL1	113258479	-2.45054703	-1.2931	0.001754	0.006275
CSTB	113253208	-2.45185544	-1.29387	0.001898	0.006719
TBC1D1	113262401	-2.45276276	-1.29441	0.001142	0.004325
MBD2	113253429	-2.45534272	-1.29592	0.000901	0.003514
VAMP7	113248338	-2.45791717	-1.29744	0.001509	0.005527
MYO5A	113269637	-2.45803042	-1.2975	0.000295	0.001318
FBXO7	113256021	-2.45954416	-1.29839	0.001003	0.00386
BCORL1	113270949	-2.46009776	-1.29872	0.00115	0.004352
IGFBP4	113265487	-2.46023966	-1.2988	0.014548	0.038442
LOC113250152	113250152	-2.46615579	-1.30226	0.018818	0.047792
RFC5	113267017	-2.46845966	-1.30361	0.002361	0.008119
LGALS8	113258940	-2.46849696	-1.30363	0.000632	0.00258
ZNF789	113267906	-2.46969885	-1.30434	0.009414	0.026547
TMEM209	113264171	-2.47060692	-1.30487	0.000562	0.002325
CPEB2	113266689	-2.47258474	-1.30602	0.001195	0.004499
CTHRC1	113252850	-2.47387743	-1.30677	0.018822	0.047793
SRD5A3	113262505	-2.47469992	-1.30725	0.00638	0.019203
TAF6L	113246934	-2.47522235	-1.30756	0.001622	0.005872
TFDP1	113247569	-2.47553837	-1.30774	0.000671	0.002721
NONO	113245615	-2.4755509	-1.30775	0.004109	0.01311
MANSC1	113257876	-2.47564423	-1.3078	0.00071	0.002862
ITSN2	113267964	-2.47578897	-1.30789	0.001836	0.006529
PGRMC1	113241269	-2.47599168	-1.30801	0.00083	0.003276

PCGF6	113251887	-2.47700937	-1.3086	0.005231	0.016211
PDE3A	113257829	-2.47739101	-1.30882	0.006268	0.018922
HNRNPUL1	113243542	-2.47934848	-1.30996	0.017342	0.044593
NINJ1	113270103	-2.48051994	-1.31064	0.001654	0.005972
MARVELD1	113251798	-2.48148252	-1.3112	0.003086	0.010251
CDC42SE2	113261694	-2.48364902	-1.31246	0.000801	0.003172
CEMIP2	113270218	-2.48425365	-1.31281	0.006833	0.020284
NECTIN1	113259953	-2.48480973	-1.31314	0.013498	0.036046
PPP1R12A	113256222	-2.48493226	-1.31321	0.003147	0.010425
LOC113246915	113246915	-2.48508823	-1.3133	0.001181	0.004452
GNG10	113260317	-2.48695005	-1.31438	0.006629	0.019812
LOC113255280	113255280	-2.48729693	-1.31458	0.019775	0.049776
GRIPAP1	113265940	-2.48736034	-1.31462	0.001189	0.00448
SH3TC1	113245686	-2.48746073	-1.31467	0.002041	0.00716
GTPBP2	113261256	-2.48916879	-1.31566	0.00171	0.006144
ILF2	113245334	-2.48940623	-1.3158	0.000563	0.002329
PHLDA1	113256247	-2.49202596	-1.31732	0.00557	0.01708
MPV17L2	113256709	-2.49648498	-1.3199	0.00102	0.00392
GSTO1	113251904	-2.49662217	-1.31998	0.001534	0.005603
GLA	113244306	-2.49745976	-1.32046	0.000592	0.002438
IFIH1	113250835	-2.49872904	-1.32119	0.002006	0.007052
DAB2	113260922	-2.49921238	-1.32147	0.00108	0.004112
ST3GAL5	113243005	-2.50025133	-1.32207	0.001316	0.004899
SLC39A7	113243792	-2.50047581	-1.3222	0.000991	0.003819
PSMB10	113252392	-2.50248314	-1.32336	0.013754	0.0366
PLPP6	113270168	-2.50365409	-1.32404	0.001872	0.006639
EPHX1	113262914	-2.50395853	-1.32421	0.019694	0.049646
GCHFR	113242025	-2.50448814	-1.32452	0.001157	0.004373

MBP	113253537	-2.50710828	-1.32602	0.014885	0.039219
SLC31A2	113266308	-2.50883333	-1.32702	0.000801	0.003172
SLC31A1	113266309	-2.50930103	-1.32729	0.000352	0.001538
MRE11	113259822	-2.51005218	-1.32772	0.001434	0.00528
MYLIP	113264487	-2.51013389	-1.32776	0.000604	0.002477
SSC5D	113247457	-2.5114984	-1.32855	0.017516	0.044964
LOC113249736	113249736	-2.51293944	-1.32938	0.005704	0.017453
PLEKHF2	113252914	-2.51299759	-1.32941	0.001323	0.004922
RIF1	113250886	-2.51313423	-1.32949	0.003915	0.012565
GPR171	113254068	-2.5155776	-1.33089	0.018582	0.047273
LOC113249190	113249190	-2.51680194	-1.33159	0.003915	0.012565
RBM15	113244907	-2.51691892	-1.33166	0.006309	0.019018
PTEN	113258976	-2.52333201	-1.33533	0.002867	0.009619
UBXN2B	113268080	-2.52501751	-1.33629	0.005363	0.016563
NUDT22	113244184	-2.5253225	-1.33647	0.000447	0.001896
ATP6V1B2	113270020	-2.52815114	-1.33808	0.002237	0.00776
ABCG2	113263158	-2.5290758	-1.33861	0.003808	0.012292
PSMD10	113241233	-2.53038972	-1.33936	0.000402	0.00173
CLP1	113246377	-2.53119862	-1.33982	0.002292	0.007929
MIER1	113254556	-2.53121193	-1.33983	0.001691	0.006083
RBPMS2	113240854	-2.53227735	-1.34044	0.001904	0.006735
RAN	113266861	-2.53256838	-1.3406	0.002345	0.008079
CRYBG1	113264779	-2.5331368	-1.34092	0.000984	0.003791
LOC113262286	113262286	-2.53380991	-1.34131	0.002602	0.008838
BTBD10	113246272	-2.53387911	-1.34135	0.00098	0.003782
HM13	113258458	-2.53435081	-1.34162	0.000433	0.001845
MINDY3	113241381	-2.53580859	-1.34245	0.000639	0.002607
SLC36A4	113259838	-2.53602973	-1.34257	0.009768	0.027388

ZNF276	113252124	-2.53696591	-1.3431	0.000821	0.003246
TNFRSF1A	113257988	-2.5378518	-1.34361	0.000285	0.001277
CBX5	113257522	-2.53996444	-1.34481	0.006481	0.019457
BCAP31	113247825	-2.54023606	-1.34496	0.011251	0.030846
FAM110A	113258436	-2.54212183	-1.34603	0.008605	0.024631
WAPL	113259244	-2.54282375	-1.34643	0.00399	0.012779
ARPC2	113250468	-2.54466958	-1.34748	0.007301	0.021471
ADAT1	113252505	-2.54517925	-1.34777	0.013542	0.036128
MGMT	113252060	-2.54730129	-1.34897	0.01039	0.028858
HHEX	113243171	-2.54887475	-1.34986	0.002114	0.007384
KLF13	113263608	-2.54976182	-1.35036	0.003031	0.010097
RNF40	113267695	-2.55051692	-1.35079	0.000308	0.001371
IRF2	113262255	-2.55162827	-1.35142	0.000202	0.000938
B9D2	113243539	-2.55316036	-1.35228	0.001836	0.006529
PGLS	113256683	-2.55498036	-1.35331	0.000622	0.002545
TLE3	113240924	-2.55567226	-1.3537	0.001865	0.006616
ALG9	113260062	-2.55568338	-1.35371	0.002346	0.008081
IFT140	113245082	-2.55662618	-1.35424	0.000803	0.003179
LOC113261234	113261234	-2.55704826	-1.35448	0.008851	0.025229
LOC113253952	113253952	-2.55867603	-1.3554	0.000134	0.000652
TGFBI	113261760	-2.5599001	-1.35609	0.010715	0.029566
HAPLN3	113263678	-2.5609689	-1.35669	0.006527	0.019577
LOC113262835	113262835	-2.56292548	-1.35779	0.004395	0.013888
LOC113264264	113264264	-2.56444712	-1.35865	0.009314	0.026315
RAB27B	113253438	-2.56458729	-1.35873	0.008689	0.024833
NUDT18	113270012	-2.5660593	-1.35955	0.002778	0.009378
GRK3	113244983	-2.56725062	-1.36022	0.005939	0.018069
SLC40A1	113250671	-2.56851509	-1.36093	0.012995	0.034949

PSAT1	113269827	-2.56853084	-1.36094	0.003901	0.012532
YWHAB	113258642	-2.56987878	-1.3617	0.006402	0.019248
ICMT	113270577	-2.57066775	-1.36214	0.000319	0.001411
SNRPF	113256135	-2.57282355	-1.36335	0.001323	0.004922
RALGPS2	113262778	-2.57368687	-1.36384	0.000279	0.001253
SAE1	113243381	-2.57382633	-1.36391	0.000251	0.001141
LOC113267225	113267225	-2.57598038	-1.36512	0.008287	0.023861
ATAD2B	113270373	-2.57671168	-1.36553	0.00098	0.003782
EBP	113265916	-2.57746232	-1.36595	0.001181	0.004453
URB2	113259050	-2.57778394	-1.36613	0.001176	0.004436
OSTM1	113264768	-2.57864187	-1.36661	0.000253	0.001148
GMNN	113264328	-2.58044729	-1.36762	0.001413	0.005212
CLTA	113260453	-2.58128929	-1.36809	0.000344	0.001507
SORT1	113244928	-2.58217567	-1.36859	0.005338	0.016497
TMEM151B	113261245	-2.58587704	-1.37065	0.006181	0.018709
BCL10	113254469	-2.586178	-1.37082	0.000337	0.001481
PICALM	113269398	-2.58734048	-1.37147	0.009674	0.02716
GBA	113245275	-2.58760591	-1.37162	0.001314	0.004894
PIK3R4	113253924	-2.58803451	-1.37186	0.000431	0.001836
GLMP	113245238	-2.59002624	-1.37297	0.000766	0.003054
SOAT1	113262770	-2.5912901	-1.37367	0.000748	0.002994
EMILIN1	113270943	-2.59370526	-1.37501	0.008466	0.024295
MSN	113246893	-2.59407851	-1.37522	0.009885	0.02763
NFE2L3	113261744	-2.59413297	-1.37525	0.017889	0.045748
LOC113256826	113256826	-2.59763608	-1.3772	0.011363	0.031109
SIAH2	113254062	-2.59983333	-1.37842	0.002126	0.007422
LOC113263782	113263782	-2.60054615	-1.37881	0.003634	0.011809
HMGB1	113251226	-2.60242506	-1.37986	0.018586	0.047273

UBE2J1	113264829	-2.60481706	-1.38118	0.000324	0.001429
BRMS1	113244068	-2.60529597	-1.38145	0.000359	0.001564
NUP50	113241663	-2.60532118	-1.38146	0.000405	0.001742
NRP2	113250549	-2.60567254	-1.38166	0.010637	0.029389
PAXBP1	113265272	-2.60571692	-1.38168	0.001677	0.00604
LOC113245466	113245466	-2.60925406	-1.38364	0.003129	0.010377
DNASE2	113247207	-2.61086255	-1.38453	0.000508	0.00213
ATP6V0C	113245138	-2.61236691	-1.38536	0.001449	0.005329
DOLPP1	113266497	-2.61629162	-1.38752	0.00033	0.001453
ANKRD13A	113267099	-2.61722251	-1.38804	0.000727	0.002919
DQX1	113243528	-2.61744776	-1.38816	0.012239	0.033152
LOC113246342	113246342	-2.62101884	-1.39013	0.000452	0.001916
KIF3B	113258478	-2.62113607	-1.39019	0.000394	0.001698
GDI1	113247862	-2.62161289	-1.39045	0.00304	0.010121
TNPO2	113247136	-2.62280623	-1.39111	0.000217	0.001005
MITD1	113246906	-2.62439848	-1.39199	0.008935	0.025402
MGAT2	113266192	-2.62599459	-1.39286	0.000229	0.001052
ARFGAP3	113241687	-2.62705498	-1.39345	0.001158	0.004375
LOC113248233	113248233	-2.62772052	-1.39381	0.001325	0.004925
LOC113246676	113246676	-2.62912228	-1.39458	0.000407	0.001748
MYL6	113256436	-2.62978987	-1.39495	0.011562	0.031596
L3MBTL2	113241736	-2.63079058	-1.3955	0.000231	0.001058
ATXN2L	113267629	-2.63095476	-1.39559	0.000457	0.00193
TNFRSF11A	113253483	-2.63171633	-1.396	0.009297	0.026279
CUNH7orf43	113267874	-2.63281203	-1.3966	0.001649	0.005959
ARF3	113257663	-2.63297216	-1.39669	0.000199	0.000925
SLC50A1	113245283	-2.63401265	-1.39726	0.000259	0.00117
SLX1A	113267643	-2.63417455	-1.39735	0.000841	0.003314

XRCC4	113255136	-2.63839905	-1.39966	0.007054	0.020853
NPC2	113270336	-2.63910245	-1.40005	0.002712	0.009177
CORO1C	113267074	-2.6408926	-1.40103	0.000256	0.001159
PELI1	113244986	-2.64205836	-1.40166	0.000614	0.002516
SEC24D	113255607	-2.642703	-1.40201	0.000789	0.00313
SLC38A1	113257702	-2.64417759	-1.40282	0.002228	0.007737
TM4SF18	113254047	-2.64441743	-1.40295	0.01144	0.031294
CAPN10	113249676	-2.64531395	-1.40344	0.000467	0.001972
BUB3	113252025	-2.64532407	-1.40344	0.0007	0.002824
ARFGEF1	113253077	-2.64542417	-1.4035	0.000453	0.001918
CLTC	113268890	-2.64688691	-1.4043	0.007404	0.021718
SUPT16H	113248472	-2.64814867	-1.40498	0.000535	0.002222
RPN2	113258575	-2.64918735	-1.40555	0.001604	0.005819
PASK	113249542	-2.64948697	-1.40571	0.001214	0.004562
DEGS1	113262903	-2.64971667	-1.40584	0.000359	0.001566
TTPAL	113258635	-2.65459192	-1.40849	0.0005	0.002099
EXOG	113253789	-2.65608159	-1.4093	0.001545	0.005636
RASGEF1B	113263224	-2.65704502	-1.40982	0.003323	0.010933
LOC113245454	113245454	-2.65887144	-1.41081	0.001298	0.004841
TACC1	113262084	-2.65966499	-1.41124	0.001704	0.006125
LOC113244531	113244531	-2.66070258	-1.41181	0.003451	0.011305
INTS6L	113249099	-2.66110046	-1.41202	0.001676	0.006038
CMAS	113257802	-2.6721174	-1.41798	0.000788	0.003129
SLC24A3	113258306	-2.67381921	-1.4189	0.008196	0.023642
SRSF7	113241564	-2.67383803	-1.41891	0.000175	0.00083
SERPINH1	113269331	-2.67454752	-1.41929	0.019501	0.04926
DGKG	113253969	-2.67628196	-1.42023	0.013518	0.036079
PTGDR2	113246980	-2.67629965	-1.42024	0.003435	0.011261

TMEM254	113259225	-2.67741965	-1.42084	0.000453	0.001917
ABCG1	113255375	-2.67880286	-1.42159	0.004265	0.013541
CSTF2	113244295	-2.6809439	-1.42274	0.000655	0.002663
OS9	113256368	-2.68250361	-1.42358	0.001973	0.006948
CUNH6orf62	113264330	-2.6825176	-1.42359	0.008873	0.025279
MICALL2	113247745	-2.6837582	-1.42425	0.002834	0.009528
LOC113263961	113263961	-2.68385402	-1.42431	0.000752	0.003006
RENBP	113247849	-2.68429965	-1.42455	0.0003	0.00134
HAUS6	113260595	-2.68536131	-1.42512	0.000266	0.0012
PLEKHO2	113240857	-2.68703488	-1.42602	0.000493	0.002075
SYNE3	113267304	-2.68711269	-1.42606	0.001077	0.004103
LIMK2	113245055	-2.68736625	-1.42619	0.000216	0.001
C1R	113257944	-2.68817706	-1.42663	0.00091	0.003545
LOC113250606	113250606	-2.69060307	-1.42793	0.006692	0.019963
GDAP2	113244368	-2.6913485	-1.42833	0.001142	0.004325
ABCB10	113259052	-2.69213744	-1.42875	0.008022	0.023265
RNF135	113268993	-2.69248867	-1.42894	0.001347	0.005
FAM184A	113259397	-2.69564361	-1.43063	0.013394	0.03581
ZBTB42	113267191	-2.6965404	-1.43111	0.002318	0.008
GALNT12	113260408	-2.69756655	-1.43166	0.000225	0.001036
SLC10A7	113255729	-2.70157577	-1.4338	0.000856	0.003367
LOC113266293	113266293	-2.70222629	-1.43415	0.013703	0.0365
SPG21	113240860	-2.70285158	-1.43448	0.000123	0.000601
IFNAR1	113264796	-2.704744	-1.43549	0.001071	0.004085
NEMP2	113250657	-2.70497155	-1.43561	0.000236	0.001079
SLC6A6	113257221	-2.70558914	-1.43594	0.000775	0.003086
RYR3	113242078	-2.70656263	-1.43646	0.009204	0.026064
SPHK2	113243339	-2.70904747	-1.43779	0.001825	0.0065

EML4	113241799	-2.70943356	-1.43799	0.000434	0.001846
PRPS2	113240731	-2.71251902	-1.43963	0.000184	0.000868
LIN52	113270348	-2.71309105	-1.43994	0.000522	0.002181
TAOK3	113267022	-2.71700577	-1.44202	0.000166	0.000788
BMP1	113270006	-2.7184142	-1.44277	0.004375	0.013834
TBC1D31	113252760	-2.71882435	-1.44298	0.00015	0.000721
PNMA1	113270356	-2.71898722	-1.44307	0.009524	0.026811
MAPK14	113243852	-2.72047795	-1.44386	0.003319	0.010924
TMEM160	113243393	-2.72055953	-1.4439	0.000424	0.001809
ACSL4	113241237	-2.72398608	-1.44572	0.002816	0.009484
LOC113246848	113246848	-2.72469319	-1.44609	0.005567	0.017078
KIF2A	113255297	-2.72531451	-1.44642	0.00014	0.000676
MCMBP	113252003	-2.72593857	-1.44675	0.000295	0.001318
FAM129C	113256685	-2.72692747	-1.44728	0.001989	0.006997
RAB31	113253214	-2.72868491	-1.44821	0.004502	0.014193
ADAM10	113269679	-2.73065677	-1.44925	0.000125	0.000613
NPRL3	113245355	-2.73228705	-1.45011	0.00062	0.002537
SPATA1	113254480	-2.73268462	-1.45032	0.003175	0.010508
PCNX4	113242395	-2.73280571	-1.45038	0.000876	0.003428
GBE1	113247670	-2.73367942	-1.45084	0.001783	0.006368
DPY19L3	113244716	-2.73414519	-1.45109	0.000243	0.001107
ATOX1	113263981	-2.73661619	-1.45239	0.000117	0.000578
NCBP3	113269164	-2.73709904	-1.45265	0.000534	0.00222
LOC113249352	113249352	-2.73970248	-1.45402	0.007005	0.020728
FBXL6	113247686	-2.73982008	-1.45408	0.000685	0.002769
TMED3	113263787	-2.74109631	-1.45475	0.002343	0.008075
CFAP410	113251792	-2.74193816	-1.4552	0.005397	0.016638
EME2	113245103	-2.74203567	-1.45525	0.008159	0.023566

TRADD	113252355	-2.74359483	-1.45607	0.000109	0.000541
EIF4A1	113271150	-2.74379352	-1.45617	0.003711	0.012015
PGGHG	113248810	-2.74781999	-1.45829	0.000839	0.003307
LRRK2	113257729	-2.75084146	-1.45987	0.001489	0.005461
ENC1	113255212	-2.75092597	-1.45992	0.000215	0.000994
GAK	113245708	-2.75105779	-1.45999	0.000116	0.000575
RAB11FIP1	113262102	-2.75196221	-1.46046	0.000955	0.003699
TTLL7	113254488	-2.75207417	-1.46052	0.003757	0.012144
ADD3	113251916	-2.75211098	-1.46054	0.000686	0.002772
KLF16	113242658	-2.7526924	-1.46084	0.00011	0.000548
CNPPD1	113250439	-2.75343763	-1.46123	0.000298	0.001331
FOXRED2	113255989	-2.75510319	-1.46211	0.000722	0.002903
LOC113249657	113249657	-2.75512116	-1.46212	0.004933	0.015411
LOC113269409	113269409	-2.75611376	-1.46264	0.007111	0.021002
NECAP2	113270452	-2.75664416	-1.46291	8.69E-05	0.000442
ARHGAP42	113260156	-2.76020268	-1.46477	0.001656	0.005978
MAGI2	113265932	-2.76149908	-1.46545	0.01814	0.046296
HMGCR	113255200	-2.76353364	-1.46651	0.001354	0.005019
CYTIP	113250866	-2.76432265	-1.46693	0.002549	0.008693
KCND1	113265939	-2.76659408	-1.46811	0.00921	0.026075
SLC12A3	113252268	-2.76695008	-1.4683	0.009527	0.026811
POGLUT1	113251857	-2.76770633	-1.46869	0.000182	0.00086
LOC113256804	113256804	-2.76812269	-1.46891	0.004587	0.014439
EP300	113241737	-2.768643	-1.46918	0.006055	0.018376
LOC113254891	113254891	-2.76970469	-1.46973	0.0096	0.026988
WDPCP	113245028	-2.77334954	-1.47163	0.004738	0.014863
KDM6B	113271139	-2.7760002	-1.47301	0.001612	0.00584
POLH	113261257	-2.7761757	-1.4731	0.000648	0.002637

KCTD11	113271130	-2.77682818	-1.47344	0.000541	0.002245
KLF5	113251418	-2.7801368	-1.47516	0.005393	0.016635
ADSS	113262960	-2.78155563	-1.47589	0.000201	0.000936
MIF4GD	113244642	-2.78359746	-1.47695	0.000137	0.000663
LOC113246662	113246662	-2.78404375	-1.47718	0.01925	0.048781
MRC2	113265302	-2.7843881	-1.47736	0.013442	0.035918
GLB1L	113250436	-2.78698477	-1.47871	0.001952	0.006883
ABCB6	113250434	-2.78830209	-1.47939	0.005839	0.017821
LOC113257933	113257933	-2.78943444	-1.47997	0.002293	0.00793
CCDC15	113259910	-2.79245583	-1.48153	0.000749	0.002994
ARL4A	113260801	-2.79327286	-1.48196	0.000791	0.003137
LRCH4	113267852	-2.79451259	-1.4826	0.000661	0.002682
CBX2	113244545	-2.79480271	-1.48275	0.009789	0.027434
TMEM135	113269408	-2.79809446	-1.48444	0.001788	0.006384
IL7	113253022	-2.79916278	-1.485	0.010266	0.028562
GUSB	113267745	-2.80005787	-1.48546	0.000152	0.00073
ACBD7	113241387	-2.80092555	-1.4859	0.007254	0.021351
BACE2	113256938	-2.80274918	-1.48684	0.000112	0.000557
B3GNT2	113245108	-2.80361261	-1.48729	0.000239	0.001092
SMAP2	113268231	-2.80496447	-1.48798	0.001432	0.005274
LOC113265665	113265665	-2.80626653	-1.48865	0.011616	0.031717
USP1	113254591	-2.80727192	-1.48917	0.000162	0.000773
LOC113253800	113253800	-2.81112429	-1.49115	0.001645	0.00595
STS	113240763	-2.8113972	-1.49129	0.019354	0.04897
PAN2	113256422	-2.81563657	-1.49346	0.000579	0.002391
TRIM27	113248142	-2.81657174	-1.49394	0.000186	0.000875
GEM	113252928	-2.81738996	-1.49436	0.004316	0.013677
CCP110	113270733	-2.81812686	-1.49474	0.000583	0.002408

LCMT2	113241970	-2.81841563	-1.49488	0.00639	0.019219
UCK2	113268697	-2.81918508	-1.49528	0.000229	0.001048
CEP135	113262516	-2.82260316	-1.49703	0.000777	0.003092
MOB1A	113243805	-2.82282583	-1.49714	0.001683	0.00606
CUNH21orf91	113243447	-2.82408944	-1.49779	2.2E-05	0.000128
SPEF1	113258387	-2.82470392	-1.4981	0.009489	0.026728
SERINC5	113255162	-2.8248308	-1.49816	0.00011	0.000546
STAG2	113270930	-2.82754176	-1.49955	0.003513	0.011481
PJVK	113250715	-2.82858452	-1.50008	0.015306	0.040152
GNB1	113270618	-2.82950455	-1.50055	0.002229	0.007741
SKA2	113268883	-2.83361069	-1.50264	0.000533	0.002217
CAMKK2	113266952	-2.83743158	-1.50459	0.013413	0.035848
P2RX4	113266953	-2.83760886	-1.50468	0.000361	0.001572
TMEM260	113242335	-2.83783175	-1.50479	8.89E-05	0.000451
USP32	113268909	-2.83803328	-1.50489	0.000907	0.003536
PPP6R1	113247377	-2.83843686	-1.5051	0.000565	0.002336
TRAM2	113261193	-2.83950574	-1.50564	0.000228	0.001045
LOC113271103	113271103	-2.84004276	-1.50591	0.009884	0.02763
GRAMD4	113241638	-2.84163436	-1.50672	0.000246	0.001119
HEXIM2	113265327	-2.84521228	-1.50854	0.004749	0.01489
CDK18	113242443	-2.84541058	-1.50864	0.00613	0.018578
GGH	113268062	-2.84544808	-1.50866	0.000151	0.000724
ORC5	113258845	-2.84831636	-1.51011	0.000513	0.002148
GDPD5	113269330	-2.8490393	-1.51048	0.012563	0.033896
LMF2	113241605	-2.84919299	-1.51055	0.000198	0.000924
STEAP3	113263450	-2.84943462	-1.51068	0.00021	0.000971
GIT2	113267098	-2.85203281	-1.51199	0.000303	0.001349
SEC62	113255273	-2.85213224	-1.51204	0.001806	0.006441

STK38	113243862	-2.85344861	-1.51271	0.000115	0.000568
PPP1R9B	113265610	-2.85522601	-1.5136	0.000508	0.00213
SBNO2	113242718	-2.8567904	-1.5144	0.000122	0.000596
LYVE1	113245789	-2.85681242	-1.51441	0.01602	0.041734
CEP192	113245976	-2.85706073	-1.51453	0.000253	0.001146
RNF213	113244535	-2.85999863	-1.51601	0.000409	0.001754
TMEM176B	113241471	-2.86571933	-1.5189	0.000392	0.001691
PPP2R1B	113260072	-2.86643968	-1.51926	6.32E-05	0.000332
ODF2	113266470	-2.8711565	-1.52163	0.000169	0.000804
PLVAP	113256677	-2.87130581	-1.52171	0.000217	0.001002
CDC42SE1	113246132	-2.87300918	-1.52256	0.000183	0.000862
CLDN7	113271113	-2.87456833	-1.52335	0.013736	0.036566
ACAT2	113259619	-2.87576905	-1.52395	0.000121	0.000592
ATP11B	113254601	-2.87767914	-1.52491	0.003959	0.012694
LOC113252395	113252395	-2.88123896	-1.52669	0.001354	0.005019
EEF1AKMT3	113256362	-2.88467376	-1.52841	0.008413	0.024166
KIF27	113269811	-2.88506011	-1.5286	0.00322	0.010635
ARRDC5	113242849	-2.88554365	-1.52884	0.009286	0.026253
FAM126A	113261337	-2.88670453	-1.52942	0.000133	0.000646
LOC113267063	113267063	-2.8876333	-1.52989	2.76E-05	0.000157
SAG	113250331	-2.88788323	-1.53001	0.008531	0.024439
UNG	113267082	-2.88875786	-1.53045	0.00015	0.00072
ODC1	113269829	-2.88920752	-1.53067	0.000151	0.000724
ZNRF2	113262287	-2.8931378	-1.53264	0.00039	0.001684
LOC113247073	113247073	-2.89362921	-1.53288	0.001269	0.004747
MCM9	113259395	-2.89420294	-1.53317	0.00064	0.002609
CUNH20orf96	113258451	-2.89485857	-1.53349	0.006472	0.019433
LIME1	113258819	-2.89621526	-1.53417	0.008009	0.023243

HEXB	113255209	-2.89876734	-1.53544	0.000118	0.00058
SLC29A3	113259150	-2.89977334	-1.53594	0.003176	0.010509
PSMB8	113243782	-2.90058263	-1.53634	0.000135	0.000655
AMIGO3	113256883	-2.9006438	-1.53637	0.001019	0.003916
MTA2	113246959	-2.90103366	-1.53657	0.000121	0.000595
POLD3	113269314	-2.90122144	-1.53666	0.000623	0.002545
NEK8	113269046	-2.90152579	-1.53681	0.004669	0.014682
S100A11	113246225	-2.90175847	-1.53693	0.00048	0.002024
VDR	113257683	-2.90199108	-1.53704	0.015881	0.041481
LRRC59	113265621	-2.90530217	-1.53869	0.000165	0.000786
CLPTM1L	113260789	-2.90547681	-1.53877	9.08E-05	0.000459
TDP1	113267370	-2.90793999	-1.54	0.000868	0.003405
INTS10	113245552	-2.90908332	-1.54056	3.01E-05	0.000169
RGS8	113262729	-2.90912844	-1.54059	0.005266	0.016298
PKHD1L1	113252814	-2.90919762	-1.54062	0.003054	0.010163
NUP43	113259549	-2.90938743	-1.54072	0.000296	0.00132
CCDC14	113252401	-2.91293679	-1.54247	0.001393	0.005149
DNAJC13	113253939	-2.91538365	-1.54369	0.00105	0.004018
QPCTL	113243418	-2.9189876	-1.54547	0.0002	0.00093
LOC113248523	113248523	-2.92027896	-1.54611	0.003061	0.010182
LOC113258529	113258529	-2.9204038	-1.54617	9.3E-06	5.92E-05
TXNDC5	113264432	-2.92149312	-1.54671	0.000281	0.001259
MOCS1	113243900	-2.92570963	-1.54879	1.9E-05	0.000112
LOC113246330	113246330	-2.92968546	-1.55075	0.006989	0.020687
LRRC45	113244488	-2.92991672	-1.55086	8.94E-05	0.000453
PTX3	113254114	-2.93259339	-1.55218	0.014275	0.037816
FBXL19	113267707	-2.93592157	-1.55381	0.001055	0.004034
LOC113262782	113262782	-2.93749866	-1.55459	0.000515	0.002155

TMCO1	113268694	-2.93978108	-1.55571	1.18E-05	7.34E-05
G2E3	113266090	-2.94297435	-1.55727	9.88E-05	0.000496
ITSN1	113263634	-2.94341414	-1.55749	0.000217	0.001002
ARMC5	113267725	-2.94698593	-1.55924	0.000217	0.001004
PGM3	113261065	-2.94760555	-1.55954	0.000297	0.001325
PARP9	113252262	-2.95013675	-1.56078	2.38E-05	0.000137
CASP8AP2	113264820	-2.95043808	-1.56093	0.000528	0.002199
ARSA	113241595	-2.95223445	-1.56181	0.000223	0.001029
PPIB	113240844	-2.95316904	-1.56226	0.002612	0.008863
WDR34	113266476	-2.95505892	-1.56319	7.43E-05	0.000383
TUBE1	113264717	-2.9553361	-1.56332	0.008215	0.023677
TBC1D5	113253896	-2.95706674	-1.56417	0.000131	0.000639
DYNLL1	113267044	-2.96154835	-1.56635	0.000111	0.000553
HFE	113264259	-2.96175132	-1.56645	2.52E-05	0.000144
UHRF2	113260624	-2.96361509	-1.56736	0.000151	0.000724
LOC113252869	113252869	-2.96398529	-1.56754	0.000995	0.003833
MPHOSPH9	113266905	-2.96696756	-1.56899	0.000125	0.000613
LOC113250446	113250446	-2.9677689	-1.56938	0.009372	0.026439
TRARG1	113269092	-2.96924043	-1.57009	0.010713	0.029566
CTSA	113258664	-2.97169744	-1.57129	5.16E-05	0.000276
BAZ1A	113266121	-2.97502885	-1.5729	0.000855	0.003364
AP3B1	113255176	-2.9759875	-1.57337	3.55E-05	0.000196
RNF26	113259961	-2.97653693	-1.57363	4.04E-05	0.00022
DTNB	113270659	-2.97684046	-1.57378	0.001388	0.005134
AATK	113244526	-2.97762145	-1.57416	0.001834	0.006524
PDE8B	113255180	-2.97798959	-1.57434	0.00905	0.025676
ACSF2	113265618	-2.97910158	-1.57488	0.004861	0.015197
SLC2A10	113258680	-2.98099284	-1.57579	0.009212	0.026076

KLF3	113262407	-2.98172147	-1.57615	0.000165	0.000787
LRWD1	113267820	-2.98269695	-1.57662	9.94E-05	0.000499
SNX30	113266299	-2.98324933	-1.57688	0.000558	0.002313
PTH1R	113256800	-2.98417675	-1.57733	0.015172	0.039873
ELMO3	113252348	-2.98484297	-1.57766	0.003532	0.011536
AP1B1	113245013	-2.98640498	-1.57841	0.00069	0.002788
SENP1	113257680	-2.98950084	-1.5799	0.000107	0.000532
SUV39H1	113265922	-2.9909402	-1.5806	0.000466	0.001969
TM7SF3	113257779	-2.99387442	-1.58201	5.87E-05	0.00031
CHEK2	113244997	-2.99389275	-1.58202	0.004312	0.013669
HDHD3	113266317	-2.99397506	-1.58206	0.004276	0.013567
STK17A	113258037	-2.99691615	-1.58348	4.31E-05	0.000233
JDP2	113270315	-3.00002597	-1.58497	8.84E-05	0.000449
LYST	113259277	-3.00233513	-1.58609	0.001711	0.006144
KLHL2	113255823	-3.00407371	-1.58692	4.86E-05	0.000261
GNAI2	113256902	-3.00440226	-1.58708	0.003653	0.011857
SLC18A2	113251976	-3.0053329	-1.58752	0.001857	0.006592
LOC113248746	113248746	-3.00535556	-1.58754	0.016792	0.04339
MAFG	113244494	-3.00633962	-1.58801	0.00151	0.005528
EDEM1	113257120	-3.009666	-1.5896	2.99E-05	0.000168
SDC1	113270259	-3.01003073	-1.58978	0.009539	0.02684
RBP4	113243178	-3.01014679	-1.58983	0.011434	0.031284
TMEM63A	113262913	-3.01424637	-1.5918	1.13E-05	7.03E-05
UNC93B1	113244011	-3.01494652	-1.59213	2.99E-05	0.000168
FAM241B	113259131	-3.01696594	-1.5931	0.002255	0.007816
MKRN1	113265080	-3.01885134	-1.594	0.001034	0.003964
ROCK1	113253267	-3.01899926	-1.59407	0.000129	0.000627
CBS	113254145	-3.02077054	-1.59492	0.002237	0.00776

ASAP1	113252718	-3.02280441	-1.59589	9.98E-05	0.000501
SNCA	113263144	-3.0269953	-1.59789	0.003887	0.012498
BCR	113247026	-3.02705355	-1.59791	7.77E-05	0.000399
PLA2R1	113250847	-3.02713449	-1.59795	0.000183	0.000862
ZDHHC20	113251179	-3.02882123	-1.59876	4.59E-05	0.000248
TRAPPC6A	113243441	-3.02982339	-1.59923	0.000264	0.001194
SLC37A1	113255147	-3.03244043	-1.60048	0.001943	0.006855
SYNJ1	113265426	-3.03309295	-1.60079	4.38E-05	0.000237
BANK1	113263104	-3.0332397	-1.60086	0.001057	0.004039
CXXC1	113253415	-3.03399363	-1.60122	2.78E-05	0.000158
SFT2D1	113259642	-3.03417217	-1.6013	3.41E-05	0.000189
SLC35D1	113254557	-3.03497188	-1.60168	0.000526	0.002194
ARHGEF2	113245255	-3.0392731	-1.60373	0.001328	0.004938
THG1L	113264024	-3.04279818	-1.6054	0.000792	0.003137
PDE1B	113257511	-3.04302535	-1.60551	0.002166	0.007552
PROSER2	113241063	-3.04397193	-1.60596	0.009584	0.026961
DHCR7	113243974	-3.04429048	-1.60611	0.000127	0.000619
FBXO48	113244705	-3.04433626	-1.60613	0.000864	0.003392
NASP	113254738	-3.04497077	-1.60643	1.62E-05	9.74E-05
SACM1L	113256778	-3.04583227	-1.60684	3.02E-05	0.000169
SRSF9	113267041	-3.04677647	-1.60728	3.25E-05	0.000181
CD14	113261829	-3.0468291	-1.60731	4.27E-05	0.000231
ARHGAP23	113265689	-3.04707953	-1.60743	0.0007	0.002824
ABHD15	113269074	-3.0511142	-1.60934	0.00056	0.002321
OGG1	113257143	-3.05480597	-1.61108	0.003333	0.010958
MRTFA	113241747	-3.05497601	-1.61116	0.000438	0.001861
ILDR2	113262892	-3.05845779	-1.6128	0.01484	0.039122
COL4A5	113241173	-3.05894673	-1.61303	0.0005	0.0021

CEP76	113245893	-3.0592673	-1.61319	0.000589	0.002428
RCC1	113268413	-3.06071026	-1.61387	2.22E-05	0.000129
LOC113271280	113271280	-3.06357696	-1.61522	0.006826	0.020281
TCHP	113267096	-3.06718498	-1.61692	0.000381	0.00165
PHACTR1	113264462	-3.06778276	-1.6172	5.8E-05	0.000307
SCML1	113242261	-3.07013377	-1.6183	0.000222	0.001026
JPT1	113244646	-3.0706027	-1.61852	1.8E-05	0.000107
FKBP5	113243846	-3.07123546	-1.61882	0.000671	0.002721
BID	113258065	-3.07233189	-1.61933	4.66E-05	0.000251
KCNN1	113256697	-3.07697772	-1.62151	0.002123	0.007413
ILK	113245818	-3.07790396	-1.62195	0.000321	0.001418
MARK2	113244194	-3.07971299	-1.6228	5.67E-05	0.0003
LGMN	113267343	-3.08072241	-1.62327	0.000975	0.003766
SEMA4F	113243445	-3.08256813	-1.62413	3.12E-05	0.000174
MAP3K5	113259481	-3.08427963	-1.62493	0.000118	0.000583
FBP1	113269785	-3.08545359	-1.62548	0.00765	0.022332
MOGS	113243493	-3.08755062	-1.62646	1.92E-05	0.000113
RCOR1	113267236	-3.08762232	-1.6265	6.44E-05	0.000337
CNOT6	113261545	-3.08773505	-1.62655	0.000105	0.000526
TPCN2	113243987	-3.0881054	-1.62672	3.24E-05	0.000181
TIRAP	113259882	-3.08838616	-1.62685	7.09E-05	0.000368
CHN2	113262210	-3.08849625	-1.6269	0.001556	0.005672
CASP10	113250594	-3.0939805	-1.62946	0.000145	0.000697
SEC61B	113260404	-3.09562774	-1.63023	2.54E-05	0.000145
NARF	113244470	-3.09729687	-1.63101	0.000438	0.001861
NUAK2	113242489	-3.09736702	-1.63104	0.003084	0.010246
OAT	113252032	-3.10060113	-1.63255	0.000334	0.001468
CD40	113258674	-3.10186013	-1.63313	0.002791	0.009416

AGAP2	113256365	-3.10192332	-1.63316	0.001501	0.005504
ARSB	113255173	-3.10224605	-1.63331	1.75E-05	0.000104
VAMP8	113243140	-3.10253771	-1.63345	7.08E-05	0.000368
GRK2	113244040	-3.10291645	-1.63362	0.000878	0.003435
CHP1	113242012	-3.1034644	-1.63388	0.001157	0.004373
UNC5A	113264134	-3.10487683	-1.63454	0.000766	0.003053
ADGRG7	113250587	-3.10687479	-1.63546	0.009896	0.027654
TDG	113256064	-3.10891701	-1.63641	7.82E-05	0.000401
TKFC	113246914	-3.10924512	-1.63656	1.83E-05	0.000109
GPR108	113242887	-3.11165969	-1.63768	3.7E-06	2.55E-05
SUCO	113262826	-3.11322708	-1.63841	5.6E-05	0.000296
MAN2A1	113255007	-3.11779176	-1.64052	6.88E-05	0.000358
LOC113267043	113267043	-3.12042254	-1.64174	0.000442	0.001878
ABCA6	113265232	-3.12530742	-1.644	4.01E-05	0.000218
CEP57	113259811	-3.12704482	-1.6448	1.35E-05	8.24E-05
PRIM2	113261153	-3.12784235	-1.64517	4.15E-05	0.000226
BMP2K	113263235	-3.12884139	-1.64563	0.000252	0.001145
C4BPA	113242607	-3.13181976	-1.647	0.006544	0.019613
ATP13A3	113253389	-3.13311132	-1.6476	2.93E-05	0.000165
MAD2L1BP	113261255	-3.13336306	-1.64771	0.000331	0.001457
TFPI	113250680	-3.13341201	-1.64773	0.000277	0.001246
UBE2S	113247425	-3.13517486	-1.64855	0.000314	0.001392
SHC4	113269597	-3.13669301	-1.64924	0.00822	0.023687
SPINT2	113243624	-3.13894885	-1.65028	0.000154	0.000738
ADORA1	113242522	-3.14101663	-1.65123	0.019737	0.049737
FAM216A	113266961	-3.14170981	-1.65155	0.002715	0.009184
RFC4	113253907	-3.14432625	-1.65275	0.000136	0.000657
SHMT2	113256389	-3.14551028	-1.65329	3.31E-05	0.000184

GMPPB	113256885	-3.14619245	-1.65361	7.52E-05	0.000388
PDCD4	113251928	-3.14674697	-1.65386	0.000128	0.000625
RAB39B	113248333	-3.1509963	-1.65581	0.013175	0.035321
BRPF1	113257137	-3.15501492	-1.65765	7.18E-05	0.000372
CCDC142	113243499	-3.15844281	-1.65921	0.001216	0.004569
NAGA	113241706	-3.16001918	-1.65993	1.24E-05	7.69E-05
ZSCAN25	113267892	-3.16161137	-1.66066	0.004051	0.012953
ITPK1	113267338	-3.16168131	-1.66069	6.12E-06	4.05E-05
CERS6	113250813	-3.16375006	-1.66164	0.000192	0.000899
USP6NL	113241059	-3.16628223	-1.66279	9.08E-05	0.000459
SH3BGRL3	113268465	-3.16687356	-1.66306	6.69E-05	0.000349
TMX1	113266223	-3.17116617	-1.66501	8.19E-05	0.000419
SRBD1	113246176	-3.17324606	-1.66596	1.05E-05	6.62E-05
B3GNT8	113243533	-3.17354185	-1.66609	0.001152	0.004359
LOC113246677	113246677	-3.17418132	-1.66638	2.93E-06	2.06E-05
RSPH3	113259337	-3.17585428	-1.66714	0.005411	0.01667
PIGQ	113245379	-3.17658138	-1.66747	0.000164	0.000779
FAM91A1	113252750	-3.17868076	-1.66843	1.77E-05	0.000105
PRR7	113264154	-3.17870391	-1.66844	0.007143	0.021076
INTS7	113262669	-3.17947317	-1.66879	6.4E-05	0.000335
TIGD2	113263147	-3.18095743	-1.66946	3.05E-05	0.000171
AOC2	113265394	-3.18442588	-1.67103	7.17E-05	0.000372
PTGIR	113243392	-3.1865215	-1.67198	0.005683	0.017394
KCTD12	113251429	-3.1876563	-1.6725	0.005545	0.017027
TEKT5	113270804	-3.18841585	-1.67284	0.001531	0.005595
FAM117B	113250575	-3.19023369	-1.67366	0.000129	0.00063
GTF3C3	113250636	-3.19121738	-1.67411	4.18E-05	0.000227
CUNH16orf58	113267727	-3.1968889	-1.67667	9.36E-06	5.94E-05

ACAP2	113253318	-3.19690332	-1.67668	1.71E-05	0.000102
ATP10D	113262460	-3.19708525	-1.67676	3.73E-05	0.000205
CBX4	113244543	-3.19760266	-1.67699	0.00041	0.001759
CERS2	113246156	-3.19919781	-1.67771	1.76E-05	0.000104
FGL1	113262155	-3.19920223	-1.67771	8.84E-05	0.000449
MAPRE1	113258485	-3.19958149	-1.67788	1.33E-05	8.17E-05
RAB7B	113242471	-3.19983406	-1.678	0.01246	0.033652
HGF	113259246	-3.20027956	-1.6782	0.001514	0.00554
KIAA1147	113265223	-3.20282734	-1.67935	0.000231	0.001058
SUN2	113241768	-3.20439939	-1.68005	0.001076	0.004102
SEC61A1	113257265	-3.20550966	-1.68055	0.000931	0.003618
TREX2	113247856	-3.20630553	-1.68091	0.003737	0.012089
NR1H3	113264967	-3.21211495	-1.68352	0.000679	0.002751
LOC113246261	113246261	-3.21274904	-1.68381	0.000345	0.001511
LRRCC1	113252991	-3.21590634	-1.68523	2.63E-06	1.86E-05
SLC20A1	113242501	-3.21743172	-1.68591	0.000322	0.001422
CMPK2	113269399	-3.21830337	-1.6863	0.000172	0.000818
STEAP4	113259574	-3.21859173	-1.68643	0.000134	0.000649
TP53INP1	113252917	-3.22072927	-1.68739	0.000118	0.00058
CCDC170	113259566	-3.22348905	-1.68862	0.00011	0.000546
UNC13D	113244621	-3.22537686	-1.68947	0.005521	0.016977
UHRF1BP1L	113256105	-3.22572922	-1.68963	2.28E-05	0.000132
CEBPD	113268122	-3.22881956	-1.69101	0.000162	0.000773
STAT2	113256419	-3.23110346	-1.69203	1.14E-05	7.09E-05
RAB19	113265067	-3.2335464	-1.69312	0.012827	0.034559
CNPY3	113261285	-3.23581691	-1.69413	1.67E-05	9.99E-05
SMPD5	113247627	-3.23621117	-1.69431	0.001165	0.004399
ULK4	113253814	-3.23631618	-1.69435	0.000242	0.001102

IL6R	113245310	-3.23727906	-1.69478	0.000157	0.000749
SMC3	113251924	-3.24144399	-1.69664	0.000273	0.001229
FAM102A	113266447	-3.24168286	-1.69674	0.000239	0.001091
LOC113250012	113250012	-3.24257634	-1.69714	0.005745	0.017561
ARHGAP25	113244661	-3.24407774	-1.69781	5.29E-05	0.000282
HYAL1	113256908	-3.24865951	-1.69984	0.000464	0.00196
PPIL1	113243869	-3.24890776	-1.69995	1.54E-05	9.3E-05
HAUS8	113256663	-3.25108971	-1.70092	8.31E-05	0.000425
TLN1	113260465	-3.25364226	-1.70206	0.013517	0.036079
CFLAR	113250596	-3.25568264	-1.70296	2.55E-05	0.000146
RECQL	113257809	-3.25940138	-1.70461	2.41E-05	0.000138
DAPK1	113269788	-3.26206549	-1.70579	0.003228	0.010653
LOC113257855	113257855	-3.26230309	-1.70589	9.38E-06	5.95E-05
KIF22	113267670	-3.26405603	-1.70667	7.75E-05	0.000398
GJD3	113265489	-3.26493274	-1.70705	0.003231	0.010657
RB1	113251325	-3.26669544	-1.70783	0.000149	0.000715
NUBP1	113270807	-3.26872904	-1.70873	4.28E-06	2.92E-05
F2RL3	113256662	-3.2711304	-1.70979	0.011375	0.031136
KCNAB3	113271148	-3.27823976	-1.71292	0.006237	0.01884
STK17B	113250640	-3.27851745	-1.71304	0.000795	0.003149
IRF1	113261712	-3.27903276	-1.71327	6.29E-06	4.16E-05
XDH	113240940	-3.2818721	-1.71452	0.005268	0.016301
PQLC3	113269928	-3.28447175	-1.71566	4.42E-05	0.000239
MGAT4A	113246802	-3.28939528	-1.71782	0.000905	0.00353
PTBP1	113242704	-3.28964301	-1.71793	0.000746	0.002987
FANCE	113243843	-3.28969556	-1.71795	0.001408	0.005197
AP3B2	113263743	-3.29044752	-1.71828	0.002009	0.007062
MYH9	113255994	-3.29226815	-1.71908	0.017115	0.044119

INHBA	113258079	-3.29258809	-1.71922	0.019011	0.048221
SESN2	113268419	-3.2929895	-1.7194	0.004047	0.012944
TMTC4	113251505	-3.2931891	-1.71949	0.000624	0.002548
STAB1	113256956	-3.29649841	-1.72093	5.52E-05	0.000293
MDGA1	113243883	-3.29906968	-1.72206	0.009109	0.025817
SCN5A	113253790	-3.29998938	-1.72246	0.007209	0.021242
NID2	113242379	-3.30302678	-1.72379	0.004382	0.013849
CAPN15	113245375	-3.30601891	-1.72509	2.64E-05	0.000151
ADAM12	113252043	-3.30793476	-1.72593	0.017309	0.044518
PPT1	113268238	-3.30796979	-1.72595	4.7E-06	3.19E-05
EFCAB11	113267372	-3.30998405	-1.72682	0.001562	0.00569
ELL2	113255072	-3.31011359	-1.72688	1.4E-06	1.04E-05
PYGB	113248361	-3.31484283	-1.72894	2.21E-05	0.000128
ESYT1	113256437	-3.32193716	-1.73202	0.00033	0.001453
LOC113270441	113270441	-3.32448725	-1.73313	0.002978	0.009964
TMED8	113270291	-3.32484321	-1.73329	1.1E-05	6.87E-05
ADA	113258634	-3.32519005	-1.73344	2.53E-05	0.000145
ACTG1	113244519	-3.32810878	-1.7347	0.016582	0.042924
DFFB	113270585	-3.32983599	-1.73545	0.001087	0.004138
PNKP	113243279	-3.33119395	-1.73604	2.3E-05	0.000133
LOC113243704	113243704	-3.33206479	-1.73642	0.012029	0.032683
ATP6AP2	113265872	-3.33666642	-1.73841	8.07E-06	5.2E-05
CARD11	113267978	-3.337599	-1.73881	0.001479	0.005428
PARVB	113241671	-3.33916242	-1.73949	0.001296	0.004834
CREB3L1	113264990	-3.34039503	-1.74002	0.00967	0.02716
HAUS7	113247855	-3.34056165	-1.74009	0.000427	0.00182
CCDC150	113250637	-3.34162009	-1.74055	0.009038	0.025648
LOC113248623	113248623	-3.34229234	-1.74084	0.001652	0.005969

HAUS2	113241985	-3.34736956	-1.74303	0.000505	0.002119
NEU1	113243747	-3.34794645	-1.74328	2.09E-05	0.000122
CDKN2D	113242994	-3.34905122	-1.74375	0.000828	0.00327
ANAPC1	113242372	-3.35012998	-1.74422	0.000149	0.000716
TBC1D2B	113263575	-3.35207852	-1.74506	6.42E-05	0.000336
DDX39A	113247151	-3.3575326	-1.7474	0.000108	0.000536
LOC113251291	113251291	-3.3621949	-1.7494	0.005205	0.016143
HCAR1	113266918	-3.36356239	-1.74999	0.005386	0.016621
ZBTB34	113266420	-3.36423753	-1.75028	8.7E-05	0.000443
CCDC112	113261601	-3.36932309	-1.75246	0.000441	0.001872
PDE3B	113246251	-3.37173416	-1.75349	1.9E-05	0.000112
CDH16	113252336	-3.37866495	-1.75645	0.000586	0.002414
PDIA3	113241953	-3.37967043	-1.75688	0.000407	0.001748
TLR2	113255769	-3.37968442	-1.75689	0.000432	0.00184
KIAA0556	113267614	-3.38038751	-1.75719	6.61E-06	4.34E-05
RIPK1	113264397	-3.38180241	-1.75779	6.68E-06	4.39E-05
RAP2A	113251482	-3.38279036	-1.75821	9.29E-06	5.91E-05
LOC113256790	113256790	-3.38632485	-1.75972	0.002017	0.007084
PTMA	113250347	-3.38772559	-1.76032	0.011041	0.030349
DESI1	113241723	-3.38897336	-1.76085	1.12E-05	7.02E-05
CDKN2C	113254692	-3.39238881	-1.7623	2.29E-06	1.63E-05
MMP23B	113270624	-3.3938363	-1.76292	0.008479	0.024315
GSAP	113259069	-3.39458506	-1.76324	3.39E-05	0.000188
TPST2	113244992	-3.39557546	-1.76366	6.21E-06	4.11E-05
PML	113240956	-3.39736885	-1.76442	2.01E-06	1.45E-05
NUDT5	113241068	-3.39816704	-1.76476	0.000383	0.00166
SMC6	113270140	-3.40318778	-1.76689	1.08E-05	6.75E-05
WNK4	113265400	-3.4034438	-1.767	0.000405	0.001742

PHKG2	113267697	-3.4042041	-1.76732	5.76E-06	3.83E-05
LOC113256263	113256263	-3.40577959	-1.76799	2.22E-05	0.000129
ZNF518B	113266679	-3.40706631	-1.76853	7.34E-05	0.00038
SKP2	113260895	-3.40722338	-1.7686	7.42E-05	0.000383
TMEM8A	113245368	-3.40875216	-1.76924	6.98E-06	4.57E-05
SLC19A1	113242695	-3.41000477	-1.76977	9.45E-06	5.99E-05
TIMP1	113265992	-3.41044453	-1.76996	0.000186	0.000873
NUDT1	113247716	-3.41419177	-1.77154	0.00041	0.001758
OFD1	113240718	-3.41675314	-1.77263	0.000252	0.001142
PDP2	113252337	-3.4176578	-1.77301	0.000303	0.001351
TP73	113270591	-3.41939091	-1.77374	0.003813	0.012302
SYCE1	113252112	-3.42519073	-1.77618	0.003304	0.010878
SGPL1	113259146	-3.42608362	-1.77656	3.07E-06	2.14E-05
SLC25A6	113240791	-3.42908351	-1.77782	0.006636	0.019828
ENPP2	113252777	-3.42976069	-1.77811	0.000325	0.001436
CASP3	113262259	-3.4299199	-1.77817	1.78E-06	1.3E-05
GK	113265832	-3.43225888	-1.77916	0.000186	0.000875
LOC113249507	113249507	-3.43269543	-1.77934	0.000389	0.001681
FBXO4	113260940	-3.43829762	-1.78169	0.000624	0.002548
LOC113257773	113257773	-3.43981201	-1.78233	1.58E-05	9.52E-05
FLNA	113247826	-3.44011402	-1.78246	0.014033	0.037291
SLC35E3	113256297	-3.44017577	-1.78248	1.5E-05	9.03E-05
PFAS	113271180	-3.44256336	-1.78348	2.89E-05	0.000164
ABCC10	113261265	-3.44257711	-1.78349	0.000127	0.000619
EFCAB14	113254712	-3.44262134	-1.78351	0.000301	0.001341
CPD	113269090	-3.44483259	-1.78443	0.001058	0.004042
ANKRD50	113255646	-3.44597295	-1.78491	0.000295	0.001318
ARPC4	113257149	-3.4459897	-1.78492	2E-06	1.44E-05

PPP1R15B	113242504	-3.44690772	-1.7853	6.32E-05	0.000332
ADAMTS6	113255284	-3.4482335	-1.78586	0.000707	0.00285
LOC113263111	113263111	-3.4497327	-1.78648	0.000525	0.002191
RFWD3	113252487	-3.45167603	-1.7873	1.58E-05	9.52E-05
MPND	113242830	-3.45236221	-1.78758	1.91E-06	1.39E-05
PRKACB	113254486	-3.4563818	-1.78926	2.57E-06	1.82E-05
RIN3	113267346	-3.45874153	-1.79025	2.51E-05	0.000144
NIPSNAP3A	113260370	-3.45948933	-1.79056	3.86E-05	0.000212
WDR78	113254555	-3.46096198	-1.79117	0.000825	0.003256
FAM84B	113252730	-3.46282229	-1.79195	0.000354	0.001544
SYT12	113244043	-3.4632921	-1.79214	0.000875	0.003428
SLC1A4	113244860	-3.46411708	-1.79249	0.000563	0.00233
TMEM120A	113267809	-3.46439408	-1.7926	5.72E-06	3.81E-05
B2M	113241942	-3.46528172	-1.79297	0.012397	0.033527
S1PR5	113242990	-3.46548378	-1.79306	0.001979	0.006966
TBC1D24	113245095	-3.46822141	-1.7942	0.000321	0.001417
RRM1	113269248	-3.46878227	-1.79443	3.83E-05	0.00021
MAN2B1	113247191	-3.4754864	-1.79721	0.000954	0.003699
POLR3G	113255112	-3.47635265	-1.79757	0.002461	0.008425
MCPH1	113262189	-3.47793038	-1.79823	2.62E-05	0.00015
PIEZO1	113252145	-3.47824998	-1.79836	0.000527	0.002196
LOC113243093	113243093	-3.4834828	-1.80053	0.004859	0.015196
PDIA5	113252317	-3.48399014	-1.80074	1.17E-05	7.26E-05
FBH1	113241138	-3.48554656	-1.80138	6.49E-05	0.000339
ZNF800	113263694	-3.48612226	-1.80162	8.37E-05	0.000427
HNRNPF	113243139	-3.48720374	-1.80207	0.000862	0.003387
LOC113260431	113260431	-3.48808613	-1.80244	0.004158	0.013252
PGM2	113262400	-3.48925744	-1.80292	3.1E-06	2.16E-05

FLRT3	113258157	-3.49033812	-1.80337	0.006277	0.018944
LOC113258957	113258957	-3.49199357	-1.80405	4.69E-05	0.000252
KCNK6	113243621	-3.49300577	-1.80447	0.000331	0.001457
SLC16A4	113244906	-3.49640022	-1.80587	0.006248	0.01887
SMIM3	113263960	-3.4973536	-1.80626	0.001097	0.004173
FAS	113258968	-3.50199345	-1.80818	0.000516	0.00216
LTC4S	113261556	-3.5030569	-1.80861	0.000938	0.003642
RNF24	113258391	-3.506277	-1.80994	0.001229	0.004612
BTLA	113251284	-3.50799962	-1.81065	0.002045	0.00717
TMEM50B	113264480	-3.50960593	-1.81131	4.77E-06	3.23E-05
TTYH3	113247736	-3.51067719	-1.81175	7.08E-06	4.62E-05
DNAH14	113262629	-3.51108437	-1.81192	0.003929	0.012603
INKA1	113256888	-3.51863039	-1.81501	0.000122	0.000599
ATG16L2	113269279	-3.51874363	-1.81506	2.09E-05	0.000122
CD38	113266704	-3.52098759	-1.81598	0.000143	0.000688
NUPR1	113267638	-3.52638886	-1.81819	0.012412	0.033549
ANKRD35	113247781	-3.52723736	-1.81854	0.000315	0.001394
SAMD15	113270287	-3.52842461	-1.81902	0.002563	0.008731
MCM7	113267879	-3.52892156	-1.81923	0.000197	0.000917
LOC113247684	113247684	-3.52959013	-1.8195	0.000684	0.002766
NUP205	113264561	-3.52992797	-1.81964	2.46E-06	1.75E-05
EPB41L3	113253238	-3.53058937	-1.81991	0.000104	0.000519
LOC113261708	113261708	-3.5323424	-1.82063	0.000134	0.00065
TIAM1	113266710	-3.53282914	-1.82082	0.000139	0.000673
DERA	113257842	-3.53362639	-1.82115	3.69E-05	0.000204
SEC23B	113258310	-3.53585753	-1.82206	3.62E-06	2.5E-05
LOC113241453	113241453	-3.53869338	-1.82322	0.004766	0.014941
SLC35C1	113265000	-3.5414615	-1.82434	1.8E-05	0.000107

FGD4	113257743	-3.54267873	-1.82484	4.52E-07	3.67E-06
AS3MT	113251882	-3.54295422	-1.82495	0.000196	0.000914
CHFR	113266833	-3.54333784	-1.82511	1.15E-05	7.16E-05
FAM241A	113255577	-3.54373148	-1.82527	1.74E-06	1.27E-05
CUNH3orf62	113256870	-3.54396973	-1.82537	0.000199	0.000926
LOC113264260	113264260	-3.54457978	-1.82561	0.011646	0.031795
IMPDH1	113263791	-3.54881607	-1.82734	1.89E-06	1.37E-05
EFHC2	113265902	-3.54882785	-1.82734	0.004505	0.014197
ERGIC1	113264097	-3.55296185	-1.82902	6.92E-06	4.53E-05
SUZ12	113268999	-3.55329768	-1.82916	8.56E-06	5.49E-05
XPR1	113262704	-3.55919692	-1.83155	7.97E-06	5.15E-05
LOC113246021	113246021	-3.56064052	-1.83214	0.001282	0.004789
LOC113259257	113259257	-3.56117993	-1.83236	0.017385	0.04468
MAOB	113265900	-3.56134079	-1.83242	0.002198	0.007649
PTGDR	113242377	-3.56258951	-1.83293	0.00547	0.01684
SLC9A1	113268442	-3.56356951	-1.83332	4.76E-07	3.85E-06
TIPIN	113240884	-3.56391566	-1.83346	7.38E-05	0.000382
EBI3	113242825	-3.56637846	-1.83446	0.008236	0.023723
NEK6	113266394	-3.56705581	-1.83473	1.93E-05	0.000114
LIPC	113269678	-3.56812288	-1.83517	0.010361	0.028783
SAMHD1	113258570	-3.5766983	-1.83863	6.76E-06	4.43E-05
DZIP1L	113253977	-3.57747564	-1.83894	0.002816	0.009484
TMEM173	113261804	-3.57765955	-1.83902	0.000254	0.00115
PLCXD1	113240798	-3.57933931	-1.83969	0.001615	0.005849
ZYX	113265542	-3.58140584	-1.84053	0.000309	0.001375
STUM	113262927	-3.58360159	-1.84141	0.000775	0.003087
VSIR	113259153	-3.5858778	-1.84233	0.000441	0.001872
PRR5L	113265030	-3.58969269	-1.84386	0.001541	0.005625

FEN1	113246985	-3.59014742	-1.84404	1.82E-05	0.000108
KBTBD7	113251380	-3.59035216	-1.84413	0.000381	0.001652
HSP90B1	113256067	-3.5911366	-1.84444	0.001105	0.004197
NT5C3B	113265444	-3.59119986	-1.84447	4.99E-06	3.36E-05
WIPF1	113250755	-3.59254902	-1.84501	6.3E-05	0.000331
PIBF1	113251417	-3.59712953	-1.84685	0.000849	0.003341
TNFRSF21	113261226	-3.59773846	-1.84709	7.07E-06	4.62E-05
LOC113248453	113248453	-3.59968882	-1.84787	9.09E-07	6.99E-06
SLC12A6	113242080	-3.60449289	-1.8498	0.000415	0.001775
UBE2O	113244596	-3.60710847	-1.85084	0.000755	0.003017
SLAMF9	113246664	-3.60881619	-1.85153	0.000964	0.003727
PDCL	113266380	-3.61343722	-1.85337	7.38E-06	4.81E-05
MYC	113252726	-3.61657344	-1.85462	1.21E-05	7.51E-05
AZIN2	113268335	-3.61799292	-1.85519	1.21E-05	7.51E-05
EZR	113259607	-3.62041201	-1.85615	0.000242	0.001101
GMCL1	113244505	-3.62105151	-1.85641	2.98E-05	0.000168
PTBP3	113260634	-3.62352278	-1.85739	3.14E-05	0.000175
ACTR2	113244810	-3.62840499	-1.85934	0.000981	0.003782
NOL4L	113258480	-3.63134185	-1.8605	2.23E-05	0.000129
MARC1	113268719	-3.63298124	-1.86115	0.009254	0.026183
TOP1	113258607	-3.63334571	-1.8613	7.39E-05	0.000382
GRAMD1A	113244767	-3.63344038	-1.86134	0.000192	0.000896
ATP6V0B	113254775	-3.63993463	-1.86391	8.34E-07	6.45E-06
SVIP	113265104	-3.64103001	-1.86435	6.62E-05	0.000346
LHFPL2	113255174	-3.64324537	-1.86522	6.04E-06	4.01E-05
CTPS1	113254822	-3.64710436	-1.86675	2.1E-05	0.000122
AP1S1	113267838	-3.64828067	-1.86722	7.87E-06	5.1E-05
RAD9A	113244032	-3.64903665	-1.86752	1.27E-06	9.46E-06

REC8	113245986	-3.64929589	-1.86762	0.000456	0.001929
TMSB4X	113240727	-3.6512328	-1.86838	0.009029	0.025627
ESYT3	113253985	-3.65386274	-1.86942	0.00056	0.00232
E2F4	113252353	-3.66132014	-1.87236	8.14E-06	5.24E-05
MBOAT7	113247398	-3.66648793	-1.8744	2.86E-06	2.01E-05
SFXN1	113264117	-3.67144518	-1.87635	1.92E-06	1.39E-05
NUCB2	113246624	-3.67311195	-1.877	5.65E-06	3.76E-05
SORL1	113259938	-3.67422333	-1.87744	1.32E-06	9.78E-06
LRRC27	113252077	-3.67703452	-1.87854	0.000212	0.000982
PLOD2	113254030	-3.67705448	-1.87855	1.63E-05	9.78E-05
ZNF711	113242165	-3.68091257	-1.88006	0.009025	0.025626
PPM1M	113256945	-3.68182073	-1.88042	4.63E-07	3.75E-06
ABCB4	113259445	-3.68655311	-1.88227	0.001543	0.005632
ALG14	113254379	-3.69200539	-1.8844	0.000178	0.000843
LOC113265643	113265643	-3.69334709	-1.88493	4.21E-06	2.88E-05
SMCHD1	113253255	-3.69352934	-1.885	5.2E-05	0.000278
RHPN1	113247654	-3.6953748	-1.88572	0.00981	0.027483
CALR	113247206	-3.69988861	-1.88748	0.000901	0.003514
CDK2AP2	113244021	-3.70112669	-1.88796	3.54E-07	2.93E-06
LRP11	113259551	-3.70594824	-1.88984	0.000101	0.000508
TLR6	113262410	-3.71187547	-1.89215	1.49E-05	8.97E-05
EML2	113243421	-3.71307129	-1.89261	8.04E-06	5.18E-05
CPNE2	113252272	-3.7180709	-1.89455	2.23E-06	1.59E-05
ARMC9	113250357	-3.72076191	-1.8956	1.7E-05	0.000101
KIAA0513	113252185	-3.72097271	-1.89568	0.000224	0.001031
FUCA1	113268513	-3.72735774	-1.89815	1.52E-06	1.12E-05
PPIA	113257787	-3.72841604	-1.89856	0.00043	0.001833
GSR	113262134	-3.72933399	-1.89892	1.39E-06	1.03E-05

LOC113246311	113246311	-3.73031429	-1.8993	0.002584	0.008787
MYO9B	113256664	-3.73117769	-1.89963	8.89E-05	0.000451
ADAMTS14	113258879	-3.73371668	-1.90061	0.000522	0.002181
RHBDF2	113244595	-3.73404197	-1.90074	9.14E-06	5.82E-05
ZC3HAV1	113264867	-3.73409439	-1.90076	9.31E-07	7.15E-06
SLC25A10	113244506	-3.73470956	-1.901	0.001708	0.006136
ANPEP	113263695	-3.73594503	-1.90147	0.014813	0.039058
MYH10	113271188	-3.73649202	-1.90168	0.000451	0.001911
SLC5A2	113267726	-3.73862525	-1.90251	0.000275	0.001238
CARD6	113260930	-3.74358565	-1.90442	8.32E-07	6.44E-06
ENTPD5	113270347	-3.74399733	-1.90458	5.13E-05	0.000274
EMP3	113243353	-3.74535305	-1.9051	1.48E-05	8.94E-05
RABIF	113242533	-3.74548407	-1.90515	1.3E-06	9.69E-06
HAT1	113250775	-3.74837132	-1.90626	1.23E-06	9.23E-06
TMEM176A	113241472	-3.74855756	-1.90634	0.000252	0.001142
ELF1	113251381	-3.74982293	-1.90682	4.95E-06	3.34E-05
COL5A3	113242683	-3.75226109	-1.90776	0.015289	0.040119
PAPSS1	113255537	-3.762843	-1.91182	3.23E-06	2.25E-05
SLC45A4	113247951	-3.76394732	-1.91225	6.13E-05	0.000323
RAVER1	113242981	-3.76421361	-1.91235	2.67E-06	1.88E-05
DCLRE1A	113251953	-3.76456729	-1.91248	1.84E-06	1.34E-05
PSMB9	113243783	-3.7667202	-1.91331	4E-05	0.000218
HVCN1	113266965	-3.76725699	-1.91351	1.59E-05	9.55E-05
HAUS1	113253375	-3.76736328	-1.91356	1.73E-05	0.000103
SERP1	113254059	-3.77252136	-1.91553	2.13E-05	0.000124
CASP7	113251949	-3.77305515	-1.91573	2.89E-06	2.03E-05
CTSZ	113258770	-3.77688742	-1.9172	6.23E-07	4.93E-06
MYEF2	113269586	-3.777774	-1.91754	0.001258	0.004713

RANBP3L	113260896	-3.78213001	-1.9192	0.002608	0.008854
CDC14A	113254350	-3.78455292	-1.92012	2.63E-05	0.00015
ADAM19	113264021	-3.78703919	-1.92107	8.56E-06	5.49E-05
GPAT3	113263192	-3.78937761	-1.92196	2.98E-06	2.08E-05
MFSD14B	113270083	-3.79003309	-1.92221	1.55E-06	1.14E-05
ACKR1	113246679	-3.79704377	-1.92488	7.4E-06	4.82E-05
HPS1	113251807	-3.80273474	-1.92704	1.36E-06	1.01E-05
PRDM1	113264785	-3.80432093	-1.92764	0.000228	0.001045
TLR7	113240728	-3.8046811	-1.92778	0.000589	0.002426
PTPN18	113251152	-3.81043372	-1.92996	0.000118	0.000583
AK3	113270164	-3.81108877	-1.9302	4.69E-07	3.79E-06
AMPD2	113244918	-3.81132812	-1.93029	4.45E-06	3.03E-05
ARID3B	113240966	-3.81166396	-1.93042	1.44E-05	8.76E-05
TMEM205	113243020	-3.81377761	-1.93122	0.000635	0.00259
JAZF1	113262144	-3.81449597	-1.93149	1.87E-05	0.00011
TTC39C	113253296	-3.81522388	-1.93177	0.000277	0.001243
ADGRE5	113247147	-3.81761595	-1.93267	9.02E-07	6.94E-06
ERP29	113266976	-3.81780597	-1.93274	3.7E-07	3.04E-06
MSRB1	113245140	-3.824362	-1.93522	5.19E-05	0.000277
CUNH5orf30	113255025	-3.82539337	-1.93561	5.94E-06	3.94E-05
CTSH	113263792	-3.82826703	-1.93669	1.55E-06	1.14E-05
GPR34	113265887	-3.83015347	-1.9374	2.86E-05	0.000162
LOC113243786	113243786	-3.83074095	-1.93762	9.66E-07	7.4E-06
LOC113254133	113254133	-3.83266545	-1.93835	3.96E-05	0.000216
POLA2	113244119	-3.8344426	-1.93902	3.04E-06	2.12E-05
SFI1	113245062	-3.83482987	-1.93916	3.39E-05	0.000188
STARD5	113263765	-3.83909004	-1.94076	8.53E-06	5.48E-05
UBN1	113270831	-3.84278121	-1.94215	1.09E-05	6.85E-05

TNFAIP3	113259486	-3.84348484	-1.94241	1.98E-05	0.000116
LRRC3	113251316	-3.8470491	-1.94375	0.002795	0.009426
SPATS2	113257650	-3.84816234	-1.94417	1.13E-06	8.51E-06
IFI6	113268433	-3.84836858	-1.94425	0.000499	0.002097
SLC25A16	113259113	-3.84964694	-1.94473	0.001338	0.00497
ZSWIM6	113254899	-3.84987401	-1.94481	6.92E-05	0.00036
MARCKSL1	113268358	-3.85076296	-1.94514	0.000135	0.000656
GPSM2	113244939	-3.85564844	-1.94697	8.83E-07	6.8E-06
GZMK	113263921	-3.85836104	-1.94799	0.001419	0.005232
NCOA7	113259416	-3.87049706	-1.95252	3.19E-07	2.66E-06
LRRC3C	113265504	-3.87226836	-1.95318	0.011796	0.032152
ELMO1	113262812	-3.87303129	-1.95346	1.03E-06	7.85E-06
TUBA1C	113257654	-3.87440545	-1.95397	0.00027	0.001215
PRCP	113269378	-3.87454448	-1.95403	7.55E-07	5.88E-06
RINL	113243605	-3.87572216	-1.95447	0.00051	0.002136
HNRNPAB	113261580	-3.8786664	-1.95556	0.000147	0.000708
NINL	113248359	-3.87884361	-1.95563	0.000138	0.000665
NADK	113270619	-3.88119224	-1.9565	9.25E-05	0.000467
PRELID1	113264141	-3.88136294	-1.95656	5.2E-06	3.49E-05
C1RL	113257942	-3.88308343	-1.9572	0.000599	0.00246
CEP128	113270273	-3.88322414	-1.95725	0.000137	0.000665
TOR2A	113266433	-3.88370225	-1.95743	2.64E-05	0.000151
CHPT1	113256089	-3.88552667	-1.95811	0.001859	0.006598
CHST15	113252027	-3.88853357	-1.95923	1.41E-05	8.62E-05
PHGDH	113244360	-3.89108441	-1.96017	5.81E-06	3.86E-05
TNS3	113257644	-3.89221806	-1.96059	0.000358	0.001563
LOC113249452	113249452	-3.90085049	-1.96379	0.000342	0.001497
CHST11	113256057	-3.90165522	-1.96409	2.92E-05	0.000165

KCP	113263934	-3.90796087	-1.96642	0.00045	0.00191
IRAK4	113257718	-3.91163777	-1.96777	5.52E-07	4.41E-06
LOC113256915	113256915	-3.91538157	-1.96915	0.000311	0.00138
C1S	113257945	-3.91540971	-1.96916	2.22E-05	0.000128
AK2	113268337	-3.91621653	-1.96946	1.24E-05	7.65E-05
LOC113269263	113269263	-3.91638234	-1.96952	4.46E-06	3.04E-05
DOK1	113243457	-3.91742153	-1.9699	1.84E-05	0.000109
HMGA1	113243823	-3.92323595	-1.97204	0.00031	0.001375
CBL	113259962	-3.92470037	-1.97258	3.08E-06	2.15E-05
RAP1B	113256300	-3.92698742	-1.97342	7.67E-05	0.000394
ACO1	113260538	-3.92749463	-1.97361	0.001665	0.006007
EXOSC4	113247677	-3.93174788	-1.97517	1.34E-05	8.2E-05
FCER1A	113246733	-3.93176711	-1.97518	0.001198	0.004508
GPX1	113256871	-3.93466517	-1.97624	0.000367	0.001595
FADS3	113246997	-3.93478439	-1.97628	0.002164	0.007546
NDE1	113270761	-3.93489694	-1.97633	1.15E-06	8.62E-06
RILPL2	113266896	-3.93589079	-1.97669	4.91E-06	3.32E-05
MON1B	113252512	-3.938575	-1.97767	4.51E-06	3.07E-05
TMSB15A	113244311	-3.93886375	-1.97778	1.47E-05	8.9E-05
ATL1	113266214	-3.93891142	-1.9778	0.001741	0.00624
KLHL25	113263670	-3.9402318	-1.97828	9.95E-05	0.000499
ARHGAP27	113265320	-3.94023603	-1.97828	1.45E-05	8.78E-05
CD47	113250904	-3.94157691	-1.97877	1.79E-06	1.3E-05
SLC7A7	113246073	-3.94488506	-1.97998	1.9E-06	1.38E-05
PTGER3	113254534	-3.94695849	-1.98074	0.0017	0.006114
MAN1C1	113268483	-3.94852631	-1.98131	1.45E-05	8.81E-05
OSTF1	113269842	-3.95156321	-1.98242	3.14E-06	2.19E-05
LOC113248320	113248320	-3.95277873	-1.98287	0.00161	0.005836

ADPGK	113240943	-3.96025935	-1.98559	1.89E-07	1.65E-06
DTX3L	113252271	-3.96300356	-1.98659	4.1E-07	3.36E-06
GAS7	113271206	-3.96402466	-1.98697	2.05E-07	1.77E-06
C3AR1	113257930	-3.96930396	-1.98889	0.000331	0.001457
PCLO	113259286	-3.97413537	-1.99064	0.000566	0.002337
DEDD2	113243512	-3.98007607	-1.9928	5.38E-05	0.000286
CELF6	113240938	-3.98120914	-1.99321	0.000498	0.002092
TRERF1	113261295	-3.98154456	-1.99333	3.86E-05	0.000212
TUT7	113269792	-3.9889162	-1.996	1.33E-05	8.15E-05
CMTM6	113253836	-3.99032923	-1.99651	7.16E-07	5.61E-06
LOC113248329	113248329	-3.99330039	-1.99758	1.93E-07	1.68E-06
MAST3	113256703	-3.99774098	-1.99918	6.44E-06	4.25E-05
ZGRF1	113255586	-4.00029531	-2.00011	5.48E-05	0.000291
PLD1	113255060	-4.00474839	-2.00171	3.34E-05	0.000186
LYPD6	113250899	-4.00660644	-2.00238	0.004944	0.015427
LIPE	113243521	-4.00662341	-2.00239	0.003844	0.012384
MOXD1	113259456	-4.01565691	-2.00564	0.000661	0.002682
MX2	113256765	-4.01614105	-2.00581	0.000533	0.002217
CCDC18	113254399	-4.02118347	-2.00762	7.5E-06	4.88E-05
EGFL6	113240724	-4.02377972	-2.00855	0.003487	0.0114
LASP1	113265534	-4.02383382	-2.00857	5.4E-06	3.62E-05
HAAO	113246496	-4.02395055	-2.00861	1.23E-05	7.59E-05
GNAZ	113247070	-4.02689089	-2.00967	0.000645	0.002628
IL10RA	113260009	-4.03094526	-2.01112	1.26E-05	7.8E-05
CKB	113267218	-4.03193489	-2.01147	0.000147	0.000706
ARMC4	113244257	-4.03402684	-2.01222	0.002802	0.00944
C4BPB	113242606	-4.03476863	-2.01249	1.12E-06	8.43E-06
CSF1R	113263940	-4.03912972	-2.01404	6.33E-06	4.18E-05

MINPP1	113258980	-4.0405557	-2.01455	2.29E-07	1.97E-06
CHRNA5	113263807	-4.04150076	-2.01489	0.007451	0.021843
KLRG1	113257921	-4.0461773	-2.01656	2.01E-05	0.000117
HELB	113256310	-4.04795505	-2.01719	3.01E-05	0.000169
HEBP1	113257865	-4.05408814	-2.01938	1.77E-06	1.29E-05
DCAF12	113260516	-4.05637442	-2.02019	6.36E-05	0.000334
SMC1A	113247516	-4.07434953	-2.02657	0.000135	0.000653
LOC113263976	113263976	-4.0757432	-2.02706	0.006618	0.01979
SRC	113258578	-4.07613395	-2.0272	6.49E-06	4.28E-05
EAF2	113252133	-4.076701	-2.0274	0.002201	0.007654
GLUL	113262718	-4.07693504	-2.02748	0.001237	0.004641
LMNB2	113242766	-4.07716269	-2.02757	1.19E-06	8.93E-06
ARHGAP33	113244760	-4.07846351	-2.02803	5.97E-05	0.000315
SDK2	113265212	-4.08032417	-2.02868	0.00817	0.023594
CASP8	113250592	-4.08414189	-2.03003	1.2E-07	1.09E-06
CUNH19orf71	113242803	-4.08614203	-2.03074	0.000711	0.002863
BTN1A1	113264254	-4.0951686	-2.03392	1.22E-06	9.15E-06
ITGB7	113257564	-4.0992241	-2.03535	1.89E-05	0.000112
AZIN1	113252854	-4.10230062	-2.03643	9.85E-05	0.000495
RFC3	113251252	-4.10545195	-2.03754	9.34E-06	5.94E-05
IFI35	113265387	-4.10556882	-2.03758	1.01E-06	7.68E-06
VWA2	113251959	-4.11186746	-2.03979	0.00485	0.015177
MYD88	113253781	-4.12837074	-2.04557	1.23E-08	1.33E-07
WRN	113262139	-4.1374276	-2.04873	1.26E-05	7.76E-05
MFSD10	113245734	-4.13956336	-2.04948	1.08E-06	8.21E-06
GIPR	113243420	-4.14659822	-2.05193	0.008202	0.023651
LETM2	113262088	-4.1480443	-2.05243	6.52E-05	0.00034
CUNH11orf80	113244051	-4.14867373	-2.05265	0.014138	0.037506

DNAH11	113261258	-4.15393261	-2.05448	0.00022	0.001017
LOC113264282	113264282	-4.17015631	-2.0601	0.00118	0.004451
SIRT7	113244493	-4.17171325	-2.06064	4.26E-07	3.48E-06
SERPINF1	113269130	-4.17404266	-2.06145	0.001583	0.005757
PPIP5K2	113255027	-4.17405019	-2.06145	9.72E-07	7.44E-06
NCAPH	113241929	-4.17741914	-2.06261	2.43E-07	2.08E-06
SLC25A38	113253799	-4.17749035	-2.06264	8.62E-07	6.65E-06
MAD2L1	113255618	-4.17971152	-2.0634	7.06E-07	5.53E-06
CGAS	113261117	-4.18320015	-2.06461	0.000585	0.002413
BCL2A1	113263784	-4.18898392	-2.0666	0.000173	0.000822
ARMH2	113264325	-4.19209436	-2.06767	0.003355	0.01102
PARP12	113265009	-4.19321994	-2.06806	1.09E-06	8.24E-06
RETN	113242926	-4.19482764	-2.06861	0.000237	0.001082
SSBP3	113254633	-4.19520766	-2.06874	7.88E-07	6.13E-06
CGN	113246194	-4.20359224	-2.07162	0.001816	0.00647
TTC39B	113260608	-4.20547972	-2.07227	7.04E-05	0.000366
CDHR4	113256614	-4.20575249	-2.07236	0.002551	0.008693
BCAT1	113257794	-4.20981721	-2.07376	9.4E-06	5.97E-05
GRN	113265352	-4.21194166	-2.07449	2.56E-05	0.000146
PDE5A	113255615	-4.2128862	-2.07481	4.12E-06	2.82E-05
SLBP	113245752	-4.21297979	-2.07484	7.26E-07	5.68E-06
TSPAN14	113259228	-4.21980673	-2.07718	1.11E-07	1.01E-06
PMM2	113270815	-4.22271384	-2.07817	2.42E-07	2.07E-06
LOC113269256	113269256	-4.22833263	-2.08009	0.001929	0.006809
ETV6	113257879	-4.23510221	-2.0824	2.2E-06	1.57E-05
MCUB	113255555	-4.2355163	-2.08254	8.46E-08	7.91E-07
PPP1R18	113264357	-4.23717057	-2.0831	1.06E-06	8.04E-06
SPHK1	113244597	-4.23895642	-2.08371	8.2E-08	7.7E-07

RHBDL2	113268258	-4.23951034	-2.0839	0.009338	0.026366
C7	113260932	-4.24037841	-2.08419	9.35E-06	5.94E-05
CHRNA3	113263806	-4.24339374	-2.08522	0.005253	0.016262
SLC49A3	113245736	-4.24358166	-2.08528	3.66E-05	0.000202
HAUS5	113244774	-4.24885601	-2.08707	2.76E-06	1.94E-05
GRINA	113247592	-4.25067875	-2.08769	5.73E-06	3.81E-05
ATP1B3	113254012	-4.25818867	-2.09024	4.88E-07	3.94E-06
PDK3	113242221	-4.25897724	-2.09051	3.99E-06	2.74E-05
TFDP2	113254014	-4.26870737	-2.0938	0.000115	0.000568
SFN	113268452	-4.26896506	-2.09389	0.000584	0.00241
AGER	113243764	-4.27739284	-2.09673	0.000416	0.00178
UROS	113252048	-4.27889224	-2.09724	1.27E-05	7.85E-05
DENND4A	113240877	-4.2825589	-2.09847	3.03E-05	0.00017
TLL2	113251770	-4.28541393	-2.09943	0.009593	0.026974
UROD	113254751	-4.28571942	-2.09954	1.88E-06	1.36E-05
LOC113248439	113248439	-4.28829132	-2.1004	0.005063	0.015764
EXOC3L2	113243437	-4.30331048	-2.10545	0.003785	0.01223
FNBP1	113266518	-4.31720132	-2.1101	1.93E-07	1.68E-06
TRIM37	113268881	-4.32012203	-2.11107	7.95E-08	7.49E-07
ENTPD7	113251816	-4.32018383	-2.11109	1.01E-05	6.36E-05
SLC16A6	113265239	-4.32226225	-2.11179	0.000657	0.002668
MFNG	113241808	-4.32581847	-2.11297	1.45E-07	1.29E-06
RAB8A	113256641	-4.32645654	-2.11319	2.73E-07	2.31E-06
WDR31	113266314	-4.32856879	-2.11389	0.000651	0.002648
APOC1	113243451	-4.33006071	-2.11439	0.000756	0.003017
ECHDC1	113259426	-4.33324557	-2.11545	3.73E-05	0.000205
ENDOD1	113259814	-4.33396627	-2.11569	8.7E-05	0.000443
MTBP	113252769	-4.33731608	-2.1168	1.44E-05	8.77E-05

TENT5A	113261073	-4.34882364	-2.12063	7.37E-05	0.000381
TLR4	113266343	-4.35228306	-2.12177	6.3E-07	4.98E-06
HELLS	113243188	-4.35289969	-2.12198	5.55E-06	3.7E-05
LDLR	113243010	-4.35723782	-2.12341	5.38E-06	3.6E-05
RAB3D	113243016	-4.35765507	-2.12355	4.83E-06	3.27E-05
HYOU1	113259976	-4.35962919	-2.12421	1.1E-07	1.01E-06
TAP1	113243781	-4.3599963	-2.12433	8.07E-08	7.6E-07
GRAMD1B	113259927	-4.36339796	-2.12545	2.82E-05	0.00016
CCK	113253819	-4.36694433	-2.12662	0.0016	0.005808
LOC113243706	113243706	-4.37082951	-2.12791	8.56E-07	6.6E-06
SMPDL3A	113259406	-4.37555169	-2.12946	1.9E-05	0.000112
RAB38	113269414	-4.37770526	-2.13017	0.000785	0.003116
RNF17	113251157	-4.38331475	-2.13202	0.000721	0.002898
CPZ	113245674	-4.3834324	-2.13206	0.002156	0.007521
GLYCTK	113256948	-4.38459294	-2.13244	1.71E-06	1.25E-05
MRNIP	113261552	-4.38870731	-2.1338	8.96E-06	5.72E-05
TTC25	113265437	-4.38990788	-2.13419	0.000178	0.000843
INPP4A	113246757	-4.3904051	-2.13435	7E-07	5.49E-06
LOC113243878	113243878	-4.39902859	-2.13718	0.000375	0.001625
NHSL2	113245601	-4.39980607	-2.13744	2.19E-05	0.000127
MMD	113265653	-4.40034668	-2.13762	2.01E-07	1.74E-06
HECTD4	113266981	-4.40512662	-2.13918	8.95E-05	0.000453
SLC25A37	113269979	-4.40730243	-2.1399	0.000615	0.002518
LOC113245037	113245037	-4.4075716	-2.13998	0.013018	0.034998
DTX4	113246549	-4.40836415	-2.14024	1.24E-07	1.12E-06
TRPV2	113271253	-4.40963685	-2.14066	3.09E-07	2.59E-06
SRPX	113265859	-4.4111003	-2.14114	0.000225	0.001034
PGAM1	113251781	-4.41579381	-2.14267	1.9E-05	0.000112

RTN3	113244199	-4.4179656	-2.14338	3.49E-05	0.000193
CGREF1	113270980	-4.41850406	-2.14356	0.000205	0.000949
PPM1H	113256344	-4.41950363	-2.14388	2.91E-08	2.94E-07
SMCO3	113257853	-4.41957923	-2.14391	0.000313	0.001389
CCHCR1	113264341	-4.42233887	-2.14481	3.16E-07	2.64E-06
L3MBTL1	113258620	-4.42693956	-2.14631	0.001136	0.004306
FANCC	113269780	-4.42847696	-2.14681	3.77E-05	0.000207
STXBP5	113259533	-4.43479385	-2.14887	3.67E-07	3.02E-06
TSC22D4	113267863	-4.43876125	-2.15016	6.97E-07	5.47E-06
CRLF1	113256600	-4.44001939	-2.15057	0.012025	0.032683
PHLPP2	113252456	-4.44101968	-2.15089	1.76E-05	0.000105
LOC113270472	113270472	-4.44535921	-2.1523	1.29E-05	7.95E-05
ACVR1C	113250865	-4.44615015	-2.15256	0.00136	0.005039
PRSS35	113261060	-4.44891466	-2.15345	0.008155	0.023561
ATP6V1A	113251490	-4.45112216	-2.15417	6.2E-07	4.91E-06
CDH2	113253311	-4.46174543	-2.15761	0.008011	0.023243
SNX15	113244141	-4.48073376	-2.16374	2.62E-06	1.86E-05
NOD2	113252225	-4.48077301	-2.16375	4.01E-05	0.000218
CD55	113242611	-4.48385476	-2.16474	5.01E-06	3.38E-05
CUNH7orf61	113267864	-4.48581857	-2.16537	0.000135	0.000656
LOC113270979	113270979	-4.49096081	-2.16702	3.23E-07	2.69E-06
DBNL	113257931	-4.49173512	-2.16727	9.31E-08	8.62E-07
CFAP43	113251901	-4.49428604	-2.16809	0.000259	0.00117
PDIA4	113241454	-4.49488697	-2.16828	4.9E-07	3.95E-06
RANGAP1	113241735	-4.50223403	-2.17064	2.14E-08	2.21E-07
RARRES1	113254123	-4.51053485	-2.1733	0.000239	0.001089
ARL6IP6	113250878	-4.51602424	-2.17505	3.16E-07	2.64E-06
PIP4K2A	113241338	-4.52270132	-2.17718	5.69E-07	4.53E-06

ABHD3	113253273	-4.52386482	-2.17756	2.83E-06	1.99E-05
P2RY12	113254065	-4.52506147	-2.17794	0.000108	0.000536
PARP8	113260964	-4.5314162	-2.17996	5.57E-07	4.45E-06
ARL6IP1	113270748	-4.53435151	-2.1809	1.29E-05	7.95E-05
HAP1	113265447	-4.53589011	-2.18139	0.000395	0.001703
FAM214B	113260486	-4.53591112	-2.18139	1.11E-07	1.01E-06
IRF4	113264375	-4.53676651	-2.18166	4.17E-06	2.85E-05
SLCO4A1	113258796	-4.53855126	-2.18223	1.7E-05	0.000101
CTSW	113244090	-4.54118957	-2.18307	0.001499	0.005498
XPO6	113267617	-4.54504072	-2.18429	7.81E-08	7.38E-07
PLD4	113267186	-4.55825015	-2.18848	0.000344	0.001508
VCAN	113255135	-4.56843903	-2.1917	0.001036	0.003971
LOC113264292	113264292	-4.56882668	-2.19182	0.000161	0.000769
BST2	113256678	-4.57070634	-2.19242	6.25E-07	4.95E-06
LPCAT2	113252250	-4.57690791	-2.19437	8.2E-06	5.28E-05
HMGN2	113268458	-4.58198246	-2.19597	0.001754	0.006275
PTGS2	113262761	-4.58221175	-2.19604	0.000211	0.000978
SIPA1	113244101	-4.58596829	-2.19723	4.65E-06	3.16E-05
MARCH3	113261671	-4.58904922	-2.1982	0.000191	0.000894
ADAMTS2	113261565	-4.59597196	-2.20037	0.001259	0.004715
WDHD1	113242349	-4.60192817	-2.20224	1.63E-08	1.73E-07
SIDT1	113251459	-4.60648778	-2.20367	0.001837	0.006531
VASP	113243424	-4.60651049	-2.20367	7.3E-06	4.75E-05
LEF1	113255544	-4.60696506	-2.20382	0.001455	0.005347
GLIPR2	113260456	-4.61148719	-2.20523	1.12E-07	1.02E-06
DPY19L1	113262663	-4.61799206	-2.20727	1.22E-08	1.33E-07
ARAP1	113269274	-4.62544929	-2.20959	1.64E-07	1.45E-06
DHFR	113255158	-4.63144353	-2.21146	5.07E-09	5.86E-08

STRA8	113267039	-4.63580052	-2.21282	1.29E-07	1.16E-06
FZD3	113269945	-4.63812694	-2.21354	0.001382	0.005114
ERICH3	113254525	-4.64012353	-2.21416	0.002867	0.009619
LOC113260144	113260144	-4.64183105	-2.21469	8.45E-06	5.43E-05
PRXL2A	113259227	-4.64371874	-2.21528	0.002348	0.008081
LOC113241797	113241797	-4.64378828	-2.2153	0.000347	0.001516
EPSTI1	113251367	-4.64636933	-2.2161	0.00013	0.000635
GLDN	113269624	-4.6469378	-2.21628	0.001287	0.004805
NLRP1	113271055	-4.65137339	-2.21766	0.000101	0.000507
SHTN1	113251974	-4.65500088	-2.21878	1.43E-06	1.06E-05
MPL	113254790	-4.66206809	-2.22097	0.000185	0.00087
SLC2A3	113257927	-4.66311079	-2.22129	5.63E-05	0.000298
GALC	113267389	-4.67379585	-2.22459	3.87E-05	0.000212
NAAA	113263259	-4.67664794	-2.22547	8.66E-08	8.08E-07
RBL1	113258573	-4.67702775	-2.22559	6.83E-07	5.37E-06
BCAS1	113258740	-4.67969152	-2.22641	0.000187	0.000879
HPD	113266938	-4.68085714	-2.22677	0.00213	0.007433
PRKAR2B	113258614	-4.68415963	-2.22779	1.45E-06	1.07E-05
PFKP	113241114	-4.68491327	-2.22802	4.95E-08	4.83E-07
LOC113246847	113246847	-4.68715123	-2.22871	5.32E-05	0.000283
WDR86	113241489	-4.68909384	-2.22931	0.004195	0.013347
CSK	113240974	-4.71337102	-2.23676	2.08E-07	1.8E-06
LOC113264273	113264273	-4.71518254	-2.23731	0.001006	0.00387
SMPD2	113264749	-4.72230375	-2.23949	1.55E-05	9.33E-05
SKAP1	113265573	-4.72898131	-2.24153	0.000756	0.003017
CENPJ	113251158	-4.73764613	-2.24417	9.77E-05	0.000491
SCARB1	113266877	-4.74128416	-2.24528	2E-08	2.08E-07
SLC7A3	113245611	-4.74889211	-2.24759	0.00086	0.00338

RGS2	113268748	-4.75075918	-2.24816	8.32E-06	5.35E-05
FAM49B	113252720	-4.7573043	-2.25014	4.27E-08	4.23E-07
CDKL1	113266209	-4.76196451	-2.25156	4.51E-08	4.44E-07
S100A4	113245344	-4.76224971	-2.25164	0.000153	0.000732
TRPC6	113260153	-4.76348628	-2.25202	2.98E-05	0.000168
SCLT1	113255666	-4.76484793	-2.25243	4.74E-06	3.21E-05
PAN3	113251214	-4.77255855	-2.25476	4.2E-07	3.43E-06
G6PC3	113265366	-4.78412565	-2.25826	2.01E-09	2.53E-08
KBTBD11	113262011	-4.78437832	-2.25833	0.000311	0.00138
SLC30A1	113262666	-4.78940766	-2.25985	1.15E-08	1.26E-07
PRIMPOL	113262260	-4.79267611	-2.26083	3.58E-07	2.96E-06
KIAA1551	113257747	-4.79321677	-2.26099	6.36E-07	5.02E-06
CPNE3	113252972	-4.79654557	-2.262	8E-06	5.17E-05
BAIAP2L1	113267925	-4.79847855	-2.26258	0.000961	0.003718
LMO2	113265053	-4.80022532	-2.2631	1.49E-07	1.34E-06
ZNF217	113258297	-4.80542716	-2.26466	1.59E-07	1.41E-06
CPOX	113250381	-4.80613327	-2.26488	1.56E-05	9.41E-05
NFKBIE	113261039	-4.80634034	-2.26494	7.02E-07	5.51E-06
ME1	113261331	-4.81518911	-2.26759	0.010397	0.028859
RPGRIP1L	113252239	-4.82351724	-2.27009	9.49E-06	6.02E-05
RUNX2	113261240	-4.82702818	-2.27114	0.003338	0.010972
GLCCI1	113260511	-4.82772115	-2.27134	5.02E-07	4.04E-06
IRAK3	113256312	-4.83142602	-2.27245	4.49E-06	3.05E-05
SLC25A5	113241271	-4.84087608	-2.27527	6.47E-09	7.34E-08
ARPC1B	113267914	-4.84116305	-2.27535	5.56E-06	3.71E-05
FIGNL1	113257549	-4.84172218	-2.27552	6.72E-05	0.00035
KCNQ1	113243965	-4.8457246	-2.27671	9.72E-08	8.97E-07
PUS10	113245273	-4.85008273	-2.27801	3.05E-06	2.13E-05

ALMS1	113243949	-4.85053978	-2.27815	3.48E-06	2.41E-05
MPZL3	113260004	-4.85636967	-2.27988	0.00243	0.008338
LOC113247266	113247266	-4.85796843	-2.28035	7.47E-05	0.000385
LOC113257657	113257657	-4.85852725	-2.28052	0.001284	0.004794
MNS1	113269659	-4.86255681	-2.28172	0.000256	0.001159
RP2	113265987	-4.87469312	-2.28531	5.3E-09	6.11E-08
ALDH16A1	113243301	-4.88413825	-2.2881	9.82E-10	1.32E-08
ERAP2	113255061	-4.88429129	-2.28815	4.6E-08	4.52E-07
PPOX	113246728	-4.89968964	-2.29269	7.91E-08	7.47E-07
RNPEP	113242543	-4.90133629	-2.29318	2.55E-09	3.15E-08
DNASE1L3	113257014	-4.90681401	-2.29479	0.001351	0.005013
ARMH4	113242325	-4.91474418	-2.29712	0.000273	0.001228
MAPK13	113243854	-4.91583591	-2.29744	9.94E-06	6.29E-05
SH3BGRL	113242157	-4.91675586	-2.29771	3.8E-08	3.78E-07
HDAC1	113268357	-4.93141192	-2.302	2.46E-07	2.1E-06
MFSD12	113242802	-4.95953768	-2.31021	4.01E-09	4.72E-08
ARPC3	113266963	-4.9620916	-2.31095	1.98E-07	1.72E-06
PFN1	113271075	-4.96985098	-2.3132	2.42E-05	0.000139
ARHGAP19	113251776	-4.97329841	-2.3142	1.98E-06	1.43E-05
BLVRA	113257951	-4.97557496	-2.31486	9.41E-10	1.27E-08
IQCD	113266992	-4.97949272	-2.316	0.000185	0.000871
TYK2	113242985	-4.98174544	-2.31665	9.33E-07	7.16E-06
IFI30	113256708	-4.99042006	-2.31916	3.25E-08	3.26E-07
IDO1	113245545	-4.99086004	-2.31929	0.005398	0.016638
AGPAT2	113248634	-5.00456762	-2.32325	8.25E-05	0.000422
FECH	113253451	-5.00788914	-2.3242	0.000333	0.001462
CRLF3	113268998	-5.00908022	-2.32455	2.7E-09	3.31E-08
IGFBP2	113250479	-5.02843392	-2.33011	0.000879	0.003439

CENPQ	113261211	-5.03269704	-2.33133	9.01E-06	5.75E-05
LOC113245122	113245122	-5.04662653	-2.33532	1.09E-05	6.81E-05
BLM	113263720	-5.04663779	-2.33532	3.28E-06	2.28E-05
SHKBP1	113243561	-5.0488328	-2.33595	6.07E-10	8.47E-09
RUNDC3A	113265355	-5.05653113	-2.33815	7.23E-05	0.000375
RPE	113250510	-5.06251598	-2.33985	1.18E-09	1.56E-08
SAMD9L	113259987	-5.0684277	-2.34154	5.94E-09	6.78E-08
LOC113248368	113248368	-5.08184901	-2.34535	1.84E-05	0.000109
BLNK	113251766	-5.08246225	-2.34553	8.35E-06	5.37E-05
RNF149	113247198	-5.09197364	-2.34822	5.27E-07	4.22E-06
SMIM5	113244631	-5.09341454	-2.34863	0.000873	0.003419
KLB	113262421	-5.09693765	-2.34963	0.000266	0.001198
CUNH9orf40	113269847	-5.1041085	-2.35166	1.58E-07	1.41E-06
PLAUR	113243488	-5.10527905	-2.35199	2.49E-06	1.77E-05
SLC15A3	113246988	-5.1099094	-2.3533	9.74E-09	1.07E-07
SLC1A5	113243390	-5.1151607	-2.35478	2.57E-09	3.16E-08
HAVCR1	113264012	-5.11671166	-2.35522	1.1E-05	6.88E-05
LPL	113270022	-5.13840044	-2.36132	0.010894	0.029999
WIF1	113256324	-5.15710655	-2.36656	0.000596	0.002449
KLRD1	113257897	-5.16897949	-2.36988	3.01E-05	0.000169
TTC7A	113245984	-5.16989753	-2.37014	1.28E-08	1.37E-07
RAB11FIP4	113269005	-5.1759279	-2.37182	0.000357	0.001557
TAP2	113243780	-5.18055245	-2.37311	6.91E-09	7.78E-08
LOC113243784	113243784	-5.1833694	-2.37389	1.49E-07	1.33E-06
LXN	113254121	-5.18987876	-2.3757	8.49E-09	9.46E-08
FNDC10	113270684	-5.19286442	-2.37653	6.67E-07	5.25E-06
LOXL3	113243508	-5.19351395	-2.37671	1.98E-05	0.000116
MSRA	113269936	-5.20192177	-2.37904	5.16E-10	7.32E-09

CENPM	113241713	-5.20431254	-2.37971	1.71E-07	1.51E-06
F13A1	113264421	-5.20563993	-2.38008	7.5E-10	1.03E-08
PTPRCAP	113244029	-5.2109879	-2.38156	8.08E-07	6.27E-06
LOC113247025	113247025	-5.21151771	-2.3817	0.000434	0.001846
LOC113258964	113258964	-5.21385449	-2.38235	1.73E-08	1.82E-07
PTPRE	113252056	-5.21535037	-2.38276	1.22E-08	1.33E-07
CDK2	113256451	-5.21645411	-2.38307	9.97E-08	9.19E-07
ABTB1	113257272	-5.21928959	-2.38385	5.2E-07	4.17E-06
LOC113253437	113253437	-5.22163018	-2.3845	0.00155	0.005653
RNASEL	113262726	-5.22955394	-2.38669	4.91E-09	5.72E-08
LOC113244439	113244439	-5.23107253	-2.38711	7.39E-05	0.000382
CLIC1	113243757	-5.23119889	-2.38714	1.3E-07	1.17E-06
TAF8	113261300	-5.23135485	-2.38718	3.54E-07	2.92E-06
NMI	113250889	-5.23662703	-2.38864	1.37E-09	1.79E-08
LYN	113268089	-5.2509535	-2.39258	2.99E-09	3.63E-08
LOC113264274	113264274	-5.25228394	-2.39294	1.84E-05	0.000109
MARCH8	113243122	-5.25565708	-2.39387	4.14E-08	4.1E-07
HES6	113250049	-5.26073291	-2.39526	0.000101	0.000507
LOC113264493	113264493	-5.2615335	-2.39548	1.25E-08	1.35E-07
PON2	113260347	-5.26306792	-2.3959	8.45E-07	6.53E-06
SLC6A4	113269085	-5.26732044	-2.39707	4.62E-07	3.75E-06
TSPO	113241678	-5.27675079	-2.39965	1.62E-05	9.72E-05
ST6GAL1	113253796	-5.27898845	-2.40026	5.33E-08	5.18E-07
RCC2	113270442	-5.28257508	-2.40124	2.74E-08	2.77E-07
SPTLC2	113270285	-5.28536019	-2.402	7.92E-08	7.48E-07
CD3D	113260002	-5.2895822	-2.40315	4.36E-05	0.000236
RPP25	113240982	-5.29163685	-2.40371	7.44E-05	0.000384
LOC113249521	113249521	-5.30337679	-2.40691	2.47E-05	0.000141

LOC113249511	113249511	-5.30371017	-2.407	1.44E-06	1.06E-05
DCLRE1C	113241390	-5.30481391	-2.4073	9.84E-08	9.07E-07
MOB3A	113242756	-5.3048879	-2.40732	1.19E-09	1.57E-08
LOC113246872	113246872	-5.30900289	-2.40844	8.13E-06	5.23E-05
FANCL	113245380	-5.31026317	-2.40878	1.59E-07	1.42E-06
LOC113251097	113251097	-5.31352808	-2.40967	4.42E-07	3.6E-06
PABPC1	113252872	-5.32278173	-2.41218	0.000713	0.00287
METTL4	113253257	-5.33386783	-2.41518	2.17E-05	0.000126
SLC37A2	113259908	-5.33492041	-2.41547	6.55E-06	4.31E-05
LRRC8D	113254428	-5.33976429	-2.41678	1.38E-09	1.8E-08
CX3CR1	113253798	-5.34073747	-2.41704	6.01E-08	5.77E-07
IDNK	113269814	-5.34440218	-2.41803	1.29E-06	9.63E-06
TEC	113262469	-5.35109797	-2.41983	7E-06	4.58E-05
BATF3	113262678	-5.35316844	-2.42039	0.000118	0.00058
SATB2	113250620	-5.35763427	-2.4216	0.006779	0.02016
SPIDR	113268121	-5.36059326	-2.42239	2.35E-07	2.01E-06
PIK3AP1	113251772	-5.36990621	-2.4249	1.13E-07	1.02E-06
TESC	113267012	-5.38090656	-2.42785	0.000266	0.001199
ITGB3BP	113254585	-5.38174549	-2.42807	2.39E-06	1.7E-05
BRI3BP	113266871	-5.38305837	-2.42843	2.48E-10	3.73E-09
TALDO1	113248794	-5.38610837	-2.42924	3.52E-06	2.44E-05
LOC113241124	113241124	-5.38633768	-2.4293	0.000469	0.001978
ADAP1	113247735	-5.38654655	-2.42936	2.48E-06	1.76E-05
VPS26B	113259848	-5.39491865	-2.4316	1.69E-07	1.49E-06
SP110	113250368	-5.39730349	-2.43224	1.22E-06	9.14E-06
CAPNS2	113252251	-5.39958694	-2.43285	0.000417	0.001783
FANCG	113260492	-5.42669666	-2.44007	2.47E-08	2.53E-07
ALPL	113268551	-5.44000123	-2.44361	0.007587	0.022202

EXD1	113242013	-5.44308994	-2.44443	0.001595	0.005793
DNM3	113262829	-5.44478952	-2.44488	4.64E-09	5.42E-08
CFL1	113244093	-5.45162687	-2.44669	3.61E-05	0.000199
NCAPG2	113241531	-5.45234196	-2.44688	1.77E-08	1.86E-07
ADORA2B	113271262	-5.47009774	-2.45157	8.61E-06	5.52E-05
ADCY7	113252223	-5.47609411	-2.45315	1.7E-08	1.8E-07
CRELD2	113241628	-5.48075868	-2.45438	6.52E-10	9.05E-09
CD74	113263948	-5.4822713	-2.45477	2.6E-06	1.84E-05
SAT1	113242226	-5.48608164	-2.45578	1.56E-09	2.01E-08
LITAF	113270792	-5.49262102	-2.45749	4.26E-09	5E-08
FES	113263718	-5.50211183	-2.45999	2.55E-08	2.6E-07
CENPH	113255259	-5.50617238	-2.46105	2.61E-08	2.66E-07
CIITA	113270802	-5.51392386	-2.46308	1.55E-07	1.39E-06
PDLIM2	113269998	-5.51454325	-2.46324	8.48E-05	0.000433
TMPO	113256114	-5.51773713	-2.46408	1.12E-05	7E-05
GPR183	113251493	-5.53036199	-2.46737	0.000146	0.000702
SEPT5	113245887	-5.5306652	-2.46745	1.78E-09	2.26E-08
F12	113264151	-5.53746803	-2.46923	4.57E-07	3.71E-06
TDRD6	113261231	-5.55682767	-2.47426	2.16E-05	0.000126
PPARG	113257185	-5.5588066	-2.47478	9.99E-06	6.31E-05
CHAC2	113245835	-5.56879187	-2.47736	4.46E-05	0.000241
KIF20B	113243153	-5.56890039	-2.47739	1.17E-07	1.06E-06
CAP1	113268237	-5.57529487	-2.47905	5.56E-06	3.71E-05
SNCG	113259249	-5.5768937	-2.47946	0.011843	0.032267
RASGRP1	113242056	-5.58624475	-2.48188	5.01E-05	0.000268
LOXL2	113269985	-5.58858814	-2.48248	0.002148	0.007495
CARD9	113248628	-5.59109138	-2.48313	4.35E-05	0.000235
FANCF	113265106	-5.6027188	-2.48613	1.89E-07	1.65E-06

TNFRSF14	113270606	-5.60376831	-2.4864	1.14E-07	1.04E-06
AP3S1	113261609	-5.609922	-2.48798	4.96E-09	5.77E-08
NIN	113266216	-5.61136155	-2.48835	3.85E-09	4.56E-08
FLVCR2	113270311	-5.61699763	-2.4898	0.000145	0.000698
RETSAT	113243209	-5.62926739	-2.49295	7.4E-07	5.79E-06
CPVL	113262172	-5.63304453	-2.49391	3.94E-07	3.23E-06
KCNK3	113270882	-5.63434319	-2.49425	0.003744	0.012107
RAB20	113251540	-5.65927048	-2.50062	1.61E-05	9.67E-05
R3HDM4	113242708	-5.65958452	-2.5007	4.05E-06	2.78E-05
CUNH11orf86	113244046	-5.66033881	-2.50089	0.008949	0.025438
SDF2L1	113247034	-5.66386158	-2.50179	1.56E-07	1.4E-06
OTULINL	113260832	-5.66854022	-2.50298	5.87E-10	8.22E-09
FXYD5	113244829	-5.6724005	-2.50396	1.24E-09	1.62E-08
FAM122B	113270975	-5.68029534	-2.50597	3.24E-08	3.26E-07
SLC16A7	113256349	-5.69845455	-2.51057	6.21E-09	7.07E-08
LOC113248579	113248579	-5.70075349	-2.51115	1.44E-05	8.76E-05
ADCY5	113252326	-5.70627411	-2.51255	4.04E-05	0.00022
VRK1	113267288	-5.70762212	-2.51289	5.73E-08	5.53E-07
LPIN2	113253253	-5.71461188	-2.51466	2.58E-09	3.17E-08
ASNS	113260459	-5.71641494	-2.51511	4.68E-09	5.47E-08
ARHGEF1	113243505	-5.71902836	-2.51577	1.55E-06	1.14E-05
IFITM5	113248811	-5.72280433	-2.51672	0.003623	0.011784
FRAT2	113251778	-5.72526443	-2.51734	1.04E-08	1.14E-07
GALNT7	113269901	-5.7266051	-2.51768	1.73E-08	1.82E-07
XRCC2	113241505	-5.73055529	-2.51867	1.39E-05	8.51E-05
RTP4	113253777	-5.73376238	-2.51948	1.25E-06	9.34E-06
PADI3	113270444	-5.73969842	-2.52097	9.04E-08	8.41E-07
CITED4	113254823	-5.74011599	-2.52108	1.74E-07	1.53E-06

UBASH3B	113259933	-5.74487326	-2.52228	2.43E-07	2.07E-06
VSIG4	113246902	-5.7607354	-2.52625	1.25E-09	1.64E-08
APAF1	113256111	-5.78177761	-2.53151	1.13E-07	1.03E-06
MDM1	113256303	-5.78342924	-2.53193	6.58E-07	5.19E-06
FAM111B	113246546	-5.78460886	-2.53222	5.3E-10	7.51E-09
SLC2A8	113266423	-5.79155392	-2.53395	2.25E-08	2.31E-07
LOC113243779	113243779	-5.79224903	-2.53412	6.39E-05	0.000335
BASP1	113260843	-5.79976727	-2.536	0.000298	0.001329
CXCL12	113243131	-5.80711277	-2.53782	0.002408	0.008263
MFHAS1	113262185	-5.81103731	-2.5388	3.31E-09	3.98E-08
ELOVL1	113254789	-5.81458024	-2.53968	5.13E-10	7.29E-09
ADGRG3	113252291	-5.81605192	-2.54004	2.15E-06	1.54E-05
IL21R	113267611	-5.82886372	-2.54321	4.03E-05	0.000219
GCLC	113261177	-5.83271288	-2.54417	5.06E-07	4.07E-06
MGAT5	113263431	-5.84396849	-2.54695	8.57E-08	8.01E-07
LOC113246352	113246352	-5.84413289	-2.54699	5.3E-06	3.55E-05
CDR2	113267558	-5.85186687	-2.5489	3.12E-08	3.14E-07
ADRB1	113251957	-5.86022533	-2.55096	2.46E-05	0.000141
CUNH15orf39	113240987	-5.86725296	-2.55269	1.88E-07	1.64E-06
CSRP2	113256237	-5.87020945	-2.55341	0.001957	0.006897
PLA2G4A	113262763	-5.87734966	-2.55517	1.02E-10	1.64E-09
DSCC1	113252776	-5.88649084	-2.55741	5.59E-10	7.88E-09
BPGM	113264369	-5.88863169	-2.55793	1.43E-06	1.06E-05
SLC35F1	113259662	-5.89406857	-2.55926	1.92E-05	0.000113
GPCPD1	113258363	-5.91237797	-2.56374	1.91E-09	2.42E-08
CAMK4	113254999	-5.91406071	-2.56415	7.72E-05	0.000397
FAM166B	113260482	-5.9148024	-2.56433	0.001519	0.005555
RHOG	113269253	-5.92020885	-2.56565	5.81E-10	8.15E-09

LOC113247364	113247364	-5.92766282	-2.56746	3.42E-05	0.000189
IL16	113263766	-5.95149247	-2.57325	2.1E-07	1.81E-06
ARPC5	113262738	-5.96087362	-2.57552	2.12E-07	1.83E-06
RUNX1	113262125	-5.9807967	-2.58034	1.28E-05	7.87E-05
MST1	113256881	-5.98140111	-2.58048	9.17E-05	0.000463
LOC113261057	113261057	-5.98278914	-2.58082	1.65E-09	2.11E-08
CUNH6orf141	113261209	-5.99070208	-2.58273	0.000244	0.00111
LOC113252091	113252091	-5.99752313	-2.58437	2.94E-07	2.47E-06
PHF7	113256952	-5.99805684	-2.5845	5.57E-06	3.71E-05
ZNF367	113269774	-5.99924195	-2.58478	5.82E-09	6.66E-08
ISM1	113258332	-6.00117695	-2.58525	1.45E-05	8.81E-05
PADI6	113270662	-6.00179004	-2.58539	2.86E-05	0.000162
TUBAL3	113241127	-6.0038978	-2.5859	4.67E-05	0.000251
WDR90	113245385	-6.00760753	-2.58679	2.68E-07	2.27E-06
TCIRG1	113244006	-6.00869453	-2.58705	1.06E-08	1.16E-07
ACTN1	113270401	-6.01185479	-2.58781	1.13E-05	7.05E-05
CKAP4	113256046	-6.01301463	-2.58809	1.43E-06	1.06E-05
EPB41	113268397	-6.01559381	-2.58871	4.3E-07	3.51E-06
CD44	113265043	-6.03151344	-2.59252	8.48E-10	1.15E-08
SPINT1	113242021	-6.04544369	-2.59585	2.34E-06	1.67E-05
CD68	113271154	-6.04663989	-2.59613	3.15E-05	0.000176
SLC2A6	113266569	-6.04984019	-2.5969	1.71E-08	1.81E-07
TXNRD3	113257278	-6.05094601	-2.59716	0.000658	0.002674
RBBP8	113253281	-6.05580915	-2.59832	1.46E-09	1.89E-08
SYN2	113257181	-6.05977828	-2.59927	0.000475	0.002002
ALDH1L2	113255960	-6.0733673	-2.6025	2.43E-05	0.000139
SPP1	113263166	-6.08425603	-2.60508	0.007331	0.021542
ALDH18A1	113251756	-6.08938925	-2.6063	3.06E-10	4.53E-09

TMC6	113244576	-6.09473715	-2.60756	2.14E-08	2.21E-07
LPXN	113246554	-6.09592711	-2.60785	1.96E-08	2.05E-07
LOC113248575	113248575	-6.09984353	-2.60877	1.38E-05	8.44E-05
TNFRSF1B	113270492	-6.10330235	-2.60959	4.75E-11	8.09E-10
CENPX	113244490	-6.11195434	-2.61163	5.69E-09	6.52E-08
BGLAP	113245244	-6.12168943	-2.61393	0.008404	0.024146
IDH1	113250517	-6.12383634	-2.61444	5.27E-08	5.12E-07
GPBAR1	113250466	-6.12884825	-2.61562	1.33E-05	8.15E-05
GPR152	113243918	-6.12909574	-2.61567	0.000478	0.002015
TUBB	113264352	-6.14904452	-2.62036	0.000188	0.000879
CPA3	113254040	-6.15432098	-2.6216	1.02E-06	7.79E-06
NME4	113245373	-6.16388571	-2.62384	2.09E-07	1.81E-06
MXD1	113244487	-6.16788072	-2.62477	4.33E-06	2.95E-05
CAPG	113243184	-6.17072593	-2.62544	2.85E-10	4.26E-09
SCPEP1	113268848	-6.17221132	-2.62579	2.89E-10	4.32E-09
PYCR1	113244492	-6.17548972	-2.62655	9.35E-08	8.65E-07
MXD3	113264140	-6.18266939	-2.62823	7.52E-08	7.14E-07
IRF8	113252179	-6.18882236	-2.62966	7.51E-10	1.03E-08
RGS14	113264145	-6.18958748	-2.62984	1.93E-07	1.68E-06
MARCH2	113242900	-6.19047282	-2.63005	9.54E-10	1.28E-08
SCML2	113242257	-6.19562002	-2.63125	1.63E-05	9.78E-05
DBF4B	113265346	-6.20531641	-2.6335	0.000101	0.000508
LOC113250367	113250367	-6.20691468	-2.63388	1.17E-10	1.86E-09
CAPZA1	113244421	-6.20777246	-2.63408	1.83E-08	1.92E-07
ADM5	113243288	-6.21088745	-2.6348	2.35E-05	0.000136
ACER3	113269345	-6.21231281	-2.63513	1.96E-10	3E-09
ZNF710	113263703	-6.21769206	-2.63638	3.98E-09	4.69E-08
ELF4	113270951	-6.21990362	-2.63689	3.49E-07	2.89E-06

LOC113244473	113244473	-6.22263235	-2.63753	1.24E-10	1.95E-09
LOC113243777	113243777	-6.22986014	-2.6392	5.59E-08	5.4E-07
POLQ	113252082	-6.23344885	-2.64003	6.36E-06	4.19E-05
EMILIN2	113253252	-6.24019225	-2.64159	1.74E-07	1.53E-06
DGKA	113256448	-6.24067975	-2.6417	8.84E-09	9.83E-08
SLC12A9	113267841	-6.24178195	-2.64196	5.67E-07	4.52E-06
CD5	113246919	-6.25771373	-2.64564	0.000112	0.000555
KIF24	113260654	-6.25961457	-2.64607	5.28E-05	0.000282
ENO1	113270547	-6.26020402	-2.64621	1.62E-05	9.71E-05
ANKRD44	113250630	-6.27020007	-2.64851	5.61E-09	6.43E-08
LOC113246145	113246145	-6.27185488	-2.64889	5.57E-08	5.39E-07
SLC39A11	113265219	-6.28228164	-2.65129	7.91E-10	1.08E-08
CIDEA	113245954	-6.28565602	-2.65206	0.000359	0.001565
FKBP11	113257665	-6.28880761	-2.65279	3.22E-08	3.24E-07
POLD1	113247242	-6.30356309	-2.65617	4.97E-09	5.77E-08
OMP	113269350	-6.3236411	-2.66076	8.61E-05	0.000438
GALNS	113252143	-6.32891102	-2.66196	3.92E-10	5.71E-09
SECTM1	113244474	-6.33093652	-2.66242	1.27E-05	7.85E-05
NANP	113248364	-6.33193416	-2.66265	1.61E-06	1.18E-05
NPY	113261579	-6.33406532	-2.66313	0.000911	0.00355
LOC113249383	113249383	-6.33409584	-2.66314	1.75E-05	0.000104
VWA5A	113259924	-6.33655366	-2.6637	4.3E-11	7.37E-10
DDX11	113258026	-6.34366512	-2.66532	2.7E-08	2.74E-07
DGAT1	113247638	-6.35514609	-2.66793	3.1E-09	3.75E-08
VAV3	113244955	-6.35753519	-2.66847	7.58E-09	8.49E-08
CENPP	113270095	-6.36665245	-2.67054	9.99E-06	6.31E-05
HMBS	113259975	-6.37632331	-2.67272	3.11E-06	2.17E-05
GCH1	113242352	-6.37780163	-2.67306	6.87E-05	0.000358

PCBP3	113264451	-6.39299768	-2.67649	7.46E-06	4.85E-05
CTSS	113246210	-6.41422357	-2.68127	1.39E-08	1.49E-07
LRFN1	113243595	-6.4196688	-2.6825	2.56E-07	2.17E-06
RFLNB	113269104	-6.43076837	-2.68499	2.41E-05	0.000139
GPR160	113255265	-6.43214401	-2.6853	8.54E-07	6.59E-06
SIRPA	113258419	-6.43459476	-2.68585	4.45E-07	3.62E-06
NODAL	113259143	-6.44998452	-2.6893	0.000711	0.002864
CD209	113242920	-6.46336178	-2.69228	0.002356	0.008102
LOC113250070	113250070	-6.46491515	-2.69263	3.07E-10	4.54E-09
KCTD19	113252361	-6.46769252	-2.69325	1.28E-05	7.88E-05
IL24	113242598	-6.4701191	-2.69379	0.000417	0.001782
ACP5	113243033	-6.47558304	-2.69501	0.000172	0.000818
RFFL	113268972	-6.48214073	-2.69647	1.23E-12	2.85E-11
LOC113244203	113244203	-6.48895768	-2.69799	1.7E-09	2.17E-08
CMTM7	113253837	-6.49367082	-2.69903	5.86E-08	5.64E-07
LOC113243775	113243775	-6.49459945	-2.69924	9.14E-09	1.01E-07
PRR13	113257552	-6.49701369	-2.69978	1.74E-08	1.83E-07
BICDL1	113267031	-6.49855568	-2.70012	1.51E-05	9.11E-05
C2	113243740	-6.50572834	-2.70171	1.63E-05	9.74E-05
LOC113260124	113260124	-6.50728055	-2.70205	1.66E-11	3.11E-10
TTLL12	113241677	-6.51181769	-2.70306	4.04E-08	4E-07
ADORA2A	113247074	-6.52248946	-2.70542	7.78E-07	6.05E-06
ISG15	113270654	-6.52853944	-2.70676	2.77E-08	2.81E-07
CEP152	113269595	-6.52865591	-2.70679	6.25E-07	4.95E-06
SLC9B2	113263091	-6.54120911	-2.70956	0.000137	0.000663
S100B	113263954	-6.54580071	-2.71057	0.000287	0.001283
LOC113267526	113267526	-6.56619941	-2.71506	1.25E-06	9.32E-06
HSD17B12	113265017	-6.56890901	-2.71565	9.42E-09	1.04E-07

LOC113243778	113243778	-6.57034208	-2.71597	2.06E-10	3.14E-09
SKAP2	113261826	-6.5764553	-2.71731	5.15E-11	8.71E-10
TIFA	113255582	-6.58974596	-2.72022	2.19E-06	1.57E-05
EVI2A	113269010	-6.6003173	-2.72254	1.6E-07	1.43E-06
FRAT1	113251777	-6.6006976	-2.72262	3.87E-08	3.84E-07
TEKT1	113271340	-6.60677279	-2.72395	6.6E-05	0.000344
FADS6	113244678	-6.61023218	-2.7247	0.000268	0.001208
DTL	113262670	-6.62831823	-2.72864	3.71E-08	3.69E-07
LOC113249622	113249622	-6.63100528	-2.72923	0.001674	0.006033
ALAD	113266319	-6.63418789	-2.72992	7.54E-12	1.51E-10
SGMS2	113255540	-6.63450706	-2.72999	6.16E-05	0.000324
PIM1	113243874	-6.64644494	-2.73258	1.2E-08	1.31E-07
MFSD9	113247474	-6.64858116	-2.73305	3.83E-09	4.54E-08
CNTRL	113266355	-6.64986302	-2.73332	1.6E-07	1.42E-06
LGALS9	113269017	-6.67299071	-2.73833	2.53E-12	5.53E-11
NEK3	113251290	-6.67345715	-2.73843	1.65E-08	1.75E-07
HMOX1	113256008	-6.68220336	-2.74032	7.75E-08	7.34E-07
LOC113264219	113264219	-6.69253338	-2.74255	1.19E-05	7.38E-05
DOCK11	113241263	-6.72716036	-2.75	4.5E-10	6.45E-09
CASC1	113257791	-6.72917885	-2.75043	8.2E-07	6.35E-06
CCSAP	113259055	-6.73092678	-2.75081	1.23E-05	7.59E-05
CHAF1B	113260328	-6.73392403	-2.75145	4.37E-08	4.31E-07
SQLE	113252737	-6.75344163	-2.75562	1.67E-07	1.48E-06
ERI1	113262115	-6.77349486	-2.7599	1.37E-06	1.02E-05
STK26	113270961	-6.77600309	-2.76043	2.89E-08	2.92E-07
CUNH1orf162	113244429	-6.78922534	-2.76325	1.16E-05	7.23E-05
DYRK3	113242593	-6.79018156	-2.76345	5.98E-07	4.75E-06
GNLY	113243045	-6.79138302	-2.76371	0.000153	0.000734

MIIP	113270494	-6.82872101	-2.77162	1.34E-07	1.2E-06
FCMR	113242597	-6.83138986	-2.77218	4.53E-05	0.000244
VCAM1	113254345	-6.83463686	-2.77286	3.71E-05	0.000205
TREM2	113261318	-6.83546009	-2.77304	1.85E-07	1.62E-06
TMEM35B	113268316	-6.84976188	-2.77605	5.42E-10	7.67E-09
POU2F2	113243511	-6.85030107	-2.77617	1.04E-05	6.56E-05
SYTL3	113259605	-6.85690221	-2.77756	2.37E-07	2.03E-06
SEMA4B	113263706	-6.86357218	-2.77896	2.34E-09	2.92E-08
PFKFB4	113256839	-6.86401464	-2.77905	1.04E-07	9.54E-07
LOC113243776	113243776	-6.86425577	-2.7791	5.93E-10	8.29E-09
NABP1	113250648	-6.87264668	-2.78087	1.73E-08	1.82E-07
HACD2	113252377	-6.88078775	-2.78257	1.71E-06	1.25E-05
HPCAL1	113269824	-6.88267217	-2.78297	1.39E-12	3.2E-11
RASGRP2	113244161	-6.88333532	-2.78311	3.88E-09	4.59E-08
HIPK1	113244398	-6.88396163	-2.78324	1.16E-05	7.24E-05
ACLY	113265439	-6.88421673	-2.78329	2.7E-06	1.91E-05
ACSM3	113267499	-6.90600471	-2.78785	5.82E-06	3.86E-05
RAB32	113259529	-6.92150449	-2.79109	1.44E-12	3.3E-11
TLR1	113262411	-6.92170932	-2.79113	2.09E-08	2.16E-07
CD79B	113265279	-6.93409009	-2.79371	0.000333	0.001462
FMNL1	113265322	-6.93885716	-2.7947	7.48E-09	8.38E-08
LTB	113243712	-6.93916362	-2.79476	5.27E-05	0.000281
ORAI2	113267823	-6.94641184	-2.79627	4.6E-09	5.38E-08
BORA	113251415	-6.95663448	-2.79839	3.7E-10	5.41E-09
LY9	113246634	-6.96070947	-2.79923	4.84E-06	3.28E-05
SIGLEC1	113258394	-6.96680636	-2.8005	3.98E-09	4.69E-08
CYTH4	113241809	-6.98416498	-2.80409	7.98E-13	1.91E-11
MBOAT1	113264512	-7.01964292	-2.8114	1.42E-11	2.69E-10

CARMIL2	113252372	-7.02219864	-2.81192	0.000189	0.000886
SLC17A9	113258803	-7.02987353	-2.8135	2.9E-07	2.44E-06
TRAIP	113256889	-7.03130594	-2.81379	2.38E-08	2.44E-07
TSSK3	113268355	-7.03234815	-2.81401	7.28E-06	4.74E-05
LOC113261044	113261044	-7.05803285	-2.81927	2.38E-05	0.000137
LOC113260550	113260550	-7.06671679	-2.82104	4.59E-08	4.51E-07
DMTN	113270013	-7.0714712	-2.82201	4.13E-09	4.85E-08
KIAA1211	113262517	-7.09093107	-2.82598	2.48E-07	2.11E-06
HK1	113259128	-7.09751977	-2.82731	3.42E-08	3.43E-07
LPGAT1	113262668	-7.10057727	-2.82794	1.3E-10	2.04E-09
CELSR3	113256843	-7.11390823	-2.83064	3.95E-05	0.000216
TAB3	113265837	-7.11409759	-2.83068	2.42E-08	2.48E-07
CLEC4G	113242922	-7.12806277	-2.83351	0.00316	0.010462
XPO7	113270014	-7.12898774	-2.8337	3.93E-06	2.7E-05
TNFAIP6	113250888	-7.13689995	-2.8353	4.15E-07	3.4E-06
GALNT14	113240904	-7.1985405	-2.8477	6.88E-05	0.000358
LOC113247175	113247175	-7.20757527	-2.84951	2.36E-08	2.42E-07
LOC113243734	113243734	-7.21021863	-2.85004	9.47E-10	1.28E-08
SKA1	113253416	-7.21098321	-2.8502	2.67E-08	2.71E-07
SLC25A21	113266138	-7.23179979	-2.85435	3.24E-07	2.7E-06
LRRC25	113256717	-7.24021687	-2.85603	1.14E-08	1.25E-07
RUNX3	113268494	-7.24361315	-2.85671	3.75E-05	0.000206
LOC113243774	113243774	-7.24708057	-2.8574	5.57E-08	5.38E-07
ASRGL1	113246907	-7.27461856	-2.86287	1.95E-09	2.47E-08
SYNPR	113257036	-7.28161522	-2.86426	0.000783	0.003113
PRDX5	113244172	-7.28579107	-2.86509	2.52E-09	3.11E-08
CDCA4	113267185	-7.2913091	-2.86618	2.26E-09	2.83E-08
LOC113269787	113269787	-7.29707502	-2.86732	2.92E-07	2.45E-06

GIMAP6	113249233	-7.31632006	-2.87112	2.37E-08	2.43E-07
SEPT6	113241276	-7.33415723	-2.87463	5.97E-09	6.81E-08
SQOR	113269573	-7.33886031	-2.87556	4.39E-10	6.32E-09
PIP4P2	113252947	-7.35381848	-2.87849	2.65E-06	1.87E-05
FLT3	113251210	-7.35996862	-2.8797	3.08E-06	2.15E-05
LSS	113258959	-7.3695228	-2.88157	1.01E-09	1.36E-08
AP1G2	113246053	-7.38951918	-2.88548	2.47E-09	3.07E-08
PREX1	113258691	-7.38961347	-2.8855	1.02E-06	7.77E-06
LOC113245585	113245585	-7.39337629	-2.88623	3.67E-06	2.53E-05
RAB6B	113253951	-7.39750632	-2.88704	0.000152	0.000727
ARNTL2	113257775	-7.41385766	-2.89022	0.000235	0.001075
IKBKE	113242466	-7.41867924	-2.89116	1.46E-08	1.57E-07
SLC25A39	113265354	-7.41883458	-2.89119	3.84E-09	4.55E-08
SLX4IP	113258340	-7.44499593	-2.89627	4.15E-08	4.11E-07
LAT	113267622	-7.44738081	-2.89673	1.93E-06	1.39E-05
IQGAP2	113255190	-7.44876976	-2.897	2.35E-12	5.17E-11
LOC113270849	113270849	-7.4733408	-2.90175	2.81E-09	3.43E-08
ACSL5	113251936	-7.48463779	-2.90393	1.06E-11	2.08E-10
AIF1	113243717	-7.48749229	-2.90448	1.17E-13	3.22E-12
BSN	113256613	-7.48757397	-2.9045	4.88E-05	0.000262
GFRA3	113261787	-7.48897741	-2.90477	6.52E-08	6.25E-07
GPNMB	113261422	-7.50484848	-2.90782	5.96E-07	4.74E-06
SLC22A31	113252137	-7.52013223	-2.91076	0.000162	0.000772
C1QA	113268538	-7.52062381	-2.91085	1.94E-10	2.96E-09
LOC113258955	113258955	-7.52760522	-2.91219	9.23E-10	1.25E-08
MAP4K1	113243613	-7.53279764	-2.91319	2.86E-08	2.89E-07
ACTR3	113263434	-7.54348177	-2.91523	4.97E-08	4.85E-07
KCNK5	113243894	-7.55699267	-2.91781	8.86E-06	5.67E-05

MIS18BP1	113266179	-7.58614443	-2.92337	4.57E-08	4.49E-07
KLHL6	113254507	-7.60918398	-2.92774	3.76E-07	3.09E-06
HYAL3	113256904	-7.62292105	-2.93034	1.51E-07	1.35E-06
CD22	113244765	-7.63151057	-2.93197	2.41E-06	1.72E-05
SLC7A1	113251221	-7.6381996	-2.93323	9.7E-09	1.07E-07
CD86	113252170	-7.67795193	-2.94072	2.65E-12	5.74E-11
CD244	113246635	-7.68369365	-2.9418	1.38E-08	1.49E-07
RAB3IL1	113246962	-7.69003035	-2.94299	1.68E-11	3.15E-10
DUT	113269589	-7.69050775	-2.94308	3.78E-10	5.52E-09
LOC113261589	113261589	-7.69793893	-2.94447	2.74E-05	0.000156
LCK	113268359	-7.71504333	-2.94767	1.05E-06	7.98E-06
CNN2	113242715	-7.72022035	-2.94864	2.58E-07	2.19E-06
EPHX3	113248703	-7.72690047	-2.94989	8.37E-07	6.47E-06
BNIP3L	113269964	-7.72796547	-2.95009	7.05E-07	5.53E-06
VAMP1	113257980	-7.73969114	-2.95228	3.6E-09	4.28E-08
ZWILCH	113240888	-7.73973213	-2.95228	1.03E-08	1.14E-07
PRIM1	113256404	-7.7650396	-2.95699	7.57E-10	1.04E-08
CYSLTR1	113242149	-7.77139786	-2.95817	5.46E-09	6.27E-08
LOC113266598	113266598	-7.78193383	-2.96013	4.56E-07	3.7E-06
SERPINA3	113267307	-7.7856646	-2.96082	0.001558	0.005679
BMX	113240704	-7.80705727	-2.96478	3.26E-06	2.27E-05
PARP14	113252275	-7.81682215	-2.96658	8.85E-12	1.75E-10
ICAM5	113242980	-7.82217388	-2.96757	1.12E-05	7.01E-05
CD247	113262884	-7.84251233	-2.97132	3.59E-05	0.000198
PAG1	113253010	-7.87568399	-2.97741	8.89E-09	9.88E-08
LOC113252151	113252151	-7.89102289	-2.98021	1.84E-10	2.82E-09
GEN1	113270148	-7.891612	-2.98032	3.52E-09	4.2E-08
GIMAP2	113249228	-7.90437986	-2.98265	3.22E-07	2.68E-06

MFSD2B	113270408	-7.91658968	-2.98488	2.62E-07	2.22E-06
ADH4	113263120	-7.93445175	-2.98813	1.98E-05	0.000116
STK10	113264088	-7.94023427	-2.98918	2.29E-10	3.46E-09
PRKCD	113256975	-7.96272401	-2.99326	2.33E-10	3.51E-09
AADACL2	113254071	-8.00168619	-3.0003	4.84E-07	3.91E-06
TOMM34	113258645	-8.01587061	-3.00286	1.13E-06	8.52E-06
CIB1	113263707	-8.03293962	-3.00593	3.41E-14	1.03E-12
MAP7	113259482	-8.06735434	-3.0121	4.02E-09	4.73E-08
LOC113246321	113246321	-8.07827832	-3.01405	0.000454	0.001919
RAB27A	113269646	-8.0935582	-3.01677	1.95E-08	2.03E-07
EFHD2	113270478	-8.11506147	-3.0206	1.7E-09	2.17E-08
ANP32B	113260418	-8.12891142	-3.02306	1.03E-07	9.5E-07
IGSF22	113265118	-8.13463844	-3.02408	6.92E-08	6.6E-07
EIF2AK1	113267934	-8.13793432	-3.02466	9.12E-09	1.01E-07
AMPD3	113245788	-8.14498666	-3.02591	9.59E-06	6.07E-05
EPHB6	113265418	-8.16756166	-3.02991	1.85E-06	1.35E-05
C5AR2	113243375	-8.18464263	-3.03292	1.03E-05	6.51E-05
PANK2	113258385	-8.19792435	-3.03526	4.38E-13	1.1E-11
CENPO	113270597	-8.21262379	-3.03784	5.74E-07	4.57E-06
SH3BP1	113241803	-8.22759412	-3.04047	1.7E-10	2.62E-09
CUNH18orf54	113253433	-8.23002671	-3.0409	1.2E-09	1.57E-08
PSRC1	113244931	-8.24612406	-3.04372	3.71E-09	4.4E-08
CXCL10	113263254	-8.25393867	-3.04508	0.000247	0.001125
AURKA	113258749	-8.25893172	-3.04596	6.73E-09	7.59E-08
LRG1	113242844	-8.27056647	-3.04799	3.29E-13	8.41E-12
LOC113264440	113264440	-8.27288726	-3.04839	1.4E-08	1.5E-07
DNMT1	113242975	-8.27566293	-3.04887	5.58E-09	6.41E-08
LOC113250369	113250369	-8.28751104	-3.05094	9.91E-06	6.27E-05

HASPIN	113269162	-8.28971106	-3.05132	4.61E-10	6.6E-09
LOC113268656	113268656	-8.2897584	-3.05133	2.21E-13	5.84E-12
NIPSNAP1	113245017	-8.30219884	-3.05349	7.02E-06	4.59E-05
RAD54B	113252926	-8.32398837	-3.05727	7.41E-08	7.03E-07
PLXNC1	113256150	-8.3246675	-3.05739	1.18E-09	1.56E-08
ALPK1	113255579	-8.3293408	-3.0582	2.34E-11	4.27E-10
LOC113261839	113261839	-8.37945906	-3.06686	1.48E-06	1.09E-05
LY96	113253042	-8.38576547	-3.06794	8.03E-08	7.56E-07
PMAIP1	113253468	-8.38944568	-3.06858	2.62E-06	1.86E-05
LOC113245198	113245198	-8.39221429	-3.06905	8.96E-06	5.72E-05
NSL1	113262679	-8.4058844	-3.0714	6.49E-12	1.32E-10
CDC25A	113256823	-8.4198472	-3.07379	7.37E-08	7E-07
PCTP	113265657	-8.43365097	-3.07616	1.37E-13	3.74E-12
LOC113263203	113263203	-8.43998075	-3.07724	1.12E-05	7.02E-05
CCDC134	113241717	-8.44157676	-3.07751	5.77E-08	5.56E-07
ADGRE2	113247146	-8.45174231	-3.07925	2.14E-08	2.21E-07
STK4	113258646	-8.50946776	-3.08907	1.03E-10	1.66E-09
TRIP13	113260782	-8.51991403	-3.09084	1.35E-11	2.57E-10
ZNF467	113241465	-8.5400015	-3.09424	9.66E-07	7.4E-06
CD84	113246758	-8.57866254	-3.10075	7.53E-06	4.89E-05
LOC113248423	113248423	-8.58019361	-3.10101	1.17E-07	1.06E-06
TRAK2	113250589	-8.59887885	-3.10415	2.08E-07	1.8E-06
LOC113268942	113268942	-8.59963283	-3.10428	2.51E-12	5.49E-11
RASSF2	113258371	-8.61451717	-3.10677	9.86E-11	1.59E-09
LOC113259972	113259972	-8.61827019	-3.1074	1.54E-10	2.4E-09
LOC113248519	113248519	-8.63217147	-3.10972	2.89E-07	2.43E-06
LPCAT1	113260704	-8.64123004	-3.11124	4.92E-09	5.73E-08
SPATC1	113247628	-8.67261874	-3.11647	2.39E-06	1.7E-05

MIS18A	113266182	-8.6845702	-3.11845	1.87E-08	1.95E-07
LOC113242151	113242151	-8.69351136	-3.11994	1.05E-06	7.96E-06
GMIP	113256762	-8.71704338	-3.12384	1.82E-09	2.31E-08
PCK1	113258758	-8.72385701	-3.12497	0.00024	0.001095
IL1R2	113247309	-8.73910957	-3.12749	1.37E-11	2.61E-10
TONSL	113247697	-8.74897111	-3.12911	2.98E-14	9.07E-13
TTF2	113244375	-8.75374677	-3.1299	3.26E-11	5.74E-10
KCNN4	113243484	-8.75723301	-3.13048	6.63E-08	6.34E-07
ACRBP	113257970	-8.76073775	-3.13105	1.97E-06	1.42E-05
LOC113247089	113247089	-8.78811104	-3.13555	1.28E-08	1.38E-07
SLC46A2	113266298	-8.80209601	-3.13785	3.26E-09	3.92E-08
LOC113270817	113270817	-8.82538222	-3.14166	1.5E-07	1.34E-06
DNAH9	113271222	-8.84482383	-3.14483	2.13E-07	1.84E-06
CD2	113244378	-8.86667966	-3.14839	1.71E-07	1.51E-06
NSD2	113245739	-8.897537	-3.15341	2.05E-08	2.13E-07
SLC6A12	113258029	-8.90413156	-3.15447	0.000241	0.001101
CLEC2D	113257912	-8.93267059	-3.15909	3.12E-11	5.52E-10
CD180	113255271	-8.93637939	-3.15969	1.13E-07	1.02E-06
CD3G	113260001	-8.94662528	-3.16134	1.1E-06	8.34E-06
C5AR1	113243378	-8.94667849	-3.16135	3.61E-12	7.63E-11
OSCAR	113247418	-8.96349268	-3.16406	7.57E-06	4.92E-05
CLECL1	113257911	-8.96750248	-3.16471	5.66E-15	1.94E-13
KCTD17	113255976	-8.97660269	-3.16617	1.23E-08	1.33E-07
DUSP5	113251922	-8.98383	-3.16733	3.47E-07	2.87E-06
GNG2	113242381	-9.0026687	-3.17035	2.6E-09	3.19E-08
STX11	113259518	-9.01363084	-3.17211	4.09E-12	8.55E-11
EHD3	113240931	-9.02358073	-3.1737	6.19E-10	8.62E-09
MICAL1	113264746	-9.04185987	-3.17662	2.93E-10	4.36E-09

ALG8	113269365	-9.05139045	-3.17814	1.22E-11	2.36E-10
REEP4	113270009	-9.07820656	-3.18241	8.46E-11	1.38E-09
CLIC2	113248334	-9.08077464	-3.18282	1.07E-10	1.71E-09
CCDC180	113260648	-9.08140511	-3.18292	4.86E-05	0.000261
JAML	113260006	-9.08783682	-3.18394	4.09E-10	5.91E-09
CD300A	113265201	-9.10337434	-3.1864	1.15E-09	1.52E-08
PLBD1	113257859	-9.11240109	-3.18783	2.18E-07	1.87E-06
LOC113246754	113246754	-9.12726429	-3.19018	7.5E-07	5.86E-06
FCHO1	113256689	-9.16065222	-3.19545	2.83E-07	2.39E-06
HMGB3	113246801	-9.20829067	-3.20293	3.81E-14	1.14E-12
PLA2G7	113261232	-9.22401771	-3.2054	4.91E-12	1.01E-10
SLC25A1	113245967	-9.2255345	-3.20563	1.42E-05	8.63E-05
WDFY4	113243107	-9.23069269	-3.20644	3.26E-10	4.82E-09
LOC113267642	113267642	-9.24360009	-3.20845	0.000192	0.000896
BARD1	113250496	-9.24673363	-3.20894	7.14E-10	9.86E-09
MYO1G	113257755	-9.2513283	-3.20966	1.19E-10	1.88E-09
ST6GALNAC2	113244590	-9.26150516	-3.21125	1.46E-09	1.9E-08
ADA2	113258056	-9.27110887	-3.21274	2.98E-14	9.07E-13
LOC113256785	113256785	-9.28410901	-3.21476	3.41E-12	7.26E-11
CD163	113257937	-9.28598913	-3.21506	6.36E-14	1.83E-12
HCAR2	113266917	-9.28628504	-3.2151	3.43E-06	2.38E-05
DRAM1	113256083	-9.29115973	-3.21586	5.36E-12	1.1E-10
KCNAB2	113270580	-9.29789383	-3.2169	2.48E-09	3.07E-08
LOC113247069	113247069	-9.30039786	-3.21729	3.51E-09	4.19E-08
PIF1	113240856	-9.31575752	-3.21967	3.35E-08	3.36E-07
TMEM26	113259080	-9.33038122	-3.22194	1.47E-08	1.57E-07
GLT1D1	113266868	-9.35941286	-3.22642	1.11E-06	8.41E-06
SRI	113259569	-9.37620638	-3.229	5.33E-08	5.18E-07

CARHSP1	113270818	-9.47076786	-3.24348	4.8E-14	1.41E-12
GALNT6	113257605	-9.49673151	-3.24743	8.01E-10	1.09E-08
RGS10	113251994	-9.50314131	-3.2484	5.05E-09	5.85E-08
PIM2	113265933	-9.57526672	-3.25931	3.36E-07	2.79E-06
IL18	113260053	-9.58369195	-3.26058	8.51E-10	1.15E-08
PLCG2	113252535	-9.58557326	-3.26086	1.08E-09	1.44E-08
SPECC1	113271263	-9.5942104	-3.26216	2.76E-10	4.13E-09
AUNIP	113268479	-9.60027183	-3.26308	1.36E-09	1.77E-08
LAT2	113267780	-9.65085085	-3.27066	7.28E-11	1.21E-09
AOAH	113262793	-9.65220343	-3.27086	1.98E-12	4.39E-11
MSR1	113262166	-9.65378857	-3.2711	2.98E-05	0.000168
LOC113247388	113247388	-9.65508074	-3.27129	5.2E-09	5.99E-08
PC	113244047	-9.67579116	-3.27438	0.000316	0.0014
DNPH1	113261273	-9.68118028	-3.27518	8.22E-08	7.71E-07
LOC113268965	113268965	-9.6840391	-3.27561	1.34E-13	3.67E-12
B4GALT5	113258702	-9.69100562	-3.27665	6.31E-11	1.06E-09
SH3BGRL2	113261084	-9.69187191	-3.27678	4.47E-10	6.43E-09
COTL1	113252192	-9.70012664	-3.278	6.31E-10	8.77E-09
ARRB2	113271089	-9.70420096	-3.27861	5.82E-11	9.81E-10
CIDEC	113257151	-9.70481884	-3.2787	6.26E-05	0.000329
LPAR5	113257973	-9.70879874	-3.27929	4.25E-09	4.99E-08
AK7	113267291	-9.73113949	-3.28261	6.51E-05	0.00034
CD200R1	113251345	-9.73467326	-3.28313	3.81E-14	1.14E-12
BRCA1	113265384	-9.76449235	-3.28755	1.92E-12	4.3E-11
TET3	113243822	-9.79805367	-3.2925	2.94E-10	4.36E-09
IL7R	113260884	-9.82180376	-3.29599	3.58E-06	2.48E-05
RDH16	113256401	-9.8369038	-3.2982	4.19E-11	7.21E-10
HCLS1	113252106	-9.84415654	-3.29927	1.08E-11	2.11E-10

TNFAIP8L2	113246164	-9.84508399	-3.2994	3.27E-11	5.75E-10
RAP1GAP2	113269150	-9.84996599	-3.30012	4.98E-09	5.77E-08
PSD4	113242515	-9.85193037	-3.30041	1.57E-10	2.44E-09
NLRC5	113252270	-9.86691253	-3.3026	3.34E-13	8.53E-12
NEMP1	113255866	-9.88617496	-3.30541	1.9E-11	3.53E-10
ENPEP	113255569	-9.90414175	-3.30803	2.43E-08	2.48E-07
LOC113246660	113246660	-9.94109592	-3.3134	9.79E-14	2.75E-12
LOC113266557	113266557	-9.9488246	-3.31453	2.78E-07	2.34E-06
CD48	113246744	-9.96738423	-3.31721	5.61E-13	1.38E-11
FGD2	113243873	-9.97172262	-3.31784	1.29E-13	3.54E-12
FN3KRP	113244463	-10.0233858	-3.3253	3.64E-12	7.68E-11
DNA2	113259112	-10.0401568	-3.32771	1.24E-12	2.86E-11
PTGR1	113260318	-10.0574771	-3.3302	0	0
CHAF1A	113242832	-10.0647513	-3.33124	4.44E-16	1.8E-14
TICRR	113263689	-10.1098519	-3.33769	4.27E-13	1.08E-11
TRIM22	113248232	-10.1169548	-3.3387	2.42E-14	7.48E-13
LOC113243544	113243544	-10.121182	-3.33931	2.87E-08	2.91E-07
AMMECR1	113241238	-10.1275928	-3.34022	9.24E-08	8.56E-07
DOCK10	113250392	-10.158772	-3.34465	3.02E-12	6.5E-11
CD226	113253513	-10.1738553	-3.34679	3.71E-08	3.69E-07
C3	113242886	-10.1927841	-3.34948	3.77E-11	6.55E-10
LOC113270721	113270721	-10.1995438	-3.35043	1.03E-10	1.66E-09
RIPK3	113246058	-10.2056142	-3.35129	7.81E-10	1.07E-08
STMN1	113268477	-10.2186788	-3.35314	2.42E-09	3.01E-08
CHTF18	113245386	-10.2493314	-3.35746	4.79E-11	8.14E-10
SLAIN1	113251439	-10.2523788	-3.35789	6.84E-11	1.14E-09
CLDN4	113267786	-10.2576806	-3.35863	1.69E-07	1.49E-06
SERPINB1	113264389	-10.2599368	-3.35895	6.45E-09	7.32E-08

GALNT3	113250823	-10.2706029	-3.36045	2.97E-06	2.08E-05
FAM83D	113258601	-10.2762291	-3.36124	5.21E-10	7.39E-09
LOC113246717	113246717	-10.2857796	-3.36258	8.85E-08	8.24E-07
IRF5	113263966	-10.3303105	-3.36881	4.59E-13	1.15E-11
LOC113270720	113270720	-10.3348943	-3.36945	2.04E-08	2.12E-07
ACAP1	113271119	-10.340332	-3.37021	3.04E-08	3.07E-07
MCM5	113256007	-10.3615825	-3.37317	3.05E-14	9.28E-13
GIMAP8	113241470	-10.4237737	-3.38181	1.35E-10	2.11E-09
HACD4	113260582	-10.4507455	-3.38553	2.3E-10	3.48E-09
CYFIP2	113264018	-10.4591878	-3.3867	6.16E-06	4.08E-05
RIPOR2	113264323	-10.4632324	-3.38726	1.52E-08	1.62E-07
WEE1	113245775	-10.5305467	-3.39651	6.77E-10	9.36E-09
CENPI	113244300	-10.5524015	-3.3995	6.57E-11	1.1E-09
QPCT	113241400	-10.5560842	-3.4	1.92E-10	2.95E-09
DOCK5	113269972	-10.5953614	-3.40536	1.07E-12	2.5E-11
FAM83A	113252757	-10.5991175	-3.40587	7.54E-05	0.000389
SPSB2	113257955	-10.627822	-3.40977	6.91E-12	1.39E-10
SGK2	113258622	-10.6448545	-3.41208	7.46E-08	7.08E-07
CCL16	113268945	-10.6899768	-3.41819	4.78E-08	4.68E-07
FAM107B	113241394	-10.7252456	-3.42294	1.05E-08	1.15E-07
FCER1G	113246719	-10.7508033	-3.42637	4.37E-14	1.29E-12
GINS4	113245542	-10.7724481	-3.42927	8.15E-08	7.66E-07
C1QB	113268536	-10.7827042	-3.43065	7.97E-14	2.26E-12
ATP2A3	113269168	-10.8013949	-3.43315	8.66E-08	8.08E-07
SUSD1	113260312	-10.8268851	-3.43655	3.55E-09	4.23E-08
ARAP2	113262394	-10.8544541	-3.44022	3.76E-11	6.54E-10
STIL	113254706	-10.8580406	-3.44069	4.51E-12	9.41E-11
LGALS12	113246993	-10.8595073	-3.44089	2.8E-09	3.42E-08

PIK3CG	113258627	-10.8687087	-3.44211	4.74E-12	9.84E-11
RDM1	113265317	-10.8875785	-3.44461	4.26E-11	7.32E-10
CDCA7L	113261227	-10.8956973	-3.44569	1.19E-11	2.31E-10
WDR76	113241950	-10.9048531	-3.4469	2.4E-11	4.34E-10
ISG20	113263677	-10.9159857	-3.44837	2.09E-09	2.63E-08
UCP2	113269303	-10.9607216	-3.45427	2.13E-11	3.91E-10
MMP19	113256453	-10.9625185	-3.45451	3.33E-16	1.39E-14
C1QC	113268537	-10.9675181	-3.45517	5.28E-14	1.54E-12
RASGRP4	113243614	-10.9732171	-3.45591	4.54E-11	7.75E-10
PTPRJ	113264945	-11.030717	-3.46345	1.55E-10	2.41E-09
SH2D1B	113268669	-11.0480526	-3.46572	1.67E-09	2.14E-08
RASSF5	113242463	-11.060672	-3.46737	7.33E-11	1.21E-09
PGD	113270522	-11.0658001	-3.46804	5.33E-12	1.09E-10
PAPSS2	113258979	-11.0830803	-3.47029	2E-11	3.69E-10
TMEM154	113255761	-11.0861324	-3.47068	0	0
PHKA2	113242251	-11.1004343	-3.47254	1.84E-11	3.43E-10
RAD18	113257127	-11.1047762	-3.47311	7.93E-11	1.3E-09
NBEAL2	113256803	-11.114427	-3.47436	1.22E-08	1.32E-07
CDC25B	113258383	-11.2075421	-3.4864	1.64E-09	2.1E-08
CD7	113244475	-11.2419718	-3.49082	2.91E-07	2.45E-06
CSF2RB	113255980	-11.2873996	-3.49664	4.99E-11	8.45E-10
SELP	113262857	-11.2966051	-3.49782	1.66E-07	1.47E-06
CUNH15orf48	113269571	-11.3420549	-3.50361	5.32E-06	3.56E-05
RHOH	113262430	-11.4143044	-3.51277	9.81E-07	7.5E-06
TNFSF13B	113251533	-11.4974273	-3.52324	3.11E-13	7.96E-12
DBF4	113259531	-11.522577	-3.52639	7.63E-12	1.52E-10
MTHFD1L	113259560	-11.5444509	-3.52913	9.99E-16	3.81E-14
AKNA	113266330	-11.5445685	-3.52914	7.82E-10	1.07E-08

SPNS3	113271044	-11.5628652	-3.53143	5.7E-14	1.66E-12
KPNA2	113265264	-11.6597008	-3.54346	0	0
BTK	113244303	-11.6710201	-3.54486	1.24E-12	2.87E-11
PKMYT1	113245420	-11.7149066	-3.55027	1.62E-09	2.08E-08
NLRP3	113247462	-11.7698632	-3.55703	6.56E-11	1.1E-09
CLCN2	113254245	-11.8355825	-3.56506	9.97E-11	1.61E-09
ELOVL5	113261178	-11.8368761	-3.56522	2.17E-06	1.56E-05
CCR2	113256789	-11.8480121	-3.56657	8.38E-13	2E-11
LBR	113262908	-11.8631783	-3.56842	1.12E-07	1.02E-06
MPEG1	113246548	-11.8928376	-3.57202	1.3E-11	2.49E-10
FAM117A	113265606	-11.9520727	-3.57919	1.21E-11	2.34E-10
LOC113265672	113265672	-12.0289316	-3.58844	1.09E-07	9.94E-07
GIMAP4	113249230	-12.0406267	-3.58984	1.11E-16	4.96E-15
GPR132	113267184	-12.0677681	-3.59309	3.4E-08	3.41E-07
HSD17B7	113268675	-12.1562363	-3.60362	3.53E-11	6.16E-10
DENND1C	113242880	-12.1584087	-3.60388	7.55E-13	1.83E-11
ANO3	113265098	-12.2152636	-3.61061	2.69E-08	2.73E-07
SMC4	113254137	-12.2415877	-3.61372	3.88E-11	6.72E-10
GRK6	113264153	-12.2903065	-3.61945	1.21E-10	1.92E-09
SMOX	113258376	-12.2928046	-3.61974	4.75E-12	9.85E-11
CCDC125	113255255	-12.3157484	-3.62243	4.45E-11	7.61E-10
INPP5D	113250332	-12.3922554	-3.63137	1.72E-11	3.21E-10
TMEM71	113252707	-12.405871	-3.63295	3.17E-09	3.83E-08
BLVRB	113243564	-12.4387316	-3.63677	3.29E-10	4.86E-09
PSTPIP1	113241007	-12.4522929	-3.63834	2E-12	4.44E-11
GNA15	113242779	-12.4529256	-3.63841	8.66E-15	2.86E-13
ARHGAP11A	113242073	-12.4874683	-3.64241	3.1E-13	7.96E-12
GIMAP5	113249226	-12.5603706	-3.65081	3.91E-12	8.19E-11

TSTD3	113264801	-12.5679325	-3.65168	2.63E-09	3.22E-08
SIGLEC11	113243272	-12.5933319	-3.65459	2.2E-10	3.33E-09
FGD3	113270098	-12.6341201	-3.65925	3.06E-09	3.7E-08
KIF21B	113242564	-12.6364813	-3.65952	2.42E-08	2.48E-07
GAS2L3	113256099	-12.6857803	-3.66514	1.95E-13	5.22E-12
CUNH16orf54	113267671	-12.7363496	-3.67088	9.42E-11	1.52E-09
LAPTM5	113268393	-12.7598437	-3.67354	4.46E-10	6.42E-09
ATP7B	113251293	-12.7644876	-3.67406	4.72E-10	6.75E-09
TBCEL	113259945	-12.7681596	-3.67448	7.68E-13	1.85E-11
LOC113247386	113247386	-12.7998113	-3.67805	2.32E-14	7.22E-13
P2RY8	113240789	-12.8832705	-3.68743	2.97E-11	5.28E-10
DCLRE1B	113244400	-12.9012726	-3.68944	1.94E-12	4.34E-11
LOC113267500	113267500	-12.9031769	-3.68965	6.33E-13	1.55E-11
CD82	113265010	-12.9429794	-3.6941	5.55E-16	2.21E-14
TNFAIP8	113261627	-12.9926804	-3.69963	2.31E-14	7.2E-13
DEF6	113243839	-13.0099782	-3.70155	3.53E-10	5.19E-09
ESM1	113263920	-13.0197506	-3.70263	0.000252	0.001145
CUNHXorf21	113265831	-13.0305154	-3.70382	2.75E-09	3.36E-08
WNT2	113263284	-13.0831555	-3.70964	0.000294	0.001318
CDC7	113254416	-13.107207	-3.71229	7.08E-11	1.18E-09
AGPAT4	113259630	-13.1083188	-3.71241	3.77E-15	1.32E-13
GIMAP1	113249227	-13.1379692	-3.71567	2.68E-11	4.81E-10
GINS3	113252306	-13.1458244	-3.71653	4.18E-11	7.2E-10
LIG1	113243358	-13.1753966	-3.71977	1.05E-13	2.93E-12
PCNA	113258365	-13.1926138	-3.72166	6.07E-12	1.23E-10
FAM3B	113256873	-13.2074482	-3.72328	0.000103	0.000514
MBOAT2	113269649	-13.2408763	-3.72693	3.15E-06	2.2E-05
FAM169B	113263647	-13.24837	-3.72774	1.04E-08	1.14E-07

NRGN	113259917	-13.2664493	-3.72971	2.72E-08	2.76E-07
RGS19	113258832	-13.2676439	-3.72984	1.01E-11	1.97E-10
CDA	113268562	-13.2797313	-3.73115	2.22E-16	9.61E-15
MCTP1	113255083	-13.3027627	-3.73365	2.27E-10	3.44E-09
LOC113258593	113258593	-13.3060631	-3.73401	1.08E-12	2.52E-11
LOC113245565	113245565	-13.3075297	-3.73417	9.44E-07	7.23E-06
CCDC88B	113244170	-13.3126176	-3.73472	1.95E-11	3.6E-10
LOC113247389	113247389	-13.3764447	-3.74162	1.85E-10	2.84E-09
CHEK1	113259903	-13.408682	-3.7451	4.38E-11	7.5E-10
SELPLG	113267072	-13.4343195	-3.74785	6.66E-16	2.62E-14
POC1A	113256942	-13.4972774	-3.7546	8.15E-12	1.62E-10
GCLM	113254391	-13.5882712	-3.76429	1.59E-13	4.31E-12
GTSE1	113241642	-13.6625074	-3.77215	2.33E-11	4.25E-10
APMAP	113247940	-13.7231492	-3.77854	9.91E-13	2.35E-11
NNMT	113260035	-13.833772	-3.79012	1.47E-13	4E-12
LOC113249892	113249892	-13.86531	-3.79341	7.69E-10	1.05E-08
ARHGAP4	113247863	-13.8772719	-3.79465	9.25E-12	1.82E-10
PYGL	113266218	-13.9035479	-3.79738	2.06E-12	4.57E-11
E2F1	113258272	-13.9306824	-3.80019	0	0
LRP8	113254650	-13.9503696	-3.80223	1.36E-13	3.71E-12
MILR1	113265270	-14.0443521	-3.81192	1.4E-13	3.82E-12
CP	113254043	-14.0446658	-3.81195	5.54E-06	3.7E-05
GPSM3	113243768	-14.1583379	-3.82358	1.33E-11	2.54E-10
GPR65	113267388	-14.3073783	-3.83869	6.63E-10	9.19E-09
DNAJC6	113254567	-14.3298526	-3.84095	6.57E-09	7.43E-08
KNTC1	113266920	-14.3665411	-3.84464	1.17E-09	1.55E-08
LOC113247174	113247174	-14.3925563	-3.84725	1.6E-10	2.48E-09
LOC113266586	113266586	-14.4035232	-3.84835	2.81E-13	7.25E-12

RHNO1	113258018	-14.5021789	-3.8582	6.79E-14	1.94E-12
DMXL2	113269625	-14.5109461	-3.85907	4.44E-13	1.11E-11
LOC113247411	113247411	-14.5509756	-3.86304	3.21E-09	3.87E-08
IL17RA	113258053	-14.5543696	-3.86338	1.11E-09	1.48E-08
WDR72	113269641	-14.5987012	-3.86777	2.91E-10	4.34E-09
G6PD	113247831	-14.627836	-3.87064	0	0
RBM47	113262432	-14.6376953	-3.87162	2.64E-12	5.73E-11
APBB1IP	113248698	-14.6378952	-3.87164	6.88E-13	1.68E-11
B4GALT6	113253319	-14.6455783	-3.87239	2.99E-11	5.31E-10
ITGB3	113265309	-14.6499814	-3.87283	3.24E-08	3.25E-07
TARM1	113247408	-14.6658308	-3.87439	1.21E-08	1.32E-07
KCNJ15	113258392	-14.7623677	-3.88385	7.27E-09	8.17E-08
SASH3	113270945	-14.7925609	-3.8868	6.07E-14	1.76E-12
PTAFR	113268423	-14.7932813	-3.88687	9.73E-14	2.74E-12
ACPP	113253937	-14.8772648	-3.89504	7.61E-09	8.51E-08
LOC113249986	113249986	-14.8826212	-3.89556	1.1E-10	1.76E-09
EZH2	113241452	-14.9084214	-3.89806	6.88E-15	2.3E-13
ARID3A	113242710	-14.9194229	-3.89912	8.88E-12	1.75E-10
MEGF9	113266346	-14.9552719	-3.90258	5.25E-10	7.44E-09
TKT	113256977	-14.9726363	-3.90426	5.39E-08	5.24E-07
NCAPD3	113259851	-14.9770009	-3.90468	5.54E-14	1.62E-12
MCM3	113261196	-15.0030209	-3.90718	2.03E-13	5.39E-12
CLDND2	113247281	-15.0245136	-3.90925	3.38E-07	2.81E-06
ZWINT	113258993	-15.0600898	-3.91266	5.56E-12	1.14E-10
LRRC75B	113247077	-15.0811236	-3.91467	1.07E-09	1.43E-08
LOC113246651	113246651	-15.1131055	-3.91773	1.78E-08	1.87E-07
DOCK8	113270185	-15.1849092	-3.92457	2.07E-10	3.15E-09
LOC113247262	113247262	-15.2135827	-3.92729	1.52E-09	1.96E-08

CDKN3	113242358	-15.2163107	-3.92755	1.74E-09	2.22E-08
LOC113249395	113249395	-15.219929	-3.92789	7.06E-11	1.18E-09
CD3E	113260003	-15.2572134	-3.93142	2.29E-10	3.46E-09
LRMP	113257496	-15.2968598	-3.93516	1.12E-14	3.64E-13
FGR	113268434	-15.3206596	-3.93741	3.6E-12	7.63E-11
ARHGAP9	113256382	-15.3244954	-3.93777	1.28E-11	2.46E-10
INCENP	113246995	-15.3481123	-3.93999	2.33E-15	8.43E-14
TYROBP	113244840	-15.3502668	-3.94019	1.55E-13	4.2E-12
ABCA7	113242716	-15.4221358	-3.94693	2E-15	7.28E-14
LOC113245566	113245566	-15.4545569	-3.94996	6.26E-08	6E-07
MMP9	113258671	-15.4624721	-3.9507	0.00018	0.00085
PIK3R5	113271196	-15.4696022	-3.95136	1.44E-15	5.4E-14
DOK2	113270015	-15.4998125	-3.95418	3.33E-16	1.39E-14
BRIP1	113268903	-15.5159	-3.95568	3.2E-11	5.64E-10
IL1B	113242506	-15.5712262	-3.96081	1.53E-08	1.63E-07
LOC113258423	113258423	-15.5812949	-3.96174	9.64E-10	1.3E-08
DHCR24	113254624	-15.5869137	-3.96226	1.17E-12	2.71E-11
TSPAN2	113244388	-15.6080757	-3.96422	2.49E-11	4.48E-10
GCA	113250834	-15.6311751	-3.96635	2.95E-12	6.35E-11
ATAD5	113268997	-15.641235	-3.96728	1.54E-11	2.9E-10
CD53	113244899	-15.7002942	-3.97272	2.56E-14	7.87E-13
LIPA	113258966	-15.7179506	-3.97434	6.85E-11	1.14E-09
TNFSF13	113271161	-15.7331456	-3.97574	1.11E-16	4.96E-15
SNX22	113240845	-15.7801865	-3.98004	1.9E-11	3.53E-10
BIRC5	113244569	-15.8059599	-3.9824	3.33E-15	1.17E-13
CUNH5orf34	113260956	-15.8268161	-3.9843	4.5E-10	6.45E-09
ABRACL	113259498	-15.8406121	-3.98556	0	0
GMFG	113243594	-15.8586073	-3.98719	6.06E-14	1.76E-12

ARHGDIB	113257847	-15.8698502	-3.98822	7.71E-11	1.27E-09
TIMD4	113264011	-15.9324509	-3.9939	0	0
LCP2	113264079	-15.9418694	-3.99475	7.43E-13	1.8E-11
CUNH1orf112	113262852	-15.9781833	-3.99803	1.02E-12	2.4E-11
FGFBP1	113266709	-16.1127552	-4.01013	4.52E-08	4.44E-07
FANCA	113252122	-16.1209597	-4.01087	3.26E-12	6.98E-11
LOC113265197	113265197	-16.1276209	-4.01146	6.37E-09	7.24E-08
SEMA4A	113245245	-16.1304517	-4.01171	1.59E-10	2.47E-09
STAB2	113256071	-16.1707461	-4.01531	9.11E-11	1.48E-09
RPS6KA1	113268455	-16.1828868	-4.0164	6.04E-13	1.48E-11
PIK3CD	113270537	-16.2435192	-4.02179	7.07E-12	1.42E-10
PRAM1	113242897	-16.2530131	-4.02264	1.73E-09	2.21E-08
ART4	113257852	-16.3083265	-4.02754	2.39E-11	4.33E-10
CXCL6	113263278	-16.3834278	-4.03417	9.98E-08	9.19E-07
ARHGAP30	113246713	-16.4307205	-4.03832	4.6E-13	1.15E-11
TLR8	113240729	-16.5191387	-4.04607	0	0
NCAPD2	113257978	-16.5503824	-4.04879	1.91E-13	5.12E-12
FAM111A	113246397	-16.597933	-4.05293	4.44E-16	1.8E-14
TRPM2	113251377	-16.6155131	-4.05446	5.96E-08	5.73E-07
IL2RG	113245610	-16.7101388	-4.06265	3.55E-13	9.02E-12
DAPP1	113263114	-16.71944	-4.06345	1.08E-11	2.1E-10
NUP210	113257232	-16.7356114	-4.06485	2.05E-12	4.54E-11
SPDL1	113264074	-16.7445868	-4.06562	4.98E-13	1.24E-11
JCHAIN	113263303	-16.858743	-4.07543	1.83E-05	0.000108
RAD51AP1	113257998	-16.873309	-4.07667	2.04E-13	5.41E-12
PTPN6	113257950	-16.917408	-4.08044	1.27E-14	4.09E-13
LIMD2	113265290	-17.0065091	-4.08802	7.63E-11	1.26E-09
SLC16A14	113250370	-17.0480313	-4.09153	9.66E-15	3.16E-13

TEDC2	113245100	-17.1027276	-4.09615	3.41E-12	7.26E-11
SNX20	113252229	-17.134675	-4.09885	1.02E-11	1.99E-10
ADGRE1	113242890	-17.1413148	-4.09941	3.33E-16	1.39E-14
FANCD2	113257160	-17.143052	-4.09955	9.99E-16	3.81E-14
CDK6	113259981	-17.1632232	-4.10125	3.28E-11	5.76E-10
TRAF3IP3	113262647	-17.2393004	-4.10763	5.63E-12	1.15E-10
MPP2	113265371	-17.2625131	-4.10957	0	0
ARHGAP45	113242717	-17.3156121	-4.114	2.51E-10	3.78E-09
LOC113268943	113268943	-17.325824	-4.11485	0	0
PTPN22	113244404	-17.3409526	-4.11611	6.86E-08	6.54E-07
POLE	113266839	-17.3755871	-4.11899	0	0
SLC38A5	113265913	-17.404267	-4.12137	4.11E-15	1.42E-13
GIMAP7	113249231	-17.471849	-4.12696	0	0
PLEK	113244715	-17.475395	-4.12725	6.73E-12	1.36E-10
LYL1	113247212	-17.4936134	-4.12876	1.85E-12	4.16E-11
LOC113263118	113263118	-17.5123562	-4.1303	1.08E-07	9.87E-07
LOC113258421	113258421	-17.5228373	-4.13116	8.1E-12	1.61E-10
COL17A1	113251900	-17.5774754	-4.13566	6.69E-09	7.56E-08
RAB37	113244666	-17.6037708	-4.13781	1.25E-08	1.35E-07
SKA3	113251177	-17.6384231	-4.14065	2.33E-14	7.23E-13
NCF4	113255983	-17.7089683	-4.14641	0	0
RASAL3	113248723	-17.7868698	-4.15274	2.47E-11	4.45E-10
KCNA3	113244900	-17.7992397	-4.15374	4.06E-11	7.01E-10
LOC113243251	113243251	-17.8148052	-4.155	4.86E-06	3.29E-05
EMB	113260963	-17.8191743	-4.15536	2.91E-10	4.34E-09
TOM1L1	113265649	-17.8442354	-4.15739	1.66E-10	2.56E-09
SYK	113270132	-17.8806157	-4.16032	1.8E-13	4.84E-12
PIP5K1B	113270193	-17.9166264	-4.16323	1.27E-11	2.44E-10

LOC113244815	113244815	-17.9307696	-4.16437	1.98E-10	3.02E-09
FHDC1	113255765	-18.0573748	-4.17452	2.62E-13	6.81E-12
TAL1	113254708	-18.0635945	-4.17501	1.47E-10	2.29E-09
DOK3	113264158	-18.1108442	-4.17878	6.96E-13	1.7E-11
CSF2RA	113240793	-18.1114793	-4.17883	9.8E-13	2.33E-11
PTPRC	113242583	-18.1785058	-4.18416	6.72E-12	1.36E-10
MARCH1	113255820	-18.2663344	-4.19112	3.36E-11	5.88E-10
TBC1D10C	113244031	-18.5423432	-4.21275	3.88E-10	5.65E-09
YOD1	113242605	-18.5732675	-4.21516	1.33E-11	2.54E-10
MPIG6B	113243758	-18.5929045	-4.21668	4.49E-08	4.43E-07
STXBP2	113242928	-18.6582556	-4.22174	1.11E-16	4.96E-15
TREM1	113261313	-18.8649014	-4.23763	2.04E-11	3.77E-10
IGSF6	113267561	-18.9386164	-4.24326	2.49E-14	7.66E-13
BDH1	113253271	-18.9675628	-4.24546	2E-15	7.28E-14
VAV1	113242671	-19.0446821	-4.25132	3.89E-14	1.16E-12
ADGRE3	113247149	-19.0865655	-4.25449	2.19E-09	2.75E-08
OXCT1	113260937	-19.2854628	-4.26944	1.78E-15	6.58E-14
SPI1	113264961	-19.3347906	-4.27313	4E-15	1.39E-13
MZB1	113261808	-19.4198122	-4.27946	2.75E-09	3.36E-08
TMEM273	113243112	-19.4903899	-4.28469	3.57E-13	9.06E-12
KIF15	113256766	-19.5091614	-4.28608	0	0
CSTA	113252227	-19.5429325	-4.28858	6.35E-14	1.83E-12
P2RY13	113254067	-19.5444157	-4.28868	2.49E-09	3.07E-08
LOC113243250	113243250	-19.601702	-4.29291	9.84E-07	7.52E-06
PLK4	113255653	-19.6100926	-4.29352	6.66E-16	2.62E-14
CD37	113243308	-19.632283	-4.29516	2.54E-12	5.53E-11
LOC113265198	113265198	-19.6353075	-4.29538	1.35E-11	2.57E-10
LOC113267721	113267721	-19.6477565	-4.29629	1.89E-15	6.93E-14

BCL2L15	113244403	-19.6614214	-4.2973	5.11E-08	4.98E-07
MCTP2	113263658	-19.6775409	-4.29848	9.33E-11	1.51E-09
LOC113249403	113249403	-19.7931939	-4.30693	2.2E-08	2.27E-07
CDCA3	113257959	-19.7948849	-4.30706	0	0
SYTL1	113268439	-19.8323199	-4.30978	1.47E-09	1.91E-08
FERMT3	113244183	-19.8432331	-4.31058	1.69E-12	3.82E-11
ATP8B4	113269606	-19.8665723	-4.31227	1E-13	2.8E-12
APOBR	113267635	-19.9292744	-4.31682	1.81E-08	1.89E-07
AFF2	113241893	-19.9688945	-4.31968	3.32E-10	4.9E-09
LMNB1	113261670	-20.0481027	-4.32539	3.11E-15	1.1E-13
ACSS1	113247933	-20.0541863	-4.32583	2.22E-13	5.85E-12
SGO2	113250609	-20.129296	-4.33122	1.11E-16	4.96E-15
LOC113268946	113268946	-20.139342	-4.33194	0	0
FANCI	113263684	-20.1431293	-4.33222	1.89E-15	6.93E-14
ITGAL	113267681	-20.1757306	-4.33455	1.39E-10	2.17E-09
RECQL4	113247590	-20.2089209	-4.33692	6.66E-16	2.62E-14
TEDC1	113267146	-20.2373206	-4.33895	1.35E-11	2.57E-10
CD177	113243501	-20.2674079	-4.34109	6.63E-07	5.22E-06
WAS	113265921	-20.2975435	-4.34323	7.75E-13	1.86E-11
AURKB	113271177	-20.3489962	-4.34689	4.01E-14	1.19E-12
LTB4R	113246010	-20.5012756	-4.35764	3.62E-09	4.31E-08
RAB39A	113260103	-20.5826339	-4.36336	8.58E-12	1.7E-10
PLCB2	113242038	-20.5859732	-4.36359	1.42E-10	2.22E-09
CKS2	113270124	-20.8750745	-4.38371	0	0
ZBP1	113258759	-20.8962358	-4.38517	4.44E-16	1.8E-14
CD4	113257962	-20.911203	-4.3862	2.68E-08	2.72E-07
MMS22L	113264807	-20.9433202	-4.38842	5.77E-15	1.97E-13
COL9A2	113268232	-21.0410011	-4.39513	4.41E-09	5.16E-08

IKZF3	113265509	-21.0664394	-4.39687	1.52E-09	1.96E-08
THEMIS2	113268427	-21.1008511	-4.39923	3.11E-12	6.68E-11
ITGA2B	113265351	-21.2066817	-4.40645	1.77E-10	2.72E-09
CUNH19orf38	113243003	-21.2454105	-4.40908	1.17E-09	1.55E-08
SLC6A9	113254771	-21.3029038	-4.41298	6.44E-15	2.17E-13
TFEC	113263173	-21.3492887	-4.41612	2.22E-16	9.61E-15
CMTM5	113246086	-21.4733399	-4.42447	3.54E-08	3.54E-07
NCKAP1L	113257513	-21.5315446	-4.42838	1.54E-14	4.88E-13
SEPT1	113267508	-21.5891688	-4.43224	2.43E-09	3.01E-08
ALOX5AP	113251228	-21.6063404	-4.43338	3.33E-16	1.39E-14
LOC113265816	113265816	-21.6151033	-4.43397	9.66E-10	1.3E-08
NFAM1	113241698	-21.7058387	-4.44001	1.11E-16	4.96E-15
TBXAS1	113264996	-21.9870178	-4.45858	0	0
TRIM59	113254138	-21.9975341	-4.45927	1.11E-16	4.96E-15
ENTPD3	113253806	-22.0311247	-4.46147	1.68E-07	1.48E-06
PAQR8	113261194	-22.0578516	-4.46322	4.69E-12	9.75E-11
DPYD	113254371	-22.090128	-4.46533	0	0
LOC113254439	113254439	-22.0950174	-4.46565	3E-15	1.06E-13
BIN2	113257606	-22.1554982	-4.46959	1.68E-12	3.81E-11
LOC113247268	113247268	-22.1627573	-4.47007	1.25E-09	1.64E-08
MMP25	113245428	-22.1814861	-4.47128	1.89E-11	3.51E-10
RGS18	113268745	-22.3945307	-4.48507	1.02E-09	1.36E-08
TPX2	113258463	-22.5310784	-4.49384	1.22E-15	4.64E-14
UHRF1	113242848	-22.5350661	-4.4941	0	0
LOC113250056	113250056	-22.5718309	-4.49645	1.5E-14	4.75E-13
SMC2	113260354	-22.5752711	-4.49667	0	0
SLC9A3R1	113244663	-22.6003916	-4.49828	1.18E-14	3.81E-13
LOC113265199	113265199	-22.6057921	-4.49862	0	0

CXCR4	113263416	-22.6604289	-4.5021	1.38E-11	2.62E-10
PRKCB	113267584	-22.6691179	-4.50266	1.33E-12	3.06E-11
MCM2	113257273	-22.6978494	-4.50448	0	0
LOC113247343	113247343	-22.7963833	-4.51073	1.32E-08	1.42E-07
OIP5	113242010	-22.8429509	-4.51368	3.1E-14	9.38E-13
CCND3	113261302	-23.1230099	-4.53126	2.5E-13	6.54E-12
HCK	113258471	-23.146918	-4.53275	2.45E-11	4.42E-10
GGT1	113247032	-23.1761585	-4.53457	8.31E-09	9.27E-08
IL1RN	113242510	-23.2155439	-4.53702	4.34E-14	1.28E-12
SPAG5	113269037	-23.216044	-4.53705	0	0
TNFRSF8	113270667	-23.2337602	-4.53815	8.47E-11	1.38E-09
ITGAX	113267724	-23.2483806	-4.53906	6.22E-15	2.1E-13
CLEC12A	113257906	-23.3500128	-4.54535	5.55E-16	2.21E-14
DOCK2	113264075	-23.5076902	-4.55506	4.44E-16	1.8E-14
LCP1	113251344	-23.6281288	-4.56243	3.64E-11	6.34E-10
HMGB2	113269903	-23.7380086	-4.56913	7.11E-11	1.18E-09
MCM6	113263419	-23.7416914	-4.56935	0	0
SLA	113252705	-23.8453927	-4.57564	1.27E-10	1.99E-09
TIMELESS	113256418	-23.8710479	-4.57719	0	0
AP3M2	113245532	-23.9811017	-4.58383	1.97E-12	4.39E-11
ORC1	113254668	-24.1110063	-4.59162	0	0
NUF2	113268684	-24.1399573	-4.59335	0	0
PTPN7	113242538	-24.3492911	-4.60581	1.4E-11	2.64E-10
ITGB2	113250821	-24.5000352	-4.61471	9.9E-12	1.94E-10
PTK2B	113269957	-24.5550965	-4.61795	4.27E-13	1.08E-11
CXCL9	113263257	-24.6529673	-4.62369	1.37E-10	2.14E-09
CA2	113252986	-24.6599652	-4.6241	4.9E-09	5.71E-08
FRRS1	113254364	-24.7100028	-4.62702	1.36E-12	3.12E-11

ADAM8	113252090	-24.8554909	-4.63549	6.64E-14	1.9E-12
ALOX5	113243124	-24.9447391	-4.64066	7.65E-10	1.05E-08
NCF2	113262736	-25.0714408	-4.64797	6.37E-14	1.83E-12
NUSAP1	113242008	-25.3355362	-4.66309	0	0
FYB1	113260918	-25.4023312	-4.66689	5.11E-14	1.5E-12
SNX10	113261796	-25.5357887	-4.67445	1.67E-12	3.8E-11
LOC113258420	113258420	-25.7563933	-4.68686	8.37E-13	2E-11
SAMSN1	113245916	-25.9968612	-4.70027	5.97E-11	1E-09
SLC11A1	113250457	-26.1623958	-4.70942	6.53E-13	1.6E-11
ORC6	113252201	-26.444289	-4.72488	8.55E-14	2.42E-12
EPX	113269172	-26.6645361	-4.73685	1.45E-05	8.81E-05
DIAPH3	113251398	-26.8052927	-4.74445	0	0
CENPU	113262261	-26.9163607	-4.75041	0	0
PLK1	113267582	-27.0421149	-4.75714	0	0
MYO1F	113242896	-27.6326251	-4.7883	1.05E-12	2.47E-11
SLC43A3	113246559	-27.6488742	-4.78915	1.56E-09	2.01E-08
RAD51	113242027	-28.1115442	-4.81309	0	0
NPTX1	113244533	-28.1211955	-4.81359	2.56E-09	3.15E-08
LOC113265231	113265231	-28.1704314	-4.81611	5.65E-12	1.15E-10
RAC2	113241811	-28.2501182	-4.82018	1.3E-10	2.04E-09
JAK3	113256691	-28.2888714	-4.82216	1.3E-11	2.49E-10
FASN	113244480	-28.3570595	-4.82564	5.41E-07	4.33E-06
BRCA2	113251242	-28.8590866	-4.85095	2.22E-16	9.61E-15
AQP9	113269677	-28.8607475	-4.85104	7.18E-11	1.19E-09
CORO1A	113267639	-28.9279025	-4.85439	3.61E-11	6.3E-10
LAIR1	113247419	-29.003596	-4.85816	6.24E-09	7.09E-08
CD33	113247272	-29.0354055	-4.85974	4.7E-13	1.17E-11
S100P	113245673	-29.1538679	-4.86562	2.94E-11	5.24E-10

POLE2	113266191	-29.3503578	-4.87531	2.25E-14	7.04E-13
PADI4	113270443	-29.4068842	-4.87808	1E-08	1.1E-07
TENT5C	113244369	-29.6905637	-4.89193	6.8E-13	1.66E-11
ITGAM	113267723	-29.8508025	-4.8997	1.38E-14	4.4E-13
CHI3L1	113242521	-30.0566485	-4.90961	8.69E-08	8.1E-07
TREML1	113261319	-30.2331463	-4.91806	4.1E-10	5.92E-09
NCF1	113267772	-30.2648017	-4.91957	7.67E-10	1.05E-08
CFP	113265985	-30.2670349	-4.91968	0	0
FANCM	113266177	-30.3040415	-4.92144	1.3E-14	4.18E-13
IPCEF1	113259578	-30.4527641	-4.9285	7.66E-13	1.85E-11
ZFPM1	113252155	-30.526485	-4.93199	3.33E-16	1.39E-14
LOC113261316	113261316	-30.5670877	-4.93391	3.76E-08	3.75E-07
SELL	113262856	-30.6456027	-4.93761	1.94E-10	2.97E-09
ANLN	113262779	-30.792748	-4.94452	0	0
CD300LF	113244664	-30.8148709	-4.94555	4.91E-10	7E-09
ARHGAP15	113263395	-31.021316	-4.95519	1.09E-12	2.54E-11
MTHFD2	113243787	-31.0310216	-4.95564	2.61E-13	6.8E-12
ACACA	113268927	-31.1425129	-4.96081	1.16E-08	1.26E-07
DSN1	113258564	-31.4333459	-4.97422	0	0
PCED1B	113257691	-31.5369741	-4.97897	2.38E-13	6.28E-12
BUB1B	113242041	-31.7820387	-4.99014	0	0
PTTG1	113264047	-31.8873824	-4.99491	0	0
SERPINB10	113253500	-32.1936355	-5.0087	6.89E-11	1.15E-09
KIF4A	113245588	-32.3711249	-5.01664	0	0
CES2	113252340	-32.3739582	-5.01676	6.88E-11	1.15E-09
BPI	113258590	-32.4529092	-5.02028	0	0
LOC113250055	113250055	-32.4562583	-5.02042	0	0
SASS6	113254358	-32.5242309	-5.02344	1.11E-16	4.96E-15

TMCC2	113242491	-32.570261	-5.02548	2.82E-13	7.26E-12
B3GNT5	113254536	-32.6719407	-5.02998	9.93E-14	2.78E-12
ELOVL6	113255564	-32.711297	-5.03172	1.72E-09	2.19E-08
TYMS	113253260	-32.7838125	-5.03491	0	0
LOC113267145	113267145	-32.8339248	-5.03712	1.17E-06	8.81E-06
IKZF1	113257553	-32.8349734	-5.03716	5.55E-16	2.21E-14
IQGAP3	113245228	-33.1159761	-5.04946	0	0
PCK2	113246063	-33.1983905	-5.05304	0	0
IL18R1	113247412	-33.2304484	-5.05443	4.4E-11	7.52E-10
PGLYRP1	113243405	-33.4505867	-5.06396	1.29E-07	1.16E-06
F5	113262860	-33.6074088	-5.07071	5.11E-12	1.05E-10
SLCO4C1	113255033	-33.8262685	-5.08007	2.22E-16	9.61E-15
MCEMP1	113242925	-33.8634609	-5.08166	8.88E-16	3.42E-14
EVI2B	113269008	-34.1650612	-5.09445	5.15E-13	1.28E-11
TAGAP	113259611	-34.3245992	-5.10117	2.52E-13	6.59E-12
NAPSA	113247269	-34.3573139	-5.10255	4.04E-10	5.85E-09
TFR2	113267851	-34.4652813	-5.10707	3.33E-16	1.39E-14
CCNE2	113252919	-35.141901	-5.13512	0	0
CDCA5	113244138	-35.4793627	-5.14891	1.11E-16	4.96E-15
CSF3R	113268281	-35.4930533	-5.14946	2.94E-10	4.37E-09
FBXO5	113259573	-35.5316764	-5.15103	0	0
CST7	113247929	-35.8168579	-5.16257	7.58E-11	1.25E-09
MBNL3	113270963	-36.0514265	-5.17198	0	0
KIF20A	113261782	-36.1622573	-5.17641	0	0
RMI2	113270796	-36.1977395	-5.17783	1.11E-16	4.96E-15
SLC7A5	113252161	-36.2216342	-5.17878	2.36E-14	7.32E-13
PAQR9	113254023	-36.2659648	-5.18054	1.04E-12	2.46E-11
ITGA4	113250699	-36.3276758	-5.183	1.93E-13	5.15E-12

SRGN	113259122	-36.3320078	-5.18317	3.79E-14	1.14E-12
CKAP2	113251289	-36.4210669	-5.1867	0	0
LOC113254033	113254033	-36.9228861	-5.20644	9.21E-15	3.03E-13
MGST1	113257838	-37.2011841	-5.21728	8.23E-13	1.97E-11
TREML2	113261315	-37.2657677	-5.21978	1.11E-09	1.48E-08
CXCR2	113250472	-37.4293	-5.2261	2.41E-12	5.29E-11
CENPN	113252521	-37.6819806	-5.2358	0	0
CDC20	113254788	-38.0963261	-5.25158	0	0
LOC113267312	113267312	-38.2888738	-5.25885	7.31E-13	1.77E-11
LOC113243522	113243522	-38.6092561	-5.27087	8.63E-14	2.43E-12
THRSP	113269363	-38.7528255	-5.27623	4.66E-10	6.67E-09
TMC8	113244577	-38.8666524	-5.28046	1.11E-16	4.96E-15
CENPW	113259421	-39.3039163	-5.2966	0	0
ARHGEF39	113260476	-39.461893	-5.30239	2.33E-15	8.43E-14
CDCA2	113269969	-39.6541803	-5.3094	0	0
USP35	113269368	-39.9864025	-5.32144	2.22E-16	9.61E-15
DGAT2	113269335	-40.3590021	-5.33482	2.29E-10	3.46E-09
PRG2	113246560	-40.9096912	-5.35437	2.06E-06	1.48E-05
FUT1	113243333	-41.0254905	-5.35845	1.47E-09	1.9E-08
LOC113242534	113242534	-41.3204763	-5.36878	0	0
LOC113248477	113248477	-41.3329491	-5.36922	0	0
MARCO	113263449	-41.3377297	-5.36939	6.66E-15	2.24E-13
ICAM3	113242982	-41.5087178	-5.37534	0	0
FOXM1	113258019	-41.7440948	-5.3835	0	0
SEPT12	113270833	-42.3424343	-5.40403	5.55E-16	2.21E-14
SLC7A4	113245910	-43.2059012	-5.43316	2.15E-14	6.76E-13
CCNA2	113255629	-43.8068952	-5.45309	0	0
SPN	113267673	-43.8642173	-5.45497	1.8E-12	4.06E-11

HPSE	113263199	-45.4508416	-5.50624	0	0
LTF	113256793	-45.7429268	-5.51548	3.37E-06	2.34E-05
KNSTRN	113241921	-46.2968306	-5.53284	0	0
CEBPE	113245996	-46.4712993	-5.53827	2.75E-11	4.91E-10
NFE2	113257521	-46.5539686	-5.54083	2.25E-11	4.1E-10
LYZ	113256289	-48.1166916	-5.58847	4.44E-08	4.37E-07
LOC113247060	113247060	-48.2675009	-5.59298	1.04E-07	9.57E-07
PRC1	113263713	-49.721078	-5.63579	0	0
CD5L	113246669	-50.1686342	-5.64871	3.61E-10	5.3E-09
LOC113244977	113244977	-50.3809509	-5.65481	2.55E-14	7.85E-13
ATAD2	113252754	-50.7402035	-5.66506	0	0
TACC3	113245668	-50.7511292	-5.66537	0	0
CCNB1	113255260	-51.3991487	-5.68367	0	0
FADS2	113246983	-51.9925281	-5.70023	4.4E-13	1.1E-11
ASF1B	113247134	-52.0165442	-5.7009	0	0
HMMR	113264055	-53.4146867	-5.73916	0	0
KIFC1	113243806	-53.8346353	-5.75046	0	0
CDH1	113252413	-53.8539076	-5.75098	7E-12	1.41E-10
LOC113252448	113252448	-54.360117	-5.76448	1.02E-13	2.85E-12
S100A9	113245349	-55.1509624	-5.78531	3.13E-07	2.62E-06
MCM4	113268118	-55.7806639	-5.80169	0	0
TCF19	113264340	-55.9649441	-5.80645	0	0
E2F8	113264938	-57.5635205	-5.84708	0	0
LOC113249696	113249696	-58.6291451	-5.87355	7.59E-09	8.49E-08
MYB	113259474	-59.8861956	-5.90415	1.68E-12	3.81E-11
KIF11	113243170	-60.8943564	-5.92824	0	0
TK1	113244571	-61.3837403	-5.93978	0	0
S100A8	113245155	-63.1767502	-5.98132	8.01E-08	7.55E-07

MGARP	113255684	-64.8445768	-6.01891	3E-15	1.06E-13
NAT8L	113245722	-65.2111129	-6.02705	2.59E-12	5.63E-11
CIT	113267030	-65.4290847	-6.03186	0	0
CAMP	113256532	-67.7756383	-6.08269	5.13E-07	4.11E-06
LOC113245348	113245348	-72.4009686	-6.17794	1.21E-08	1.31E-07
TSPAN33	113264015	-72.8878587	-6.18761	0	0
TOP2A	113265488	-73.2017481	-6.19381	0	0
YPEL4	113246391	-75.3361543	-6.23527	0	0
FADS1	113246984	-75.7769705	-6.24369	1.41E-14	4.49E-13
CTSG	113246003	-76.3393821	-6.25436	1.13E-07	1.03E-06
MED12L	113253630	-77.2470156	-6.27141	1.44E-15	5.4E-14
KIF23	113240920	-80.8377638	-6.33696	0	0
CDK1	113259076	-81.6697533	-6.35173	0	0
LOC113249421	113249421	-84.5801539	-6.40225	4.11E-15	1.42E-13
DCK	113263298	-87.7729524	-6.4557	0	0
KEL	113265440	-90.3262792	-6.49707	3.33E-16	1.39E-14
LOC113256587	113256587	-92.2861371	-6.52804	1.78E-15	6.58E-14
CDC6	113265490	-92.836163	-6.53661	0	0
PCLAF	113240850	-96.1159031	-6.5867	0	0
LOC113260887	113260887	-96.6345464	-6.59447	0	0
MPO	113268870	-108.788735	-6.76539	1.98E-08	2.06E-07
KNL1	113241920	-110.769096	-6.79141	0	0
GATA1	113265928	-111.06222	-6.79522	3.33E-16	1.39E-14
ASPM	113242590	-114.63282	-6.84088	0	0
MKI67	113252057	-125.191925	-6.968	0	0
ADD2	113244225	-126.010222	-6.9774	0	0
MYBL2	113258623	-143.839543	-7.16832	0	0
SCD	113251832	-159.618051	-7.31848	4.19E-14	1.24E-12

CENPF	113262693	-160.983094	-7.33077	0	0
LOC113248289	113248289	-206.414349	-7.6894	2.44E-15	8.78E-14
HBA1	113245361	-215.444473	-7.75117	5.22E-15	1.8E-13
LOC113248288	113248288	-218.961435	-7.77453	2E-15	7.28E-14
ALAS2	113248664	-297.396922	-8.21625	0	0
KLF1	113247208	-350.80796	-8.45454	0	0
EPB42	113241972	-402.766637	-8.6538	0	0
HBM	113245364	-449.081581	-8.81083	0	0
SLC4A1	113265358	-588.358209	-9.20055	0	0

Table 2S. Gene set enrichment for Gene Ontology biological processes, Reactome, KEGG, Hallmark and Biocarta gene sets in bone of black bears during hibernation as compared to summer active animals. NES – normalized enrichment score, positive values indicate elevated proportion of overexpressed genes and negative scores indicate excess of under expressed genes. FDR – false discovery rate with cutoff FDR < 0.05.

Gene Set	#genes	NES	FDR
Hallmark			
KRAS SIGNALING DN	97	2.27	0.003
E2F TARGETS	177	-7.12	<0.001
G2M CHECKPOINT	173	-6.38	<0.001
ALLOGRAFT REJECTION	128	-5.34	<0.001
INFLAMMATORY RESPONSE	148	-4.23	<0.001
INTERFERON ALPHA RESPONSE	75	-3.91	<0.001
COMPLEMENT	157	-3.87	<0.001
HEME METABOLISM	163	-3.81	<0.001
IL6 JAK STAT3 SIGNALING	67	-3.49	<0.001
MTORC1 SIGNALING	183	-3.46	<0.001
EPITHELIAL MESENCHYMAL TRANSITION	186	-2.95	<0.001
KRAS SIGNALING UP	158	-2.94	<0.001
CHOLESTEROL HOMEOSTASIS	65	-2.84	<0.001
XENOBIOTIC METABOLISM	136	-2.52	<0.001
IL2 STAT5 SIGNALING	171	-2.41	<0.001
MITOTIC SPINDLE	185	-2.38	0.001
PROTEIN SECRETION	90	-2.27	0.002
ESTROGEN RESPONSE LATE	152	-2.13	0.004
DNA REPAIR	137	-1.89	0.020
ANGIOGENESIS	31	-1.86	0.022
APICAL JUNCTION	165	-1.84	0.023
PI3K AKT MTOR SIGNALING	94	-1.78	0.030
ANDROGEN RESPONSE	89	-1.74	0.035
COAGULATION	97	-1.71	0.038
UNFOLDED PROTEIN RESPONSE	101	-1.66	0.047
KEGG			

PARKINSONS DISEASE	78	6.43	<0.001
OXIDATIVE PHOSPHORYLATION	76	6.20	<0.001
RIBOSOME	84	5.72	<0.001
ALZHEIMERS DISEASE	116	5.09	<0.001
HUNTINGTONS DISEASE	128	4.28	<0.001
CITRATE CYCLE TCA CYCLE	27	3.27	<0.001
CARDIAC MUSCLE CONTRACTION	27	3.18	<0.001
HYPERTROPHIC CARDIOMYOPATHY HCM	61	2.70	<0.001
PROTEASOME	40	2.55	<0.001
DILATED CARDIOMYOPATHY	67	2.53	<0.001
CIRCADIAN RHYTHM MAMMAL	12	2.08	0.018
CELL CYCLE	109	-4.16	<0.001
LYSOSOME	110	-3.74	<0.001
SPLICEOSOME	118	-3.72	<0.001
HEMATOPOIETIC CELL LINEAGE	51	-3.67	<0.001
PRIMARY IMMUNODEFICIENCY	23	-3.44	<0.001
N GLYCAN BIOSYNTHESIS	42	-3.12	<0.001
DNA REPLICATION	35	-3.11	<0.001
COMPLEMENT AND COAGULATION CASCADES	37	-3.04	<0.001
PORPHYRIN AND CHLOROPHYLL METABOLISM	18	-3.00	<0.001
P53 SIGNALING PATHWAY	58	-2.84	<0.001
LEUKOCYTE TRANSENDOTHELIAL MIGRATION	89	-2.69	<0.001
CHEMOKINE SIGNALING PATHWAY	128	-2.59	0.001
AMINO SUGAR AND NUCLEOTIDE SUGAR METABOLISM	38	-2.54	0.001
ANTIGEN PROCESSING AND PRESENTATION	30	-2.53	0.001
JAK STAT SIGNALING PATHWAY	95	-2.53	0.001
NATURAL KILLER CELL MEDIATED CYTOTOXICITY	73	-2.47	0.001
CYTOKINE CYTOKINE RECEPTOR INTERACTION	131	-2.45	0.001
FC GAMMA R MEDIATED PHAGOCYTOSIS	87	-2.43	0.002
SYSTEMIC LUPUS ERYTHEMATOSUS	23	-2.38	0.002
APOPTOSIS	69	-2.36	0.002

SPHINGOLIPID METABOLISM	29	-2.31	0.003
PATHOGENIC ESCHERICHIA COLI INFECTION	40	-2.30	0.003
STEROID BIOSYNTHESIS	14	-2.26	0.004
VIBRIO CHOLERAE INFECTION	42	-2.22	0.006
ASTHMA	6	-2.07	0.016
GLYCOSAMINOGLYCAN DEGRADATION	17	-2.07	0.015
GLYCEROLIPID METABOLISM	39	-2.03	0.018
TOLL LIKE RECEPTOR SIGNALING PATHWAY	75	-2.03	0.018
FC EPSILON RI SIGNALING PATHWAY	59	-2.01	0.019
OTHER GLYCAN DEGRADATION	13	-2.00	0.020
BIOSYNTHESIS OF UNSATURATED FATTY ACIDS	18	-1.95	0.026
PENTOSE PHOSPHATE PATHWAY	22	-1.95	0.025
SMALL CELL LUNG CANCER	77	-1.95	0.025
ABC TRANSPORTERS	31	-1.92	0.028
GRAFT VERSUS HOST DISEASE	5	-1.92	0.029
BASE EXCISION REPAIR	32	-1.91	0.028
B CELL RECEPTOR SIGNALING PATHWAY	67	-1.91	0.028
LEISHMANIA INFECTION	44	-1.91	0.028
CELL ADHESION MOLECULES CAMS	81	-1.85	0.039
NEUROACTIVE LIGAND RECEPTOR INTERACTION	94	-1.83	0.042
ECM RECEPTOR INTERACTION	73	-1.81	0.046
SULFUR METABOLISM	6	-1.79	0.049
Reactome			
TRANSLATION	265	7.86	<0.001
THE CITRIC ACID TCA CYCLE AND RESPIRATORY ELECTRON TRANSPORT	125	7.72	<0.001
RESPIRATORY ELECTRON TRANSPORT ATP SYNTHESIS BY CHEMIOSMOTIC COUPLING AND HEAT PRODUCTION BY UNCOUPLING PROTEINS	82	7.26	<0.001
COMPLEX I BIOGENESIS	49	6.08	<0.001
EUKARYOTIC TRANSLATION INITIATION	113	6.05	<0.001
MITOCHONDRIAL TRANSLATION	93	5.97	<0.001
EUKARYOTIC TRANSLATION ELONGATION	89	5.83	<0.001

RESPONSE OF EIF2AK4 GCN2 TO AMINO ACID DEFICIENCY	95	5.63	<0.001
SRP DEPENDENT COTRANSLATIONAL PROTEIN TARGETING TO MEMBRANE	107	4.92	<0.001
REGULATION OF EXPRESSION OF SLITS AND ROBOS	155	4.85	<0.001
MUSCLE CONTRACTION	137	4.75	<0.001
SELENOAMINO ACID METABOLISM	101	4.74	<0.001
STRIATED MUSCLE CONTRACTION	27	4.65	<0.001
NONSENSE MEDIATED DECAY NMD	111	4.63	<0.001
ACTIVATION OF THE MRNA UPON BINDING OF THE CAP BINDING COMPLEX AND EIFS AND SUBSEQUENT BINDING TO 43S	56	4.52	<0.001
SIGNALING BY ROBO RECEPTORS	196	4.21	<0.001
FORMATION OF ATP BY CHEMIOSMOTIC COUPLING	16	4.18	<0.001
RRNA PROCESSING	194	4.11	<0.001
CRISTAE FORMATION	27	4.05	<0.001
METABOLISM OF AMINO ACIDS AND DERIVATIVES	288	3.98	<0.001
PROTEIN LOCALIZATION	136	3.96	<0.001
CITRIC ACID CYCLE TCA CYCLE	21	3.92	<0.001
METABOLISM OF RNA	627	3.76	<0.001
CELLULAR RESPONSES TO EXTERNAL STIMULI	456	3.71	<0.001
PYRUVATE METABOLISM AND CITRIC ACID TCA CYCLE	47	3.68	<0.001
INFLUENZA INFECTION	142	3.37	<0.001
CARDIAC CONDUCTION	83	3.29	<0.001
PHASE 0 RAPID DEPOLARISATION	25	3.27	<0.001
NEDDYLATION	211	3.26	<0.001
DECTIN 1 MEDIATED NONCANONICAL NF KB SIGNALING	56	3.07	<0.001
MTORC1 MEDIATED SIGNALLING	23	2.93	<0.001
PINK PARKIN MEDIATED MITOPHAGY	20	2.82	<0.001
CELLULAR RESPONSE TO HYPOXIA	70	2.80	<0.001
AUTOPHAGY	126	2.77	<0.001
REGULATION OF PTEN STABILITY AND ACTIVITY	64	2.77	<0.001

MTOR SIGNALLING	39	2.74	<0.001
ANTIGEN PROCESSING UBIQUITINATION PROTEASOME DEGRADATION	274	2.73	<0.001
MYOCLONIC EPILEPSY OF LAFORA	8	2.70	<0.001
DEGRADATION OF AXIN	51	2.68	<0.001
CLEC7A DECTIN 1 SIGNALING	92	2.66	<0.001
ION HOMEOSTASIS	40	2.62	0.001
SELECTIVE AUTOPHAGY	63	2.57	0.001
NERVOUS SYSTEM DEVELOPMENT	479	2.56	0.001
DEGRADATION OF DVL	52	2.54	0.001
GLYOXYLATE METABOLISM AND GLYCINE DEGRADATION	23	2.49	0.002
DEGRADATION OF GLI1 BY THE PROTEASOME	55	2.47	0.002
PHASE 2 PLATEAU PHASE	15	2.43	0.003
NEGATIVE REGULATION OF NOTCH4 SIGNALING	51	2.42	0.003
SIGNALING BY NOTCH4	77	2.40	0.003
DEVELOPMENTAL BIOLOGY	709	2.38	0.004
RRNA MODIFICATION IN THE NUCLEUS AND CYTOSOL	58	2.37	0.004
PTEN REGULATION	132	2.36	0.004
BETA OXIDATION OF HEXANOYL COA TO BUTANOYL COA	5	2.34	0.005
BETA OXIDATION OF OCTANOYL COA TO HEXANOYL COA	5	2.30	0.006
DNA DAMAGE RECOGNITION IN GG NER	37	2.28	0.007
SIGNALING BY RETINOIC ACID	31	2.28	0.007
REGULATION OF RUNX2 EXPRESSION AND ACTIVITY	65	2.27	0.007
NOTCH3 ACTIVATION AND TRANSMISSION OF SIGNAL TO THE NUCLEUS	23	2.25	0.008
TCF DEPENDENT SIGNALING IN RESPONSE TO WNT	146	2.25	0.008
ENERGY DEPENDENT REGULATION OF MTOR BY LKB1 AMPK	28	2.25	0.008
ASYMMETRIC LOCALIZATION OF PCP PROTEINS	60	2.24	0.008
AUF1 HNRNP D0 BINDS AND DESTABILIZES MRNA	50	2.23	0.009

RRNA PROCESSING IN THE MITOCHONDRION	9	2.20	0.010
C TYPE LECTIN RECEPTORS CLRS	109	2.20	0.011
REGULATION OF PYRUVATE DEHYDROGENASE PDH COMPLEX	15	2.19	0.011
DOWNSTREAM SIGNALING EVENTS OF B CELL RECEPTOR BCR	76	2.18	0.012
PROCESSING OF SMDT1	16	2.18	0.012
GLYCOGEN METABOLISM	25	2.17	0.012
RUNX3 REGULATES YAP1 MEDIATED TRANSCRIPTION	7	2.09	0.019
GLYCOGEN SYNTHESIS	14	2.09	0.019
ION TRANSPORT BY P TYPE ATPASES	40	2.08	0.019
STABILIZATION OF P53	52	2.07	0.020
HEDGEHOG ON STATE	78	2.07	0.020
NEURONAL SYSTEM	228	2.06	0.020
HEDGEHOG LIGAND BIOGENESIS	56	2.04	0.023
RECYCLING OF EIF2 GDP	7	2.03	0.025
GLUCONEOGENESIS	29	2.00	0.030
BETA OXIDATION OF DECANOYL COA TO OCTANOYL COA COA	6	2.00	0.030
FCERI MEDIATED NF KB ACTIVATION	76	2.00	0.030
RAS ACTIVATION UPON CA2 INFLUX THROUGH NMDA RECEPTOR	13	2.00	0.030
BUDDING AND MATURATION OF HIV VIRION	26	1.99	0.031
CROSS PRESENTATION OF SOLUBLE EXOGENOUS ANTIGENS ENDOSOMES	44	1.98	0.032
RNA POLYMERASE III TRANSCRIPTION	39	1.98	0.032
DEGRADATION OF BETA CATENIN BY THE DESTRUCTION COMPLEX	78	1.97	0.034
INTRACELLULAR SIGNALING BY SECOND MESSENGERS	261	1.97	0.034
ABC TRANSPORTER DISORDERS	65	1.97	0.034
RA BIOSYNTHESIS PATHWAY	11	1.96	0.035
SIGNALING BY NOTCH1 HD DOMAIN MUTANTS IN CANCER	13	1.95	0.036

UNBLOCKING OF NMDA RECEPTORS GLUTAMATE BINDING AND ACTIVATION	11	1.95	0.037
INTERACTION BETWEEN L1 AND ANKYRINS	18	1.95	0.037
LATE ENDOSOMAL MICROAUTOPHAGY	28	1.94	0.038
MAPK6 MAPK4 SIGNALING	78	1.93	0.040
RNA POLYMERASE III TRANSCRIPTION INITIATION FROM TYPE 3 PROMOTER	26	1.92	0.041
RRNA MODIFICATION IN THE MITOCHONDRION	6	1.91	0.044
INNATE IMMUNE SYSTEM	762	-6.96	<0.001
NEUTROPHIL DEGRANULATION	374	-6.68	<0.001
CELL CYCLE	522	-5.88	<0.001
CELL CYCLE MITOTIC	429	-5.34	<0.001
IMMUNOREGULATORY INTERACTIONS BETWEEN A LYMPHOID AND A NON LYMPHOID CELL	57	-5.23	<0.001
MITOTIC PROMETAPHASE	162	-4.55	<0.001
PROCESSING OF CAPPED INTRON CONTAINING PRE MRNA	230	-4.38	<0.001
HEMOSTASIS	452	-4.32	<0.001
SIGNALING BY RHO GTPASES	326	-4.09	<0.001
RESOLUTION OF SISTER CHROMATID COHESION	92	-4.06	<0.001
RHO GTPASE EFFECTORS	215	-3.92	<0.001
CHROMOSOME MAINTENANCE	64	-3.82	<0.001
MRNA SPLICING	184	-3.70	<0.001
DNA REPAIR	267	-3.60	<0.001
RHO GTPASES ACTIVATE FORMINS	107	-3.57	<0.001
DNA STRAND ELONGATION	29	-3.52	<0.001
DEPOSITION OF NEW CENPA CONTAINING NUCLEOSOMES AT THE CENTROMERE	22	-3.50	<0.001
PLATELET ACTIVATION SIGNALING AND AGGREGATION	211	-3.48	<0.001
TRANSPORT OF MATURE TRANSCRIPT TO CYTOPLASM	76	-3.48	<0.001
G0 AND EARLY G1	24	-3.47	<0.001
SLC TRANSPORTER DISORDERS	60	-3.46	<0.001

G1 S SPECIFIC TRANSCRIPTION	24	-3.38	<0.001
RESOLUTION OF D LOOP STRUCTURES	31	-3.38	<0.001
HDR THROUGH HOMOLOGOUS RECOMBINATION HRR	63	-3.35	<0.001
CELL CYCLE CHECKPOINTS	222	-3.33	<0.001
SLC MEDIATED TRANSMEMBRANE TRANSPORT	147	-3.32	<0.001
INTERACTIONS OF REV WITH HOST CELLULAR PROTEINS	32	-3.30	<0.001
TRANSCRIPTIONAL REGULATION OF GRANULOPOIESIS	29	-3.26	<0.001
RESOLUTION OF D LOOP STRUCTURES THROUGH SYNTHESIS DEPENDENT STRAND ANNEALING SDSA	25	-3.23	<0.001
INTERFERON ALPHA BETA SIGNALING	36	-3.22	<0.001
ACTIVATION OF THE PRE REPLICATIVE COMPLEX	30	-3.17	<0.001
ANTIMICROBIAL PEPTIDES	21	-3.16	<0.001
M PHASE	302	-3.15	<0.001
AURKA ACTIVATION BY TPX2	64	-3.14	<0.001
BINDING AND UPTAKE OF LIGANDS BY SCAVENGER RECEPTORS	32	-3.13	<0.001
NUCLEAR IMPORT OF REV PROTEIN	29	-3.13	<0.001
POLO LIKE KINASE MEDIATED EVENTS	14	-3.11	<0.001
DNA DOUBLE STRAND BREAK REPAIR	124	-3.08	<0.001
MITOTIC SPINDLE CHECKPOINT	89	-3.07	<0.001
SUMOYLATION OF DNA REPLICATION PROTEINS	40	-3.05	<0.001
CYTOKINE SIGNALING IN IMMUNE SYSTEM	597	-3.03	<0.001
HOMOLOGOUS DNA PAIRING AND STRAND EXCHANGE	41	-3.01	<0.001
TRANSPORT OF THE SLBP DEPENDANT MATURE MRNA	31	-3.01	<0.001
POLYMERASE SWITCHING ON THE C STRAND OF THE TELOMERE	17	-3.00	<0.001
MITOTIC G1 PHASE AND G1 S TRANSITION	136	-2.98	<0.001
CLASS A 1 RHODOPSIN LIKE RECEPTORS	112	-2.96	<0.001
LEISHMANIA INFECTION	155	-2.96	<0.001
SUMOYLATION	158	-2.96	<0.001

IRAK4 DEFICIENCY TLR2 4	10	-2.93	<0.001
INTERFERON SIGNALING	127	-2.93	<0.001
TRANSCRIPTION OF E2F TARGETS UNDER NEGATIVE CONTROL BY DREAM COMPLEX	18	-2.93	<0.001
TRANSPORT OF MATURE MRNAS DERIVED FROM INTRONLESS TRANSCRIPTS	38	-2.90	<0.001
CONDENSATION OF PROPHASE CHROMOSOMES	13	-2.87	<0.001
EXPORT OF VIRAL RIBONUCLEOPROTEINS FROM NUCLEUS	27	-2.87	<0.001
HYALURONAN UPTAKE AND DEGRADATION	12	-2.87	<0.001
UNWINDING OF DNA	9	-2.86	<0.001
SIGNALING BY GPCR	410	-2.86	<0.001
SUMOYLATION OF UBIQUITINYLATION PROTEINS	34	-2.85	<0.001
REGULATION OF GLUCOKINASE BY GLUCOKINASE REGULATORY PROTEIN	26	-2.84	<0.001
GPCR LIGAND BINDING	163	-2.84	<0.001
TELOMERE C STRAND LAGGING STRAND SYNTHESIS	28	-2.82	<0.001
CHROMATIN MODIFYING ENZYMES	196	-2.82	<0.001
RNA POLYMERASE II TRANSCRIPTION TERMINATION	65	-2.79	<0.001
LAGGING STRAND SYNTHESIS	20	-2.78	<0.001
INITIAL TRIGGERING OF COMPLEMENT	13	-2.77	<0.001
TP53 REGULATES TRANSCRIPTION OF CELL CYCLE GENES	45	-2.76	<0.001
PD 1 SIGNALING	10	-2.76	<0.001
ADAPTIVE IMMUNE SYSTEM	577	-2.76	<0.001
GENERATION OF SECOND MESSENGER MOLECULES	20	-2.76	<0.001
INTERLEUKIN 10 SIGNALING	24	-2.75	<0.001
HOMOLOGY DIRECTED REPAIR	98	-2.73	<0.001
COMPLEMENT CASCADE	31	-2.72	<0.001
POLYMERASE SWITCHING	14	-2.71	<0.001
ROS AND RNS PRODUCTION IN PHAGOCYTES	25	-2.71	<0.001
PARASITE INFECTION	56	-2.71	<0.001
ASPARAGINE N LINKED GLYCOSYLATION	247	-2.70	<0.001

CELL SURFACE INTERACTIONS AT THE VASCULAR WALL	97	-2.69	<0.001
SUMOYLATION OF SUMOYLATION PROTEINS	30	-2.69	<0.001
TRANSCRIPTION OF E2F TARGETS UNDER NEGATIVE CONTROL BY P107 RBL1 AND P130 RBL2 IN COMPLEX WITH HDAC1	16	-2.69	<0.001
SUMOYLATION OF DNA DAMAGE RESPONSE AND REPAIR PROTEINS	70	-2.68	<0.001
E2F MEDIATED REGULATION OF DNA REPLICATION	21	-2.67	<0.001
NUCLEAR PORE COMPLEX NPC DISASSEMBLY	30	-2.66	<0.001
ACTIVATION OF ATR IN RESPONSE TO REPLICATION STRESS	33	-2.66	<0.001
REGULATION OF TLR BY ENDOGENOUS LIGAND	11	-2.66	<0.001
G2 M DNA DAMAGE CHECKPOINT	58	-2.65	<0.001
REGULATION OF PLK1 ACTIVITY AT G2 M TRANSITION	77	-2.63	<0.001
INTERACTIONS OF VPR WITH HOST CELLULAR PROTEINS	32	-2.63	<0.001
GPVI MEDIATED ACTIVATION CASCADE	33	-2.63	<0.001
PENTOSE PHOSPHATE PATHWAY	13	-2.62	<0.001
S PHASE	146	-2.62	<0.001
TELOMERE MAINTENANCE	43	-2.61	<0.001
NS1 MEDIATED EFFECTS ON HOST PATHWAYS	34	-2.61	<0.001
INTERLEUKIN 4 AND INTERLEUKIN 13 SIGNALING	78	-2.58	0.001
TRANSPORT OF SMALL MOLECULES	487	-2.57	0.001
BIOSYNTHESIS OF THE N GLYCAN PRECURSOR DOLICHOL LIPID LINKED OLIGOSACCHARIDE LLO AND TRANSFER TO A NASCENT PROTEIN	67	-2.56	0.001
TRANSPORT OF BILE SALTS AND ORGANIC ACIDS METAL IONS AND AMINE COMPOUNDS	39	-2.55	0.001
SIGNAL REGULATORY PROTEIN FAMILY INTERACTIONS	11	-2.55	0.001
RESPONSE TO ELEVATED PLATELET CYTOSOLIC CA2	103	-2.55	0.001
EXTENSION OF TELOMERES	43	-2.55	0.001
METABOLISM OF PORPHYRINS	19	-2.54	0.001

TP53 REGULATES TRANSCRIPTION OF GENES INVOLVED IN G2 CELL CYCLE ARREST	17	-2.54	0.001
ANCHORING OF THE BASAL BODY TO THE PLASMA MEMBRANE	88	-2.54	0.001
SPHINGOLIPID METABOLISM	69	-2.52	0.001
SIGNALING BY INTERLEUKINS	344	-2.52	0.001
REGULATION OF TP53 ACTIVITY THROUGH PHOSPHORYLATION	86	-2.49	0.001
FCGAMMA RECEPTOR FCGR DEPENDENT PHAGOCYTOSIS	80	-2.49	0.001
POSTMITOTIC NUCLEAR PORE COMPLEX NPC REFORMATION	25	-2.49	0.001
METABOLISM OF CARBOHYDRATES	241	-2.49	0.001
INTERFERON GAMMA SIGNALING	50	-2.48	0.001
FCGR ACTIVATION	9	-2.48	0.001
METABOLISM OF LIPIDS	554	-2.48	0.001
PHOSPHORYLATION OF EMI1	6	-2.48	0.001
HYALURONAN METABOLISM	16	-2.47	0.001
MEIOSIS	42	-2.47	0.001
ANTIGEN ACTIVATES B CELL RECEPTOR BCR LEADING TO GENERATION OF SECOND MESSENGERS	28	-2.45	0.001
DAP12 INTERACTIONS	25	-2.44	0.001
EPIGENETIC REGULATION OF GENE EXPRESSION	85	-2.44	0.002
INTEGRIN CELL SURFACE INTERACTIONS	73	-2.41	0.002
MITOTIC METAPHASE AND ANAPHASE	192	-2.41	0.002
EXTRACELLULAR MATRIX ORGANIZATION	235	-2.41	0.002
TRANSFERRIN ENDOCYTOSIS AND RECYCLING	22	-2.41	0.002
REPRODUCTION	51	-2.40	0.002
CROSSLINKING OF COLLAGEN FIBRILS	17	-2.39	0.002
RNA POLYMERASE II TRANSCRIBES SNRNA GENES	72	-2.38	0.002
CHK1 CHK2 CDS1 MEDIATED INACTIVATION OF CYCLIN B CDK1 COMPLEX	11	-2.38	0.002
FACTORS INVOLVED IN MEGAKARYOCYTE DEVELOPMENT AND PLATELET PRODUCTION	108	-2.37	0.002

NA CL DEPENDENT NEUROTRANSMITTER TRANSPORTERS	5	-2.36	0.003
INHIBITION OF REPLICATION INITIATION OF DAMAGED DNA BY RB1 E2F1	12	-2.36	0.003
CASPASE ACTIVATION VIA DEATH RECEPTORS IN THE PRESENCE OF LIGAND	11	-2.35	0.003
HEME BIOSYNTHESIS	12	-2.35	0.003
GLYCOSPHINGOLIPID METABOLISM	34	-2.34	0.003
DAP12 SIGNALING	24	-2.33	0.003
SEPARATION OF SISTER CHROMATIDS	150	-2.33	0.003
RESOLUTION OF ABASIC SITES AP SITES	38	-2.33	0.003
REGULATION OF TP53 ACTIVITY	149	-2.32	0.003
HIV INFECTION	208	-2.32	0.003
NUCLEAR ENVELOPE BREAKDOWN	47	-2.32	0.003
RECRUITMENT OF NUMA TO MITOTIC CENTROSOMES	75	-2.32	0.003
RECRUITMENT OF MITOTIC CENTROSOME PROTEINS AND COMPLEXES	69	-2.32	0.003
INTERLEUKIN 2 SIGNALING	8	-2.31	0.003
TRANS GOLGI NETWORK VESICLE BUDDING	67	-2.31	0.003
MITOTIC TELOPHASE CYTOKINESIS	13	-2.30	0.004
CHOLESTEROL BIOSYNTHESIS	22	-2.29	0.004
RNA POLYMERASE II TRANSCRIPTION	967	-2.27	0.004
DISEASES ASSOCIATED WITH N GLYCOSYLATION OF PROTEINS	16	-2.27	0.004
PROCESSIVE SYNTHESIS ON THE LAGGING STRAND	15	-2.26	0.005
BASE EXCISION REPAIR	44	-2.25	0.005
TRAFFICKING AND PROCESSING OF ENDOSOMAL TLR	12	-2.24	0.005
TRANSCRIPTIONAL REGULATION BY TP53	305	-2.24	0.005
TRANSCRIPTIONAL REGULATION BY SMALL RNAS	41	-2.23	0.006
MITOTIC G2 G2 M PHASES	170	-2.23	0.006
HIV LIFE CYCLE	135	-2.23	0.006
SCAVENGING BY CLASS A RECEPTORS	17	-2.22	0.006

THE ROLE OF NEF IN HIV 1 REPLICATION AND DISEASE PATHOGENESIS	23	-2.21	0.007
HCMV EARLY EVENTS	57	-2.21	0.007
HDR THROUGH SINGLE STRAND ANNEALING SSA	36	-2.21	0.007
DNA REPLICATION	113	-2.20	0.007
FATTY ACYL COA BIOSYNTHESIS	29	-2.20	0.007
MEIOTIC RECOMBINATION	20	-2.18	0.008
SYNTHESIS OF 5 EICOSATETRAENOIC ACIDS	6	-2.18	0.008
CASPASE ACTIVATION VIA EXTRINSIC APOPTOTIC SIGNALLING PATHWAY	20	-2.17	0.008
G ALPHA I SIGNALLING EVENTS	198	-2.17	0.008
ANTI INFLAMMATORY RESPONSE FAVOURING LEISHMANIA PARASITE INFECTION	82	-2.17	0.008
HATS ACETYLATE HISTONES	77	-2.17	0.008
TRIF MEDIATED PROGRAMMED CELL DEATH	8	-2.17	0.008
RHO GTPASE CYCLE	121	-2.16	0.009
PROSTANOID LIGAND RECEPTORS	7	-2.15	0.009
EPHB MEDIATED FORWARD SIGNALING	36	-2.15	0.010
INTERLEUKIN 12 FAMILY SIGNALING	46	-2.14	0.010
CYCLIN A B1 B2 ASSOCIATED EVENTS DURING G2 M TRANSITION	21	-2.14	0.010
G2 M CHECKPOINTS	123	-2.14	0.010
METAL ION SLC TRANSPORTERS	20	-2.14	0.010
SUMOYLATION OF CHROMATIN ORGANIZATION PROTEINS	52	-2.14	0.010
PCNA DEPENDENT LONG PATCH BASE EXCISION REPAIR	21	-2.14	0.010
RESOLUTION OF AP SITES VIA THE MULTIPLE NUCLEOTIDE PATCH REPLACEMENT PATHWAY	25	-2.13	0.010
DEGRADATION OF THE EXTRACELLULAR MATRIX	100	-2.13	0.010
PROCESSING OF INTRONLESS PRE MRNAS	19	-2.13	0.011
TRANSCRIPTIONAL REGULATION BY E2F6	31	-2.12	0.011
CONDENSATION OF PROMETAPHASE CHROMOSOMES	9	-2.11	0.012
PLATELET AGGREGATION PLUG FORMATION	30	-2.10	0.012

VESICLE MEDIATED TRANSPORT	574	-2.10	0.012
SUMOYLATION OF RNA BINDING PROTEINS	42	-2.10	0.012
PROCESSIVE SYNTHESIS ON THE C STRAND OF THE TELOMERE	13	-2.09	0.013
COLLAGEN FORMATION	72	-2.08	0.013
STING MEDIATED INDUCTION OF HOST IMMUNE RESPONSES	12	-2.07	0.014
PROCESSING OF DNA DOUBLE STRAND BREAK ENDS	60	-2.07	0.015
SNRNP ASSEMBLY	47	-2.06	0.015
FANCONI ANEMIA PATHWAY	32	-2.06	0.015
REGULATION OF RUNX1 EXPRESSION AND ACTIVITY	16	-2.06	0.016
DISEASES OF IMMUNE SYSTEM	22	-2.06	0.015
CHEMOKINE RECEPTORS BIND CHEMOKINES	19	-2.05	0.016
RECYCLING PATHWAY OF L1	32	-2.04	0.016
GOLGI ASSOCIATED VESICLE BIOGENESIS	53	-2.04	0.016
INTERLEUKIN 2 FAMILY SIGNALING	32	-2.04	0.017
GENE SILENCING BY RNA	58	-2.04	0.016
MITOTIC PROPHASE	75	-2.04	0.017
PROCESSING OF CAPPED INTRONLESS PRE MRNA	28	-2.04	0.017
DNA REPLICATION INITIATION	8	-2.03	0.017
PLASMA LIPOPROTEIN CLEARANCE	27	-2.03	0.018
COLLAGEN BIOSYNTHESIS AND MODIFYING ENZYMES	56	-2.02	0.018
PHOSPHOLIPID METABOLISM	170	-2.02	0.018
O LINKED GLYCOSYLATION OF MUCINS	33	-2.01	0.019
ACTIVATION OF NOXA AND TRANSLOCATION TO MITOCHONDRIA	5	-2.01	0.019
EARLY PHASE OF HIV LIFE CYCLE	13	-2.01	0.019
VIRAL MESSENGER RNA SYNTHESIS	38	-2.00	0.020
NF KB ACTIVATION THROUGH FADD RIP 1 PATHWAY MEDIATED BY CASPASE 8 AND 10	12	-1.99	0.022
ESTABLISHMENT OF SISTER CHROMATID COHESION	10	-1.98	0.023
METABOLISM OF NUCLEOTIDES	82	-1.98	0.022

DECTIN 2 FAMILY	5	-1.98	0.023
CREATION OF C4 AND C2 ACTIVATORS	8	-1.98	0.023
EICOSANOID LIGAND BINDING RECEPTORS	11	-1.97	0.023
IRON UPTAKE AND TRANSPORT	45	-1.97	0.024
PI METABOLISM	77	-1.97	0.024
ANTIGEN PRESENTATION FOLDING ASSEMBLY AND PEPTIDE LOADING OF CLASS I MHC	19	-1.96	0.024
PEPTIDE LIGAND BINDING RECEPTORS	60	-1.96	0.025
CYCLIN D ASSOCIATED EVENTS IN G1	39	-1.95	0.026
REGULATION OF INSULIN LIKE GROWTH FACTOR IGF TRANSPORT AND UPTAKE BY INSULIN LIKE GROWTH FACTOR BINDING PROTEINS IGFBPS	79	-1.92	0.031
INTEGRIN SIGNALING	24	-1.92	0.031
NEF MEDIATES DOWN MODULATION OF CELL SURFACE RECEPTORS BY RECRUITING THEM TO CLATHRIN ADAPTERS	16	-1.92	0.032
SYNTHESIS OF SUBSTRATES IN N GLYCAN BIOSYTHESIS	54	-1.91	0.032
ATTACHMENT OF GPI ANCHOR TO UPAR	7	-1.91	0.033
G ALPHA S SIGNALLING EVENTS	77	-1.91	0.033
COHESIN LOADING ONTO CHROMATIN	10	-1.91	0.033
INSULIN RECEPTOR RECYCLING	18	-1.90	0.033
ASSEMBLY OF COLLAGEN FIBRILS AND OTHER MULTIMERIC STRUCTURES	49	-1.90	0.034
RUNX3 REGULATES IMMUNE RESPONSE AND CELL MIGRATION	6	-1.90	0.034
POSITIVE EPIGENETIC REGULATION OF RRNA EXPRESSION	45	-1.90	0.034
FCERI MEDIATED CA 2 MOBILIZATION	27	-1.90	0.034
G ALPHA 12 13 SIGNALLING EVENTS	63	-1.89	0.035
TERMINATION OF TRANSLESION DNA SYNTHESIS	30	-1.89	0.035
INTERLEUKIN 12 SIGNALING	38	-1.89	0.036
INTERLEUKIN 3 INTERLEUKIN 5 AND GM CSF SIGNALING	39	-1.89	0.036
RHO GTPASES ACTIVATE NADPH OXIDASES	19	-1.89	0.036

ECM PROTEOGLYCANS	64	-1.88	0.036
DUAL INCISION IN TC NER	60	-1.88	0.037
NEGATIVE EPIGENETIC REGULATION OF RRNA EXPRESSION	47	-1.88	0.037
TRANSCRIPTION OF THE HIV GENOME	64	-1.88	0.037
SYNTHESIS OF LIPOXINS LX	5	-1.87	0.037
TP53 REGULATES TRANSCRIPTION OF ADDITIONAL CELL CYCLE GENES WHOSE EXACT ROLE IN THE P53 PATHWAY REMAIN UNCERTAIN	20	-1.87	0.038
METABOLISM OF STEROIDS	101	-1.87	0.038
RUNX1 REGULATES TRANSCRIPTION OF GENES INVOLVED IN DIFFERENTIATION OF MYELOID CELLS	6	-1.87	0.039
HCMV INFECTION	80	-1.86	0.039
ROLE OF LAT2 NTAL LAB ON CALCIUM MOBILIZATION	15	-1.86	0.039
TP53 REGULATES TRANSCRIPTION OF GENES INVOLVED IN G1 CELL CYCLE ARREST	11	-1.86	0.039
SHC RELATED EVENTS TRIGGERED BY IGF1R	8	-1.86	0.039
BASE EXCISION REPAIR AP SITE FORMATION	16	-1.85	0.041
P130CAS LINKAGE TO MAPK SIGNALING FOR INTEGRINS	12	-1.85	0.041
OTHER INTERLEUKIN SIGNALING	18	-1.84	0.043
DNA DAMAGE TELOMERE STRESS INDUCED SENESCENCE	24	-1.83	0.045
ACTIVATION OF GENE EXPRESSION BY SREBF SREBP	40	-1.83	0.047
MEMBRANE TRAFFICKING	544	-1.83	0.046
FCGR3A MEDIATED IL10 SYNTHESIS	30	-1.82	0.047
NEF MEDIATED DOWNREGULATION OF MHC CLASS I COMPLEX CELL SURFACE EXPRESSION	7	-1.82	0.047
ACYL CHAIN REMODELLING OF PI	5	-1.82	0.047
METABOLISM OF ANGIOTENSINOGEN TO ANGIOTENSINS	10	-1.82	0.047
E2F ENABLED INHIBITION OF PRE REPLICATION COMPLEX FORMATION	9	-1.81	0.050
REGULATION OF CHOLESTEROL BIOSYNTHESIS BY SREBP SREBF	53	-1.81	0.050

Biocarta			
AHSP PATHWAY	10	-3.10	<0.001
BARD1 PATHWAY	8	-3.07	<0.001
THELPER PATHWAY	11	-2.98	<0.001
CELLCYCLE PATHWAY	20	-2.94	<0.001
RB PATHWAY	11	-2.93	<0.001
G2 PATHWAY	22	-2.88	<0.001
STATHMIN PATHWAY	18	-2.86	<0.001
CSK PATHWAY	13	-2.83	<0.001
TCRA PATHWAY	8	-2.81	<0.001
CTL PATHWAY	9	-2.80	<0.001
DC PATHWAY	9	-2.78	<0.001
NO2IL12 PATHWAY	10	-2.78	<0.001
TCYTOTOXIC PATHWAY	10	-2.78	<0.001
COMP PATHWAY	13	-2.69	<0.001
CLASSIC PATHWAY	9	-2.69	<0.001
LAIR PATHWAY	10	-2.69	<0.001
TCAPOPTOSIS PATHWAY	6	-2.66	<0.001
MCM PATHWAY	16	-2.64	<0.001
ATRBRCA PATHWAY	22	-2.54	0.001
CDC25 PATHWAY	7	-2.49	0.001
MONOCYTE PATHWAY	10	-2.38	0.003
IL12 PATHWAY	14	-2.37	0.003
GRANULOCYTES PATHWAY	9	-2.32	0.004
G1 PATHWAY	25	-2.26	0.007
RANMS PATHWAY	10	-2.26	0.007
IL17 PATHWAY	9	-2.14	0.015
CHEMICAL PATHWAY	17	-2.11	0.019
EFP PATHWAY	15	-2.09	0.019
EICOSANOID PATHWAY	18	-2.06	0.022
NEUTROPHIL PATHWAY	7	-2.06	0.022
MHC PATHWAY	8	-2.05	0.022

PLK3 PATHWAY	7	-2.05	0.021
SODD PATHWAY	7	-2.03	0.023
HIVNEF PATHWAY	50	-2.03	0.022
ERAD PATHWAY	18	-2.02	0.023
SRCRPTP PATHWAY	10	-1.99	0.027
P53 PATHWAY	15	-1.97	0.029
LYMPHOCYTE PATHWAY	8	-1.96	0.030
FAS PATHWAY	29	-1.95	0.032
BLYMPHOCYTE PATHWAY	7	-1.94	0.033
PML PATHWAY	12	-1.94	0.032
NKCELLS PATHWAY	15	-1.93	0.033
ALTERNATIVE PATHWAY	6	-1.88	0.043
DNAFRAGMENT PATHWAY	9	-1.87	0.043
IL7 PATHWAY	15	-1.86	0.046
NPC PATHWAY	10	-1.85	0.049
TERT PATHWAY	6	-1.84	0.049
IL2RB PATHWAY	32	-1.84	0.048
INTRINSIC PATHWAY	13	-1.83	0.050
Gene Ontology			
CELLULAR RESPIRATION	137	6.39	<0.001
OXIDATIVE PHOSPHORYLATION	93	6.37	<0.001
MUSCLE SYSTEM PROCESS	321	6.07	<0.001
PEPTIDE BIOSYNTHETIC PROCESS	551	6.06	<0.001
RESPIRATORY ELECTRON TRANSPORT CHAIN	74	6.05	<0.001
ENERGY DERIVATION BY OXIDATION OF ORGANIC COMPOUNDS	210	5.99	<0.001
MUSCLE CONTRACTION	247	5.98	<0.001
ATP SYNTHESIS COUPLED ELECTRON TRANSPORT	56	5.96	<0.001
MITOCHONDRIAL TRANSLATIONAL TERMINATION	88	5.90	<0.001
NADH DEHYDROGENASE COMPLEX ASSEMBLY	56	5.88	<0.001
TRANSLATIONAL TERMINATION	101	5.70	<0.001
TRANSLATIONAL ELONGATION	127	5.56	<0.001

PEPTIDE METABOLIC PROCESS	653	5.54	<0.001
MUSCLE CELL DEVELOPMENT	140	5.22	<0.001
MYOFIBRIL ASSEMBLY	53	5.20	<0.001
STRIATED MUSCLE CONTRACTION	120	5.14	<0.001
TRANSLATIONAL INITIATION	174	5.10	<0.001
AMIDE BIOSYNTHETIC PROCESS	658	5.06	<0.001
GENERATION OF PRECURSOR METABOLITES AND ENERGY	404	4.91	<0.001
COTRANSLATIONAL PROTEIN TARGETING TO MEMBRANE	96	4.85	<0.001
ELECTRON TRANSPORT CHAIN	121	4.70	<0.001
SARCOMERE ORGANIZATION	34	4.63	<0.001
MITOCHONDRION ORGANIZATION	447	4.62	<0.001
MUSCLE FILAMENT SLIDING	26	4.62	<0.001
PROTEIN LOCALIZATION TO ENDOPLASMIC RETICULUM	129	4.60	<0.001
ESTABLISHMENT OF PROTEIN LOCALIZATION TO ENDOPLASMIC RETICULUM	108	4.59	<0.001
ACTIN MEDIATED CELL CONTRACTION	84	4.58	<0.001
RNA PROCESSING	822	4.55	<0.001
CELLULAR AMIDE METABOLIC PROCESS	844	4.49	<0.001
ATP METABOLIC PROCESS	217	4.45	<0.001
SKELETAL MUSCLE CONTRACTION	31	4.43	<0.001
MUSCLE STRUCTURE DEVELOPMENT	510	4.35	<0.001
RIBOSOME BIOGENESIS	276	4.26	<0.001
RIBONUCLEOPROTEIN COMPLEX BIOGENESIS	396	4.24	<0.001
STRIATED MUSCLE CELL DIFFERENTIATION	214	4.24	<0.001
HEART PROCESS	190	4.24	<0.001
MUSCLE CELL DIFFERENTIATION	284	4.15	<0.001
ACTIN FILAMENT BASED MOVEMENT	104	4.11	<0.001
CYTOPLASMIC TRANSLATION	85	4.08	<0.001
CELLULAR COMPONENT ASSEMBLY INVOLVED IN MORPHOGENESIS	85	4.08	<0.001
OXIDATION REDUCTION PROCESS	706	4.08	<0.001

NUCLEAR TRANSCRIBED MRNA CATABOLIC PROCESS NONSENSE MEDIATED DECAY	117	4.06	<0.001
NCRNA METABOLIC PROCESS	400	3.98	<0.001
PROTEIN TARGETING	375	3.98	<0.001
CRISTAE FORMATION	30	3.95	<0.001
NCRNA PROCESSING	345	3.92	<0.001
ATP SYNTHESIS COUPLED PROTON TRANSPORT	19	3.85	<0.001
CELLULAR PROTEIN COMPLEX DISASSEMBLY	190	3.85	<0.001
MULTICELLULAR ORGANISMAL MOVEMENT	42	3.75	<0.001
CARDIAC MUSCLE CONTRACTION	94	3.74	<0.001
CIRCULATORY SYSTEM PROCESS	358	3.72	<0.001
MUSCLE ORGAN DEVELOPMENT	308	3.71	<0.001
MRNA METABOLIC PROCESS	747	3.70	<0.001
ACTOMYOSIN STRUCTURE ORGANIZATION	163	3.64	<0.001
RRNA METABOLIC PROCESS	209	3.63	<0.001
MUSCLE FIBER DEVELOPMENT	53	3.61	<0.001
MUSCLE TISSUE DEVELOPMENT	308	3.57	<0.001
REGULATION OF BLOOD CIRCULATION	180	3.56	<0.001
MULTICELLULAR ORGANISMAL SIGNALING	121	3.56	<0.001
ESTABLISHMENT OF PROTEIN LOCALIZATION TO MEMBRANE	283	3.53	<0.001
REGULATION OF SYSTEM PROCESS	368	3.45	<0.001
CARDIAC CONDUCTION	92	3.42	<0.001
REGULATION OF MUSCLE SYSTEM PROCESS	170	3.40	<0.001
MONOVALENT INORGANIC CATION TRANSPORT	287	3.38	<0.001
ION TRANSMEMBRANE TRANSPORT	686	3.37	<0.001
REGULATION OF HEART RATE	64	3.37	<0.001
CATION TRANSMEMBRANE TRANSPORT	511	3.36	<0.001
MEMBRANE DEPOLARIZATION DURING ACTION POTENTIAL	24	3.32	<0.001
SKELETAL MUSCLE ADAPTATION	20	3.31	<0.001
SKELETAL MUSCLE ORGAN DEVELOPMENT	129	3.29	<0.001
CATION TRANSPORT	691	3.27	<0.001

REGULATION OF ION TRANSMEMBRANE TRANSPORT	278	3.26	<0.001
PROTEIN TARGETING TO MEMBRANE	173	3.26	<0.001
CARDIAC MUSCLE CELL ACTION POTENTIAL	49	3.26	<0.001
REGULATION OF ION TRANSPORT	402	3.24	<0.001
SKELETAL MUSCLE THIN FILAMENT ASSEMBLY	7	3.20	<0.001
REGULATION OF STRIATED MUSCLE CONTRACTION	64	3.18	<0.001
SKELETAL MYOFIBRIL ASSEMBLY	9	3.16	<0.001
SARCOPLASMIC RETICULUM CALCIUM ION TRANSPORT	28	3.14	<0.001
REGULATION OF CATION CHANNEL ACTIVITY	94	3.12	<0.001
CARDIAC MYOFIBRIL ASSEMBLY	19	3.11	<0.001
INORGANIC ION TRANSMEMBRANE TRANSPORT	493	3.10	<0.001
REGULATION OF MUSCLE CONTRACTION	111	3.10	<0.001
ESTABLISHMENT OF PROTEIN LOCALIZATION TO ORGANELLE	482	3.07	<0.001
REGULATION OF MEMBRANE POTENTIAL	252	3.07	<0.001
PROTEIN CONTAINING COMPLEX DISASSEMBLY	284	3.05	<0.001
ACTIN FILAMENT BASED PROCESS	618	3.04	<0.001
REGULATION OF TRANSMEMBRANE TRANSPORT	343	3.04	<0.001
CARDIAC MUSCLE CELL CONTRACTION	50	3.04	<0.001
PROTEIN LOCALIZATION TO MEMBRANE	519	3.02	<0.001
ATP BIOSYNTHETIC PROCESS	38	2.99	<0.001
ACTION POTENTIAL	84	2.98	<0.001
STRIATED MUSCLE MYOSIN THICK FILAMENT ASSEMBLY	6	2.97	<0.001
MODIFICATION DEPENDENT MACROMOLECULE CATABOLIC PROCESS	552	2.95	<0.001
PROTEIN IMPORT INTO MITOCHONDRIAL MATRIX	16	2.93	<0.001
MEMBRANE DEPOLARIZATION DURING CARDIAC MUSCLE CELL ACTION POTENTIAL	17	2.92	<0.001
NUCLEAR TRANSCRIBED MRNA CATABOLIC PROCESS	197	2.91	<0.001
CARDIAC CELL DEVELOPMENT	66	2.90	<0.001

REGULATION OF TRANSPORTER ACTIVITY	170	2.87	<0.001
REGULATION OF CALCIUM ION TRANSMEMBRANE TRANSPORTER ACTIVITY	62	2.87	<0.001
RIBOSOMAL SMALL SUBUNIT BIOGENESIS	64	2.85	<0.001
REGULATION OF CATION TRANSMEMBRANE TRANSPORT	210	2.84	<0.001
RIBOSOME ASSEMBLY	55	2.80	<0.001
STRIATED MUSCLE ADAPTATION	37	2.80	<0.001
PROTEIN MODIFICATION BY SMALL PROTEIN CONJUGATION OR REMOVAL	932	2.78	<0.001
RELEASE OF SEQUESTERED CALCIUM ION INTO CYTOSOL BY ENDOPLASMIC RETICULUM	24	2.77	<0.001
MEMBRANE DEPOLARIZATION	60	2.77	<0.001
KETONE BIOSYNTHETIC PROCESS	33	2.74	<0.001
POSTSYNAPTIC MEMBRANE ORGANIZATION	31	2.74	<0.001
REGULATION OF CARDIAC CONDUCTION	50	2.74	<0.001
REGULATION OF METAL ION TRANSPORT	248	2.70	0.001
HEART MORPHOGENESIS	196	2.68	0.001
CARDIAC MUSCLE FIBER DEVELOPMENT	12	2.68	0.001
PROTEIN TARGETING TO MITOCHONDRION	90	2.67	0.001
PROTEIN POLYUBIQUITINATION	265	2.65	0.001
PROTEIN TRANSMEMBRANE IMPORT INTO INTRACELLULAR ORGANELLE	31	2.63	0.001
CARDIAC MUSCLE CELL ACTION POTENTIAL INVOLVED IN CONTRACTION	36	2.62	0.001
RIBOSOMAL LARGE SUBUNIT BIOGENESIS	65	2.62	0.001
SMOOTH MUSCLE CELL DIFFERENTIATION	52	2.61	0.001
SCF DEPENDENT PROTEASOMAL UBIQUITIN DEPENDENT PROTEIN CATABOLIC PROCESS	83	2.61	0.001
REGULATION OF RELAXATION OF CARDIAC MUSCLE	5	2.60	0.001
REGULATION OF HEART RATE BY CARDIAC CONDUCTION	27	2.60	0.001
MUSCLE ADAPTATION	92	2.59	0.001
REGULATION OF SKELETAL MUSCLE CONTRACTION	9	2.58	0.001
MITOCHONDRIAL TRANSPORT	225	2.58	0.001

CELL COMMUNICATION INVOLVED IN CARDIAC CONDUCTION	39	2.58	0.001
CARDIAC MUSCLE CELL DIFFERENTIATION	93	2.57	0.001
TRNA METABOLIC PROCESS	153	2.57	0.001
RIBONUCLEOSIDE TRIPHOSPHATE BIOSYNTHETIC PROCESS	51	2.57	0.001
CELLULAR KETONE METABOLIC PROCESS	179	2.57	0.001
REGULATION OF SKELETAL MUSCLE ADAPTATION	11	2.56	0.001
FATTY ACID CATABOLIC PROCESS	87	2.56	0.002
POSITIVE REGULATION OF CATION CHANNEL ACTIVITY	41	2.54	0.002
NUCLEOSIDE TRIPHOSPHATE BIOSYNTHETIC PROCESS	61	2.53	0.002
CARDIOCYTE DIFFERENTIATION	116	2.51	0.002
TRANSMEMBRANE TRANSPORT	1000	2.50	0.002
SKELETAL MUSCLE CELL DIFFERENTIATION	49	2.50	0.002
MEMBRANE REPOLARIZATION	32	2.49	0.003
HEART DEVELOPMENT	457	2.48	0.003
CYTOPLASMIC TRANSLATIONAL INITIATION	27	2.47	0.003
T TUBULE ORGANIZATION	6	2.47	0.003
MYOSIN FILAMENT ORGANIZATION	8	2.47	0.003
REGULATION OF CARDIAC MUSCLE CONTRACTION BY CALCIUM ION SIGNALING	21	2.45	0.003
PROTEIN MODIFICATION BY SMALL PROTEIN CONJUGATION	767	2.45	0.004
RIBONUCLEOPROTEIN COMPLEX SUBUNIT ORGANIZATION	178	2.45	0.004
RNA CATABOLIC PROCESS	357	2.44	0.004
REGULATION OF CARDIAC MUSCLE CONTRACTION BY REGULATION OF THE RELEASE OF SEQUESTERED CALCIUM ION	17	2.44	0.004
CELL CELL SIGNALING INVOLVED IN CARDIAC CONDUCTION	22	2.44	0.004
REGULATION OF VOLTAGE GATED CALCIUM CHANNEL ACTIVITY	25	2.43	0.004
RNA MODIFICATION	141	2.43	0.004

RIBOSOMAL SMALL SUBUNIT ASSEMBLY	16	2.43	0.004
NEUROMUSCULAR JUNCTION DEVELOPMENT	40	2.42	0.004
CALCIUM ION TRANSMEMBRANE TRANSPORT	213	2.42	0.004
PROTON TRANSPORTING ATP SYNTHASE COMPLEX ASSEMBLY	7	2.41	0.004
REGULATION OF CALCIUM ION TRANSMEMBRANE TRANSPORT	103	2.41	0.004
POSITIVE REGULATION OF CALCIUM ION TRANSMEMBRANE TRANSPORTER ACTIVITY	32	2.41	0.004
TRANSITION BETWEEN FAST AND SLOW FIBER	6	2.40	0.005
CELLULAR PROTEIN CATABOLIC PROCESS	665	2.40	0.005
NUCLEOTIDE EXCISION REPAIR DNA DAMAGE RECOGNITION	22	2.40	0.005
REGULATION OF ACTIN FILAMENT BASED MOVEMENT	31	2.40	0.005
METAL ION TRANSPORT	543	2.39	0.005
2 OXOGLUTARATE METABOLIC PROCESS	13	2.38	0.005
REGULATION OF CARDIAC MUSCLE CELL ACTION POTENTIAL	21	2.38	0.005
REGULATION OF RELAXATION OF MUSCLE	6	2.36	0.006
VENTRICULAR CARDIAC MUSCLE CELL ACTION POTENTIAL	23	2.36	0.006
DNA TEMPLATED TRANSCRIPTION INITIATION	207	2.36	0.006
MATURATION OF SSU RRNA	45	2.35	0.006
PROTEIN LOCALIZATION TO MITOCHONDRION	125	2.35	0.006
RELAXATION OF CARDIAC MUSCLE	13	2.35	0.006
REGULATION OF CELLULAR PROTEIN CATABOLIC PROCESS	217	2.35	0.007
MESONEPHRIC TUBULE MORPHOGENESIS	43	2.34	0.007
VIRAL GENE EXPRESSION	176	2.34	0.007
VASCULOGENESIS	64	2.33	0.007
ACETYL COA BIOSYNTHETIC PROCESS FROM PYRUVATE	15	2.33	0.007
PHARYNGEAL SYSTEM DEVELOPMENT	18	2.33	0.007
ENDOTHELIUM DEVELOPMENT	100	2.32	0.008

REGULATION OF POTASSIUM ION TRANSMEMBRANE TRANSPORTER ACTIVITY	33	2.31	0.008
CREATINE METABOLIC PROCESS	6	2.31	0.008
MITOCHONDRIAL ACETYL COA BIOSYNTHETIC PROCESS FROM PYRUVATE	6	2.30	0.008
LUNG CELL DIFFERENTIATION	16	2.30	0.009
ISOCITRATE METABOLIC PROCESS	6	2.30	0.009
ESTABLISHMENT OF PROTEIN LOCALIZATION TO MITOCHONDRIAL MEMBRANE	16	2.30	0.009
MITOCHONDRIAL RESPIRATORY CHAIN COMPLEX IV ASSEMBLY	6	2.29	0.009
REGULATION OF CALCIUM ION TRANSPORT	164	2.29	0.009
PROTEASOMAL PROTEIN CATABOLIC PROCESS	422	2.28	0.009
TRNA PROCESSING	117	2.28	0.009
RIBOSE PHOSPHATE BIOSYNTHETIC PROCESS	154	2.28	0.009
CYTOCHROME COMPLEX ASSEMBLY	9	2.28	0.010
REGULATION OF DNA TEMPLATED TRANSCRIPTION IN RESPONSE TO STRESS	110	2.27	0.010
REGULATION OF CARDIAC MUSCLE CONTRACTION	53	2.27	0.010
RENAL TUBULE DEVELOPMENT	62	2.27	0.010
NEGATIVE REGULATION OF CANONICAL WNT SIGNALING PATHWAY	149	2.26	0.011
NADH METABOLIC PROCESS	36	2.26	0.011
RESPIRATORY CHAIN COMPLEX IV ASSEMBLY	6	2.26	0.011
POTASSIUM ION TRANSPORT	122	2.26	0.011
CARDIAC MUSCLE TISSUE DEVELOPMENT	167	2.25	0.011
GLUCOSE METABOLIC PROCESS	172	2.25	0.011
UBIQUINONE METABOLIC PROCESS	18	2.25	0.011
SUCCINATE METABOLIC PROCESS	7	2.25	0.011
POSITIVE REGULATION OF POTASSIUM ION TRANSMEMBRANE TRANSPORTER ACTIVITY	12	2.24	0.012
RESPONSE TO MUSCLE ACTIVITY	18	2.23	0.012
REGULATION OF TRANSCRIPTION FROM RNA POLYMERASE II PROMOTER IN RESPONSE TO HYPOXIA	72	2.23	0.012

LYMPHATIC ENDOTHELIAL CELL DIFFERENTIATION	7	2.23	0.012
CAMP CATABOLIC PROCESS	9	2.22	0.014
LIPID OXIDATION	87	2.20	0.015
CYCLIC NUCLEOTIDE METABOLIC PROCESS	31	2.20	0.015
TRANSMISSION OF NERVE IMPULSE	33	2.19	0.016
RELAXATION OF MUSCLE	22	2.19	0.016
REGULATION OF MUSCLE ADAPTATION	74	2.19	0.017
PROTEIN LOCALIZATION TO ORGANELLE	805	2.18	0.017
PROTEIN INSERTION INTO MITOCHONDRIAL INNER MEMBRANE	10	2.18	0.017
RIBOSOMAL LARGE SUBUNIT ASSEMBLY	26	2.18	0.018
POSITIVE REGULATION OF PROTEOLYSIS INVOLVED IN CELLULAR PROTEIN CATABOLIC PROCESS	110	2.18	0.018
PROTON TRANSMEMBRANE TRANSPORT	93	2.17	0.018
REGULATION OF POTASSIUM ION TRANSPORT	58	2.17	0.018
RIBOSOME DISASSEMBLY	7	2.17	0.018
PURINE CONTAINING COMPOUND BIOSYNTHETIC PROCESS	160	2.17	0.018
VASCULAR SMOOTH MUSCLE CELL DIFFERENTIATION	23	2.17	0.018
REGULATION OF POTASSIUM ION TRANSMEMBRANE TRANSPORT	49	2.16	0.019
FORMATION OF TRANSLATION PREINITIATION COMPLEX	7	2.16	0.019
MITOCHONDRIAL RNA METABOLIC PROCESS	43	2.16	0.019
REGULATION OF CELLULAR KETONE METABOLIC PROCESS	144	2.16	0.019
REGULATION OF MEMBRANE REPOLARIZATION	25	2.16	0.020
POSITIVE REGULATION OF VOLTAGE GATED CALCIUM CHANNEL ACTIVITY	12	2.15	0.020
CARDIAC MUSCLE CELL MEMBRANE REPOLARIZATION	25	2.15	0.020
CELLULAR RESPONSE TO PURINE CONTAINING COMPOUND	13	2.15	0.020
POSITIVE REGULATION OF BIOMINERALIZATION	36	2.14	0.021

REGULATION OF UBIQUITIN DEPENDENT PROTEIN CATABOLIC PROCESS	137	2.14	0.021
POSITIVE REGULATION OF LYASE ACTIVITY	12	2.14	0.021
CALCIUM ION IMPORT INTO SARCOPLASMIC RETICULUM	5	2.14	0.021
NEGATIVE REGULATION OF WNT SIGNALING PATHWAY	174	2.14	0.021
POSITIVE REGULATION OF BONE MINERALIZATION	33	2.13	0.022
REGULATION OF LYASE ACTIVITY	24	2.12	0.023
PROTEIN CATABOLIC PROCESS	785	2.12	0.024
REGULATION OF CYCLASE ACTIVITY	23	2.12	0.024
INCLUSION BODY ASSEMBLY	18	2.11	0.024
CIRCADIAN REGULATION OF GENE EXPRESSION	54	2.10	0.026
FORMATION OF CYTOPLASMIC TRANSLATION INITIATION COMPLEX	14	2.10	0.026
SA NODE CELL TO ATRIAL CARDIAC MUSCLE CELL COMMUNICATION	9	2.10	0.026
LUNG EPITHELIUM DEVELOPMENT	27	2.10	0.026
INTRACELLULAR PROTEIN TRANSPORT	993	2.09	0.029
REGULATION OF MITOCHONDRIAL RNA CATABOLIC PROCESS	5	2.08	0.029
TRNA MODIFICATION	81	2.08	0.029
CAMP METABOLIC PROCESS	18	2.08	0.029
REGULATION OF FATTY ACID BETA OXIDATION	16	2.08	0.029
MYOTUBE DIFFERENTIATION	75	2.07	0.030
CALCIUM MEDIATED SIGNALING	146	2.07	0.030
CELLULAR RESPONSE TO CAFFEINE	10	2.07	0.030
PHARYNGEAL ARCH ARTERY MORPHOGENESIS	6	2.07	0.031
CALCINEURIN MEDIATED SIGNALING	37	2.07	0.031
EAR DEVELOPMENT	143	2.06	0.031
CIRCULATORY SYSTEM DEVELOPMENT	851	2.06	0.032
NEGATIVE REGULATION OF TRANSCRIPTION BY RNA POLYMERASE II	657	2.06	0.032
REGULATION OF CALCIUM MEDIATED SIGNALING	72	2.06	0.032

CELLULAR COMPONENT DISASSEMBLY	462	2.06	0.032
NEUROMUSCULAR SYNAPTIC TRANSMISSION	16	2.06	0.032
VASCULAR SMOOTH MUSCLE CELL DEVELOPMENT	8	2.06	0.033
SODIUM ION TRANSPORT	122	2.05	0.033
POINTED END ACTIN FILAMENT CAPPING	6	2.05	0.033
POSTTRANSCRIPTIONAL REGULATION OF GENE EXPRESSION	494	2.05	0.034
MONOCARBOXYLIC ACID CATABOLIC PROCESS	102	2.05	0.034
RIBONUCLEOSIDE TRIPHOSPHATE METABOLIC PROCESS	65	2.04	0.034
REGULATION OF FATTY ACID OXIDATION	27	2.04	0.034
REGULATION OF MEMBRANE DEPOLARIZATION	35	2.04	0.034
SKELETAL MUSCLE ACETYLCHOLINE GATED CHANNEL CLUSTERING	12	2.04	0.035
NEPHRON MORPHOGENESIS	52	2.04	0.035
POSITIVE REGULATION OF TRANSPORTER ACTIVITY	74	2.03	0.036
LYMPH VESSEL DEVELOPMENT	24	2.03	0.036
ANTERIOR POSTERIOR PATTERN SPECIFICATION	152	2.03	0.036
DIVALENT INORGANIC CATION TRANSPORT	320	2.03	0.037
PROTEIN NEDDYLATION	15	2.03	0.037
POSITIVE REGULATION OF CYCLASE ACTIVITY	12	2.02	0.038
MUSCLE ORGAN MORPHOGENESIS	59	2.02	0.038
POSITIVE REGULATION OF MITOCHONDRIAL TRANSLATION	14	2.02	0.038
ANIMAL ORGAN MORPHOGENESIS	758	2.02	0.039
MUSCLE CELL CELLULAR HOMEOSTASIS	16	2.02	0.039
CARDIAC MUSCLE TISSUE MORPHOGENESIS	46	2.01	0.039
POSITIVE REGULATION OF CELLULAR PROTEIN CATABOLIC PROCESS	128	2.01	0.041
TRANSCRIPTION INITIATION FROM RNA POLYMERASE II PROMOTER	164	2.00	0.042
INOSITOL PHOSPHATE MEDIATED SIGNALING	44	2.00	0.042
LUNG SECRETORY CELL DIFFERENTIATION	5	2.00	0.043
SUPRAMOLECULAR FIBER ORGANIZATION	541	2.00	0.043

REGULATION OF DELAYED RECTIFIER POTASSIUM CHANNEL ACTIVITY	9	2.00	0.043
REGULATION OF WNT SIGNALING PATHWAY	296	2.00	0.043
ARTERY SMOOTH MUSCLE CONTRACTION	8	1.99	0.044
CARDIAC CHAMBER MORPHOGENESIS	102	1.98	0.048
MATURATION OF SSU RRNA FROM TRICISTRONIC RRNA TRANSCRIPT SSU RRNA 5 8S RRNA LSU RRNA	33	1.98	0.048
REGULATION OF TRANSLATIONAL FIDELITY	15	1.98	0.048
RRNA MODIFICATION	33	1.98	0.048
PROTEIN TRANSMEMBRANE TRANSPORT	54	1.97	0.048
RNA METHYLATION	75	1.97	0.048
REGULATION OF BASEMENT MEMBRANE ASSEMBLY INVOLVED IN EMBRYONIC BODY MORPHOGENESIS	5	1.97	0.048
IMMUNE EFFECTOR PROCESS	837	-7.86	<0.001
MYELOID LEUKOCYTE ACTIVATION	500	-7.73	<0.001
CELL ACTIVATION INVOLVED IN IMMUNE RESPONSE	536	-7.30	<0.001
DEFENSE RESPONSE	991	-6.92	<0.001
EXOCYTOSIS	688	-6.47	<0.001
REGULATION OF IMMUNE RESPONSE	676	-6.41	<0.001
RESPONSE TO BIOTIC STIMULUS	918	-6.32	<0.001
DEFENSE RESPONSE TO OTHER ORGANISM	668	-6.17	<0.001
INFLAMMATORY RESPONSE	452	-5.90	<0.001
LYMPHOCYTE ACTIVATION	477	-5.82	<0.001
POSITIVE REGULATION OF IMMUNE SYSTEM PROCESS	746	-5.62	<0.001
REGULATION OF CELL ACTIVATION	394	-5.60	<0.001
CHROMOSOME ORGANIZATION	928	-5.42	<0.001
POSITIVE REGULATION OF IMMUNE RESPONSE	530	-5.33	<0.001
CYTOKINE PRODUCTION	538	-5.15	<0.001
PHAGOCYTOSIS	214	-5.11	<0.001
T CELL ACTIVATION	322	-5.02	<0.001
LEUKOCYTE MIGRATION	291	-4.85	<0.001
LEUKOCYTE PROLIFERATION	192	-4.60	<0.001

DNA DEPENDENT DNA REPLICATION	132	-4.58	<0.001
RESPONSE TO BACTERIUM	352	-4.55	<0.001
REGULATION OF DEFENSE RESPONSE	512	-4.49	<0.001
CHROMATIN ORGANIZATION	581	-4.48	<0.001
LEUKOCYTE DIFFERENTIATION	377	-4.45	<0.001
POSITIVE REGULATION OF CYTOKINE PRODUCTION	311	-4.40	<0.001
TUMOR NECROSIS FACTOR SUPERFAMILY CYTOKINE PRODUCTION	115	-4.35	<0.001
RESPONSE TO CYTOKINE	837	-4.35	<0.001
REGULATION OF LYMPHOCYTE ACTIVATION	304	-4.35	<0.001
LEUKOCYTE CELL CELL ADHESION	240	-4.30	<0.001
DNA REPLICATION	237	-4.28	<0.001
MRNA PROCESSING	452	-4.28	<0.001
DNA REPAIR	471	-4.26	<0.001
NEGATIVE REGULATION OF IMMUNE SYSTEM PROCESS	317	-4.23	<0.001
REGULATION OF IMMUNE EFFECTOR PROCESS	274	-4.20	<0.001
CELL DIVISION	474	-4.18	<0.001
REGULATION OF T CELL ACTIVATION	219	-4.18	<0.001
REGULATION OF LEUKOCYTE PROLIFERATION	146	-4.16	<0.001
B CELL ACTIVATION	179	-4.14	<0.001
REGULATION OF LEUKOCYTE MEDIATED IMMUNITY	135	-4.13	<0.001
DNA METABOLIC PROCESS	740	-4.12	<0.001
POSITIVE REGULATION OF CELL ACTIVATION	234	-4.10	<0.001
CYTOKINE MEDIATED SIGNALING PATHWAY	514	-4.05	<0.001
DNA CONFORMATION CHANGE	202	-4.05	<0.001
MITOTIC CELL CYCLE	835	-3.99	<0.001
MAST CELL ACTIVATION	46	-3.93	<0.001
MYELOID LEUKOCYTE MIGRATION	128	-3.93	<0.001
COAGULATION	234	-3.92	<0.001
IMMUNE SYSTEM DEVELOPMENT	740	-3.92	<0.001
REGULATION OF RESPONSE TO EXTERNAL STIMULUS	743	-3.90	<0.001

MYELOID CELL DIFFERENTIATION	317	-3.90	<0.001
REGULATION OF INFLAMMATORY RESPONSE	229	-3.89	<0.001
INTERLEUKIN 6 PRODUCTION	102	-3.87	<0.001
NEGATIVE REGULATION OF CELL ACTIVATION	138	-3.87	<0.001
DEFENSE RESPONSE TO BACTERIUM	98	-3.87	<0.001
CELLULAR RESPONSE TO DNA DAMAGE STIMULUS	737	-3.85	<0.001
LYMPHOCYTE MEDIATED IMMUNITY	150	-3.83	<0.001
CHROMATIN ASSEMBLY OR DISASSEMBLY	93	-3.81	<0.001
MAST CELL MEDIATED IMMUNITY	39	-3.79	<0.001
RNA SPLICING	391	-3.79	<0.001
IMMUNE RESPONSE REGULATING SIGNALING PATHWAY	402	-3.79	<0.001
CYTOKINE SECRETION	144	-3.76	<0.001
MEIOTIC CELL CYCLE	133	-3.75	<0.001
HUMORAL IMMUNE RESPONSE	98	-3.71	<0.001
REGULATION OF MYELOID LEUKOCYTE MEDIATED IMMUNITY	46	-3.69	<0.001
CELL CYCLE DNA REPLICATION	58	-3.69	<0.001
CARBOHYDRATE DERIVATIVE METABOLIC PROCESS	853	-3.69	<0.001
RNA EXPORT FROM NUCLEUS	124	-3.68	<0.001
LYMPHOCYTE DIFFERENTIATION	254	-3.66	<0.001
REGULATION OF PHAGOCYTOSIS	68	-3.65	<0.001
POSITIVE REGULATION OF DEFENSE RESPONSE	333	-3.64	<0.001
RNA SPLICING VIA TRANSESTERIFICATION REACTIONS	313	-3.62	<0.001
ADAPTIVE IMMUNE RESPONSE BASED ON SOMATIC RECOMBINATION OF IMMUNE RECEPTORS BUILT FROM IMMUNOGLOBULIN SUPERFAMILY DOMAINS	171	-3.62	<0.001
POSITIVE REGULATION OF TUMOR NECROSIS FACTOR SUPERFAMILY CYTOKINE PRODUCTION	66	-3.61	<0.001
REGULATION OF BODY FLUID LEVELS	328	-3.61	<0.001
MRNA EXPORT FROM NUCLEUS	99	-3.59	<0.001
MEIOTIC CELL CYCLE PROCESS	98	-3.59	<0.001

POSITIVE REGULATION OF RESPONSE TO EXTERNAL STIMULUS	427	-3.58	<0.001
GRANULOCYTE MIGRATION	81	-3.57	<0.001
REGULATION OF LEUKOCYTE DEGRANULATION	37	-3.56	<0.001
NEGATIVE REGULATION OF LYMPHOCYTE ACTIVATION	99	-3.55	<0.001
NEGATIVE REGULATION OF CYTOKINE PRODUCTION	194	-3.54	<0.001
POSITIVE REGULATION OF INFLAMMATORY RESPONSE	88	-3.53	<0.001
CHROMOSOME SEGREGATION	244	-3.50	<0.001
CELLULAR DEFENSE RESPONSE	26	-3.50	<0.001
T CELL PROLIFERATION	120	-3.50	<0.001
DNA PACKAGING	101	-3.49	<0.001
REGULATION OF CELL CELL ADHESION	291	-3.48	<0.001
RESPONSE TO MOLECULE OF BACTERIAL ORIGIN	227	-3.48	<0.001
MRNA TRANSPORT	131	-3.48	<0.001
LEUKOCYTE CHEMOTAXIS	132	-3.46	<0.001
NUCLEOSOME ORGANIZATION	85	-3.45	<0.001
INTERFERON GAMMA PRODUCTION	63	-3.44	<0.001
NUCLEOBASE CONTAINING COMPOUND TRANSPORT	207	-3.44	<0.001
CHROMATIN ASSEMBLY	69	-3.43	<0.001
REGULATION OF LEUKOCYTE DIFFERENTIATION	199	-3.42	<0.001
DNA DEPENDENT DNA REPLICATION MAINTENANCE OF FIDELITY	38	-3.41	<0.001
REGULATION OF SECRETION	525	-3.41	<0.001
NEUROINFLAMMATORY RESPONSE	49	-3.41	<0.001
POSITIVE REGULATION OF LEUKOCYTE CELL CELL ADHESION	153	-3.41	<0.001
POSITIVE REGULATION OF IMMUNE EFFECTOR PROCESS	144	-3.40	<0.001
REGULATION OF LEUKOCYTE MIGRATION	141	-3.40	<0.001
DOUBLE STRAND BREAK REPAIR	211	-3.40	<0.001
DNA RECOMBINATION	225	-3.37	<0.001

ION HOMEOSTASIS	528	-3.37	<0.001
POSITIVE REGULATION OF CYTOKINE BIOSYNTHETIC PROCESS	49	-3.37	<0.001
TOLL LIKE RECEPTOR SIGNALING PATHWAY	110	-3.37	<0.001
HOMEOSTASIS OF NUMBER OF CELLS	199	-3.36	<0.001
POSITIVE REGULATION OF LEUKOCYTE PROLIFERATION	92	-3.35	<0.001
ALPHA BETA T CELL ACTIVATION	100	-3.35	<0.001
WOUND HEALING	398	-3.34	<0.001
SIGNAL TRANSDUCTION IN RESPONSE TO DNA DAMAGE	121	-3.34	<0.001
REGULATION OF B CELL ACTIVATION	96	-3.34	<0.001
NEUTROPHIL MIGRATION	65	-3.33	<0.001
ATP DEPENDENT CHROMATIN REMODELING	61	-3.32	<0.001
DNA REPLICATION INDEPENDENT NUCLEOSOME ORGANIZATION	32	-3.31	<0.001
PLATELET ACTIVATION	119	-3.31	<0.001
RNA LOCALIZATION	207	-3.30	<0.001
PROTEIN N LINKED GLYCOSYLATION	67	-3.28	<0.001
B CELL PROLIFERATION	60	-3.27	<0.001
CELL KILLING	76	-3.27	<0.001
ESTABLISHMENT OF RNA LOCALIZATION	174	-3.27	<0.001
CELL CYCLE CHECKPOINT	195	-3.26	<0.001
COVALENT CHROMATIN MODIFICATION	396	-3.26	<0.001
CHEMICAL HOMEOSTASIS	768	-3.26	<0.001
REGULATION OF MULTI ORGANISM PROCESS	533	-3.25	<0.001
INTERLEUKIN 12 PRODUCTION	41	-3.24	<0.001
REGULATION OF GENE EXPRESSION EPIGENETIC	243	-3.24	<0.001
ORGANELLE FISSION	324	-3.24	<0.001
POSITIVE REGULATION OF CELL CELL ADHESION	180	-3.23	<0.001
REGULATION OF ADAPTIVE IMMUNE RESPONSE	114	-3.22	<0.001
CELL CYCLE PHASE TRANSITION	513	-3.22	<0.001
REGULATION OF CYSTEINE TYPE ENDOPEPTIDASE ACTIVITY	167	-3.21	<0.001

MEIOTIC CHROMOSOME SEGREGATION	45	-3.21	<0.001
MITOTIC CELL CYCLE CHECKPOINT	147	-3.21	<0.001
B CELL RECEPTOR SIGNALING PATHWAY	44	-3.21	<0.001
MEMBRANE DOCKING	159	-3.21	<0.001
INTERLEUKIN 8 PRODUCTION	56	-3.20	<0.001
MYELOID LEUKOCYTE DIFFERENTIATION	156	-3.20	<0.001
REGULATION OF PEPTIDASE ACTIVITY	278	-3.19	<0.001
REGULATION OF RESPONSE TO BIOTIC STIMULUS	355	-3.18	<0.001
CHROMATIN REMODELING AT CENTROMERE	26	-3.18	<0.001
IMPORT INTO CELL	522	-3.17	<0.001
MEIOSIS I CELL CYCLE PROCESS	57	-3.17	<0.001
DNA STRAND ELONGATION	21	-3.17	<0.001
PEPTIDE SECRETION	411	-3.15	<0.001
B CELL MEDIATED IMMUNITY	76	-3.14	<0.001
RESPONSE TO DRUG	692	-3.14	<0.001
REGULATION OF HYDROLASE ACTIVITY	916	-3.14	<0.001
REGULATION OF CELL CYCLE PROCESS	614	-3.14	<0.001
POSITIVE REGULATION OF INTERFERON GAMMA PRODUCTION	39	-3.13	<0.001
NUCLEAR EXPORT	179	-3.12	<0.001
PATTERN RECOGNITION RECEPTOR SIGNALING PATHWAY	145	-3.12	<0.001
DNA STRAND ELONGATION INVOLVED IN DNA REPLICATION	15	-3.12	<0.001
REGULATION OF ALPHA BETA T CELL ACTIVATION	70	-3.11	<0.001
CELL CELL ADHESION	553	-3.10	<0.001
NUCLEAR CHROMOSOME SEGREGATION	190	-3.10	<0.001
T CELL DIFFERENTIATION	175	-3.09	<0.001
REGULATION OF CELL ADHESION	519	-3.08	<0.001
METAL ION HOMEOSTASIS	414	-3.08	<0.001
INTERLEUKIN 10 PRODUCTION	37	-3.08	<0.001
REGULATION OF MAST CELL ACTIVATION INVOLVED IN IMMUNE RESPONSE	26	-3.08	<0.001

INTEGRIN MEDIATED SIGNALING PATHWAY	91	-3.08	<0.001
TELOMERE MAINTENANCE VIA SEMI CONSERVATIVE REPLICATION	27	-3.06	<0.001
REGULATION OF MAST CELL ACTIVATION	33	-3.06	<0.001
POSITIVE REGULATION OF CYTOKINE SECRETION	80	-3.06	<0.001
BIOLOGICAL ADHESION	979	-3.06	<0.001
CELL CHEMOTAXIS	187	-3.05	<0.001
CENTROMERE COMPLEX ASSEMBLY	33	-3.04	<0.001
NATURAL KILLER CELL ACTIVATION	43	-3.04	<0.001
RESPONSE TO WOUNDING	480	-3.04	<0.001
POSITIVE REGULATION OF T CELL PROLIFERATION	58	-3.03	<0.001
CYTOKINE METABOLIC PROCESS	82	-3.03	<0.001
POSITIVE REGULATION OF INTERLEUKIN 6 PRODUCTION	61	-3.03	<0.001
DETECTION OF MOLECULE OF BACTERIAL ORIGIN	9	-3.03	<0.001
DETECTION OF EXTERNAL BIOTIC STIMULUS	11	-3.02	<0.001
INTERLEUKIN 1 PRODUCTION	55	-3.02	<0.001
INTERLEUKIN 1 BETA PRODUCTION	50	-3.02	<0.001
POSITIVE REGULATION OF SECRETION	284	-3.02	<0.001
ENDOCYTOSIS	429	-3.01	<0.001
INTERLEUKIN 6 SECRETION	34	-3.01	<0.001
CHROMATIN REMODELING	129	-3.00	<0.001
TRANSITION METAL ION TRANSPORT	87	-3.00	<0.001
RNA 3 END PROCESSING	133	-3.00	<0.001
CELLULAR EXTRAVASATION	56	-3.00	<0.001
MITOTIC NUCLEAR DIVISION	218	-3.00	<0.001
CELL CYCLE G1 S PHASE TRANSITION	226	-2.99	<0.001
SPINDLE ASSEMBLY	94	-2.99	<0.001
DNA REPLICATION INITIATION	32	-2.99	<0.001
CELLULAR ION HOMEOSTASIS	429	-2.99	<0.001
LYMPHOCYTE ACTIVATION INVOLVED IN IMMUNE RESPONSE	123	-2.98	<0.001
REGULATION OF B CELL PROLIFERATION	50	-2.98	<0.001

RECOMBINATIONAL REPAIR	123	-2.96	<0.001
DNA DAMAGE RESPONSE SIGNAL TRANSDUCTION BY P53 CLASS MEDIATOR	97	-2.96	<0.001
REGULATION OF MITOTIC CELL CYCLE	532	-2.95	<0.001
HEME METABOLIC PROCESS	23	-2.95	<0.001
HISTONE EXCHANGE	34	-2.95	<0.001
MRNA 3 END PROCESSING	95	-2.94	<0.001
NEGATIVE REGULATION OF TRANSPORT	335	-2.94	<0.001
NEGATIVE REGULATION OF IMMUNE RESPONSE	99	-2.93	<0.001
POSITIVE REGULATION OF CELL CYCLE PROCESS	216	-2.93	<0.001
REGULATION OF CELL CYCLE	970	-2.93	<0.001
SISTER CHROMATID SEGREGATION	150	-2.92	<0.001
HYALURONAN CATABOLIC PROCESS	16	-2.92	<0.001
DNA INTEGRITY CHECKPOINT	144	-2.91	<0.001
MITOTIC DNA INTEGRITY CHECKPOINT	95	-2.91	<0.001
REGULATION OF MRNA PROCESSING	120	-2.91	<0.001
CELLULAR RESPONSE TO BIOTIC STIMULUS	161	-2.90	<0.001
IMMUNE RESPONSE REGULATING CELL SURFACE RECEPTOR SIGNALING PATHWAY	278	-2.90	<0.001
REPLICATION FORK PROCESSING	30	-2.90	<0.001
RESPIRATORY BURST	21	-2.89	<0.001
REGULATION OF NEUROINFLAMMATORY RESPONSE	21	-2.89	<0.001
G PROTEIN COUPLED PURINERGIC RECEPTOR SIGNALING PATHWAY	17	-2.89	<0.001
POSITIVE REGULATION OF RESPONSE TO BIOTIC STIMULUS	269	-2.89	<0.001
POSITIVE REGULATION OF LEUKOCYTE MEDIATED IMMUNITY	83	-2.89	<0.001
RECEPTOR MEDIATED ENDOCYTOSIS	195	-2.88	<0.001
LEUKOCYTE HOMEOSTASIS	67	-2.88	<0.001
REGULATION OF PEPTIDE SECRETION	328	-2.87	<0.001
PROTOPORPHYRINOGEN IX METABOLIC PROCESS	11	-2.87	<0.001
RESPONSE TO INTERFERON GAMMA	119	-2.87	<0.001
INTERLEUKIN 8 SECRETION	22	-2.86	<0.001

GLIAL CELL ACTIVATION	42	-2.85	<0.001
REGULATION OF CHROMOSOME ORGANIZATION	287	-2.85	<0.001
NCRNA EXPORT FROM NUCLEUS	33	-2.85	<0.001
MACROPHAGE ACTIVATION	69	-2.84	<0.001
GENE SILENCING	187	-2.84	<0.001
MYELOID CELL DEVELOPMENT	60	-2.84	<0.001
PORPHYRIN CONTAINING COMPOUND METABOLIC PROCESS	27	-2.84	<0.001
ALCOHOL BIOSYNTHETIC PROCESS	134	-2.84	<0.001
POSITIVE REGULATION OF HYDROLASE ACTIVITY	565	-2.84	<0.001
RESPONSE TO NITROGEN COMPOUND	810	-2.84	<0.001
MICROTUBULE ORGANIZING CENTER ORGANIZATION	117	-2.83	<0.001
G PROTEIN COUPLED RECEPTOR SIGNALING PATHWAY	466	-2.83	<0.001
LIPID BIOSYNTHETIC PROCESS	528	-2.83	<0.001
RESPONSE TO TYPE I INTERFERON	61	-2.83	<0.001
ACUTE INFLAMMATORY RESPONSE	58	-2.82	<0.001
MYELOID CELL HOMEOSTASIS	128	-2.82	<0.001
RESPONSE TO VIRUS	217	-2.82	<0.001
REGULATION OF CELL CYCLE PHASE TRANSITION	379	-2.81	<0.001
LEUKOCYTE ACTIVATION INVOLVED IN INFLAMMATORY RESPONSE	34	-2.81	<0.001
PLATELET AGGREGATION	48	-2.81	<0.001
COMPLEMENT ACTIVATION	39	-2.81	<0.001
REGULATION OF NEUTROPHIL ACTIVATION	11	-2.80	<0.001
B CELL DIFFERENTIATION	92	-2.80	<0.001
INTERFERON GAMMA MEDIATED SIGNALING PATHWAY	54	-2.80	<0.001
NEGATIVE REGULATION OF SECRETION	143	-2.79	<0.001
RESPONSE TO TOXIC SUBSTANCE	360	-2.79	<0.001
LEUKOCYTE TETHERING OR ROLLING	21	-2.78	<0.001
IRON ION TRANSPORT	55	-2.78	<0.001
GLYCOPROTEIN METABOLIC PROCESS	300	-2.78	<0.001

SPINDLE ORGANIZATION	145	-2.77	<0.001
INTERLEUKIN 6 BIOSYNTHETIC PROCESS	16	-2.77	<0.001
TUMOR NECROSIS FACTOR BIOSYNTHETIC PROCESS	20	-2.76	<0.001
POSITIVE REGULATION OF LEUKOCYTE MIGRATION	97	-2.76	<0.001
MITOTIC SISTER CHROMATID SEGREGATION	126	-2.76	<0.001
REGULATION OF LYMPHOCYTE MEDIATED IMMUNITY	90	-2.76	<0.001
LEUKOCYTE MEDIATED CYTOTOXICITY	55	-2.75	<0.001
REGULATION OF TOLL LIKE RECEPTOR SIGNALING PATHWAY	51	-2.75	<0.001
ALPHA BETA T CELL PROLIFERATION	23	-2.75	<0.001
REGULATION OF TRANSFERASE ACTIVITY	773	-2.75	<0.001
POSITIVE REGULATION OF MULTI ORGANISM PROCESS	386	-2.74	<0.001
TOLL LIKE RECEPTOR 9 SIGNALING PATHWAY	19	-2.74	<0.001
DOUBLE STRAND BREAK REPAIR VIA BREAK INDUCED REPLICATION	9	-2.74	<0.001
CELLULAR HOMEOSTASIS	638	-2.73	<0.001
CELLULAR RESPONSE TO OXYGEN CONTAINING COMPOUND	850	-2.72	<0.001
REGULATION OF LEUKOCYTE APOPTOTIC PROCESS	64	-2.72	<0.001
G1 DNA DAMAGE CHECKPOINT	60	-2.72	<0.001
DNA GEOMETRIC CHANGE	103	-2.72	<0.001
ORGANOPHOSPHATE METABOLIC PROCESS	813	-2.71	<0.001
ADENOSINE RECEPTOR SIGNALING PATHWAY	9	-2.71	<0.001
POSITIVE REGULATION OF TRANSPORT	697	-2.70	<0.001
CENTROSOME DUPLICATION	57	-2.70	<0.001
REGULATION OF ALCOHOL BIOSYNTHETIC PROCESS	60	-2.70	<0.001
NATURAL KILLER CELL MEDIATED IMMUNITY	28	-2.69	<0.001
PROTEIN DNA COMPLEX SUBUNIT ORGANIZATION	176	-2.69	<0.001
FC RECEPTOR MEDIATED STIMULATORY SIGNALING PATHWAY	76	-2.69	<0.001
POSITIVE REGULATION OF PEPTIDE SECRETION	189	-2.69	<0.001

CELLULAR RESPONSE TO AMMONIUM ION	63	-2.69	<0.001
REGULATION OF KINASE ACTIVITY	683	-2.68	<0.001
NUCLEAR TRANSPORT	302	-2.67	<0.001
DETECTION OF BIOTIC STIMULUS	21	-2.67	<0.001
PRODUCTION OF MOLECULAR MEDIATOR OF IMMUNE RESPONSE	143	-2.67	<0.001
POSITIVE REGULATION OF CELL POPULATION PROLIFERATION	637	-2.67	<0.001
PURINERGIC RECEPTOR SIGNALING PATHWAY	21	-2.67	<0.001
NEGATIVE REGULATION OF TUMOR NECROSIS FACTOR SUPERFAMILY CYTOKINE PRODUCTION	40	-2.66	<0.001
GLYCOPROTEIN BIOSYNTHETIC PROCESS	249	-2.66	<0.001
PEPTIDYL LYSINE MODIFICATION	335	-2.65	<0.001
PLATELET MORPHOGENESIS	20	-2.65	<0.001
ORGANELLE LOCALIZATION	527	-2.64	0.001
REGULATION OF HUMORAL IMMUNE RESPONSE	39	-2.64	0.001
TOLL LIKE RECEPTOR 4 SIGNALING PATHWAY	25	-2.64	0.001
CELLULAR LIPID METABOLIC PROCESS	756	-2.63	0.001
POSITIVE REGULATION OF PHAGOCYTOSIS	45	-2.63	0.001
NEGATIVE REGULATION OF CELL CYCLE G1 S PHASE TRANSITION	93	-2.63	0.001
POSITIVE REGULATION OF CYSTEINE TYPE ENDOPEPTIDASE ACTIVITY	105	-2.62	0.001
DEFENSE RESPONSE TO VIRUS	156	-2.61	0.001
POSITIVE REGULATION OF INTERLEUKIN 8 PRODUCTION	34	-2.61	0.001
POSITIVE REGULATION OF ADAPTIVE IMMUNE RESPONSE	73	-2.61	0.001
REGULATION OF VESICLE MEDIATED TRANSPORT	411	-2.61	0.001
POSITIVE REGULATION OF APOPTOTIC CELL CLEARANCE	5	-2.61	0.001
SIGNAL TRANSDUCTION INVOLVED IN CELL CYCLE CHECKPOINT	68	-2.60	0.001
REGULATION OF CELL CYCLE G2 M PHASE TRANSITION	188	-2.60	0.001

RESPONSE TO PEPTIDOGLYCAN	9	-2.60	0.001
SMALL MOLECULE BIOSYNTHETIC PROCESS	540	-2.59	0.001
OLIGOSACCHARIDE LIPID INTERMEDIATE BIOSYNTHETIC PROCESS	18	-2.59	0.001
MICROTUBULE CYTOSKELETON ORGANIZATION	432	-2.58	0.001
MITOTIC SPINDLE ASSEMBLY	50	-2.58	0.001
REGULATION OF TISSUE REMODELING	55	-2.58	0.001
POSITIVE REGULATION OF CELL ADHESION	303	-2.58	0.001
NEGATIVE REGULATION OF TISSUE REMODELING	14	-2.58	0.001
REGULATION OF HEMOPOIESIS	351	-2.58	0.001
PEPTIDYL AMINO ACID MODIFICATION	997	-2.58	0.001
MEMBRANE LIPID CATABOLIC PROCESS	24	-2.58	0.001
POSITIVE REGULATION OF B CELL ACTIVATION	60	-2.57	0.001
POSITIVE REGULATION OF INTERLEUKIN 6 SECRETION	21	-2.57	0.001
POSITIVE REGULATION OF GTPASE ACTIVITY	303	-2.57	0.001
FATTY ACID DERIVATIVE METABOLIC PROCESS	94	-2.57	0.001
REGULATION OF MYELOID LEUKOCYTE DIFFERENTIATION	85	-2.56	0.001
NEGATIVE REGULATION OF LEUKOCYTE MEDIATED IMMUNITY	30	-2.56	0.001
PLATELET DEGRANULATION	98	-2.56	0.001
STEROL BIOSYNTHETIC PROCESS	61	-2.56	0.001
REGULATION OF MRNA SPLICING VIA SPLICEOSOME	84	-2.56	0.001
POSITIVE REGULATION OF INTERLEUKIN 6 BIOSYNTHETIC PROCESS	12	-2.56	0.001
CILIARY BASAL BODY PLASMA MEMBRANE DOCKING	87	-2.55	0.001
POSITIVE REGULATION OF ACUTE INFLAMMATORY RESPONSE	14	-2.55	0.001
PRODUCTION OF MOLECULAR MEDIATOR INVOLVED IN INFLAMMATORY RESPONSE	44	-2.55	0.001
ALCOHOL METABOLIC PROCESS	254	-2.55	0.001
NEGATIVE REGULATION OF ESTABLISHMENT OF PROTEIN LOCALIZATION	131	-2.55	0.001

REGULATION OF PHOSPHOLIPID CATABOLIC PROCESS	5	-2.55	0.001
REGULATION OF NEUTROPHIL DEGRANULATION	8	-2.55	0.001
GRANULOCYTE DIFFERENTIATION	29	-2.55	0.001
NEGATIVE REGULATION OF DEFENSE RESPONSE	144	-2.55	0.001
ACTIVATION OF CYSTEINE TYPE ENDOPEPTIDASE ACTIVITY INVOLVED IN APOPTOTIC PROCESS	63	-2.54	0.001
REGULATION OF PROTEIN SERINE THREONINE KINASE ACTIVITY	409	-2.54	0.001
POSITIVE REGULATION OF TYPE I INTERFERON PRODUCTION	70	-2.54	0.001
CHROMOSOME ORGANIZATION INVOLVED IN MEIOTIC CELL CYCLE	34	-2.54	0.001
POSITIVE REGULATION OF RECEPTOR SIGNALING PATHWAY VIA STAT	50	-2.54	0.001
ERYTHROCYTE HOMEOSTASIS	105	-2.53	0.001
CHEMOKINE PRODUCTION	61	-2.53	0.001
ANTIMICROBIAL HUMORAL RESPONSE	33	-2.53	0.001
FATTY ACID BIOSYNTHETIC PROCESS	106	-2.53	0.001
REACTIVE OXYGEN SPECIES METABOLIC PROCESS	193	-2.53	0.001
REGULATION OF CELL CYCLE G1 S PHASE TRANSITION	142	-2.53	0.001
POSITIVE REGULATION OF CELL CYCLE ARREST	69	-2.52	0.001
PROTOPORPHYRINOGEN IX BIOSYNTHETIC PROCESS	9	-2.52	0.001
LEUKOCYTE APOPTOTIC PROCESS	81	-2.52	0.001
MAINTENANCE OF LOCATION	258	-2.52	0.001
DNA UNWINDING INVOLVED IN DNA REPLICATION	15	-2.52	0.001
MICROTUBULE CYTOSKELETON ORGANIZATION INVOLVED IN MITOSIS	114	-2.51	0.001
TRANSITION METAL ION HOMEOSTASIS	92	-2.51	0.001
MYD88 DEPENDENT TOLL LIKE RECEPTOR SIGNALING PATHWAY	31	-2.51	0.001
MITOTIC SPINDLE ORGANIZATION	91	-2.51	0.001
O GLYCAN PROCESSING	31	-2.50	0.001

RECEPTOR SIGNALING PATHWAY VIA STAT	90	-2.50	0.001
VESICLE ORGANIZATION	285	-2.49	0.001
GENE SILENCING BY RNA	124	-2.49	0.001
TELOMERE ORGANIZATION	138	-2.49	0.001
PLASMA LIPOPROTEIN PARTICLE CLEARANCE	42	-2.49	0.001
TYPE I INTERFERON PRODUCTION	111	-2.49	0.001
POSITIVE REGULATION OF PHOSPHORUS METABOLIC PROCESS	804	-2.49	0.002
HETEROTYPIC CELL CELL ADHESION	46	-2.49	0.002
INTERSPECIES INTERACTION BETWEEN ORGANISMS	754	-2.49	0.002
REGULATION OF DNA DEPENDENT DNA REPLICATION	43	-2.48	0.002
POSITIVE REGULATION OF INTERLEUKIN 12 PRODUCTION	26	-2.48	0.002
LIPID METABOLIC PROCESS	990	-2.48	0.002
REGULATION OF RESPONSE TO DNA DAMAGE STIMULUS	196	-2.48	0.002
REGULATION OF LYMPHOCYTE DIFFERENTIATION	122	-2.48	0.002
POSITIVE REGULATION OF ALPHA BETA T CELL PROLIFERATION	13	-2.47	0.002
HEME BIOSYNTHETIC PROCESS	19	-2.47	0.002
SIGNAL TRANSDUCTION BY P53 CLASS MEDIATOR	240	-2.47	0.002
REGULATION OF MONONUCLEAR CELL MIGRATION	33	-2.47	0.002
CYTOKINE PRODUCTION INVOLVED IN IMMUNE RESPONSE	71	-2.47	0.002
POSITIVE REGULATION OF NF KAPPAB TRANSCRIPTION FACTOR ACTIVITY	121	-2.47	0.002
REGULATION OF GENE SILENCING	94	-2.47	0.002
NEGATIVE REGULATION OF CELL DEATH	712	-2.47	0.002
RESPONSE TO INTERLEUKIN 4	30	-2.47	0.002
CENTRIOLE ASSEMBLY	33	-2.47	0.002
T CELL MIGRATION	46	-2.46	0.002
POSITIVE REGULATION OF HETEROTYPIC CELL CELL ADHESION	9	-2.46	0.002

UNSATURATED FATTY ACID BIOSYNTHETIC PROCESS	38	-2.46	0.002
NEGATIVE REGULATION OF CYTOKINE SECRETION	42	-2.46	0.002
POSITIVE REGULATION OF PEPTIDASE ACTIVITY	140	-2.46	0.002
PRE REPLICATIVE COMPLEX ASSEMBLY INVOLVED IN CELL CYCLE DNA REPLICATION	7	-2.46	0.002
SNRNA PROCESSING	25	-2.46	0.002
POSITIVE REGULATION OF MYELOID LEUKOCYTE MEDIATED IMMUNITY	25	-2.46	0.002
POSITIVE REGULATION OF INTEGRIN ACTIVATION	7	-2.45	0.002
NADPH REGENERATION	16	-2.45	0.002
NEGATIVE REGULATION OF INTERLEUKIN 1 PRODUCTION	17	-2.45	0.002
CARBOHYDRATE DERIVATIVE CATABOLIC PROCESS	142	-2.45	0.002
MONONUCLEAR CELL MIGRATION	48	-2.44	0.002
MICROTUBULE BASED PROCESS	597	-2.44	0.002
PROTEIN CONTAINING COMPLEX LOCALIZATION	243	-2.44	0.002
POSITIVE REGULATION OF LYMPHOCYTE MEDIATED IMMUNITY	60	-2.44	0.002
GLYCOSYLATION	193	-2.44	0.002
REGULATION OF NUCLEOBASE CONTAINING COMPOUND TRANSPORT	16	-2.44	0.002
CHROMOSOME SEPARATION	71	-2.44	0.002
RESPONSE TO FUNGUS	24	-2.43	0.002
LYSOSOME LOCALIZATION	63	-2.43	0.002
CELL PROJECTION ASSEMBLY	443	-2.43	0.002
CD4 POSITIVE ALPHA BETA T CELL ACTIVATION	70	-2.43	0.002
REGULATION OF APOPTOTIC CELL CLEARANCE	7	-2.43	0.002
CELLULAR TRANSITION METAL ION HOMEOSTASIS	74	-2.43	0.002
NEGATIVE REGULATION OF INTERLEUKIN 6 PRODUCTION	29	-2.42	0.002
LOW DENSITY LIPOPROTEIN PARTICLE CLEARANCE	28	-2.42	0.002
ACTIVATION OF INNATE IMMUNE RESPONSE	230	-2.42	0.002
PHAGOSOME ACIDIFICATION	20	-2.42	0.002

REGULATION OF RNA SPLICING	116	-2.42	0.002
ACTIN POLYMERIZATION OR DEPOLYMERIZATION	169	-2.42	0.002
ORGANIC ACID BIOSYNTHETIC PROCESS	233	-2.42	0.002
NEGATIVE REGULATION OF LEUKOCYTE CELL CELL ADHESION	83	-2.42	0.002
CELL CYCLE G2 M PHASE TRANSITION	240	-2.41	0.002
RESPONSE TO PROTOZOAN	11	-2.41	0.002
POSITIVE REGULATION OF TOLL LIKE RECEPTOR SIGNALING PATHWAY	18	-2.41	0.002
POSITIVE REGULATION OF CELL CYCLE	293	-2.41	0.002
ANTIMICROBIAL HUMORAL IMMUNE RESPONSE MEDIATED BY ANTIMICROBIAL PEPTIDE	15	-2.41	0.002
POSITIVE REGULATION OF B CELL MEDIATED IMMUNITY	25	-2.41	0.002
REGULATION OF CHROMATIN ORGANIZATION	148	-2.41	0.003
DETECTION OF OTHER ORGANISM	8	-2.40	0.003
RESPONSE TO LIPID	647	-2.40	0.003
NUCLEIC ACID PHOSPHODIESTER BOND HYDROLYSIS	242	-2.40	0.003
REGULATION OF DNA DIRECTED DNA POLYMERASE ACTIVITY	7	-2.40	0.003
DIVALENT INORGANIC CATION HOMEOSTASIS	316	-2.39	0.003
SUPEROXIDE ANION GENERATION	19	-2.39	0.003
TISSUE REMODELING	127	-2.39	0.003
POSITIVE REGULATION OF HEMOPOIESIS	145	-2.39	0.003
POSITIVE REGULATION OF ALPHA BETA T CELL ACTIVATION	48	-2.39	0.003
REGULATION OF MACROPHAGE ACTIVATION	36	-2.39	0.003
TRIGLYCERIDE METABOLIC PROCESS	65	-2.39	0.003
HUMORAL IMMUNE RESPONSE MEDIATED BY CIRCULATING IMMUNOGLOBULIN	25	-2.39	0.003
DENDRITIC CELL DIFFERENTIATION	29	-2.39	0.003
SIGNAL TRANSDUCTION INVOLVED IN CELLULAR RESPONSE TO AMMONIUM ION	40	-2.38	0.003

REGULATION OF PRODUCTION OF MOLECULAR MEDIATOR OF IMMUNE RESPONSE	101	-2.38	0.003
HISTONE PHOSPHORYLATION	34	-2.38	0.003
REGULATION OF ESTABLISHMENT OF PROTEIN LOCALIZATION	563	-2.38	0.003
ANATOMICAL STRUCTURE HOMEOSTASIS	313	-2.38	0.003
COLLAGEN METABOLIC PROCESS	73	-2.37	0.003
MEGAKARYOCYTE DIFFERENTIATION	62	-2.37	0.003
RESPONSE TO ANTIBIOTIC	232	-2.37	0.003
NEGATIVE REGULATION OF LEUKOCYTE PROLIFERATION	46	-2.37	0.003
POSITIVE REGULATION OF TRANSFERASE ACTIVITY	514	-2.37	0.003
POSITIVE REGULATION OF LEUKOCYTE DEGRANULATION	19	-2.37	0.003
NEGATIVE REGULATION OF PEPTIDE SECRETION	87	-2.37	0.003
RESPONSE TO ALCOHOL	160	-2.37	0.003
REGULATION OF SYMBIOSIS ENCOMPASSING MUTUALISM THROUGH PARASITISM	159	-2.36	0.003
TISSUE HOMEOSTASIS	143	-2.36	0.003
T CELL ACTIVATION INVOLVED IN IMMUNE RESPONSE	65	-2.36	0.003
SYNAPSE PRUNING	11	-2.36	0.003
DOPAMINE RECEPTOR SIGNALING PATHWAY	28	-2.36	0.003
NEGATIVE REGULATION OF MULTICELLULAR ORGANISMAL PROCESS	839	-2.36	0.003
POSITIVE REGULATION OF CHEMOKINE PRODUCTION	41	-2.36	0.003
REGULATION OF INTERLEUKIN 1 SECRETION	27	-2.36	0.003
REGULATION OF LIPID BIOSYNTHETIC PROCESS	151	-2.36	0.003
ENTRY INTO HOST	96	-2.36	0.003
REGULATION OF INTERLEUKIN 4 PRODUCTION	21	-2.36	0.003
REGULATION OF SUPEROXIDE ANION GENERATION	15	-2.35	0.003
REGULATION OF MYELOID CELL DIFFERENTIATION	175	-2.35	0.003
REGULATION OF TOLL LIKE RECEPTOR 2 SIGNALING PATHWAY	7	-2.35	0.004

NUCLEOBASE CONTAINING SMALL MOLECULE METABOLIC PROCESS	488	-2.35	0.004
RESPONSE TO ZINC ION	26	-2.34	0.004
MRNA CLEAVAGE	20	-2.34	0.004
POSITIVE T CELL SELECTION	22	-2.33	0.004
REGULATION OF TOLL LIKE RECEPTOR 4 SIGNALING PATHWAY	13	-2.33	0.004
VIRAL LIFE CYCLE	244	-2.33	0.004
NEGATIVE REGULATION OF CYSTEINE TYPE ENDOPEPTIDASE ACTIVITY	66	-2.33	0.004
INTEGRIN ACTIVATION	21	-2.33	0.004
ALTERNATIVE MRNA SPLICING VIA SPLICEOSOME	67	-2.33	0.004
SEROTONIN TRANSPORT	9	-2.33	0.004
MEGAKARYOCYTE DEVELOPMENT	16	-2.32	0.004
REGULATION OF CHROMOSOME SEGREGATION	83	-2.32	0.004
SISTER CHROMATID COHESION	47	-2.31	0.005
RESPONSE TO NUTRIENT	156	-2.31	0.005
REGULATION OF MRNA METABOLIC PROCESS	280	-2.31	0.005
REGULATION OF PLASMA LIPOPROTEIN PARTICLE LEVELS	66	-2.31	0.005
TOLL LIKE RECEPTOR 2 SIGNALING PATHWAY	12	-2.31	0.005
ALPHA BETA T CELL DIFFERENTIATION	75	-2.31	0.005
PH REDUCTION	41	-2.31	0.005
NEGATIVE REGULATION OF PEPTIDASE ACTIVITY	141	-2.31	0.005
NEGATIVE REGULATION OF CELL CYCLE PROCESS	281	-2.31	0.005
REGULATION OF CELL CYCLE ARREST	90	-2.31	0.005
POSITIVE REGULATION OF TOLL LIKE RECEPTOR 4 SIGNALING PATHWAY	6	-2.30	0.005
NUCLEOTIDE SUGAR METABOLIC PROCESS	34	-2.30	0.005
NEGATIVE REGULATION OF INFLAMMATORY RESPONSE	96	-2.30	0.005
STEROL METABOLIC PROCESS	110	-2.30	0.005
NEGATIVE REGULATION OF CELL CYCLE	513	-2.29	0.005
FATTY ACID DERIVATIVE BIOSYNTHETIC PROCESS	69	-2.29	0.005

DNA REPLICATION SYNTHESIS OF RNA PRIMER	5	-2.29	0.005
REGULATION OF CYTOKINE PRODUCTION INVOLVED IN IMMUNE RESPONSE	59	-2.29	0.005
POSITIVE REGULATION OF INTERLEUKIN 1 SECRETION	18	-2.29	0.005
REGULATION OF SISTER CHROMATID SEGREGATION	68	-2.29	0.005
REGULATION OF TRANSCRIPTION INVOLVED IN G1 S TRANSITION OF MITOTIC CELL CYCLE	20	-2.29	0.005
NEGATIVE REGULATION OF BONE REMODELING	10	-2.29	0.005
OOCYTE MATURATION	16	-2.29	0.005
POSITIVE REGULATION OF PRODUCTION OF MOLECULAR MEDIATOR OF IMMUNE RESPONSE	68	-2.29	0.005
REGULATION OF LYMPHOCYTE APOPTOTIC PROCESS	41	-2.28	0.006
DEFENSE RESPONSE TO GRAM POSITIVE BACTERIUM	34	-2.28	0.006
POSITIVE REGULATION OF I KAPPAB KINASE NF KAPPAB SIGNALING	152	-2.28	0.006
RESPONSE TO FATTY ACID	61	-2.28	0.006
DEFENSE RESPONSE TO FUNGUS	14	-2.28	0.006
PHOSPHOLIPID BIOSYNTHETIC PROCESS	218	-2.28	0.006
NEGATIVE REGULATION OF B CELL ACTIVATION	26	-2.28	0.006
CARBOHYDRATE METABOLIC PROCESS	457	-2.28	0.006
RESPONSE TO INORGANIC SUBSTANCE	394	-2.28	0.006
REGULATION OF DOUBLE STRAND BREAK REPAIR	69	-2.27	0.006
CD4 POSITIVE ALPHA BETA T CELL DIFFERENTIATION	58	-2.27	0.006
CARBOHYDRATE DERIVATIVE BIOSYNTHETIC PROCESS	520	-2.27	0.006
NEGATIVE REGULATION OF CELL CELL ADHESION	127	-2.27	0.006
REGULATION OF T CELL DIFFERENTIATION	98	-2.27	0.006
RESPONSE TO BACTERIAL LIPOPROTEIN	7	-2.27	0.006
POSITIVE REGULATION OF TUMOR NECROSIS FACTOR BIOSYNTHETIC PROCESS	15	-2.27	0.006
GLUCOSE 6 PHOSPHATE METABOLIC PROCESS	20	-2.27	0.006

POSITIVE REGULATION OF INTERFERON GAMMA SECRETION	6	-2.27	0.006
SNRNA TRANSCRIPTION	69	-2.27	0.006
POSITIVE REGULATION OF INTERLEUKIN 8 BIOSYNTHETIC PROCESS	7	-2.26	0.006
REGULATION OF ACUTE INFLAMMATORY RESPONSE	26	-2.26	0.006
TOOTH ERUPTION	5	-2.26	0.006
POSITIVE REGULATION OF MONOCYTE CHEMOTAXIS	11	-2.26	0.006
HOMOLOGOUS RECOMBINATION	32	-2.26	0.006
NEUTRAL LIPID METABOLIC PROCESS	82	-2.26	0.006
NATURAL KILLER CELL ACTIVATION INVOLVED IN IMMUNE RESPONSE	8	-2.26	0.006
MEIOTIC CHROMOSOME CONDENSATION	6	-2.25	0.007
NEGATIVE REGULATION OF MITOTIC CELL CYCLE	274	-2.25	0.007
DNA TEMPLATED TRANSCRIPTION TERMINATION	71	-2.25	0.007
DEFENSE RESPONSE TO GRAM NEGATIVE BACTERIUM	29	-2.25	0.007
MAINTENANCE OF DNA METHYLATION	6	-2.25	0.007
REGULATION OF DNA RECOMBINATION	78	-2.25	0.007
INTERFERON GAMMA SECRETION	11	-2.25	0.007
RESPONSE TO ETHANOL	83	-2.25	0.007
CELLULAR RESPONSE TO DRUG	297	-2.25	0.007
RESPONSE TO RADIATION	327	-2.25	0.007
INTERLEUKIN 4 MEDIATED SIGNALING PATHWAY	7	-2.24	0.007
HYALURONAN METABOLIC PROCESS	31	-2.24	0.007
REGULATION OF CELL KILLING	48	-2.24	0.007
LYMPHOCYTE MIGRATION	64	-2.24	0.007
POSITIVE REGULATION OF PEPTIDYL TYROSINE PHOSPHORYLATION	137	-2.23	0.007
KINETOCHORE ORGANIZATION	14	-2.23	0.008
IRON ION HOMEOSTASIS	63	-2.23	0.008
REGULATION OF ISOMERASE ACTIVITY	7	-2.23	0.008
RESPONSE TO AMMONIUM ION	97	-2.23	0.008

REGULATION OF HETEROTYPIC CELL CELL ADHESION	18	-2.22	0.008
NEGATIVE REGULATION OF GENE EXPRESSION EPIGENETIC	71	-2.22	0.008
EXTRACELLULAR MATRIX DISASSEMBLY	52	-2.22	0.008
MATURE B CELL DIFFERENTIATION INVOLVED IN IMMUNE RESPONSE	16	-2.22	0.008
INTERLEUKIN 4 PRODUCTION	25	-2.22	0.008
MONOCYTE CHEMOTACTIC PROTEIN 1 PRODUCTION	12	-2.22	0.008
REGULATION OF COAGULATION	57	-2.22	0.008
MITOTIC DNA REPLICATION	13	-2.22	0.008
TOLL LIKE RECEPTOR 3 SIGNALING PATHWAY	17	-2.22	0.008
CELLULAR RESPONSE TO NITROGEN COMPOUND	512	-2.21	0.009
POSITIVE REGULATION OF MONONUCLEAR CELL MIGRATION	15	-2.21	0.009
NEGATIVE REGULATION OF MRNA METABOLIC PROCESS	69	-2.21	0.009
CERAMIDE BIOSYNTHETIC PROCESS	47	-2.21	0.009
REGULATION OF CHOLESTEROL METABOLIC PROCESS	48	-2.20	0.009
INTERLEUKIN 1 SECRETION	32	-2.20	0.009
NEGATIVE REGULATION OF LIPOPROTEIN PARTICLE CLEARANCE	6	-2.20	0.009
ACTIN FILAMENT POLYMERIZATION	145	-2.20	0.010
NEGATIVE REGULATION OF TOLL LIKE RECEPTOR SIGNALING PATHWAY	30	-2.19	0.010
REGULATION OF MONOCYTE CHEMOTAXIS	16	-2.19	0.010
REGULATION OF CELLULAR EXTRAVASATION	26	-2.19	0.010
GLYCOLIPID CATABOLIC PROCESS	12	-2.19	0.010
REGULATION OF CENTROSOME DUPLICATION	36	-2.19	0.010
POSITIVE REGULATION OF INTERLEUKIN 2 BIOSYNTHETIC PROCESS	10	-2.19	0.010
REGULATION OF DNA METABOLIC PROCESS	295	-2.19	0.010
CELLULAR RESPONSE TO TOXIC SUBSTANCE	179	-2.18	0.010

REGULATION OF PROTEIN TYROSINE PHOSPHATASE ACTIVITY	6	-2.18	0.010
DETECTION OF STIMULUS	141	-2.18	0.010
PHAGOSOME MATURATION	36	-2.18	0.011
CHROMOSOME CONDENSATION	27	-2.18	0.011
CLATHRIN COAT ASSEMBLY	16	-2.18	0.011
CERAMIDE CATABOLIC PROCESS	14	-2.18	0.011
REGULATION OF INOSITOL PHOSPHATE BIOSYNTHETIC PROCESS	11	-2.18	0.011
RNA PHOSPHODIESTER BOND HYDROLYSIS EXONUCLEOLYTIC	42	-2.18	0.011
TRANSFERRIN TRANSPORT	27	-2.17	0.011
SERINE FAMILY AMINO ACID METABOLIC PROCESS	26	-2.17	0.011
REGULATION OF INTERFERON GAMMA SECRETION	7	-2.17	0.011
POSITIVE REGULATION OF LEUKOCYTE DIFFERENTIATION	114	-2.17	0.011
REGULATION OF REGULATED SECRETORY PATHWAY	108	-2.17	0.011
POSITIVE REGULATION OF INTERLEUKIN 4 PRODUCTION	17	-2.17	0.011
CALMODULIN DEPENDENT KINASE SIGNALING PATHWAY	6	-2.17	0.011
ANION TRANSPORT	390	-2.17	0.011
RESPONSE TO EXTRACELLULAR STIMULUS	378	-2.17	0.011
NEGATIVE REGULATION OF B CELL PROLIFERATION	12	-2.16	0.011
POSITIVE REGULATION OF INTERLEUKIN 8 SECRETION	10	-2.16	0.012
RESPONSE TO METHYLMERCURY	6	-2.16	0.012
LEUKOCYTE ADHESION TO VASCULAR ENDOTHELIAL CELL	31	-2.16	0.012
NEGATIVE REGULATION OF MULTI ORGANISM PROCESS	129	-2.16	0.012
REGULATION OF GTPASE ACTIVITY	366	-2.16	0.012
REGULATION OF ANTIGEN PROCESSING AND PRESENTATION	15	-2.15	0.012
ESTABLISHMENT OF ORGANELLE LOCALIZATION	341	-2.15	0.012

REGULATION OF SIGNAL TRANSDUCTION BY P53 CLASS MEDIATOR	165	-2.15	0.012
RESPONSE TO CHEMOKINE	46	-2.15	0.012
POSITIVE REGULATION OF ERK1 AND ERK2 CASCADE	118	-2.15	0.012
FATTY ACYL COA METABOLIC PROCESS	34	-2.15	0.013
ERK1 AND ERK2 CASCADE	192	-2.15	0.013
NEGATIVE REGULATION OF IMMUNE EFFECTOR PROCESS	84	-2.15	0.013
POSITIVE REGULATION OF VASCULAR ENDOTHELIAL GROWTH FACTOR PRODUCTION	20	-2.14	0.013
LIPOXYGENASE PATHWAY	9	-2.14	0.013
TAXIS	424	-2.14	0.013
DRUG CATABOLIC PROCESS	68	-2.14	0.013
NEGATIVE REGULATION OF PLATELET DERIVED GROWTH FACTOR RECEPTOR SIGNALING PATHWAY	11	-2.14	0.013
MACROPHAGE DIFFERENTIATION	38	-2.14	0.013
CELLULAR RESPONSE TO CISPLATIN	6	-2.14	0.013
NEGATIVE REGULATION OF PHAGOCYTOSIS	18	-2.14	0.013
T CELL SELECTION	31	-2.14	0.013
FATTY ACYL COA BIOSYNTHETIC PROCESS	27	-2.14	0.013
CELLULAR RESPONSE TO FATTY ACID	40	-2.14	0.013
POSITIVE REGULATION OF MONOCYTE CHEMOTACTIC PROTEIN 1 PRODUCTION	8	-2.14	0.013
NEGATIVE REGULATION OF TROPHOBLAST CELL MIGRATION	6	-2.13	0.013
REGULATION OF PLATELET DERIVED GROWTH FACTOR RECEPTOR SIGNALING PATHWAY	20	-2.13	0.013
VASCULAR ENDOTHELIAL GROWTH FACTOR PRODUCTION	25	-2.13	0.013
COLLAGEN BIOSYNTHETIC PROCESS	33	-2.13	0.013
DEOXYRIBONUCLEOTIDE METABOLIC PROCESS	31	-2.13	0.014
REGULATION OF STEROID BIOSYNTHETIC PROCESS	70	-2.13	0.014
AMYLOID BETA CLEARANCE	29	-2.13	0.014
REGULATION OF B CELL DIFFERENTIATION	23	-2.13	0.014

RESPONSE TO VITAMIN E	9	-2.13	0.014
PROTEIN LOCALIZATION TO KINETOCHORE	14	-2.12	0.014
PENTOSE PHOSPHATE SHUNT NON OXIDATIVE BRANCH	5	-2.12	0.014
POSITIVE REGULATION OF B CELL PROLIFERATION	34	-2.12	0.014
REGULATION OF DNA REPLICATION	91	-2.12	0.014
MARGINAL ZONE B CELL DIFFERENTIATION	8	-2.12	0.014
POLYOL METABOLIC PROCESS	94	-2.12	0.014
MUCOPOLYSACCHARIDE METABOLIC PROCESS	93	-2.12	0.014
LIPOSACCHARIDE METABOLIC PROCESS	92	-2.12	0.015
PROTEIN LOCALIZATION TO CHROMOSOME CENTROMERIC REGION	17	-2.11	0.015
GLYCEROLIPID METABOLIC PROCESS	314	-2.11	0.015
LOW DENSITY LIPOPROTEIN RECEPTOR PARTICLE METABOLIC PROCESS	21	-2.11	0.015
AMINOGLYCAN CATABOLIC PROCESS	49	-2.11	0.015
SERINE FAMILY AMINO ACID BIOSYNTHETIC PROCESS	13	-2.11	0.015
DETERMINATION OF ADULT LIFESPAN	13	-2.11	0.015
RESPONSE TO PEPTIDE	394	-2.11	0.015
NEGATIVE REGULATION OF RESPONSE TO EXTERNAL STIMULUS	250	-2.11	0.015
REGULATION OF PROTEIN LOCALIZATION	771	-2.11	0.015
DENDRITIC CELL MIGRATION	19	-2.11	0.015
GROWTH HORMONE RECEPTOR SIGNALING PATHWAY VIA JAK STAT	9	-2.11	0.015
CHROMATIN ORGANIZATION INVOLVED IN REGULATION OF TRANSCRIPTION	67	-2.11	0.015
TERMINATION OF RNA POLYMERASE II TRANSCRIPTION	35	-2.11	0.015
NEGATIVE REGULATION OF CELL ADHESION	208	-2.11	0.016
COPPER ION TRANSMEMBRANE TRANSPORT	6	-2.11	0.016
POSITIVE REGULATION OF KINASE ACTIVITY	449	-2.10	0.016
MONOCYTE CHEMOTAXIS	28	-2.10	0.016

POSITIVE REGULATION OF CELLULAR EXTRAVASATION	16	-2.10	0.016
REGULATION OF RESPONSE TO CYTOKINE STIMULUS	134	-2.10	0.016
POSITIVE REGULATION OF INTERFERON ALPHA PRODUCTION	19	-2.10	0.016
RESPONSE TO MACROPHAGE COLONY STIMULATING FACTOR	10	-2.10	0.016
SNRNA METABOLIC PROCESS	34	-2.10	0.016
STEROID CATABOLIC PROCESS	12	-2.10	0.016
POSITIVE REGULATION OF PROTEIN MODIFICATION PROCESS	900	-2.09	0.016
REGULATION OF DEFENSE RESPONSE TO VIRUS BY VIRUS	26	-2.09	0.016
CYSTEINE METABOLIC PROCESS	9	-2.09	0.016
POSITIVE REGULATION OF INTRACELLULAR SIGNAL TRANSDUCTION	731	-2.09	0.016
MULTICELLULAR ORGANISMAL HOMEOSTASIS	334	-2.09	0.016
RESPONSE TO IRON ION	24	-2.09	0.016
REGULATION OF T CELL MIGRATION	32	-2.09	0.017
REGULATION OF RECEPTOR SIGNALING PATHWAY VIA STAT	75	-2.09	0.017
PROTEIN FOLDING IN ENDOPLASMIC RETICULUM	9	-2.09	0.017
RESPONSE TO IONIZING RADIATION	128	-2.08	0.017
REGULATION OF CHOLESTEROL BIOSYNTHETIC PROCESS	38	-2.08	0.017
NEGATIVE REGULATION OF DNA REPLICATION	30	-2.08	0.018
REGULATION OF CELLULAR RESPONSE TO STRESS	606	-2.08	0.018
REGULATION OF CENTROSOME CYCLE	50	-2.08	0.018
CELLULAR RESPONSE TO IRON ION	8	-2.08	0.018
ORGANIC ANION TRANSPORT	314	-2.08	0.018
CILIUM ORGANIZATION	284	-2.08	0.018
NEUTRAL AMINO ACID TRANSPORT	27	-2.08	0.018
ACTIVATION OF GTPASE ACTIVITY	69	-2.08	0.018
RESPONSE TO METAL ION	249	-2.08	0.018

NEGATIVE REGULATION OF MAST CELL ACTIVATION	10	-2.07	0.018
PIGMENT GRANULE ORGANIZATION	24	-2.07	0.019
REGULATION OF MEGAKARYOCYTE DIFFERENTIATION	45	-2.07	0.019
EXTRACELLULAR STRUCTURE ORGANIZATION	286	-2.07	0.019
POSITIVE REGULATION OF INTERLEUKIN 1 PRODUCTION	29	-2.07	0.019
REGULATION OF LYMPHOCYTE MIGRATION	42	-2.07	0.019
RESPONSE TO MECHANICAL STIMULUS	156	-2.06	0.019
NEGATIVE REGULATION OF CELL CYCLE PHASE TRANSITION	212	-2.06	0.019
APOPTOTIC CELL CLEARANCE	39	-2.06	0.019
SNRNA 3 END PROCESSING	19	-2.06	0.020
MONOVALENT INORGANIC ANION HOMEOSTASIS	14	-2.06	0.020
NEUTROPHIL HOMEOSTASIS	14	-2.06	0.020
POSITIVE REGULATION OF MYELOID CELL DIFFERENTIATION	69	-2.06	0.020
GLYCEROLIPID CATABOLIC PROCESS	40	-2.06	0.020
POSITIVE REGULATION OF CELL DEATH	547	-2.06	0.020
KILLING OF CELLS OF OTHER ORGANISM	19	-2.06	0.020
REGULATION OF DNA REPAIR	109	-2.06	0.020
REGULATION OF POSTTRANSCRIPTIONAL GENE SILENCING	74	-2.06	0.020
CHEMOKINE BIOSYNTHETIC PROCESS	11	-2.06	0.020
INTERACTION WITH SYMBIONT	55	-2.06	0.020
NUCLEOTIDE SUGAR BIOSYNTHETIC PROCESS	21	-2.05	0.020
POSITIVE REGULATION OF CHROMOSOME ORGANIZATION	151	-2.05	0.020
RESPONSE TO LEUKEMIA INHIBITORY FACTOR	82	-2.05	0.020
DEOXYRIBOSE PHOSPHATE CATABOLIC PROCESS	20	-2.05	0.020
TETRAPYRROLE METABOLIC PROCESS	40	-2.05	0.021
T HELPER 1 TYPE IMMUNE RESPONSE	31	-2.05	0.021
NEGATIVE REGULATION OF ALPHA BETA T CELL ACTIVATION	28	-2.05	0.021

REGULATION OF NUCLEAR DIVISION	144	-2.05	0.021
REGULATION OF PEPTIDYL TYROSINE PHOSPHORYLATION	192	-2.05	0.021
TETRAPYRROLE BIOSYNTHETIC PROCESS	25	-2.04	0.022
NEGATIVE REGULATION OF VIRAL PROCESS	56	-2.04	0.022
INTERFERON ALPHA PRODUCTION	23	-2.04	0.022
POSITIVE REGULATION OF LEUKOCYTE CHEMOTAXIS	63	-2.04	0.022
REGULATION OF B CELL MEDIATED IMMUNITY	37	-2.04	0.022
COLLAGEN ACTIVATED SIGNALING PATHWAY	14	-2.04	0.022
GLYCOSYLCERAMIDE CATABOLIC PROCESS	7	-2.04	0.022
L SERINE METABOLIC PROCESS	9	-2.04	0.022
NATURAL KILLER CELL DEGRANULATION	6	-2.04	0.022
POSITIVE REGULATION OF INTERLEUKIN 10 PRODUCTION	23	-2.04	0.022
CELLULAR RESPONSE TO LIPOPROTEIN PARTICLE STIMULUS	26	-2.04	0.022
NEGATIVE REGULATION OF MAST CELL DEGRANULATION	5	-2.03	0.022
MATURE B CELL DIFFERENTIATION	21	-2.03	0.022
LIPOPOLYSACCHARIDE MEDIATED SIGNALING PATHWAY	43	-2.03	0.022
ANTIGEN RECEPTOR MEDIATED SIGNALING PATHWAY	181	-2.03	0.022
POSITIVE REGULATION OF DNA BINDING TRANSCRIPTION FACTOR ACTIVITY	195	-2.03	0.022
REGULATION OF EXTRINSIC APOPTOTIC SIGNALING PATHWAY VIA DEATH DOMAIN RECEPTORS	45	-2.03	0.023
POSITIVE REGULATION OF TYROSINE PHOSPHORYLATION OF STAT PROTEIN	38	-2.03	0.023
TOLERANCE INDUCTION	11	-2.03	0.023
ORGANIC HYDROXY COMPOUND TRANSPORT	137	-2.03	0.023
REGULATION OF CD4 POSITIVE ALPHA BETA T CELL ACTIVATION	46	-2.03	0.023
INTERACTION WITH HOST	158	-2.02	0.024
POSITIVE THYMIC T CELL SELECTION	10	-2.02	0.024

RESPONSE TO HYDROPEROXIDE	17	-2.02	0.024
MRNA CLEAVAGE INVOLVED IN MRNA PROCESSING	12	-2.02	0.024
MYELOID DENDRITIC CELL ACTIVATION	16	-2.02	0.024
NEGATIVE REGULATION OF INTERLEUKIN 12 PRODUCTION	10	-2.02	0.024
MULTI ORGANISM MEMBRANE ORGANIZATION	9	-2.02	0.024
IMMUNOGLOBULIN PRODUCTION	78	-2.02	0.024
G PROTEIN COUPLED ACETYLCHOLINE RECEPTOR SIGNALING PATHWAY	12	-2.02	0.024
HOMOLOGOUS CHROMOSOME SEGREGATION	24	-2.02	0.024
NEGATIVE REGULATION OF SPHINGOLIPID BIOSYNTHETIC PROCESS	5	-2.02	0.024
REGULATION OF GLIAL CELL MIGRATION	13	-2.02	0.024
REGULATION OF ANTIGEN RECEPTOR MEDIATED SIGNALING PATHWAY	45	-2.02	0.024
L AMINO ACID TRANSPORT	46	-2.02	0.024
POSITIVE REGULATION OF MAST CELL ACTIVATION INVOLVED IN IMMUNE RESPONSE	12	-2.02	0.024
GLYCINE TRANSPORT	6	-2.02	0.024
REGULATION OF PLATELET DERIVED GROWTH FACTOR RECEPTOR BETA SIGNALING PATHWAY	7	-2.01	0.024
REGULATION OF GRANULOCYTE DIFFERENTIATION	14	-2.01	0.024
NEUROTRANSMITTER UPTAKE	26	-2.01	0.025
POSITIVE REGULATION OF LYMPHOCYTE DIFFERENTIATION	72	-2.01	0.025
SKIN MORPHOGENESIS	7	-2.01	0.025
REGULATION OF LEUKOCYTE MEDIATED CYTOTOXICITY	37	-2.01	0.025
SEROTONIN UPTAKE	5	-2.01	0.025
SPHINGOID BIOSYNTHETIC PROCESS	9	-2.01	0.025
REGULATION OF SPINDLE ASSEMBLY	24	-2.01	0.025
ADENYLATE CYCLASE ACTIVATING DOPAMINE RECEPTOR SIGNALING PATHWAY	5	-2.01	0.025
POSITIVE REGULATION OF CHROMATIN ORGANIZATION	89	-2.01	0.026

INTERSTRAND CROSS LINK REPAIR	46	-2.01	0.026
REGULATION OF NUCLEOCYTOPLASMIC TRANSPORT	95	-2.01	0.026
PHOSPHATIDYLINOSITOL BIOSYNTHETIC PROCESS	103	-2.00	0.026
PROTEIN ACETYLATION	172	-2.00	0.026
RESPONSE TO ANTINEOPLASTIC AGENT	76	-2.00	0.026
REGULATION OF CARBOHYDRATE METABOLIC PROCESS	171	-2.00	0.026
POSITIVE REGULATION OF CELL CYCLE G2 M PHASE TRANSITION	27	-2.00	0.026
PIGMENT ACCUMULATION	12	-2.00	0.026
MEMBRANE INVAGINATION	61	-2.00	0.026
LEUKOCYTE AGGREGATION	12	-1.99	0.027
UDP N ACETYLGLUCOSAMINE METABOLIC PROCESS	14	-1.99	0.027
METAPHASE ANAPHASE TRANSITION OF CELL CYCLE	47	-1.99	0.027
INTERLEUKIN 2 BIOSYNTHETIC PROCESS	17	-1.99	0.027
CARBOHYDRATE DERIVATIVE TRANSPORT	53	-1.99	0.027
PROTEIN LOCALIZATION TO CHROMOSOME	72	-1.99	0.027
MANGANESE ION TRANSMEMBRANE TRANSPORT	5	-1.99	0.027
PEPTIDYL ASPARAGINE MODIFICATION	29	-1.99	0.027
CERAMIDE METABOLIC PROCESS	67	-1.99	0.027
ACYLGLYCEROL HOMEOSTASIS	21	-1.99	0.027
MEMBRANE LIPID BIOSYNTHETIC PROCESS	112	-1.99	0.028
REGULATION OF PLATELET ACTIVATION	26	-1.99	0.028
UNSATURATED FATTY ACID METABOLIC PROCESS	57	-1.99	0.028
POSITIVE REGULATION OF MAPK CASCADE	369	-1.99	0.028
CLATHRIN DEPENDENT ENDOCYTOSIS	39	-1.99	0.028
POSITIVE REGULATION OF NIK NF KAPPAB SIGNALING	59	-1.99	0.028
NEGATIVE REGULATION OF MRNA PROCESSING	29	-1.99	0.028
REGULATION OF CYCLIN DEPENDENT PROTEIN KINASE ACTIVITY	84	-1.98	0.028

T CELL DIFFERENTIATION INVOLVED IN IMMUNE RESPONSE	51	-1.98	0.028
INFLAMMATORY RESPONSE TO WOUNDING	8	-1.98	0.028
G QUADRUPLEX DNA UNWINDING	7	-1.98	0.029
POSITIVE REGULATION OF AMYLOID BETA CLEARANCE	6	-1.98	0.029
COPPER ION TRANSPORT	12	-1.98	0.029
V D J RECOMBINATION	12	-1.98	0.029
PEPTIDYL TYROSINE MODIFICATION	281	-1.98	0.029
POSITIVE REGULATION OF LIPID LOCALIZATION	56	-1.98	0.029
AGING	233	-1.97	0.030
REGULATION OF SPINDLE ORGANIZATION	37	-1.97	0.030
NEGATIVE REGULATION OF CERAMIDE BIOSYNTHETIC PROCESS	5	-1.97	0.030
PYRIMIDINE DEOXYRIBONUCLEOTIDE METABOLIC PROCESS	19	-1.97	0.031
PROTEIN LOCALIZATION TO CELL CORTEX	11	-1.97	0.031
CELLULAR RESPONSE TO PROSTAGLANDIN STIMULUS	16	-1.97	0.031
POSITIVE REGULATION OF MAST CELL ACTIVATION	16	-1.97	0.031
ACUTE PHASE RESPONSE	18	-1.97	0.031
POSITIVE REGULATION OF NATURAL KILLER CELL MEDIATED IMMUNITY	11	-1.97	0.031
REGULATION OF ORGANELLE ASSEMBLY	162	-1.96	0.031
ORGANIC HYDROXY COMPOUND BIOSYNTHETIC PROCESS	178	-1.96	0.031
SPHINGOSINE METABOLIC PROCESS	8	-1.96	0.031
NADP METABOLIC PROCESS	30	-1.96	0.031
POSITIVE REGULATION OF RESPONSE TO DNA DAMAGE STIMULUS	87	-1.96	0.031
T CELL MEDIATED IMMUNITY	61	-1.96	0.032
SMALL GTPASE MEDIATED SIGNAL TRANSDUCTION	478	-1.96	0.032
G PROTEIN COUPLED PURINERGIC NUCLEOTIDE RECEPTOR SIGNALING PATHWAY	10	-1.96	0.032

POSITIVE REGULATION OF MACROPHAGE ACTIVATION	12	-1.96	0.032
STEROID METABOLIC PROCESS	202	-1.96	0.032
NEGATIVE REGULATION OF NEUROINFLAMMATORY RESPONSE	10	-1.96	0.032
NEGATIVE REGULATION OF DNA DEPENDENT DNA REPLICATION	18	-1.96	0.032
CELL CELL ADHESION MEDIATED BY INTEGRIN	14	-1.96	0.032
COLLAGEN FIBRIL ORGANIZATION	49	-1.96	0.032
POSITIVE REGULATION OF CYTOKINE PRODUCTION INVOLVED IN IMMUNE RESPONSE	38	-1.96	0.032
HISTONE H3 ACETYLATION	56	-1.96	0.032
POSITIVE REGULATION OF FAT CELL DIFFERENTIATION	51	-1.95	0.032
PYRIMIDINE NUCLEOTIDE METABOLIC PROCESS	52	-1.95	0.033
POSITIVE REGULATION OF NUCLEOBASE CONTAINING COMPOUND TRANSPORT	9	-1.95	0.033
RESPONSE TO ACIDIC PH	14	-1.95	0.033
WOUND HEALING INVOLVED IN INFLAMMATORY RESPONSE	5	-1.95	0.033
AMINO SUGAR METABOLIC PROCESS	29	-1.95	0.033
REGULATION OF ALPHA BETA T CELL DIFFERENTIATION	49	-1.95	0.033
LOW DENSITY LIPOPROTEIN PARTICLE RECEPTOR BIOSYNTHETIC PROCESS	9	-1.95	0.033
KINETOCHORE ASSEMBLY	11	-1.95	0.033
POSITIVE REGULATION OF PROTEIN LOCALIZATION TO NUCLEUS	67	-1.95	0.034
POSITIVE REGULATION OF GENE EXPRESSION EPIGENETIC	47	-1.94	0.034
HEAD DEVELOPMENT	544	-1.94	0.034
POSITIVE REGULATION OF SISTER CHROMATID COHESION	9	-1.94	0.034
SERTOLI CELL DEVELOPMENT	12	-1.94	0.034
REGULATION OF T CELL APOPTOTIC PROCESS	25	-1.94	0.034
RESPONSE TO XENOBIOTIC STIMULUS	156	-1.94	0.034

STEROID BIOSYNTHETIC PROCESS	131	-1.94	0.034
GLYCEROLIPID BIOSYNTHETIC PROCESS	206	-1.94	0.034
POSITIVE REGULATION OF ESTABLISHMENT OF PROTEIN LOCALIZATION	344	-1.94	0.034
REGULATION OF VIRAL LIFE CYCLE	96	-1.94	0.034
REGULATION OF INTEGRIN ACTIVATION	12	-1.94	0.034
CYTOKINE SECRETION INVOLVED IN IMMUNE RESPONSE	13	-1.94	0.035
RESPONSE TO PH	28	-1.94	0.035
NEGATIVE REGULATION OF LEUKOCYTE DEGRANULATION	9	-1.94	0.035
HEMOGLOBIN METABOLIC PROCESS	10	-1.94	0.035
DNA REPLICATION CHECKPOINT	12	-1.94	0.035
LYMPHOCYTE HOMEOSTASIS	46	-1.94	0.035
NEUTRAL LIPID BIOSYNTHETIC PROCESS	34	-1.93	0.035
REGULATION OF NATURAL KILLER CELL ACTIVATION	21	-1.93	0.035
NEGATIVE REGULATION OF EXTRINSIC APOPTOTIC SIGNALING PATHWAY VIA DEATH DOMAIN RECEPTORS	28	-1.93	0.036
RESPONSE TO DOPAMINE	42	-1.93	0.036
NEUTROPHIL EXTRAVASATION	12	-1.93	0.036
RESPONSE TO DSRNA	34	-1.93	0.036
NEGATIVE REGULATION OF REPRODUCTIVE PROCESS	32	-1.93	0.036
RNA PHOSPHODIESTER BOND HYDROLYSIS	123	-1.93	0.037
T HELPER 1 CELL DIFFERENTIATION	17	-1.93	0.037
REGULATION OF VIRAL TRANSCRIPTION	54	-1.93	0.037
REGULATION OF T CELL MEDIATED IMMUNITY	44	-1.93	0.037
COPPER ION IMPORT	5	-1.93	0.037
POLY N ACETYLLACTOSAMINE METABOLIC PROCESS	6	-1.93	0.037
REGULATION OF PROTEOLYSIS	512	-1.92	0.037
NUCLEAR PORE COMPLEX ASSEMBLY	9	-1.92	0.037
REGULATION OF PROTEIN LOCALIZATION TO NUCLEUS	104	-1.92	0.037

POSITIVE REGULATION OF ISOTYPE SWITCHING	19	-1.92	0.037
REGULATION OF FC RECEPTOR MEDIATED STIMULATORY SIGNALING PATHWAY	10	-1.92	0.038
REGULATION OF NIK NF KAPPAB SIGNALING	82	-1.92	0.038
EXTRINSIC APOPTOTIC SIGNALING PATHWAY VIA DEATH DOMAIN RECEPTORS	69	-1.92	0.038
REGULATION OF ENDOCYTOSIS	166	-1.92	0.038
DNA TOPOLOGICAL CHANGE	8	-1.92	0.038
REGULATION OF CHROMOSOME SEPARATION	51	-1.92	0.038
POSITIVE REGULATION OF CHEMOTAXIS	101	-1.92	0.038
REGULATION OF CD40 SIGNALING PATHWAY	5	-1.92	0.038
MACROPHAGE MIGRATION	32	-1.92	0.038
REGULATION OF MACROPHAGE MIGRATION	26	-1.92	0.038
REGULATION OF ANGIOTENSIN LEVELS IN BLOOD	7	-1.92	0.038
GLYCOSPHINGOLIPID METABOLIC PROCESS	52	-1.92	0.038
ZINC ION HOMEOSTASIS	19	-1.92	0.038
B CELL ACTIVATION INVOLVED IN IMMUNE RESPONSE	57	-1.91	0.039
NEUTROPHIL APOPTOTIC PROCESS	6	-1.91	0.039
PHOSPHOLIPID METABOLIC PROCESS	343	-1.91	0.039
NEGATIVE REGULATION OF TOLL LIKE RECEPTOR 4 SIGNALING PATHWAY	5	-1.91	0.039
THROMBIN ACTIVATED RECEPTOR SIGNALING PATHWAY	9	-1.91	0.040
REGULATION OF TYPE 2 IMMUNE RESPONSE	22	-1.91	0.040
PHOSPHORYLATION OF RNA POLYMERASE II C TERMINAL DOMAIN	11	-1.91	0.040
RESPONSE TO ANTIMETABOLITE	14	-1.91	0.040
SEROTONIN SECRETION	5	-1.91	0.040
DENDRITIC CELL ANTIGEN PROCESSING AND PRESENTATION	10	-1.91	0.040
PLATELET ACTIVATING FACTOR METABOLIC PROCESS	5	-1.91	0.040
CELLULAR RESPONSE TO PEPTIDE	298	-1.90	0.040
CELLULAR RESPONSE TO ALCOHOL	66	-1.90	0.041

NEGATIVE REGULATION OF MYELOID LEUKOCYTE MEDIATED IMMUNITY	9	-1.90	0.041
NEGATIVE REGULATION OF PROTEIN POLYMERIZATION	57	-1.90	0.042
SPHINGOLIPID METABOLIC PROCESS	119	-1.90	0.042
POSITIVE REGULATION OF T CELL MIGRATION	23	-1.89	0.043
ANTIBIOTIC METABOLIC PROCESS	63	-1.89	0.043
IONOTROPIC GLUTAMATE RECEPTOR SIGNALING PATHWAY	10	-1.89	0.043
CYTOKINE PRODUCTION INVOLVED IN INFLAMMATORY RESPONSE	28	-1.89	0.043
DENDRITIC CELL CYTOKINE PRODUCTION	12	-1.89	0.044
NEGATIVE REGULATION OF INTERLEUKIN 10 PRODUCTION	13	-1.89	0.044
REGULATION OF EXOCYTOSIS	153	-1.89	0.044
REGULATION OF PLASMINOGEN ACTIVATION	11	-1.89	0.044
POSITIVE REGULATION OF LYMPHOCYTE MIGRATION	26	-1.89	0.044
REGULATION OF LIPID METABOLIC PROCESS	325	-1.88	0.045
SECOND MESSENGER MEDIATED SIGNALING	274	-1.88	0.045
DNA TEMPLATED TRANSCRIPTION ELONGATION	101	-1.88	0.045
SPHINGOLIPID BIOSYNTHETIC PROCESS	75	-1.88	0.045
SPHINGOID METABOLIC PROCESS	11	-1.88	0.045
RESPONSE TO LIPOTEICHOIC ACID	9	-1.88	0.045
AUDITORY RECEPTOR CELL MORPHOGENESIS	10	-1.88	0.045
COFACTOR CATABOLIC PROCESS	36	-1.88	0.046
NCRNA TRANSCRIPTION	99	-1.88	0.046
LIPID CATABOLIC PROCESS	220	-1.88	0.046
ANION HOMEOSTASIS	37	-1.88	0.046
NEGATIVE REGULATION OF VIRAL LIFE CYCLE	44	-1.88	0.046
REGULATION OF CELL ADHESION MEDIATED BY INTEGRIN	39	-1.87	0.047
TUMOR NECROSIS FACTOR SECRETION	20	-1.87	0.047
LIPID HOMEOSTASIS	93	-1.87	0.048

NEUTROPHIL DIFFERENTIATION	8	-1.87	0.048
DNA METHYLATION	43	-1.87	0.048
REGULATION OF DNA DAMAGE RESPONSE SIGNAL TRANSDUCTION BY P53 CLASS MEDIATOR	32	-1.87	0.048
POSITIVE REGULATION OF DNA RECOMBINATION	31	-1.87	0.048
NEGATIVE REGULATION OF REGULATED SECRETORY PATHWAY	18	-1.87	0.048
REGULATION OF ENDOPLASMIC RETICULUM STRESS INDUCED INTRINSIC APOPTOTIC SIGNALING PATHWAY	29	-1.87	0.048
FEMALE MEIOTIC NUCLEAR DIVISION	22	-1.87	0.048
RESPONSE TO THYROID HORMONE	20	-1.87	0.049
SUPEROXIDE METABOLIC PROCESS	46	-1.86	0.049
NEGATIVE REGULATION OF CHROMOSOME ORGANIZATION	114	-1.86	0.049
NEUTROPHIL CLEARANCE	5	-1.86	0.049
COMPLEMENT ACTIVATION ALTERNATIVE PATHWAY	8	-1.86	0.049
PYRIMIDINE CONTAINING COMPOUND METABOLIC PROCESS	74	-1.86	0.049
REGULATION OF LEUKOCYTE CHEMOTAXIS	78	-1.86	0.049
INTERLEUKIN 2 PRODUCTION	51	-1.86	0.050
REGULATION OF HISTONE MODIFICATION	121	-1.86	0.050
ENDOPLASMIC RETICULUM TO GOLGI VESICLE MEDIATED TRANSPORT	166	-1.86	0.050

Table 3S. GO Bone Resorption (GO:0045453) genes ranked by GSEA [57] according to expression differences in hibernating phenotype comparing to summer active animals. Rank in gene list indicates position of the gene in the ranked list of all genes. Rank metric scores – defines position of the gene in the ranked list. Running enrichment score (ES) reflects the degree to which a gene set is overrepresented at the top or bottom of a ranked list of genes. Core enrichment - the subset of down-regulated genes that contributes most to the enrichment of bone resorption by under expressed genes (NES = -2.87, Table 1) is on the bottom of the ranked list.

Row	Gene Symbol	Rank In Gene List	Rank Metric Score	Running ES	Core Enrichment
1	PPARGC1B	585	6.552232	-0.02237	No
2	PDK4	641	5.952428	-0.00559	No
3	NOX4	1189	3.610009	-0.02515	No
4	PRKCA	2280	2.255146	-0.08481	No
5	S1PR1	2542	2.069265	-0.08325	No
6	P2RX7	2636	2.015344	-0.06928	No
7	ADRB2	2948	1.859373	-0.07142	No
8	TMEM64	3625	1.592993	-0.1005	No
9	DEF8	4562	1.317232	-0.14879	No
10	EGFR	4716	1.283268	-0.13925	No
11	NCDN	6173	1.016641	-0.22594	No
12	CTNNB1	6246	1.003771	-0.21042	No
13	LTBP3	6378	-1.01596	-0.19926	No
14	TRAF6	6927	-1.10003	-0.21889	No
15	NF1	7159	-1.14425	-0.21512	No
16	RAC1	7289	-1.16586	-0.20381	No
17	ATP6AP1	8964	-1.50259	-0.30659	No
18	GPR137	9395	-1.61668	-0.31751	**Yes**
19	PLEKHM1	9664	-1.70595	-0.31647	**Yes**
20	TNFRSF11B	9734	-1.72695	-0.30073	**Yes**
21	GPR137B	9922	-1.79158	-0.29371	**Yes**
22	TFRC	10131	-1.86873	-0.28823	**Yes**
23	TPP1	10182	-1.89307	-0.27109	**Yes**
24	LRRK1	10473	-2.01486	-0.27167	**Yes**

25	TNFRSF11A	11336	-2.63172	-0.31449	**Yes**
26	TMEM119	11369	-2.66523	-0.29602	**Yes**
27	CTSK	11440	-2.74643	-0.28036	**Yes**
28	IL7	11484	-2.79916	-0.2627	**Yes**
29	PTH1R	11630	-2.98418	-0.25258	**Yes**
30	CD38	11965	-3.52099	-0.25641	**Yes**
31	TNFAIP3	12123	-3.84348	-0.24717	**Yes**
32	CSF1R	12206	-4.03913	-0.23239	**Yes**
33	SRC	12215	-4.07613	-0.21215	**Yes**
34	RAB3D	12297	-4.35766	-0.19729	**Yes**
35	CSK	12398	-4.71337	-0.18384	**Yes**
36	UBASH3B	12601	-5.74487	-0.17793	**Yes**
37	TCIRG1	12643	-6.00869	-0.16012	**Yes**
38	SPP1	12655	-6.08426	-0.1401	**Yes**
39	BGLAP	12662	-6.12169	-0.11971	**Yes**
40	ACP5	12724	-6.47558	-0.10338	**Yes**
41	INPP5D	13126	-12.3923	-0.11216	**Yes**
42	ITGB3	13198	-14.65	-0.09657	**Yes**
43	SYK	13299	-17.8806	-0.08312	**Yes**
44	PTK2B	13409	-24.5551	-0.07034	**Yes**
45	CA2	13411	-24.66	-0.04958	**Yes**
46	ADAM8	13413	-24.8555	-0.02882	**Yes**
47	SNX10	13418	-25.5358	-0.00828	**Yes**
48	RAC2	13432	-28.2501	0.011594	**Yes**

Table 4S. GO Ossification (GO:0001503) genes ranked by GSEA [57] according to expression differences in hibernating phenotype comparing to summer active animals. Rank in gene list indicates position of the gene in the ranked list of all genes. Rank metric scores – defines position of the gene in the ranked list. Running enrichment score (ES) reflects the degree to which a gene set is overrepresented at the top or bottom of a ranked list of genes. Core enrichment - the subset of down-regulated genes that contributes most to the non-significant enrichment of ossification (NES = -1.45, FDR = 0.220, Table 1) by under expressed genes.

Row	Gene Symbol	Rank In Gene List	Rank Metric Score	Running ES	Core Enrichment
1	FZD9	85	44.41041	-0.00297	No
2	RYR1	86	44.37972	4.57E-04	No
3	COL2A1	109	40.31741	0.002228	No
4	MN1	216	22.24195	-0.00232	No
5	ACVR2B	363	10.97933	-0.00987	No
6	BMPR1B	366	10.95325	-0.0066	No
7	FHL2	424	9.08148	-0.00746	No
8	AXIN2	566	6.768892	-0.01464	No
9	ESRRA	575	6.679886	-0.01182	No
10	PPARGC1B	585	6.552232	-0.00907	No
11	SCX	612	6.290541	-0.0076	No
12	WNT11	652	5.886754	-0.00711	No
13	MEF2C	722	5.44419	-0.00887	No
14	CYR61	805	4.908345	-0.01161	No
15	TOB1	819	4.861364	-0.00917	No
16	TGFB2	846	4.711645	-0.0077	No
17	PAM16	888	4.517055	-0.00735	No
18	ATP5F1B	893	4.498354	-0.00423	No
19	KREMEN1	903	4.462406	-0.00148	No
20	MEF2D	942	4.307003	-9.16E-04	No
21	WNT10B	954	4.27075	0.001682	No
22	ZBTB16	959	4.249826	0.004806	No

23	WNT4	960	4.248253	0.00823	No
24	RDH14	1026	4.053619	0.006767	No
25	PDLIM7	1056	3.970432	0.008011	No
26	MYOC	1116	3.803393	0.006999	No
27	SBDS	1148	3.740005	0.008092	No
28	ID1	1253	3.461663	0.003696	No
29	IGFBP3	1284	3.391406	0.004865	No
30	PTN	1443	3.152027	-0.00359	No
31	LIMD1	1477	3.097736	-0.00265	No
32	RPL38	1478	3.096636	7.76E-04	No
33	PBX1	1491	3.081328	0.003298	No
34	THRA	1600	2.918083	-0.0014	No
35	OSR1	1668	2.832593	-0.00301	No
36	SOX8	1708	2.786111	-0.00252	No
37	ACVR2A	1725	2.765061	-2.99E-04	No
38	LGR4	1825	2.658684	-0.00432	No
39	COL11A1	1958	2.516644	-0.01082	No
40	ASPN	1963	2.51369	-0.0077	No
41	TOB2	1987	2.490378	-0.006	No
42	NELL1	2002	2.477811	-0.00363	No
43	BMPR1A	2020	2.464702	-0.00148	No
44	PTCH1	2120	2.374346	-0.0055	No
45	FBN2	2141	2.356641	-0.00358	No
46	CHRD	2198	2.319417	-0.00437	No
47	SFRP2	2223	2.29926	-0.00275	No
48	TEK	2326	2.223473	-0.007	No
49	FAT4	2336	2.215351	-0.00425	No
50	KLF10	2367	2.194227	-0.00308	No
51	CSGALNACT1	2372	2.19019	4.48E-05	No

52	PRKACA	2469	2.120383	-0.00375	No
53	HDAC8	2515	2.087945	-0.00371	No
54	S1PR1	2542	2.069265	-0.00224	No
55	P2RX7	2636	2.015344	-0.00581	No
56	FGFR3	2751	1.957225	-0.01096	No
57	DHRS3	2794	1.931193	-0.01069	No
58	FSTL3	2831	1.912486	-0.00997	No
59	SMAD1	2842	1.907486	-0.0073	No
60	ZHX3	2924	1.869929	-0.00997	No
61	ADRB2	2948	1.859373	-0.00827	No
62	GDF5	2960	1.855842	-0.00567	No
63	WWTR1	3006	1.83151	-0.00563	No
64	ACVR1	3021	1.824415	-0.00326	No
65	ID4	3042	1.814729	-0.00134	No
66	GTPBP4	3049	1.810515	0.001633	No
67	CTNNBIP1	3096	1.789065	0.001598	No
68	DHH	3138	1.770452	0.00194	No
69	FGF18	3143	1.767204	0.005064	No
70	PHB	3258	1.727516	-8.45E-05	No
71	BMP6	3301	1.710551	1.82E-04	No
72	PSMC2	3430	1.663264	-0.00602	No
73	CEBPB	3440	1.658558	-0.00327	No
74	NBR1	3444	1.657959	-7.21E-05	No
75	STC1	3448	1.657632	0.003127	No
76	RPS15	3478	1.645544	0.004371	No
77	JAG1	3484	1.643701	0.007419	No
78	LRP5	3603	1.599047	0.001971	No
79	TMEM64	3625	1.592993	0.003816	No
80	EPHA2	3636	1.590183	0.006489	No

81	MAPK3	3642	1.588164	0.009537	No
82	BMP5	3643	1.587673	0.012962	No
83	BMP4	3670	1.5805	0.014432	No
84	NPR2	3707	1.56776	0.015149	No
85	ERCC2	3778	1.540374	0.01331	No
86	SUFU	3886	1.509224	0.008688	No
87	ID3	3901	1.504271	0.01106	No
88	PTK2	3991	1.477389	0.007792	No
89	HSPE1	4149	1.432703	-5.90E-04	No
90	SOX9	4230	1.412182	-0.00318	No
91	TAPT1	4244	1.407326	-7.34E-04	No
92	BMPR2	4348	1.376451	-0.00505	No
93	MYBBP1A	4416	1.356628	-0.00667	No
94	BCOR	4429	1.351345	-0.00415	No
95	RXRA	4439	1.349107	-0.0014	No
96	INPPL1	4496	1.335281	-0.00218	No
97	NAB2	4514	1.331397	-3.86E-05	No
98	PLXNB1	4518	1.330342	0.00316	No
99	ATRAID	4537	1.324868	0.005232	No
100	SMAD6	4669	1.295229	-0.00119	No
101	EGFR	4716	1.283268	-0.00123	No
102	PIAS2	4749	1.275592	-2.11E-04	No
103	IGFBP5	4765	1.272363	0.002085	No
104	HOXA2	4922	1.239201	-0.00622	No
105	SMURF1	4946	1.23498	-0.00453	No
106	GLI1	4969	1.231047	-0.00276	No
107	DCHS1	4975	1.230464	2.93E-04	No
108	TUFT1	5012	1.220605	0.001011	No
109	CTGF	5146	1.192969	-0.00557	No

110	SMAD3	5216	1.180675	-0.00733	No
111	AKT1	5232	1.177399	-0.00503	No
112	PKDCC	5247	1.17457	-0.00266	No
113	FBXL15	5264	1.169848	-4.40E-04	No
114	CLEC3B	5327	1.159333	-0.00168	No
115	UFL1	5344	1.156117	5.43E-04	No
116	HDAC7	5348	1.155275	0.003742	No
117	RIOX1	5409	1.14384	0.002655	No
118	TGFB3	5603	1.111189	-0.00843	No
119	MMP2	5630	1.107567	-0.00696	No
120	OSR2	5708	1.093254	-0.00933	No
121	EXT1	5763	1.085759	-0.00997	No
122	BCL2	5823	1.077137	-0.01098	No
123	LRP6	5857	1.071088	-0.01003	No
124	CCDC47	5869	1.069238	-0.00744	No
125	SKI	5903	1.063881	-0.00649	No
126	IMPAD1	5967	1.052245	-0.00781	No
127	ZBTB40	6040	1.039423	-0.0098	No
128	IGSF10	6098	1.0296	-0.01066	No
129	EGR2	6100	1.029376	-0.00731	No
130	ANKH	6135	1.024233	-0.00644	No
131	GLI2	6159	1.018865	-0.00475	No
132	CTNNB1	6246	1.003771	-0.00779	No
133	IARS	6316	-1.00618	-0.00955	No
134	MMP14	6371	-1.01441	-0.01019	No
135	RRAS2	6376	-1.01505	-0.00707	No
136	PHEX	6385	-1.01688	-0.00424	No
137	GLI3	6406	-1.02026	-0.00232	No
138	PEX7	6453	-1.02819	-0.00236	No

139	SEMA7A	6642	-1.05399	-0.01307	No
140	NPNT	6737	-1.06893	-0.01671	No
141	TRAF6	6927	-1.10003	-0.0275	No
142	SYNCRIP	6967	-1.10623	-0.02701	No
143	ANO6	7058	-1.12188	-0.03035	No
144	GPC3	7074	-1.12633	-0.02806	No
145	HDAC4	7076	-1.12665	-0.02471	No
146	NF1	7159	-1.14425	-0.02745	No
147	SHOX2	7205	-1.1501	-0.02741	No
148	SNAI2	7356	-1.17967	-0.03526	No
149	FGF9	7524	-1.21086	-0.0444	No
150	ROR2	7559	-1.21653	-0.04353	No
151	TWSG1	7573	-1.21845	-0.04108	No
152	NAB1	7644	-1.23199	-0.04292	No
153	CITED1	7671	-1.23646	-0.04145	No
154	SH3PXD2B	7677	-1.23731	-0.0384	No
155	GJA1	7752	-1.24942	-0.04054	No
156	GPLD1	7775	-1.25335	-0.03877	No
157	SLC26A2	7840	-1.26459	-0.04016	No
158	NOCT	7969	-1.28608	-0.04636	No
159	SMO	7971	-1.2868	-0.04301	No
160	FBL	8068	-1.30509	-0.04681	No
161	ENPP1	8074	-1.30577	-0.04376	No
162	SMAD5	8085	-1.30737	-0.04108	No
163	ACHE	8264	-1.34323	-0.05105	No
164	WNT5A	8276	-1.34493	-0.04845	No
165	CHRDL1	8365	-1.3665	-0.05164	**Yes**
166	DHX36	8394	-1.37412	-0.05032	**Yes**
167	MAPK1	8450	-1.38569	-0.05103	**Yes**

168	SNRNP200	8478	-1.39221	-0.04964	**Yes**
169	DDR2	8488	-1.39438	-0.04689	**Yes**
170	IL6ST	8567	-1.4104	-0.04933	**Yes**
171	TP53INP2	8593	-1.41609	-0.04779	**Yes**
172	DDX21	8619	-1.42139	-0.04624	**Yes**
173	IGF2	8653	-1.43059	-0.0453	**Yes**
174	EXT2	8688	-1.4389	-0.04443	**Yes**
175	SEMA4D	8769	-1.45704	-0.04702	**Yes**
176	TRPM4	8782	-1.4596	-0.0445	**Yes**
177	LRP4	8838	-1.47257	-0.04521	**Yes**
178	ASF1A	8864	-1.47983	-0.04367	**Yes**
179	FOXC1	8896	-1.48823	-0.04257	**Yes**
180	BMP7	8931	-1.49446	-0.04171	**Yes**
181	KAZALD1	8961	-1.50175	-0.04046	**Yes**
182	ATP6AP1	8964	-1.50259	-0.03719	**Yes**
183	DDX5	9001	-1.50868	-0.03647	**Yes**
184	TXLNG	9089	-1.53334	-0.03959	**Yes**
185	RSL1D1	9115	-1.53822	-0.03804	**Yes**
186	ID2	9295	-1.58467	-0.04808	**Yes**
187	FOXC2	9296	-1.5858	-0.04465	**Yes**
188	BMP2	9328	-1.59553	-0.04356	**Yes**
189	SNAI1	9368	-1.60919	-0.04307	**Yes**
190	SMOC1	9379	-1.61196	-0.0404	**Yes**
191	RBMX	9396	-1.6169	-0.03817	**Yes**
192	FZD1	9420	-1.62758	-0.03648	**Yes**
193	HIF1A	9433	-1.63337	-0.03396	**Yes**
194	SP3	9439	-1.63569	-0.03091	**Yes**
195	GREM1	9447	-1.63799	-0.02801	**Yes**
196	HNRNPU	9505	-1.65709	-0.02887	**Yes**

197	CAT	9543	-1.66617	-0.02823	**Yes**
198	CLEC11A	9551	-1.66846	-0.02533	**Yes**
199	HSD17B4	9625	-1.69189	-0.0274	**Yes**
200	SFRP1	9662	-1.70569	-0.02668	**Yes**
201	TNC	9705	-1.71851	-0.02641	**Yes**
202	PHOSPHO1	9806	-1.75118	-0.03051	**Yes**
203	INTU	9831	-1.75892	-0.02889	**Yes**
204	SLC8A1	9849	-1.7648	-0.02674	**Yes**
205	TWIST1	9901	-1.78393	-0.02715	**Yes**
206	ITGA11	9912	-1.78786	-0.02448	**Yes**
207	RBPJ	9954	-1.80121	-0.02414	**Yes**
208	PRKD1	9971	-1.80762	-0.02192	**Yes**
209	GPM6B	10012	-1.82414	-0.0215	**Yes**
210	MEN1	10017	-1.82559	-0.01838	**Yes**
211	CSF1	10033	-1.83093	-0.01608	**Yes**
212	TPM4	10046	-1.83434	-0.01356	**Yes**
213	RHOA	10142	-1.87576	-0.01728	**Yes**
214	NIPBL	10157	-1.8826	-0.0149	**Yes**
215	FGFR2	10173	-1.88761	-0.01261	**Yes**
216	ALOX15	10270	-1.93085	-0.0164	**Yes**
217	GDF10	10293	-1.93716	-0.01463	**Yes**
218	BCAP29	10389	-1.97468	-0.01835	**Yes**
219	COL6A1	10449	-2.00236	-0.01936	**Yes**
220	ALYREF	10539	-2.05807	-0.02263	**Yes**
221	CDH11	10551	-2.06147	-0.02003	**Yes**
222	HNRNPC	10552	-2.06197	-0.01661	**Yes**
223	ECM1	10582	-2.07768	-0.01537	**Yes**
224	GDPD2	10609	-2.09343	-0.0139	**Yes**
225	OMD	10676	-2.13253	-0.01543	**Yes**

226	CBFB	10680	-2.13583	-0.01224	**Yes**
227	CHSY1	10687	-2.14004	-0.00926	**Yes**
228	MMP16	10704	-2.14879	-0.00704	**Yes**
229	PTGER4	10742	-2.16505	-0.0064	**Yes**
230	IGF1	10806	-2.20048	-0.00771	**Yes**
231	DHX9	10821	-2.21088	-0.00534	**Yes**
232	EIF2AK3	10826	-2.21263	-0.00222	**Yes**
233	ADAR	10920	-2.28006	-0.00578	**Yes**
234	WWOX	10933	-2.28466	-0.00326	**Yes**
235	SND1	10958	-2.30432	-0.00164	**Yes**
236	TGFB1	11058	-2.38282	-0.00566	**Yes**
237	THBS3	11128	-2.44351	-0.00743	**Yes**
238	CEBPA	11138	-2.45531	-0.00468	**Yes**
239	RRBP1	11155	-2.47157	-0.00246	**Yes**
240	CTHRC1	11160	-2.47388	6.66E-04	**Yes**
241	SORT1	11290	-2.58218	-0.00561	**Yes**
242	MGP	11316	-2.61021	-0.00406	**Yes**
243	TNFRSF11A	11336	-2.63172	-0.00207	**Yes**
244	LEP	11353	-2.64546	1.52E-04	**Yes**
245	CLTC	11355	-2.64689	0.003502	**Yes**
246	TMEM119	11369	-2.66523	0.005949	**Yes**
247	BMP1	11413	-2.71841	0.00614	**Yes**
248	MAPK14	11416	-2.72048	0.009414	**Yes**
249	SP7	11425	-2.73138	0.012237	**Yes**
250	CTSK	11440	-2.74643	0.014609	**Yes**
251	MRC2	11473	-2.78439	0.015628	**Yes**
252	SBNO2	11531	-2.85679	0.014766	**Yes**
253	DKK1	11540	-2.87068	0.017589	**Yes**
254	DNAJC13	11584	-2.91538	0.01778	**Yes**

255	PTH1R	11630	-2.98418	0.017821	**Yes**
256	SPARC	11641	-2.99719	0.020493	**Yes**
257	ILK	11702	-3.0779	0.019406	**Yes**
258	SUCO	11730	-3.11323	0.0208	**Yes**
259	BMP2K	11736	-3.12884	0.023849	**Yes**
260	COL5A2	11737	-3.12982	0.027274	**Yes**
261	HGF	11785	-3.20028	0.027164	**Yes**
262	IL6R	11806	-3.23728	0.029085	**Yes**
263	CREB3L1	11860	-3.34039	0.028524	**Yes**
264	COL1A1	11947	-3.4877	0.025481	**Yes**
265	TWIST2	12016	-3.60017	0.023792	**Yes**
266	RANBP3L	12095	-3.78213	0.021351	**Yes**
267	OSTF1	12177	-3.95156	0.018685	**Yes**
268	MINPP1	12207	-4.04056	0.019929	**Yes**
269	VCAN	12360	-4.56844	0.011923	**Yes**
270	PTGS2	12365	-4.58221	0.015047	**Yes**
271	LEF1	12372	-4.60697	0.018021	**Yes**
272	RUNX2	12425	-4.82703	0.017535	**Yes**
273	FIGNL1	12430	-4.84172	0.020659	**Yes**
274	CLIC1	12500	-5.2312	0.018895	**Yes**
275	SATB2	12531	-5.35763	0.020063	**Yes**
276	ALPL	12544	-5.44	0.022586	**Yes**
277	IFITM5	12594	-5.7228	0.022325	**Yes**
278	RUNX1	12632	-5.9808	0.022968	**Yes**
279	SPP1	12655	-6.08426	0.024738	**Yes**
280	BGLAP	12662	-6.12169	0.027711	**Yes**
281	RFLNB	12716	-6.43077	0.027151	**Yes**
282	ISG15	12735	-6.52854	0.029222	**Yes**
283	RUNX3	12818	-7.24361	0.02648	**Yes**

284	GPNMB	12846	-7.50485	0.027874	**Yes**
285	RASSF2	12932	-8.61452	0.024907	**Yes**
286	MMP9	13227	-15.4625	0.006223	**Yes**
287	CDK6	13277	-17.1632	0.005963	**Yes**
288	PTK2B	13409	-24.5551	-4.64E-04	**Yes**
289	FASN	13434	-28.3571	0.001156	**Yes**
290	SRGN	13497	-36.332	-8.14E-05	**Yes**
291	LTF	13526	-45.7429	0.001238	**Yes**
292	GATA1	13575	-111.062	0.001053	**Yes**
